# Difunctionalization of bicyclo[1.1.0]butanes enabled by merging C−C cleavage and ruthenium-catalysed remote C−H activation

**DOI:** 10.1038/s44160-025-00745-3

**Published:** 2025-02-17

**Authors:** Shan Chen, Zhimin Xu, Binbin Yuan, Xue-Ya Gou, Lutz Ackermann

**Affiliations:** Wöhler-Research Institute for Sustainable Chemistry, Georg-August-Universität Göttingen, Göttingen, Germany

**Keywords:** Synthetic chemistry methodology, Homogeneous catalysis

## Abstract

The high fraction of *sp*^3^-hybridized carbon atom (F*sp*^3^) character of cyclobutane derivatives renders them as highly promising bioisosteres for otherwise typically flat arenes. Here, to address the current needs in medicinal chemistry for F*sp*^3^-rich molecules, we disclose a distinct strategy that exploits the merger of C–C scission in bicyclo[1.1.0]butanes (BCBs) with ruthenium-catalysed remote C−H functionalization of heteroarenes, affording densely substituted cyclobutanes in a chemo-controlled manner. This approach enabled the rapid and efficient synthesis of versatile tri- and tetrasubstituted cyclobutanes by coupling a wide range of mono- or disubstituted BCBs with heteroarenes and alkyl halides under mild reaction conditions, featuring ample substrate scope. The C–C/C–H functionalization was ensured by a multifunctional ruthenium(II) catalyst that enabled ruthenacycle-mediated halogen-atom transfer (Ru-XAT), as well as the selective functionalization of BCBs by strain release. Experimental and computational mechanistic studies unravelled a multi-catalysis manifold, while the C–H/C–C functionalization strategy allowed for telescoping late-stage modification.

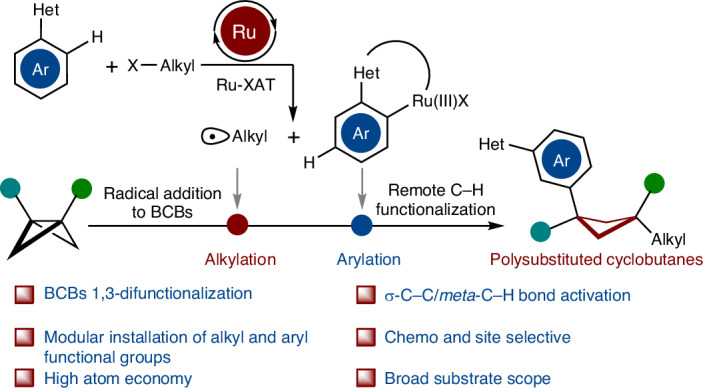

## Abstract (web summary)

The high fraction of *sp*^3^-hybridized carbon atoms (F*sp*^3^) in cyclobutanes makes them promising bioisosteres for arenes. Now by combining bicyclo[1.1.0]butane C–C activation with remote *meta*-C–H functionalization, via a ruthenacycle-mediated halogen-atom transfer process, densely decorated F*sp*^3^-rich cyclobutanes can be formed in a single step with high chemo- and position selectivity.

## Main

**Fig. 1:**
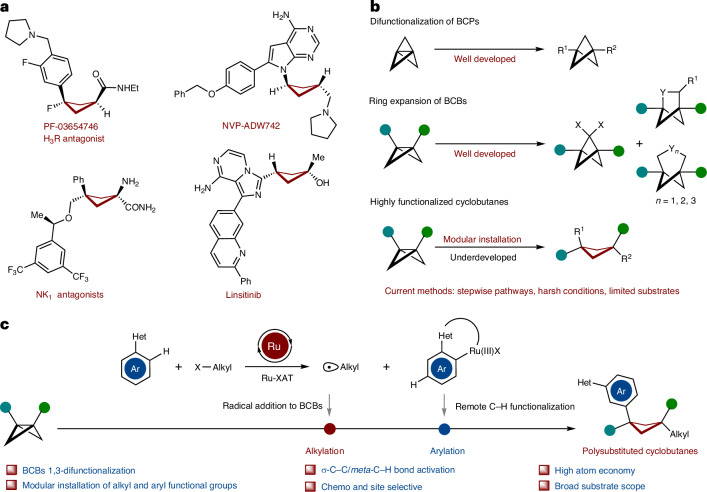
Design blueprint for the difunctionalization of BCBs to access 1,1,3-trisubstituted and 1,1,3,3-tetrasubstituted cyclobutanes enabled by remote C–H activation. **a**, Selected drug molecules containing 1,3-difunctionalized cyclobutane skeleton. **b**, Current strategies^50–54^ for the synthesis of highly functionalized cyclobutanes via strain release. **c**, Our hypothesis on the 1,3-difunctionalization of BCBs by remote C−H activation to access valuable 1,1,3-trisubstituted and 1,1,3,3-tetrasubstituted cyclobutanes via a Ru-XAT process. Ar, aryl.; Het, heteroarenes.

In the quest of ‘escaping from flatland’^1–8^, highly functionalized cyclobutanes with unique puckered linear geometry have received increasing attention in medicinal chemistry. The fraction of *sp*^3^-hybridized carbon atoms (F*sp*^3^) was identified as a key descriptor of drug likeness, and the increased F*sp*^3^ in cyclobutanes renders them as a privileged motif for isosteres in drug design^9^. The incorporation of cyclobutane scaffolds often enhances the physicochemical and pharmacokinetic properties of drug molecules. In contrast to usually flat arene rings, polysubstituted cyclobutanes with high F*sp*^3^ can provide, among others, improved solubility due to their nonplanar substituent vectors. Indeed, several pharmaceuticals featuring 1,3-bifunctional cyclobutanes have been clinically tested, such as PF-03654746 (ref. ^10^), NVP-ADW 742 (ref. ^11^), NK1 selective antagonists^12^ and Linsitinib^13^, translating into a strong need for innovative cyclobutane syntheses (Fig. 1a). However, in contrast to rather well-established bifunctionalization of bicyclo[1.1.0]pentanes (BCPs)^14–18^ and ring expansions of bicyclo[1.1.0]butanes (BCBs)^19–41^ (Fig. 1b), strategies to access structurally complex cyclobutanes are, unfortunately, scarce. Thus far, these syntheses are limited to radical or nucleophilic additions to BCBs, typically resulting in mono- or disubstituted cyclobutanes^42–49^, with only few examples for densely substituted cyclobutanes^50–54^. Furthermore, the aforementioned transformations are largely limited to rather harsh conditions and elements of prefunctionalization on the coupling partner, while the merger of C–C activations^55–60^ of BCBs with remote C–H activation^61^ has thus far proven elusive. To address these current topical needs, we have now developed the merger of BCBs C–C functionalization with remote C–H functionalization^62–73^ by a single, yet powerful, ruthenium(II) catalyst. Salient features of our findings include (1) versatile 1,3-difunctionalization of BCBs for tri- and tetrasubstituted F*sp*^3^-rich cyclobutanes; (2) a single ruthenium complex for a multi-catalysis manifold including an efficient ruthenacycle-mediated halogen-atom transfer (Ru-XAT) process, C–C scission and *meta*-C–H functionalization; (3) exceedingly mild reaction conditions and (4) outstanding chemo- and site selectivities (Fig. 1c).

## Results and discussion

### Optimization studies

We initiated our studies on BCBs 1,3-difunctionalization through remote C–H activation, with benzyl-bicyclo[1.1.0]butane-1-carboxylate (**1a**), 2-phenylpyridine (**2a**) and ethyl-2-bromo-2,2-difluoroacetate (**3a**) as the model substrates (Table 1). We were pleased to find that the desired product **4** was efficiently obtained in 75% isolated yield with Ru(O_2_CMes)_2_(*p*-cymene) as the catalyst and P(4-CF_3_C_6_H_4_)_3_ (Table 1, entry 1). Next, a series of phosphines, as well as alternative ligands such as bipyridine (bpy) and N-heterocyclic carbene (NHC), were tested, and P(4-CF_3_C_6_H_4_)_3_ was found to be superior (Table 1, entries 2 and 3). Ru(OAc)_2_(*p*-cymene) gave the desired product **4** with 52% yield, while [RuCl_2_(*p*-cymene)]_2_ was ineffective (Table 1, entry 4), highlighting the importance of carboxylate assistance in the C–H ruthenation^74^. [Ru(^*t*^BuCN)_5_(H_2_O)](BF_4_)_2_^75^ as a precatalyst gave inferior results (Table 1, entry 5). Control experiments revealed the crucial role of the ruthenium catalyst and the phosphine ligand for the BCBs C–C cleavage difunctionalization (Table 1, entry 6).

**Table 1:** Optimization of the reaction parameters


Entry	Variation from the standard conditions^a^	Yield (%)^b^
1	None	75
2	PPh_3_/P[3,5-(CF_3_)_2_C_6_H_3_]_3_/P(4-OMeC_6_H_4_)_3_	54/34/43
3	bpy/NHC as ligand^c^	0/trace
4	[Ru(OAc)_2_(*p*-cymene)]/[RuCl_2_(*p*-cymene)]_2_	52/5
5	[Ru(^*t*^BuCN)_5_(H_2_O)](BF_4_)_2_	21^d^/ 30
6	No catalyst/no ligand	0/0

^a^Reaction conditions: **1a** (0.3 mmol), **2a** (3.0 equiv.), **3a** (3.0 equiv.), [Ru] (10 mol%), ligand (10 mol%), Na_2_CO_3_ (2.0 equiv.), 1,4-dioxane (2.0 ml), *T* = 65 °C, *t* = 24 h.

^b^Yield of isolated products.

^c^bpy, 2,2′-bipyridine, NHC ligand used 1,3-bis(2,6-diisopropylphenyl)imidazolium chloride.

^d^*T* = 50 °C.

Ac, acetyl; Bn, benzyl; Mes, mesityl; 2-Py, 2-pyridyl.

**Fig. 2:**
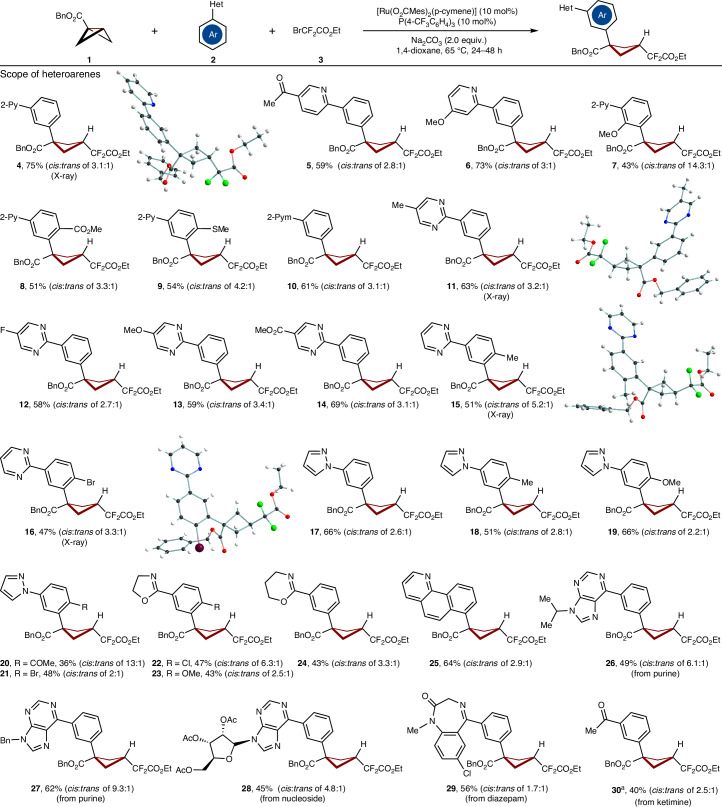
Scope of heteroarenes. Reaction conditions: **1** (0.3 mmol), **2** (3.0 equiv.), **3** (3.0 equiv.), [Ru(O_2_CMes)_2_(*p*-cymene)] (10 mol%), P(4-CF_3_C_6_H_4_)_3_ (10 mol%), Na_2_CO_3_ (2.0 equiv.), 1,4-dioxane (2.0 ml), 65 °C, 24–48 h. All yields are isolated yields. The ratios of the diastereomers (*cis* and *trans*) were determined by ^1^H-NMR spectroscopy or isolated yield. ^a^Work-up with 3 N HCl and stirring for a further 3 h. Note that the presented structures are the major isomers. Bn, benzyl; Mes, mesityl; Py, pyridyl; Pym, pyrimidinyl.

With the optimized reaction conditions in hand, we subsequently evaluated the viable substrate scope for the bifunctionalization of BCBs **1** with differently substituted heteroarenes **2** (Fig. 2). Arenes with distinct electronic features and substituents, such as fluorine, thioether, bromine, ester and keto groups, were fully tolerated by the versatile catalyst (**5**–**29**). Transformable pyrazoles, ketimine and oxazolines could be employed to guarantee *meta*-selectivity (**17**–**24**, **30**). Drug-relevant motifs, such as diazepam, purines and nucleoside proved to be viable for the ruthenium-catalysed BCBs difunctionalization (**26**–**29**).

**Fig. 3:**
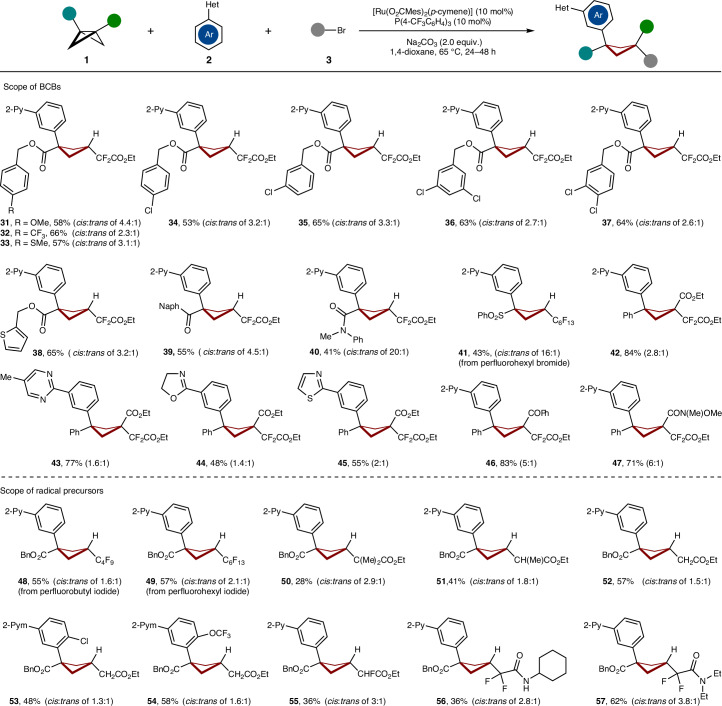
Scope of BCBs and radical precursors. Reaction conditions: **1** (0.3 mmol), **2** (3.0 equiv.), **3** (3.0 equiv.), [Ru(O_2_CMes)_2_(*p*-cymene)] (10 mol%), P(4-CF_3_C_6_H_4_)_3_ (10 mol%), Na_2_CO_3_ (2.0 equiv.), 1,4-dioxane (2.0 mL), 65 °C, 24–48 h. All yields are isolated yields. The ratios of the diastereomers were determined by ^1^H-NMR spectroscopy or isolated yields. Note that the presented structures are the major isomers. In the case of the 1,1,3-trisubstituted cyclobutanes, the *cis* structure is the major, while for the 1,1,3,3-tetrasubstituted cyclobutanes, the (1*r*,3*r*) structure is the major, which was confirmed by X-ray crystallographic analysis (see the X-ray structure of compound **62** in Supplementary Data 6 for details). Naph, 2-naphthyl group.

A series of substituted BCB esters **1** furnished the 1,1,3-trisubstituted cyclobutanes **31**–**37** (Fig. 3). Thus, BCBs featuring sensitive functional groups, including ester, thiophene, ketone, amide and sulfone (**38**–**41**) were efficiently converted to desired products. Furthermore, disubstituted BCBs were identified as amenable substrates (**42**–**47**). Here, disubstituted BCBs favoured to form a benzylic radical rather than a tertiary radical, chemo-selectively delivering diarylcyclobutane motifs. Likewise, a wide range of alkyl halides, such as perfluoroalkane halides, fluorine-free alkyl bromide, monofluoroalkyl bromide and difluoroalkyl amides were tolerated to give the desired cyclobutanes **48**–**57**.

### Gram-scale and late-stage derivatization

**Fig. 4:**
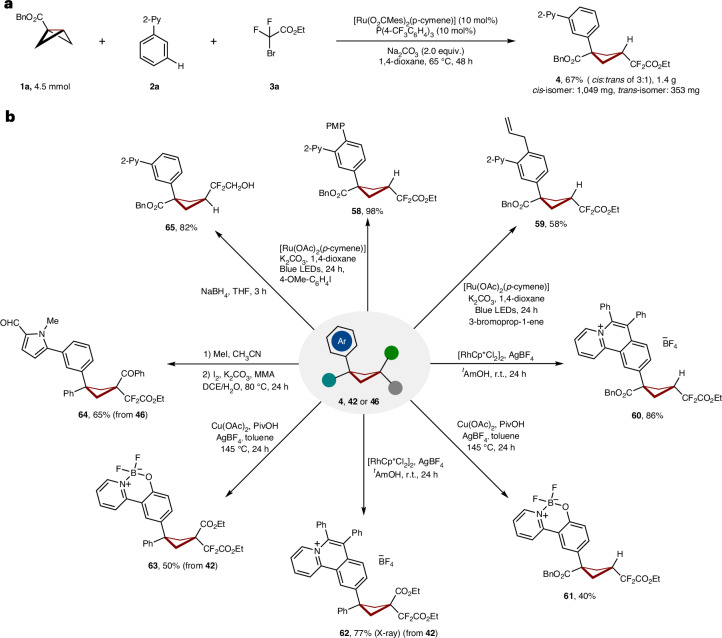
Gram-scale and late-stage derivatization. **a**, Gram-scale reaction gave the desired product **4** in high yield. **b**, Various downstream functionalization of products **4**, **42** or **46**. Cp*, 1,2,3,4,5-pentamethylcyclopentadienyl; DCE, 1,2-dichloroethane; LED, light emitting diode; MMA, methyl methacrylate; Piv, pivaloyl; PMP, *p*-methoxyphenyl; r.t., room temperature; THF, tetrahydrofuran.

To demonstrate the practical utility of our BCBs C–C scission/remote activation strategy, cyclobutane **4** was prepared at gram scale with comparable efficacy (Fig. 4a). The site selectivity ensured that pyrimidine, oxazoline and ketimine (vide infra) as well as pyridine and pyrazole were efficiently diversified, extending the viable portfolio (Fig. 4b). Further, a triple activation manifold proved viable in terms of ruthenium(II)-catalysed C−C/*meta*-C−H and *ortho*-photo-induced C−H activation (**58** and **59**).

### DFT calculation and mechanism studies

**Fig. 5:**
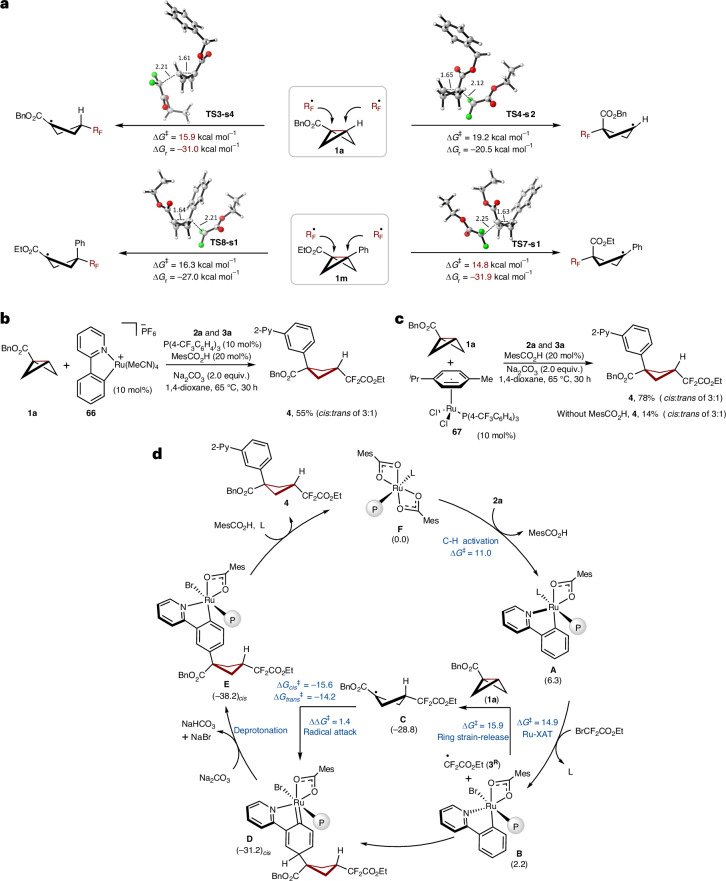
DFT calculation and mechanism studies. **a**, Computed relative Gibbs free energies (Δ*G*_338.15_) in kcal mol^−1^ for radical attack at the monosubstituted BCB **1a** and disubstituted BCB **1m** were conducted at the B3LYP-D3BJ/def2-TZVP-SMD(1,4-dioxane)//PBE0-D3BJ/def2-SVP level of theory. **b**, *p*-Cymene-free ruthenacycle complex **66** as the catalyst. **c**, Carboxylate-free ruthenium(II) phosphine complex **67** as the catalyst. **d**, Proposed catalytic cycle associated with relative Gibbs free energies (Δ*G*_338.15_) in kcal mol^−1^ based on our mechanism studies and DFT calculation. RF, difluoroalkyl; P, P(4-CF_3_C_6_H_4_)_3_; L, 1,4-dioxane, **2a** and so on.

To gain insights into the reaction mechanism, the site selectivity of radical addition at the two possible BCB sites was probed by means of density functional theory (DFT) calculations (Fig. 5a; see Supplementary Figs. 1, 2, 7 and 8 for details). In the case of monosubstituted BCB **1a**, the difluoroalkyl radical preferentially attacked at the unsubstituted carbon, leading to the formation of a more thermodynamically stable tertiary radical. The shorter bridge C−C distance (1.61 Å) and the longer C−C_RF_ distance (2.21 Å) in **TS3-s4**, compared with **TS4-s2**, indicated that the radical attack at the unsubstituted site proceeded through an earlier transition state that structurally resembled the starting BCB **1a**. In the case of radical addition to the disubstituted BCB **1m**, the ester-substituted site is favoured, resulting in the formation of thermodynamically stable benzylic radical. During the cleavage of the *σ*-bridge bond in disubstituted BCB **1m,** **TS7-s1** exhibited an earlier transition state characteristic, evidenced by the slightly elongated C–C bridge distance of BCB **1a** (1.63 Å in **TS7-s1** and 1.64 Å in **TS8-s1**) and the relatively larger C–C distances between the BCB **1m** and the difluoroalkyl radical (2.25 Å in **TS7-s1** and 2.21 Å in **TS8-s1**). The presynthesized *p*-cymene-free ruthenacycle **66** yielded the desired product **4** in the presence of MesCO_2_H and phosphine ligand (Fig. 5b). The control experiment without a phosphine ligand as well as with alternative ligands, such as bpy or NHC, in the optimization table failed to provide the desired product, highlighting the essential role of the phosphine ligand assistance^76–79^. The key role of the phosphine ligand was further demonstrated by attempting this transformation with carboxylate-free ruthenium(II) phosphine complex **67**, yielding the desired product in high yield when MesCO_2_H was added (Fig. 5c). The carboxylate-free ruthenium(II) phosphine complex **67** could afford the desired product only in 14% yield even in the absence of MesCO₂H (Fig. 5c). Additionally, radical trapping experiments validated the radical mechanism (see Supplementary Data 7.1.1 for details). On the basis of our mechanism and computational findings, a plausible catalytic cycle is put forwards in Fig. 5d, featuring ruthenacycle **A** in a ruthenacycle-mediated XAT process to furnish ruthenium(III) intermediate **B** with an energy barrier of 14.9 kcal mol^−1^. The thus formed alkyl radical **3**^**R**^ attacks the BCB **1a** at the unsubstituted site to induce strain-release C–C scission with an energy barrier of 15.9 kcal mol^−1^, forming the tertiary radical **C**. Species **C** reacts at the ruthena(III)cycle **B** *para* to the C_Ar_–Ru bond, delivering the stabilized singlet metallacycle **D**. Here, the formation of the *cis* product is favoured over the *trans* product by 1.4 kcal mol^−1^. The subsequent rearomatization and proto-demetallation releases the desired cyclobutane **4**, thereby regenerating the catalytically active ruthenium(II) species **F**.

## Conclusions

We have achieved the merger of BCBs C–C activation with remote *meta*-C–H functionalization by a multipotent ruthenium catalyst. The double activation proceeded in a highly chemo- and position-selective fashion and provided access to densely decorated F*sp*^3^-rich cyclobutanes in a single step. Mechanistic studies were suggestive of a Ru-XAT process enabling chemo-selective BCBs opening and *meta*-diversification.

## Methods

### General methods for 1,3-difunctionalization of BCBs enabled by ruthenium-catalysed remote C–H activation

The general procedure for 1,3-difunctionalization of BCBs was as follows: [Ru(O_2_CMes)_2_(*p*-cymene)] (16.8 mg, 10.0 mol%), P(4-CF_3_C_6_H_4_)_3_ (14.0 mg, 10.0 mol%), Na_2_CO_3_ (64 mg, 0.6 mmol, 2.0 equiv.), 1,4-dioxane (2.0 ml), **1** (0.3 mmol, 1.0 equiv.), **2** (0.9 mmol, 3.0 equiv.) and **3** (0.9 mmol, 3.0 equiv.) were added into an oven-dried 20 ml pressure tube. The reaction mixture was stirred at 65 °C for 24–48 h. After cooling to ambient temperature, the mixture was purified by column chromatography on silica gel to afford the corresponding cyclobutanes **4**–**57**.

## Supplementary information

Supplementary Information Supplementary Figs. 1–18, Tables 1–7 and experimental procedures, products characterization, late-stage derivatization, mechanism studies, computational details and NMR spectra.

Supplementary Data 1 Cartesian coordinates for all the calculated structures.

Supplementary Data 2 Crystallographic data for compound **4** (CCDC 2344922).

Supplementary Data 3 Crystallographic data for compound **11** (CCDC 2344920).

Supplementary Data 4 Crystallographic data for compound **15** (CCDC 2344919).

Supplementary Data 5 Crystallographic data for compound **16** (CCDC 2344921).

Supplementary Data 6 Crystallographic data for compound **62** (CCDC 2344923).

## Data Availability

The authors declare that the data supporting the findings of this study are available within the paper and its Supplementary Information files. All other requests for materials and information should be addressed to the corresponding authors. Crystallographic data for the structures reported in this article have been deposited at the Cambridge Crystallographic Data Centre, under deposition numbers CCDC 2344919 (**15**), 2344920 (**11**), 2344921 (**16**), 2344922 (**4**) and 2344923 (**62**). Copies of the data can be obtained free of charge via https://www.ccdc.cam.ac.uk/structures/.
