# Supplementary material for: Difunctionalization of bicyclo[1.1.0]butanes enabled by merging C−C cleavage and ruthenium-catalysed remote C−H activation
Source: Nat Synth. 2025 Feb 17;4(5):655–63. doi: 10.1038/s44160-025-00745-3 (PMC12075002; doi:10.1038/s44160-025-00745-3)
Supplement: Supplementary file 1 — Supplementary Figs. 1–18, Tables 1–7 and experimental procedures, products characterization, late-stage derivatization, mechanism studies, computational details and NMR spectra. [file 44160_2025_745_MOESM1_ESM.pdf]

nature synthesis

Supplementary information https://doi.org/10.1038/s44160-025-00745-3

# Difunctionalization of bicyclo[1.1.0]butanes enabled by merging C–C cleavage and ruthenium-catalysed remote C–H activation

In the format provided by the authors and unedited

# Table of Contents

1. General Remark ........................................................................................S2

2. Synthesis of Starting Materials ..................................................................S3

3. Optimization of the Reaction Conditions ..................................................S8

4. General Procedure and Products Characterization ..................................S9

5. Gram-Scale Synthesis and Late-Stage Derivatization ...........................S86

6. X-Ray Crystallographic Analysis ...........................................................S97

7. Mechanism studies and Computational studies ...................................S106

8. References..............................................................................................S124

9. NMR Spectra .........................................................................................S126

## 1. General Remark

Catalytic reactions were performed under an $N_2$ atmosphere using pre-dried glassware. 1,4-Dioxane was dried over sodium and freshly distilled under $N_2$. Ethyl acetate and *n*-hexane were distilled prior to use. The reaction temperature was measured by digital thermometer PCE-T 390, which was in the range of 65 to 70 °C. The substrates were either purchased directly from commercial suppliers or prepared according to previously reported procedures. All other reagents and solvents used in this study were purchased from commercial sources and used as received. Chromatography was carried out on Merck silica gel 60 (40–63 μm). NMR spectra were recorded on an AVANCE NEO 300 MHz or Bruker Avance III 400/500/600 in the solvent indicated; chemical shifts (δ) are given in ppm relative to the residual solvent peak. All IR spectra were recorded on a Bruker FT-IR Alpha-P device. EI-MS spectra were recorded on Jeol AccuTOF at 70eV, ESI-MS spectra on Bruker MicrOTOF and maXis.

## 2. Synthesis of Starting Materials

### General Procedure for Synthesis of BCB esters 1[1]

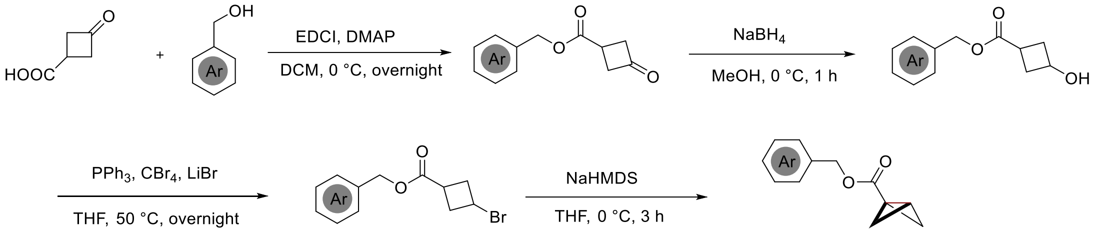

1a-1i

**Step-1**: To a stirred solution of 3-oxocyclobutane-1-carboxylic acid (1.1 equiv.) and EDCI (1.2 equiv.) in DCM (0.4 M) was added substituted benzyl alcohol (1 equiv.) and DMAP (20 mol%). The resulting mixture was stirred overnight at room temperature then diluted with DCM and saturated $NH_4Cl$ solution. The separated aqueous layer was extracted with DCM. The combined organic layers were washed with brine and dried over $MgSO_4$ and concentrated in vacuo. The residue as a colourless oil was used for the next step without further purification.

**Step-2**: To a stirred solution of the above ester in MeOH (3 M) was added $NaBH_4$ (0.5 equiv.) portionwise at 0 °C. The resulting solution was stirred for 30 min at 0 °C then diluted with saturated $NH_4Cl$ solution. The aqueous layer was extracted three times with DCM, and the combined organic layers were washed with brine, dried over $MgSO_4$ and concentrated in vacuo. The residue was purified by column chromatography on silica gel (*n*-hexane/EtOAc = 5/1→2.5/1) to give the substituted benzyl 3-hydroxycyclobutane-1-carboxylate as a colourless liquid.

**Step-3**: To a stirred solution of the above hydroxy-ester and $PPh_3$ (1.8 equiv.) in dry THF (0.3 M) was added $CBr_4$ (1.8 equiv.) and LiBr (1.9 equiv.) and the resulting mixture was stirred overnight at 50 °C. The reaction mixture was then diluted with $Et_2O$ and solid materials were removed by filtration through a pad of celite. The filtrate was washed with water and brine, dried over $MgSO_4$ and concentrated in vacuo. The residue was purified by column chromatography on silica gel (*n*-hexane/EtOAc = 100/0→20/1) to give the desired product, substituted benzyl 3-bromocyclobutane-1-carboxylate as a colourless liquid.

**Step-4:** To a stirred solution of the above bromo-ester in dry THF (0.2 M) was added

NaHMDS (2 M in THF, 1.03 equiv.) dropwise at 0 °C and the resulting mixture was stirred for 3 h at 0 °C. The reaction was then quenched with saturated $NH_4Cl$ solution and the aqueous phase was extracted with $Et_2O$. The combined organic layers were washed with brine, dried over $MgSO_4$ and concentrated in vacuo (at room temperature). The residue was purified by column chromatography on silica gel (*n*-hexane/EtOAc = 100/0→25/1) to give the desired product. Note: The data for **1a** and **1b** are consistent with those previously reported in the literature[1].

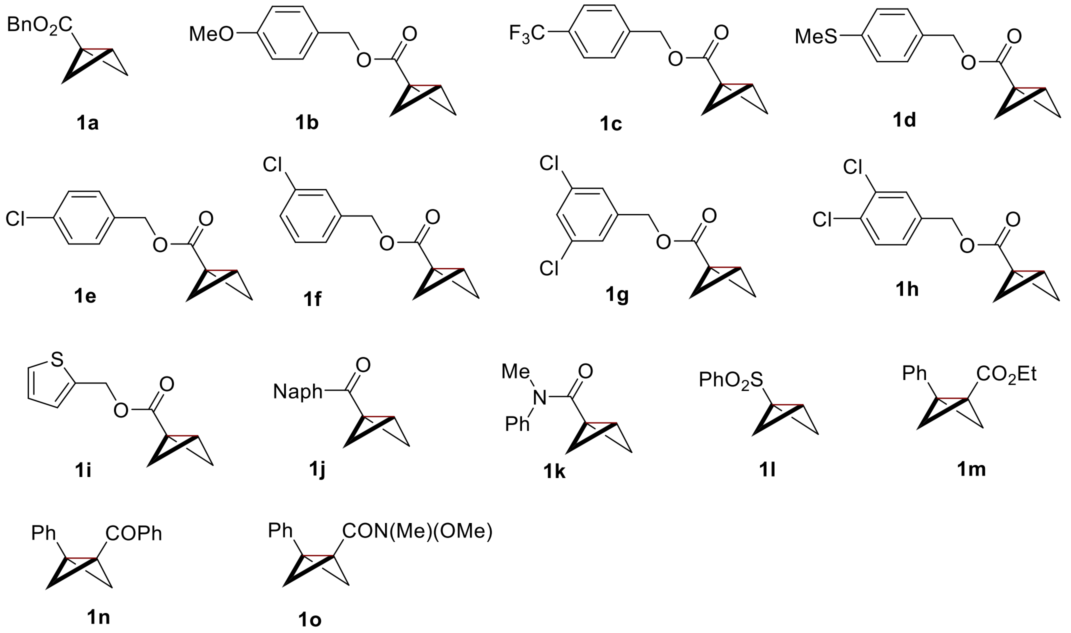


BCBs **1j-1o** were prepared according to previous literature[1-4].

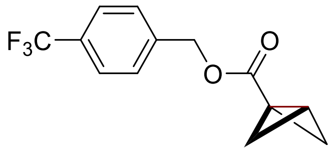


**4-(Trifluoromethyl)benzyl bicyclo[1.1.0]butane-1-carboxylate (1c).** The product **1c** (1.18 g. 31%) as a colorless oil was obtained following the general procedure using (4-(trifluoromethyl)phenyl)methanol (2.64 g, 15 mmol). **R**$_f$ = 0.48 (TLC: *n*-hexane/EtOAc = 10/1). **$^1$H NMR** (400 MHz, $CDCl_3$) δ = 7.61 (d, *J* = 8.0 Hz, 2H), 7.45 (d, *J* = 8.0 Hz, 2H), 5.20 (s, 2H), 2.44 – 2.37 (m, 2H), 2.14 (p, *J* = 3.1 Hz, 1H), 1.23 – 1.17 (m, 2H). **$^{13}$C NMR** (101 MHz, $CDCl_3$) δ = 172.9 ($C_q$), 140.4 (q, *J* = 1.5 Hz, $C_q$), 130.4 (q, *J* = 32.4 Hz, $C_q$), 128.1 (CH), 125.6 (q, *J* = 3.8 Hz, CH), 124.0 (q, *J* = 272.7 Hz, $C_q$), 65.5 ($CH_2$), 35.9 ($CH_2$), 17.3 (CH), 9.2 ($C_q$). **$^{19}$F NMR** (377 MHz,

$CDCl_3$) δ = -62.62. **IR** (ATR): 2949, 1707, 1405, 1322, 1123, 1063, 1017, 818, 745 $cm^{-1}$. **MS** (ESI) m/z (relative intensity): 257 $[M+H]^+$ (50), 279 $[M+Na]^+$ (100). **HR-MS** (ESI) m/z calcd for $C_{13}H_{11}O_2F_3$ $[M+Na]^+$ : 279.0603, found: 279.0610.

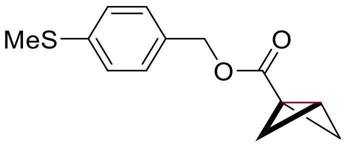


**4-(Methylthio)benzyl bicyclo[1.1.0]butane-1-carboxylate (1d).** The product **1d** (1.80 g, 51%) as a colorless oil was obtained following the general procedure using (4-(methylthio)phenyl)methanol (2.31 g, 15 mmol). **R**$_f$= 0.43 (TLC: *n*-hexane/EtOAc = 10/1). **$^1$H NMR** (400 MHz, $CDCl_3$) δ = 7.30 – 7.23 (m, 4H), 5.12 (s, 2H), 2.49 (s, 3H), 2.39 (dt, *J* = 3.5, 1.1 Hz, 2H), 2.11 (p, *J* = 3.2 Hz, 1H), 1.18 (dt, *J* = 2.8, 1.0 Hz, 2H). **$^{13}$C NMR** (101 MHz, $CDCl_3$) δ = 173.0 ($C_q$), 138.7 ($C_q$), 133.1 ($C_q$), 128.8 (CH), 126.6 (CH), 66.1 ($CH_2$), 35.8 ($CH_2$), 16.9 (CH), 15.8 ($CH_3$), 9.2 ($C_q$). **IR** (ATR): 2946, 1702, 1411, 1357, 1191, 1146, 1017, 800, 751 $cm^{-1}$. **MS** (ESI) m/z (relative intensity): 257 $[M+Na]^+$ (100), 289 $[M+CH_3OH]^+$ (20). **HR-MS** (ESI) m/z calcd for $C_{13}H_{14}O_2S$ $[M+Na]^+$ : 257.0607, found: 257.0609.

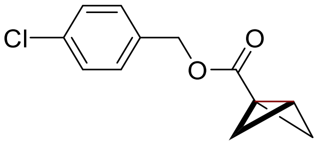


**4-Chlorobenzyl bicyclo[1.1.0]butane-1-carboxylate** (**1e**). The product **1e** (1.10 g, 33%) as a colorless oil was obtained following the general procedure using (4-chlorophenyl)methanol (2.14 g, 15 mmol). **R**$_f$ = 0.46 (TLC: *n*-hexane/EtOAc = 10/1). **$^1$H NMR** (400 MHz, $CDCl_3$) δ = 7.33 – 7.23 (m, 4H), 5.08 (s, 2H), 2.39 – 2.33 (m, 2H), 2.09 (p, *J* = 3.3 Hz, 1H), 1.18 – 1.14 (m, 2H). **$^{13}$C NMR** (101 MHz, $CDCl_3$) δ = 172.9 ($C_q$), 134.8 ($C_q$), 134.1 ($C_q$), 129.5 (CH), 128.8 (CH), 65.6 ($CH_2$), 35.8 ($CH_2$), 17.1 (CH), 9.2 ($C_q$). **IR** (ATR): 2948, 1786, 1493, 1414, 1192, 1095, 1016, 836, 752 $cm^{-1}$. **MS** (ESI) m/z (relative intensity): 245 $[M+Na]^+$ (100) ($^{35}$Cl), 247 $[M+Na]^+$ (50) ($^{37}$Cl). **HR-MS** (ESI) m/z calcd for $C_{12}H_{11}O_2Cl$ $[M+Na]^+$ : 245.0340, found:

245.0334.

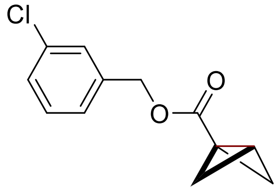

**3-Chlorobenzyl bicyclo[1.1.0]butane-1-carboxylate** (**1f**). The product **1f** (983 mg, 30%) as a colorless oil was obtained following the general procedure using (3-chlorophenyl)methanol (2.14 g, 15 mmol). $\mathbf{R_f}$ = 0.47 (TLC: *n*-hexane/EtOAc = 10/1). **$^1$H NMR** (400 MHz, $CDCl_3$) δ = 7.32 – 7.30 (m, 1H), 7.29 – 7.24 (m, 2H), 7.20 (ddd, *J* = 5.4, 4.1, 1.6 Hz, 1H), 5.10 (s, 2H), 2.38 (dt, *J* = 3.5, 1.1 Hz, 2H), 2.12 (p, *J* = 3.2 Hz, 1H), 1.17 (dt, *J* = 2.8, 1.0 Hz, 2H). **$^{13}$C NMR** (101 MHz, $CDCl_3$) δ = 172.8 ($C_q$), 138.3 ($C_q$), 134.4 ($C_q$), 129.9 (CH), 128.3 (CH), 128.0 (CH), 126.0 (CH), 65.4 ($CH_2$), 35.8 ($CH_2$), 17.1 (CH), 9.1 ($C_q$). **IR** (ATR): 2944, 1704, 1405, 1355, 1190, 1145, 1077, 868, 750 $cm^{-1}$. **MS** (ESI) m/z (relative intensity): 245 $[M+Na]^+$ (100) ($^{35}$Cl), 247 $[M+Na]^+$ (30) ($^{37}$Cl). **HR-MS** (ESI) m/z calcd for $C_{12}H_{11}O_2Cl$ $[M+Na]^+$ : 245.0340, found: 245.0340.

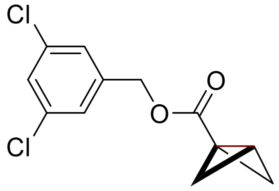

**3,5-Dichlorobenzyl bicyclo[1.1.0]butane-1-carboxylate** (**1g**). The product **1g** (760 mg, 20%) as a colorless oil was obtained following the general procedure using (3,5-dichlorophenyl)methanol (2.66 g, 15 mmol). $\mathbf{R_f}$= 0.49 (TLC: *n*-hexane/EtOAc = 10/1). **$^1$H NMR** (400 MHz, $CDCl_3$) δ = 7.34 (t, *J* = 1.9 Hz, 1H), 7.26 (d, *J* = 2.0 Hz, 2H), 5.12 (s, 2H), 2.45 (dt, 2H), 2.21 (p, *J* = 3.2 Hz, 1H), 1.26 (dt, 2H). **$^{13}$C NMR** (101 MHz, $CDCl_3$) δ = 172.7 ($C_q$), 139.7 ($C_q$), 135.2 ($C_q$), 128.3 (CH), 126.2 (CH), 64.7 ($CH_2$), 35.9 ($CH_2$), 17.5 (CH), 9.1 ($C_q$). **IR** (ATR): 3077, 2969, 1706, 1570, 1352, 1190, 1145, 1130, 841, 752 $cm^{-1}$. **MS** (ESI) m/z (relative intensity): 279 $[M+Na]^+$ (100) ($^{35}$Cl), 281 $[M+Na]^+$ (70) ($^{37}$Cl). **HR-MS** (ESI) m/z calcd for $C_{12}H_{11}O_2Cl_2$

$[M+Na]^+$: 278.9950, found: 278.9955.

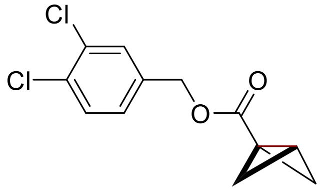

**3,4-Dichlorobenzyl bicyclo[1.1.0]butane-1-carboxylate** (**1h**). The product **1h** (1.25 g, 33%) as a colorless oil was obtained following the general procedure using (3,4-dichloro)phenyl)methanol (2.66 g, 15 mmol). $\mathbf{R_f}$ = 0.43 (TLC: *n*-hexane/EtOAc = 10/1). **$^1$H NMR** (400 MHz, $CDCl_3$) δ = 7.43 – 7.39 (m, 2H), 7.16 (dd, *J* = 8.2, 2.1 Hz, 1H), 5.07 (s, 2H), 2.38 (dt, *J* = 3.5, 1.1 Hz, 2H), 2.13 (p, *J* = 3.2 Hz, 1H), 1.18 (dt, *J* = 2.9, 1.1 Hz, 2H). **$^{13}$C NMR** (101 MHz, $CDCl_3$) δ = 172.8 ($C_q$), 136.6 ($C_q$), 132.7 ($C_q$), 132.28 ($C_q$), 130.6 (CH), 130.0 (CH), 127.3 (CH), 64.8 ($CH_2$), 35.9 ($CH_2$), 17.4 (CH), 9.1 ($C_q$). **IR** (ATR): 2946, 1708, 1390, 1191, 1148, 1104, 1032, 877, 754 $cm^{-1}$. **MS** (ESI) m/z (relative intensity): 279 $[M+Na]^+$ (70), 311 $[M+Na+CH_3OH]^+$ (100). **HR-MS** (ESI) m/z calcd for $C_{12}H_{11}O_2Cl_2$ $[M+Na]^+$: 278.9950, found: 278.9943.

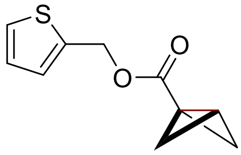

**Thiophen-2-ylmethyl bicyclo[1.1.0]butane-1-carboxylate** (**1i**). The product **1i** (900 mg, 32%) as a colorless oil was obtained following the general procedure using thiophen-2-ylmethanol (1.71 g, 15 mmol). $\mathbf{R_f}$ = 0.46 (TLC: *n*-hexane/EtOAc = 10/1). **$^1$H NMR** (400 MHz, $CDCl_3$) δ = 7.31 (dd, *J* = 5.1, 1.3 Hz, 1H), 7.07 (ddt, *J* = 3.5, 1.4, 0.7 Hz, 1H), 6.98 (dd, *J* = 5.1, 3.5 Hz, 1H), 5.30 (d, *J* = 0.6 Hz, 2H), 2.38 (dd, *J* = 3.5, 1.1 Hz, 2H), 2.11 – 2.08 (m, 1H), 1.17 (dt, *J* = 2.9, 1.1 Hz, 2H). **$^{13}$C NMR** (101 MHz, $CDCl_3$) δ = 173.0 ($C_q$), 138.5 ($C_q$), 128.0 (CH), 126.9 (CH), 126.8 (CH), 60.9 ($CH_2$), 35.9 ($CH_2$), 17.2 (CH), 9.2 ($C_q$). **IR** (ATR): 2984, 1728, 1682, 1479, 1399, 1225, 1127, 824, 735, $cm^{-1}$. **MS** (ESI) m/z (relative intensity): 217 $[M+Na]^+$ (100), 249 $[M+Na+CH_3OH]^+$ (30). **HR-MS** (ESI) m/z calcd for $C_{10}H_{10}O_2S$ $[M+Na]^+$: 217.0294, found: 217.0299.

# 3. Optimization of the Reaction Conditions[a]

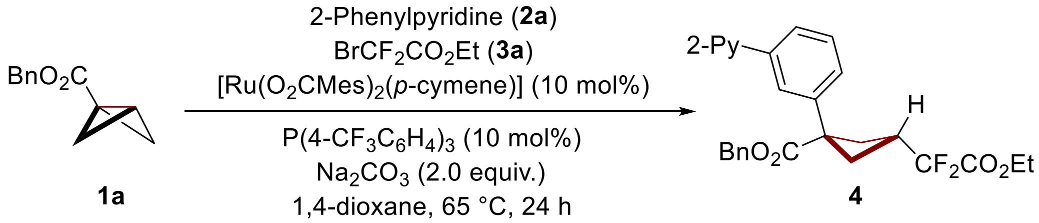


| Entry | Variation from standard conditions | Yield (%)[b] |
|---|---|---|
| 1 | None | 75 |
| 2 | $PPh_3$ | 54 |
| 3 | $P[3,5\text{-}(CF_3)_2C_6H_3]_3$ | 34 |
| 4 | $P(4\text{-}OMeC_6H_4)_3$ | 43 |
| 5 | bpy / NHC | 0 / trace |
| 6 | 5 mol% [Ru] and 5 mol% $P(4\text{-}CF_3C_6H_4)_3$ | 58[c] |
| 7 | $[Ru(OAc)_2(p\text{-cymene})]$ | 52 |
| 8 | $[RuCl_2(p\text{-cymene})]_2$ | 5 |
| 9 | $[Ru(^tBuCN)_5(H_2O)](BF_4)_2$ | 21[d] |
| 10 | $[Ru(^tBuCN)_5(H_2O)](BF_4)_2$ | 30 |
| 11 | 3.0 equiv $Na_2CO_3$ | 74 |
| 12 | $K_2CO_3$ | 45 |
| 13 | No $P(4\text{-}CF_3C_6H_4)_3$ | 0 |
| 14 | No Ru catalyst | 0 |

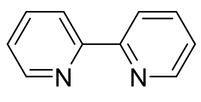


bpy ligand

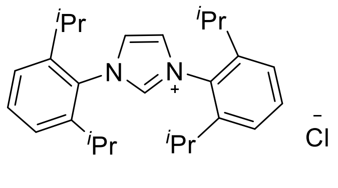


NHC ligand

**Table S1. Optimization of the reaction parameters: a**, Reaction conditions: **1a** (0.3 mmol), **2a** (3.0 equiv.), **3a** (3.0 equiv.), [Ru] (10 mol%), ligand (10 mol%), $Na_2CO_3$ (2.0 equiv.), 1,4-dioxane (2.0 mL), T = 65 °C, t = 24 h. **b**, Yield of isolated products. **c**, Using [Ru] (5 mol%), ligand (5 mol%), T = 75 °C and t = 24 h. **d**, T = 50 °C.

## 4. General Procedure and Products Characterization

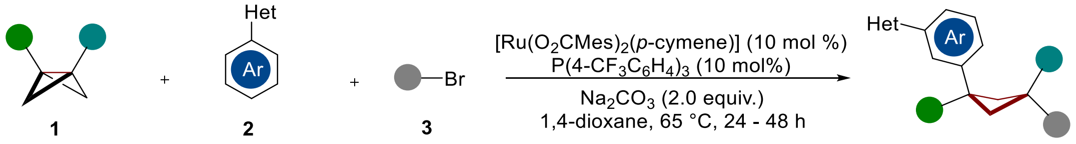


In an $N_2$ filled glovebox, $[Ru(O_2CMes)_2(p\text{-cymene})]$ (16.8 mg, 10.0 mol%), $P(4\text{-}CF_3C_6H_4)_3$ (14.0 mg, 10.0 mol%), $Na_2CO_3$ (64 mg, 0.6 mmol, 2.0 equiv.), 1,4-dioxane (2.0 mL), **1** (0.3 mmol, 1.0 equiv.), **2** (0.9 mmol, 3.0 equiv.) and **3** (0.9 mmol, 3.0 equiv.) were added into an oven-dried 20 mL pressure tube. The reaction mixture was stirred at 65 °C for 24 - 48 h. After cooling to ambient temperature, the mixture was purified by column chromatography on silica gel to afford the corresponding polysubstituted cyclobutanes **4-57** with two diastereomers and the ratio was determined by isolated yield, **$^1$H-NMR** or **$^{19}$F-NMR**. Note: In the preparation of 1,1,3-substituted cyclobutanes, the *cis*-structure is major isomer, while for the synthesis of 1,1,3,3-substituted cyclobutanes, the (*1r,3r*)-structure is major isomer, which is confirmed by X-ray crystallographic analysis (see X-ray structure of compound **62** in part 6), NMR spectra and is consistent with DFT studies.

**Characterization Data of Products**

Compound **4** was obtained following the general procedure with **1a** (56 mg, 0.3 mmol), 2-phenylpyridine (140 mg, 0.9 mmol), and ethyl 2-bromo-2,2-difluoroacetate (183 mg, 0.9 mmol) for 24 h. Purification by column chromatography on silica gel (*n*-hexane/EtOAc = 10/1 → 5/1) yielded **4** (105 mg, 75%) with two separable diastereomers (*cis*: *trans* = 3.1:1). **R**$_f$ = 0.40 for *cis*-**4** and 0.47 for *trans*-**4** (TLC: *n*-hexane/EtOAc = 3/1).

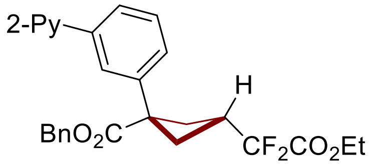


**Benzyl (1s,3s)-3-(2-ethoxy-1,1-difluoro-2-oxoethyl)-1-(3-(pyridin-2-yl)phenyl)cyclobutane-1-carboxylate** (*cis*-**4**, 79 mg, 57%) was obtained as a white solid. Recrystallization on DCM/*n*-hexanes. **M.p.**: 99 – 101 °C. $^{1}$**H NMR** (500 MHz, $CDCl_3$) δ = 8.70 (ddd, *J* = 4.8, 1.8, 1.0 Hz, 1H), 8.06 – 8.04 (m, 1H), 7.93 – 7.89 (m, 1H), 7.77 – 7.72 (m, 1H), 7.69 – 7.66 (m, 1H), 7.49 – 7.46 (m, 2H), 7.27 – 7.23 (m, 4H), 7.20 – 7.16 (m, 2H), 5.08 (s, 2H), 4.29 (q, *J* = 7.1 Hz, 2H), 3.13 – 3.07 (m, 2H), 2.95 – 2.76 (m, 3H), 1.33 (t, *J* = 7.1 Hz, 3H). $^{13}$**C NMR** (126 MHz, $CDCl_3$) δ = 174.3 ($C_q$), 163.8 (t, *J* = 33.4 Hz, $C_q$), 157.2 ($C_q$), 149.9 (CH), 141.6 ($C_q$), 140.0 ($C_q$), 136.9 (CH), 135.9 ($C_q$), 129.2 (CH), 128.6 (CH), 128.2 (CH), 127.8 (CH), 127.3 (CH), 126.1 (CH), 125.4 (CH), 122.5 (CH), 120.9 (CH), 115.5 (t, *J* = 250.2 Hz, $C_q$), 67.0 ($CH_2$), 63.0 ($CH_2$), 47.9 ($C_q$), 32.1 (t, *J* = 25.8 Hz, CH) , 31.2 (t, *J* = 4.9 Hz, $CH_2$), 14.1 ($CH_3$). $^{19}$**F NMR** (471 MHz, $CDCl_3$) δ = -114.27 (d, *J* = 15.2 Hz). **IR** (ATR): 2965, 1766, 1731, 1462, 1308, 1224, 1042, 773 $cm^{-1}$. **MS** (ESI) *m*/*z* (relative intensity): 466 $[M+H]^+$ (30), 488 $[M+Na]^+$ (100). **HR-MS** (ESI) *m*/*z* calcd for $C_{27}H_{25}F_2NO_4$ $[M+H]^+$: 466.1824, found: 466.1825.

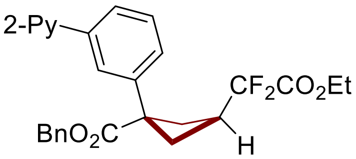


**Benzyl (1r,3r)-3-(2-ethoxy-1,1-difluoro-2-oxoethyl)-1-(3-(pyridin-2-yl)phenyl)cyclobutane-1-carboxylate** (*trans*-**4**, 26 mg, 19%) was obtained as a white solid. **M.p.**: 88 – 90 °C. **$^1$H NMR** (600 MHz, $CDCl_3$) δ = 8.71 (ddd, *J* = 4.8, 1.9, 0.9 Hz, 1H), 7.92 – 7.85 (m, 2H), 7.75 (td, *J* = 7.7, 1.9 Hz, 1H), 7.68 (dt, *J* = 8.0, 1.1 Hz, 1H), 7.45 (t, *J* = 7.7 Hz, 1H), 7.33 – 7.28 (m, 1H), 7.29 – 7.22 (m, 4H), 7.20 – 7.15 (m, 2H), 5.13 (s, 2H), 4.29 (q, *J* = 7.1 Hz, 2H), 3.20 – 3.01 (m, 3H), 2.80 – 2.73 (m, 2H), 1.32 (t, *J* = 7.2 Hz, 3H). **$^{13}$C NMR** (151 MHz, $CDCl_3$) δ = 174.7 ($C_q$), 163.8 (t, *J* = 33.3 Hz, $C_q$), 157.2 ($C_q$), 149.8 (CH), 143.3 ($C_q$), 139.8 ($C_q$), 136.8 (CH), 135.9 ($C_q$), 129.0 (CH), 128.6 (CH), 128.2 (CH), 127.7 (CH), 126.7 (CH), 125.8 (CH), 124.7 (CH), 122.4 (CH), 120.8 (CH), 114.9 (t, *J* = 250.0 Hz, $C_q$), 67.2 ($CH_2$), 63.0 ($CH_2$), 48.6 ($C_q$), 33.6 (t, *J* = 26.0 Hz, CH), 31.7 (t, *J* = 5.0 Hz, $CH_2$), 14.1 ($CH_3$). **$^{19}$F NMR** (471 MHz, $CDCl_3$) δ = -114.31 (d, *J* = 15.0 Hz). **IR** (ATR): 2959, 1762, 1727, 1464, 1308, 1268, 1122, 1052, 737 $cm^{-1}$. **MS** (ESI) *m*/*z* (relative intensity): 466 $[M+H]^+$ (30), 488 $[M+Na]^+$ (100). **HR-MS** (ESI) *m*/*z* calcd for $C_{27}H_{25}F_2NO_4$ $[M+H]^+$: 466.1824, found: 466.1828.

Compound **5** was obtained following the general procedure with **1a** (56 mg, 0.3 mmol), 1-(6-phenylpyridin-3-yl)ethan-1-one (177 mg, 0.9 mmol), and ethyl 2-bromo-2,2-difluoroacetate (183 mg, 0.9 mmol) for 24 h. Purification by column chromatography on silica gel (*n*-hexane/EtOAc/DCM = 8/1/1) yielded **5** (90 mg, 59%) as a colorless oil with two separable diastereomers (*cis*: *trans* = 2.8:1). **R**$_f$= 0.36 for *cis*-**5** and 0.40 for *trans*-**5** (TLC: *n*-hexane/EtOAc/DCM = 3/1/1).

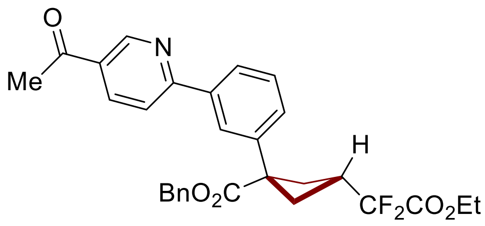


**Benzyl (1s,3s)-1-(3-(5-acetylpyridin-2-yl)phenyl)-3-(2-ethoxy-1,1-difluoro-2-oxoethyl)cyclobutane-1-carboxylate** (*cis*-**5**, 66 mg, 43%) was obtained as a colorless oil. **$^1$H NMR** (500 MHz, $CDCl_3$) δ = 9.24 (dd, *J* = 2.3, 0.9 Hz, 1H), 8.29 (dd, *J* = 8.3, 2.3 Hz, 1H), 8.13 (td, *J* = 1.8, 0.6 Hz, 1H), 7.99 (dt, *J* = 7.1, 1.7 Hz, 1H), 7.79 (dd, *J* = 8.3, 0.9 Hz, 1H), 7.57 – 7.47 (m, 2H), 7.29 – 7.25 (m, 3H), 7.21 – 7.18 (m, 2H), 5.10 (s, 2H), 4.31 (q, *J* = 7.1 Hz, 2H), 3.16 – 3.08 (m, 2H), 2.97 – 2.77 (m, 3H), 2.68 (s, 3H), 1.34 (t, *J* = 7.1 Hz, 3H). **$^{13}$C NMR** (126 MHz, $CDCl_3$) δ = 196.6 ($C_q$), 174.2 ($C_q$), 163.8 (t, *J* = 33.3 Hz, $C_q$), 160.6 ($C_q$), 150.3 (CH), 141.9 ($C_q$), 138.7 ($C_q$), 136.5 (CH), 135.8 ($C_q$), 130.9 ($C_q$), 129.3 (CH), 128.6 (CH), 128.4 (CH), 128.2 (CH), 127.9 (CH), 126.5 (CH), 125.9 (CH), 120.4 (CH), 115.4 (t, *J* = 250.3 Hz, $C_q$), 67.0 ($CH_2$), 63.0 ($CH_2$), 47.9 ($C_q$), 32.1 (t, *J* = 25.7 Hz, CH), 31.2 (t, *J* = 4.9 Hz, $CH_2$), 26.9 ($CH_3$), 14.1 ($CH_3$). **$^{19}$F NMR** (471 MHz, $CDCl_3$) δ = -114.26 (d, *J* = 15.3 Hz). **IR** (ATR): 2962, 1763, 1729, 1686, 1588, 1271, 1128, 957, 744, 697 $cm^{-1}$. **MS** (ESI) *m/z* (relative intensity): 508 $[M+H]^+$ (30), 530 $[M+Na]^+$ (100). **HR-MS** (ESI) *m/z* calcd for $C_{29}H_{27}F_2NO_5$ $[M+H]^+$: 530.1750, found: 530.1761.

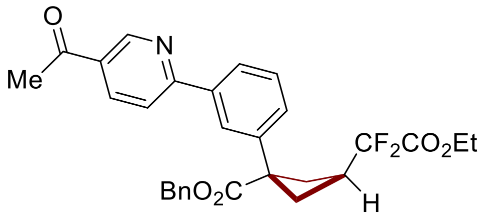


**Benzyl (1r,3r)-1-(3-(5-acetylpyridin-2-yl)phenyl)-3-(2-ethoxy-1,1-difluoro-2-oxoethyl)cyclobutane-1-carboxylate** (*trans*-**5**, 24 mg, 16%) was obtained as a colorless oil. **$^1$H NMR** (500 MHz, $CDCl_3$) δ = 9.23 (dd, *J* = 2.3, 0.9 Hz, 1H), 8.28 (dd, *J* = 8.3, 2.3 Hz, 1H), 7.96 – 7.91 (m, 2H), 7.78 (dd, *J* = 8.3, 0.9 Hz, 1H), 7.47 (t, *J* = 7.7 Hz, 1H), 7.36

– 7.33 (m, 1H), 7.28 – 7.24 (m, 3H), 7.20 – 7.14 (m, 2H), 5.12 (s, 2H), 4.29 (q, *J* = 7.2 Hz, 2H), 3.19 – 2.99 (m, 3H), 2.79 – 2.70 (m, 2H), 2.67 (s, 3H), 1.32 (t, *J* = 7.1 Hz, 3H). **$^{13}$C NMR** (126 MHz, $CDCl_3$) δ = 196.6 ($C_q$), 174.6 ($C_q$), 163.8 (t, *J* = 33.3 Hz, $C_q$), 160.7 ($C_q$), 150.6 (CH), 143.6 ($C_q$), 138.5 ($C_q$), 136.5 (CH), 135.8 ($C_q$) 130.8 ($C_q$), 129.2 (CH), 128.6 (CH), 128.3 (CH), 127.8 (CH), 127.7 (CH), 126.3 (CH), 125.2 (CH), 120.4 (CH), 114.9 (t, *J* = 249.9 Hz, $C_q$), 67.3 ($CH_2$), 63.0 ($CH_2$), 48.5 ($C_q$), 33.6 (t, *J* = 26.0 Hz, CH), 31.7 (t, *J* = 5.0 Hz, $CH_2$), 26.9 ($CH_3$), 14.1 ($CH_3$). **$^{19}$F NMR** (471 MHz, $CDCl_3$) δ = -114.40 (d, *J* = 14.8 Hz). **IR** (ATR): 2960, 1763, 1728, 1686, 1588, 1269, 1125, 957, 746, 694 $cm^{-1}$. **MS** (ESI) *m/z* (relative intensity): 508 $[M+H]^+$ (30), 530 $[M+Na]^+$ (100). **HR-MS** (ESI) *m/z* calcd for $C_{29}H_{27}F_2NO_5$ $[M+Na]^+$: 530.1750, found: 530.1759.

Compound **6** was obtained following the general procedure with **1a** (56 mg, 0.3 mmol), 4-methoxy-2-phenylpyridine (167 mg, 0.9 mmol), and ethyl 2-bromo-2,2-difluoroacetate (183 mg, 0.9 mmol) for 24 h. Purification by column chromatography on silica gel (*n*-hexane/EtOAc/DCM = 5/1/1) yielded **6** (109 mg, 73%) as a colorless oil with two separable diastereomers (*cis*: *trans* = 3:1). **R**$_f$= 0.35 for *cis*-**6** and 0.38 for *trans*-**6** (TLC: *n*-hexane/EtOAc/DCM = 3/1/1).

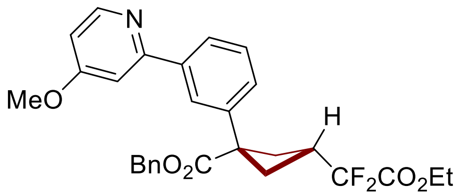


**Benzyl (1s,3s)-3-(2-ethoxy-1,1-difluoro-2-oxoethyl)-1-(3-(4-methoxypyridin-2-yl)phenyl)cyclobutane-1-carboxylate** (*cis*-**6**, 82 mg, 55%) was obtained as a colorless oil. **$^1$H NMR** (500 MHz, $CDCl_3$) δ = 8.53 (dd, *J* = 5.7, 0.5 Hz, 1H), 8.04 – 8.00 (m, 1H), 7.90 – 7.84 (m, 1H), 7.49 – 7.44 (m, 2H), 7.29 – 7.22 (m, 3H), 7.18 (dd, *J* = 6.5, 2.3 Hz, 3H), 6.79 (dd, *J* = 5.7, 2.4 Hz, 1H), 5.08 (s, 2H), 4.29 (q, *J* = 7.2 Hz, 2H), 3.90 (s, 3H), 3.09 (dd, *J* = 13.1, 8.1 Hz, 2H), 2.93 – 2.75 (m, 3H), 1.33 (t, *J* = 7.1 Hz, 3H). **$^{13}$C**

**NMR** (126 MHz, $CDCl_3$) δ = 174.3 ($C_q$), 166.5 ($C_q$), 163.8 (t, *J* = 33.3 Hz, $C_q$), 159.0 ($C_q$), 151.1 (CH), 141.5 ($C_q$), 140.1 ($C_q$), 135.9 ($C_q$), 129.1 (CH), 128.6 (CH), 128.2 (CH), 127.8 (CH), 127.4 (CH), 126.2 (CH), 125.5 (CH), 115.5 (t, *J* = 250.2 Hz, $C_q$), 108.3 (CH), 107.3 (CH), 67.0 ($CH_2$), 63.0 ($CH_2$), 55.4 ($CH_3$), 47.9 ($C_q$), 32.1 (t, *J* = 25.8 Hz, CH), 31.2 (t, *J* = 4.9 Hz, $CH_2$), 14.1 ($CH_3$). **$^{19}$F NMR** (471 MHz, $CDCl_3$) δ = -114.27 (d, *J* = 15.3 Hz). **IR** (ATR): 2962, 1763, 1729, 1591, 1372, 1207, 1036, 743, 697 $cm^{-1}$. **MS** (ESI) *m*/*z* (relative intensity): 496 $[M+H]^+$ (100), 518 $[M+Na]^+$ (100). **HR-MS** (ESI) *m*/*z* calcd for $C_{28}H_{27}F_2NO_5$ $[M+Na]^+$: 518.1750, found:518.1756.

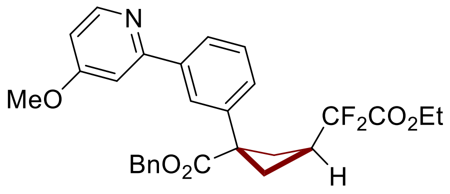


**Benzyl (1r,3r)-3-(2-ethoxy-1,1-difluoro-2-oxoethyl)-1-(3-(4-methoxypyridin-2-yl)phenyl)cyclobutane-1-carboxylate** (*trans*-**6**, 27 mg, 18%) was obtained as a colorless oil. **$^1$H NMR** (500 MHz, $CDCl_3$) δ = 8.53 (d, *J* = 5.7 Hz, 1H), 7.88 – 7.80 (m, 2H), 7.43 (t, *J* = 7.7 Hz, 1H), 7.30 – 7.25 (m, 4H), 7.21 – 7.14 (m, 3H), 6.79 (dd, *J* = 5.7, 2.4 Hz, 1H), 5.12 (s, 2H), 4.29 (q, *J* = 7.1 Hz, 2H), 3.91 (s, 3H), 3.19 – 2.98 (m, 3H), 2.79 – 2.71 (m, 2H), 1.32 (t, *J* = 7.1 Hz, 3H). **$^{13}$C NMR** (126 MHz, $CDCl_3$) δ = 174.7 ($C_q$), 166.5 ($C_q$), 163.8 (t, *J* = 33.4 Hz, $C_q$), 159.0 ($C_q$), 151.1 (CH), 143.3 ($C_q$), 139.8 ($C_q$), 135.9 ($C_q$), 128.9 (CH), 128.6 (CH), 128.2 (CH), 127.7 (CH), 126.8 (CH), 125.9 (CH), 124.8 (CH), 114.9 (t, *J* = 250.2 Hz, $C_q$), 108.3 (CH), 107.3 (CH), 67.2 ($CH_2$), 63.0 ($CH_2$), 55.4 ($CH_3$), 48.6 ($C_q$), 33.6 (t, *J* = 26.1 Hz, CH), 31.7 (t, *J* = 4.9 Hz, $CH_2$), 14.1 ($CH_3$). **$^{19}$F NMR** (471 MHz, $CDCl_3$) δ = -114.33 (d, *J* = 14.7 Hz). **IR** (ATR): 2959, 1763, 1728, 1591, 1373, 1206, 1036, 737, 702 $cm^{-1}$. **MS** (ESI) *m*/*z* (relative intensity): 496 $[M+H]^+$ (100), 518 $[M+Na]^+$ (75). **HR-MS** (ESI) *m*/*z* calcd for $C_{28}H_{27}F_2NO_5$ $[M+Na]^+$: 518.1750, found:518.1759.

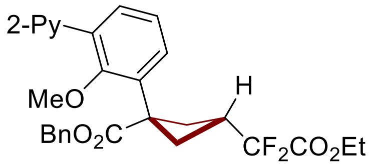


**Benzyl 3-(2-ethoxy-1,1-difluoro-2-oxoethyl)-1-(2-methoxy-3-(pyridin-2-yl)phenyl)cyclobutane-1-carboxylate** (**7**). The product was obtained following the general procedure with **1a** (56 mg, 0.3 mmol), 2-(2-methoxyphenyl)pyridine (167 mg, 0.9 mmol), and ethyl 2-bromo-2,2-difluoroacetate (183 mg, 0.9 mmol) for 24 h. Purification by column chromatography on silica gel (*n*-hexane/EtOAc = 10/1→3/1) yielded **7** (64 mg, 43%) as a colorless oil with two diastereomers (*cis*: *trans* = 14.3:1, determined by **$^{19}$F-NMR**). **R$_f$** = 0.38 (TLC: *n*-hexane/EtOAc = 3/1). **$^1$H NMR** (500 MHz, $CDCl_3$) (*cis*-isomer) δ = 8.71 (d, *J* = 5.0 Hz, 1H), 7.68 (td, *J* = 7.7, 1.8 Hz, 1H), 7.62 (dd, *J* = 7.7, 1.7 Hz, 1H), 7.58 (d, *J* = 7.9 Hz, 1H), 7.49 (dd, *J* = 7.8, 1.7 Hz, 1H), 7.29 – 7.20 (m, 7H), 5.13 (s, 2H), 4.30 (q, *J* = 7.2 Hz, 2H), 3.16 – 3.06 (m, 5H), 3.00 – 2.90 (m, 1H), 2.65 – 2.58 (m, 2H), 1.33 (t, *J* = 7.2 Hz, 3H). **$^{13}$C NMR** (126 MHz, $CDCl_3$) (*cis*-isomer) δ = 174.2 ($C_q$), 163.9 (t, *J* = 33.4 Hz, $C_q$), 156.7 ($C_q$), 156.6 ($C_q$), 149.9 (CH), 136.4 (CH), 136.1 ($C_q$), 134.9 ($C_q$), 133.4 ($C_q$), 131.3 (CH), 128.5 (CH), 128.1 (CH), 128.0 (CH), 127.0 (CH), 124.5 (CH), 123.8 (CH), 122.2 (CH), 115.4 (t, *J* = 250.2 Hz, $C_q$), 66.7 ($CH_2$), 62.9 ($CH_2$), 60.9 ($CH_3$), 45.6 ($C_q$), 32.6 (t, *J* = 25.8 Hz, CH), 30.5 (t, *J* = 4.9 Hz, $CH_2$), 14.1 ($CH_3$). **$^{19}$F NMR** (471 MHz, $CDCl_3$) δ = -113.82 (d, *J* = 15.2 Hz, *cis*), -114.18 (d, *J* = 15.6 Hz, *trans*). **IR** (ATR): 2986, 1729, 1455, 1231, 1127, 1041, 1002, 759 $cm^{-1}$. **MS** (ESI) *m*/*z* (relative intensity): 496 $[M+H]^+$ (10), 518 $[M+Na]^+$ (100). **HR-MS** (ESI) *m*/*z* calcd for $C_{28}H_{27}F_2NO_5$ $[M+H]^+$: 496.1935, found: 496.1930.

Compound **8** was obtained following the general procedure with **1a** (56 mg, 0.3 mmol), methyl 4-(pyridin-2-yl)benzoate (192 mg, 0.9 mmol), and ethyl 2-bromo-2,2-difluoroacetate (183 mg, 0.9 mmol) for 24 h. Purification by column chromatography on silica gel (*n*-hexane/EtOAc/DCM = 5/1/1) yielded **8** (79 mg, 51%)

as a colorless oil with two separable diastereomers (*cis*: *trans* = 3.3:1). **R**$_f$= 0.35 for *cis*-**8** and 0.38 for *trans*-**8** (TLC: *n*-hexane/EtOAc/DCM = 3/1/1).

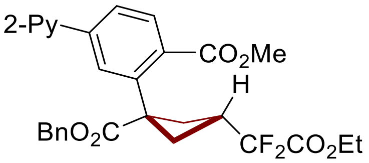


**Methyl 2-((1s,3s)-1-((benzyloxy)carbonyl)-3-(2-ethoxy-1,1-difluoro-2-oxoethyl)cyclobutyl)-4-(pyridin-2-yl)benzoate** (*cis*-**8**, 61 mg, 39%) was obtained as a colorless oil. **$^1$H NMR** (500 MHz, $CDCl_3$) δ = 8.74 (dt, *J* = 4.8, 1.4 Hz, 1H), 8.33 (d, *J* = 1.7 Hz, 1H), 8.11 (d, *J* = 8.1 Hz, 1H), 7.98 (dd, *J* = 8.1, 1.7 Hz, 1H), 7.83 – 7.78 (m, 2H), 7.34 – 7.20 (m, 6H), 5.11 (s, 2H), 4.28 (q, *J* = 7.2 Hz, 2H), 3.67 (s, 3H), 3.21 (dd, *J* = 13.1, 10.1 Hz, 2H), 2.92 – 2.80 (m, 1H), 2.65 – 2.56 (m, 2H), 1.31 (t, *J* = 7.1 Hz, 3H). **$^{13}$C NMR** (126 MHz, $CDCl_3$) δ = 173.9 ($C_q$), 167.5 ($C_q$), 163.9 (t, *J* = 33.2 Hz, $C_q$), 156.1 ($C_q$), 150.1 (CH), 142.8 ($C_q$), 142.3 ($C_q$), 137.1 (CH), 136.2 ($C_q$), 132.5 (CH), 129.7 ($C_q$), 128.5 (CH), 128.0 (CH), 128.0 (CH), 126.4 (CH), 125.7 (CH), 123.1 (CH), 121.2 (CH), 115.2 (t, *J* = 250.4 Hz, $C_q$), 66.8 ($CH_2$), 63.0 ($CH_2$), 52.2 ($CH_3$), 48.4 ($C_q$), 32.7 (t, *J* = 25.9 Hz, CH), 31.7 (t, *J* = 5.0 Hz, $CH_2$), 14.1 ($CH_3$). **$^{19}$F NMR** (471 MHz, $CDCl_3$) δ = -113.64 (d, *J* = 14.7 Hz). **IR** (ATR): 2955, 1763, 1716, 1583, 1434, 1216, 1117, 1043, 730, 699 cm$^{-1}$. **MS** (ESI) *m*/*z* (relative intensity): 524 $[M+H]^+$ (15), 546 $[M+Na]^+$ (100). **HR-MS** (ESI) *m*/*z* calcd for $C_{29}H_{27}F_2NO_6$ $[M+H]^+$: 524.1879, found: 524.1881.

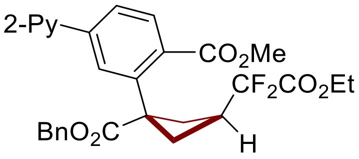


**Methyl 2-((1r,3r)-1-((benzyloxy)carbonyl)-3-(2-ethoxy-1,1-difluoro-2-oxoethyl)cyclobutyl)-4-(pyridin-2-yl)benzoate** (*trans*-**8**, 18 mg, 12%) was obtained as a colorless oil. **$^1$H NMR** (500 MHz, $CDCl_3$) δ = 8.73 (dt, *J* = 4.8, 1.5 Hz, 1H), 8.02 (d, *J* = 1.6 Hz, 1H),

8.00 – 7.93 (m, 2H), 7.80 – 7.78 (m, 2H), 7.31 – 7.28 (m, 1H), 7.27 – 7.26 (m, 1H), 7.26 – 7.24 (m, 2H), 7.20 – 7.16 (m, 2H), 5.15 (s, 2H), 4.27 (q, *J* = 7.1 Hz, 2H), 3.71 (s, 3H), 3.46 – 3.35 (m, 1H), 2.95 – 2.89 (m, 2H), 2.67 (t, *J* = 10.9 Hz, 2H), 1.30 (t, *J* = 7.1 Hz, 3H). **$^{13}$C NMR** (126 MHz, $CDCl_3$) δ = 174.9 ($C_q$), 167.5 ($C_q$), 163.9 (t, *J* = 33.3 Hz, $C_q$), 156.1 ($C_q$), 150.0 (CH), 144.6 ($C_q$), 142.7 ($C_q$), 137.0 (CH), 136.2 ($C_q$), 131.5 (CH), 128.6 ($C_q$), 128.5 (CH), 128.0 (CH), 127.8 (CH), 127.2 (CH), 125.4 (CH), 123.1 (CH), 121.2 (CH), 115.1 (t, *J* = 249.7 Hz, $C_q$), 66.9 ($CH_2$), 63.0 ($CH_2$), 52.2 ($CH_3$), 48.5 ($C_q$), 33.2 (t, *J* = 25.5 Hz, CH), 31.9 (t, *J* = 5.2 Hz, $CH_2$), 14.1 ($CH_3$). **$^{19}$F NMR** (471 MHz, $CDCl_3$) δ = -114.57 (d, *J* = 15.5 Hz). **IR** (ATR): 2959, 1762, 1727, 1581, 1461, 1315, 1130, 1040, 734, 702 $cm^{-1}$. **MS** (ESI) *m*/*z* (relative intensity): 524 $[M+H]^+$ (10), 546 $[M+Na]^+$ (100). **HR-MS** (ESI) *m*/*z* calcd for $C_{29}H_{27}F_2NO_6$ $[M+H]^+$: 524.1879, found: 524.1877.

Compound **9** was obtained following the general procedure with **1a** (56 mg, 0.3 mmol), 2-(4-(methylthio)phenyl)pyridine (181 mg, 0.9 mmol), and ethyl 2-bromo-2,2-difluoroacetate (183 mg, 0.9 mmol) for 24 h. Purification by column chromatography on silica gel (*n*-hexane/EtOAc/DCM = 8/1/1→5/1/1) yielded **9** (83 mg, 54%) with two separable diastereomers (*cis*: *trans* = 4.2:1). **R**$_f$ = 0.21 for *cis*-**9** and 0.26 for *trans*-**9** (TLC: *n*-hexane/EtOAc/DCM = 5/1/1).

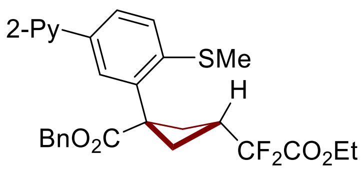


**Benzyl (1s,3s)-3-(2-ethoxy-1,1-difluoro-2-oxoethyl)-1-(2-(methylthio)-5-(pyridin-2-yl)phenyl)cyclobutane-1-carboxylate** (*cis*-**9**, 67 mg, 44%) was obtained as a colorless oil. **$^1$H NMR** (500 MHz, $CDCl_3$) δ = 8.70 (ddd, *J* = 4.8, 1.8, 1.1 Hz, 1H), 8.16 (d, *J* = 1.9 Hz, 1H), 7.90 (dd, *J* = 8.2, 1.9 Hz, 1H), 7.82 – 7.71 (m, 3H), 7.38 (d, *J* = 8.2 Hz, 1H), 7.29 – 7.26 (m, 1H), 7.26 – 7.20 (m, 4H), 5.13 (s, 2H), 4.29 (q, *J* = 7.1 Hz, 2H), 3.18

– 3.09 (m, 2H), 3.01 – 2.84 (m, 1H), 2.80 – 2.71 (m, 2H), 2.33 (s, 3H), 1.33 (t, *J* = 7.1 Hz, 3H). **$^{13}$C NMR** (126 MHz, $CDCl_3$) δ = 173.9 ($C_q$), 163.9 (t, *J* = 33.2 Hz, $C_q$), 156.9 ($C_q$), 149.9 (CH), 139.8 ($C_q$), 139.1 ($C_q$), 136.9 (CH), 136.5 ($C_q$), 136.1 ($C_q$), 128.4 (CH), 128.2 (CH), 128.0 (CH), 128.0 (CH), 126.4 (CH), 124.9 (CH), 122.3 (CH), 120.5 (CH), 115.3 (t, *J* = 250.4 Hz, $C_q$), 66.9 ($CH_2$), 63.0 ($CH_2$), 48.1 ($C_q$), 32.8 (t, *J* = 25.9 Hz, CH), 30.3 (t, *J* = 4.9 Hz, $CH_2$), 16.8 ($CH_3$), 14.1 ($CH_3$). **$^{19}$F NMR** (471 MHz, $CDCl_3$) δ = -113.69 (d, *J* = 15.1 Hz). **IR** (ATR): 2953, 1764, 1725, 1434, 1283, 1126, 1056, 745 cm$^{-1}$. **MS** (ESI) *m*/*z* (relative intensity): 512 $[M+H]^+$ (80), 534 $[M+Na]^+$ (100). **HR-MS** (ESI) *m*/*z* calcd for $C_{28}H_{27}F_2NO_4S$ $[M+H]^+$: 512.1702, found: 512.1710.

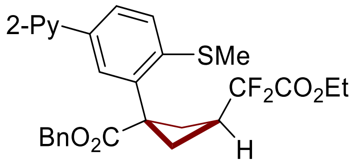


**Benzyl (1r,3r)-3-(2-ethoxy-1,1-difluoro-2-oxoethyl)-1-(2-(methylthio)-5-(pyridin-2-yl)phenyl)cyclobutane-1-carboxylate** (*trans*-**9**, 16 mg, 10%) was obtained as a colorless oil. **$^{1}$H NMR** (500 MHz, $CDCl_3$) δ = 8.69 (ddd, *J* = 4.8, 1.8, 1.0 Hz, 1H), 7.92 – 7.85 (m, 2H), 7.79 – 7.69 (m, 2H), 7.31 (d, *J* = 8.0 Hz, 1H), 7.28 – 7.21 (m, 4H), 7.18 – 7.13 (m, 2H), 5.14 (s, 2H), 4.29 (q, *J* = 7.1 Hz, 2H), 3.41 – 3.25 (m, 1H), 3.04 – 2.95 (m, 2H), 2.82 – 2.73 (m, 2H), 2.36 (s, 3H), 1.32 (t, *J* = 7.2 Hz, 3H). **$^{13}$C NMR** (126 MHz, $CDCl_3$) δ = 174.8 ($C_q$), 163.9 (t, *J* = 33.4 Hz, $C_q$), 156.8 ($C_q$), 149.8 (CH), 140.9 ($C_q$), 138.7 ($C_q$), 136.9 (CH), 136.3 ($C_q$), 136.1 ($C_q$), 128.5 (CH), 128.1 (CH), 127.8 (CH), 127.1 (CH), 126.2 (CH), 125.7 (CH), 122.2 (CH), 120.5 (CH), 115.0 (t, *J* = 249.8 Hz, $C_q$), 66.9 ($CH_2$), 63.0 ($CH_2$), 48.9 ($C_q$), 33.5 (t, *J* = 25.7 Hz, CH), 31.6 (t, *J* = 5.1 Hz, $CH_2$), 16.5 ($CH_3$), 14.1 ($CH_3$). **$^{19}$F NMR** (471 MHz, $CDCl_3$) δ = -114.44 (d, *J* = 15.2 Hz). **IR** (ATR): 2957, 1762, 1725, 1431, 1310, 1121, 1053, 738 cm$^{-1}$. **MS** (ESI) *m*/*z* (relative intensity): 512 $[M+H]^+$ (70), 534 $[M+Na]^+$ (100). **HR-MS** (ESI) *m*/*z* calcd for $C_{28}H_{27}F_2NO_4S$ $[M+H]^+$: 512.1702, found: 512.1707.

Compound **10** was obtained following the general procedure with **1a** (56 mg, 0.3 mmol), 2-phenylpyrimidine (140 mg, 0.9 mmol), and ethyl 2-bromo-2,2-difluoroacetate (183 mg, 0.9 mmol) for 24 h. Purification by column chromatography on silica gel (*n*-hexane/EtOAc = 10/1→5/1) yielded **10** (84 mg, 61%) with two separable diastereomers (*cis*: *trans* = 3.1:1). **R**$_f$ = 0.10 for *cis*-**10** and 0.13 for *trans*-**10** (TLC: *n*-hexane/EtOAc = 5/1).

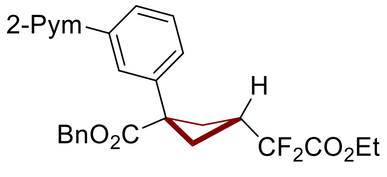


**Benzyl (1s,3s)-3-(2-ethoxy-1,1-difluoro-2-oxoethyl)-1-(3-(pyrimidin-2-yl)phenyl)cyclobutane-1-carboxylate** (*cis*-**10**, 64 mg, 46%) was obtained as a colorless oil. **$^1$H NMR** (500 MHz, $CDCl_3$) δ = 8.83 (d, *J* = 4.8 Hz, 2H), 8.58 (t, *J* = 1.9 Hz, 1H), 8.40 (dt, *J* = 7.6, 1.5 Hz, 1H), 7.55 (dt, *J* = 7.8, 1.6 Hz, 1H), 7.51 (t, *J* = 7.7 Hz, 1H), 7.30 – 7.25 (m, 3H), 7.23 – 7.19 (m, 3H), 5.10 (s, 2H), 4.31 (q, *J* = 7.1 Hz, 2H), 3.16 – 3.10 (m, 2H), 2.96 – 2.79 (m, 3H), 1.34 (t, *J* = 7.1 Hz, 3H). **$^{13}$C NMR** (126 MHz, $CDCl_3$) δ = 174.3 ($C_q$), 164.4 ($C_q$), 163.8 (t, *J* = 33.3 Hz, $C_q$), 157.4 (CH), 141.5 ($C_q$), 138.1 ($C_q$), 135.9 ($C_q$), 129.3 (CH), 129.1 (CH), 128.5 (CH), 128.1 (CH), 127.8 (CH), 127.3 (CH), 126.5 (CH), 119.4 (CH), 115.5 (t, *J* = 250.2 Hz, $C_q$), 66.9 ($CH_2$), 63.0 ($CH_2$), 47.9 ($C_q$), 32.1 (t, *J* = 25.7 Hz, CH), 31.2 (t, *J* = 4.9 Hz, $CH_2$), 14.1 ($CH_3$). **$^{19}$F NMR** (471 MHz, $CDCl_3$) δ = -114.25 (d, *J* = 15.2 Hz). **IR** (ATR): 2961, 1763, 1729, 1562, 1414, 1225, 1038, 700 cm$^{-1}$. **MS** (ESI) *m*/*z* (relative intensity): 467 $[M+H]^+$ (50), 489 $[M+Na]^+$ (100). **HR-MS** (ESI) *m*/*z* calcd for $C_{26}H_{24}N_2O_4F_2$ $[M+H]^+$: 467.1777, found: 467.1774.

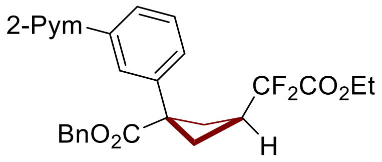


**Benzyl**

**(1r,3r)-3-(2-ethoxy-1,1-difluoro-2-oxoethyl)-1-(3-(pyrimidin-2-yl)phenyl)cyclobutane-1-carboxylate** (*trans*-**10**, 20 mg, 14%) was obtained as a colorless oil. **$^1$H NMR** (500 MHz, $CDCl_3$) δ = 8.81 (d, *J* = 4.8 Hz, 2H), 8.39 – 8.33 (m, 2H), 7.46 (td, *J* = 7.6, 1.1 Hz, 1H), 7.36 (dd, *J* = 7.9, 1.4 Hz, 1H), 7.27 – 7.24 (m, 3H), 7.20 (t, *J* = 4.8 Hz, 1H), 7.18 – 7.15 (m, 2H), 5.12 (s, 2H), 4.28 (q, *J* = 7.1 Hz, 2H), 3.19 – 3.00 (m, 3H), 2.80 – 2.72 (m, 2H), 1.32 (t, *J* = 7.2 Hz, 3H). **$^{13}$C NMR** (126 MHz, $CDCl_3$) δ = 174.7 ($C_q$), 164.5 ($C_q$), 163.8 (t, *J* = 33.4 Hz, $C_q$), 157.4 (CH), 143.3 ($C_q$), 137.9 ($C_q$), 135.9 ($C_q$), 128.9 (CH), 128.6 (CH), 128.2 (CH), 127.6 (CH), 127.1 (CH), 125.9 (CH), 119.4 (CH), 115.0 (t, *J* = 250.0 Hz, $C_q$), 67.2 ($CH_2$), 63.0 ($CH_2$), 48.5 ($C_q$), 33.6 (t, *J* = 25.9 Hz, CH), 31.7 (t, *J* = 5.0 Hz, $CH_2$), 14.1 ($CH_3$). **$^{19}$F NMR** (471 MHz, $CDCl_3$) δ = -114.34 (d, *J* = 14.9 Hz). **IR** (ATR): 2960, 1763, 1727, 1562, 1415, 1225, 1057, 701 $cm^{-1}$. **MS** (ESI) *m*/*z* (relative intensity): 467 $[M+H]^+$ (20), 489 $[M+Na]^+$ (100). **HR-MS** (ESI) *m*/*z* calcd for $C_{26}H_{24}N_2O_4F_2$ $[M+H]^+$: 467.1777, found: 467.1771.

**Compound 11** was obtained following the general procedure with **1a** (56 mg, 0.3 mmol), 5-methyl-2-phenylpyrimidine (153 mg, 0.9 mmol), and ethyl 2-bromo-2,2-difluoroacetate (183 mg, 0.9 mmol) for 36 h. Purification by column chromatography on silica gel (*n*-hexane/EtOAc = 10/1→5/1) yielded **11** (90 mg, 63%) with two separable diastereomers (*cis*: *trans* = 3.2:1). **R**$_f$ = 0.18 for *cis*-**11** and 0.20 for *trans*-**11** (TLC: *n*-hexane/EtOAc = 5/1 for 2 times).

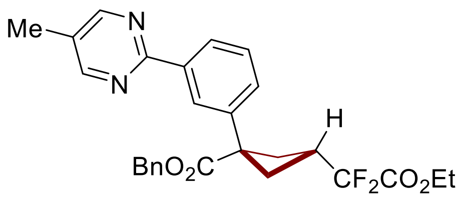


**Benzyl (1s,3s)-3-(2-ethoxy-1,1-difluoro-2-oxoethyl)-1-(3-(5-methylpyrimidin-2-yl)phenyl) cyclobutane-1-carboxylate** (*cis*-**11**, 69 mg, 48%) was obtained as a colorless oil. **$^1$H NMR** (500 MHz, $CDCl_3$) δ = 8.64 (d, *J* = 0.8 Hz, 2H), 8.53 (t, *J* = 1.6 Hz, 1H), 8.34 (dt, *J* = 7.4, 1.6 Hz, 1H), 7.53 – 7.44 (m, 2H), 7.26 – 7.24 (m, 3H), 7.20 – 7.17 (m,

2H), 5.08 (s, 2H), 4.29 (q, *J* = 7.2 Hz, 2H), 3.13 – 3.08 (m, 2H), 2.93 – 2.79 (m, 3H), 2.35 (s, 3H), 1.33 (t, *J* = 7.1 Hz, 3H). **$^{13}$C NMR** (126 MHz, $CDCl_3$) δ = 174.3 ($C_q$), 163.8 (t, *J* = 33.3 Hz, $C_q$), 162.1 ($C_q$), 157.5 (CH), 141.4 ($C_q$), 138.1 ($C_q$), 135.9 ($C_q$), 129.0 (CH), 128.8 (CH), 128.7 ($C_q$), 128.5 (CH), 128.1 (CH), 127.7 (CH), 127.1 (CH), 126.2 (CH), 115.5 (t, *J* = 250.1 Hz, $C_q$), 66.9 ($CH_2$), 63.0 ($CH_2$), 47.9 ($C_q$), 32.1 (t, *J* = 25.8 Hz, CH), 31.2 (t, *J* = 4.9 Hz, $CH_2$), 15.7 ($CH_3$), 14.1 ($CH_3$). **$^{19}$F NMR** (471 MHz, $CDCl_3$) δ = -114.25 (d, *J* = 15.3 Hz). **IR** (ATR): 2957, 1730, 1551, 1429, 1219, 1166, 1039, 782, 701 $cm^{-1}$. **MS** (ESI) m/z (relative intensity): 481 $[M+H]^+$ (40), 503 $[M+Na]^+$ (100). **HR-MS** (ESI) m/z calcd for $C_{27}H_{26}N_2O_4F_2$ $[M+H]^+$: 481.1933, found: 481.1941.

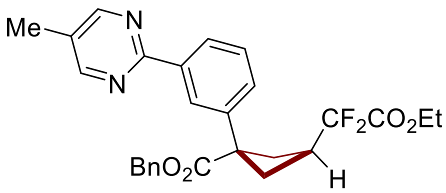


**Benzyl (1r,3r)-3-(2-ethoxy-1,1-difluoro-2-oxoethyl)-1-(3-(5-methylpyrimidin-2-yl)phenyl) cyclobutane-1-carboxylate** (*trans*-**11**, 21 mg, 15%) was obtained as a white solid. Recrystallization on DCM/ *n*-hexane. **$^1$H NMR** (400 MHz, $CDCl_3$) δ = 8.64 (d, *J* = 0.9 Hz, 2H), 8.32 – 8.28 (m, 2H), 7.46 – 7.42 (m, 1H), 7.34 – 7.31 (m, 1H), 7.26 – 7.22 (m, 3H), 7.18 – 7.14 (m, 2H), 5.11 (s, 2H), 4.28 (q, *J* = 7.1 Hz, 2H), 3.18 – 3.00 (m, 3H), 2.80 – 2.73 (m, 2H), 2.35 (s, 3H), 1.31 (t, *J* = 7.2 Hz, 3H). **$^{13}$C NMR** (101 MHz, $CDCl_3$) δ = 174.7 ($C_q$), 163.8 (t, *J* = 33.4 Hz, $C_q$), 162.2 ($C_q$), 157.5 (CH), 143.2 ($C_q$), 137.9 ($C_q$), 135.9 ($C_q$), 128.8 (CH), 128.6 ($C_q$), 128.6 (CH), 128.2 (CH), 128.2 (CH), 127.6 (CH), 126.8 (CH), 125.6 (CH), 115.0 (t, *J* = 250.5 Hz, $C_q$), 67.1 ($CH_2$), 63.0 ($CH_2$), 48.6 ($C_q$), 33.6 (t, *J* = 26.0 Hz, CH), 31.7 (t, *J* = 5.0 Hz, $CH_2$), 15.7 ($CH_3$), 14.1 ($CH_3$). **$^{19}$F NMR** (377 MHz, $CDCl_3$) δ = -114.30 (d, *J* = 14.6 Hz). **IR** (ATR): 2956, 1730, 1551, 1430, 1215, 1125, 1024, 782, 699 $cm^{-1}$. **MS** (ESI) m/z (relative intensity): 481 $[M+H]^+$ (30), 503 $[M+Na]^+$ (100). **HR-MS** (ESI) m/z calcd for $C_{27}H_{26}N_2O_4F_2$ $[M+Na]^+$: 503.1753, found: 503.1754.

**Compound 12** was obtained following the general procedure with **1a** (56 mg, 0.3 mmol), 5-fluoro-2-phenylpyrimidine (157 mg, 0.9 mmol), and ethyl 2-bromo-2,2-difluoroacetate (183 mg, 0.9 mmol) for 24 h. Purification by column chromatography on silica gel (*n*-hexane/EtOAc/DCM = 25/1/1→20/1/1) yielded **12** (84 mg, 58%) with two separable diastereomers (*cis*: *trans* = 2.7:1). **R**$_f$ = 0.47 for *cis*-**12** and 0.50 for *trans*-**12** (TLC: *n*-hexane/EtOAc/DCM = 5/1/1).

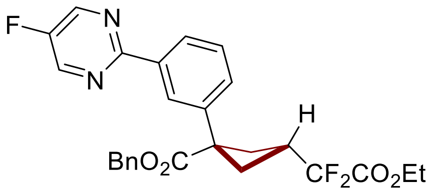


**Benzyl (1s,3s)-3-(2-ethoxy-1,1-difluoro-2-oxoethyl)-1-(3-(5-fluoropyrimidin-2-yl)phenyl) cyclobutane-1-carboxylate** (*cis*-**12,** 61 mg, 42%) was obtained as a colorless oil. **$^1$H NMR** (500 MHz, $CDCl_3$) δ = 8.66 (s, 2H), 8.50 (t, *J* = 1.9 Hz, 1H), 8.32 (dt, *J* = 7.6, 1.5 Hz, 1H), 7.56 – 7.45 (m, 2H), 7.28 – 7.23 (m, 3H), 7.21 – 7.15 (m, 2H), 5.08 (s, 2H), 4.30 (q, *J* = 7.1 Hz, 2H), 3.14 – 3.08 (m, 2H), 2.94 – 2.75 (m, 3H), 1.33 (t, *J* = 7.1 Hz, 3H). **$^{13}$C NMR** (126 MHz, $CDCl_3$) δ = 174.2 ($C_q$), 163.8 (t, *J* = 33.3 Hz, $C_q$), 160.7 (d, *J* = 5.4 Hz, $C_q$), 156.9 (d, *J* = 264.9 Hz, $C_q$), 145.1 (d, *J* = 20.2 Hz, CH), 141.6 ($C_q$), 137.1 ($C_q$), 135.9 ($C_q$), 129.1 (CH), 129.1 (CH), 128.5 (CH), 128.2 (CH), 127.8 (CH), 127.3 (CH), 126.5 (CH), 115.5 (t, *J* = 250.2 Hz, $C_q$), 67.0 ($CH_2$), 63.0 ($CH_2$), 47.9 ($C_q$), 32.1 (t, *J* = 25.7 Hz, CH), 31.2 (t, *J* = 4.9 Hz, $CH_2$), 14.1 ($CH_3$). **$^{19}$F NMR** (471 MHz, $CDCl_3$) δ = -114.25 (d, *J* = 15.2 Hz), -140.24. **IR** (ATR): 2960, 1763, 1729, 1418, 1220, 1127, 1038, 779, 699 $cm^{-1}$. **MS** (ESI) m/z (relative intensity): 485 $[M+H]^+$ (5), 507 $[M+Na]^+$ (100). **HR-MS** (ESI) m/z calcd for $C_{26}H_{23}N_2O_4F_3$ $[M+Na]^+$: 507.1502, found: 507.1509.

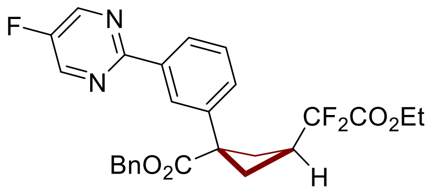


**Benzyl (1r,3r)-3-(2-ethoxy-1,1-difluoro-2-oxoethyl)-1-(3-(5-fluoropyrimidin-2-yl)phenyl) cyclobutane-1-carboxylate** (*trans*-**12,** 23 mg, 16%) was obtained as a colorless oil. **$^1$H NMR** (400 MHz, $CDCl_3$) δ = 8.65 (s, 2H), 8.29 (dd, *J* = 7.3, 1.4 Hz, 2H), 7.50 – 7.41 (m, 1H), 7.36 – 7.33 (m, 1H), 7.29 – 7.22 (m, 3H), 7.20 – 7.13 (m, 2H), 5.12 (s, 2H), 4.29 (q, *J* = 7.2 Hz, 2H), 3.21 – 2.98 (m, 3H), 2.79 – 2.70 (m, 2H), 1.32 (t, *J* = 7.2 Hz, 3H). **$^{13}$C NMR** (101 MHz, $CDCl_3$) δ = 174.7 ($C_q$), 163.8 (t, *J* = 33.3 Hz, $C_q$), 160.8 (d, *J* = 5.5 Hz, $C_q$), 156.9 (d, *J* = 264.9 Hz, $C_q$), 145.1 (d, *J* = 20.2 Hz, CH), 145.0, 143.3 ($C_q$), 137.0 ($C_q$), 135.9 ($C_q$), 128.9 (CH), 128.6 (CH), 128.4 (CH), 128.2 (CH), 127.6 (CH), 127.0 (CH), 125.9 (CH), 115.0 (t, *J* = 250.1 Hz, $C_q$), 67.2 ($CH_2$), 63.0 ($CH_2$), 48.5 ($C_q$), 33.6 (t, *J* = 26.0 Hz, CH), 31.6 (t, *J* = 5.1 Hz, $CH_2$), 14.1 ($CH_3$). $^{19}$F NMR (377 MHz, $CDCl_3$) δ = -114.36 (d, *J* = 14.6 Hz), -140.37. **IR** (ATR): 2960, 1763, 1727, 1418, 1213, 1122, 1054, 730, 698 $cm^{-1}$. **MS** (ESI) m/z (relative intensity): 485 $[M+H]^+$ (10), 507 $[M+Na]^+$ (100). **HR-MS** (ESI) m/z calcd for $C_{26}H_{23}N_2O_4F_3$ $[M+Na]^+$: 507.1502, found: 507.1507.

**Compound 13** was obtained following the general procedure with **1a** (56 mg, 0.3 mmol), 5-methoxy-2-phenylpyrimidine (167 mg, 0.9 mmol), and ethyl 2-bromo-2,2-difluoroacetate (183 mg, 0.9 mmol) for 24 h. Purification by column chromatography on silica gel (*n*-hexane/EtOAc/DCM = 20/1/1→8/1/1) yielded **13** (88 mg, 59%) with two separable diastereomers (*cis*: *trans* = 3.4:1). **R**$_f$= 0.27 for *cis*-**13** and 0.30 for *trans*-**13** (TLC: *n*-hexane/EtOAc/DCM = 5/1/1).

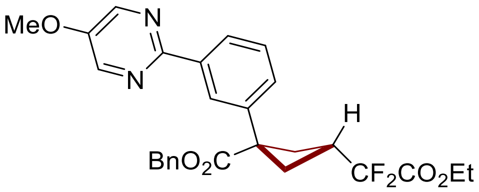

**Benzyl (1s,3s)-3-(2-ethoxy-1,1-difluoro-2-oxoethyl)-1-(3-(5-methoxypyrimidin-2-yl)phenyl)cyclobutane-1-carboxylate** (*cis*-**13**, 68 mg, 46%) was obtained as a white solid. **M.p.**: 99 – 101 °C. **$^1$H NMR** (400 MHz, $CDCl_3$) δ = 8.51 – 8.48 (m, 3H), 8.34 – 8.27 (m, 1H), 7.50 – 7.44 (m, 2H), 7.30 – 7.24 (m, 3H), 7.22 – 7.18 (m, 2H), 5.10 (s, 2H), 4.31 (q, *J* = 7.1 Hz, 2H), 3.98 (s, 3H), 3.15 – 3.08 (m, 2H), 2.97 – 2.79 (m, 3H), 1.34 (t, *J* = 7.1 Hz, 3H). **$^{13}$C NMR** (101 MHz, $CDCl_3$) δ = 174.3 ($C_q$), 163.8 (t, *J* = 33.5 Hz, $C_q$), 157.5 ($C_q$), 152.2 ($C_q$), 143.5 (CH), 141.4 ($C_q$), 137.9 ($C_q$), 135.9 ($C_q$), 129.0 (CH), 128.5 (CH), 128.2 (CH), 128.1 (CH), 127.7 (CH), 126.7 (CH), 125.9 (CH), 115.5 (t, *J* = 251.5 Hz, $C_q$), 66.9 ($CH_2$), 63.0 ($CH_2$), 56.2 ($CH_3$), 47.9 ($C_q$), 32.2 (t, *J* = 25.8 Hz, CH), 31.2 (t, *J* = 4.9 Hz, $CH_2$), 14.1 ($CH_3$). **$^{19}$F NMR** (376 MHz, $CDCl_3$) δ = -114.24 (d, *J* = 15.0 Hz). **IR** (ATR): 2959, 1730, 1548, 1421, 1276, 1127, 1013, 738, 700 $cm^{-1}$. **MS** (ESI) m/z (relative intensity): 497 $[M+H]^+$ (15), 519 $[M+Na]^+$ (100). **HR-MS** (ESI) m/z calcd for $C_{27}H_{26}N_2O_5F_2$ $[M+H]^+$: 497.1883, found: 497.1885.

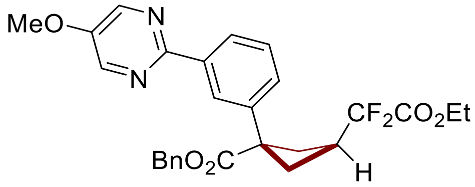


**Benzyl (1r,3r)-3-(2-ethoxy-1,1-difluoro-2-oxoethyl)-1-(3-(5-methoxypyrimidin-2-yl)phenyl)cyclobutane-1-carboxylate** (*trans*-**13**, 20 mg, 13%) was obtained as a colorless oil. **$^1$H NMR** (500 MHz, $CDCl_3$) δ = 8.48 – 8.47 (m, 2H), 8.27 – 8.25 (m, 2H), 7.45 – 7.41 (m, 1H), 7.31 – 7.28 (m, 1H), 7.27 – 7.23 (m, 3H), 7.18 – 7.15 (m, 2H), 5.11 (s, 2H), 4.28 (q, *J* = 7.2 Hz, 2H), 3.96 (s, 3H), 3.18 – 2.99 (m, 3H), 2.80 – 2.73 (m, 2H), 1.31 (t, *J* = 7.1 Hz, 3H). **$^{13}$C NMR** (126 MHz, $CDCl_3$) δ = 174.8 ($C_q$), 163.8 (t, *J* = 33.5 Hz, $C_q$), 157.6 ($C_q$), 152.2 ($C_q$), 143.5 (CH), 143.2 ($C_q$), 137.7 ($C_q$), 135.9 ($C_q$), 128.8 (CH), 128.6 (CH), 128.2 (CH), 127.6 (CH), 127.6 (CH), 126.4 (CH), 125.3 (CH), 115.0 (t, *J* = 250.0 Hz, $C_q$), 67.1 ($CH_2$), 63.0 ($CH_2$), 56.2 ($CH_3$), 48.6 ($C_q$), 33.6

(t, $J$ = 25.9 Hz, CH), 31.7 (t, $J$ = 4.9 Hz, $CH_2$), 14.1 ($CH_3$). **$^{19}$F NMR** (471 MHz, $CDCl_3$) δ = -114.31 (d, $J$ = 14.8 Hz). **IR** (ATR): 2961, 1728, 1548, 1423, 1276, 1123, 1017, 743, 699 $cm^{-1}$. **MS** (ESI) m/z (relative intensity): 497 $[M+H]^+$ (15), 519 $[M+Na]^+$ (100). **HR-MS** (ESI) m/z calcd for $C_{27}H_{26}N_2O_5F_2$ $[M+H]^+$: 497.1883, found: 497.1885.

Compound **14** was obtained following the general procedure with **1a** (56 mg, 0.3 mmol), methyl 2-phenylpyrimidine-5-carboxylate (193 mg, 0.9 mmol), and ethyl 2-bromo-2,2-difluoroacetate (183 mg, 0.9 mmol) for 24 h. Purification by column chromatography on silica gel (*n*-hexane/EtOAc/DCM = 10/1/1→8/1/1) yielded **14** (108 mg, 69%) with two separable diastereomers (*cis*: *trans* = 3.1:1). **R**$_f$ = 0.33 for *cis*-**14** and 0.35 for *trans*-**14** (TLC: *n*-hexane/EtOAc/DCM = 5/1/1).

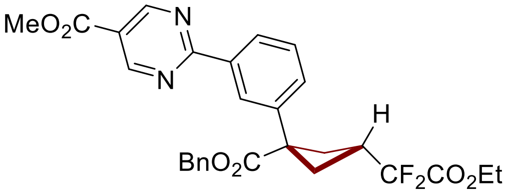


**Methyl 2-(3-((1s,3s)-1-((benzyloxy)carbonyl)-3-(2-ethoxy-1,1-difluoro-2-oxoethyl)cyclobutyl)phenyl)pyrimidine-5-carboxylate** (*cis*-**14**, 82 mg, 52%) was obtained as a white solid. **M.p.**: 103 – 105 °C. **$^1$H NMR** (500 MHz, $CDCl_3$) δ = 9.32 (s, 2H), 8.63 (t, $J$ = 1.7 Hz, 1H), 8.49 – 8.43 (m, 1H), 7.61 – 7.55 (m, 1H), 7.51 (t, $J$ = 7.7 Hz, 1H), 7.28 – 7.23 (m, 3H), 7.21 – 7.16 (m, 2H), 5.09 (s, 2H), 4.30 (q, $J$ = 7.1 Hz, 2H), 4.01 (s, 3H), 3.15 – 3.09 (m, 2H), 2.96 – 2.78 (m, 3H), 1.33 (t, $J$ = 7.1 Hz, 3H). **$^{13}$C NMR** (126 MHz, $CDCl_3$) δ = 174.2 ($C_q$), 166.9 ($C_q$), 164.6 ($C_q$), 163.8 (t, $J$ = 33.3 Hz, $C_q$), 158.6 (CH), 141.8 ($C_q$), 137.1 ($C_q$), 135.8 ($C_q$), 130.3 (CH), 129.2 (CH), 128.5 (CH), 128.2 (CH), 127.8 (CH), 127.3 (CH), 121.8 ($C_q$), 115.4 (t, $J$ = 250.3 Hz, $C_q$), 67.0 ($CH_2$), 63.0 ($CH_2$), 52.8 ($CH_3$), 47.9 ($C_q$), 32.1 (t, $J$ = 25.7 Hz, CH), 31.2 (t, $J$ = 4.9 Hz, $CH_2$), 14.1 ($CH_3$). **$^{19}$F NMR** (471 MHz, $CDCl_3$) δ = -114.25 (d, $J$ = 15.2 Hz). **IR** (ATR): 2956, 1727, 1584, 1424, 1295, 1128, 1037, 732, 698 $cm^{-1}$. **MS** (ESI) m/z (relative

intensity): 525 $[M+H]^+$ (10), 547 $[M+Na]^+$ (100). **HR-MS** (ESI) m/z calcd for $C_{28}H_{26}N_2O_6F_2$ $[M+H]^+$: 525.1832, found: 525.1831.

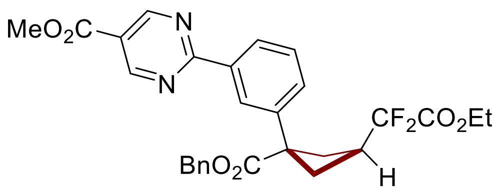


**Methyl 2-(3-((1r,3r)-1-((benzyloxy)carbonyl)-3-(2-ethoxy-1,1-difluoro-2-oxoethyl)cyclobutyl)phenyl)pyrimidine-5-carboxylate** (*trans*-**14**, 26 mg, 17%) was obtained as a colorless oil. **$^1$H NMR** (500 MHz, $CDCl_3$) δ = 9.32 – 9.31 (m, 2H), 8.45 – 8.41 (m, 2H), 7.48 (t, $J$ = 7.7 Hz, 1H), 7.42 – 7.39 (m, 1H), 7.27 – 7.23 (m, 3H), 7.19 – 7.15 (m, 2H), 5.12 (s, 2H), 4.29 (q, $J$ = 7.1 Hz, 2H), 4.00 (s, 3H), 3.20 – 3.02 (m, 3H), 2.80 – 2.73 (m, 2H), 1.32 (t, $J$ = 7.1 Hz, 3H). **$^{13}$C NMR** (126 MHz, $CDCl_3$) δ = 174.6 ($C_q$), 167.0 ($C_q$), 164.6 ($C_q$), 163.8 (t, $J$ = 33.4 Hz, $C_q$), 158.5 (CH), 143.5 ($C_q$), 136.9 ($C_q$), 135.8 ($C_q$), 129.7 (CH), 129.0 (CH), 128.6 (CH), 128.2 (CH), 127.9 (CH), 127.7 (CH), 126.7 (CH), 121.8 ($C_q$), 114.9 (t, $J$ = 249.9 Hz, $C_q$), 67.2 ($CH_2$), 63.0 ($CH_2$), 52.8 ($CH_3$), 48.5 ($C_q$), 33.6 (t, $J$ = 26.0 Hz, CH), 31.7 (t, $J$ = 4.9 Hz, $CH_2$), 14.1 ($CH_3$). **$^{19}$F NMR** (471 MHz, $CDCl_3$) δ = -114.39 (d, $J$ = 14.8 Hz). **IR** (ATR): 2957, 1727, 1585, 1415, 1298, 1127, 1060, 731, 697 $cm^{-1}$. **MS** (ESI) m/z (relative intensity): 525 $[M+H]^+$ (20), 547 $[M+Na]^+$ (100). **HR-MS** (ESI) m/z calcd for $C_{28}H_{26}N_2O_6F_2$ $[M+H]^+$: 525.1832, found: 525.1832.

Compound **15** was obtained following the general procedure with **1a** (56 mg, 0.3 mmol), 2-(p-tolyl)pyrimidine (153 mg, 0.9 mmol), and ethyl 2-bromo-2,2-difluoroacetate (183 mg, 0.9 mmol) for 24 h. Purification by column chromatography on silica gel (*n*-hexane/EtOAc = 10/1→5/1) yielded **15** (74 mg, 51%) with two separable diastereomers (*cis*: *trans* = 5.2:1). **$R_f$** = 0.24 for *cis*-**15** and 0.26 for *trans*-**15** (TLC: *n*-hexane/EtOAc = 3/1).

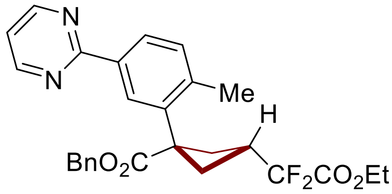


**Benzyl (1s,3s)-3-(2-ethoxy-1,1-difluoro-2-oxoethyl)-1-(2-methyl-5-(pyrimidin-2-yl)phenyl)cyclobutane-1-carboxylate** (*cis*-**15**, 62 mg, 43%) was obtained as a colorless oil. **$^1$H NMR** (400 MHz, $CDCl_3$) δ = 8.82 (d, *J* = 4.8 Hz, 2H), 8.59 (d, *J* = 1.7 Hz, 1H), 8.31 (dd, *J* = 7.9, 1.8 Hz, 1H), 7.31 – 7.24 (m, 4H), 7.22 – 7.15 (m, 3H), 5.12 (s, 2H), 4.32 (q, *J* = 7.1 Hz, 2H), 3.21 – 2.80 (m, 5H), 2.18 (s, 3H), 1.36 (t, *J* = 7.1 Hz, 3H). **$^{13}$C NMR** (101 MHz, $CDCl_3$) δ = 174.1 ($C_q$), 164.6 ($C_q$), 163.9 (t, *J* = 33.3 Hz, $C_q$), 157.3 (CH), 140.1 ($C_q$), 139.2 ($C_q$), 136.0 ($C_q$), 135.5 ($C_q$), 132.2 (CH), 128.5 (CH), 128.1 (CH), 127.8 (CH), 127.3 (CH), 126.2 (CH), 119.1 (CH), 115.6 (t, *J* = 250.4 Hz, $C_q$), 66.9 ($CH_2$), 63.0 ($CH_2$), 48.0 ($C_q$), 32.3 (t, *J* = 25.7 Hz, CH), 30.5 (t, *J* = 4.9 Hz, $CH_2$), 19.9 ($CH_3$), 14.1 ($CH_3$). **$^{19}$F NMR** (377 MHz, $CDCl_3$) δ = -113.95 (d, *J* = 15.3 Hz). **IR** (ATR): 2962, 1728, 1560, 1420, 1314, 1129, 1039, 802, 737 $cm^{-1}$. **MS** (ESI) m/z (relative intensity): 481 $[M+H]^+$ (10), 503 $[M+Na]^+$ (100). **HR-MS** (ESI) m/z calcd for $C_{27}H_{26}N_2O_4F_2$ $[M+Na]^+$: 503.1753, found: 503.1757.

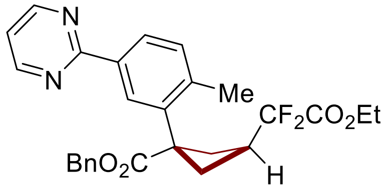


**Benzyl (1r,3r)-3-(2-ethoxy-1,1-difluoro-2-oxoethyl)-1-(2-methyl-5-(pyrimidin-2-yl)phenyl)cyclobutane-1-carboxylate** (*trans*-**15**, 12 mg, 8%) was obtained as a white solid. **M.p.**: 118 – 120 °C. Recrystallization on DCM/ *n*-hexane. **$^1$H NMR** (400 MHz, $CDCl_3$) δ = 8.80 (d, *J* = 4.8 Hz, 2H), 8.32 (d, *J* = 1.8 Hz, 1H), 8.26 (dd, *J* = 7.9, 1.8 Hz, 1H), 7.26 – 7.21 (m, 4H), 7.17 (t, *J* = 4.8 Hz, 1H), 7.15 – 7.07 (m, 2H), 5.12 (s, 2H), 4.30 (q, *J* = 7.1 Hz, 2H), 3.29 – 3.13 (m, 1H), 3.04 – 2.96 (m, 2H), 2.85 – 2.74 (m, 2H), 2.16 (s, 3H), 1.33 (t, *J* = 7.1 Hz, 3H). **$^{13}$C NMR** (101 MHz, $CDCl_3$) δ = 174.8

($C_q$), 163.6 (t, $J$ = 33.3 Hz, $C_q$), 157.3 (CH), 140.9 ($C_q$), 139.1 ($C_q$), 136.0 ($C_q$), 135.6 ($C_q$), 131.6 (CH), 128.6 (CH), 128.2 (CH), 127.6 (CH), 127.2 (CH), 126.9 (CH), 119.1 (CH), 114.9 (t, $J$ = 249.9 Hz, $C_q$), 67.0 ($CH_2$), 63.0 ($CH_2$), 48.9 ($C_q$), 33.7 (t, $J$ = 25.9 Hz, CH), 31.9 (t, $J$ = 5.0 Hz, $CH_2$) 19.9 ($CH_3$), 14.1 ($CH_3$). **$^{19}$F NMR** (377 MHz, $CDCl_3$) δ = -114.31 (d, $J$ = 14.9 Hz). **IR** (ATR): 2961, 1726, 1561, 1421, 1313, 1125, 1059, 804, 740 $cm^{-1}$. **MS** (ESI) m/z (relative intensity): 481 $[M+H]^+$ (20), 503 $[M+Na]^+$ (100). **HR-MS** (ESI) m/z calcd for $C_{27}H_{26}N_2O_4F_2$ $[M+H]^+$: 481.1933, found: 481.1936.

Compound **16** was obtained following the general procedure with **1a** (56 mg, 0.3 mmol), 2-(4-bromophenyl)pyrimidine (212 mg, 0.9 mmol), and ethyl 2-bromo-2,2-difluoroacetate (183 mg, 0.9 mmol) for 24 h. Purification by column chromatography on silica gel ($n$-hexane/EtOAc = 10/1→5/1) yielded **16** (77 mg, 47%) with two separable diastereomers (*cis*: *trans* = 3.3:1). **R**$_f$ = 0.24 for *cis*-**16** and 0.27 for *trans*-**16** (TLC: $n$-hexane/EtOAc = 3/1).

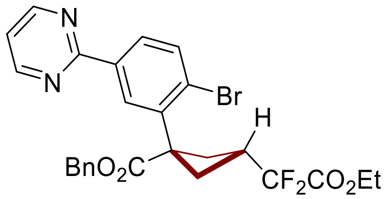


**Benzyl (1s,3s)-1-(2-bromo-5-(pyrimidin-2-yl)phenyl)-3-(2-ethoxy-1,1-difluoro-2-oxoethyl)cyclobutane-1-carboxylate** (*cis*-**16**, 59 mg, 36%) was obtained as a colorless oil. **$^1$H NMR** (500 MHz, $CDCl_3$) δ = 8.81 (d, $J$ = 4.8 Hz, 2H), 8.69 (d, $J$ = 2.1 Hz, 1H), 8.28 (dd, $J$ = 8.3, 2.1 Hz, 1H), 7.71 (d, $J$ = 8.3 Hz, 1H), 7.30 – 7.25 (m, 3H), 7.24 – 7.19 (m, 3H), 5.13 (s, 2H), 4.30 (q, $J$ = 7.1 Hz, 2H), 3.20 – 3.14 (m, 2H), 2.98 – 2.87 (m, 1H), 2.82 – 2.75 (m, 2H), 1.33 (t, $J$ = 7.1 Hz, 3H). **$^{13}$C NMR** (126 MHz, $CDCl_3$) δ = 173.2 ($C_q$), 163.8 (t, $J$ = 32.8 Hz, $C_q$), 163.8 ($C_q$), 157.4 (CH), 139.9 ($C_q$), 137.0 ($C_q$), 135.8 ($C_q$), 134.5 (CH), 128.7 (CH), 128.5 (CH), 128.1 (CH), 128.0 (CH), 127.7 ($C_q$), 127.4 (CH), 119.6 (CH), 115.2 (t, $J$ = 250.6 Hz, $C_q$), 67.3 ($CH_2$), 63.0 ($CH_2$), 49.3 ($C_q$),

32.6 (t, $J$ = 25.9 Hz, CH), 30.3 (t, $J$ = 5.0 Hz, $CH_2$), 14.1 ($CH_3$). **$^{19}$F NMR** (471 MHz, $CDCl_3$) δ = -113.70 (d, $J$ = 14.8 Hz). **IR** (ATR): 2965, 1729, 1560, 1419, 1315, 1219, 1017, 802, 703 $cm^{-1}$. **MS** (ESI) *m*/*z* (relative intensity): 567 $[M+Na]^+$ (100) ($^{79}$Br), 569 $[M+Na]^+$ (97) ($^{81}$Br). **HR-MS** (ESI) *m*/*z* calcd for $C_{26}H_{23}N_2O_4BrF_2$ $[M+Na]^+$: 567.0701, found: 567.0710.

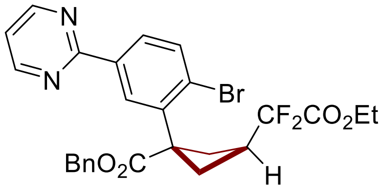


**Benzyl (1r,3r)-1-(2-bromo-5-(pyrimidin-2-yl)phenyl)-3-(2-ethoxy-1,1-difluoro-2-oxoethyl)cyclobutane-1-carboxylate** (*trans*-**16**, 18 mg, 11%) was obtained as a white solid. **M.p.**: 105 – 107 °C. Recrystallized on DCM/*n*-hexane. **$^1$H NMR** (400 MHz, $CDCl_3$) δ = 8.81 (d, $J$ = 4.8 Hz, 2H), 8.41 (d, $J$ = 2.1 Hz, 1H), 8.24 (dd, $J$ = 8.3, 2.1 Hz, 1H), 7.64 (d, $J$ = 8.3 Hz, 1H), 7.29 – 7.26 (m, 1H), 7.26 – 7.24 (m, 2H), 7.22 (t, $J$ = 4.8 Hz, 1H), 7.19 – 7.15 (m, 2H), 5.15 (s, 2H), 4.30 (q, $J$ = 7.2 Hz, 2H), 3.43 – 3.30 (m, 1H), 3.08 – 3.00 (m, 2H), 2.84 – 2.76 (m, 2H), 1.33 (t, $J$ = 7.1 Hz, 3H). **$^{13}$C NMR** (101 MHz, $CDCl_3$) δ = 174.3 ($C_q$), 163.8 ($C_q$), 157.4 (CH), 142.1 ($C_q$), 136.9 ($C_q$), 135.8 ($C_q$), 133.9 (CH), 128.6 (CH), 128.5 (CH), 128.4 (CH), 128.2 (CH), 127.8 (CH), 125.8 ($C_q$), 119.6 (CH), 115.0 (t, $J$ = 251.5 Hz, $C_q$), 67.3 ($CH_2$), 63.0 ($CH_2$), 49.9 ($C_q$), 33.1 (t, $J$ = 25.8 Hz, CH), 31.7 (t, $J$ = 5.1 Hz, $CH_2$), 14.1 ($CH_3$). **$^{19}$F NMR** (377 MHz, $CDCl_3$) δ = -114.51 (d, $J$ = 15.2 Hz). **IR** (ATR): 2961, 1728, 1562, 1419, 1317, 1212, 1123, 804, 705 $cm^{-1}$. **MS** (ESI) *m*/*z* (relative intensity): 567 $[M+Na]^+$ (100) ($^{79}$Br), 569 $[M+Na]^+$ (97) ($^{81}$Br). **HR-MS** (ESI) *m*/*z* calcd for $C_{26}H_{23}N_2O_4BrF_2$ $[M+Na]^+$: 567.0701, found: 567.0714.

Compound **17** was obtained following the general procedure with **1a** (56 mg, 0.3 mmol), 1-phenyl-1H-pyrazole (130 mg, 0.9 mmol), and ethyl 2-bromo-2,2-difluoroacetate (183 mg, 0.9 mmol) for 24 h. Purification by column

chromatography on silica gel (*n*-hexane/EtOAc = 10/1→5/1) yielded **17** (90 mg, 66%) with two separable diastereomers (*cis*: *trans* = 2.6:1). **R**$_f$ = 0.40 for *cis*-**17** and 0.43 for *trans*-**17** (TLC: *n*-hexane/EtOAc = 3/1).

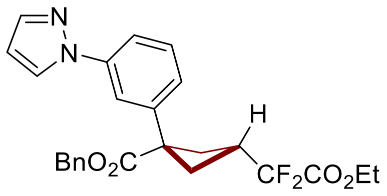

**Benzyl (1s,3s)-1-(3-(1H-pyrazol-1-yl)phenyl)-3-(2-ethoxy-1,1-difluoro-2-oxoethyl)cyclobutane-1-carboxylate** (*cis*-**17**, 65 mg, 48%) was obtained as a colorless oil. **$^1$H NMR** (500 MHz, $CDCl_3$) δ = 7.87 (d, *J* = 2.2 Hz, 1H), 7.76 (t, *J* = 2.0 Hz, 1H), 7.73 (d, *J* = 1.5 Hz, 1H), 7.61 (ddd, *J* = 8.1, 2.2, 1.0 Hz, 1H), 7.44 (t, *J* = 7.9 Hz, 1H), 7.33 (ddd, *J* = 7.8, 1.9, 1.0 Hz, 1H), 7.28 – 7.25 (m, 3H), 7.22 – 7.16 (m, 2H), 6.47 (dd, *J* = 2.5, 1.8 Hz, 1H), 5.09 (s, 2H), 4.30 (q, *J* = 7.1 Hz, 2H), 3.13 – 3.05 (m, 2H), 2.93 – 2.83 (m, 1H), 2.79 – 2.72 (m, 2H), 1.33 (t, *J* = 7.1 Hz, 3H). **$^{13}$C NMR** (126 MHz, $CDCl_3$) δ = 174.0 ($C_q$), 163.8 (t, *J* = 33.3 Hz, $C_q$), 142.8 ($C_q$), 141.4 (CH), 140.6 ($C_q$), 135.8 ($C_q$), 129.8 (CH), 128.6 (CH), 128.3 (CH), 127.9 (CH), 127.0 (CH), 124.8 (CH), 118.3 (CH), 117.9 (CH), 115.4 (t, *J* = 250.2 Hz, $C_q$), 107.9 (CH), 67.1 ($CH_2$), 63.1 ($CH_2$), 47.9 ($C_q$), 32.1 (t, *J* = 25.8 Hz, CH), 31.2 (t, *J* = 4.9 Hz, $CH_2$), 14.1 ($CH_3$). **$^{19}$F NMR** (471 MHz, $CDCl_3$) δ = -114.30 (d, *J* = 15.5 Hz). **IR** (ATR): 2982, 1729, 1599, 1308, 1223, 1125, 1039, 748, 696 cm$^{-1}$. **MS** (ESI) *m*/*z* (relative intensity): 455 $[M+H]^+$ (10), 477 $[M+Na]^+$ (100). **HR-MS** (ESI) *m*/*z* calcd for $C_{25}H_{24}N_2O_4F_2$ $[M+H]^+$: 455.1777, found: 455.1782.

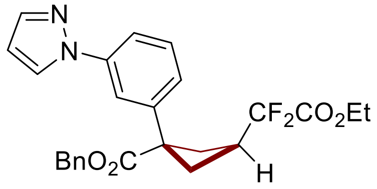

**Benzyl (1r,3r)-1-(3-(1H-pyrazol-1-yl)phenyl)-3-(2-ethoxy-1,1-difluoro-2-oxoethyl)cyclobu**

**tane-1-carboxylate** (*trans*-**17**, 25 mg, 18%) was obtained as a colorless oil. **$^1$H NMR** (500 MHz, $CDCl_3$) δ = 7.88 (dd, *J* = 2.5, 0.6 Hz, 1H), 7.73 (d, *J* = 1.8 Hz, 1H), 7.60 – 7.54 (m, 2H), 7.41 (dd, *J* = 8.8, 7.7 Hz, 1H), 7.30 – 7.26 (m, 3H), 7.20 – 7.12 (m, 3H), 6.46 (dd, *J* = 2.5, 1.8 Hz, 1H), 5.12 (s, 2H), 4.29 (q, *J* = 7.2 Hz, 2H), 3.16 – 2.98 (m, 3H), 2.76 – 2.69 (m, 2H), 1.32 (t, *J* = 7.1 Hz, 3H). **$^{13}$C NMR** (126 MHz, $CDCl_3$) δ = 174.3 ($C_q$), 163.8 (t, *J* = 34.0 Hz, $C_q$), 144.4 ($C_q$), 141.3 (CH), 140.4 ($C_q$), 135.8 ($C_q$), 129.7 (CH), 128.7 (CH), 128.3 (CH), 127.8 (CH), 127.0 (CH), 124.2 (CH), 118.0 (CH), 117.2 (CH), 114.9 (t, *J* = 249.9 Hz, $C_q$), 107.8 (CH), 67.3 ($CH_2$), 63.0 ($CH_2$), 48.5 ($C_q$), 33.5 (t, *J* = 25.9 Hz, CH), 31.6 (t, *J* = 5.0 Hz, $CH_2$), 14.1 ($CH_3$). **$^{19}$F NMR** (471 MHz, $CDCl_3$) δ = -114.46 (d, *J* = 14.8 Hz). **IR** (ATR): 2983, 1728, 1600, 1312, 1218, 1124, 1054, 750, 697 $cm^{-1}$. **MS** (ESI) *m*/*z* (relative intensity): 455 $[M+H]^+$ (20), 477 $[M+Na]^+$ (100). **HR-MS** (ESI) *m*/*z* calcd for $C_{25}H_{24}N_2O_4F_2$ $[M+H]^+$: 455.1777, found: 455.1783.

Compound **18** was obtained following the general procedure with **1a** (56 mg, 0.3 mmol), 1-(p-tolyl)-1H-pyrazole (142 mg, 0.9 mmol), and ethyl 2-bromo-2,2-difluoroacetate (183 mg, 0.9 mmol) for 24 h. Purification by column chromatography on silica gel (*n*-hexane/EtOAc/DCM = 15/1/1→10/1/1) yielded **18** (72 mg, 51%) with two separable diastereomers (*cis*: *trans* = 2.8:1). **R**$_f$ = 0.39 for *cis*-**18** and 0.45 for *trans*-**18** (TLC: *n*-hexane/EtOAc/DCM = 5/1/1).

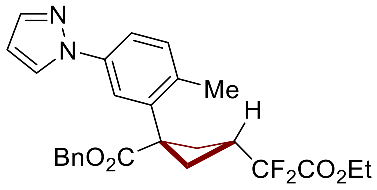


**Benzyl (1s,3s)-3-(2-ethoxy-1,1-difluoro-2-oxoethyl)-1-(2-methyl-5-(1H-pyrazol-1-yl)phenyl)cyclobutane-1-carboxylate** (*cis*-**18**, 53 mg, 38%) was obtained as a colorless oil. **$^1$H NMR** (500 MHz, $CDCl_3$) δ = 7.91 (dd, *J* = 2.5, 0.6 Hz, 1H), 7.79 (d, *J* = 2.3 Hz, 1H), 7.73 (dd, *J* = 1.8, 0.6 Hz, 1H), 7.47 (dd, *J* = 8.1, 2.3 Hz, 1H), 7.29 – 7.25 (m, 3H),

7.21 (d, *J* = 8.2 Hz, 1H), 7.18 – 7.14 (m, 2H), 6.47 (dd, *J* = 2.5, 1.8 Hz, 1H), 5.10 (s, 2H), 4.30 (q, *J* = 7.1 Hz, 2H), 3.17 – 3.09 (m, 2H), 2.97 – 2.70 (m, 3H), , 2.12 (s, 3H), 1.33 (t, *J* = 7.1 Hz, 3H). **$^{13}$C NMR** (126 MHz, $CDCl_3$) δ = 173.7 ($C_q$), 163.8 (t, *J* = 33.2 Hz, $C_q$), 141.1 (CH), 140.3 ($C_q$), 138.6 ($C_q$), 135.8 ($C_q$), 135.3 ($C_q$), 132.7 (CH), 128.5 (CH), 128.2 (CH), 127.9 (CH), 127.0 (CH), 118.2 (CH), 118.0 (CH), 115.4 (t, *J* = 250.5 Hz, $C_q$), 107.7 (CH), 67.1 ($CH_2$), 63.0 ($CH_2$), 48.1 ($C_q$), 32.3 (t, *J* = 25.7 Hz, CH), 30.6 (t, *J* = 4.8 Hz, $CH_2$), 19.4 ($CH_3$), 14.1 ($CH_3$). **$^{19}$F NMR** (471 MHz, $CDCl_3$) δ = -114.03 (d, *J* = 15.8 Hz). **IR** (ATR): 2959, 1763, 1728, 1309, 1223, 1124, 1085, 1039, 740 $cm^{-1}$. **MS** (ESI) *m/z* (relative intensity): 469 $[M+H]^+$ (10), 491 $[M+Na]^+$ (100). **HR-MS** (ESI) *m/z* calcd for $C_{26}H_{26}N_2O_4F_2$ $[M+Na]^+$: 491.1753, found: 491.1770.

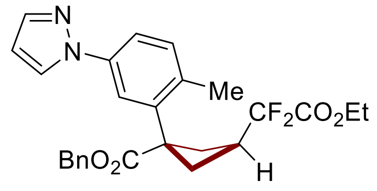


**Benzyl (1r,3r)-3-(2-ethoxy-1,1-difluoro-2-oxoethyl)-1-(2-methyl-5-(1H-pyrazol-1-yl)phenyl)cyclobutane-1-carboxylate** (*trans*-**18**, 19 mg, 13%) was obtained as a colorless oil. **$^{1}$H NMR** (500 MHz, $CDCl_3$) δ = 7.90 (dd, *J* = 2.5, 0.6 Hz, 1H), 7.72 (dd, *J* = 1.8, 0.6 Hz, 1H), 7.55 (d, *J* = 2.4 Hz, 1H), 7.45 (dd, *J* = 8.2, 2.3 Hz, 1H), 7.29 – 7.25 (m, 3H), 7.16 (dd, *J* = 8.1, 0.8 Hz, 1H), 7.14 – 7.11 (m, 2H), 6.46 (dd, *J* = 2.4, 1.8 Hz, 1H), 5.12 (s, 2H), 4.30 (q, *J* = 7.2 Hz, 2H), 3.26 – 3.11 (m, 1H), 3.00 – 2.92 (m, 2H), 2.78 – 2.71 (m, 2H), 2.12 (s, 3H), 1.33 (t, *J* = 7.1 Hz, 3H). **$^{13}$C NMR** (126 MHz, $CDCl_3$) δ = 174.4 ($C_q$), 163.8 (t, *J* = 33.4 Hz, $C_q$), 141.9 ($C_q$), 141.1 (CH), 138.6 ($C_q$), 1358 ($C_q$), 134.3 ($C_q$), 132.1 (CH), 128.6 (CH), 128.3 (CH), 127.7 (CH), 126.9 (CH), 118.4 (CH), 118.1 (CH), 114.8 (t, *J* = 249.9 Hz, $C_q$), 107.6 (CH), 67.1 ($CH_2$), 63.0 ($CH_2$), 48.8 ($C_q$), 33.6 (t, *J* = 25.9 Hz, CH), 31.8 (t, *J* = 5.1 Hz, $CH_2$), 19.4 ($CH_3$), 14.1 ($CH_3$). **$^{19}$F NMR** (471 MHz, $CDCl_3$) δ = -114.45 (d, *J* = 14.8 Hz). **IR** (ATR): 2955, 1762, 1725, 1310, 1203, 1118, 1039, 751 $cm^{-1}$. **MS** (ESI) *m/z* (relative intensity): 469 $[M+H]^+$ (20), 491

$[M+Na]^+$ (100). **HR-MS** (ESI) *m/z* calcd for $C_{26}H_{26}N_2O_4F_2$ $[M+Na]^+$: 491.1753, found: 491.1762.

Compound **19** was obtained following the general procedure with **1a** (56 mg, 0.3 mmol), 1-(4-methoxyphenyl)-1H-pyrazole (157 mg, 0.9 mmol), and ethyl 2-bromo-2,2-difluoroacetate (183 mg, 0.9 mmol) for 24 h. Purification by column chromatography on silica gel (*n*-hexane/EtOAc/DCM = 15/1/1→8/1/1) yielded **19** (96 mg, 66%) with two separable diastereomers (*cis*: *trans* = 2.2:1). $\mathbf{R_f}$= 0.48 for *cis*-**19** and 0.55 for *trans*-**19** (TLC: *n*-hexane/EtOAc/DCM = 3/1/1).

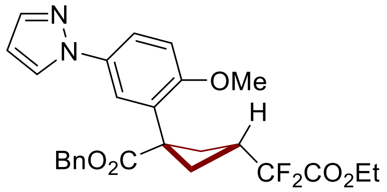


**Benzyl (1s,3s)-3-(2-ethoxy-1,1-difluoro-2-oxoethyl)-1-(2-methoxy-5-(1H-pyrazol-1-yl)phenyl)cyclobutane-1-carboxylate** (*cis*-**19**, 66 mg, 45%) was obtained as a colorless oil. **$^1$H NMR** (400 MHz, $CDCl_3$) δ = 7.85 (d, *J* = 2.4 Hz, 1H), 7.76 (d, *J* = 2.6 Hz, 1H), 7.71 (d, *J* = 1.8 Hz, 1H), 7.51 (dd, *J* = 8.7, 2.6 Hz, 1H), 7.31 – 7.22 (m, 3H), 7.21 – 7.15 (m, 2H), 6.85 (d, *J* = 8.7 Hz, 1H), 6.45 (t, *J* = 2.1 Hz, 1H), 5.09 (s, 2H), 4.28 (q, *J* = 7.1 Hz, 2H), 3.55 (s, 3H), 3.10 – 2.82 (m, 3H), 2.57 – 2.47 (m, 2H), 1.31 (t, *J* = 7.2 Hz, 3H). **$^{13}$C NMR** (101 MHz, $CDCl_3$) δ = 173.8 ($C_q$), 163.9 (t, *J* = 34.8 Hz, $C_q$), 156.2 ($C_q$), 140.9 (CH), 136.3 ($C_q$), 133.9 ($C_q$), 130.6 ($C_q$), 128.5 (CH), 128.1 (CH), 127.2 (CH), 119.8 (CH), 118.8 (CH), 115.3 (t, *J* = 250.6 Hz, $C_q$), 111.2 (CH), 107.5 (CH), 66.6 ($CH_2$), 63.0 ($CH_2$), 55.5 ($CH_3$), 45.3 ($C_q$), 32.8 (t, *J* = 25.9 Hz, CH), 29.7 (t, *J* = 5.0 Hz, $CH_2$), 14.1 ($CH_3$). **$^{19}$F NMR** (376 MHz, $CDCl_3$) δ = -113.79 (d, *J* = 14.7 Hz). **IR** (ATR): 2963, 1763, 1729, 1495, 1218, 1123, 1037, 727 $cm^{-1}$. **MS** (ESI) *m/z* (relative intensity): 485 $[M+H]^+$ (10), 507 $[M+Na]^+$ (100). **HR-MS** (ESI) *m/z* calcd for $C_{26}H_{26}N_2O_5F_2$ $[M+Na]^+$: 507.1702, found: 507.1702.

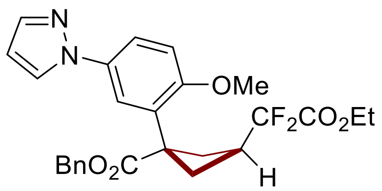


**Benzyl (1r,3r)-3-(2-ethoxy-1,1-difluoro-2-oxoethyl)-1-(2-methoxy-5-(1H-pyrazol-1-yl)phenyl)cyclobutane-1-carboxylate** (*trans*-**19**, 30 mg, 21%) was obtained as a colorless oil. **$^{1}$H NMR** (500 MHz, $CDCl_3$) δ = 7.85 (dd, $J$ = 2.4, 0.6 Hz, 1H), 7.71 (dd, $J$ = 1.8, 0.6 Hz, 1H), 7.51 (d, $J$ = 6.8 Hz, 2H), 7.31 – 7.23 (m, 3H), 7.18 – 7.14 (m, 2H), 6.84 (d, $J$ = 8.6 Hz, 1H), 6.45 (dd, $J$ = 2.4, 1.8 Hz, 1H), 5.12 (s, 2H), 4.29 (q, $J$ = 7.1 Hz, 2H), 3.62 (s, 3H), 3.37 – 3.23 (m, 1H), 2.89 – 2.81 (m, 2H), 2.68 – 2.61 (m, 2H), 1.32 (t, $J$ = 7.1 Hz, 3H). **$^{13}$C NMR** (126 MHz, $CDCl_3$) δ = 174.9 ($C_q$), 163.9 (t, $J$ = 33.3 Hz, $C_q$), 155.7 ($C_q$), 140.8 (CH), 136.2 ($C_q$), 133.9 ($C_q$), 132.2 ($C_q$), 128.5 (CH), 128.1 (CH), 127.8 (CH), 127.1 (CH), 119.5 (CH), 119.2 (CH), 115.1 (t, $J$ = 249.8 Hz, $C_q$), 110.9 (CH), 107.4 (CH), 66.7 ($CH_2$), 63.0 ($CH_2$), 55.5 ($CH_3$), 46.3 ($C_q$), 33.7 (t, $J$ = 25.7 Hz, CH), 30.9 (t, $J$ = 5.1 Hz, $CH_2$), 14.1 ($CH_3$). **$^{19}$F NMR** (471 MHz, $CDCl_3$) δ = -114.53 (d, $J$ = 15.4 Hz). **IR** (ATR): 2959, 1763, 1728, 1496, 1251, 1208, 1117, 1023, 750 $cm^{-1}$. **MS** (ESI) *m*/*z* (relative intensity): 485 $[M+H]^+$ (15), 507 $[M+Na]^+$ (100). **HR-MS** (ESI) *m*/*z* calcd for $C_{26}H_{26}N_2O_5F_2$ $[M+Na]^+$: 507.1702, found: 507.1713.

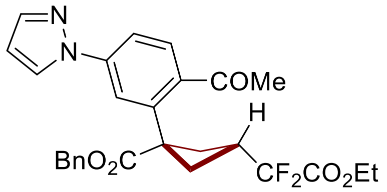


**Benzyl 1-(2-acetyl-5-(1H-pyrazol-1-yl)phenyl)-3-(2-ethoxy-1,1-difluoro-2-oxoethyl)cyclobutane-1-carboxylate** (**20**). The product was obtained following the general procedure with **1a** (56 mg, 0.3 mmol), 1-(4-(1H-pyrazol-1-yl)phenyl)ethan-1-one (167 mg, 0.9 mmol), and ethyl 2-bromo-2,2-difluoroacetate (183 mg, 0.9 mmol) for 24 h. Purification by column chromatography on silica gel (*n*-hexane/EtOAc = 5/1→ 3/1) yielded **20** (54 mg, 36%) as a colorless oil with two diastereomers (*cis*: *trans* =

13:1) determined by **$^{19}$F-NMR**. **R$_f$** = 0.22 (TLC: *n*-hexane/EtOAc = 3/1). **$^1$H NMR** (500 MHz, $CDCl_3$) δ = 8.06 (d, *J* = 2.2 Hz, 0.86H, *cis*), 8.01 (d, *J* = 2.6 Hz, 0.87H, *cis*), 7.99 – 7.93 (m, 1.41H, *cis+trans*), 7.77 – 7.76 (m, 1H, *cis+trans*), 7.70 – 7.67 (m, 1H, *cis+trans*), 7.31 – 7.22 (m, 5H, *cis+trans*), 6.52 (t, *J* = 2.2 Hz, 1H, *cis+trans*), 5.12 – 5.09 (m, 2H, *cis+trans*), 4.28 – 4.22 (m, 2H, *cis+trans*), 3.23 – 3.13 (m, 2H, *cis+trans*), 2.85 – 2.75 (m, 1H, *cis+trans*), 2.52 – 2.48 (m, 3H, *cis+trans*), 2.46 – 2.39 (m, 2H, *cis+trans*), 1.31 – 1.27 (m, 3H, *cis+trans*). **$^{13}$C NMR** (126 MHz, $CDCl_3$) (*cis*-isomer) δ = 199.2 ($C_q$), 173.3 ($C_q$), 163.9 (t, *J* = 33.2 Hz, $C_q$), 143.6 ($C_q$), 142.6 ($C_q$), 142.3 (CH), 136.2 ($C_q$), 133.5 ($C_q$), 133.2 (CH), 128.5 (CH), 128.0 (CH), 128.0 (CH), 127.1 (CH), 118.5 (CH), 116.7 (CH), 115.2 (t, *J* = 250.7 Hz, $C_q$), 108.9 (CH), 66.8 ($CH_2$), 63.0 ($CH_2$), 48.7 ($C_q$), 32.8 (t, *J* = 26.0 Hz, CH), 31.5 (t, *J* = 4.9 Hz, $CH_2$), 28.7 ($CH_3$), 14.1 ($CH_3$). **$^{19}$F NMR** (471 MHz, $CDCl_3$) δ = -113.45 (d, *J* = 14.6 Hz, *cis*), -114.12 (d, *J* = 15.6 Hz, *trans*). **IR** (ATR): 2966, 1726, 1606, 1394, 1223, 1125, 1041, 751, 698 $cm^{-1}$. **MS** (ESI) *m/z* (relative intensity): 497 $[M+H]^+$ (10), 519 $[M+Na]^+$ (100). **HR-MS** (ESI) *m/z* calcd for $C_{27}H_{26}N_2O_5F_2$ $[M+H]^+$: 497.1883, found: 497.1883.

Compound **21** was obtained following the general procedure with **1a** (56 mg, 0.3 mmol), 1-(4-bromophenyl)-1H-pyrazole (201 mg, 0.9 mmol), and ethyl 2-bromo-2,2-difluoroacetate (183 mg, 0.9 mmol) for 24 h. Purification by column chromatography on silica gel (*n*-hexane/EtOAc/DCM = 15/1/1→10/1/1) yielded **21** (77 mg, 48%) with two separable diastereomers (*cis*: *trans* = 2:1). **R$_f$** = 0.53 for *cis*-**21** and 0.62 for *trans*-**21** (TLC: *n*-hexane/EtOAc/DCM = 3/1/1).

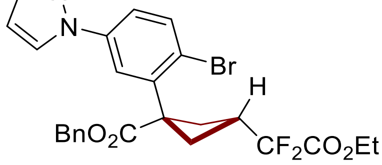


**Benzyl**

**(1s,3s)-1-(2-bromo-5-(1H-pyrazol-1-yl)phenyl)-3-(2-ethoxy-1,1-difluoro-2-oxoeth**

**yl)cyclobutane-1-carboxylate** (*cis*-**21**, 51 mg, 32%) was obtained as a colorless oil. **$^1$H NMR** (400 MHz, $CDCl_3$) δ = 7.95 (dd, *J* = 8.8, 2.6 Hz, 2H), 7.75 (d, *J* = 1.7 Hz, 1H), 7.65 (d, *J* = 8.5 Hz, 1H), 7.45 (dd, *J* = 8.5, 2.6 Hz, 1H), 7.33 – 7.25 (m, 3H), 7.25 – 7.20 (m, 2H), 6.50 (t, *J* = 2.2 Hz, 1H), 5.13 (s, 2H), 4.30 (q, *J* = 7.2 Hz, 2H), 3.21 – 3.12 (m, 2H), 2.99 – 2.82 (m, 1H), 2.74 – 2.66 (m, 2H), 1.33 (t, *J* = 7.2 Hz, 3H). **$^{13}$C NMR** (101 MHz, $CDCl_3$) δ = 172.8 ($C_q$), 163.7 ($C_q$), 141.7 (CH), 141.2 ($C_q$), 139.7 ($C_q$), 135.7 ($C_q$), 135.0 (CH), 128.6 (CH), 128.3 (CH), 128.1 (CH), 127.0 (CH), 121.7 ($C_q$), 119.2 (CH), 119.1 (CH), 115.1 ($C_q$), 108.4 (CH), 67.4 ($CH_2$), 63.1 ($CH_2$), 49.3 ($C_q$), 32.5 (t, *J* = 26.1 Hz, CH), 30.3 (d, *J* = 5.1 Hz, $CH_2$), 14.1 ($CH_3$). **$^{19}$F NMR** (376 MHz, $CDCl_3$) δ = -113.79 (d, *J* = 14.7 Hz). **IR** (ATR): 2963, 1763, 1729, 1473, 1309, 1221, 1127, 1045, 750, 695 $cm^{-1}$. **MS** (ESI) *m/z* (relative intensity): 533 $[M+H]^+$ (100) ($^{79}$Br), 535 $[M+H]^+$ (97) ($^{81}$Br). **HR-MS** (ESI) *m/z* calcd for $C_{25}H_{23}N_2O_4F_2Br$ $[M+H]^+$: 533.0882, found: 533.0894.

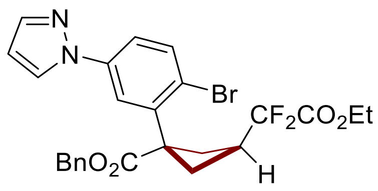


**Benzyl (1r,3r)-1-(2-bromo-5-(1H-pyrazol-1-yl)phenyl)-3-(2-ethoxy-1,1-difluoro-2-oxoethyl)cyclobutane-1-carboxylate** (*trans*-**21**, 26 mg, 16%) was obtained as a colorless oil. **$^1$H NMR** (400 MHz, $CDCl_3$) δ = 7.92 (dd, *J* = 2.5, 0.6 Hz, 1H), 7.74 (dd, *J* = 1.8, 0.6 Hz, 1H), 7.67 (d, *J* = 2.6 Hz, 1H), 7.58 (d, *J* = 8.6 Hz, 1H), 7.43 (dd, *J* = 8.5, 2.6 Hz, 1H), 7.31 – 7.26 (m, 3H), 7.22 – 7.17 (m, 2H), 6.49 (dd, *J* = 2.5, 1.8 Hz, 1H), 5.15 (s, 2H), 4.30 (q, *J* = 7.2 Hz, 2H), 3.41 – 3.26 (m, 1H), 3.06 – 2.96 (m, 2H), 2.78 – 2.69 (m, 2H), 1.33 (t, *J* = 7.1 Hz, 3H). **$^{13}$C NMR** (101 MHz, $CDCl_3$) δ = 173.8 ($C_q$), 163.7 (t, *J* = 33.3 Hz, $C_q$), 143.2 ($C_q$), 141.6 (CH), 139.6 ($C_q$), 135.7 ($C_q$), 134.4 (CH), 128.6 (CH), 128.3 (CH), 127.9 (CH), 127.0 (CH), 119.9 ($C_q$), 119.6 (CH), 119.1 (CH), 114.9 (t, *J* = 250.1 Hz, $C_q$), 108.3 (CH), 67.4 ($CH_2$), 63.1 ($CH_2$), 49.9 ($C_q$), 33.0 (t, *J* = 25.8 Hz, CH), 31.6 (t, *J* = 5.1 Hz, $CH_2$), 14.1 ($CH_3$). **$^{19}$F NMR** (377 MHz, $CDCl_3$) δ =

-114.61 (d, $J$ = 15.2 Hz). **IR** (ATR): 2986, 1763, 1727, 1475, 1305, 1208, 1120, 1040, 751, 698 $cm^{-1}$. **MS** (ESI) $m/z$ (relative intensity): 533 $[M+H]^+$ (100) ($^{79}Br$), 535 $[M+H]^+$ (97) ($^{81}Br$). **HR-MS** (ESI) $m/z$ calcd for $C_{25}H_{23}N_2O_4F_2Br$ $[M+H]^+$: 533.0882, found: 533.0877.

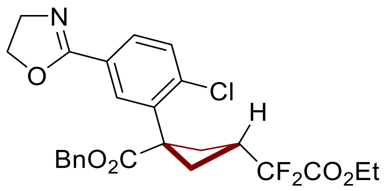


**Benzyl 1-(2-chloro-5-(4,5-dihydrooxazol-2-yl)phenyl)-3-(2-ethoxy-1,1-difluoro-2-oxoethyl)cyclobutane-1-carboxylate** (**22**). The product was obtained following the general procedure with **1a** (56 mg, 0.3 mmol), 2-(4-chlorophenyl)-4,5-dihydrooxazole (163 mg, 0.9 mmol), and ethyl 2-bromo-2,2-difluoroacetate (183 mg, 0.9 mmol) for 24 h. Purification by column chromatography on silica gel ($n$-hexane/EtOAc = 5/1→3/1) yielded **22** (69 mg, 47%) as a colorless oil with two diastereomers (*cis*: *trans* = 6.3:1). **$R_f$** = 0.20 (TLC: $n$-hexane/EtOAc = 2/1). **$^1$H NMR** (400 MHz, $CDCl_3$) δ = 8.12 (d, $J$ = 2.0 Hz, 0.85H, *cis*), 7.87 (d, $J$ = 2.0 Hz, 0.12H, *trans*), 7.85 – 7.78 (m, 1H, *cis*+*trans*), 7.45 – 7.35 (m, 1H, *cis*+*trans*), 7.32 – 7.24 (m, 3H, *cis*+*trans*), 7.21 – 7.12 (m, 2H, *cis*+*trans*), 5.12 (s, 0.26H, *trans*), 5.10 (s, 1.63H, *cis*), 4.49 – 4.41 (m, 2H, *cis*+*trans*), 4.29 (q, $J$ = 7.1 Hz, 2H, *cis*+*trans*), 4.11 – 4.03 (m, 2H, *cis*+*trans*), 3.42 – 2.60 (m, 5H, *cis*+*trans*), 1.35 – 1.30 (m, 3H, *cis*+*trans*). **$^{13}$C NMR** (101 MHz, $CDCl_3$) δ = 173.9 ($C_q$, *trans*), 172.9 ($C_q$, *cis*), 163.8 ($C_q$, *cis*), 163.8 ($C_q$, *trans*), 163.8 (t, $J$ = 33.3 Hz, $C_q$, *cis*+*trans*), 140.3 ($C_q$, *trans*), 138.5 ($C_q$, *cis*), 137.9 ($C_q$, *cis*+*trans*), 135.7 ($C_q$, *cis*), 136.5 ($C_q$, *trans*), 130.8 (CH, *cis*), 130.2 (CH, *trans*), 128.6 (CH, *cis*), 128.6 (CH, *trans*), 128.5 (CH, *cis*), 128.3 (CH, *trans*), 128.2 (CH, *trans*), 128.2 (CH, *cis*), 128.1 (CH, *trans*), 127.9 (CH, *cis*), 127.7 (CH, *trans*), 127.2 (CH, *cis*), 126.7 ($C_q$, *cis*), 126.6 ($C_q$, *trans*), 115.1 (t, $J$ = 250.5 Hz, $C_q$, *cis*), 114.9 (t, $J$ = 250.5 Hz, $C_q$, *trans*), 68.0 ($CH_2$, *cis*), 67.9 ($CH_2$, *trans*), 67.3 ($CH_2$, cis+trans), 63.1 ($CH_2$, cis), 63.0 ($CH_2$, *trans*), 55.1 ($CH_2$, *cis*), 55.1 ($CH_2$, *trans*), 48.3 ($C_q$, *trans*), 47.6 ($C_q$, cis), 33.3 (t, $J$ = 25.8 Hz,

CH, *trans*), 32.5 (t, $J$ = 26.0 Hz, CH, *cis*), 31.3 (t, $J$ = 5.2 Hz, $CH_2$, *trans*), 29.9 (t, $J$ = 5.1 Hz, $CH_2$, *cis*), 14.1 ($CH_3$, *cis*+*trans*). **$^{19}$F NMR** (377 MHz, $CDCl_3$) δ = -113.82 (d, $J$ = 14.7 Hz, *cis*), -114.67 (d, $J$ = 15.3 Hz, *trans*). **IR** (ATR): 2961, 1731, 1651, 1307, 1267, 1081, 949, 734 $cm^{-1}$. **MS** (ESI) *m/z* (relative intensity): 492 $[M+H]^+$ (20), 514 $[M+Na]^+$ (100). **HR-MS** (ESI) *m/z* calcd for $C_{25}H_{24}NO_5F_2Cl$ $[M+H]^+$: 492.1384, found: 492.1392.

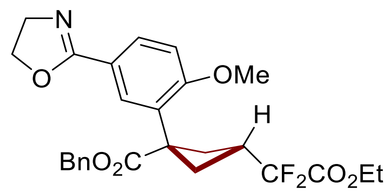


**Benzyl 1-(5-(4,5-dihydrooxazol-2-yl)-2-methoxyphenyl)-3-(2-ethoxy-1,1-difluoro-2-oxoethyl)cyclobutane-1-carboxylate** (**23**). The product was obtained following the general procedure with **1a** (56 mg, 0.3 mmol), 2-(4-methoxyphenyl)-4,5-dihydrooxazole (159 mg, 0.9 mmol), and ethyl 2-bromo-2,2-difluoroacetate (183 mg, 0.9 mmol) for 24 h. Purification by column chromatography on silica gel (*n*-hexane/EtOAc = 5/1→3/1) yielded **23** (63 mg, 43%) as a colorless oil with two diastereomers (*cis*: *trans* = 2.5:1). **R$_f$** = 0.15 (TLC: *n*-hexane/EtOAc = 1/1). **$^1$H NMR** (400 MHz, $CDCl_3$) δ = 8.03 (d, $J$ = 2.1 Hz, 0.72H, *cis*), 7.91 – 7.83 (m, 1H, *cis*+*trans*), 7.80 (d, $J$ = 2.1 Hz, 0.27H, *trans*), 7.34 – 7.22 (m, 3H, *cis*+*trans*), 7.18 – 7.10 (m, 2H, *cis*+*trans*), 6.83 – 6.77 (m, 1H, *cis*+*trans*), 5.08 (s, 0.55H, *trans*), 5.06 (s, 1.40H, *cis*), 4.46 – 4.38 (m, 2H, *cis*+*trans*), 4.28 (q, $J$ = 7.2 Hz, 2H, *cis*+*trans*), 4.10 – 4.01 (m, 2H, *cis*+*trans*), 3.61 (s, 0.80H, *trans*), 3.54 (s, 2.07H, *cis*), 3.36 – 2.78 (m, 3H, *cis*+*trans*), 2.67 – 2.47 (m, 2H, *cis*+*trans*), 1.34 – 1.29 (m, 3H, *cis*+*trans*). **$^{13}$C NMR** (101 MHz, $CDCl_3$) δ = 175.0 ($C_q$, *trans*), 173.9 ($C_q$, *cis*), 164.5 ($C_q$, *cis*), 164.5 ($C_q$, *trans)*, 163.9 (t, $J$ = 33.3 Hz, $C_q$, *cis*+*trans*), 159.9 ($C_q$, *cis*), 159.5 ($C_q$, *trans*), 136.3 ($C_q$, *cis*), 136.3 ($C_q$, *trans*), 131.2 ($C_q$, *trans*), 129.4 ($C_q$, *cis*), 129.3 (CH, *cis*), 129.0 (CH, *trans*), 128.5 (CH, *trans*), 128.4 (CH, *cis*), 128.0 (CH, *cis*+*trans*), 127.7 (CH, *cis*), 127.2 (CH, *trans*), 126.3 (CH, *cis*+*trans*), 120.1 ($C_q$, *trans*), 120.0 ($C_q$, *cis*), 115.3 (t, $J$ = 251.5 Hz, $C_q$, *cis*), 115.1 (t,

$J$ = 33.3 Hz, $C_q$, *trans*), 110.3 (CH, *cis*), 110.0 (CH, *trans*), 67.7 ($CH_2$, *cis*), 67.7 ($CH_2$, *trans*), 66.6 ($CH_2$, *trans*), 66.5 ($CH_2$, *cis*), 62.9 ($CH_2$, *cis*), 62.9 ($CH_2$, *trans*), 55.3 ($CH_3$, *cis*), 55.3 ($CH_3$, *trans*), 55.0 ($CH_2$, *cis*), 55.0 ($CH_2$, *trans*), 46.2 ($C_q$, *trans*), 45.1 ($C_q$, *cis*), 33.7 (t, $J$ = 25.7 Hz, CH, *trans*), 32.8 (t, $J$ = 25.9 Hz, CH, *cis*), 30.8 (t, $J$ = 5.2 Hz, $CH_2$, *trans*), 29.5 (t, $J$ = 5.0 Hz, $CH_2$, *cis*), 14.1 ($CH_3$, *cis+trans*). **$^{19}$F NMR** (377 MHz, $CDCl_3$) δ = -113.79 (d, $J$ = 14.8 Hz, *cis*), -114.54 (d, $J$ = 15.5 Hz, *trans*). **IR** (ATR): 2962, 1729, 1649, 1258, 1123, 1026, 948, 740 $cm^{-1}$. **MS** (ESI) *m/z* (relative intensity): 488 $[M+H]^+$ (20), 510 $[M+Na]^+$ (100). **HR-MS** (ESI) *m/z* calcd for $C_{26}H_{27}NO_6F_2$ $[M+H]^+$: 488.1879, found: 488.1892.

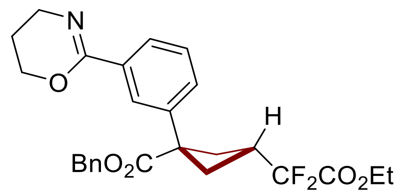


**Benzyl 1-(3-(5,6-dihydro-4H-1,3-oxazin-2-yl)phenyl)-3-(2-ethoxy-1,1-difluoro-2-oxoethyl)cyclobutane-1-carboxylate** (**24**). The product was obtained following the general procedure with **1a** (56 mg, 0.3 mmol), 2-phenyl-5,6-dihydro-4H-1,3-oxazine (145 mg, 0.9 mmol), and ethyl 2-bromo-2,2-difluoroacetate (183 mg, 0.9 mmol) for 24 h. Purification by column chromatography on silica gel (*n*-hexane/EtOAc = 5/1→2/1) yielded **24** (60 mg, 43%) as a colorless oil with two diastereomers (*cis*: *trans* = 3.3:1). **$R_f$**= 0.30 (TLC: *n*-hexane/EtOAc = 2/1). **$^1$H NMR** (400 MHz, $CDCl_3$) δ = 7.97 (t, $J$ = 1.9 Hz, 0.77H, *cis*), 7.85 – 7.75 (m, 1.31H, *cis+trans*), 7.45 (ddd, $J$ = 7.8, 2.1, 1.2 Hz, 0.80H, *cis*), 7.38 – 7.26 (m, 4.24H, *cis+trans*), 7.19 – 7.13 (m, 2H, *cis+trans*), 5.09 (s, 0.46H, *trans*), 5.06 (s, 1.50H, *cis*), 4.36 (t, $J$ = 5.4 Hz, 2H, *cis+trans*), 4.31 – 4.24 (m, 2H, *cis+trans*), 3.61 (t, $J$ = 5.8 Hz, 2H, *cis+trans*), 3.17 – 2.65 (m, 5H, *cis+trans*), 2.02 – 1.94 (m, 2H, *cis+trans*), 1.34 – 1.29 (m, 3H, *cis+trans*). **$^{13}$C NMR** (101 MHz, $CDCl_3$) δ = 174.6 ($C_q$, *trans*), 174.2 ($C_q$, *cis*), 163.8 (t, $J$ = 33.3 Hz, $C_q$, *cis*), 163.8 (t, $J$ = 33.3 Hz, $C_q$, *trans*), 155.4 ($C_q$, *cis+trans*), 142.6 ($C_q$, *trans*), 140.8 ($C_q$, *cis*), 135.9 ($C_q$, *cis*), 135.9 ($C_q$, *trans*), 134.6 ($C_q$, *cis*), 134.4 ($C_q$, *trans*), 128.8 (CH, *cis*), 128.6

(CH, *trans*), 128.5 (CH, *cis*), 128.4 (CH, *cis*), 128.2 (CH, *trans*), 128.2 (CH, *trans*), 128.1 (CH, *cis*), 128.1 (CH, *trans*), 127.7 (CH, *cis*), 127.6 (CH, *trans*), 126.1 (CH, *cis*), 125.8 (CH, *trans*), 125.2 (CH, *cis*), 124.6 (CH, *trans*), 115.4 (t, *J* = 251.5 Hz, $C_q$, *cis*), 114.9 (t, *J* = 251.5 Hz, $C_q$, *trans*), 67.1 ($CH_2$, *trans*), 66.9 ($CH_2$, *cis*), 65.4 ($CH_2$, *cis*), 65.3 ($CH_2$, *trans*), 63.0 ($CH_2$, *cis+trans*), 48.4 ($C_q$, *trans*), 47.7 ($C_q$, *cis*), 42.8 ($CH_2$, *cis+trans*), 33.5 (t, *J* = 26.0 Hz, CH, *trans*), 32.1 (t, *J* = 25.8 Hz, CH, *cis*), 31.6 (t, *J* = 5.0 Hz, $CH_2$, *trans*), 31.1 (t, *J* = 4.9 Hz, $CH_2$, *cis*), 22.0 ($CH_2$, *cis+trans*), 14.1 ($CH_3$, *cis+trans*). **$^{19}$F NMR** (377 MHz, $CDCl_3$) δ = -114.27 (d, *J* = 15.2 Hz, *cis*), -114.37 (d, *J* = 14.9 Hz, *trans*). **IR** (ATR): 2960, 1764, 1730, 1656, 1224, 1131, 1038, 693 $cm^{-1}$. **MS** (ESI) *m*/*z* (relative intensity): 472 $[M+H]^+$ (20), 494 $[M+Na]^+$ (100). **HR-MS** (ESI) *m*/*z* calcd for $C_{26}H_{27}NO_5F_2$ $[M+H]^+$: 472.1930, found: 472.1943.

Compound **25** was obtained following the general procedure with **1a** (56 mg, 0.3 mmol), benzo[h]quinoline (161 mg, 0.9 mmol), and ethyl 2-bromo-2,2-difluoroacetate (183 mg, 0.9 mmol) for 24 h. Purification by column chromatography on silica gel (*n*-hexane/EtOAc/DCM = 20/1/1 → 10/1/1) yielded **25** (94 mg, 64%) with two separable diastereomers (*cis*: *trans* = 2.9:1). **$R_f$** = 0.45 for *cis*-**25** and 0.50 for *trans*-**25** (TLC: *n*-hexane/EtOAc/DCM = 5/1/1).

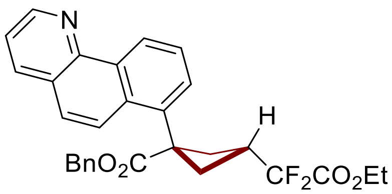


**Benzyl (1s,3s)-1-(benzo[h]quinolin-7-yl)-3-(2-ethoxy-1,1-difluoro-2-oxoethyl)cyclobutane-1-carboxylate** (*cis*-**25**, 70 mg, 48%) was obtained as a colorless oil. **$^1$H NMR** (500 MHz, $CDCl_3$) δ = 9.38 (dt, *J* = 8.2, 1.0 Hz, 1H), 9.02 (dd, *J* = 4.3, 1.8 Hz, 1H), 8.16 (dd, *J* = 8.1, 1.8 Hz, 1H), 7.85 (dd, *J* = 7.4, 1.3 Hz, 1H), 7.80 – 7.75 (m, 2H), 7.61 (d, *J* = 9.2 Hz, 1H), 7.54 (dd, *J* = 8.0, 4.4 Hz, 1H), 7.20 – 7.12 (m, 3H), 7.04 – 6.98 (m, 2H), 5.04 (s, 2H), 4.31 (q, *J* = 7.1 Hz, 2H), 3.33 (d, *J* = 5.8 Hz, 2H), 3.00 – 2.87 (m,

3H), 1.33 (t, $J$ = 7.1 Hz, 3H). **$^{13}$C NMR** (126 MHz, $CDCl_3$) δ = 174.5 ($C_q$), 163.8 (t, $J$ = 33.2 Hz, $C_q$), 149.1 (CH), 146.9 ($C_q$), 136.7 ($C_q$), 135.8 (CH), 135.7 ($C_q$), 132.9 ($C_q$), 131.4 ($C_q$), 128.4 (CH), 128.1 (CH), 127.7 (CH), 126.7 (CH), 126.5 (CH), 125.9 ($C_q$), 125.7 (CH), 124.7 (CH), 124.1 (CH), 122.2 (CH), 115.4 (t, $J$ = 250.5 Hz, $C_q$), 67.1 ($CH_2$), 63.1 ($CH_2$), 48.1 ($C_q$), 32.8 (t, $J$ = 25.7 Hz, CH), 31.4 (t, $J$ = 4.9 Hz, $CH_2$), 14.1 ($CH_3$). **$^{19}$F NMR** (471 MHz, $CDCl_3$) δ = -114.01 (d, $J$ = 13.3 Hz). **IR** (ATR): 2959, 1762, 1726, 1423, 1226, 1127, 1047, 835, 765, 695 $cm^{-1}$. **MS** (ESI) $m/z$ (relative intensity): 490 $[M+H]^+$ (70), 512 $[M+Na]^+$ (100). **HR-MS** (ESI) $m/z$ calcd for $C_{29}H_{25}NO_4F_2$ $[M+H]^+$: 490.1824, found: 490.1827.

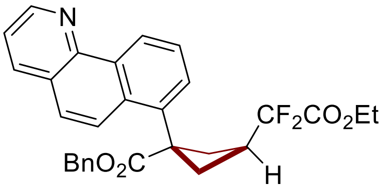


**Benzyl (1r,3r)-1-(benzo[h]quinolin-7-yl)-3-(2-ethoxy-1,1-difluoro-2-oxoethyl)cyclobutane -1-carboxylate** (*trans*-**25**, 24 mg, 16%) was obtained as a colorless oil. **$^1$H NMR** (500 MHz, $CDCl_3$) δ = 9.33 (dt, $J$ = 8.2, 1.0 Hz, 1H), 9.01 (dd, $J$ = 4.4, 1.8 Hz, 1H), 8.16 (dd, $J$ = 8.1, 1.8 Hz, 1H), 7.75 (dd, $J$ = 8.3, 7.3 Hz, 1H), 7.70 (dd, $J$ = 9.2, 0.8 Hz, 1H), 7.65 (dd, $J$ = 7.4, 1.3 Hz, 1H), 7.61 (d, $J$ = 9.2 Hz, 1H), 7.54 (dd, $J$ = 8.0, 4.3 Hz, 1H), 7.19 – 7.09 (m, 3H), 7.02 – 6.94 (m, 2H), 5.06 (s, 2H), 4.30 (q, $J$ = 7.1 Hz, 2H), 3.36 – 3.16 (m, 3H), 2.92 (t, $J$ = 10.0 Hz, 2H), 1.32 (t, $J$ = 7.1 Hz, 3H). **$^{13}$C NMR** (126 MHz, $CDCl_3$) δ = 175.2 ($C_q$), 163.8 (t, $J$ = 33.3 Hz, $C_q$), 149.1 (CH), 146.9 ($C_q$), 138.7 ($C_q$), 135.8 (CH), 135.7 ($C_q$), 132.4 ($C_q$), 131.0 ($C_q$), 128.5 (CH), 128.1 (CH), 127.6 (CH), 127.2 (CH), 126.7 (CH), 125.9 ($C_q$), 125.7 (CH), 124.3 (CH), 123.9 (CH), 122.1 (CH), 114.9 (t, $J$ = 250.2 Hz, $C_q$), 67.2 ($CH_2$), 63.0 ($CH_2$), 48.9 ($C_q$), 33.7 (t, $J$ = 25.9 Hz, CH), 32.6 ($CH_2$), 14.1 ($CH_3$). **$^{19}$F NMR** (471 MHz, $CDCl_3$) δ = -114.41 (d, $J$ = 14.7 Hz). **IR** (ATR): 2956, 1762, 1724, 1422, 1193, 1123, 1052, 833, 776, 696 $cm^{-1}$. **MS** (ESI) $m/z$ (relative intensity): 490 $[M+H]^+$ (100), 512 $[M+Na]^+$ (60). **HR-MS** (ESI) $m/z$ calcd for $C_{29}H_{25}NO_4F_2$ $[M+H]^+$: 490.1824, found: 490.1825.

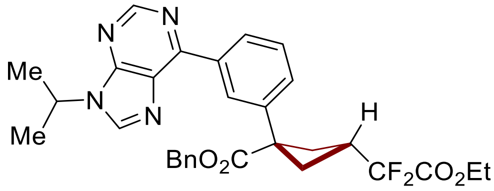


**Benzyl 3-(2-ethoxy-1,1-difluoro-2-oxoethyl)-1-(3-(9-isopropyl-9H-purin-6-yl)phenyl)cyclobutane-1-carboxylate** (**26**). The product was obtained following the general procedure with **1a** (56 mg, 0.3 mmol), 9-isopropyl-6-phenyl-9H-purine (214 mg, 0.9 mmol), and ethyl 2-bromo-2,2-difluoroacetate (183 mg, 0.9 mmol) for 24 h. Purification by column chromatography on silica gel (*n*-hexane/EtOAc = 5/1→3/1) yielded **26** (80 mg, 49%) as a colorless oil with two diastereomers (*cis*: *trans* = 6.1:1). **$R_f$** = 0.29 (TLC: *n*-hexane/EtOAc = 2/1). **$^1$H NMR** (400 MHz, $CDCl_3$) δ = 9.03 – 9.01 (m, 1H, *cis*+*trans*), 8.91 – 8.89 (m, 0.84H, *cis*), 8.80 – 8.75 (m, 1H, *cis*+*trans*), 8.67 (t, *J* = 1.8 Hz, 0.13H, *trans*), 8.18 (s, 1H, *cis*+*trans*), 7.58 – 7.51 (m, 2H, *cis*+*trans*), 7.25 – 7.16 (m, 5H, *cis*+*trans*), 5.12 (s, 0.28H, *trans*), 5.09 (s, 1.71H, *cis*), 5.03 – 4.95 (m, 1H, *cis*+*trans*), 4.33 – 4.25 (m, 2H, *cis*+*trans*), 3.17 – 2.76 (m, 5H, *cis*+*trans*), 1.68 (dd, *J* = 6.7, 1.1 Hz, 6H, *cis*+*trans*), 1.35 – 1.29 (m, 3H, *cis*+*trans*). **$^{13}$C NMR** (101 MHz, $CDCl_3$) (*cis*-isomer) δ = 174.3 ($C_q$), 163.8 (t, *J* = 33.3 Hz, $C_q$), 154.4 ($C_q$), 152.3 ($C_q$), 152.1 (CH), 142.2 (CH), 141.5 ($C_q$), 136.3 ($C_q$), 135.9 ($C_q$), 131.7 ($C_q$), 129.3 (CH), 129.2 (CH), 129.0 (CH), 128.5 (CH), 128.1 (CH), 127.9 (CH), 127.8 (CH), 115.5 (t, *J* = 250.1 Hz, $C_q$), 66.9 ($CH_2$), 63.0 ($CH_2$), 47.9 ($C_q$), 47.4 (CH), 32.1 (t, *J* = 25.7 Hz, CH), 31.2 (t, *J* = 4.9 Hz, $CH_2$), 22.7 ($CH_3$), 14.1 ($CH_3$). **$^{19}$F NMR** (377 MHz, $CDCl_3$) δ = -114.16 – -114.27 (m, *cis*+*trans*). **IR** (ATR): 2982, 1763, 1729, 1450, 1319, 1222, 1125, 700 $cm^{-1}$. **MS** (ESI) *m*/*z* (relative intensity): 549 $[M+H]^+$ (20), 571 $[M+Na]^+$ (100). **HR-MS** (ESI) *m*/*z* calcd for $C_{30}H_{30}N_4O_4F_2$ $[M+H]^+$: 549.2308, found: 549.2310.

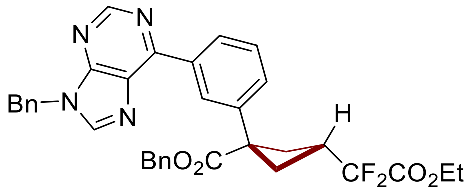

**Benzyl 1-(3-(9-benzyl-9H-purin-6-yl)phenyl)-3-(2-ethoxy-1,1-difluoro-2-oxoethyl)cyclobutane-1-carboxylate** (**27**). The product was obtained following the general procedure with **1a** (56 mg, 0.3 mmol), 9-benzyl-6-phenyl-9H-purine (257 mg, 0.9 mmol), and ethyl 2-bromo-2,2-difluoroacetate (183 mg, 0.9 mmol) for 24 h. Purification by column chromatography on silica gel (*n*-hexane/EtOAc = 5/1→3/1) yielded **27** (110 mg, 62%) as a colorless oil with two diastereomers (*cis*: *trans* = 9.3:1). $\mathbf{R_f}$ = 0.31 (TLC: *n*-hexane/EtOAc = 2/1). **$^1$H NMR** (500 MHz, $CDCl_3$) δ = 9.06 – 9.05 (m, 1H, *cis*+*trans*), 8.93 – 8.92 (m, 0.87H, *cis*), 8.80 – 8.76 (m, 1H, *cis*+*trans*), 8.69 (t, *J* = 1.8 Hz, 0.11H, *trans*), 8.09 (s, 1H, *cis*+*trans*), 7.58 – 7.51 (m, 2H, *cis*+*trans*), 7.40 – 7.35 (m, 3H, *cis*+*trans*), 7.34 – 7.32 (m, 2H, *cis*+*trans*), 7.24 – 7.17 (m, 5H, *cis*+*trans*), 5.49 (s, 2H, *cis*+*trans*), 5.12 (s, 0.19H, *trans*), 5.08 (s, 1.77H, *cis*), 4.32 – 4.27 (m, 2H, *cis*+*trans*), 3.17 – 2.79 (m, 5H, *cis*+*trans*), 1.35 – 1.30 (m, 3H, *cis*+*trans*). **$^{13}$C NMR** (126 MHz, $CDCl_3$) (*cis*-isomer) δ = 174.3 ($C_q$), 163.8 (t, *J* = 33.3 Hz, $C_q$), 154.5 ($C_q$), 152.7 ($C_q$), 152.7 (CH), 144.4 (CH), 141.6 ($C_q$), 136.2 ($C_q$), 135.9 ($C_q$), 135.3 ($C_q$), 131.2 ($C_q$), 129.4 (CH), 129.3 (CH), 129.2 (CH), 129.0 (CH), 128.7 (CH), 128.5 (CH), 128.1 (CH), 128.0 (CH), 128.0 (CH), 127.8 (CH), 115.5 (t, *J* = 250.1 Hz, $C_q$), 66.9 ($CH_2$), 63.0 ($CH_2$), 47.9 ($C_q$), 47.4 ($CH_2$), 32.1 (t, *J* = 25.7 Hz, CH), 31.2 (t, *J* = 4.9 Hz, $CH_2$), 14.1 ($CH_3$). **$^{19}$F NMR** (471 MHz, $CDCl_3$) δ = -114.17 – -114.28 (m, *cis*+*trans*). **IR** (ATR): 2959, 1762, 1727, 1450, 1317, 1220, 1168, 727 $cm^{-1}$. **MS** (ESI) *m*/*z* (relative intensity): 597 $[M+H]^+$ (20), 619 $[M+Na]^+$ (100). **HR-MS** (ESI) *m*/*z* calcd for $C_{34}H_{30}N_4O_4F_2$ $[M+H]^+$: 597.2308, found: 597.2318.

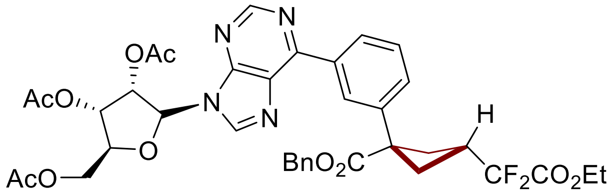

**(2S,3S,4S,5R)-2-(acetoxymethyl)-5-(6-(3-(1-((benzyloxy)carbonyl)-3-(2-ethoxy-1,1-difluoro-2-oxoethyl)cyclobutyl)phenyl)-9H-purin-9-yl)tetrahydrofuran-3,4-diyl diacetate** (**28**). The product was obtained following the general procedure with [Ru($O_2$CMes)$_2$(*p*-cymene)] (11.2 mg, 10.0 mol%), P(4-$CF_3C_6H_4$)$_3$ (9.3 mg, 10.0 mol%), $Na_2CO_3$ (42 mg, 0.4 mmol), **1a** (38 mg, 0.2 mmol), (2S,3S,4S,5R)-2-(acetoxymethyl)-5-(6-phenyl-9H-purin-9-yl)tetrahydrofuran-3,4-diyl diacetate (272 mg, 0.6 mmol), ethyl 2-bromo-2,2-difluoroacetate (122 mg, 0.6 mmol,) and 1,4-dioxane (1.3 mL) for 24 h. Purification by column chromatography on silica gel (*n*-hexane/EtOAc = 1/1→1/2) yielded **28** (69 mg, 45%) as a colorless oil with two diastereomers (*cis*: *trans* = 4.8:1). **R**$_f$ = 0.25 (TLC: *n*-hexane/EtOAc = 1/1). **$^1$H NMR** (500 MHz, $CDCl_3$) δ = 9.04 – 9.03 (m, 1H, *cis*+*trans*), 8.89 (t, *J* = 2.1 Hz, 0.77H, *cis*), 8.77 – 8.74 (m, 1H, *cis*+*trans*), 8.65 (t, *J* = 1.8 Hz, 0.18H, *trans*), 8.27 – 8.26 (m, 1H, *cis*+*trans*), 7.59 – 7.51 (m, 2H, *cis*+*trans*), 7.25 – 7.21 (m, 3H, *cis*+*trans*), 7.20 – 7.15 (m, 2H, *cis*+*trans*), 6.32 – 6.29 (m, 1H, *cis*+*trans*), 6.02 (t, *J* = 5.4 Hz, 1H, *cis*+*trans*), 5.73 – 5.69 (m, 1H, *cis*+*trans*), 5.12 (s, 0.33H, *trans*), 5.08 (s, 1.57H, *cis*), 4.51 – 4.45 (m, 2H, *cis*+*trans*), 4.44 – 4.38 (m, 1H, *cis*+*trans*), 4.31 – 4.26 (m, 2H, *cis*+*trans*), 3.16 – 2.76 (m, 5H, *cis*+*trans*), 2.17 (s, 3H, *cis*+*trans*), 2.14 (s, 3H, *cis*+*trans*), 2.10 – 2.08 (m, 3H, *cis*+*trans*), 1.35 – 1.31 (m, 3H, *cis*+*trans*). **$^{13}$C NMR** (126 MHz, $CDCl_3$) (*cis*-isomer) δ = 174.2 ($C_q$), 170.5 ($C_q$), 169.7 ($C_q$), 169.5 ($C_q$), 163.8 (t, *J* = 33.3 Hz, $C_q$), 155.0 ($C_q$), 152.8 (CH), 152.2 ($C_q$), 143.3 ($C_q$), 142.8 (CH), 141.7 ($C_q$), 135.9 ($C_q$), 131.9 ($C_q$), 129.6 (CH), 129.2 (CH), 129.1 (CH), 128.5 (CH), 128.1 (CH), 128.1 (CH), 127.8 (CH), 115.5 (t, *J* = 250.2 Hz, $C_q$), 86.5 (CH), 80.5 (CH), 73.2 (CH), 70.8 (CH), 67.0 ($CH_2$), 63.2 ($CH_2$), 63.0 ($CH_2$), 47.9 ($C_q$), 32.1 (t, *J* = 25.7 Hz, CH), 31.2 (t, *J* = 4.9 Hz, $CH_2$), 20.9 ($CH_3$), 20.7 ($CH_3$), 20.5 ($CH_3$), 14.1 ($CH_3$). **$^{19}$F NMR** (471 MHz, $CDCl_3$) δ = -114.18 – -114.29 (m, *cis*+*trans*). **IR** (ATR): 2958, 1751, 1574, 1372, 1212, 1040, 909, 732 $cm^{-1}$. **HR-MS** (ESI) *m*/*z* calcd for $C_{38}H_{38}N_4O_{11}F_2$ $[M+Na]^+$: 787.2397, found: 787.2396.

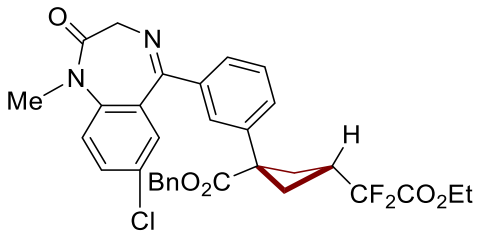

**Benzyl 1-(3-(7-chloro-1-methyl-2-oxo-2,3-dihydro-1H-benzo[e][1,4]diazepin-5-yl)phenyl)-3-(2-ethoxy-1,1-difluoro-2-oxoethyl)cyclobutane-1-carboxylate** (**29**). The product was obtained following the general procedure with **1a** (56 mg, 0.3 mmol), 7-chloro-1-methyl-5-phenyl-1,3-dihydro-2H-benzo[e][1,4]diazepin-2-one (256 mg, 0.9 mmol), and ethyl 2-bromo-2,2-difluoroacetate (183 mg, 0.9 mmol) for 24 h. Purification by column chromatography on silica gel (*n*-hexane/EtOAc = 2/1→1/1) yielded **29** (110 mg, 56%) as a colorless oil with two diastereomers (*cis*: *trans* = 1.7:1). $\mathbf{R_f}$ = 0.30 (TLC: *n*-hexane/EtOAc = 1/1). **$^1$H NMR** (400 MHz, $CDCl_3$) δ = 7.75 (s, 0.60H, *cis*), 7.57 (s, 0.35H, *trans*), 7.55 – 7.49 (m, 2H, *cis+trans*), 7.44 – 7.34 (m, 2H, *cis+trans*), 7.33 – 7.27 (m, 4H, *cis+trans*), 7.24 – 7.22 (m, 1H, *cis+trans*), 7.19 – 7.14 (m, 2H, *cis+trans*), 5.13 – 5.04 (m, 2H, *cis+trans*), 4.87 – 4.82 (m, 1H, *cis+trans*), 4.32 – 4.25 (m, 2H, *cis+trans*), 3.76 (d, *J* = 10.7 Hz, 1H, *cis+trans*), 3.41 – 3.38 (m, 3H, *cis+trans*), 3.11 – 2.64 (m, 5H, *cis+trans*), 1.35 – 1.29 (m, 3H, *cis+trans*). **$^{13}$C NMR** (101 MHz, $CDCl_3$) δ = 174.4 ($C_q$, *trans*), 174.0 ($C_q$, *cis*), 170.0 ($C_q$, *trans*), 169.9 ($C_q$, *cis*), 168.6 ($C_q$, *cis*), 168.6 ($C_q$, *trans*), 163.7 (t, *J* = 33.3 Hz, $C_q$, *cis+trans*), 143.2 ($C_q$, *trans*), 142.8 ($C_q$, *cis*), 142.7 ($C_q$, *trans*), 141.5 ($C_q$, *cis*), 138.6 ($C_q$, *cis*), 138.4 ($C_q$, *trans*), 135.8 ($C_q$, *cis*), 135.7 ($C_q$, *trans*), 131.7 (CH, *cis*), 131.7 (CH, *trans*), 130.0 ($C_q$, *trans*), 130.0 (CH, *trans*), 129.9 ($C_q$, *cis*), 129.9 (CH, *cis*), 129.4 ($C_q$, *cis+trans*), 129.1 (CH, *cis*), 128.9 (CH, *cis*), 128.7 (CH, *cis*), 128.7 (CH, *trans*), 128.6 (CH, *trans*), 128.6 (CH, *cis*), 128.5 (CH, *trans*), 128.5 (CH, *trans*), 128.3 (CH, *trans*), 128.3 (CH, *cis*), 127.8 (CH, *cis*), 127.7 (CH, *cis*), 127.7 (CH, *trans*), 127.0 (CH, *trans*), 122.8 (CH, *cis*), 122.7 (CH, *trans*), 115.4 (t, *J* = 250.5 Hz, $C_q$, *cis*), 114.8 (t, *J* = 250.5 Hz, $C_q$, *trans*), 67.2 ($CH_2$, *trans*), 67.0 ($CH_2$, *cis*), 63.0 ($CH_2$, *cis*), 63.0 ($CH_2$, *trans*), 57.1 ($CH_2$, *cis*), 57.1 ($CH_2$, *trans*), 48.4 ($C_q$, *trans*), 47.8 ($C_q$, *cis*), 35.0 ($CH_3$, *cis+trans*), 33.5 (t, *J* = 26.0 Hz, CH, *trans*), 32.1 (t, *J* = 25.7 Hz,

CH, *cis*), 31.6 (t, $J$ = 5.0 Hz, $CH_2$, *trans*), 31.1 (dt, $J$ = 10.7, 4.6 Hz, $CH_2$, *cis*), 14.1 ($CH_3$, *cis*+*trans*). **$^{19}$F NMR** (377 MHz, $CDCl_3$) δ = -114.21 – -114.47 (m, *cis*+*trans*). **IR** (ATR): 2984, 1763, 1682, 1399, 1315, 1127, 824, 735 $cm^{-1}$. **MS** (ESI) *m*/*z* (relative intensity): 595 $[M+H]^+$ (5), 617 $[M+Na]^+$ (100). **HR-MS** (ESI) *m*/*z* calcd for $C_{34}H_{30}N_4O_4F_2$ $[M+H]^+$: 595.1806, found: 595.1820.

Compound **30** was obtained following the general procedure with **1a** (56 mg, 0.3 mmol), N-(4-methoxyphenyl)-1-phenylethan-1-imine (203 mg, 0.9 mmol), and ethyl 2-bromo-2,2-difluoroacetate (183 mg, 0.9 mmol) for 24 h and work-up with 3 N HCl for another 3 h. Afterwards, the organic phase was extracted by EtOAc and dried over $Na_2SO_4$. The combined organic solvent was removed under reduced pressure. The residue was purified by column chromatography on silica gel (*n*-hexane/EtOAc/DCM = 20/1/1→10/1/1) yielded **30** (52 mg, 40%) with two separable diastereomers (*cis*: *trans* = 2.5:1). **R$_f$** = 0.27 for *cis*-**30** and 0.33 for *trans*-**30** (TLC: *n*-hexane/EtOAc/DCM = 8/1/1).

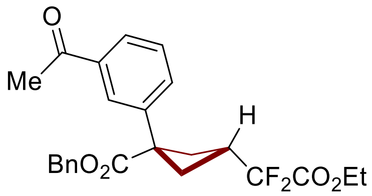


**Benzyl (1s,3s)-1-(3-acetylphenyl)-3-(2-ethoxy-1,1-difluoro-2-oxoethyl)cyclobutane-1-carboxylate** (*cis*-**30**, 37 mg, 29%) was obtained as a colorless oil. **$^1$H NMR** (500 MHz, $CDCl_3$) δ = 7.99 (t, $J$ = 1.9 Hz, 1H), 7.87 (dt, $J$ = 7.8, 1.4 Hz, 1H), 7.64 – 7.58 (m, 1H), 7.46 (t, $J$ = 7.7 Hz, 1H), 7.30 – 7.27 (m, 3H), 7.19 – 7.13 (m, 2H), 5.07 (s, 2H), 4.30 (q, $J$ = 7.1 Hz, 2H), 3.13 – 3.05 (m, 2H), 2.90 – 2.69 (m, 3H), 2.58 (s, 3H), 1.33 (t, $J$ = 7.1 Hz, 3H). **$^{13}$C NMR** (126 MHz, $CDCl_3$) δ = 197.9 ($C_q$), 173.9 ($C_q$), 163.7 (t, $J$ = 33.2 Hz, $C_q$), 141.8 ($C_q$), 137.6 ($C_q$), 135.7 ($C_q$), 131.5 (CH), 129.1 (CH), 128.6 (CH), 128.3 (CH), 127.9 (CH), 127.6 (CH), 126.4 (CH), 115.3 (t, $J$ = 250.7 Hz, $C_q$), 67.1 ($CH_2$), 63.1 ($CH_2$), 47.8 ($C_q$), 32.1 (t, $J$ = 25.8 Hz, CH), 31.1 (t, $J$ = 5.0 Hz, $CH_2$),

26.8 ($CH_3$), 14.1 ($CH_3$). **$^{19}$F NMR** (471 MHz, $CDCl_3$) δ = -114.30 (d, $J$ = 15.3 Hz). **IR** (ATR): 2961, 1763, 1729, 1685, 1217, 1127, 1038, 742, 698 $cm^{-1}$. **MS** (ESI) $m/z$ (relative intensity): 431 $[M+H]^+$ (5), 453 $[M+Na]^+$ (100). **HR-MS** (ESI) $m/z$ calcd for $C_{24}H_{24}O_5F_2$ $[M+H]^+$: 431.1665, found: 431.1664.

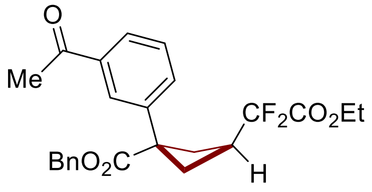


**Benzyl (1r,3r)-1-(3-acetylphenyl)-3-(2-ethoxy-1,1-difluoro-2-oxoethyl)cyclobutane-1-carboxylate** (*trans*-**30**, 15 mg, 11%) was obtained as a colorless oil. **$^1$H NMR** (500 MHz, $CDCl_3$) δ = 7.87 – 7.81 (m, 1H), 7.80 (s, 1H), 7.44 – 7.41 (m, 2H), 7.31 – 7.27 (m, 3H), 7.18 – 7.13 (m, 2H), 5.10 (s, 2H), 4.29 (q, $J$ = 7.2 Hz, 2H), 3.19 – 2.98 (m, 3H), 2.71 – 2.65 (m, 2H), 2.57 (s, 3H), 1.32 (t, $J$ = 7.1 Hz, 3H). **$^{13}$C NMR** (126 MHz, $CDCl_3$) δ = 197.9 ($C_q$), 174.3 ($C_q$), 163.7 (t, $J$ = 33.3 Hz, $C_q$), 143.4 ($C_q$), 137.4 ($C_q$), 135.7 ($C_q$), 130.8 (CH), 128.9 (CH), 128.7 (CH), 128.4 (CH), 127.8 (CH), 127.3 (CH), 125.9 (CH), 114.8 (t, $J$ = 250.7 Hz, $C_q$), 67.4 ($CH_2$), 63.1 ($CH_2$), 48.3 ($C_q$), 33.5 (t, $J$ = 26.0 Hz, CH), 31.6 (t, $J$ = 5.0 Hz, $CH_2$), 26.8 ($CH_3$), 14.1 ($CH_3$). **$^{19}$F NMR** (471 MHz, $CDCl_3$) δ = -114.55 (d, $J$ = 15.0 Hz). **IR** (ATR): 2922, 1763, 1728, 1685, 1260, 1211, 1057, 745, 68 $cm^{-1}$. **MS** (ESI) $m/z$ (relative intensity): 431 $[M+H]^+$ (5), 453 $[M+Na]^+$ (100). **HR-MS** (ESI) $m/z$ calcd for $C_{24}H_{24}O_5F_2$ $[M+H]^+$: 431.1665, found: 431.1671.

Compound **31** was obtained following the general procedure with **1b** (65 mg, 0.3 mmol), 2-phenylpyridine (140 mg, 0.9 mmol), and ethyl 2-bromo-2,2-difluoroacetate (183 mg, 0.9 mmol) for 36 h. Purification by column chromatography on silica gel (*n*-hexane/EtOAc = 10/1 → 5/1) yielded **31** (86 mg, 58%) with two separable diastereomers (*cis*: *trans* = 4.4:1). **$R_f$** = 0.30 for *cis*-**31** and 0.33 for *trans*-**31** (TLC: *n*-hexane/EtOAc/DCM = 5/1/1 for 2 times).

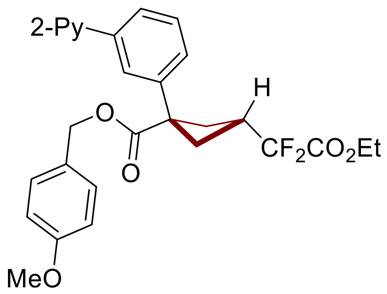


**4-Methoxybenzyl (1s,3s)-3-(2-ethoxy-1,1-difluoro-2-oxoethyl)-1-(3-(pyridin-2-yl)phenyl)cyclobutane-1-carboxylate** (*cis*-**31**, 70 mg, 47%) was obtained as a colorless oil. **$^1$H NMR** (400 MHz, Chloroform-*d*) δ = 8.70 (ddd, *J* = 4.8, 1.8, 0.9 Hz, 1H), 8.01 (s, 1H), 7.90 (ddd, *J* = 6.1, 2.9, 1.7 Hz, 1H), 7.75 (td, *J* = 7.7, 1.8 Hz, 1H), 7.65 (d, *J* = 8.0 Hz, 1H), 7.46 (dd, *J* = 5.8, 1.0 Hz, 2H), 7.29 – 7.21 (m, 1H), 7.17 – 7.09 (m, 2H), 6.83 – 6.74 (m, 2H), 5.02 (s, 2H), 4.29 (q, *J* = 7.1 Hz, 2H), 3.76 (s, 3H), 3.13 – 3.02 (m, 2H), 2.95 – 2.71 (m, 3H), 1.32 (t, *J* = 7.2 Hz, 3H). **$^{13}$C NMR** (101 MHz, $CDCl_3$) δ = 174.4 ($C_q$), 163.8 (t, *J* = 33.3 Hz, $C_q$), 159.6 ($C_q$), 157.1 ($C_q$), 149.7 (CH), 141.6 ($C_q$), 139.8 ($C_q$), 137.0 (CH), 129.7 (CH), 129.6 (CH), 129.1 (CH), 128.0 (CH), 127.3 (CH), 126.1 (CH), 125.4 (CH), 122.4 (CH), 120.9 (CH), 115.5 (t, *J* = 251.5 Hz, $C_q$), 113.9 (CH), 66.9 ($CH_2$), 63.0 ($CH_2$), 55.3 ($CH_3$), 47.9 ($C_q$), 32.1 (t, *J* = 25.7 Hz, CH), 31.1 (t, *J* = 4.9 Hz, $CH_2$), 14.1 ($CH_3$). **$^{19}$F NMR** (377 MHz, $CDCl_3$) δ = -114.25 (d, *J* = 15.3 Hz). **IR** (ATR): 2959, 1762, 1727, 1466, 1219, 1036, 823, 775 $cm^{-1}$. **MS** (ESI) *m*/*z* (relative intensity): 496 $[M+H]^+$ (20), 518 $[M+Na]^+$ (100). **HR-MS** (ESI) *m*/*z* calcd for $C_{28}H_{27}NO_5F_2$ $[M+H]^+$: 496.1930, found: 496.1936.

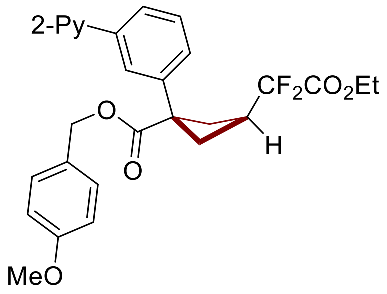


**4-Methoxybenzyl (1r,3r)-3-(2-ethoxy-1,1-difluoro-2-oxoethyl)-1-(3-(pyridin-2-yl)phenyl)cyclobutane-1-carboxylate** (*trans*-**31**, 16 mg, 11%) was obtained as a colorless oil. **$^1$H NMR**

(400 MHz, $CDCl_3$) δ = 8.71 – 8.68 (m, 1H), 7.88 (dt, *J* = 7.9, 1.3 Hz, 1H), 7.84 – 7.76 (m, 2H), 7.77 – 7.70 (m, 1H), 7.66 (dt, *J* = 8.0, 1.1 Hz, 1H), 7.43 (t, *J* = 7.8 Hz, 1H), 7.29 – 7.23 (m, 1H), 7.16 – 7.09 (m, 2H), 6.82 – 6.74 (m, 2H), 5.05 (s, 2H), 4.28 (q, *J* = 7.2 Hz, 2H), 3.76 (s, 3H), 3.16 – 2.94 (m, 3H), 2.78 – 2.67 (m, 2H), 1.31 (t, *J* = 7.1 Hz, 3H). **$^{13}$C NMR** (101 MHz, $CDCl_3$) δ = 174.7 ($C_q$), 163.8 (t, *J* = 33.3 Hz, $C_q$), 159.6 ($C_q$), 157.2 ($C_q$), 149.7 (CH), 143.4 ($C_q$), 139.6 ($C_q$), 137.0 (CH), 129.6 (CH), 129.0 (CH), 128.0 ($C_q$), 126.8 (CH), 125.8 (CH), 124.7 (CH), 122.4 (CH), 120.9 (CH), 115.0 (t, *J* = 250.5 Hz, $C_q$), 114.0 (CH), 67.1 ($CH_2$), 63.0 ($CH_2$), 55.4 ($CH_3$), 48.6 ($C_q$), 33.6 (t, *J* = 25.9 Hz, CH), 31.6 (t, *J* = 5.0 Hz, $CH_2$), 14.1 ($CH_3$). **$^{19}$F NMR** (377 MHz, $CDCl_3$) δ = -114.32 (d, *J* = 14.7 Hz). **IR** (ATR): 2958, 1763, 1726, 1466, 1173, 1060, 831, 776 $cm^{-1}$. **MS** (ESI) *m/z* (relative intensity): 496 $[M+H]^+$ (40), 518 $[M+Na]^+$ (100). **HR-MS** (ESI) *m/z* calcd for $C_{28}H_{27}NO_5F_2$ $[M+H]^+$: 496.1930, found: 496.1936.

Compound **32** was obtained following the general procedure with **1c** (77 mg, 0.3 mmol), 2-phenylpyridine (140 mg, 0.9 mmol), and ethyl 2-bromo-2,2-difluoroacetate (183 mg, 0.9 mmol) for 24 h. Purification by column chromatography on silica gel (*n*-hexane/EtOAc/DCM = 20/1/1→10/1/1) yielded **32** (106 mg, 66%) with two separable diastereomers (*cis*: *trans* = 2.3:1). **$R_f$** = 0.35 for *cis*-**32** and 0.38 for *trans*-**32** (TLC: *n*-hexane/EtOAc/DCM = 5/1/1).

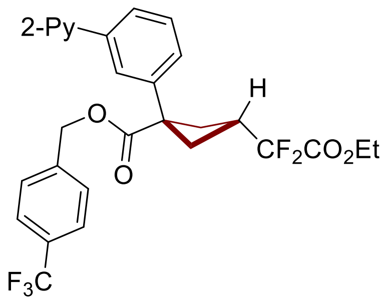


**4-(Trifluoromethyl)benzyl (1s,3s)-3-(2-ethoxy-1,1-difluoro-2-oxoethyl)-1-(3-(pyridin-2-yl)phenyl)cyclobutane-1-carboxylate** (*cis*-**32**, 74 mg, 46%) was obtained as a colorless oil. **$^1$H NMR** (500 MHz, $CDCl_3$) δ = 8.70 (ddd, *J* = 5.0, 1.8, 0.9 Hz, 1H), 8.06 (t, *J* = 1.9 Hz, 1H), 7.91

(dt, $J$ = 7.3, 1.7 Hz, 1H), 7.76 (td, $J$ = 7.7, 1.9 Hz, 1H), 7.68 (dt, $J$ = 8.0, 1.1 Hz, 1H), 7.51 – 7.44 (m, 4H), 7.28 – 7.23 (m, 3H), 5.13 (s, 2H), 4.30 (q, $J$ = 7.2 Hz, 2H), 3.13 – 3.07 (m, 2H), 2.98 – 2.78 (m, 3H), 1.33 (t, $J$ = 7.1 Hz, 3H). **$^{13}$C NMR** (126 MHz, $CDCl_3$) δ = 174.1 ($C_q$), 163.8 (t, $J$ = 33.2 Hz, $C_q$), 157.0 ($C_q$), 149.9 (CH), 141.4 ($C_q$), 140.1 ($C_q$), 139.9 ($C_q$), 137.0 (CH), 130.3 (q, $J$ = 32.5 Hz, $C_q$), 129.2 (CH), 127.8 (CH), 127.2 (CH), 126.2 (CH), 125.5 (q, $J$ = 3.8 Hz, CH), 125.5 (CH), 123.0 ($C_q$), 122.6 (CH), 120.8 (CH), 115.5 (t, $J$ = 250.2 Hz, $C_q$), 65.9 ($CH_2$), 63.1 ($CH_2$), 47.9 ($C_q$), 32.2 (t, $J$ = 25.7 Hz, CH), 31.1 (t, $J$ = 4.9 Hz, $CH_2$), 14.1 ($CH_3$). **$^{19}$F NMR** (471 MHz, $CDCl_3$) δ = -62.64, -114.33 (d, $J$ = 15.3 Hz). **IR** (ATR): 2962, 1764, 1731, 1589, 1322, 1119, 1061, 823, 772 $cm^{-1}$. **MS** (ESI) *m/z* (relative intensity): 534 $[M+H]^+$ (70), 556 $[M+Na]^+$ (100). **HR-MS** (ESI) *m/z* calcd for $C_{28}H_{24}NO_4F_5$ $[M+Na]^+$: 556.1518, found: 556.1529.

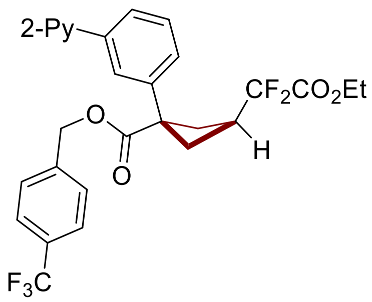


**4-(Trifluoromethyl)benzyl (1r,3r)-3-(2-ethoxy-1,1-difluoro-2-oxoethyl)-1-(3-(pyridin-2-yl)phenyl)cyclobutane-1-carboxylate (*trans*-32**, 32 mg, 20%) was obtained as a colorless oil. **$^1$H NMR** (500 MHz, $CDCl_3$) δ = 8.70 (ddd, $J$ = 4.8, 1.8, 1.0 Hz, 1H), 7.91 – 7.84 (m, 2H), 7.79 – 7.71 (m, 1H), 7.68 (dt, $J$ = 8.0, 1.1 Hz, 1H), 7.49 – 7.43 (m, 3H), 7.31 – 7.20 (m, 4H), 5.16 (s, 2H), 4.29 (q, $J$ = 7.2 Hz, 2H), 3.20 – 2.98 (m, 3H), 2.82 – 2.73 (m, 2H), 1.32 (t, $J$ = 7.1 Hz, 3H). **$^{13}$C NMR** (126 MHz, $CDCl_3$) δ = 174.5 ($C_q$), 163.8 (t, $J$ = 33.2 Hz, $C_q$), 157.1 ($C_q$), 149.9 (CH), 143.1 ($C_q$), 139.9 ($C_q$), 139.8 ($C_q$), 136.9 (CH), 130.4 (q, $J$ = 32.4 Hz, $C_q$), 129.1 (CH), 127.6 (CH), 126.6 (CH), 125.9 (CH), 125.6 (q, $J$ = 3.7 Hz, CH), 124.8 (CH), 122.5 (CH), 120.8 (CH), 114.9 (t, $J$ = 250.2 Hz, $C_q$), 66.2 ($CH_2$), 63.0 ($CH_2$), 48.6 ($C_q$), 33.6 (t, $J$ = 26.0 Hz, CH), 31.6 (t, $J$ = 5.0 Hz, $CH_2$),

14.1 ($CH_3$). **$^{19}$F NMR** (471 MHz, $CDCl_3$) δ = -62.67, -114.34 (d, $J$ = 14.8 Hz). **IR** (ATR): 2959, 1764, 1730, 1579, 1322, 1117, 1060, 822, 774 $cm^{-1}$. **MS** (ESI) *m*/*z* (relative intensity): 534 $[M+H]^+$ (70), 556 $[M+Na]^+$ (100). **HR-MS** (ESI) *m*/*z* calcd for $C_{28}H_{24}NO_4F_5$ $[M+Na]^+$: 556.1518, found: 556.1517.

Compound **33** was obtained following the general procedure with **1d** (70 mg, 0.3 mmol), 2-phenylpyridine (140 mg, 0.9 mmol), and ethyl 2-bromo-2,2-difluoroacetate (183 mg, 0.9 mmol) for 24 h. Purification by column chromatography on silica gel (*n*-hexane/EtOAc/DCM = 15/1/1→12/1/1) yielded **33** (87 mg, 57%) with two separable diastereomers (*cis*: *trans* = 3.1:1). **R**$_f$ = 0.43 for *cis*-**33** and 0.48 for *trans*-**33** (TLC: *n*-hexane/EtOAc/DCM = 3/1/1).

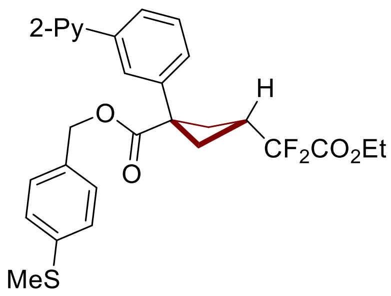


**4-(Methylthio)benzyl (1s,3s)-3-(2-ethoxy-1,1-difluoro-2-oxoethyl)-1-(3-(pyridin-2-yl)phenyl)cyclobutane-1-carboxylate** (*cis*-**33**, 66 mg, 43%) was obtained as a colorless oil. **$^1$H NMR** (500 MHz, $CDCl_3$) δ = 8.73 – 8.67 (m, 1H), 8.03 (t, $J$ = 1.8 Hz, 1H), 7.90 (dt, $J$ = 6.8, 2.0 Hz, 1H), 7.75 (td, $J$ = 7.7, 1.9 Hz, 1H), 7.65 (dt, $J$ = 7.9, 1.1 Hz, 1H), 7.51 – 7.43 (m, 2H), 7.27 – 7.23 (m, 1H), 7.14 – 7.08 (m, 4H), 5.03 (s, 2H), 4.29 (q, $J$ = 7.1 Hz, 2H), 3.11 – 3.05 (m, 2H), 2.93 – 2.74 (m, 3H), 2.43 (s, 3H), 1.33 (t, $J$ = 7.1 Hz, 3H). **$^{13}$C NMR** (126 MHz, $CDCl_3$) δ = 174.3 ($C_q$), 163.8 (t, $J$ = 33.3 Hz, $C_q$), 157.1 ($C_q$), 149.8 (CH), 141.5 ($C_q$), 140.0 ($C_q$), 138.6 ($C_q$), 136.9 (CH), 132.6 ($C_q$), 129.1 (CH), 128.5 (CH), 127.2 (CH), 126.5 (CH), 126.1 (CH), 125.4 (CH), 122.5 (CH), 120.8 (CH), 115.4 (t, $J$ = 250.2 Hz, $C_q$), 66.6 ($CH_2$), 63.0 ($CH_2$), 47.8 ($C_q$), 32.1 (t, $J$ = 25.6 Hz, CH), 31.1 (t, $J$ = 4.9 Hz, $CH_2$), 15.8 ($CH_3$), 14.1 ($CH_3$). **$^{19}$F NMR** (471 MHz, $CDCl_3$) δ = -114.26 (d, $J$ = 15.4 Hz). **IR** (ATR): 2990, 1762, 1728, 1437, 1220, 1128, 1039,

772 cm$^{-1}$. **MS** (ESI) *m*/*z* (relative intensity): 512 [M+H]$^+$ (70), 534 [M+Na]$^+$ (100). **HR-MS** (ESI) *m*/*z* calcd for $C_{28}H_{27}NO_4SF_2$ [M+H]$^+$: 512.1702, found: 512.1713.

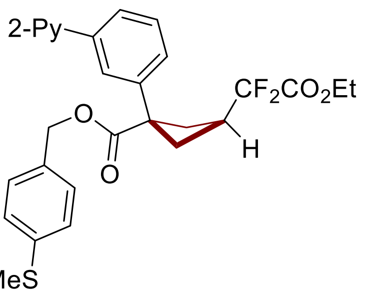


**4-(Methylthio)benzyl (1r,3r)-3-(2-ethoxy-1,1-difluoro-2-oxoethyl)-1-(3-(pyridin-2-yl)phenyl)cyclobutane-1-carboxylate** (*trans*-**33**, 21 mg, 14%) was obtained as a colorless oil. **$^1$H NMR** (500 MHz, $CDCl_3$) δ = 8.70 (ddd, *J* = 4.8, 1.8, 1.0 Hz, 1H), 7.91 – 7.85 (m, 1H), 7.82 (t, *J* = 1.6 Hz, 1H), 7.79 – 7.71 (m, 1H), 7.66 (dt, *J* = 8.0, 1.1 Hz, 1H), 7.44 (td, *J* = 7.7, 0.5 Hz, 1H), 7.29 – 7.23 (m, 2H), 7.14 – 7.06 (m, 4H), 5.06 (s, 2H), 4.28 (q, *J* = 7.1 Hz, 2H), 3.18 – 2.96 (m, 3H), 2.77 – 2.70 (m, 2H), 2.43 (s, 3H), 1.32 (t, *J* = 7.1 Hz, 3H). **$^{13}$C NMR** (126 MHz, $CDCl_3$) δ = 174.7 ($C_q$), 163.8 (t, *J* = 33.3 Hz, $C_q$), 157.2 ($C_q$), 149.8 (CH), 143.3 ($C_q$), 139.7 ($C_q$), 138.8 ($C_q$), 136.9 (CH), 132.6 ($C_q$), 129.0 (CH), 128.5 (CH), 126.7 (CH), 126.5 (CH), 125.8 (CH), 124.7 (CH), 122.4 (CH), 120.9 (CH), 114.9 (t, *J* = 250.1 Hz, $C_q$), 66.9 (CH2), 63.0 ($CH_2$), 48.6 ($C_q$), 33.6 (t, *J* = 26.0 Hz, CH), 31.6 (t, *J* = 4.9 Hz, $CH_2$), 15.8 ($CH_3$), 14.1 ($CH_3$). **$^{19}$F NMR** (471 MHz, $CDCl_3$) δ = -114.33 (d, *J* = 14.8 Hz). **IR** (ATR): 2989, 1762, 1726, 1437, 1211, 1122, 1053, 774 cm$^{-1}$. **MS** (ESI) *m*/*z* (relative intensity): 512 [M+H]$^+$ (95), 534 [M+Na]$^+$ (100). **HR-MS** (ESI) *m*/*z* calcd for $C_{28}H_{27}NO_4SF_2$ [M+H]$^+$: 512.1702, found: 512.1709.

Compound **34** was obtained following the general procedure with **1e** (67 mg, 0.3 mmol), 2-phenylpyridine (140 mg, 0.9 mmol), and ethyl 2-bromo-2,2-difluoroacetate (183 mg, 0.9 mmol) for 24 h. Purification by column chromatography on silica gel (*n*-hexane/EtOAc = 10/1→5/1) yielded **34** (80 mg, 53%) with two separable diastereomers (*cis*: *trans* = 3.2:1). **R$_f$** = 0.35 for *cis*-**34** and 0.40 for *trans*-**34** (TLC:

*n*-hexane/EtOAc = 3/1).

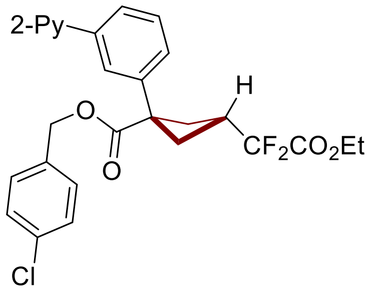


**4-Chlorobenzyl (1s,3s)-3-(2-ethoxy-1,1-difluoro-2-oxoethyl)-1-(3-(pyridin-2-yl)phenyl)cyclobutane-1-carboxylate** (*cis*-**34**, 61 mg, 41%) was obtained as a cololess oil. **$^1$H NMR** (500 MHz, $CDCl_3$) δ = 8.70 (ddd, $J$ = 4.8, 1.8, 1.0 Hz, 1H), 8.03 (t, $J$ = 1.5 Hz, 1H), 7.90 (dt, $J$ = 7.3, 1.7 Hz, 1H), 7.76 (ddd, $J$ = 8.0, 7.4, 1.8 Hz, 1H), 7.66 (dt, $J$ = 8.0, 1.1 Hz, 1H), 7.49 – 7.43 (m, 2H), 7.29 – 7.23 (m, 1H), 7.22 – 7.18 (m, 2H), 7.11 – 7.08 (m, 2H), 5.04 (s, 2H), 4.30 (q, $J$ = 7.2 Hz, 2H), 3.11 – 3.05 (m, 2H), 2.94 – 2.75 (m, 3H), 1.33 (t, $J$ = 7.1 Hz, 3H). **$^{13}$C NMR** (126 MHz, $CDCl_3$) δ = 174.2 ($C_q$), 163.8 (t, $J$ = 33.3 Hz, $C_q$), 157.1 ($C_q$), 149.8 (CH), 141.5 ($C_q$), 139.9 ($C_q$), 137.0 (CH), 134.4 ($C_q$), 134.0 ($C_q$), 129.2 (CH), 129.2 (CH), 128.7 (CH), 127.2 (CH), 126.1 (CH), 125.4 (CH), 122.5 (CH), 120.9 (CH), 115.5 (t, $J$ = 250.2 Hz, $C_q$), 66.1 ($CH_2$), 63.0 ($CH_2$), 47.9 ($C_q$), 32.1 (t, $J$ = 25.7 Hz, CH), 31.1 (t, $J$ = 4.9 Hz, $CH_2$), 14.1 ($CH_3$). **$^{19}$F NMR** (471 MHz, $CDCl_3$) δ = -114.30 (d, $J$ = 15.4 Hz). **IR** (ATR): 2961, 1730, 1590, 1220, 1127, 1040, 774, 695 cm$^{-1}$. **MS** (ESI) *m*/*z* (relative intensity): 500 $[M+H]^+$ (80), 522 $[M+Na]^+$ (100). **HR-MS** (ESI) *m*/*z* calcd for $C_{27}H_{24}NO_4F_2Cl$ $[M+H]^+$: 500.1435, found: 500.1431.

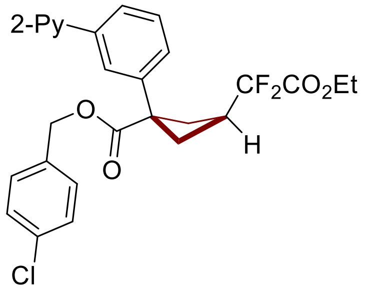


**4-Chlorobenzyl**

**(1r,3r)-3-(2-ethoxy-1,1-difluoro-2-oxoethyl)-1-(3-(pyridin-2-yl)phenyl)cyclobutan e-1-carboxylate** (*trans*-**34**, 19 mg, 12%) was obtained as a colorless oil. **$^1$H NMR** (500 MHz, $CDCl_3$) δ = 8.70 (ddd, $J$ = 4.8, 1.8, 1.0 Hz, 1H), 7.88 (dt, $J$ = 7.8, 1.5 Hz, 1H), 7.83 (t, $J$ = 1.8 Hz, 1H), 7.76 (td, $J$ = 7.7, 1.9 Hz, 1H), 7.67 (dt, $J$ = 8.0, 1.1 Hz, 1H), 7.44 (t, $J$ = 7.7 Hz, 1H), 7.28 – 7.24 (m, 2H), 7.22 – 7.18 (m, 2H), 7.11 – 7.04 (m, 2H), 5.07 (s, 2H), 4.29 (q, $J$ = 7.1 Hz, 2H), 3.18 – 2.98 (m, 3H), 2.79 – 2.72 (m, 2H), 1.32 (t, $J$ = 7.1 Hz, 3H). **$^{13}$C NMR** (126 MHz, $CDCl_3$) δ = 174.6 ($C_q$), 163.8 (t, $J$ = 33.3 Hz, $C_q$), 157.1 ($C_q$), 149.8 (CH), 143.2 ($C_q$), 139.8 ($C_q$), 137.0 (CH), 134.3 (CH), 134.1 (CH), 129.1 (CH), 129.0 (CH), 128.8 (CH), 126.7 (CH), 125.9 (CH), 124.7 (CH), 122.5 (CH), 120.8 (CH), 114.9 (t, $J$ = 250.1 Hz, $C_q$), 66.4 ($CH_2$), 63.0 ($CH_2$), 48.5 ($C_q$), 33.6 (t, $J$ = 26.0 Hz, CH), 31.6 (t, $J$ = 4.9 Hz, $CH_2$), 14.1 ($CH_3$). **$^{19}$F NMR** (471 MHz, $CDCl_3$) δ = -114.33 (d, $J$ = 14.9 Hz). **IR** (ATR): 2957, 1728, 1591, 1271, 1126, 1017, 775, 700 $cm^{-1}$. **MS** (ESI) *m/z* (relative intensity): 500 $[M+H]^+$ (100), 522 $[M+Na]^+$ (80). **HR-MS** (ESI) *m/z* calcd for $C_{27}H_{24}NO_4F_2Cl$ $[M+H]^+$: 500.1435, found: 500.1438.

Compound **35** was obtained following the general procedure with **1f** (67 mg, 0.3 mmol), 2-phenylpyridine (140 mg, 0.9 mmol), and ethyl 2-bromo-2,2-difluoroacetate (183 mg, 0.9 mmol) for 24 h. Purification by column chromatography on silica gel (*n*-hexane/EtOAc/DCM = 10/1/1 → 8/1/1) yielded **35** (98 mg, 65%) with two separable diastereomers (*cis*: *trans* = 3.3:1). **$R_f$** = 0.29 for *cis*-**35** and 0.34 for *trans*-**35** (TLC: *n*-hexane/EtOAc/DCM = 3/1/1).

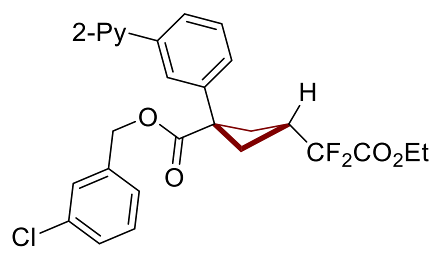


**3-Chlorobenzyl (1s,3s)-3-(2-ethoxy-1,1-difluoro-2-oxoethyl)-1-(3-(pyridin-2-yl)phenyl)cyclobutan e-1-carboxylate** (*cis*-**35**, 75 mg, 50%) was obtained as a colorless oil. **$^1$H NMR** (500

MHz, $CDCl_3$) δ = 8.70 (ddd, *J* = 4.8, 1.8, 0.9 Hz, 1H), 8.06 (t, *J* = 1.6 Hz, 1H), 7.91 (dt, *J* = 7.3, 1.7 Hz, 1H), 7.75 (td, *J* = 7.7, 1.8 Hz, 1H), 7.68 (dt, *J* = 8.0, 1.1 Hz, 1H), 7.52 – 7.44 (m, 2H), 7.27 – 7.23 (m, 1H), 7.23 – 7.09 (m, 3H), 7.03 (d, *J* = 7.4 Hz, 1H), 5.05 (s, 2H), 4.30 (q, *J* = 7.1 Hz, 2H), 3.14 – 3.06 (m, 2H), 2.95 – 2.77 (m, 3H), 1.33 (t, *J* = 7.1 Hz, 3H). **$^{13}$C NMR** (126 MHz, $CDCl_3$) δ = 174.1 ($C_q$), 163.8 (t, *J* = 33.3 Hz, $C_q$), 157.1 ($C_q$), 149.9 (CH), 141.4 ($C_q$), 140.1 ($C_q$), 137.9 ($C_q$), 136.9 (CH), 134.4 ($C_q$), 129.8 (CH), 129.2 (CH), 128.3 (CH), 127.7 (CH), 127.3 (CH), 126.2 (CH), 125.7 (CH), 125.4 (CH), 122.5 (CH), 120.9 (CH), 115.5 (t, *J* = 250.2 Hz, $C_q$), 66.0 ($CH_2$), 63.0 ($CH_2$), 47.9 ($C_q$), 32.1 (t, *J* = 25.7 Hz, CH), 31.1 (t, *J* = 4.9 Hz, $CH_2$), 14.1 ($CH_3$). **$^{19}$F NMR** (471 MHz, $CDCl_3$) δ = -114.30 (d, *J* = 15.1 Hz). **IR** (ATR): 2923, 1763, 1730, 1577, 1437, 1217, 1039, 774, 739 $cm^{-1}$. **MS** (ESI) *m*/*z* (relative intensity): 500 $[M+H]^+$ (65), 522 $[M+Na]^+$ (100). **HR-MS** (ESI) *m*/*z* calcd for $C_{27}H_{24}NO_4F_2Cl$ $[M+H]^+$: 500.1435, found: 500.1437.

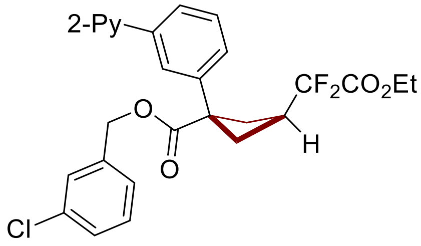


**3-Chlorobenzyl (1r,3r)-3-(2-ethoxy-1,1-difluoro-2-oxoethyl)-1-(3-(pyridin-2-yl)phenyl)cyclobutane-1-carboxylate** (*trans*-**35**, 23 mg, 15%) was obtained as a colorless oil. **$^1$H NMR** (500 MHz, $CDCl_3$) δ = 8.70 (ddd, *J* = 4.8, 1.9, 1.0 Hz, 1H), 7.91 – 7.83 (m, 2H), 7.79 – 7.71 (m, 1H), 7.69 (dt, *J* = 8.0, 1.1 Hz, 1H), 7.45 (td, *J* = 7.7, 0.6 Hz, 1H), 7.31 – 7.26 (m, 1H), 7.26 – 7.23 (m, 1H), 7.22 – 7.14 (m, 2H), 7.11 – 7.08 (m, 1H), 7.02 – 6.99 (m, 1H), 5.08 (s, 2H), 4.29 (q, *J* = 7.1 Hz, 2H), 3.20 – 2.98 (m, 3H), 2.81 – 2.72 (m, 2H), 1.32 (t, *J* = 7.2 Hz, 3H). **$^{13}$C NMR** (126 MHz, $CDCl_3$) δ = 174.6 ($C_q$), 163.8 (t, *J* = 33.3 Hz, $C_q$), 157.2 ($C_q$), 149.8 (CH), 143.1 ($C_q$), 139.8 ($C_q$), 137.8 ($C_q$), 136.9 (CH), 134.5 ($C_q$), 129.9 (CH), 129.1 (CH), 128.3 (CH), 127.6 (CH), 126.7 (CH), 126.0 (CH), 125.6 (CH), 124.7 (CH), 122.4 (CH), 120.9 (CH), 114.9 (t, *J* = 250.1 Hz, $C_q$), 66.2 ($CH_2$), 63.0 ($CH_2$), 48.6 ($C_q$), 33.6 (t, *J* = 26.0 Hz, CH), 31.6 (t, *J* = 5.0 Hz,

$CH_2$), 14.1 ($CH_3$). **$^{19}$F NMR** (471 MHz, $CDCl_3$) δ = -114.33 (d, *J* = 14.8 Hz). **IR** (ATR): 2956, 1763, 1729, 1578, 1269, 1210, 1123, 777, 692 cm$^{-1}$. **MS** (ESI) *m*/*z* (relative intensity): 500 $[M+H]^+$ (90), 522 $[M+Na]^+$ (100). **HR-MS** (ESI) *m*/*z* calcd for $C_{27}H_{24}NO_4F_2Cl$ $[M+H]^+$: 500.1435, found: 500.1447.

Compound **36** was obtained following the general procedure with **1g** (77 mg, 0.3 mmol), 2-phenylpyridine (140 mg, 0.9 mmol), and ethyl 2-bromo-2,2-difluoroacetate (183 mg, 0.9 mmol) for 24 h. Purification by column chromatography on silica gel (*n*-hexane/EtOAc/DCM = 12/1/1→10/1/1) yielded **36** (101 mg, 63%) with two separable diastereomers (*cis*: *trans* = 2.7:1). **R**$_f$ = 0.37 for *cis*-**36** and 0.40 for *trans*-**36** (TLC: *n*-hexane/EtOAc/DCM = 3/1/1).

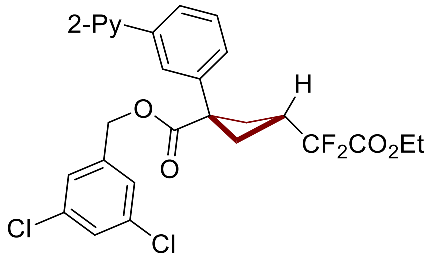


**3,5-Dichlorobenzyl (1s,3s)-3-(2-ethoxy-1,1-difluoro-2-oxoethyl)-1-(3-(pyridin-2-yl)phenyl)cyclobutane-1-carboxylate** (*cis*-**36**, 74 mg, 46%) was obtained as a colorless oil. **$^1$H NMR** (500 MHz, $CDCl_3$) δ = 8.70 (ddd, *J* = 4.8, 1.8, 1.0 Hz, 1H), 8.07 (t, *J* = 1.6 Hz, 1H), 7.94 – 7.88 (m, 1H), 7.79 – 7.72 (m, 1H), 7.70 (dt, *J* = 8.0, 1.1 Hz, 1H), 7.53 – 7.42 (m, 2H), 7.27 – 7.23 (m, 1H), 7.20 (t, *J* = 1.9 Hz, 1H), 6.97 (dt, *J* = 1.9, 0.6 Hz, 2H), 5.01 (s, 2H), 4.31 (q, *J* = 7.1 Hz, 2H), 3.15 – 3.06 (m, 2H), 2.96 – 2.79 (m, 3H), 1.34 (t, *J* = 7.1 Hz, 3H). **$^{13}$C NMR** (126 MHz, $CDCl_3$) δ = 173.9 ($C_q$), 163.8 (t, *J* = 33.2 Hz, $C_q$), 157.0 ($C_q$), 149.9 (CH), 141.3 ($C_q$), 140.1 ($C_q$), 139.2 ($C_q$), 136.9 (CH), 135.1 ($C_q$), 129.3 (CH), 128.2 (CH), 127.2 (CH), 126.3 (CH), 125.8 (CH), 125.3 (CH), 122.5 (CH), 120.8 (CH), 115.4 (t, *J* = 250.2 Hz, $C_q$), 65.1 ($CH_2$), 63.1 ($CH_2$), 47.9 ($C_q$), 32.2 (t, *J* = 25.7 Hz, CH), 31.1 (t, *J* = 4.9 Hz, $CH_2$), 14.1 ($CH_3$). **$^{19}$F NMR** (471 MHz, $CDCl_3$) δ = -114.33 (d, *J* = 15.2 Hz). **IR** (ATR): 2958, 1764, 1731, 1564, 1215, 1160,

1039, 854, 771 cm$^{-1}$. **MS** (ESI) *m*/*z* (relative intensity): 534 [M+H]$^+$ (70), 556 [M+Na]$^+$ (100). **HR-MS** (ESI) *m*/*z* calcd for $C_{27}H_{23}NO_4F_2Cl_2$ [M+H]$^+$: 534.1045, found: 534.1055.

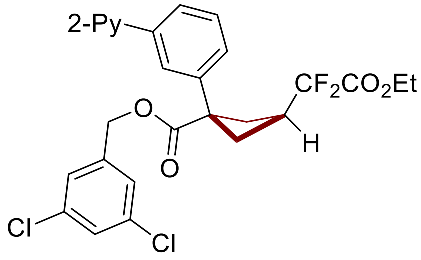


**3,5-Dichlorobenzyl (1r,3r)-3-(2-ethoxy-1,1-difluoro-2-oxoethyl)-1-(3-(pyridin-2-yl)phenyl)cyclobutane-1-carboxylate** (*trans*-**36**, 27 mg, 17%) was obtained as a colorless oil. **$^1$H NMR** (400 MHz, $CDCl_3$) δ = 8.70 (ddd, *J* = 4.8, 1.8, 0.9 Hz, 1H), 7.92 – 7.85 (m, 2H), 7.78 – 7.68 (m, 2H), 7.47 (t, *J* = 8.0 Hz, 1H), 7.30 – 7.19 (m, 3H), 6.94 (d, *J* = 1.9 Hz, 2H), 5.04 (s, 2H), 4.29 (q, *J* = 7.1 Hz, 2H), 3.23 – 2.98 (m, 3H), 2.83 – 2.73 (m, 2H), 1.33 (t, *J* = 7.1 Hz, 3H). **$^{13}$C NMR** (101 MHz, $CDCl_3$) δ = 174.4 ($C_q$), 163.8 (t, *J* = 33.3 Hz, $C_q$), 157.1 ($C_q$), 149.8 (CH), 143.0 ($C_q$), 139.9 ($C_q$), 139.2 ($C_q$), 136.9 (CH), 135.2 ($C_q$), 129.1 (CH), 128.3 (CH), 126.6 (CH), 126.1 (CH), 125.6 (CH), 124.6 (CH), 122.5 (CH), 120.8 (CH), 114.9 (t, *J* = 250.2 Hz, $C_q$), 65.3 ($CH_2$), 63.0 ($CH_2$), 48.5 ($C_q$), 33.6 (t, *J* = 26.0 Hz, CH), 31.6 (t, *J* = 5.0 Hz, $CH_2$), 14.1 ($CH_3$). **$^{19}$F NMR** (377 MHz, $CDCl_3$) δ = -114.31 (d, *J* = 14.6 Hz). **IR** (ATR): 2958, 1763, 1729, 1564, 1208, 1112, 1052, 854, 772 cm$^{-1}$. **MS** (ESI) *m*/*z* (relative intensity): 534 [M+H]$^+$ (100), 556 [M+Na]$^+$ (90). **HR-MS** (ESI) *m*/*z* calcd for $C_{27}H_{23}NO_4F_2Cl_2$ [M+H]$^+$: 534.1045, found: 534.1061.

Compound **37** was obtained following the general procedure with **1h** (77 mg, 0.3 mmol), 2-phenylpyridine (140 mg, 0.9 mmol), and ethyl 2-bromo-2,2-difluoroacetate (183 mg, 0.9 mmol) for 24 h. Purification by column chromatography on silica gel (*n*-hexane/EtOAc/DCM = 20/1/1 → 10/1/1) yielded **37** (103 mg, 64%) with two separable diastereomers (*cis*: *trans* = 2.6:1). **R**$_f$ = 0.29 for *cis*-**37** and 0.31 for *trans*-**37**

(TLC: *n*-hexane/EtOAc/DCM = 3/1/1).

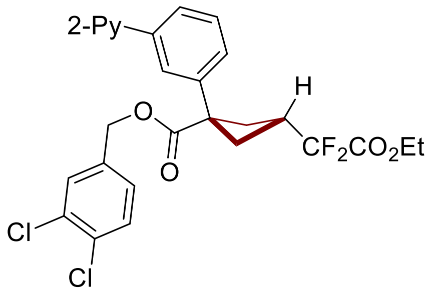


**3,4-Dichlorobenzyl (1r,3r)-3-(2-ethoxy-1,1-difluoro-2-oxoethyl)-1-(3-(pyridin-2-yl)phenyl)cyclobutane-1-carboxylate** (*cis*-**37**, 74 mg, 46%) was obtained as a colorless oil. **$^1$H NMR** (500 MHz, $CDCl_3$) δ = 8.70 (ddd, *J* = 4.9, 1.9, 0.9 Hz, 1H), 8.05 (t, *J* = 1.9 Hz, 1H), 7.90 (dt, *J* = 7.6, 1.5 Hz, 1H), 7.76 (td, *J* = 7.7, 1.8 Hz, 1H), 7.68 (dt, *J* = 8.0, 1.1 Hz, 1H), 7.51 – 7.43 (m, 2H), 7.30 – 7.24 (m, 2H), 7.19 (d, *J* = 2.0 Hz, 1H), 6.97 (dd, *J* = 8.2, 2.0 Hz, 1H), 5.02 (s, 2H), 4.30 (q, *J* = 7.1 Hz, 2H), 3.13 – 3.05 (m, 2H), 2.95 – 2.77 (m, 3H), 1.33 (t, *J* = 7.1 Hz, 3H). **$^{13}$C NMR** (126 MHz, $CDCl_3$) δ = 174.0 ($C_q$), 163.8 (t, *J* = 33.3 Hz, $C_q$), 157.0 ($C_q$), 149.9 (CH), 141.4 ($C_q$), 140.1 ($C_q$), 137.0 (CH), 136.1 ($C_q$), 132.7 ($C_q$), 132.2 ($C_q$), 130.5 (CH), 129.6 (CH), 129.2 (CH), 127.2 (CH), 127.0 (CH), 126.2 (CH), 125.3 (CH), 122.5 (CH), 120.8 (CH), 115.4 (t, *J* = 250.2 Hz, $C_q$), 65.3 ($CH_2$), 63.1 ($CH_2$), 47.9 ($C_q$), 32.1 (t, *J* = 25.8 Hz, CH), 31.1 (t, *J* = 4.9 Hz, $CH_2$), 14.1 ($CH_3$). **$^{19}$F NMR** (471 MHz, $CDCl_3$) δ = -114.33 (d, *J* = 15.2 Hz). **IR** (ATR): 2960, 1764, 1730, 1589, 1468, 1216, 1126, 1036, 773 $cm^{-1}$. **MS** (ESI) *m*/*z* (relative intensity): 534 $[M+H]^+$ (80), 556 $[M+Na]^+$ (100). **HR-MS** (ESI) *m*/*z* calcd for $C_{27}H_{23}NO_4F_2Cl_2$ $[M+H]^+$: 534.1045, found: 534.1056.

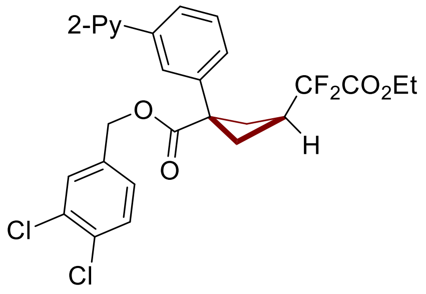


**3,4-Dichlorobenzyl**

**(1s,3s)-3-(2-ethoxy-1,1-difluoro-2-oxoethyl)-1-(3-(pyridin-2-yl)phenyl)cyclobutane-1-carboxylate** (*trans*-**37**, 29 mg, 18%) was obtained as a colorless oil. **$^1$H NMR** (500 MHz, $CDCl_3$) δ = 8.70 (ddd, *J* = 4.8, 1.9, 0.9 Hz, 1H), 7.91 – 7.83 (m, 2H), 7.79 – 7.72 (m, 1H), 7.68 (dt, *J* = 8.0, 1.1 Hz, 1H), 7.45 (td, *J* = 7.7, 0.6 Hz, 1H), 7.31 – 7.22 (m, 3H), 7.17 (d, *J* = 2.0 Hz, 1H), 6.94 (dd, *J* = 8.2, 2.1 Hz, 1H), 5.04 (s, 2H), 4.29 (q, *J* = 7.2 Hz, 2H), 3.19 – 2.97 (m, 3H), 2.81 – 2.73 (m, 2H), 1.32 (t, *J* = 7.1 Hz, 3H). **$^{13}$C NMR** (126 MHz, $CDCl_3$) δ = 174.5 ($C_q$), 163.8 (t, *J* = 33.3 Hz, $C_q$), 157.1 ($C_q$), 149.8 (CH), 143.0 ($C_q$), 139.8 ($C_q$), 137.0 (CH), 136.0 ($C_q$), 132.8 ($C_q$), 132.3 ($C_q$), 130.6 (CH), 129.5 (CH), 129.1 (CH), 126.8 (CH), 126.6 (CH), 126.0 (CH), 124.7 (CH), 122.5 (CH), 120.8 (CH), 114.9 (t, *J* = 250.7 Hz, $C_q$), 65.5 ($CH_2$), 63.1 ($CH_2$), 48.5 ($C_q$), 33.6 (t, *J* = 26.0 Hz, CH), 31.6 (t, *J* = 5.0 Hz, $CH_2$), 14.1 ($CH_3$). **$^{19}$F NMR** (471 MHz, $CDCl_3$) δ = -114.33 (d, *J* = 14.9 Hz). **IR** (ATR): 2959, 1764, 1730, 1589, 1469, 1211, 1123, 1024, 775 cm$^{-1}$. **MS** (ESI) *m*/*z* (relative intensity): 534 $[M+H]^+$ (100), 556 $[M+Na]^+$ (100). **HR-MS** (ESI) *m*/*z* calcd for $C_{27}H_{23}NO_4F_2Cl_2$ $[M+H]^+$: 534.1045, found: 534.1060.

Compound **38** was obtained following the general procedure with **1i** (58 mg, 0.3 mmo), 2-phenylpyridine (140 mg, 0.9 mmol), and ethyl 2-bromo-2,2-difluoroacetate (183 mg, 0.9 mmol) for 24 h. Purification by column chromatography on silica gel (*n*-hexane/EtOAc/DCM = 15/1/1 → 8/1/1) yielded **38** (92 mg, 65%) with two separable diastereomers (*cis*: *trans* = 3.2:1). **R$_f$** = 0.29 for *cis*-**38** and 0.31 for *trans*-**38** (TLC: *n*-hexane/EtOAc/DCM = 5/1/1).

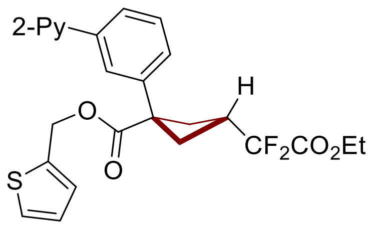


**Thiophen-2-ylmethyl (1s,3s)-3-(2-ethoxy-1,1-difluoro-2-oxoethyl)-1-(3-(pyridin-2-yl)phenyl)cyclobutane-1-carboxylate** (*cis*-**38**, 70 mg, 50%) as a colorless oil. **$^1$H NMR** (500 MHz, $CDCl_3$)

δ = 8.72 – 8.67 (m, 1H), 8.02 (s, 1H), 7.93 – 7.87 (m, 1H), 7.74 (td, *J* = 7.7, 1.9 Hz, 1H), 7.67 (d, *J* = 8.0 Hz, 1H), 7.49 – 7.43 (m, 2H), 7.28 – 7.21 (m, 2H), 6.98 (d, *J* = 2.4 Hz, 1H), 6.91 (dd, *J* = 5.1, 3.5 Hz, 1H), 5.23 (s, 2H), 4.29 (q, *J* = 7.2 Hz, 2H), 3.11 – 3.03 (m, 2H), 2.95 – 2.73 (m, 3H), 1.32 (t, *J* = 7.2 Hz, 3H). **$^{13}$C NMR** (126 MHz, $CDCl_3$) δ = 174.1 ($C_q$), 163.8 (t, *J* = 33.3 Hz, $C_q$), 157.2 ($C_q$), 149.8 (CH), 141.3 ($C_q$), 139.9 ($C_q$), 137.8 ($C_q$), 136.9 (CH), 129.1 (CH), 127.9 (CH), 127.3 (CH), 126.8 (CH), 126.7 (CH), 126.1 (CH), 125.3 (CH), 122.4 (CH), 120.9 (CH), 115.4 (t, *J* = 250.2 Hz, $C_q$), 63.0 ($CH_2$), 61.5 ($CH_2$), 47.7 ($C_q$), 32.1 (t, *J* = 25.8 Hz, CH), 31.1 (t, *J* = 4.9 Hz, $CH_2$), 14.1 ($CH_3$). **$^{19}$F NMR** (471 MHz, $CDCl_3$) δ = -114.26 (d, *J* = 15.4 Hz). **IR** (ATR): 2959, 1762, 1467, 1308, 1220, 1036, 773, 700 $cm^{-1}$. **MS** (ESI) *m*/*z* (relative intensity): 472 $[M+H]^+$ (60), 494 $[M+Na]^+$ (100). **HR-MS** (ESI) *m*/*z* calcd for $C_{25}H_{23}NO_4F_2S$ $[M+Na]^+$: 494.1208, found: 494.1207.

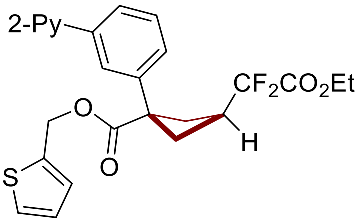


**Thiophen-2-ylmethyl (1r,3r)-3-(2-ethoxy-1,1-difluoro-2-oxoethyl)-1-(3-(pyridin-2-yl)phenyl)cyclobutane-1-carboxylate** (*trans*-**38**, 22 mg, 15%) as a colorless oil. **$^1$H NMR** (500 MHz, $CDCl_3$) δ = 8.72 – 8.66 (m, 1H), 7.87 (dt, *J* = 7.7, 1.4 Hz, 1H), 7.81 (t, *J* = 1.9 Hz, 1H), 7.74 (td, *J* = 7.7, 1.9 Hz, 1H), 7.67 (d, *J* = 8.0 Hz, 1H), 7.43 (t, *J* = 7.7 Hz, 1H), 7.28 – 7.22 (m, 3H), 6.99 (d, *J* = 2.5 Hz, 1H), 6.92 (dd, *J* = 5.1, 3.5 Hz, 1H), 5.27 (s, 2H), 4.28 (q, *J* = 7.1 Hz, 2H), 3.16 – 3.03 (m, 1H), 3.04 – 2.95 (m, 2H), 2.77 – 2.69 (m, 2H), 1.31 (t, *J* = 7.1 Hz, 3H). **$^{13}$C NMR** (126 MHz, $CDCl_3$) δ = 174.6 ($C_q$), 163.8 (t, *J* = 33.3 Hz, $C_q$), 157.3 ($C_q$), 149.8 (CH), 143.1 ($C_q$), 139.8 ($C_q$), 137.9 ($C_q$), 136.9 (CH), 129.0 (CH), 127.9 (CH), 126.8 (CH), 126.8 (CH), 126.7 (CH), 125.9 (CH), 124.7 (CH), 122.4 (CH), 120.9 (CH), 114.94 (t, *J* = 250.0 Hz, $C_q$), 63.0 ($CH_2$), 61.8 ($CH_2$), 48.5 ($C_q$), 33.6 (t, *J* = 25.9 Hz, CH), 31.7 (t, *J* = 5.0 Hz, $CH_2$), 14.1 ($CH_3$). **$^{19}$F NMR** (471 MHz, $CDCl_3$) δ = -114.34 (d, *J* = 15.0 Hz). **IR** (ATR): 2959, 1763, 1468,

1309, 1210, 1052, 775, 703 cm$^{-1}$. **MS** (ESI) *m*/*z* (relative intensity): 472 $[M+H]^+$ (70), 494 $[M+Na]^+$ (100). **HR-MS** (ESI) *m*/*z* calcd for $C_{25}H_{23}NO_4F_2S$ $[M+Na]^+$: 494.1208, found: 494.1214.

Compound **39** was obtained following the general procedure with **1j** (62 mg, 0.3 mmol), 2-phenylpyridine (140 mg, 0.9 mmol), and ethyl 2-bromo-2,2-difluoroacetate (183 mg, 0.9 mmol) for 24 h. Purification by column chromatography on silica gel (*n*-hexane/EtOAc = 10/1 → 5/1) yielded **39** (80 mg, 55%) with two separable diastereomers (*cis*: *trans* = 4.5:1). **R**$_f$ = 0.25 for *cis*-**39** and 0.28 for *trans*-**39** (TLC: *n*-hexane/EtOAc = 5/1).

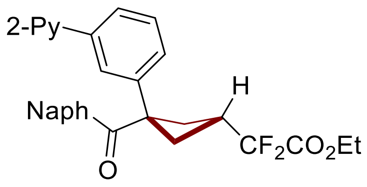


**Ethyl 2-((1s,3s)-3-(2-naphthoyl)-3-(3-(pyridin-2-yl)phenyl)cyclobutyl)-2,2-difluoroacetate (*cis*-39**, 65 mg, 45%) was obtained as a colorless oil. **$^1$H NMR** (500 MHz, $CDCl_3$) δ = 8.71 (dt, *J* = 4.9, 1.3 Hz, 1H), 8.30 – 8.22 (m, 2H), 7.90 – 7.80 (m, 3H), 7.79 – 7.66 (m, 4H), 7.56 (dt, *J* = 7.8, 1.4 Hz, 1H), 7.55 – 7.42 (m, 3H), 7.27 – 7.20 (m, 1H), 4.30 (q, *J* = 7.1 Hz, 2H), 3.21 (q, *J* = 7.9, 7.3 Hz, 3H), 2.90 – 2.81 (m, 2H), 1.32 (t, *J* = 7.1 Hz, 3H). **$^{13}$C NMR** (126 MHz, $CDCl_3$) δ = 199.7 ($C_q$), 163.9 (t, *J* = 33.2 Hz, $C_q$), 157.0 ($C_q$), 149.8 (CH), 142.2 ($C_q$), 140.7 ($C_q$), 136.9 (CH), 135.4 ($C_q$), 132.4 ($C_q$), 131.7 (CH), 131.0 (Cq), 129.8 (CH), 129.7 (CH), 128.6 (CH), 128.3 (CH), 127.7 (CH), 126.7 (CH), 126.4 (CH), 126.0 (CH), 125.2 (CH), 124.3 (CH), 122.5 (CH), 120.8 (CH), 115.4 (t, *J* = 250.3 Hz, $C_q$), 63.0 ($CH_2$), 52.6 ($C_q$), 32.4 (t, *J* = 25.8 Hz, CH), 31.6 (t, *J* = 4.8 Hz, $CH_2$), 14.1 ($CH_3$). **$^{19}$F NMR** (471 MHz, $CDCl_3$) δ = -114.01 (d, *J* = 13.2 Hz). **IR** (ATR): 2986, 1762, 1673, 1316, 1171, 1131, 773 cm$^{-1}$. **MS** (ESI) *m*/*z* (relative intensity): 486 $[M+H]^+$ (40), 508 $[M+Na]^+$ (100). **HR-MS** (ESI) *m*/*z* calcd for $C_{30}H_{25}NO_3F_2$ $[M+H]^+$: 486.1875, found: 486.1887.

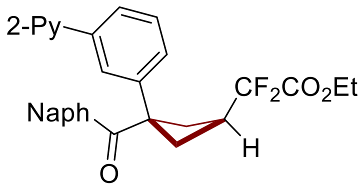


**Ethyl 2-((1r,3r)-3-(2-naphthoyl)-3-(3-(pyridin-2-yl)phenyl)cyclobutyl)-2,2-difluoroacetate (*trans*-39**,15 mg, 10%) was obtained as a colorless oil. **$^{1}$H NMR** (500 MHz, $CDCl_3$) δ = 8.69 (d, *J* = 4.2 Hz, 1H), 8.33 (s, 1H), 8.04 (s, 1H), 7.88 – 7.70 (m, 6H), 7.67 (d, *J* = 7.9 Hz, 1H), 7.56 – 7.38 (m, 4H), 7.25 – 7.22 (m, 1H), 4.29 (q, *J* = 7.2 Hz, 2H), 3.23 – 3.14 (m, 2H), 3.04 – 2.93 (m, 3H), 1.32 (t, *J* = 7.2 Hz, 3H). **$^{13}$C NMR** (126 MHz, $CDCl_3$) δ = 199.6 ($C_q$), 163.9 (t, *J* = 33.4 Hz, $C_q$), 157.0 ($C_q$), 149.8 (CH), 143.4 ($C_q$), 135.3 ($C_q$), 132.4 ($C_q$), 132.1 (CH), 130.9 ($C_q$), 129.8 (CH), 129.7 (CH), 128.7 (CH), 128.2 (CH), 127.7 (CH), 126.8 (CH), 126.4 (CH), 125.8 (CH), 125.6 (CH), 124.3 (CH), 122.5 (CH), 120.9 (CH), 115.3 (t, *J* = 249.9 Hz, $C_q$), 63.0 ($CH_2$), 53.3 ($C_q$), 32.9 (t, *J* = 25.9 Hz, CH), 31.4 (t, *J* = 4.8 Hz, $CH_2$), 14.1 ($CH_3$). **$^{19}$F NMR** (471 MHz, $CDCl_3$) δ = -114.11 (d, *J* = 13.7 Hz). **IR** (ATR): 2928, 1762, 1673, 1437, 1312, 1119, 1057, 769 $cm^{-1}$. **MS** (ESI) *m/z* (relative intensity): 486 $[M+H]^+$ (70), 508 $[M+Na]^+$ (100). **HR-MS** (ESI) *m/z* calcd for $C_{30}H_{25}NO_3F_2$ $[M+H]^+$: 486.1875, found: 486.1881.

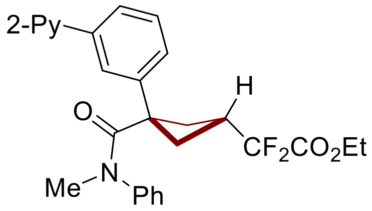


**Ethyl 2,2-difluoro-2-(3-(methyl(phenyl)carbamoyl)-3-(3-(pyridin-2-yl)phenyl)cyclobutyl)acetate (40)**. The product was obtained following the general procedure with **1k** (56 mg, 0.3 mmol), 2-phenylpyridine (140 mg, 0.9 mmol), and ethyl 2-bromo-2,2-difluoroacetate (183 mg, 0.9 mmol) for 24 h. Purification by column

chromatography on silica gel (*n*-hexane/EtOAc = 10/1→2/1) yielded **40** (57 mg, 41%) as a colorless oil with two diastereomers (*cis*: *trans* = 20:1, determined by **$^{19}$F-NMR**). **R$_f$** = 0.15 (TLC: *n*-hexane/EtOAc = 3/1). **$^1$H NMR** (500 MHz, $CDCl_3$) (*cis*-isomer) δ = 8.69 (d, *J* = 4.7 Hz, 1H), 7.91 (d, *J* = 7.7 Hz, 1H), 7.75 (td, *J* = 7.7, 1.8 Hz, 1H), 7.67 – 7.56 (m, 2H), 7.35 (t, *J* = 7.9 Hz, 1H), 7.26 – 7.06 (m, 5H), 6.64 (d, *J* = 7.5 Hz, 2H), 4.24 (q, *J* = 7.0 Hz, 2H), 3.20 (s, 3H), 2.89 (t, *J* = 10.9 Hz, 2H), 2.73 – 2.59 (m, 1H), 2.10 – 1.99 (m, 2H), 1.28 (t, *J* = 7.2 Hz, 3H). **$^{13}$C NMR** (126 MHz, $CDCl_3$) (*cis*-isomer) δ = 174.0 ($C_q$), 163.9 (t, *J* = 33.5 Hz, $C_q$), 157.2 ($C_q$), 149.8 (CH), 142.5 ($C_q$), 142.2 ($C_q$), 139.9 ($C_q$), 136.8 (CH), 129.1 (CH), 128.9 (CH), 128.0 (CH), 126.3 (CH), 125.7 (CH), 124.2 (CH), 122.4 (CH), 120.7 (CH), 115.2 (t, *J* = 250.1 Hz, $C_q$), 62.9 ($CH_2$), 49.0 ($C_q$), 39.3 ($CH_3$), 32.2 ($CH_2$), 32.0 (t, *J* = 25.8 Hz, CH), 14.1 ($CH_3$). **$^{19}$F NMR** (471 MHz, $CDCl_3$) δ = -113.63 (d, *J* = 14.7 Hz, *cis*), -114.12 (d, *J* = 14.9 Hz, *trans*). **IR** (ATR): 2995, 1761, 1645, 1367, 1121, 773, 740, 699 cm$^{-1}$. **MS** (ESI) *m*/*z* (relative intensity): 465 $[M+H]^+$ (10), 487 $[M+Na]^+$ (100). **HR-MS** (ESI) *m*/*z* calcd for $C_{27}H_{26}N_2O_3F_2$ $[M+H]^+$: 465.1984, found: 465.1979.

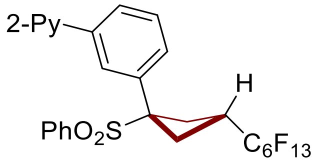


**2-(3-(3-(Perfluorohexyl)-1-(phenylsulfonyl)cyclobutyl)phenyl)pyridine (41)**. The product was obtained following the general procedure with **1l** (58 mg, 0.3 mmol), 2-phenylpyridine (140 mg, 0.9 mmol), and ethyl 2-bromo-2,2-difluoroacetate (183 mg, 0.9 mmol) for 24 h. Purification by column chromatography on silica gel (*n*-hexane/EtOAc = 10/1→3/1) yielded **41** (85 mg, 43%) as a colorless oil with two diastereomers, separable by column chromatography (*cis*: *trans* = 16:1). **R$_f$** = 0.26 (TLC: *n*-hexane/EtOAc = 3/1). **$^1$H NMR** (500 MHz, $CDCl_3$) (*cis*-isomer) δ = 8.66 (ddd, *J* = 4.8, 1.8, 0.9 Hz, 1H), 7.97 (ddd, *J* = 7.8, 1.8, 1.0 Hz, 1H), 7.77 – 7.70 (m, 2H), 7.56 – 7.47 (m, 2H), 7.40 – 7.28 (m, 5H), 7.24 (ddd, *J* = 7.5, 4.8, 1.1 Hz, 1H), 7.15 (ddd, *J* = 7.8, 2.0, 1.0 Hz, 1H), 3.58 (td, *J* = 10.0, 2.7 Hz, 2H), 3.01 – 2.88 (m, 1H), 2.82 – 2.73 (m, 2H). **$^{13}$C NMR** (126 MHz, $CDCl_3$) (*cis*-isomer) δ = 156.5 ($C_q$),

149.9 (CH), 139.7 ($C_q$), 136.9 (CH), 134.9 ($C_q$), 134.4 ($C_q$), 133.9 (CH), 129.8 (CH), 129.6 (CH), 128.8 (CH), 128.7 (CH), 128.0 (CH), 127.6 (CH), 122.7 (CH), 120.7 (CH), 65.4 ($C_q$), 29.5 (CH), 29.3 (t, $J$ = 5.4 Hz, $CH_2$). **$^{19}$F NMR** (471 MHz, $CDCl_3$) δ = -80.79, -120.60 (q, $J$ = 16.0, 15.0 Hz), -122.14 (t, $J$ = 14.5 Hz), -122.87, -126.09 – -126.20 (m). **IR** (ATR): 3069, 2962, 1199, 1144, 770, 691, 616, 587 cm$^{-1}$. **MS** (ESI) *m*/*z* (relative intensity): 668 $[M+H]^+$ (10), 690 $[M+Na]^+$ (100). **HR-MS** (ESI) *m*/*z* calcd for $C_{27}H_{18}NO_2F_{13}S$ $[M+Na]^+$: 690.0743, found: 690.0747.

Compound **42** was obtained following the general procedure with **1m** (61 mg, 0.3 mmol), 2-phenylpyridine (140 mg, 0.9 mmol), and ethyl 2-bromo-2,2-difluoroacetate (183 mg, 0.9 mmol) for 24 h. Purification by column chromatography on silica gel (*n*-hexane/EtOAc = 10/1→5/1) yielded **42** (121 mg, 84%) with two separable diastereomers (*major*: *minor* = 2.8:1). **R**$_f$= 0.30 for *major*-**42** and 0.33 for *minor*-**42** (TLC: *n*-hexane/EtOAc = 3/1).

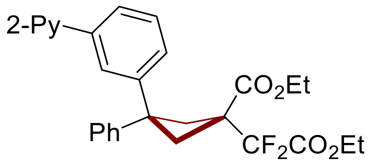


**Ethyl (1r,3r)-1-(2-ethoxy-1,1-difluoro-2-oxoethyl)-3-phenyl-3-(3-(pyridin-2-yl)phenyl)cyclobutane-1-carboxylate** (*major*-**42**, 89 mg, 62%) was obtained as a colorless oil. **$^1$H NMR** (500 MHz, $CDCl_3$) δ 8.66 = (ddd, $J$ = 4.8, 1.9, 1.0 Hz, 1H), 8.03 (t, $J$ = 1.8 Hz, 1H), 7.77 – 7.69 (m, 2H), 7.67 – 7.64 (m, 1H), 7.41 – 7.34 (m, 2H), 7.28 – 7.22 (m, 4H), 7.20 (ddd, $J$ = 7.3, 4.8, 1.2 Hz, 1H), 7.12 – 7.09 (m, 1H), 4.28 (q, $J$ = 7.2 Hz, 2H), 3.88 (q, $J$ = 7.1 Hz, 2H), 3.52 – 3.41 (m, 4H), 1.32 (t, $J$ = 7.1 Hz, 3H), 0.87 (t, $J$ = 7.1 Hz, 3H). **$^{13}$C NMR** (126 MHz, $CDCl_3$) δ = 170.9 (t, $J$ = 5.1 Hz, $C_q$), 163.2 (t, $J$ = 32.8 Hz, $C_q$), 157.5 ($C_q$), 149.8 (CH), 149.2 ($C_q$), 147.9 ($C_q$), 139.5 ($C_q$), 136.9 (CH), 128.9 (CH), 128.6 (CH), 127.3 (CH), 126.1 (CH), 125.6 (CH), 125.1 (CH), 124.8 (CH), 122.3 (CH), 120.8 (CH), 114.2 (t, $J$ = 253.8 Hz, $C_q$), 63.0 ($CH_2$), 61.9 ($CH_2$), 48.3 (t, $J$ = 25.9 Hz, $C_q$), 45.6 ($C_q$), 38.7 (t, $J$ = 3.7 Hz, $CH_2$), 14.0 ($CH_3$), 13.5 ($CH_3$).

**$^{19}$F NMR** (471 MHz, $CDCl_3$) δ = -112.96. **IR** (ATR): 2981, 1771, 1726, 1314, 1135, 1067, 775, 703 cm$^{-1}$. **MS** (ESI) *m*/*z* (relative intensity): 480 $[M+H]^+$ (80), 502 $[M+Na]^+$ (100). **HR-MS** (ESI) *m*/*z* calcd for $C_{28}H_{27}NO_4F_2$ $[M+Na]^+$: 502.1800, found: 502.1813.

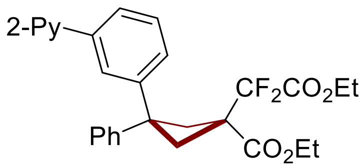


**Ethyl (1s,3s)-1-(2-ethoxy-1,1-difluoro-2-oxoethyl)-3-phenyl-3-(3-(pyridin-2-yl)phenyl)cyclobutane-1-carboxylate** (*minor*-**42**, 32 mg, 22%) was obtained as a colorless oil. **$^1$H NMR** (400 MHz, $CDCl_3$) δ = 8.67 (dd, *J* = 5.1, 1.5 Hz, 1H), 7.87 (t, *J* = 1.9 Hz, 1H), 7.75 – 7.62 (m, 3H), 7.44 – 7.32 (m, 3H), 7.28 – 7.17 (m, 4H), 7.14 – 7.08 (m, 1H), 4.29 (q, *J* = 7.2 Hz, 2H), 3.91 (q, *J* = 7.1 Hz, 2H), 3.52 – 3.42 (m, 4H), 1.33 (t, *J* = 7.1 Hz, 3H), 0.93 (t, *J* = 7.1 Hz, 3H). **$^{13}$C NMR** (101 MHz, $CDCl_3$) δ = 170.9 (t, *J* = 5.2 Hz, $C_q$), 163.2 (t, *J* = 32.8 Hz, $C_q$), 157.5 ($C_q$), 150.0 ($C_q$), 149.8 (CH), 147.1 (CH), 139.8 (CH), 136.8 (CH), 129.0 (CH), 128.5 (CH), 126.5 (CH), 126.2 (CH), 126.1 (CH), 124.7 (CH), 124.1 (CH), 122.3 (CH), 120.8 (CH), 114.2 (t, *J* = 253.9 Hz, $C_q$), 63.0 ($CH_2$), 61.9 ($CH_2$), 48.3 (t, *J* = 25.9 Hz, $C_q$), 45.66 ($C_q$), 38.7 (t, *J* = 3.7 Hz, $CH_2$), 14.0 ($CH_3$), 13.6 ($CH_3$). **$^{19}$F NMR** (377 MHz, $CDCl_3$) δ = -112.87. **IR** (ATR): 2981, 1770, 1726, 1311, 1137, 1072, 774, 702 cm$^{-1}$. **MS** (ESI) *m*/*z* (relative intensity): 480 $[M+H]^+$ (60), 502 $[M+Na]^+$ (100). **HR-MS** (ESI) *m*/*z* calcd for $C_{28}H_{27}NO_4F_2$ $[M+Na]^+$: 502.1800, found: 502.1819.

Compound **43** was obtained following the general procedure with **1m** (61 mg, 0.3 mmol), 5-methyl-2-phenylpyrimidine (153 mg, 0.9 mmol), and ethyl 2-bromo-2,2-difluoroacetate (183 mg, 0.9 mmol) for 24 h. Purification by column chromatography on silica gel (*n*-hexane/EtOAc/DCM = 20/1/1→12/1/1) yielded **43** (114 mg, 77%) with two separable diastereomers (*major*: *minor* = 1.6:1). **$R_f$** = 0.29 for

*major*-**43** and 0.31 for *minor*-**43** (TLC: *n*-hexane/EtOAc/DCM = 5/1/1).

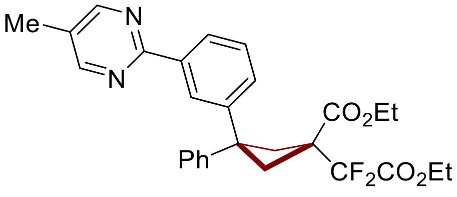


**Ethyl (1r,3r)-1-(2-ethoxy-1,1-difluoro-2-oxoethyl)-3-(3-(5-methylpyrimidin-2-yl)phenyl)-3-phenylcyclobutane-1-carboxylate** (*major*-**43**, 70 mg, 47%) was obtained as a colorless oil. **$^1$H NMR** (500 MHz, $CDCl_3$) δ = 8.61 (d, *J* = 0.8 Hz, 2H), 8.50 (t, *J* = 1.9 Hz, 1H), 8.20 (dt, *J* = 7.8, 1.4 Hz, 1H), 7.49 – 7.43 (m, 1H), 7.39 (t, *J* = 7.7 Hz, 1H), 7.32 – 7.24 (m, 4H), 7.15 – 7.08 (m, 1H), 4.31 (q, *J* = 7.2 Hz, 2H), 3.88 (q, *J* = 7.2 Hz, 2H), 3.57 – 3.44 (m, 4H), 2.33 (s, 3H), 1.35 (t, *J* = 7.2 Hz, 3H), 0.87 (t, *J* = 7.1 Hz, 3H). **$^{13}$C NMR** (126 MHz, $CDCl_3$) δ = 171.0 ($C_q$), 163.2 (t, *J* = 32.7 Hz, $C_q$), 162.4 ($C_q$), 157.5 (CH), 149.4 ($C_q$), 147.8 ($C_q$), 137.8 ($C_q$), 128.8 (CH), 128.7 (CH), 128.6 (CH), 128.5 ($C_q$), 126.0 (CH), 125.9 (CH), 125.8 (CH), 125.6 (CH), 114.3 (t, *J* = 253.6 Hz, $C_q$), 63.0 ($CH_2$), 61.9 ($CH_2$), 48.3 (t, *J* = 25.9 Hz, $C_q$), 45.6 ($C_q$), 38.7 (t, *J* = 3.7 Hz, $C_q$), 15.7 ($CH_3$), 14.1 ($CH_3$), 13.5 ($CH_3$). **$^{19}$F NMR** (471 MHz, $CDCl_3$) δ = -113.03. **IR** (ATR): 2988, 1771, 1725, 1428, 1312, 1136, 1065, 703 $cm^{-1}$. **MS** (ESI) *m*/*z* (relative intensity): 495 $[M+H]^+$ (40), 517 $[M+Na]^+$ (100). **HR-MS** (ESI) *m*/*z* calcd for $C_{28}H_{28}N_2O_4F_2$ $[M+Na]^+$: 517.1909, found: 517.1920.

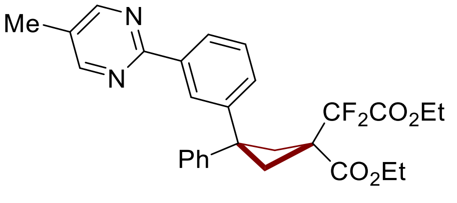


**Ethyl (1s,3s)-1-(2-ethoxy-1,1-difluoro-2-oxoethyl)-3-(3-(5-methylpyrimidin-2-yl)phenyl)-3-phenylcyclobutane-1-carboxylate** (*minor*-**43**, 44 mg, 30%) was obtained as a colorless oil. **$^1$H NMR** (500 MHz, $CDCl_3$) δ = 8.61 (d, *J* = 0.8 Hz, 2H), 8.33 (t, *J* = 1.7 Hz, 1H), 8.18 (dt, *J* = 7.7, 1.4 Hz, 1H), 7.46 – 7.43 (m, 2H), 7.38 (t, *J* = 7.4 Hz, 1H),

7.35 – 7.29 (m, 1H), 7.29 – 7.24 (m, 2H), 7.12 (t, $J$ = 7.4 Hz, 1H), 4.30 (q, $J$ = 7.1 Hz, 2H), 3.93 (q, $J$ = 7.1 Hz, 2H), 3.54 – 3.44 (m, 4H), 2.32 (s, 3H), 1.34 (t, $J$ = 7.1 Hz, 3H), 0.94 (t, $J$ = 7.1 Hz, 3H). **$^{13}$C NMR** (126 MHz, $CDCl_3$) δ = 170.9 ($C_q$), 163.2 (t, $J$ = 32.8 Hz, $C_q$), 162.4 ($C_q$), 157.4 (CH), 149.9 ($C_q$), 147.3 ($C_q$), 138.0 ($C_q$), 128.9 (CH), 128.5 (CH), 128.4 ($C_q$), 127.6 (CH), 126.4 (CH), 126.1 (CH), 125.7 (CH), 124.8 (CH), 114.2 (t, $J$ = 253.9 Hz, $C_q$), 63.0 ($CH_2$), 61.9 ($CH_2$), 48.35 (t, $J$ = 26.0 Hz, $C_q$), 45.7 ($C_q$), 38.6 (t, $J$ = 3.7 Hz, $CH_2$), 15.7 ($CH_3$), 14.1 ($CH_3$), 13.6 ($CH_3$). **$^{19}$F NMR** (471 MHz, $CDCl_3$) δ = -112.92. **IR** (ATR): 2988, 1771, 1723, 1310, 1135, 1071, 910, 732 $cm^{-1}$. **MS** (ESI) *m*/*z* (relative intensity): 495 $[M+H]^+$ (40), 517 $[M+Na]^+$ (100). **HR-MS** (ESI) *m*/*z* calcd for $C_{28}H_{28}N_2O_4F_2$ $[M+Na]^+$: 517.1909, found: 517.1916.

Compound **44** was obtained following the general procedure with **1m** (61 mg, 0.3 mmol), 2-phenyl-4,5-dihydrooxazole (132 mg, 0.9 mmol), and ethyl 2-bromo-2,2-difluoroacetate (183 mg, 0.9 mmol) for 24 h. Purification by column chromatography on silica gel (*n*-hexane/EtOAc = 5/1→2/1) yielded **44** (68 mg, 48%) with two separable diastereomers (*major*: *minor* = 1.4:1). **R**$_f$= 0.14 for *major*-**44** and 0.17 for *minor*-**44** (TLC: *n*-hexane/EtOAc = 3/1).

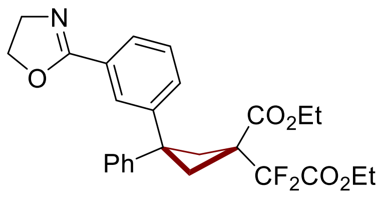


**Ethyl (1r,3r)-3-(3-(4,5-dihydrooxazol-2-yl)phenyl)-1-(2-ethoxy-1,1-difluoro-2-oxoethyl)-3-phenylcyclobutane-1-carboxylate** (*major*-**44**, 40 mg, 28%) was obtained as a colorless oil. **$^1$H NMR** (500 MHz, $CDCl_3$) δ = 8.00 (t, $J$ = 1.6 Hz, 1H), 7.72 (dt, $J$ = 7.7, 1.2 Hz, 1H), 7.49 (ddd, $J$ = 7.9, 2.1, 1.2 Hz, 1H), 7.33 (t, $J$ = 7.5 Hz, 1H), 7.28 – 7.26 (m, 1H), 7.26 – 7.22 (m, 3H), 7.14 – 7.10 (m, 1H), 4.41 (t, $J$ = 9.4 Hz, 2H), 4.30 (q, $J$ = 7.2 Hz, 2H), 4.05 (t, $J$ = 9.5 Hz, 2H), 3.91 (q, $J$ = 7.1 Hz, 2H), 3.46 – 3.42 (m, 4H), 1.34 (t, $J$ = 7.1 Hz, 3H), 0.92 (t, $J$ = 7.2 Hz, 3H). **$^{13}$C NMR** (126 MHz, $CDCl_3$) δ

= 170.8 (t, $J$ = 5.0 Hz, $C_q$), 164.7 ($C_q$), 163.1 (t, $J$ = 32.7 Hz, $C_q$), 149.0 ($C_q$), 147.9 ($C_q$), 129.5 (CH), 128.7 (CH), 128.6 (CH), 127.9 ($C_q$), 126.1 (CH), 126.1 (CH), 126.0 (CH), 125.5 (CH), 114.2 (t, $J$ = 253.5 Hz, $C_q$), 67.7 ($CH_2$), 63.0 ($CH_2$), 61.9 ($CH_2$), 55.0 ($CH_2$), 48.1 (t, $J$ = 26.0 Hz, $C_q$), 45.4 ($C_q$), 38.6 (t, $J$ = 3.4 Hz, $CH_2$), 14.0 ($CH_3$), 13.5 ($CH_3$). **$^{19}$F NMR** (471 MHz, $CDCl_3$) δ = -113.07. **IR** (ATR): 2986, 1771, 1726, 1310, 1136, 1065, 704, 583 $cm^{-1}$. **MS** (ESI) *m*/*z* (relative intensity): 472 $[M+H]^+$ (85), 494 $[M+Na]^+$ (100). **HR-MS** (ESI) *m*/*z* calcd for $C_{26}H_{27}NO_5F_2$ $[M+Na]^+$: 494.1750, found: 494.1760.

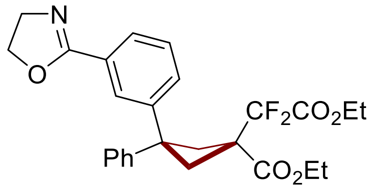


**Ethyl (1s,3s)-3-(3-(4,5-dihydrooxazol-2-yl)phenyl)-1-(2-ethoxy-1,1-difluoro-2-oxoethyl)-3-phenylcyclobutane-1-carboxylate** (*minor*-**44**, 28 mg, 20%) was obtained as a colorless oil. **$^1$H NMR** (400 MHz, $CDCl_3$) δ = 7.87 (t, $J$ = 1.8 Hz, 1H), 7.71 (dt, $J$ = 7.3, 1.6 Hz, 1H), 7.41 – 7.31 (m, 4H), 7.29 – 7.23 (m, 2H), 7.15 – 7.10 (m, 1H), 4.41 (t, $J$ = 9.5 Hz, 2H), 4.30 (q, $J$ = 7.2 Hz, 2H), 4.04 (t, $J$ = 9.5 Hz, 2H), 3.92 (q, $J$ = 7.1 Hz, 2H), 3.46 – 3.42 (m, 4H), 1.34 (t, $J$ = 7.1 Hz, 3H), 0.93 (t, $J$ = 7.1 Hz, 3H). **$^{13}$C NMR** (101 MHz, $CDCl_3$) δ = 170.8 (t, $J$ = 5.2 Hz, $C_q$), 164.8 ($C_q$), 163.1 (t, $J$ = 32.8 Hz, $C_q$), 149.9 ($C_q$), 147.0 ($C_q$), 128.7 (CH), 128.6 (CH), 128.5(CH), 127.9 ($C_q$), 126.3 (CH), 126.2 (CH), 125.9 (CH), 125.1 (CH), 114.1 (t, $J$ = 253.8 Hz, $C_q$), 67.7 ($CH_2$), 63.0 ($CH_2$), 61.9 ($CH_2$), 54.9 ($CH_2$), 48.2 (t, $J$ = 26.0 Hz, $C_q$), 45.4 ($C_q$), 38.5 (t, $J$ = 3.8 Hz, $CH_2$), 14.0 ($CH_3$), 13.5 ($CH_3$). **$^{19}$F NMR** (377 MHz, $CDCl_3$) δ = -113.05. **IR** (ATR): 2985, 1770, 1725, 1310, 1136, 1066, 810, 705 $cm^{-1}$. **MS** (ESI) *m*/*z* (relative intensity): 472 $[M+H]^+$ (60), 494 $[M+Na]^+$ (100). **HR-MS** (ESI) *m*/*z* calcd for $C_{26}H_{27}NO_5F_2$ $[M+Na]^+$: 494.1750, found: 494.1767.

Compound **45** was obtained following the general procedure with **1m** (61 mg, 0.3

mmol), 2-phenylthiazole (145 mg, 0.9 mmol), and ethyl 2-bromo-2,2-difluoroacetate (183 mg, 0.9 mmol) for 24 h. Purification by column chromatography on silica gel (*n*-hexane/EtOAc/DCM = 20/1/1 → 12/1/1) yielded **45** (80 mg, 55%) with two separable diastereomers (*major*: *minor* = 2:1). $\mathbf{R_f}$ = 0.40 for *major*-**45** and 0.43 for *minor*-**45** (TLC: *n*-hexane/EtOAc/DCM = 5/1/1).

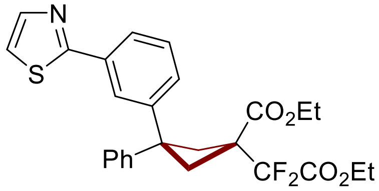


**Ethyl (1r,3r)-1-(2-ethoxy-1,1-difluoro-2-oxoethyl)-3-phenyl-3-(3-(thiazol-2-yl)phenyl)cyclobutane-1-carboxylate** (*major*-**45**, 53 mg, 36%) was obtained as a colorless oil. **$^1$H NMR** (400 MHz, $CDCl_3$) δ = 8.02 (d, *J* = 1.9 Hz, 1H), 7.83 (d, *J* = 3.2 Hz, 1H), 7.69 (dt, *J* = 7.7, 1.4 Hz, 1H), 7.41 (d, *J* = 8.1 Hz, 1H), 7.33 (t, *J* = 7.7 Hz, 1H), 7.29 (d, *J* = 3.3 Hz, 1H), 7.27 – 7.22 (m, 4H), 7.15 – 7.06 (m, 1H), 4.28 (q, *J* = 7.1 Hz, 2H), 3.90 (q, *J* = 7.1 Hz, 2H), 3.46 (s, 4H), 1.32 (t, *J* = 7.1 Hz, 3H), 0.89 (t, *J* = 7.1 Hz, 3H). **$^{13}$C NMR** (101 MHz, $CDCl_3$) δ = 170.8 ($C_q$), 168.5 ($C_q$), 163.1 (t, *J* = 32.8 Hz, $C_q$), 148.9 ($C_q$), 148.6 ($C_q$), 143.8 (CH), 133.8 ($C_q$), 129.2 (CH), 128.7 (CH), 128.3 (CH), 126.2 (CH), 125.5 (CH), 124.6 (CH), 124.4 (CH), 119.0 (CH), 114.2 (t, *J* = 253.7 Hz, $C_q$), 63.0 ($CH_2$), 62.0 ($CH_2$), 48.14 (t, *J* = 26.0 Hz, $C_q$), 45.5 ($C_q$), 38.63 (t, *J* = 3.8 Hz, $CH_2$), 14.0 ($CH_3$), 13.5 ($CH_3$). **$^{19}$F NMR** (376 MHz, $CDCl_3$) δ = -113.01. **IR** (ATR): 2986, 1771, 1723, 1311, 1136, 1029, 910, 731 $cm^{-1}$. **MS** (ESI) *m/z* (relative intensity): 486 $[M+H]^+$ (40), 508 $[M+Na]^+$ (100). **HR-MS** (ESI) *m/z* calcd for $C_{26}H_{25}NO_4F_2S$ $[M+Na]^+$: 508.1365, found: 508.1369.

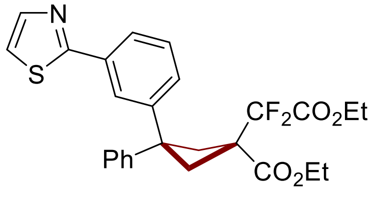


**Ethyl**

**(1s,3s)-1-(2-ethoxy-1,1-difluoro-2-oxoethyl)-3-phenyl-3-(3-(thiazol-2-yl)phenyl)cyclobutane-1-carboxylate** (*minor*-**45**, 27 mg, 19%) was obtained as a colorless oil. **$^1$H NMR** (400 MHz, $CDCl_3$) δ = 7.84 (t, *J* = 1.8 Hz, 1H), 7.81 (d, *J* = 3.3 Hz, 1H), 7.64 (d, *J* = 7.5 Hz, 1H), 7.40 – 7.35 (m, 2H), 7.34 – 7.19 (m, 5H), 7.09 (t, *J* = 7.4 Hz, 1H), 4.26 (q, *J* = 7.2 Hz, 2H), 3.88 (q, *J* = 7.2 Hz, 2H), 3.42 (s, 4H), 1.30 (t, *J* = 7.1 Hz, 3H), 0.89 (t, *J* = 7.1 Hz, 3H). **$^{13}$C NMR** (101 MHz, $CDCl_3$) δ = 170.8 ($C_q$) 168.4 ($C_q$), 163.1 ($C_q$), 150.5 ($C_q$), 146.9 ($C_q$), 143.8 (CH), 133.9 ($C_q$), 129.3 (CH), 128.6 (CH), 127.2 (CH), 126.4 (CH), 126.3 (CH), 124.6 (CH), 123.4 (CH), 119.0 (CH), 114.2 (t, *J* = 253.7 Hz, $C_q$), 63.0 ($CH_2$), 61.9 ($CH_2$), 48.3 (t, *J* = 26.0 Hz, $C_q$), 45.5 ($C_q$), 38.6 (t, *J* = 3.7 Hz, $CH_2$), 14.0 ($CH_3$), 13.6 ($CH_3$). **$^{19}$F NMR** (376 MHz, $CDCl_3$) δ = -112.97. **IR** (ATR): 2986, 1770, 1723, 1310, 1135, 1064, 1020, 700 $cm^{-1}$. **MS** (ESI) *m*/*z* (relative intensity): 486 $[M+H]^+$ (30), 508 $[M+Na]^+$ (100). **HR-MS** (ESI) *m*/*z* calcd for $C_{26}H_{25}NO_4F_2S$ $[M+H]^+$: 486.1345, found: 486.1346.

Compound **46** was obtained following the general procedure with **1h** (70 mg, 0.3 mmol), 2-phenylpyridine (140 mg, 0.9 mmol), and ethyl 2-bromo-2,2-difluoroacetate (183 mg, 0.9 mmol) for 24 h. Purification by column chromatography on silica gel (*n*-hexane/EtOAc = 10/1→5/1) yielded **46** (127 mg, 83%) with two separable diastereomers (*major*: *minor* = 5:1). **R$_f$** = 0.21 for *major*-**46** and 0.19 for *minor*-**46** (TLC: *n*-hexane/EtOAc = 3/1).

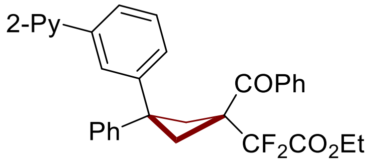


**Ethyl 2-((1r,3r)-1-benzoyl-3-phenyl-3-(3-(pyridin-2-yl)phenyl)cyclobutyl)-2,2-difluoroacetate** (*major*-**46**, 106 mg, 69%) was obtained as a colorless oil. **$^1$H NMR** (500 MHz, $CDCl_3$) δ = 8.58 (ddd, *J* = 4.9, 1.9, 0.9 Hz, 1H), 7.69 – 7.59 (m, 3H), 7.61 – 7.55 (m, 2H), 7.40 – 7.34 (m, 2H), 7.29 – 7.22 (m, 6H), 7.20 – 7.07 (m, 4H), 4.15 (q, *J* = 7.2 Hz, 2H), 3.71 – 3.64 (m, 4H), 1.21 (t, *J* = 7.1 Hz, 3H). **$^{13}$C NMR** (126 MHz, $CDCl_3$) δ

= 199.7 (t, $J$ = 2.6 Hz, $C_q$), 162.7 (t, $J$ = 32.5 Hz, $C_q$), 157.3 ($C_q$), 149.6 (CH), 148.5 ($C_q$), 147.2 ($C_q$), 139.5 ($C_q$), 136.7 (CH), 135.3 ($C_q$), 132.7 (CH), 129.1 (t, $J$ = 2.3 Hz, CH), 129.0 (CH), 128.6 (CH), 128.2 (CH), 127.3 (CH), 126.1 (CH), 125.7 (CH), 125.0 (CH), 124.9 (CH), 122.2 (CH), 120.7 (CH), 114.9 (t, $J$ = 256.0 Hz, $C_q$), 63.3 ($CH_2$), 53.4 (t, $J$ = 24.1 Hz, $C_q$), 45.6 ($C_q$), 40.9 (t, $J$ = 3.8 Hz, $CH_2$), 13.9 ($CH_3$). **$^{19}$F NMR** (471 MHz, $CDCl_3$) δ = -108.28. **IR** (ATR): 3059, 1763, 1673, 1469, 1312, 1131, 730, 697 $cm^{-1}$. **MS** (ESI) *m/z* (relative intensity): 512 $[M+H]^+$ (100), 534 $[M+Na]^+$ (85). **HR-MS** (ESI) *m/z* calcd for $C_{32}H_{27}NO_3F_2$ $[M+Na]^+$: 534.1851, found: 534.1845.

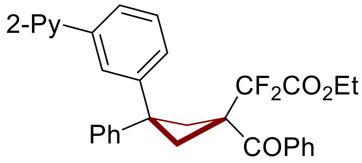


**Ethyl 2-((1s,3s)-1-benzoyl-3-phenyl-3-(3-(pyridin-2-yl)phenyl)cyclobutyl)-2,2-difluoroacetate** (*minor*-**46**, 21 mg, 14%) was obtained as a colorless oil. **$^1$H NMR** (500 MHz, $CDCl_3$) δ = 8.61 (ddd, $J$ = 4.8, 1.8, 0.9 Hz, 1H), 7.81 – 7.74 (m, 3H), 7.73 (t, $J$ = 1.9 Hz, 1H), 7.63 (td, $J$ = 7.7, 1.9 Hz, 1H), 7.48 – 7.41 (m, 1H), 7.41 – 7.29 (m, 6H), 7.31 – 7.21 (m, 3H), 7.19 – 7.16 (m, 2H), 4.09 – 3.99 (m, 4H), 3.24 (dd, $J$ = 13.1, 1.6 Hz, 2H), 1.11 (t, $J$ = 7.1 Hz, 3H). **$^{13}$C NMR** (126 MHz, $CDCl_3$) δ = 199.8 ($C_q$), δ 163.3 (t, $J$ = 33.4 Hz, $C_q$), 157.1 ($C_q$), 149.7 (CH), 143.6 ($C_q$), 140.3 ($C_q$), 139.3 ($C_q$), 136.7 (CH), 133.8 ($C_q$), 132.8 (CH), 123.0 (CH), 129.4 (CH), 128.6 (CH), 128.5 (CH), 128.3 (CH), 127.8 (CH), 126.5 (CH), 125.6 (CH), 124.6 (CH), 122.3 (CH), 120.7 (CH), 116.4 (t, $J$ = 256.1 Hz, $C_q$), 62.6 ($CH_2$), 50.9 ($C_q$), 45.3 (t, $J$ = 23.5 Hz, $C_q$), 39.4 (t, $J$ = 4.4 Hz, $CH_2$), 13.9 ($CH_3$). **$^{19}$F NMR** (471 MHz, $CDCl_3$) δ = -114.06. **IR** (ATR): 3061, 1760, 1678, 1589, 1314, 1136, 730, 698 $cm^{-1}$. **MS** (ESI) *m/z* (relative intensity): 512 $[M+H]^+$ (60), 534 $[M+Na]^+$ (100). **HR-MS** (ESI) *m/z* calcd for $C_{32}H_{27}NO_3F_2$ $[M+Na]^+$: 534.1851, found: 534.1857.

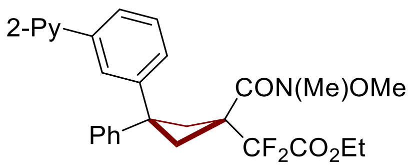

**Ethyl 2,2-difluoro-2-(1-(methoxy(methyl)carbamoyl)-3-phenyl-3-(3-(pyridin-2-yl)phenyl)cyclobutyl)acetate** (**47**). The product was obtained following the general procedure with **1o** (65 mg, 0.3 mmol), 2-phenylpyridine (140 mg, 0.9 mmol), and ethyl 2-bromo-2,2-difluoroacetate (183 mg, 0.9 mmol) for 24 h. Purification by column chromatography on silica gel (*n*-hexane/EtOAc = 10/1→3/1) yielded **47** (105 mg, 71%) as a colorless oil with two diastereomers (*major*: *minor* = 6:1). $\mathbf{R_f}$ = 0.17 (TLC: *n*-hexane/EtOAc = 2/1). **$^1$H NMR** (500 MHz, $CDCl_3$) δ = 8.68 – 8.61 (m, 1H, *major+minor*), 7.97 (t, *J* = 1.8 Hz, 0.84H, *major*), 7.79 – 7.63 (m, 3.13H, *major+minor*), 7.38 – 7.26 (m, 6H, *major+minor*), 7.23 – 7.11 (m, 2H, *major+minor*), 4.23 (q, *J* = 7.1 Hz, 1.73H, *major*), 4.08 (q, *J* = 7.2 Hz, 0.29H, *minor*), 3.63 – 3.51 (m, 6H, *major+minor*), 3.21 – 2.97 (m, 4H, *major+minor*), 1.31 (t, *J* = 7.2 Hz, 2.61H, *major*), 1.14 (t, *J* = 7.1 Hz, 0.40H, *minor*). **$^{13}$C NMR** (126 MHz, $CDCl_3$) δ = 169.7 ($C_q$, *major+minor*), 163.2 ($C_q$, *major+minor*), 157.5 ($C_q$, *major*), 157.2 ($C_q$, *minor*), 149.7 (CH, *major*), 149.7 (CH, *minor*), 149.0 ($C_q$, *major+minor*), 148.7 ($C_q$, *major+minor*), 139.7 ($C_q$, *minor*), 139.6 ($C_q$, *major*), 136.8 (CH, *major*), 136.66 (CH, *minor*), 129.0 (CH, *major*), 128.8 (CH, *minor*), 128.5 (CH, *major*), 128.4 (CH, *minor*), 128.2 (CH, *minor*), 127.6 (CH, *minor*), 127.1 (CH, *major*), 126.4 (CH, *minor*), 125.9 (CH, *major*), 125.8 (CH, *major*), 125.2 (CH, *minor*), 124.8 (CH, *major*), 124.7 (CH, *major*), 124.6 (CH, *minor*), 122.2 (CH, *major+minor*), 120.7 (CH, *major*), 120.7 (CH, *minor*), 116.9 (t, *J* = 255.8 Hz, $C_q$, *minor*), 114.8 (t, *J* = 255.8 Hz, $C_q$, *major*), 63.1 ($CH_2$, *major*), 62.5 ($CH_2$, *minor*), 60.8 ($CH_3$, *major*), 60.4 ($CH_3$, *minor*), 48.8 (t, *J* = 25.0 Hz, $C_q$, *major+minor*), 45.1 ($C_q$, *major+minor*), 39.3 (t, *J* = 3.8 Hz, $CH_2$, *major*), 38.2 ($CH_2$, *minor*), 14.0 ($CH_3$, *major*), 13.9 ($CH_3$, *minor*). **$^{19}$F NMR** (471 MHz, $CDCl_3$) δ = -111.32 (*major*), -114.00 (*minor*). **IR** (ATR): 2978, 1763, 1642, 1468, 1310, 1133, 995, 733 $cm^{-1}$. **MS** (ESI) *m/z* (relative intensity): 495 $[M+H]^+$ (70), 517 $[M+Na]^+$ (100).**HR-MS** (ESI) *m/z* calcd for $C_{28}H_{28}N_2O_4F_2$ $[M+Na]^+$: 517.1909, found:

517.1906.

Compound **48** was obtained following the general procedure with **1a** (56 mg, 0.3 mmol), 2-phenylpyridine (140 mg, 0.9 mmol), and 1,1,1,2,2,3,3,4,4-nonafluoro-4-iodobutane (311 mg, 0.9 mmol) for 24 h. Purification by column chromatography on silica gel (*n*-hexane/EtOAc/DCM = 30/1/1→20/1/1) yielded **48** (93 mg, 55%) with two separable diastereomers (*cis*: *trans* = 1.6:1). $\mathbf{R_f}$ = 0.30 for *cis*-**48** and 0.33 for *trans*-**48** (TLC: *n*-hexane/EtOAc/DCM = 10/1/1).

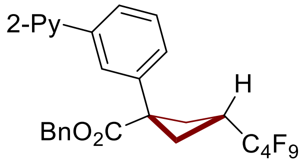


**Benzyl (1s,3s)-3-(perfluorobutyl)-1-(3-(pyridin-2-yl)phenyl)cyclobutane-1-carboxylate** (*cis*-**48**, 57 mg, 34%) was obtained as a colorless oil. **$^1$H NMR** (500 MHz, $CDCl_3$) δ = 8.71 (ddd, *J* = 4.9, 1.9, 1.0 Hz, 1H), 8.08 (dt, *J* = 2.5, 1.1 Hz, 1H), 7.96 – 7.90 (m, 1H), 7.76 (td, *J* = 7.7, 1.8 Hz, 1H), 7.68 (dt, *J* = 8.0, 1.1 Hz, 1H), 7.52 – 7.47 (m, 2H), 7.29 – 7.24 (m, 4H), 7.20 – 7.14 (m, 2H), 5.09 (s, 2H), 3.18 – 2.81 (m, 5H). **$^{13}$C NMR** (126 MHz, $CDCl_3$) δ = 174.0 ($C_q$), 157.1 ($C_q$), 149.9 (CH), 140.7 ($C_q$), 140.1 ($C_q$), 137.0 (CH), 135.8 ($C_q$), 129.3 (CH), 128.6 (CH), 128.3 (CH), 127.8 (CH), 127.3 (CH), 126.3 (CH), 125.5 (CH), 122.5 (CH), 120.9 (CH), 67.1 ($CH_2$), 48.0 ($C_q$), 31.2 (t, *J* = 5.2 Hz, $CH_2$), 29.7 (t, *J* = 24.8 Hz, CH). **$^{19}$F NMR** (471 MHz, $CDCl_3$) δ = -80.93 – -81.06 (m), -120.95 (q, *J* = 13.5 Hz), -123.80 (dt, *J* = 8.9, 4.3 Hz), -126.32 (td, *J* = 12.4, 3.9 Hz). **IR** (ATR): 1731, 1464, 1229, 1161, 1128, 773, 740, 694 $cm^{-1}$. **MS** (ESI) *m*/*z* (relative intensity): 562 $[M+H]^+$ (100), 584 $[M+Na]^+$ (80). **HR-MS** (ESI) *m*/*z* calcd for $C_{27}H_{20}NO_2F_9$ $[M+Na]^+$: 584.1243, found: 584.1244.

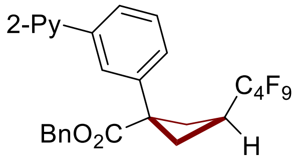

**Benzyl (1r,3r)-3-(perfluorobutyl)-1-(3-(pyridin-2-yl)phenyl)cyclobutane-1-carboxylate** (*trans*-**48**, 36 mg, 21%) was obtained as a colorless oil. $^1$**H NMR** (500 MHz, $CDCl_3$) δ = 8.70 (ddd, *J* = 4.8, 1.8, 1.0 Hz, 1H), 7.92 – 7.86 (m, 1H), 7.85 (t, *J* = 1.8 Hz, 1H), 7.79 – 7.71 (m, 1H), 7.68 (dt, *J* = 8.0, 1.1 Hz, 1H), 7.45 (t, *J* = 7.7 Hz, 1H), 7.30 – 7.23 (m, 5H), 7.20 – 7.14 (m, 2H), 5.13 (s, 2H), 3.31 – 3.04 (m, 3H), 2.84 – 2.75 (m, 2H). $^{13}$**C NMR** (126 MHz, $CDCl_3$) δ = 174.4 ($C_q$), 157.1 ($C_q$), 149.9 (CH), 143.0 ($C_q$), 139.9 ($C_q$), 136.9 (CH), 135.8 ($C_q$), 129.1 (CH), 128.6 (CH), 128.3 (CH), 127.7 (CH), 126.6 (CH), 126.0 (CH), 124.6 (CH), 122.5 (CH), 120.8 (CH), 67.3 ($CH_2$), 48.9 ($C_q$), 31.8 (t, *J* = 5.2 Hz, $CH_2$), 31.2 (t, *J* = 25.1 Hz, CH). $^{19}$**F NMR** (471 MHz, $CDCl_3$) δ = -80.95 – -81.06 (m), -121.00 (q, *J* = 13.3 Hz), -123.93 (dt, *J* = 9.2, 4.1 Hz), -126.34 (td, *J* = 12.3, 3.8 Hz). **IR** (ATR): 1730, 1463, 1231, 1167, 1131, 741, 693 $cm^{-1}$. **MS** (ESI) *m*/*z* (relative intensity): 562 $[M+H]^+$ (100), 584 $[M+Na]^+$ (65). **HR-MS** (ESI) *m*/*z* calcd for $C_{27}H_{20}NO_2F_9$ $[M+Na]^+$: 584.1243, found: 584.1249.

Compound **49** was obtained following the general procedure **1a** (56 mg, 0.3 mmol), 2-phenylpyridine (140 mg, 0.9 mmol), and 1,1,1,2,2,3,3,4,4,5,5,6,6-tridecafluoro-6-iodohexane (401 mg, 0.9 mmol) for 24 h. Purification by column chromatography on silica gel (*n*-hexane/EtOAc/DCM = 30/1/1 →20/1/1) yielded **49** (113 mg, 57%) with two separable diastereomers (*cis*: *trans* = 2.1:1). **R**$_f$ = 0.30 for *cis*-**49** and 0.33 for *trans*-**49** (TLC: *n*-hexane/EtOAc/DCM = 10/1/1).

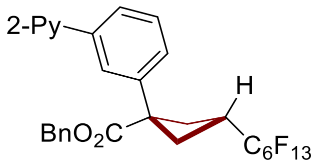


**Benzyl (1s,3s)-3-(perfluorohexyl)-1-(3-(pyridin-2-yl)phenyl)cyclobutane-1-carboxylate** (*cis*-**49**, 76 mg, 38%) was obtained as a colorless oil. $^1$**H NMR** (500 MHz, $CDCl_3$) δ = 8.71 (ddd, *J* = 4.8, 1.8, 0.9 Hz, 1H), 8.08 (t, *J* = 1.8 Hz, 1H), 7.96 – 7.90 (m, 1H), 7.76

(td, *J* = 7.7, 1.8 Hz, 1H), 7.68 (dt, *J* = 8.0, 1.1 Hz, 1H), 7.54 – 7.47 (m, 2H), 7.30 – 7.23 (m, 4H), 7.21 – 7.14 (m, 2H), 5.09 (s, 2H), 3.18 – 3.09 (m, 2H), 3.05 – 2.81 (m, 3H). **$^{13}$C NMR** (126 MHz, $CDCl_3$) δ = 174.0 ($C_q$), 157.1 ($C_q$), 149.9 (CH), 140.7 ($C_q$), 140.1 ($C_q$), 136.9 (CH), 135.8 ($C_q$), 129.3 (CH), 128.6 (CH), 128.3 (CH), 127.8 (CH), 127.30 (CH), 126.3 (CH), 125.5 (CH), 122.5 (CH), 120.9 (CH), 67.1 ($CH_2$), 48.0 ($C_q$), 31.1 (t, *J* = 5.1 Hz, $CH_2$), 29.8 (t, *J* = 25.0 Hz, CH). **$^{19}$F NMR** (471 MHz, $CDCl_3$) δ = -80.79, -120.73 (q, *J* = 15.2, 14.5 Hz), -122.20 (t, *J* = 14.6 Hz), -122.86, -126.15 (td, *J* = 15.1, 14.3, 5.6 Hz). **IR** (ATR): 1731, 1236, 1199, 1139, 909, 732, 699 $cm^{-1}$. **MS** (ESI) *m*/*z* (relative intensity): 662 $[M+H]^+$ (100), 684 $[M+Na]^+$ (95). **HR-MS** (ESI) *m*/*z* calcd for $C_{29}H_{20}NO_2F_{13}$ $[M+Na]^+$: 684.1179, found: 684.1187.

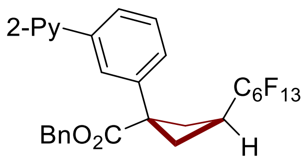


**Benzyl (1r,3r)-3-(perfluorohexyl)-1-(3-(pyridin-2-yl)phenyl)cyclobutane-1-carboxylate**

(*trans*-**49**, 37 mg, 19%) was obtained as a colorless oil. **$^{1}$H NMR** (500 MHz, $CDCl_3$) δ = 8.70 (ddd, *J* = 4.8, 1.8, 1.0 Hz, 1H), 7.89 (dt, *J* = 7.8, 1.4 Hz, 1H), 7.85 (t, *J* = 1.8 Hz, 1H), 7.75 (td, *J* = 7.7, 1.8 Hz, 1H), 7.68 (dt, *J* = 8.0, 1.1 Hz, 1H), 7.45 (t, *J* = 7.7 Hz, 1H), 7.29 – 7.23 (m, 5H), 7.19 – 7.15 (m, 2H), 5.13 (s, 2H), 3.30 – 3.04 (m, 3H), 2.84 – 2.75 (m, 2H). **$^{13}$C NMR** (126 MHz, $CDCl_3$) δ = 174.4 ($C_q$), 157.1 ($C_q$), 149.9 (CH), 143.0 ($C_q$), 139.9 ($C_q$), 136.9 (CH), 135.8 ($C_q$), 129.1 (CH), 128.6 (CH), 128.3 (CH), 127.7 (CH), 126.6 (CH), 126.0 (CH), 124.6 (CH), 122.5 (CH), 120.9 (CH), 67.3 ($CH_2$), 48.9 ($C_q$), 31.8 (t, *J* = 5.1 Hz, $CH_2$), 31.3 (t, *J* = 25.0 Hz, CH). **$^{19}$F NMR** (471 MHz, $CDCl_3$) δ = -80.77 (t, *J* = 9.7 Hz), -120.76 (qd, *J* = 14.3, 4.0 Hz), -122.20 (t, *J* = 13.6 Hz), -122.94 (d, *J* = 67.5 Hz), -126.13 (td, *J* = 14.9, 6.6 Hz). **IR** (ATR): 1730, 1462, 1237, 1145, 1138, 734, 700 $cm^{-1}$. **MS** (ESI) *m*/*z* (relative intensity): 662 $[M+H]^+$ (100), 684 $[M+Na]^+$ (70). **HR-MS** (ESI) *m*/*z* calcd for $C_{29}H_{20}NO_2F_{13}$ $[M+Na]^+$: 684.1179, found: 684.1174.

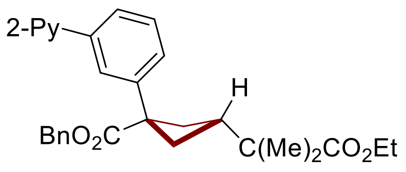


**Benzyl 3-(1-ethoxy-2-methyl-1-oxopropan-2-yl)-1-(3-(pyridin-2-yl)phenyl)cyclobutane-1-carboxylate** (**50**). The product was obtained following the general procedure with **1a** (56 mg, 0.3 mmol), 2-phenylpyridine (140 mg, 0.9 mmol), and ethyl 2-bromo-2-methylpropanoate (176 mg, 0.9 mmol) for 24 h. Purification by column chromatography on silica gel (*n*-hexane/EtOAc = 10/1→5/1) yielded **50** (38 mg, 28%) as a colorless oil with two diastereomers (*cis*: *trans* = 2.9:1). $\mathbf{R_f}$ = 0.34 (TLC: *n*-hexane/EtOAc = 3/1). **$^1$H NMR** (500 MHz, $CDCl_3$) δ = 8.71 – 8.68 (m, 1H, *cis*+*trans*), 8.07 (t, *J* = 1.7 Hz, 0.68H, *cis*), 7.93 – 7.82 (m, 1.33H, *cis*+*trans*), 7.76 – 7.71 (m, 1H, *cis*+*trans*), 7.68 – 7.65 (m, 1H, *cis*+*trans*), 7.53 – 7.50 (m, 0.71H, *cis*), 7.49 – 7.45 (m, 0.72H, *cis*), 7.43 – 7.39 (m, 0.44H, *trans*), 7.31 – 7.28 (m, 0.41H, *trans*), 7.26 – 7.21 (m, 4H, *cis*+*trans*), 7.19 – 7.14 (m, 2H, *cis*+*trans*), 5.10 (s, 0.46H, *trans*), 5.05 (s, 1.33H, *cis*), 4.12 – 4.00 (m, 2H, *cis*+*trans*), 2.95 – 2.37 (m, 5H, *cis*+*trans*), 1.22 (t, *J* = 7.1 Hz, 2.44H, *cis*), 1.15 (t, *J* = 7.1 Hz, 0.96H, *trans*), 1.12 – 1.06 (m, 6H, *cis*+*trans*). **$^{13}$C NMR** (126 MHz, $CDCl_3$) δ = 177.0 ($C_q$, *cis*), 177.0 ($C_q$, *trans*), 175.5 ($C_q$, *trans*), 175.2 ($C_q$, *cis*), 157.5 ($C_q$, *cis)*, 157.4 ($C_q$, *trans*), 149.8 (CH, *cis*), 149.8 (CH, *trans*), 144.5 ($C_q$, *trans*), 142.1 ($C_q$, *cis*), 139.8 ($C_q$, *cis*), 137.6 ($C_q$, *trans*), 136.8 (CH, *cis*), 136.8 (CH, *trans*), 136.2 ($C_q$, *trans*), 136.2 ($C_q$, *cis*), 128.9 (CH, *cis*), 128.8 (CH, *trans*), 128.5 (CH), 128.1 (CH), 128.0 (CH), 127.8 (CH), 127.7 (CH), 127.5 (CH), 126.9 (CH), 125.9 (CH, *cis*), 125.8 (CH, *cis*), 125.5 (CH, *trans*), 124.9 (CH, *trans*), 122.3 (CH, *cis*), 122.3 (CH, *trans*), 120.9 (CH, *cis*), 120.8 (CH, *trans*), 66.8 ($CH_2$, *trans*), 66.6 ($CH_2$, *cis*), 60.5 ($CH_2$, *cis*+*trans*), 48.3 ($C_q$, *trans*), 47.0 ($C_q$, *cis*), 43.3 ($C_q$, *trans*), 43.2 ($C_q$, cis), 38.7 (CH, *trans*), 36.8 (CH, *cis*), 34.2 ($CH_2$, *trans*), 33.4 ($CH_2$, *cis*), 21.9 ($CH_3$, *trans*), 21.8 ($CH_3$, *cis*), 14.4 ($CH_3$, *cis*), 14.3 ($CH_3$, *trans*). **IR** (ATR): 2980, 1722, 1466, 1272, 1138, 1025, 775, 744, 699 $cm^{-1}$. **MS** (ESI) *m*/*z* (relative intensity): 458 $[M+H]^+$ (40), 480 $[M+Na]^+$ (100). **HR-MS** (ESI) *m*/*z* calcd for $C_{29}H_{31}NO_4$ $[M+H]^+$: 458.2326, found: 458.2326.

Compound **51** was obtained following the general procedure with **1a** (56 mg, 0.3 mmol), 2-phenylpyridine (140 mg, 0.9 mmol), and ethyl 2-bromopropanoate (163 mg, 0.9 mmol) for 24 h. Purification by column chromatography on silica gel (*n*-hexane/EtOAc/DCM = 20/1/1 → 10/1/1) yielded **51** (54 mg, 41%) with two separable diastereomers (*cis*: *trans* = 1.8:1). **R**$_f$ = 0.33 for *cis*-**51** and 0.36 for *trans*-**51** (TLC: *n*-hexane/EtOAc/DCM = 5/1/1). Note: product **51** was obtained from a prochiral radical species, only two isomers were observed in this case, i.e. *cis*-**51** and *trans*-**51**.

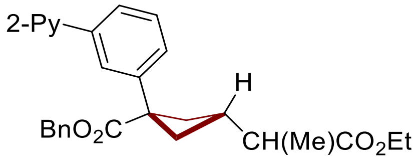


**Benzyl (1s,3s)-3-(1-ethoxy-1-oxopropan-2-yl)-1-(3-(pyridin-2-yl)phenyl)cyclobutane-1-carboxylate** (*cis*-**51**, 35 mg, 26%) as a colorless oil. **$^1$H NMR** (500 MHz, $CDCl_3$) δ = 8.70 (ddd, *J* = 4.8, 1.8, 1.0 Hz, 1H), 8.04 (td, *J* = 1.8, 0.7 Hz, 1H), 7.90 (dt, *J* = 6.8, 1.8 Hz, 1H), 7.77 – 7.70 (m, 1H), 7.67 (dt, *J* = 8.0, 1.1 Hz, 1H), 7.50 – 7.44 (m, 2H), 7.26 – 7.21 (m, 4H), 7.19 – 7.14 (m, 2H), 5.06 (s, 2H), 4.10 (qd, *J* = 7.1, 1.6 Hz, 2H), 2.84 – 2.75 (m, 2H), 2.70 – 2.62 (m, 1H), 2.61 – 2.54 (m, 1H), 2.48 – 2.41 (m, 1H), 2.39 – 2.30 (m, 1H), 1.24 (t, *J* = 7.1 Hz, 3H), 1.07 (d, *J* = 6.9 Hz, 3H). **$^{13}$C NMR** (126 MHz, $CDCl_3$) δ = 175.4 ($C_q$), 175.3 ($C_q$), 157.4 ($C_q$), 149.8 (CH), 142.2 ($C_q$), 139.8 ($C_q$), 136.8 (CH), 136.1 ($C_q$), 129.0 (CH), 128.5 (CH), 128.1 (CH), 127.7 (CH), 127.6 (CH), 125.8 (CH), 125.7 (CH), 122.3 (CH), 120.9 (CH), 66.7 ($CH_2$), 60.4 ($CH_2$), 48.1 ($C_q$), 45.2 (CH), 36.7 ($CH_2$), 36.2 ($CH_2$), 32.4 (CH), 14.5 ($CH_3$), 14.4 ($CH_3$). **IR** (ATR): 2980, 1726, 1589, 1462, 1166, 1134, 775, 699 $cm^{-1}$. **MS** (ESI) *m*/*z* (relative intensity): 444 $[M+H]^+$ (70), 466 $[M+Na]^+$ (100). **HR-MS** (ESI) *m*/*z* calcd for $C_{28}H_{29}NO_4$ $[M+Na]^+$: 466.1989, found: 466.1995.

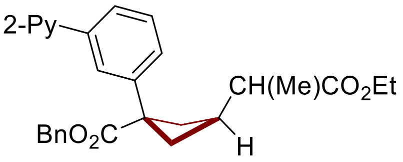


**Benzyl (1r,3r)-3-(1-ethoxy-1-oxopropan-2-yl)-1-(3-(pyridin-2-yl)phenyl)cyclobutane-1-carboxylate** (*trans*-**51**, 19 mg, 14%) as a colorless oil. **$^1$H NMR** (500 MHz, $CDCl_3$) δ = 8.70 (ddd, *J* = 4.8, 1.8, 1.0 Hz, 1H), 7.87 – 7.83 (m, 2H), 7.77 – 7.70 (m, 1H), 7.67 (dt, *J* = 8.0, 1.1 Hz, 1H), 7.42 (td, *J* = 7.6, 0.8 Hz, 1H), 7.31 – 7.28 (m, 1H), 7.26 – 7.21 (m, 4H), 7.19 – 7.14 (m, 2H), 5.10 (s, 2H), 4.10 (qd, *J* = 7.1, 1.8 Hz, 2H), 3.16 – 3.02 (m, 2H), 2.62 – 2.52 (m, 1H), 2.43 – 2.35 (m, 1H), 2.36 – 2.27 (m, 1H), 2.26 – 2.20 (m, 1H), 1.22 (t, *J* = 7.1 Hz, 3H), 1.07 (d, *J* = 6.9 Hz, 3H). **$^{13}$C NMR** (126 MHz, $CDCl_3$) δ = 175.3 ($C_q$), 175.3 ($C_q$), 157.4 ($C_q$), 149.8 (CH), 144.6 ($C_q$), 139.7 ($C_q$), 136.8 (CH), 136.2 ($C_q$), 128.9 (CH), 128.5 (CH), 128.0 (CH), 127.6 (CH), 126.9 (CH), 125.5 (CH), 124.9 (CH), 122.3 (CH), 120.9 (CH), 66.9 ($CH_2$), 60.4 ($CH_2$), 49.1 ($C_q$), 45.8 (CH), 37.7 ($CH_2$), 37.2 ($CH_2$), 34.0 (CH), 14.4 ($CH_3$), 14.3 ($CH_3$). **IR** (ATR): 2980, 1724, 1588, 1457, 1166, 1131, 776, 698 $cm^{-1}$. **MS** (ESI) *m*/*z* (relative intensity): 444 $[M+H]^+$ (100), 466 $[M+Na]^+$ (95). **HR-MS** (ESI) *m*/*z* calcd for $C_{28}H_{29}NO_4$ $[M+H]^+$: 444.2169, found: 444.2174.

Compound **52** was obtained following the general procedure with **1a** (56 mg, 0.3 mmol), 2-phenylpyridine (140 mg, 0.9 mmol), and ethyl 2-bromoacetate (150 mg, 0.9 mmol) for 24 h. Purification by column chromatography on silica gel (*n*-hexane/EtOAc/DCM = 20/1/1 → 10/1/1) yielded **52** (74 mg, 57%) with two separable diastereomers (*cis*: *trans* = 1.5:1). **R**$_f$ = 0.30 for *cis*-**52** and 0.32 for *trans*-**52** (TLC: *n*-hexane/EtOAc/DCM = 5/1/1).

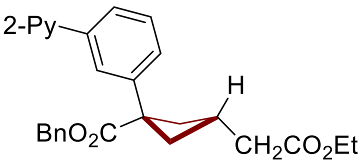


**Benzyl**

**(1s,3s)-3-(2-ethoxy-2-oxoethyl)-1-(3-(pyridin-2-yl)phenyl)cyclobutane-1-carboxylate** (*cis*-**52**, 44 mg, 34%) was obtained as a colorless oil. **$^{1}$H NMR** (400 MHz, $CDCl_3$) δ = 8.70 (ddd, *J* = 4.9, 1.9, 1.0 Hz, 1H), 8.03 (s, 1H), 7.92 – 7.88 (m, 1H), 7.73 (dd, *J* = 7.5, 1.8 Hz, 1H), 7.67 (dt, *J* = 8.0, 1.2 Hz, 1H), 7.48 – 7.41 (m, 2H), 7.27 – 7.21 (m, 4H), 7.20 – 7.15 (m, 2H), 5.07 (s, 2H), 4.10 (q, *J* = 7.2 Hz, 2H), 2.93 – 2.82 (m, 2H), 2.67 – 2.57 (m, 3H), 2.50 – 2.43 (m, 2H), 1.24 (t, *J* = 7.1 Hz, 3H). **$^{13}$C NMR** (101 MHz, $CDCl_3$) δ = 175.4 ($C_q$), 172.3 ($C_q$), 157.4 ($C_q$), 149.8 (CH), 142.4 ($C_q$), 139.8 ($C_q$), 136.8 (CH), 136.1 ($C_q$), 129.0 (CH), 128.5 (CH), 128.1 (CH), 127.7 (CH), 127.5 (CH), 125.7 (CH), 125.6 (CH), 122.3 (CH), 120.9 (CH), 66.7 ($CH_2$), 60.4 ($CH_2$), 48.8 ($C_q$), 40.6 ($CH_2$), 37.7 ($CH_2$), 25.8 (CH), 14.4 ($CH_3$). **IR** (ATR): 2983, 1725, 1588, 1464, 1131, 1025, 742, 700 $cm^{-1}$. **MS** (ESI) *m*/*z* (relative intensity): 430 $[M+H]^+$ (100), 452 $[M+Na]^+$ (70). **HR-MS** (ESI) *m*/*z* calcd for $C_{27}H_{27}NO_4$ $[M+H]^+$: 430.2013, found: 430.2015.

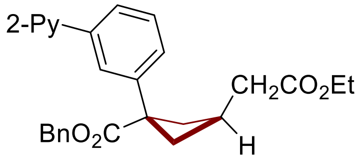


**Benzyl (1r,3r)-3-(2-ethoxy-2-oxoethyl)-1-(3-(pyridin-2-yl)phenyl)cyclobutane-1-carboxylate** (*trans*-**52**, 30 mg, 23%) was obtained as a colorless oil. **$^{1}$H NMR** (400 MHz, $CDCl_3$) δ = 8.69 (ddd, *J* = 5.0, 1.9, 1.0 Hz, 1H), 7.88 – 7.82 (m, 2H), 7.76 – 7.64 (m, 2H), 7.42 (td, *J* = 7.5, 0.9 Hz, 1H), 7.31 – 7.21 (m, 5H), 7.20 – 7.14 (m, 2H), 5.10 (s, 2H), 4.10 (q, *J* = 7.1 Hz, 2H), 3.23 – 3.12 (m, 2H), 2.90 – 2.77 (m, 1H), 2.43 – 2.26 (m, 4H), 1.22 (t, *J* = 7.1 Hz, 3H). **$^{13}$C NMR** (101 MHz, $CDCl_3$) δ = 175.3 ($C_q$), 172.2 ($C_q$), 157.4 ($C_q$), 149.8 (CH), 144.6 ($C_q$), 139.7 ($C_q$), 136.8 (CH), 136.2 ($C_q$), 128.9 (CH), 128.5 (CH), 128.0 (CH), 127.6 (CH), 127.0 (CH), 125.5 (CH), 124.9 (CH), 122.3 (CH), 120.8 (CH), 66.9 ($CH_2$), 60.4 ($CH_2$), 49.8 ($C_q$), 41.2 ($CH_2$), 38.7 ($CH_2$), 27.3 (CH), 14.4 ($CH_3$). **IR** (ATR): 2982, 1725, 1588, 1457, 1144, 1028, 738, 698 $cm^{-1}$. **MS** (ESI) *m*/*z* (relative intensity): 430 $[M+H]^+$ (100), 452 $[M+Na]^+$ (95). **HR-MS** (ESI) *m*/*z* calcd for $C_{27}H_{27}NO_4$ $[M+H]^+$: 430.2013, found: 430.2029.

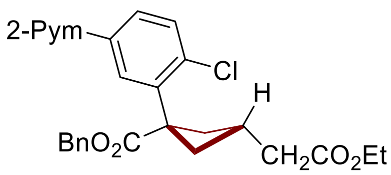


**Benzyl 1-(2-chloro-5-(pyrimidin-2-yl)phenyl)-3-(2-ethoxy-2-oxoethyl)cyclobutane-1-carboxylate** (**53**). The product was obtained following the general procedure with **1a** (56 mg, 0.3 mmol), 2-(4-chlorophenyl)pyrimidine (171 mg, 0.9 mmol), and ethyl 2-bromoacetate (150 mg, 0.9 mmol) for 46 h. Purification by column chromatography on silica gel (*n*-hexane/EtOAc = 10/1→3/1) yielded **53** (67 mg, 48%) as a colorless oil with two diastereomers (*cis*: *trans* = 1.3:1). $\mathbf{R_f}$ = 0.27 (TLC: *n*-hexane/EtOAc = 3/1). **$^1$H NMR** (500 MHz, $CDCl_3$) δ = 8.81 – 8.78 (m, 2H, *cis*+*trans*), 8.67 (d, $J$ = 2.1 Hz, 0.54H, *cis*), 8.42 (d, $J$ = 2.1 Hz, 0.34H, *trans*), 8.34 (dd, $J$ = 8.3, 2.1 Hz, 0.59H, *cis*), 8.29 (dd, $J$ = 8.3, 2.1 Hz, 0.37H, *trans*), 7.48 (d, $J$ = 8.4 Hz, 0.58H, *cis*), 7.43 (d, $J$ = 8.3 Hz, 0.37H, *trans*), 7.27 – 7.22 (m, 3H, *cis*+*trans*), 7.21 – 7.15 (m, 3H, *cis*+*trans*), 5.14 (s, 0.85H, *trans*), 5.10 (s, 1.11H, *cis*), 4.13 – 4.08 (m, 2H, *cis*+*trans*), 3.19 – 3.02 (m, 1H, *cis*+*trans*), 2.91 – 2.85 (m, 1H, *cis*+*trans*), 2.73 – 2.56 (m, 3H, *cis*+*trans*), 2.46 – 2.34 (m, 2H, *cis*+*trans*), 1.26 – 1.21 (m, 3H, *cis*+*trans*). **$^{13}$C NMR** (126 MHz, $CDCl_3$) δ = 174.8 ($C_q$, *cis*), 174.6 ($C_q$, *trans*), 172.5 ($C_q$, *cis*), 172.2 ($C_q$, *trans*), 163.9 ($C_q$, *cis*), 163.9 ($C_q$, *trans*), 157.4 (CH, *cis*+*trans*), 141.8 ($C_q$, *trans*), 139.8 ($C_q$, *cis*), 137.3 ($C_q$, *cis*+*trans*), 136.2 ($C_q$, *trans*), 136.2 ($C_q$, *cis*), 136.2 ($C_q$, *trans*), 136.0 ($C_q$, *cis*), 130.6 (CH, *cis*), 130.3 (CH, *trans*), 128.5 (CH, *trans*), 128.5 (CH, *cis*), 128.3 (CH, *trans*), 128.1 (CH, *cis*), 128.0 (CH, *cis*), 128.0 (CH, *trans*), 128.0 (CH, *trans*), 127.8 (CH, *cis*), 127.6 (CH, *cis*), 127.5 (CH, *trans*), 119.4 (CH, *cis*+*trans*), 67.0 ($CH_2$, *cis*), 66.9 ($CH_2$, *trans*), 60.4 ($CH_2$, *trans*), 60.4 ($CH_2$, *cis*), 49.7 ($C_q$, *trans*), 49.0 ($C_q$, *cis*), 41.4 ($CH_2$, *trans*), 40.4 ($CH_2$, *cis*), 38.4 ($CH_2$, *trans*), 36.4 ($CH_2$, *cis*), 26.7 (CH, *trans*), 25.7 (CH, *cis*), 14.4 ($CH_3$, *cis*), 14.4 ($CH_3$, *trans*). **IR** (ATR): 2982, 1726, 1560, 1418, 1181, 1137, 1028, 803, 701 $cm^{-1}$. **MS** (ESI) *m*/*z* (relative intensity): 465 $[M+H]^+$ (5), 487 $[M+Na]^+$ (100). **HR-MS** (ESI) *m*/*z* calcd for

$C_{26}H_{25}N_2O_4Cl$ [M+Na]$^+$: 487.1395, found: 487.1398.

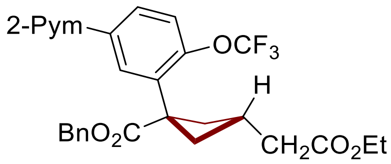


**Benzyl 3-(2-ethoxy-2-oxoethyl)-1-(5-(pyrimidin-2-yl)-2-(trifluoromethoxy)phenyl)cyclobutane-1-carboxylate** (**54**). The product was obtained following the general procedure with **1a** (56 mg, 0.3 mmol), 2-(4-(trifluoromethoxy)phenyl)pyrimidine (216 mg, 0.9 mmol), and ethyl 2-bromoacetate (150 mg, 0.9 mmol) for 24 h. Purification by column chromatography on silica gel (*n*-hexane/EtOAc = 10/1→3/1) yielded **54** (90 mg, 58%) as a colorless oil with two diastereomers (*cis*: *trans* = 1.6:1). $\mathbf{R_f}$ = 0.30 (TLC: *n*-hexane/EtOAc = 3/1). **$^1$H NMR** (500 MHz, $CDCl_3$) δ = 8.81 – 8.78 (m, 2H, *cis*+*trans*), 8.67 (d, $J$ = 2.2 Hz, 0.50H), 8.45 – 8.38 (m, 1.41H, *cis*+*trans*), 7.35 – 7.24 (m, 4H, *cis*+*trans*), 7.21 – 7.17 (m, 3H, *cis*+*trans*), 5.12 (s, 0.73H, *trans*), 5.09 (s, 1.19H, *cis*), 4.13 – 4.08 (m, 2H, *cis*+*trans*), 3.15 – 3.00 (m, 1H, *cis*+*trans*), 2.86 – 2.80 (m, 1H, *cis*+*trans*), 2.74 – 2.65 (m, 2H, *cis*+*trans*), 2.59 – 2.55 (m, 1H, *cis*+*trans*), 2.42 – 2.31 (m, 2H, *cis*+*trans*), 1.25 – 1.21 (m, 3H, *cis*+*trans*). **$^{13}$C NMR** (126 MHz, $CDCl_3$) δ = 174.6 ($C_q$, *cis*), 172.4 ($C_q$, *trans*), 172.4 ($C_q$, *cis*), 172.1 ($C_q$, *trans*), 163.6 ($C_q$, *cis*), 163.4 ($C_q$, *trans*), 157.4 (CH, *cis*+*trans*), 150.0 ($C_q$, *cis*), 149.4 ($C_q$, *trans*), 136.1 ($C_q$, *trans*), 136.0 ($C_q$, *cis*), 135.4 ($C_q$, *trans*), 135.4 ($C_q$, *cis*), 135.2 ($C_q$, *cis*), 133.5 ($C_q$, *trans*), 128.7 (CH), 128.6 (CH), 128.5 (CH), 128.1 (CH), 128.0 (CH), 128.0 (CH), 127.9 (CH), 127.7 (CH), 119.4 (CH, *cis*+*trans*), 118.3 (d, $J$ = 2.2 Hz, CH, *trans*), 118.0 (d, $J$ = 2.4 Hz, CH, *cis*), 66.9 ($CH_2$, *cis*), 66.9 ($CH_2$, *trans*), 60.4 ($CH_2$, *trans*), 60.4 ($CH_2$, *cis*), 47.5 ($C_q$, *trans*), 46.7 ($C_q$, *cis*), 41.3 ($CH_2$, *trans*), 40.3 ($CH_2$, *cis*), 38.4 ($CH_2$, *trans*), 36.3 ($CH_2$, *cis*), 27.3 (CH, *trans*), 25.7 (CH, *cis*), 14.4 ($CH_3$, *cis*), 14.3 ($CH_3$, *trans*). **$^{19}$F NMR** (471 MHz, $CDCl_3$) δ = -55.97 (d, $J$ = 1.8 Hz, *trans*), -55.98 (d, $J$ = 1.9 Hz, *cis*). **IR** (ATR): 2984, 1728, 1563, 1424, 1252, 1128, 1025, 804, 703 cm$^{-1}$. **MS** (ESI) *m*/*z* (relative intensity): 515 [M+H]$^+$ (10), 537 [M+Na]$^+$ (100). **HR-MS** (ESI) *m*/*z* calcd for $C_{27}H_{25}N_2O_5F_3$ [M+Na]$^+$: 537.1608,

found: 537.1609.

Compound **55** was obtained following the general procedure with **1a** (56 mg, 0.3 mmol), 2-phenylpyridine (140 mg, 0.9 mmol), and ethyl 2-bromo-2-fluoroacetate (166 mg, 0.9 mmol) for 24 h. Purification by column chromatography on silica gel (*n*-hexane/EtOAc = 10/1 → 5/1) yielded **55** (48 mg, 36%) with two separable diastereomers (*cis*: *trans* = 3:1). **R**$_f$ = 0.22 for *cis*-**55** and 0.25 for *trans*-**55** (TLC: *n*-hexane/EtOAc = 3/1). Note: product **55** was obtained from a prochiral radical species, only two isomers were observed in this case, i.e. *cis*-**55** and *trans*-**55**.

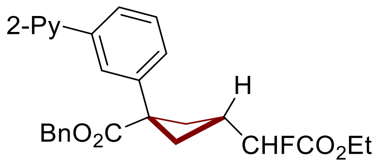


**Benzyl (1s,3s)-3-(2-ethoxy-1-fluoro-2-oxoethyl)-1-(3-(pyridin-2-yl)phenyl)cyclobutane-1-carboxylate** (*cis*-**55**, 36 mg, 27%) was obtained as a colorless oil. **$^1$H NMR** (500 MHz, $CDCl_3$) δ = 8.70 (ddd, *J* = 4.8, 1.8, 0.9 Hz, 1H), 8.05 (dt, *J* = 1.8, 1.1 Hz, 1H), 7.93 – 7.89 (m, 1H), 7.75 (td, *J* = 7.7, 1.8 Hz, 1H), 7.67 (dt, *J* = 8.0, 1.1 Hz, 1H), 7.49 – 7.44 (m, 2H), 7.27 – 7.23 (m, 4H), 7.20 – 7.16 (m, 2H), 5.08 (s, 2H), 4.93 – 4.78 (m, 1H), 4.22 (qd, *J* = 7.1, 3.7 Hz, 2H), 3.04 – 2.94 (m, 2H), 2.84 – 2.64 (m, 3H), 1.28 (t, *J* = 7.1 Hz, 3H). **$^{13}$C NMR** (126 MHz, $CDCl_3$) δ = 174.7 ($C_q$), 168.8 (d, *J* = 24.4 Hz, $C_q$), 157.2 ($C_q$), 149.7 (CH), 141.9 ($C_q$), 139.7 ($C_q$), 137.0 (CH), 136.0 ($C_q$), 129.1 (CH), 128.6 (CH), 128.5 (CH), 128.1 (CH), 127.8 (CH), 127.5 (CH), 126.0 (CH), 125.6 (CH), 122.4 (CH), 120.9 (CH), 89.8 (d, *J* = 185.7 Hz, CH), 66.8 ($CH_2$), 61.7 ($CH_2$), 48.2 ($C_q$), 33.1 (d, *J* = 5.9 Hz, $CH_2$), 32.5 (d, *J* = 4.3 Hz, $CH_2$), 31.0 (d, *J* = 22.7 Hz, CH), 14.3 ($CH_3$). **$^{19}$F NMR** (282 MHz, $CDCl_3$) δ = -199.10. **IR** (ATR): 2955, 1728, 1589, 1465, 1267, 1162, 1024, 775, 744 $cm^{-1}$. **MS** (ESI) *m*/*z* (relative intensity): 448 $[M+H]^+$ (30), 470 $[M+Na]^+$ (100). **HR-MS** (ESI) *m*/*z* calcd for $C_{27}H_{26}NO_4F$ $[M+H]^+$: 448.1919, found: 448.1920.

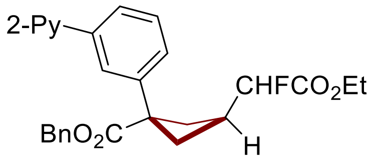


**Benzyl (1r,3r)-3-(2-ethoxy-1-fluoro-2-oxoethyl)-1-(3-(pyridin-2-yl)phenyl)cyclobutane-1-carboxylate** (*trans*-**55**, 12 mg, 9%) was obtained as a colorless oil. **$^{1}$H NMR** (500 MHz, $CDCl_3$) δ = 8.70 (ddd, *J* = 4.8, 1.9, 1.0 Hz, 1H), 7.89 – 7.86 (m, 1H), 7.85 (t, *J* = 1.9 Hz, 1H), 7.75 (ddd, *J* = 8.0, 7.4, 1.8 Hz, 1H), 7.67 (dt, *J* = 8.0, 1.1 Hz, 1H), 7.43 (t, *J* = 8.0 Hz, 1H), 7.31 – 7.28 (m, 1H), 7.26 – 7.23 (m, 4H), 7.19 – 7.15 (m, 2H), 5.11 (s, 2H), 4.83 – 4.71 (m, 1H), 4.22 (q, *J* = 7.1 Hz, 2H), 3.08 – 2.93 (m, 3H), 2.72 – 2.62 (m, 2H), 1.28 (t, *J* = 7.1 Hz, 3H). **$^{13}$C NMR** (126 MHz, $CDCl_3$) δ = 175.0 ($C_q$), 168.8 (d, *J* = 24.5 Hz, $C_q$), 157.2 ($C_q$), 149.7 (CH), 143.8 ($C_q$), 139.5 ($C_q$), 137.0 (CH), 136.0 ($C_q$), 129.0 (CH), 128.6 (CH), 128.2 (CH), 127.7 (CH), 126.9 (CH), 125.7 (CH), 124.9 (CH), 122.4 (CH), 120.9 (CH), 89.5 (d, *J* = 185.7 Hz, CH), 67.1 ($CH_2$), 61.7 ($CH_2$), 49.1 ($C_q$), 33.7 (d, *J* = 5.9 Hz, $CH_2$), 32.9 (d, *J* = 4.3 Hz, $CH_2$), 32.5 (d, *J* = 22.5 Hz, CH), 14.3 ($CH_3$). **$^{19}$F NMR** (282 MHz, $CDCl_3$) δ = -199.21. **IR** (ATR): 2956, 1728, 1585, 1462, 1265, 1162, 1025, 778, 740 $cm^{-1}$. **MS** (ESI) *m*/*z* (relative intensity): 448 $[M+H]^+$ (80), 470 $[M+Na]^+$ (100). **HR-MS** (ESI) *m*/*z* calcd for $C_{27}H_{26}NO_4F$ $[M+H]^+$: 448.1919, found: 448.1921.

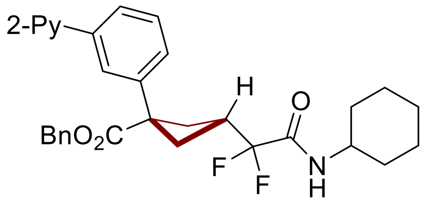


**Benzyl 3-(2-(cyclohexylamino)-1,1-difluoro-2-oxoethyl)-1-(3-(pyridin-2-yl)phenyl)cyclobutane-1-carboxylate** (**56**). The product was obtained following the general procedure with **1a** (56 mg, 0.3 mmol), 2-phenylpyridine (140 mg, 0.9 mmol), and 2-bromo-N-cyclohexyl-2,2-difluoroacetamide (230 mg, 0.9 mmol) for 24 h. Purification by column chromatography on silica gel (*n*-hexane/EtOAc = 10/1→2/1) yielded **56** (56 mg, 36%) as a colorless oil with two diastereomers (*cis*: *trans* = 2.8:1).

$R_f$ = 0.13 (TLC: *n*-hexane/EtOAc = 2/1). **$^1$H NMR** (500 MHz, $CDCl_3$) δ = 8.71 – 8.68 (m, 1H, *cis*+*trans*), 8.02 (s, 0.72H, *cis*), 7.94 – 7.85 (m, 1H, *cis*+*trans*), 7.85 (t, *J* = 1.8 Hz, 0.29H, *trans*), 7.76 – 7.72 (m, 1H, *cis*+*trans*), 7.68 – 7.65 (m, 1H, *cis*+*trans*), 7.48 – 7.41 (m, 2H, *cis*+*trans*), 7.29 – 7.21 (m, 4H, *cis*+*trans*), 7.22 – 7.13 (m, 2H, *cis*+*trans*), 5.11 (s, 0.49H, *trans*), 5.08 (s, 1.38H, *cis*), 3.80 – 3.70 (m, 1H, *cis*+*trans*), 3.27 – 2.93 (m, 3H, *cis*+*trans*), 2.84 – 2.67 (m, 2H, *cis*+*trans*), 1.96 – 1.87 (m, 2H, *cis*+*trans*), 1.77 – 1.67 (m, 2H, *cis*+*trans*), 1.67 – 1.59 (m, 1H, *cis*+*trans*), 1.42 – 1.11 (m, 6H, *cis*+*trans*). **$^{13}$C NMR** (126 MHz, $CDCl_3$) δ = 174.7 ($C_q$, *trans*), 174.5 ($C_q$, *cis*), 162.7 (t, *J* = 29.0 Hz, $C_q$, *trans*), 162.7 (t, *J* = 29.0 Hz, $C_q$, *cis*), 157.3 ($C_q$, *trans*), 157.2 ($C_q$, *cis*), 149.8 (CH, *cis*), 149.8 (CH, *trans*), 143.5 ($C_q$, *trans*), 141.7 ($C_q$, *cis*), 139.9 ($C_q$, *cis*), 139.7 ($C_q$, *trans*), 136.9 (CH, *cis*), 136.8 (CH, *trans*), 135.9 ($C_q$, *cis*), 135.9 ($C_q$, *trans*), 129.1 (CH, *cis*), 128.9 (CH, *trans*), 128.6 (CH, *trans*), 128.5 (CH, *cis*), 128.2 (CH, *trans*), 128.2 (CH, *cis*), 127.8 (CH, *cis*), 127.7 (CH, *trans*), 127.3 (CH, *cis*), 126.7 (CH, *trans*), 126.1 (CH, *cis*), 125.8 (CH, *trans*), 125.4 (CH, *cis*), 124.7 (CH, *trans*), 122.4 (CH, *cis*), 122.4 (CH, *trans*), 120.8 (CH, *cis*+*trans*), 117.3 (t, *J* = 253.2 Hz, $C_q$, *cis*), 116.7 (t, *J* = 253.2 Hz, $C_q$, *trans*), 67.1 ($CH_2$, *trans*), 66.9 ($CH_2$, *cis*), 48.6 (CH, *cis*), 48.6 (CH, *trans*), 48.1 ($C_q$, *cis*+*trans*), 33.3 (t, *J* = 26.3 Hz, CH, *trans*), 32.8 ($CH_2$, *cis*), 32.8 ($CH_2$, *trans*), 31.8 (t, *J* = 4.9 Hz, $CH_2$, *trans*), 31.7 (t, *J* = 26.3 Hz, CH, *cis*), 31.3 (t, *J* = 4.9 Hz, $CH_2$, *cis*), 25.4 ($CH_2$, *cis*), 25.4 ($CH_2$, *trans*), 24.8 ($CH_2$, *cis*), 24.8 ($CH_2$, *trans*). **$^{19}$F NMR** (471 MHz, $CDCl_3$) δ = -114.84 – -115.01 (m, *cis*+*trans*). **IR** (ATR): 3330, 2932, 1730, 1683, 1218, 1171, 1130, 740 $cm^{-1}$. **MS** (ESI) *m*/*z* (relative intensity): 519 $[M+H]^+$ (30), 541 $[M+Na]^+$ (100). **HR-MS** (ESI) *m*/*z* calcd for $C_{31}H_{32}N_2O_3F_2$ $[M+H]^+$: 519.2454, found: 519.2459.

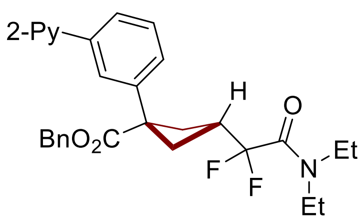


**Benzyl 3-(2-(diethylamino)-1,1-difluoro-2-oxoethyl)-1-(3-(pyridin-2-yl)phenyl)cyclobuta**

**ne-1-carboxylate** (**57**). The product was obtained following the general procedure with **1a** (56 mg, 0.3 mmol), 2-phenylpyridine (140 mg, 0.9 mmol), and 2-bromo-N,N-diethyl-2,2-difluoroacetamide (207 mg, 0.9 mmol) for 24 h. Purification by column chromatography on silica gel (*n*-hexane/EtOAc = 10/1→2/1) yielded **57** (92 mg, 62%) as a colorless oil with two diastereomers (*cis*: *trans* = 3.8:1). **R**$_f$= 0.2 (TLC: *n*-hexane/EtOAc = 2/1). **$^1$H NMR** (400 MHz, $CDCl_3$) δ = 8.71 – 8.68 (m, 1H, *cis*+*trans*), 8.05 (t, *J* = 1.9 Hz, 0.74H, *cis*), 7.94 – 7.84 (m, 1.28H, *cis*+*trans*), 7.76 – 7.70 (m, 1H, *cis*+*trans*), 7.69 – 7.64 (m, 1H, *cis*+*trans*), 7.49 – 7.41 (m, 1.71H, *cis*+*trans*), 7.33 – 7.30 (m, 0.43H, *trans*), 7.26 – 7.20 (m, 4H, *cis*+*trans*), 7.20 – 7.15 (m, 2H, *cis*+*trans*), 5.11 (s, 0.39H, *trans*), 5.08 (s, 1.49H, *cis*), 3.57 – 3.44 (m, 2H, *cis*+*trans*), 3.39 – 3.30 (m, 2H, *cis*+*trans*), 3.19 – 3.01 (m, 3H, *cis*+*trans*), 2.91 – 2.67 (m, 2H, *cis*+*trans*), 1.22 – 1.12 (m, 6H, *cis*+*trans*). **$^{13}$C NMR** (101 MHz, $CDCl_3$) δ = 174.9 ($C_q$, *trans*), 174.8 ($C_q$, *cis*), 162.3 (t, *J* = 29.2 Hz, $C_q$, *trans*), 162.2 (t, *J* = 29.2 Hz, $C_q$, *cis*), 157.3 ($C_q$, *trans*), 157.3 ($C_q$, *cis*), 149.8 (CH, *cis*), 149.8 (CH, *trans*), 143.9 ($C_q$, *trans*), 142.1 ($C_q$, *cis*), 139.8 ($C_q$, *cis*), 139.6 ($C_q$, *trans*), 136.8 (CH, *cis*), 136.8 (CH, *trans*), 136.1 ($C_q$, cis), 136.0 ($C_q$, trans), 129.0 (CH, *cis*), 128.9 (CH, *trans*), 128.5 (CH, *trans*), 128.5 (CH, *cis*), 128.1 (CH, *trans*), 128.0 (CH, *cis*), 127.7 (CH, *cis*), 127.6 (CH, *trans*), 127.4 (CH, *cis*), 126.9 (CH, *trans*), 125.9 (CH, *cis*), 125.6 (CH, *trans*), 125.5 (CH, *cis*), 124.8 (CH, *trans*), 122.3 (CH, *cis*), 122.3 (CH, *trans*), 120.8 (CH, *cis*+*trans*), 119.1 (t, *J* = 256.5 Hz, $C_q$, *cis*), 118.5 (t, *J* = 256.5 Hz, $C_q$, *trans*), 67.0 ($CH_2$, *trans*), 66.7 ($CH_2$, *cis*), 48.7 ($C_q$, *trans*), 48.1 ($C_q$, *cis*), 41.7 ($CH_2$, *cis*), 41.6 ($CH_2$, *trans)*, 41.3 ($CH_2$, *cis*), 41.3 ($CH_2$, *trans*), 34.1 (t, *J* = 26.2 Hz, CH, *trans*), 32.7 (t, *J* = 26.0 Hz, CH, *cis*), 32.3 (t, *J* = 5.4 Hz, $CH_2$, *trans*), 31.9 (t, *J* = 5.4 Hz, $CH_2$, *cis)*, 14.4 ($CH_3$, *cis*), 14.3 ($CH_3$, *trans*), 12.4 ($CH_3$, *cis*+*trans*). **$^{19}$F NMR** (377 MHz, $CDCl_3$) δ = -108.53 (d, *J* = 15.8 Hz, *trans*), -108.67 (d, *J* = 15.6 Hz, *cis*). **IR** (ATR): 2979, 1729 1658, 1459, 1213, 1108, 1025, 745 $cm^{-1}$. **MS** (ESI) *m*/*z* (relative intensity): 493 $[M+H]^+$ (30), 515 $[M+Na]^+$ (100). **HR-MS** (ESI) *m*/*z* calcd for $C_{29}H_{30}N_2O_3F_2$ $[M+H]^+$: 493.2297, found: 493.2318.

## 5. Gram-Scale Synthesis and Late-Stage Derivatization

### 5.1 Gram-scale

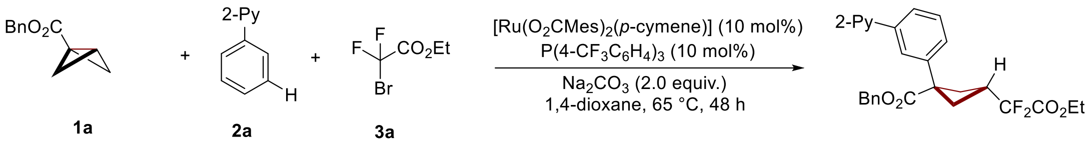


In an $N_2$ filled glovebox, $[Ru(O_2CMes)_2(p\text{-cymene})]$ (252 mg, 10.0 mol%), $P(4\text{-}CF_3C_6H_4)_3$ (210 mg, 10.0 mol%), $Na_2CO_3$ (954 mg, 9. 0 mmol, 2.0 equiv.), 1,4-dioxane (30.0 mL), **1a** (840 mg, 4.5 mmol, 1.0 equiv.), **2a** (2.1 g, 13.5 mmol, 3.0 equiv.), **3a** (2.75 g, 13.5 mmol, 3.0 equiv.) were added into an oven-dried 100 mL round bottomed flask. The flask was capped with a septum, sealed with electrical tape, and moved out of the glovebox. The reaction mixture was stirred at 65 °C for 48 h. After the completion of reaction, the mixture was filtered through a pad of Celite, washing with EtOAc. The combined organic solvent was removed under reduced pressure. The residue was purified by column chromatography on silica gel (*n*-hexane/EtOAc =5/1) to afford the desired product **4** in 1.4 g, 67% yield (isolated *trans*-isomers: 353 mg, *cis*-isomers: 1049 mg, *cis: trans* =3:1).

### 5.2 Late-Stage Derivatization

#### 5.2.1. Photo-induced ruthenium-catalyzed *ortho*-C(sp2)−H functionalization[5]

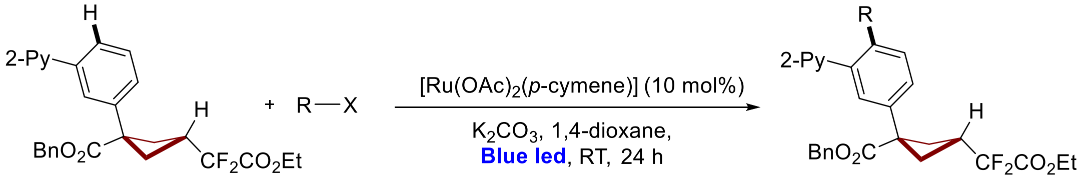


*cis*-**4**

In an $N_2$ filled glovebox, $[Ru(OAc)_2(p\text{-cymene})]$ (7.1 mg, 10.0 mol %), $K_2CO_3$ (55 mg, 0.4 mmol, 2.0 equiv.), 1,4-dioxane (1.0 mL), *cis*-**4** (93 mg, 0.2 mmol, 1.0 equiv.) and ArI or RBr (0.4 mmol, 2.0 equiv.) were added into an oven-dried 10 mL vial. The vial was capped with a septum, sealed with electrical tape, and moved out of the glovebox. The reaction mixture was stirred under visible light irradiation (2 x Kessil A360N, temperature was maintained between 30~35 °C). The distance of the lamps from the reaction vial is 9 cm. After 24 h, the mixture was filtered through a pad of Celite ($Et_2O$),

and the solvent was removed under reduced pressure. The desired products arylation **58** and allylation **59** were purified by column chromatography on silica gel (*n*-hexane/EtOAc).

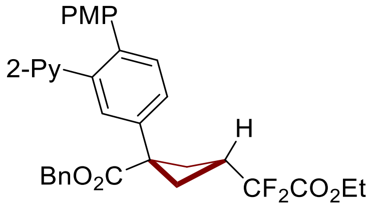


**Benzyl (1s,3s)-3-(2-ethoxy-1,1-difluoro-2-oxoethyl)-1-(4'-methoxy-2-(pyridin-2-yl)-[1,1'-biphenyl]-4-yl)cyclobutane-1-carboxylate** (**58**). The product was obtained following the above procedure with 1-iodo-4-methoxybenzene (94 mg, 0.4 mmol). $\mathbf{R_f}$ = 0.47 (TLC: *n*-hexane/EtOAc = 2/1). Purification by column chromatography on silica gel (*n*-hexane/EtOAc = 5/1→2/1) yielded **58** (112 mg, 98%) as a colorless oil. **$^1$H NMR** (500 MHz, $CDCl_3$) δ = 8.64 (ddd, *J* = 4.9, 1.8, 1.0 Hz, 1H), 7.72 (d, *J* = 2.1 Hz, 1H), 7.49 (dd, *J* = 8.0, 2.1 Hz, 1H), 7.43 – 7.39 (m, 2H), 7.28 – 7.25 (m, 3H), 7.22 – 7.19 (m, 2H), 7.12 (ddd, *J* = 7.5, 4.9, 1.1 Hz, 1H), 7.08 – 7.05 (m, 2H), 6.88 (dt, *J* = 7.9, 1.1 Hz, 1H), 6.80 – 6.76 (m, 2H), 5.10 (s, 2H), 4.29 (q, *J* = 7.2 Hz, 2H), 3.78 (s, 3H), 3.12 – 3.04 (m, 2H), 2.96 – 2.76 (m, 3H), 1.33 (t, *J* = 7.1 Hz, 3H). **$^{13}$C NMR** (126 MHz, $CDCl_3$) δ = 174.3 ($C_q$), 163.8 (t, *J* = 33.3 Hz, $C_q$), 159.3 ($C_q$), 158.8 ($C_q$), 149.6 (CH), 139.9 ($C_q$), 139.7 ($C_q$), 139.5 ($C_q$), 135.9 ($C_q$), 135.4 (CH), 133.2 ($C_q$), 130.8 (CH), 128.9 (CH), 128.5 (CH), 128.1 (CH), 127.8 (CH), 127.0 (CH), 125.6 (CH), 121.6 (CH), 115.4 (t, *J* = 250.7 Hz, $C_q$), 113.7 (CH), 66.9 ($CH_2$), 63.0 ($CH_2$), 55.3 ($CH_3$), 47.6 ($C_q$), 32.1 (t, *J* = 25.8 Hz, CH), 31.1 (t, *J* = 4.8 Hz, $CH_2$), 14.1 ($CH_3$). **$^{19}$F NMR** (471 MHz, $CDCl_3$) δ = -114.26 (d, *J* = 15.4 Hz). **IR** (ATR): 2961, 1763, 1730, 1463, 1306, 1249, 1039, 745 $cm^{-1}$. **MS** (ESI) *m*/*z* (relative intensity): 572 $[M+H]^+$ (30), 594 $[M+Na]^+$ (100). **HR-MS** (ESI) *m*/*z* calcd for $C_{34}H_{31}NO_5F_2$ $[M+Na]^+$: 594.2063, found: 594.2068.

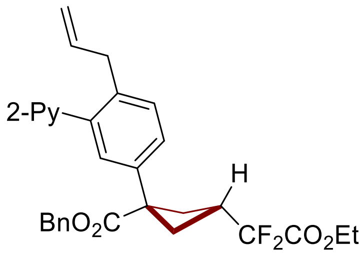


**Benzyl (1s,3s)-1-(4-allyl-3-(pyridin-2-yl)phenyl)-3-(2-ethoxy-1,1-difluoro-2-oxoethyl)cyclobutane-1-carboxylate** (**59**). The product was obtained following the above procedure with 3-bromoprop-1-ene (48 mg, 0.4 mmol). **R**$_f$ = 0.15 (TLC: *n*-hexane/EtOAc = 5/1). Purification by column chromatography on silica gel (*n*-hexane/EtOAc = 10/1→5/1) yielded **59** (59 mg, 58%) as a colorless oil. $^1$**H NMR** (500 MHz, $CDCl_3$) δ = 8.68 (ddd, *J* = 4.9, 1.8, 1.0 Hz, 1H), 7.71 (td, *J* = 7.7, 1.8 Hz, 1H), 7.42 – 7.38 (m, 2H), 7.30 (dd, *J* = 7.8, 1.1 Hz, 2H), 7.28 – 7.24 (m, 4H), 7.20 – 7.16 (m, 2H), 5.85 (ddt, *J* = 16.6, 10.1, 6.5 Hz, 1H), 5.07 (s, 2H), 4.98 – 4.94 (m, 1H), 4.88 (dq, *J* = 17.0, 1.7 Hz, 1H), 4.27 (q, *J* = 7.1 Hz, 2H), 3.47 (dt, *J* = 6.5, 1.6 Hz, 2H), 3.05 – 2.99 (m, 2H), 2.90 – 2.68 (m, 3H), 1.31 (t, *J* = 7.2 Hz, 3H). $^{13}$**C NMR** (126 MHz, $CDCl_3$) δ = 174.3 ($C_q$), 163.8 (t, *J* = 33.3 Hz, $C_q$), 159.6 ($C_q$), 149.3 (CH), 140.8 ($C_q$), 138.9 ($C_q$), 137.4 (CH), 137.2 ($C_q$), 136.3 (CH), 136.0 ($C_q$), 130.5 (CH), 128.5 (CH), 128.3 (CH), 128.1 (CH), 127.8 (CH), 126.8 (CH), 124.3 (CH), 122.1 (CH), 115.9 ($CH_2$), 115.4 (t, *J* = 249.5 Hz, $CH_2$), 66.9 ($CH_2$), 63.0 ($CH_2$), 47.4 ($C_q$), 37.2 ($CH_2$), 32.1 (t, *J* = 25.7 Hz, CH), 31.1 (t, *J* = 4.8 Hz, $CH_2$), 14.1 ($CH_3$). $^{19}$**F NMR** (471 MHz, $CDCl_3$) δ = -114.28 (d, *J* = 15.5 Hz). **IR** (ATR): 2978, 1763, 1730, 1465, 1308, 1226, 1039, 746 cm$^{-1}$. **MS** (ESI) *m*/*z* (relative intensity): 506 $[M+H]^+$ (40), 528 $[M+Na]^+$ (100). **HR-MS** (ESI) *m*/*z* calcd for $C_{30}H_{29}NO_4F_2$ $[M+Na]^+$: 528.1957, found: 528.1963.

### 5.2.2. Conversion of pyridine ring to polycyclic pyridinium salt[5]

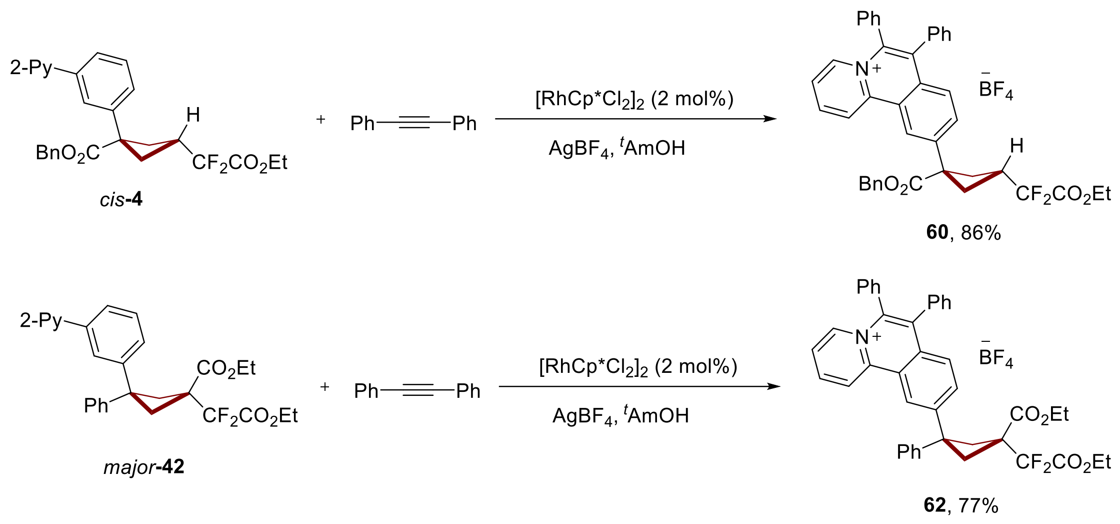


A 15 mL Schlenk tube was charged with alkyne (18 mg, 0.1 mmol), $[RhCl_2Cp^*]_2$ (1.3 mg, 2 mol%), $AgBF_4$ (39 mg, 0.2 mmol, 2.0 equiv.), $^t$AmOH (1 mL) and *cis*-**4** (56 mg, 0.12 mmol, 1.2 equiv.) or *major*-**42** (57 mg, 0.12 mmol, 1.2 equiv.). The mixture was stirred at RT for 24 h. Afterwards, the mixture was diluted with $CH_2Cl_2$ and filtered through a pad of celite, which was washed with $CH_2Cl_2$. The filtrate was concentrated in vacuo. The desired products **60** and **62** were purified by column chromatography on silica gel (DCM/MeOH), respectively.

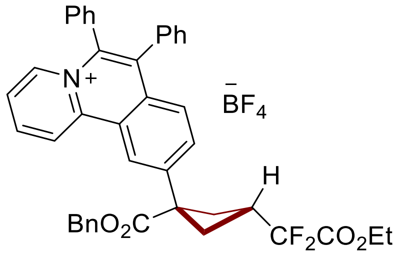


**10-((1s,3s)-1-((Benzyloxy)carbonyl)-3-(2-ethoxy-1,1-difluoro-2-oxoethyl)cyclobutyl)-6,7-diphenylpyrido[2,1-a]isoquinolin-5-ium tetrafluoroborate** (**60**). The product was obtained following the above procedure. **R**$_f$= 0.24 (TLC: DCM/MeOH = 20/1). Purification by column chromatography on silica gel (DCM/MeOH = 20/1) yielded **60** (75 mg, 86%) as a yellow solid. **M.p.**: 200 – 206 °C. **$^1$H NMR** (500 MHz, $CDCl_3$) δ = 9.44 (d, $J$ = 8.7 Hz, 1H), 8.97 (s, 1H), 8.75 (d, $J$ = 6.9 Hz, 1H), 8.54 (t, $J$ = 7.9 Hz, 1H), 7.92 – 7.85 (m, 2H), 7.59 (d, $J$ = 8.6 Hz, 1H), 7.50 – 7.37 (m, 6H), 7.35 – 7.25 (m, 5H), 7.26 – 7.19 (m, 4H), 5.10 (s, 2H), 4.32 (q, $J$ = 7.0 Hz, 2H), 3.28 –

3.19 (m, 2H), 3.01 – 2.91 (m, 3H), 1.34 (t, $J$ = 7.1 Hz, 3H). $^{13}$**C NMR** (126 MHz, $CDCl_3$) δ = 173.4 ($C_q$), 163.6 (t, $J$ = 33.1 Hz, $C_q$), 144.7 ($C_q$), 143.6 ($C_q$), 140.4 (CH), 138.6 ($C_q$), 136.7 ($C_q$), 136.3 (CH), 135.5 ($C_q$), 134.0 ($C_q$), 132.9 (CH), 131.8 ($C_q$), 131.1 (CH), 130.7 ($C_q$), 130.6 (CH), 130.1 (CH), 129.9 (CH), 128.7 (CH), 128.6 (CH), 128.4 (CH), 128.3 (CH), 128.2 (CH), 125.0 ($C_q$), 124.7 (CH), 124.3 (CH), 123.2 (CH), 115.3 (t, $J$ = 250.1 Hz, $C_q$), 67.6 ($CH_2$), 63.3 ($CH_2$), 48.7 ($C_q$), 32.1 (t, $J$ = 25.7 Hz, CH), 31.2 (t, $J$ = 4.8 Hz, $CH_2$), 14.1 ($CH_3$). $^{19}$**F NMR** (471 MHz, $CDCl_3$) δ = -114.46 (d, $J$ = 13.9 Hz), -153.30, -153.36. **IR** (ATR): 2962, 1762, 1729, 1466, 1314, 1055, 910, 703 cm$^{-1}$. **HR-MS** (ESI) *m*/*z* calcd for $C_{41}H_{34}NO_4F_2$ $[M]^+$: 642.2450, found: 642.2448.

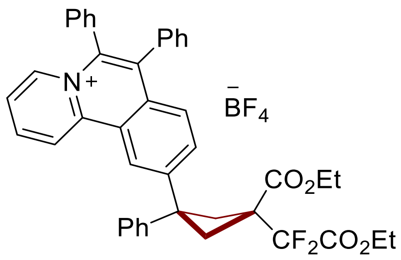


**10-((1r,3r)-3-(2-Ethoxy-1,1-difluoro-2-oxoethyl)-3-(ethoxycarbonyl)-1-phenylcyclobutyl)-6,7-diphenylpyrido[2,1-a]isoquinolin-5-ium tetrafluoroborate** (**62**). The product was obtained following the above procedure. Purification by column chromatography on silica gel (DCM/MeOH = 20/1) yielded **62** (57 mg, 77%) as a yellow solid. Recrystallized on DCM/*n*-hexanes. **M.p.**: >243 °C. $^{1}$**H NMR** (500 MHz, $CDCl_3$) δ = 9.79 (d, $J$ = 9.1 Hz, 1H), 9.23 (d, $J$ = 1.8 Hz, 1H), 8.76 – 8.66 (m, 2H), 7.94 – 7.87 (m, 2H), 7.53 (d, $J$ = 8.7 Hz, 1H), 7.46 – 7.39 (m, 5H), 7.33 – 7.27 (m, 7H), 7.16 – 7.11 (m, 3H), 4.35 (q, $J$ = 7.2 Hz, 2H), 3.99 (q, $J$ = 7.1 Hz, 2H), 3.71 – 3.64 (m, 4H), 1.40 (t, $J$ = 7.2 Hz, 3H), 1.01 (t, $J$ = 7.1 Hz, 3H). $^{13}$**C NMR** (126 MHz, $CDCl_3$) δ = 171.0 (t, $J$ = 5.1 Hz, $C_q$), 163.1 (t, $J$ = 33.0 Hz, $C_q$), 152.5 ($C_q$), 148.0 ($C_q$), 143.7 ($C_q$), 140.6 (CH), 137.7 ($C_q$), 137.0 ($C_q$), 135.7 (CH), 133.9 ($C_q$), 133.4 (CH), 131.0 (CH), 130.7 (CH), 130.7 ($C_q$), 130.7 ($C_q$), 130.1 (CH), 130.0 (CH), 129.1 (CH), 128.7 (CH), 128.6 (CH), 128.2 (CH), 126.6 (CH), 125.8 (CH), 125.0 ($C_q$), 125.0 (CH), 124.5 (CH), 122.7 (CH), 114.0 (t, $J$ = 252.8 Hz, $C_q$), 63.3 ($CH_2$), 62.5 ($CH_2$), 47.8 (t, $J$

= 25.9 Hz, $C_q$), 46.1 ($C_q$), 38.6 (t, $J$ = 3.8 Hz, $CH_2$), 14.0 ($CH_3$), 13.7 ($CH_3$). **$^{19}$F NMR** (471 MHz, $CDCl_3$) δ = -112.59, -152.87, -152.93. **IR** (ATR): 2972, 1770, 1727, 1468, 1311, 1062, 703 $cm^{-1}$. **HR-MS** (ESI) *m*/*z* calcd for $C_{42}H_{36}NO_4F_2$ $[M]^+$: 656.2607, found: 656.2616.

### 5.2.3. Copper-catalyzed synthesis of N,O-bidentate organic difluoroboron complexes[6]

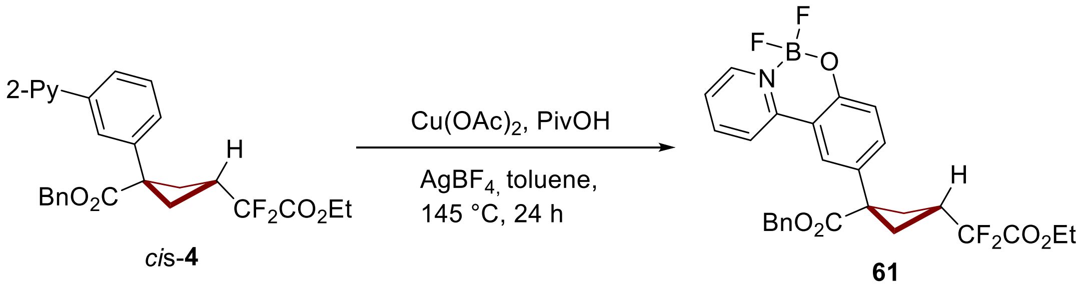


To a 20 mL Schlenk tube with a magnetic stir bar was charged with $Cu(OAc)_2$ (7.3 mg, 20 mol%), $AgBF_4$ (58 mg, 1.5 equiv.), PivOH (31 mg, 1.5 equiv.), and *cis*-**4** (93 mg, 0.2 mmol) in toluene (1.0 mL) under air atmosphere. The resulting mixture was stirred at 145 °C for 24 h and then diluted with 3 mL of dichloromethane. The solution was filtered through a celite pad and washed with dichloromethane. The filtrate was concentrated, and the residue was purified by column chromatography on silica gel to provide the desired product **61**.

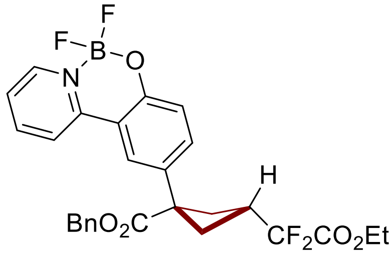


**Benzyl (1s,3s)-1-(6,6-difluoro-6H-6l4,7l4-benzo[e]pyrido[1,2-c][1,3,2]oxazaborinin-2-yl)-3-(2-ethoxy-1,1-difluoro-2-oxoethyl)cyclobutane-1-carboxylate** (**61**). The product was obtained following the above procedure. **$R_f$** = 0.15 (TLC: *n*-hexane/EtOAc/DCM = 3/1/1). Purification by column chromatography on silica gel

($n$-hexane/EtOAc/DCM = 10/5/1→6/2/1) yielded **61** (42 mg, 40%) as a yellow solid. **M.p.**: 150 – 152 °C. **$^{1}$H NMR** (500 MHz, $CDCl_3$) δ = 8.72 (ddd, $J$ = 6.0, 1.7, 0.8 Hz, 1H), 8.17 (ddd, $J$ = 8.4, 7.5, 1.7 Hz, 1H), 8.01 (d, $J$ = 8.4 Hz, 1H), 7.79 (d, $J$ = 2.4 Hz, 1H), 7.62 (ddd, $J$ = 7.3, 5.9, 1.2 Hz, 1H), 7.56 (dd, $J$ = 8.6, 2.3 Hz, 1H), 7.31 – 7.23 (m, 3H), 7.22 – 7.14 (m, 3H), 5.09 (s, 2H), 4.30 (q, $J$ = 7.2 Hz, 2H), 3.12 – 3.04 (m, 2H), 2.94 – 2.80 (m, 1H), 2.74 – 2.65 (m, 2H), 1.34 (t, $J$ = 7.1 Hz, 3H). **$^{13}$C NMR** (126 MHz, $CDCl_3$) δ = 174.2 ($C_q$), 163.7 (t, $J$ = 33.2 Hz, $C_q$), 155.3 (t, $J$ = 2.9 Hz, $C_q$), 150.0 ($C_q$), 142.5 (CH), 141.5 (t, $J$ = 2.2 Hz, CH), 135.7 (CH), 133.2 ($C_q$), 133.1 (CH), 128.7 (CH), 128.4 (CH), 128.0 (CH), 123.7 (CH), 123.3 (CH), 121.2 (CH), 120.8 (CH), 116.0 (CH), 115.4 (t, $J$ = 250.7 Hz, $C_q$), 67.1 ($CH_2$), 63.1 ($CH_2$), 47.3 ($C_q$), 32.0 (t, $J$ = 25.7 Hz, CH), 31.1 (t, $J$ = 4.9 Hz, $CH_2$) 14.1 ($CH_3$). **$^{19}$F NMR** (471 MHz, $CDCl_3$) δ = -114.25 (d, $J$ = 15.6 Hz), -147.04 (d, $J$ = 22.6 Hz). **$^{11}$B NMR** (161 MHz, $CDCl_3$) δ = 1.06. **IR** (ATR): 2961, 1762, 1728, 1498, 1308, 1065, 920, 739 $cm^{-1}$. **HR-MS** (ESI) $m/z$ calcd for $C_{27}H_{24}NO_5F_4B$ $[M+Na]^+$: 552.1581, found: 552.1581.

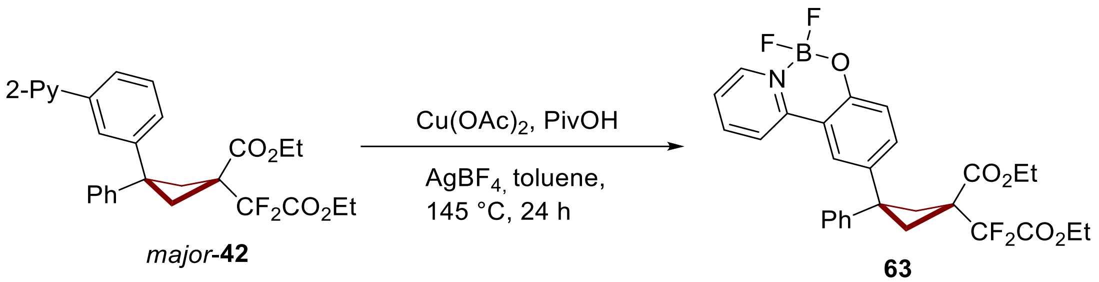


*major*-**42** **63**

To a 20 mL Schlenk tube with a magnetic stir bar was charged with $Cu(OAc)_2$ (8.0 mg, 20 mol%), $AgBF_4$ (64 mg, 1.5 equiv.), PivOH (34 mg, 1.5 equiv.), and *major*-**42** (109 mg, 0.21 mmol) in toluene (1.1 mL) under air atmosphere. The resulting mixture was stirred at 145 °C for 24 h and then diluted with 3 mL of dichloromethane. The solution was filtered through a celite pad and washed with dichloromethane. The filtrate was concentrated, and the residue was purified by column chromatography on silica gel to provide the desired product **63**.

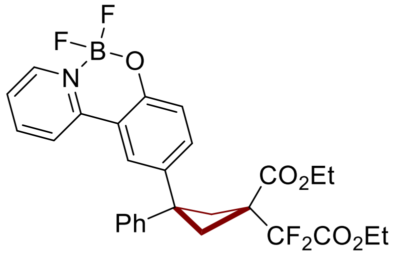

**Ethyl (1r,3r)-3-(6,6-difluoro-6H-6l4,7l4-benzo[e]pyrido[1,2-c][1,3,2]oxazaborinin-2-yl)-1-(2-ethoxy-1,1-difluoro-2-oxoethyl)-3-phenylcyclobutane-1-carboxylate (63)**. The product was obtained following the above procedure. **R**$_f$ = 0.15 (TLC: *n*-hexane/EtOAc/DCM = 3/1/1). Purification by column chromatography on silica gel (*n*-hexane/EtOAc/DCM = 10/5/1→6/2/1) yielded **63** (57 mg, 50%) as a colorless oil. **$^1$H NMR** (500 MHz, $CDCl_3$) δ = 8.67 (dt, *J* = 5.9, 1.3 Hz, 1H), 8.18 (ddd, *J* = 8.8, 7.3, 1.6 Hz, 1H), 8.13 (d, *J* = 8.4 Hz, 1H), 7.85 (d, *J* = 2.3 Hz, 1H), 7.56 (ddd, *J* = 7.3, 5.9, 1.3 Hz, 1H), 7.47 (dd, *J* = 8.7, 2.4 Hz, 1H), 7.33 – 7.26 (m, 2H), 7.26 – 7.20 (m, 2H), 7.17 (t, *J* = 7.3 Hz, 1H), 7.11 (d, *J* = 8.6 Hz, 1H), 4.31 (q, *J* = 7.1 Hz, 2H), 3.93 (q, *J* = 7.1 Hz, 2H), 3.48 – 3.37 (m, 4H), 1.34 (t, *J* = 7.1 Hz, 3H), 0.95 (t, *J* = 7.1 Hz, 3H). **$^{13}$C NMR** (126 MHz, $CDCl_3$) δ = 170.9 ($C_q$), 163.1 (t, *J* = 32.8 Hz, $C_q$), 154.3 (t, *J* = 3.0 Hz, $C_q$), 150.3 ($C_q$), 148.7 ($C_q$), 142.5 (CH), 141.4 (t, *J* = 2.2 Hz, CH), 139.4 ($C_q$), 133.7 (CH), 128.8 (CH), 126.4 (CH), 125.6 (CH), 123.1 (CH), 123.1 (CH), 121.0 (CH), 120.7 (CH), 115.5 ($C_q$), 114.1 (t, *J* = 254.5 Hz, $C_q$), 63.1 ($CH_2$), 62.2 ($CH_2$), 48.2 (t, *J* = 25.8 Hz, $C_q$), 45.1 ($C_q$), 38.7 (t, *J* = 3.5 Hz, $CH_2$), 14.0 ($CH_3$), 13.7 ($CH_3$). **$^{19}$F NMR** (471 MHz, $CDCl_3$) δ = -112.73, -146.95 (d, *J* = 20.6 Hz). **$^{11}$B NMR** (161 MHz, $CDCl_3$) δ = 1.06. **IR** (ATR): 2989, 1769, 1725, 1497, 1307, 1065, 916, 731 cm$^{-1}$. **HR-MS** (ESI) *m*/*z* calcd for $C_{28}H_{26}NO_5F_4B$ $[M+Na]^+$: 566.1737, found: 566.1764.

**5.2.4. Coversion pyridine ring to to 2-formylpyrrolepolycyclic[5]**

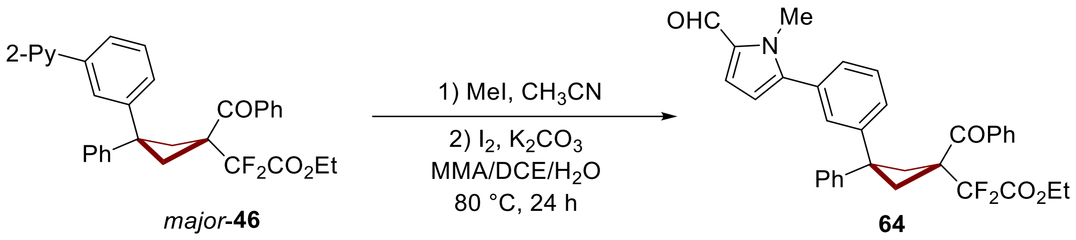

To a solution of *major*-**46** (92 mg, 0.18 mmol) in acetonitrile (0.5 mL) was added $CH_3I$ (82 mg, 0.54 mmol) under $N_2$ in a two-necked flask. The mixture was stirred at 80 °C for 12 h, and then cooled to room temperature. The solvent was removed under reduced pressure. The residue was washed with EtOAc to afford a colorless liquid, which was used for the next step without further purification. A 10 mL Schlenk tube was charged with the colorless liquid, $I_2$ (28 mg, 0.11 mmol), $K_2CO_3$ (100 mg, 0.72 mmol), methyl methacrylate (MMA) (20 μL, 0.18 mmol), $H_2O$ (0.4 mL) and DCE (0.4 mL) in the air. The mixture was stirred at 80 °C for 24 hours. After cooling to room temperature, water was added into the solution, and the mixture was extracted with EtOAc. The combined organic layers were washed with brine, dried over $MgSO_4$, filtered and concentrated. The residue was purified by chromatography on silica gel to afford the corresponding product **64**.

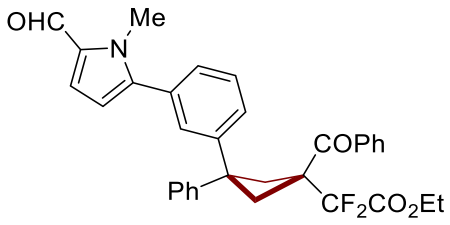

**Ethyl 2-(1-benzoyl-3-(3-(5-formyl-1-methyl-1H-pyrrol-2-yl)phenyl)-3-phenylcyclobutyl)-2,2-difluoroacetate (64)**. The product was obtained following the above procedure. **R**$_f$= 0.29 (TLC: *n*-hexane/EtOAc = 3/1). Purification by column chromatography on silica gel (*n*-hexane/EtOAc = 10/1→5/1) yielded **64** (63 mg, 65%) as a colorless oil. **$^1$H NMR** (400 MHz, $CDCl_3$) δ = 9.54 (s, 1H), 7.63 (d, *J* = 7.0 Hz, 2H), 7.49 (t, *J* = 7.5 Hz, 1H), 7.38 – 7.27 (m, 4H), 7.26 – 7.08 (m, 7H), 6.91 (d, *J* = 4.1 Hz, 1H), 6.07 (d, *J* = 4.1 Hz, 1H), 4.19 (q, *J* = 7.1 Hz, 2H), 3.72 – 3.60 (m, 7H), 1.24 (t, *J* = 7.1 Hz, 3H). **$^{13}$C NMR** (101 MHz, $CDCl_3$) δ = 199.2 ($C_q$), 179.7 (CH), 162.6 (t, *J* = 32.6 Hz,

Cq), 148.3 (Cq), 147.3 (Cq), 144.1 (Cq), 135.1 (Cq), 133.1 (Cq), 133.1 (CH), 131.1 (Cq), 129.2 (CH), 128.9 (CH), 128.7 (CH), 128.3 (CH), 127.5 (CH), 127.1 (CH), 126.8 (CH), 126.3 (CH), 125.5 (CH), 124.5 (CH), 114.7 (t, $J$ = 256.3 Hz, Cq), 110.8 (CH), 63.3 ($CH_2$), 53.4 (t, $J$ = 24.1 Hz, Cq), 45.6 (Cq), 40.7 (t, $J$ = 3.8 Hz, $CH_2$), 34.3 ($CH_3$), 13.9 ($CH_3$). **$^{19}$F NMR** (377 MHz, $CDCl_3$) δ = -108.26. **IR** (ATR): 2988, 1762, 1658, 1451, 1308, 1134, 781, 703 $cm^{-1}$. **MS** (ESI) *m*/*z* (relative intensity): 542 $[M+H]^+$ (10), 564 $[M+Na]^+$ (100). **HR-MS** (ESI) *m*/*z* calcd for $C_{33}H_{29}NO_4F_2$ $[M+Na]^+$: 564.1957, found: 564.1966.

### 5.2.5. Reduction to product to alcohol derivatives[7]

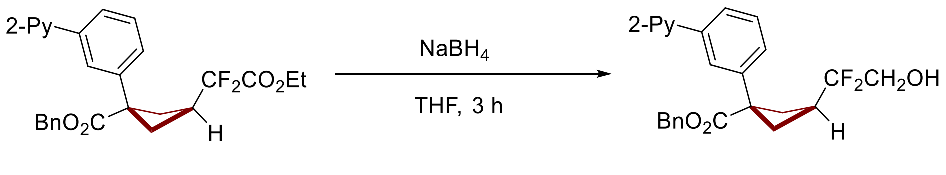


*trans*-**4** **65**, 82%

To a solution of *trans*-**4** (47 mg, 0.1 mmol) in THF (3 mL) was slowly added $NaBH_4$ (38 mg, 10 equiv.) at 0 °C. The resulting mixture was stirred at room temperature for 3 h and then the crude was diluted with saturated NaCl solution and then extracted with DCM (3 times). The combined organic layers were concentrated under vacuo. The residue was purified by column chromatography on silica gel to provide the desired product **65**.

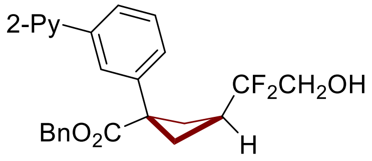


**Benzyl (1r,3r)-3-(1,1-difluoro-2-hydroxyethyl)-1-(3-(pyridin-2-yl)phenyl)cyclobutane-1-carboxylate (65)**. The product was obtained following the above procedure. **$R_f$**= 0.21 (TLC: *n*-hexane/EtOAc = 2/1). Purification by column chromatography on silica gel (*n*-hexane/EtOAc = 5/1→2/1) yielded **65** (35 mg, 82%) as a colorless oil. **$^1$H NMR** (400 MHz, $CDCl_3$) δ = 8.70 (ddd, $J$ = 4.9, 1.9, 0.9 Hz, 1H), 7.88 – 7.83 (m, 2H), 7.76

(td, $J$ = 7.7, 1.9 Hz, 1H), 7.68 (dt, $J$ = 8.0, 1.1 Hz, 1H), 7.45 (t, $J$ = 7.5 Hz, 1H), 7.33 – 7.23 (m, 5H), 7.21 – 7.16 (m, 2H), 5.13 (s, 2H), 3.68 (t, $J$ = 12.6 Hz, 2H), 3.14 – 2.98 (m, 3H), 2.82 – 2.68 (m, 3H). **$^{13}$C NMR** (101 MHz, $CDCl_3$) δ = 175.0 ($C_q$), 157.3 ($C_q$), 149.7 (CH), 143.7 ($C_q$), 139.6 ($C_q$), 137.0 (CH), 135.9 ($C_q$), 128.9 (CH), 128.6 (CH), 128.6 (CH), 128.2 (CH), 127.7 (CH), 126.8 (CH), 125.8 (CH), 124.8 (CH), 122.4 (CH), 122.0 (t, $J$ = 243.4 Hz, $C_q$), 121.0 (CH), 67.1 ($CH_2$), 63.0 (t, $J$ = 31.8 Hz, $CH_2$), 48.8 ($C_q$), 32.6 (t, $J$ = 26.7 Hz, CH), 31.9 (t, $J$ = 5.3 Hz, $CH_2$). **$^{19}$F NMR** (377 MHz, $CDCl_3$) δ = -117.57 (q, $J$ = 13.1 Hz). **IR** (ATR): 2955, 2151, 1726, 1462, 1274, 1076, 776, 744 $cm^{-1}$. **MS** (ESI) *m*/*z* (relative intensity): 424 $[M+H]^+$ (70), 446 $[M+Na]^+$ (100). **HR-MS** (ESI) *m*/*z* calcd for $C_{25}H_{23}NO_3F_2$ $[M+Na]^+$: 446.1538, found: 446.1542.

# 6. X-Ray Crystallographic Analysis

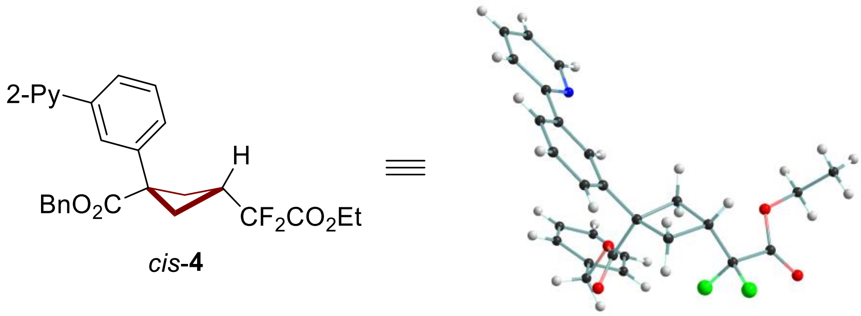


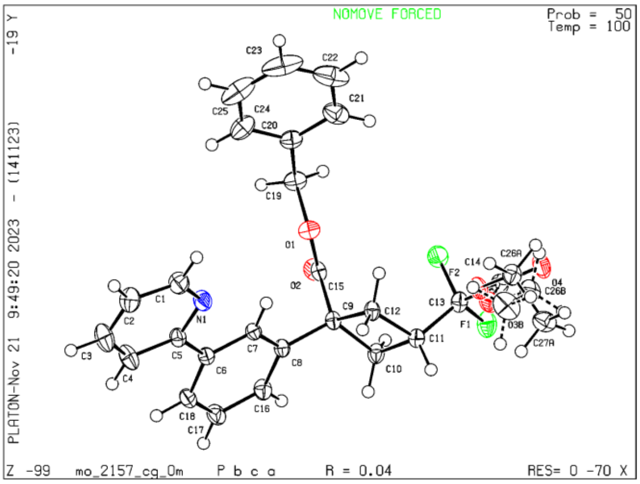


## Compound 4:

| | |
|---|---|
| CCDC number | 2344922 |
| Empirical formula | $C_{27}H_{25}F_2NO_4$ |
| Formula weight | 465.48 |
| Temperature [K] | 100.00 |
| Crystal system | orthorhombic |
| Space group (number) | *Pbca* (61) |
| *a* [Å] | 8.3511(5) |
| *b* [Å] | 18.8360(12) |
| *c* [Å] | 28.8201(19) |
| α [°] | 90 |
| β [°] | 90 |
| γ [°] | 90 |
| Volume [Å$^3$] | 4533.4(5) |
| *Z* | 8 |
| $\rho_{calc}$ [gcm$^{-3}$] | 1.364 |
| $\mu$ [mm$^{-1}$] | 0.102 |
| *F*(000) | 1952 |
| Crystal size [mm$^3$] | 0.64×0.528×0.052 |
| Crystal colour | colourless |

| | |
|---|---|
| Crystal shape | plate |
| Radiation | Mo$K_\alpha$ (λ=0.71073 Å) |
| 2θ range [°] | 4.32 to 57.41 (0.74 Å) |
| Index ranges | $-11 \leq h \leq 11$<br>$-25 \leq k \leq 25$<br>$-38 \leq l \leq 36$ |
| Reflections collected | 97486 |
| Independent reflections | 5855<br>$R_{int}$ = 0.0422<br>$R_{sigma}$ = 0.0139 |
| Completeness to θ = 25.242° | 100.0 % |
| Data / Restraints / Parameters | 5855/5/337 |
| Absorption correction $T_{min}/T_{max}$ (method) | 0.9291/1.0000 (numerical) |
| Goodness-of-fit on $F^2$ | 1.015 |
| Final $R$ indexes [$I \geq 2\sigma(I)$] | $R_1$ = 0.0412<br>w$R_2$ = 0.1053 |
| Final $R$ indexes [all data] | $R_1$ = 0.0507<br>w$R_2$ = 0.1136 |
| Largest peak/hole [eÅ$^{-3}$] | 0.45/−0.26 |

## Compound 11

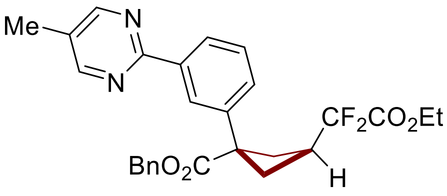

*trans*-**11**

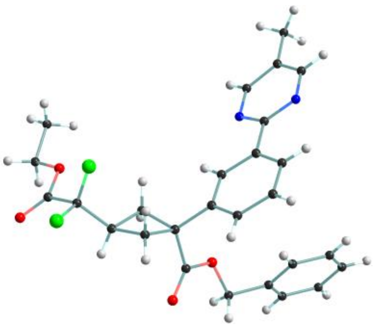

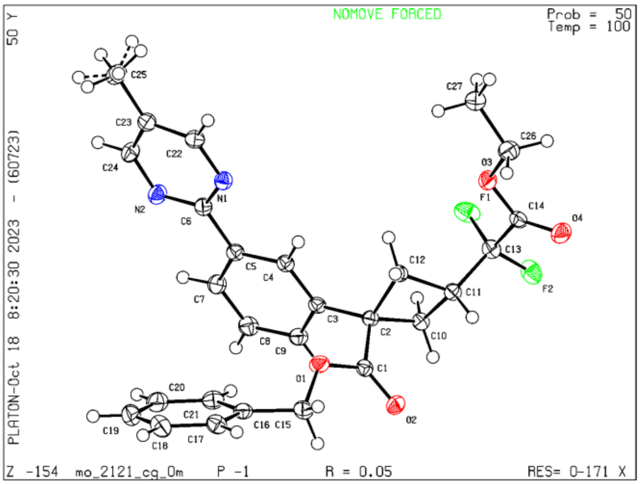


| | |
|---|---|
| CCDC number | 2344920 |
| Empirical formula | $C_{27}H_{26}F_2N_2O_4$ |
| Formula weight | 480.50 |
| Temperature [K] | 100.00 |
| Crystal system | triclinic |
| Space group (number) | $P\bar{1}$ (2) |
| *a* [Å] | 5.9961(3) |
| *b* [Å] | 13.4143(6) |
| *c* [Å] | 16.2788(8) |
| α [°] | 67.329(2) |
| β [°] | 80.531(2) |
| γ [°] | 89.643(2) |
| Volume [Å$^3$] | 1189.38(10) |
| *Z* | 2 |
| $\rho_{calc}$ [gcm$^{-3}$] | 1.342 |
| $\mu$ [mm$^{-1}$] | 0.101 |
| *F*(000) | 504 |
| Crystal size [mm$^3$] | 0.482×0.064×0.05 |
| Crystal colour | colourless |
| Crystal shape | needle |
| Radiation | Mo$K_\alpha$ (λ=0.71073 Å) |
| 2θ range [°] | 5.05 to 57.53 (0.74 Å) |
| Index ranges | $-8 \leq h \leq 8$ |
| | $-17 \leq k \leq 18$ |
| | $-21 \leq l \leq 22$ |
| Reflections collected | 54218 |
| Independent reflections | 6170 |
| | $R_{int}$ = 0.0605 |
| | $R_{sigma}$ = 0.0336 |
| Completeness to θ = 25.242° | 99.9 % |

| | |
|---|---|
| Data / Restraints / Parameters | 6170/5/334 |
| Goodness-of-fit on $F^2$ | 1.067 |
| Final $R$ indexes [$I \geq 2\sigma(I)$] | $R_1$ = 0.0454<br>w$R_2$ = 0.1133 |
| Final $R$ indexes [all data] | $R_1$ = 0.0629<br>w$R_2$ = 0.1248 |
| Largest peak/hole [eÅ$^{-3}$] | 0.31/-0.29 |

## Compound 15

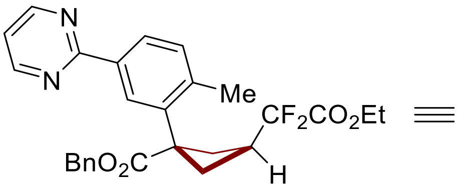


*trans*-**15**

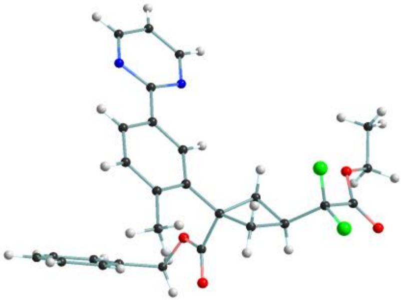

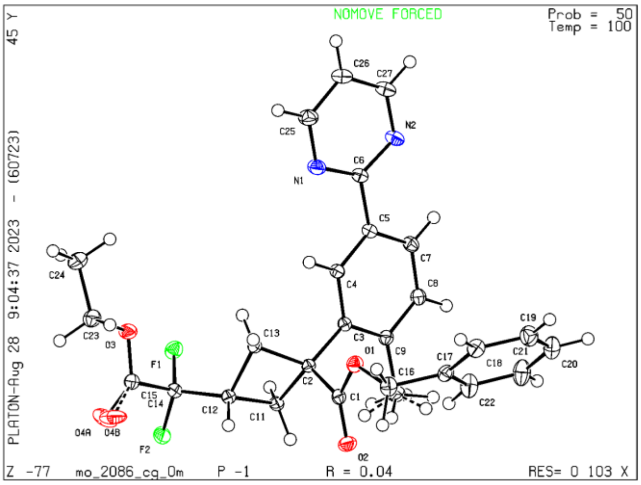


| | |
|---|---|
| CCDC number | 2344919 |
| Empirical formula | $C_{27}H_{26}F_2N_2O_4$ |
| Formula weight | 480.50 |
| Temperature [K] | 100.00 |
| Crystal system | triclinic |
| Space group (number) | $P\bar{1}$ (2) |
| $a$ [Å] | 6.2826(9) |
| $b$ [Å] | 9.2964(19) |
| $c$ [Å] | 20.794(4) |
| α [°] | 79.178(6) |
| β [°] | 88.421(9) |
| γ [°] | 73.984(7) |

| | |
|---|---|
| Volume [Å$^3$] | 1146.2(4) |
| $Z$ | 2 |
| $\rho_{calc}$ [gcm$^{-3}$] | 1.392 |
| $\mu$ [mm$^{-1}$] | 0.105 |
| $F$(000) | 504 |
| Crystal size [mm$^3$] | 0.688×0.206×0.092 |
| Crystal colour | colourless |
| Crystal shape | plank |
| Radiation | Mo$K_\alpha$ (λ=0.71073 Å) |
| 2θ range [°] | 3.99 to 65.22 (0.66 Å) |
| Index ranges | $-9 \leq h \leq 6$ |
| | $-13 \leq k \leq 14$ |
| | $-31 \leq l \leq 31$ |
| Reflections collected | 59997 |
| Independent reflections | 7944 |
| | $R_{int}$ = 0.0217 |
| | $R_{sigma}$ = 0.0125 |
| Completeness to θ = 25.242° | 100.0 % |
| Data / Restraints / Parameters | 7944/7/347 |
| Goodness-of-fit on $F^2$ | 1.032 |
| Final $R$ indexes [$I$≥2σ($I$)] | $R_1$ = 0.0367 |
| | w$R_2$ = 0.1009 |
| Final $R$ indexes [all data] | $R_1$ = 0.0417 |
| | w$R_2$ = 0.1056 |
| Largest peak/hole [eÅ$^{-3}$] | 0.50/-0.26 |

## Compound 16

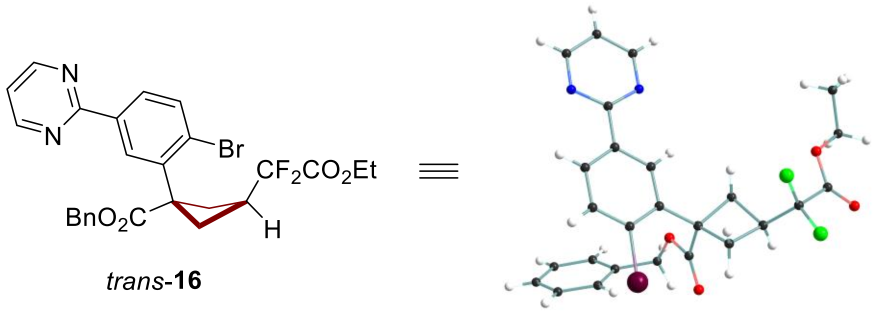


*trans*-**16**

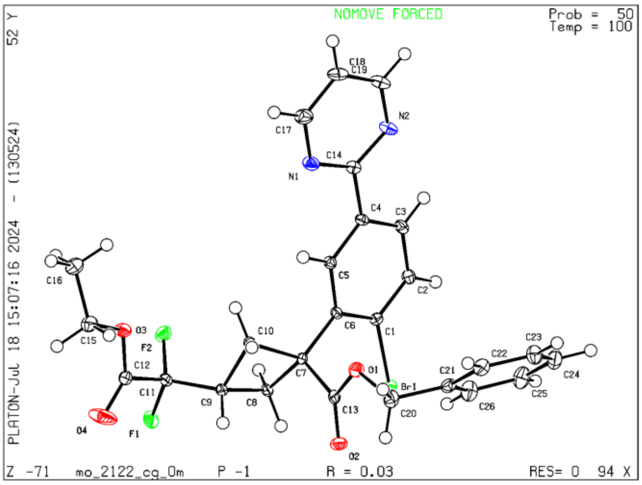


| | |
|---|---|
| CCDC number | 2344921 |
| Empirical formula | $C_{26}H_{23}BrF_2N_2O_4$ |
| Formula weight | 545.37 |
| Temperature [K] | 100.00 |
| Crystal system | triclinic |
| Space group (number) | $P\overline{1}$ (2) |
| $a$ [Å] | 6.2690(4) |
| $b$ [Å] | 9.2980(12) |
| $c$ [Å] | 20.950(3) |
| α [°] | 79.183(3) |
| β [°] | 88.173(3) |
| γ [°] | 73.796(2) |
| Volume [Å$^3$] | 1151.5(2) |
| $Z$ | 2 |
| $\rho_{calc}$ [gcm$^{-3}$] | 1.573 |
| $\mu$ [mm$^{-1}$] | 1.838 |
| $F$(000) | 556 |
| Crystal size [mm$^3$] | 1.021×0.064×0.062 |
| Crystal colour | colourless |
| Crystal shape | needle |
| Radiation | Mo$K_\alpha$ (λ=0.71073 Å) |
| 2θ range [°] | 3.96 to 59.15 (0.72 Å) |
| Index ranges | $-6 \le h \le 8$ |
| | $-12 \le k \le 12$ |
| | $-29 \le l \le 29$ |
| Reflections collected | 56568 |
| Independent reflections | 6471 |
| | $R_{int}$ = 0.0318 |
| | $R_{sigma}$ = 0.0176 |
| Completeness to | 100.0 % |
| θ = 25.242° | |

| | |
|---|---|
| Data / Restraints / Parameters | 6471/0/317 |
| Goodness-of-fit on $F^2$ | 1.143 |
| Final $R$ indexes [$I \geq 2\sigma(I)$] | $R_1$ = 0.0298<br>$wR_2$ = 0.0695 |
| Final $R$ indexes [all data] | $R_1$ = 0.0324<br>$wR_2$ = 0.0708 |
| Largest peak/hole [eÅ$^{-3}$] | 0.55/-0.89 |

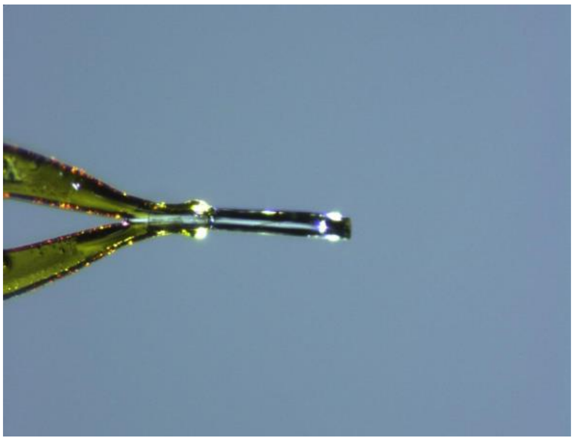

**Note**: There is a B-level Alert (expalanation: This crystal has a long needle-like shape, with one side being particularly elongated, as shown in the picture. However, this does not affect the crystal's quality). The excellent quality of our crystal is reflected in its R value and $wR_2$ value.

## Compound 62

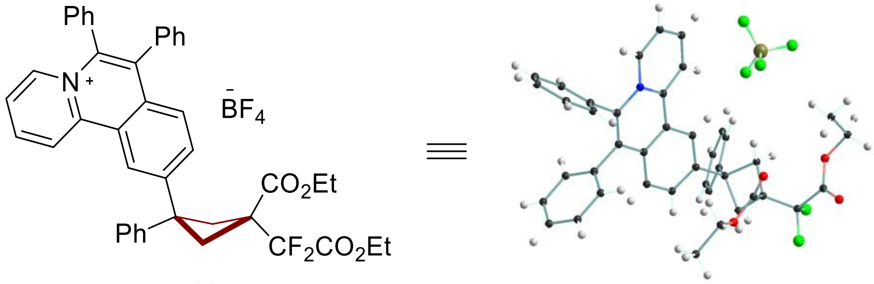


**62**

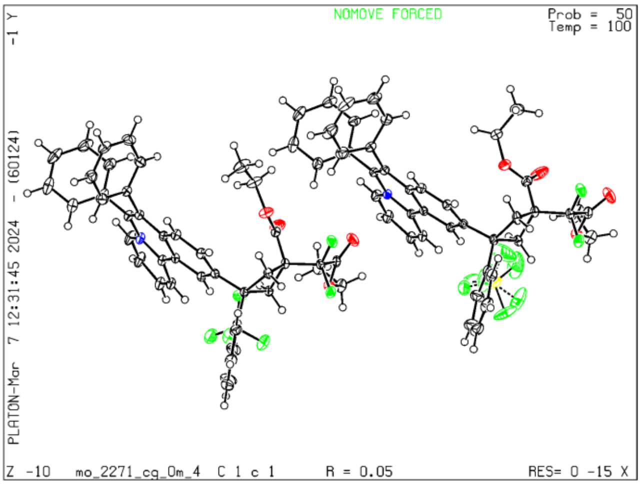


| | |
|---|---|
| CCDC number | 2344923 |
| Empirical formula | $C_{42}H_{36}BF_6NO_4$ |
| Formula weight | 743.53 |
| Temperature [K] | 100.00 |
| Crystal system | monoclinic |
| Space group (number) | *Cc* (9) |
| *a* [Å] | 21.9525(19) |
| *b* [Å] | 19.4921(16) |
| *c* [Å] | 16.6861(11) |
| α [°] | 90 |
| β [°] | 92.932(2) |
| γ [°] | 90 |
| Volume [Å$^3$] | 7130.6(10) |
| *Z* | 8 |
| $\rho_{calc}$ [gcm$^{-3}$] | 1.385 |
| $\mu$ [mm$^{-1}$] | 0.109 |
| *F*(000) | 3088 |
| Crystal size [mm$^3$] | 0.44×0.374×0.206 |
| Crystal colour | colourless |
| Crystal shape | plate |
| Radiation | Mo$K_\alpha$ (λ=0.71073 Å) |
| 2θ range [°] | 3.65 to 59.35 (0.72 Å) |
| Index ranges | $-30 \le h \le 30$ |
| | $-27 \le k \le 27$ |
| | $-23 \le l \le 23$ |
| Reflections collected | 20095 |
| Independent reflections | 20095 |
| | $R_{int}$ = 0.0558 |
| | $R_{sigma}$ = 0.0248 |
| Completeness to θ = 25.242° | 100.0 % |
| Data / Restraints / | 20095/78/1023 |

| | |
|---|---|
| Parameters | |
| Absorption correction | 0.668091/0.849665 |
| $T_{min}/T_{max}$ (method) | (multi-scan) |
| Goodness-of-fit on $F^2$ | 1.031 |
| Final $R$ indexes | $R_1$ = 0.0464 |
| [$I$≥2σ($I$)] | w$R_2$ = 0.1234 |
| Final $R$ indexes | $R_1$ = 0.0482 |
| [all data] | w$R_2$ = 0.1252 |
| Largest peak/hole [eÅ$^{-3}$] | 0.45/−0.31 |
| Flack X parameter | 0.2(2) |

# 7. Mechanism studies and Computational studies

## 7.1 Mechanism studies

### 7.1.1 Radical trapping experiment

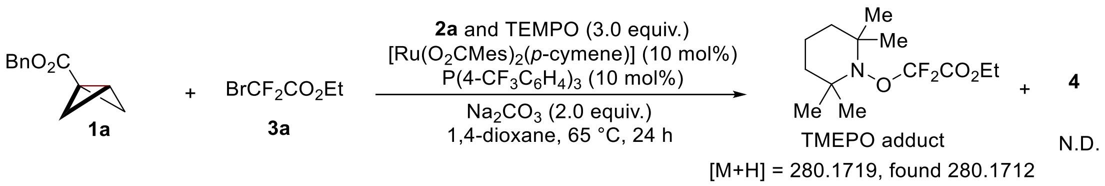


In an $N_2$ filled glovebox, $[Ru(O_2CMes)_2(p\text{-cymene})]$ (16.8 mg, 10.0 mol%), $P(4\text{-}CF_3C_6H_4)_3$ (7.0 mg, 10.0 mol%), $Na_2CO_3$ (64 mg, 0.6 mmol, 2.0 equiv.), 1,4-dioxane (2.0 mL), **1a** (56 mg, 0.3 mmol, 1.0 equiv.), **2a** (140 mg, 0.9 mmol, 3.0 equiv.), **3a** (183 mg, 0.9 mmol, 3.0 equiv.) and TEMPO (141 mg, 0.9 mmol, 3.0 equiv.) were added into an oven-dried 20 mL pressure tube. The reaction mixture was stirred at 65 ° for 24 h. After cooling to ambient temperature, no desired product was observed and the difluoroalkyl radical TEMPO adduct was detected *via* high resolution mass-spectrometry (HRMS), which means the mechanism involves a radical process.

### 7.1.2. *p*-Cymene-free ruthenium complex as the catalyst

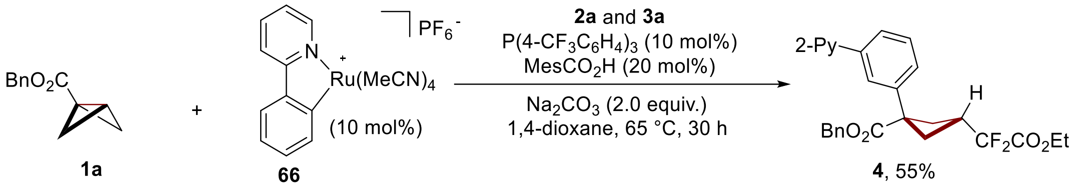


In an $N_2$ filled glovebox, ruthenium complex **66** (17 mg, 10.0 mol%), $P(4\text{-}CF_3C_6H_4)_3$ (14.0 mg, 5.0 mol%), $MesCO_2H$ (9.8 mg, 20.0 mol%), $Na_2CO_3$ (64 mg, 0.6 mmol, 2.0 equiv.), 1,4-dioxane (2.0 mL), **1a** (56 mg, 0.3 mmol, 1.0 equiv.), **2a** (140 mg, 0.9 mmol, 3.0 equiv.) and **3a** (183 mg, 0.9 mmol, 3.0 equiv.) were added into an oven-dried 20 mL pressure tube. The reaction mixture was stirred at 65 °C for 30 h. After cooling to ambient temperature, the mixture was purified by column chromatography on silica gel (*n*-hexane/EtOAc = 5/1) to afford the desired product **4** in 55% yield (*cis*: *trans* =3:1, determined by $^{19}$**F-NMR**).

### 7.1.3. Carboxylate-free ruthenium(II) phosphine complex as the catalyst

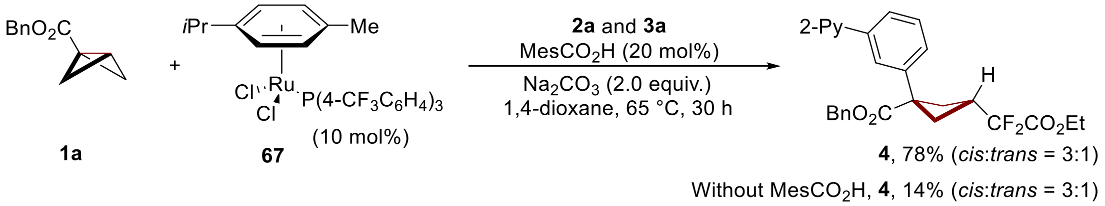


In an $N_2$ filled glovebox, Carboxylate-free ruthenium(II) phosphine complex **67** (56 mg, 10.0 mol%), $MesCO_2H$ (9.8 mg, 20.0 mol%), $Na_2CO_3$ (64 mg, 0.6 mmol, 2.0 equiv.), 1,4-dioxane (2.0 mL), **1a** (56 mg, 0.3 mmol, 1.0 equiv.), **2a** (140 mg, 0.9 mmol, 3.0 equiv.) and **3a** (183 mg, 0.9 mmol, 3.0 equiv.) were added into an oven-dried 20 mL pressure tube. The reaction mixture was stirred at 65 °C for 24 h. After cooling to ambient temperature, the mixture was purified by column chromatography on silica gel (*n*-hexane/EtOAc = 5/1) to afford the desired product **4** in 78% yield (*cis*: *trans* = 3:1, determined by $^{19}$**F-NMR**).

Control experiment was conducted without $MesCO_2H$, the desired product **4** was obtained in 14% yield (*cis*: *trans* =3:1, determined by $^{19}$**F-NMR**).

### 7.2 Computational studies

All DFT calculations were performed with Gaussian 16, Revision A.03 package[8]. Geometry optimizations were conducted at the PBE0[9,10] level of theory in combination with Grimme's D3 dispersion corrections with a Becke-Johnson damping scheme (D3BJ)[11,12] in the gas phase. All atoms were described with a def2-SVP basis set[13,14], while ruthenium was also described with a SDD pseudopotential[15,16]. Analytical frequency calculations were also carried out at the same level of theory to confirm each optimized stationary point as an energy minimum (zero imaginary frequencies) or a transition state (one imaginary frequency) and to further provide thermal and nonthermal corrections to the Gibbs free energy at 338.15 K and 1 atm. Furthermore, three DFT functionals (PBE0, B3LYP[17,18], and M06[19]) with a def2-TZVP[13,14] basis and a SDD pseudopotential for ruthenium[15,16] were further used to compute the solvation single-point energies in 1,4-dioxane with implicit solvation model SMD[20]. All reported energies are based on gas-phase Gibbs

free energies with def2-SVP basis set for which the electronic energies were corrected with a def2-TZVP basis set and solvent effects.

### 7.2.1. Reaction with monosubstitued BCB 1a.

Following the generation of the difluoroalkyl radical through ruthenacycle-mediated-halogen atom transfer (Ru-XAT) process, subsequent regio-selectivity of radical attack at the two possible sites on BCB was probed using DFT calculations. (**Figure S1-S2**). As illustrated in **Figure S1**, for monosubstituted BCB **1a**, the difluoroalkyl radical attack at the unsubstituted carbon, considering various conformers, was initially explored. Within the tested various functionals, **TS3-s4** was identified as the most stable conformer where the difluoroalkyl radical oriented inward to the BCB backbone. In the case of radical attack at the ester-substituted site, **TS4-s2** exhibits greater stability compared with other conformers with the difluoroalkyl radical lying parallel to the ester group of the BCB. During the cleavage of the σ-bridge bond in BCB **1a**, the slightly shorter bridge C−C bond distance (1.61 Å) and the longer $C{-}C_{RF}$ bond distance (2.21 Å) in **TS3-s4,** compared to **TS4-s2**, indicated the first radical attacked at the unsubstituted site proceeded through an earlier transition state that structurally resembles the starting BCBs. This was also evidenced by the relatively lower spin densities observed on the carbon atom of BCB distal to the attack pathway in **TS3-4** (0.21), compared to that on in **TS4-2 (**0.35**).** Related results are illustrated in **Figure S3**.

**Conformers for TS3:**

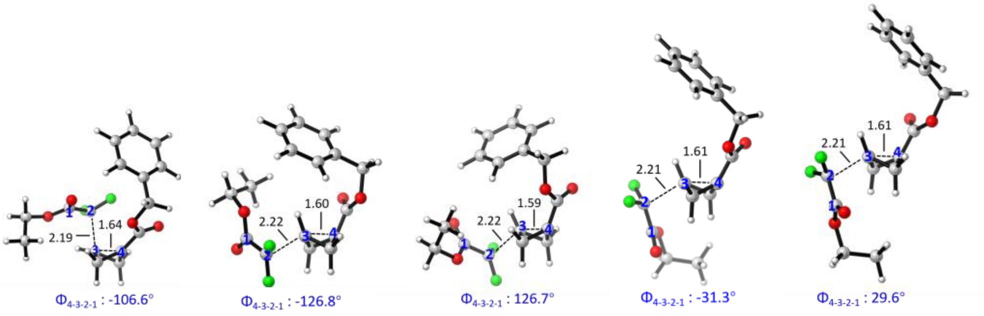


| | TS3-s1 | TS3-s2 | TS3-s3 | TS3-s4 | TS3-s5 |
|---|---|---|---|---|---|
| $\Delta G^{\ddagger}$ (kcal $mol^{-1}$) | 39.6 | 15.7 | 15.7 | 14.2 | 14.6 |
| $\Delta G^{\ddagger}$ (kcal $mol^{-1}$) | 40.8 | 16.2 | 16.3 | 14.9 | 15.4 |
| $\Delta G^{\ddagger}$ (kcal $mol^{-1}$) | 38.0 | 16.8 | 16.7 | 15.0 | 15.4 |
| $\Delta G^{\ddagger}$ (kcal $mol^{-1}$) | 41.3 | 17.3 | 17.4 | 15.9 | 16.3 |
| $\Delta G^{\ddagger}$ (kcal $mol^{-1}$) | 49.8 | 23.4 | 24.5 | 25.6 | 25.8 |

PBE0-D4/def2-TZVP-SDD-SMD(1,4-dioxane)//PBE0-D3(BJ)/def2-SVP-SDD
PBE0-D3/def2-TZVP-SDD-SMD(1,4-dioxane)//PBE0-D3(BJ)/def2-SVP-SDD
B3LYP-D4/def2-TZVP-SDD-SMD(1,4-dioxane)//PBE0-D3(BJ)/def2-SVP-SDD
B3LYP-D3/def2-TZVP-SDD-SMD(1,4-dioxane)//PBE0-D3(BJ)/def2-SVP-SDD
M06-D3/def2-TZVP-SDD-SMD(1,4-dioxane)//PBE0-D3(BJ)/def2-SVP-SDD

**Figure S1.** Conformational searches for the first radical attack step at the unsubstituted site of monosubstituted BCB **1a** at 338.15K using various functionals. Energies are presented in the unit of kcal $mol^{-1}$.

# Conformers for TS4:

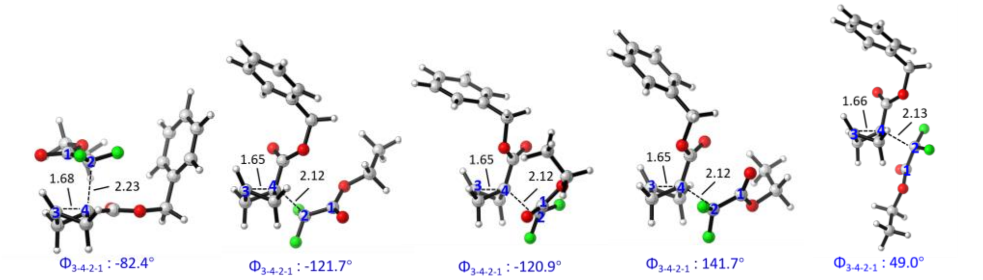


| | TS4-s1 | TS4-s2 | TS4-s3 | TS4-s4 | TS4-s5 |
|---|---|---|---|---|---|
| $\Delta G^{\ddagger}$ (kcal $mol^{-1}$) | 43.5 | 17.3 | 18.1 | 17.7 | 18.2 |
| $\Delta G^{\ddagger}$ (kcal $mol^{-1}$) | 44.8 | 18.4 | 19.2 | 18.9 | 19.3 |
| $\Delta G^{\ddagger}$ (kcal $mol^{-1}$) | 43.0 | 17.6 | 18.6 | 18.0 | 18.7 |
| $\Delta G^{\ddagger}$ (kcal $mol^{-1}$) | 44.7 | 19.2 | 20.0 | 19.7 | 20.2 |
| $\Delta G^{\ddagger}$ (kcal $mol^{-1}$) | 54.2 | 28.5 | 29.1 | 29.0 | 30.4 |

PBE0-D4/def2-TZVP-SDD-SMD(1,4-dioxane)//PBE0-D3(BJ)/def2-SVP-SDD
PBE0-D3/def2-TZVP-SDD-SMD(1,4-dioxane)//PBE0-D3(BJ)/def2-SVP-SDD
B3LYP-D4/def2-TZVP-SDD-SMD(1,4-dioxane)//PBE0-D3(BJ)/def2-SVP-SDD
B3LYP-D3/def2-TZVP-SDD-SMD(1,4-dioxane)//PBE0-D3(BJ)/def2-SVP-SDD
M06-D3/def2-TZVP-SDD-SMD(1,4-dioxane)//PBE0-D3(BJ)/def2-SVP-SDD

**Figure S2.** Conformational searches for the first radical attack step at the ester-substituted site of monosubstituted BCB **1a** at 338.15K using various functionals. Energies are presented in the unit of kcal $mol^{-1}$.

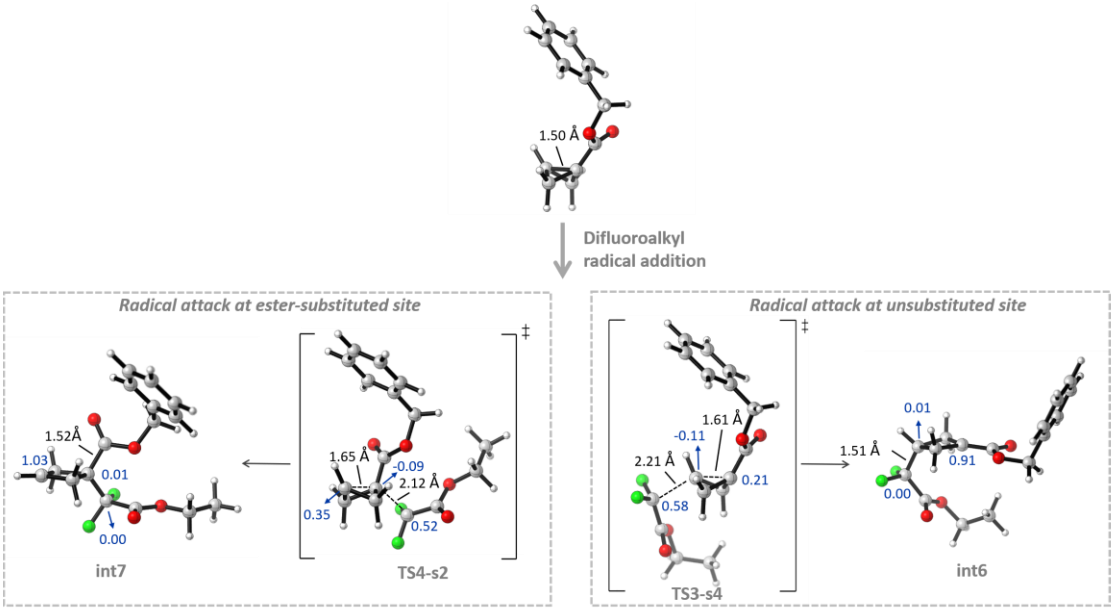


**Figure S3.** Optimized geometries and spin densities of computed structures in the first radical attack step with monosubstituted BCB **1a**.

For the second electrophilic radical attack at the phenyl ring of the ruthenium complex, the energy barriers for the generation of *cis*-and *trans*-diastereomers was found to be closely comparable. Therefore, Boltzmann distribution analysis, in combination with different hybrid functionals, was employed to further elucidate the diastereomeric ratio. According to Maxwell-Boltzmann statistics, the parameters of each low energy conformer were weighted using the Boltzmann distribution (equations 1 and 2) where the energy of a given conformer was calculated relative to the lowest energy conformation.

$$c_i = \frac{e^{-\Delta G_i^{\ddagger}/RT}}{e^{-\Delta G_1^{\ddagger}/RT} + e^{-\Delta G_2^{\ddagger}/RT} + \cdots + e^{-\Delta G_n^{\ddagger}/RT}} \quad (1)$$

$$\Delta G_w^{\ddagger} = c_1 \Delta G_1^{\ddagger} + c_2 \Delta G_2^{\ddagger} + \cdots + c_n \Delta G_n^{\ddagger} \quad (2)$$

Regarding the formation of the *cis*-diastereomer, five low-energy transition state conformers (with energy differences within 2 kcal $mol^{-1}$) were identified, including three conformers associated with radical attack from below the arene plane and the other two conformers from above. Similarly, five lowest-lying conformations were also calculated for the *trans*-diastereomer. Subsequently, based on different

regioisomeric transition states and their associated energy barriers, Boltzmann-weighted Gibbs free energies $\Delta G_w^{\ddagger}$ were calculated for the formation of *cis*- and *trans*-diastereomers, respectively. The corresponding results are presented in **Table S2**.

## Conformers for TS5$^{oss}$:

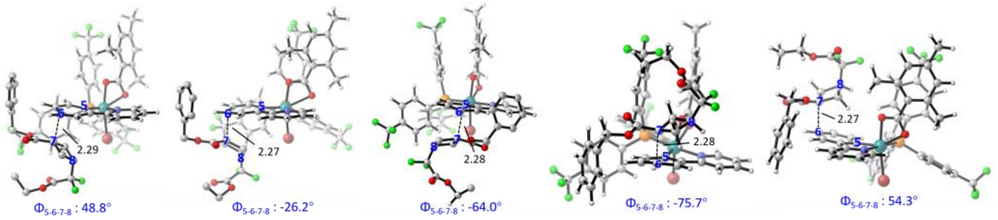


| | TS5-s1 | TS5-s2 | TS5-s3 | TS5-s4 | TS5-s5 |
|---|---|---|---|---|---|
| ΔG‡ (kcal mol$^{-1}$) | 16.7 | 16.2 | 14.8 | 17.1 | 15.0 |
| ΔG‡ (kcal mol$^{-1}$) | 16.2 | 16.0 | 14.7 | 16.0 | 14.5 |
| ΔG‡ (kcal mol$^{-1}$) | 15.4 | 15.2 | 13.9 | 16.1 | 14.2 |
| ΔG‡ (kcal mol$^{-1}$) | 14.5 | 14.6 | 13.5 | 14.4 | 13.2 |
| ΔG‡ (kcal mol$^{-1}$) | 10.6 | 12.0 | 10.3 | 8.6 | 10.4 |

PBE0-D4/def2-TZVP-SDD-SMD(1,4-dioxane)//PBE0-D3(BJ)/def2-SVP-SDD
PBE0-D3/def2 TZVP-SDD-SMD(1,4-dioxane)//PBE0-D3(BJ)/def2-SVP-SDD
B3LYP-D4/def2-TZVP-SDD-SMD(1,4-dioxane)//PBE0-D3(BJ)/def2-SVP-SDD
B3LYP-D3/def2-TZVP-SDD-SMD(1,4-dioxane)//PBE0-D3(BJ)/def2-SVP-SDD
M06-D3/def2-TZVP-SDD-SMD(1,4-dioxane)//PBE0-D3(BJ)/def2-SVP-SDD

**Figure S4.** Conformational searches for the second radical attack step at the arene moiety of the ruthenium complex at the open-shell singlet state for the generation of *cis*-product at 338.15K using various functionals. Energies are presented in the unit of kcal mol$^{-1}$.

## Conformers for TS6$^{oss}$:

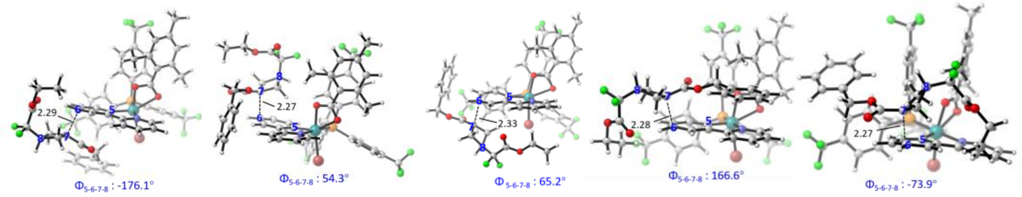


| | TS6-s1 | TS6-s2 | TS6-s3 | TS6-s4 | TS6-s5 |
|---|---|---|---|---|---|
| ΔG‡ (kcal mol$^{-1}$) | 16.8 | 17.9 | 16.9 | 18.7 | 18.7 |
| ΔG‡ (kcal mol$^{-1}$) | 16.6 | 18.2 | 16.6 | 18.5 | 17.8 |
| ΔG‡ (kcal mol$^{-1}$) | 16.1 | 16.3 | 15.3 | 16.9 | 18.2 |
| ΔG‡ (kcal mol$^{-1}$) | 15.5 | 16.5 | 14.6 | 16.4 | 16.8 |
| ΔG‡ (kcal mol$^{-1}$) | 12.5 | 12.9 | 10.8 | 12.2 | 11.1 |

PBE0-D4/def2-TZVP-SDD-SMD(1,4-dioxane)//PBE0-D3(BJ)/def2-SVP-SDD
PBE0-D3/def2 TZVP-SDD-SMD(1,4-dioxane)//PBE0-D3(BJ)/def2-SVP-SDD
B3LYP-D4/def2-TZVP-SDD-SMD(1,4-dioxane)//PBE0-D3(BJ)/def2-SVP-SDD
B3LYP-D3/def2-TZVP-SDD-SMD(1,4-dioxane)//PBE0-D3(BJ)/def2-SVP-SDD
M06/def2-TZVP-SDD-SMD(1,4-dioxane)//PBE0-D3(BJ)/def2-SVP-SDD

**Figure S5.** Conformational searches for the second radical attack step at the arene moiety of the ruthenium complex at the open-shell singlet state for the generation of *trans*-product at 338.15K using various functionals. Energies are presented in the unit of kcal mol$^{-1}$.

The calculated Boltzmann-weighted energy barrier for the formation of

*cis*-diastereomer was generally lower than that of the *trans*-diastereomer formation when employing PBE0 and B3LYP hybrid functionals. However, when employing Minnesota M06 functional, opposite selectivity was observed where the *cis*-structure became the predominant species. For each functional without dispersion correction, including PBE0-noD and B3LYP-noD, the calculated energy barrier ranged from to 30 to 38 kcal $mol^{-1}$, rendering the formation of the desired product unfeasible. These results highlight the critical role of dispersion corrections in many hybrid functionals. Further analysis of different dispersion correction versions revealed that the energy barriers calculated with D3 corrections were generally lower than those obtained with D4 corrections. Among all the tested functionals, B3LYP-D3(BJ) outperformed the others, indicating the generation of the *cis*-diastereomer being more favorable than that of the *trans*-diastereomer, with a calculated *cis*:*trans* ratio of 2.4:1 at 65 °C. This aligns with the experimentally determined ratio (3.1:1), although the computed ratio is slightly lower.

**Table S2.** Boltzmann-weighted Gibbs free energies and the corresponding ratios of the two diastereomers for the reaction with monosubstituted BCB **1a** with different functionals.

| | PBE0-D4 | PBE0-D3 | PBE0-noD | B3LYP-D4 | B3LYP-D3 | B3LYP-noD | M06 |
|---|---|---|---|---|---|---|---|
| $\boldsymbol{\Delta G_w^{\ddagger}}$(TS6$^{oss}$) | 15.0 | 14.8 | 30.5 | 14.2 | 14.4 | 36.6 | 11.6 |
| $\boldsymbol{\Delta G_w^{\ddagger}}$(TS7$^{oss}$) | 17.0 | 16.8 | 31.7 | 15.7 | 15.0 | 38.2 | 11.1 |
| $\Delta\Delta G^{\ddagger}$ | -2.0 | -2.0 | -1.2 | -1.5 | -0.6 | -1.6 | 0.5 |
| Calculated ratio | 19.6/1 | 19.6/1 | 6.0/1 | 9.3/1 | **2.4/1** | 10.8/1 | 1/2.1 |
| Experimental ratio | | | | 3.1/1 | | | |

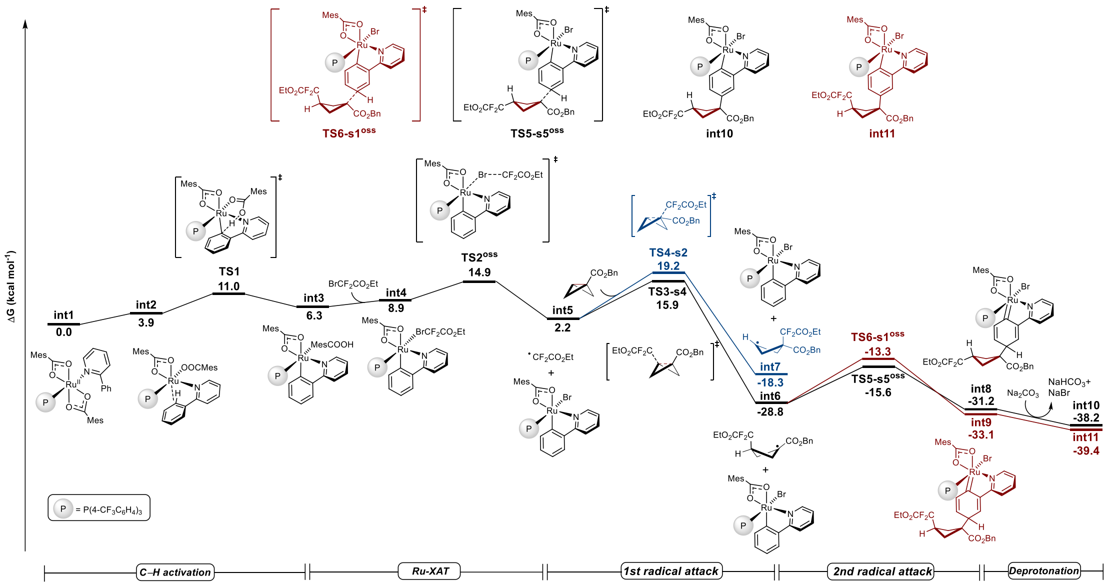

**Figure S6.** Computed relative Gibbs free energies ($\Delta G_{338.15}$) in kcal mol$^{-1}$ for ruthenium-catalyzed *meta*-C–H alkylation reaction with BCB **1a** at the B3LYP-D3/def2-TZVP-SMD(1,4-dioxane)//PBE0-D3(BJ)/def2-SVP level of theory.

### 7.2.2. Reaction with disubstituted BCB 1m.

In addition to the monosubstituted BCB **1a**, conformers investigation for the radical attack at the disubstituted BCB **1m** was also explored. Related results for phenyl- and ester-disubstituted BCB are presented from **Figure S7** to **S12**. For the first radical attack step, four distinct conformers exhibited different dihedral angles between the bridge C−C bond of the BCB core and the difluoroalkyl radical backbone. Besides, two pathways were considered depending on whether the radical attack occurs at the ester-substituted carbon (as shown in **Figure S7**) or at the phenyl-substituted site (as shown in **Figure S8**). Among the conformers explored, **TS7-s1** was considered to the most stable structure when radical attack occurred at the ester-substituted site, with consideration of different computational functionals. And for the alternative pathway, **TS8-s1** showed greater stability relative to other conformers. A comparison of these two transition states revealed that **TS7-s1** is more favorable by 1.5 kcal mol$^{-1}$ with B3LYP-D3(BJ), due to the formation of a more stable benzylic radical. The higher stability of the benzylic radical than the ester-substituted radical is supported by the relatively lower spin densities on the benzylic carbon in **int12** (0.79) than that in **int13** (0.92), as shown in **Figure S9**. In this scenario, the transition states for radical attack at the phenyl site was classified as earlier transition states, characterized by the slightly

elongated C–C bridge bond distance of BCB (1.63 Å) and the quite far C–C bond distance between the BCB and the difluoro alkyl radicals (2.25 Å) in **TS7-s1**.

## Conformers for TS7:

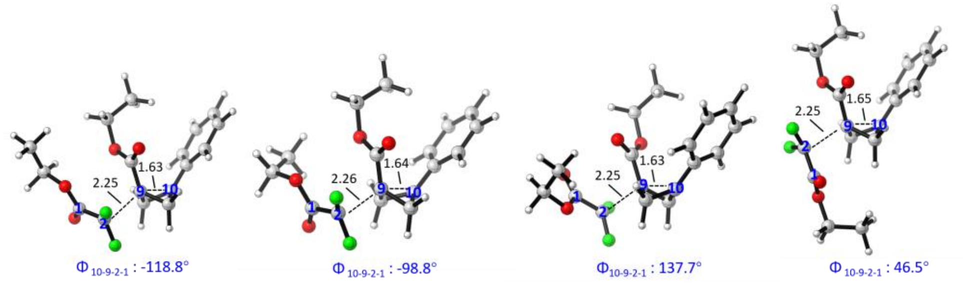


| | TS7-s1 | TS7-s2 | TS7-s3 | TS7-s4 |
|---|---|---|---|---|
| $\Delta G^{\ddagger}$ (kcal mol$^{-1}$) | 13.3 | 14.5 | 13.6 | 15.2 |
| $\Delta G^{\ddagger}$ (kcal mol$^{-1}$) | 14.3 | 15.5 | 14.7 | 16.2 |
| $\Delta G^{\ddagger}$ (kcal mol$^{-1}$) | 13.5 | 14.6 | 13.8 | 15.4 |
| $\Delta G^{\ddagger}$ (kcal mol$^{-1}$) | 14.8 | 16.0 | 15.3 | 16.7 |
| $\Delta G^{\ddagger}$ (kcal mol$^{-1}$) | 24.0 | 25.6 | 24.2 | 26.4 |

PBE0-D4/def2-TZVP-SDD-SMD(1,4-dioxane)//PBE0-D3(BJ)/def2-SVP-SDD
PBE0-D3/def2-TZVP-SDD-SMD(1,4-dioxane)//PBE0-D3(BJ)/def2-SVP-SDD
B3LYP-D4/def2-TZVP-SDD-SMD(1,4-dioxane)//PBE0-D3(BJ)/def2-SVP-SDD
B3LYP-D3/def2-TZVP-SDD-SMD(1,4-dioxane)//PBE0-D3(BJ)/def2-SVP-SDD
M06-D3/def2-TZVP-SDD-SMD(1,4-dioxane)//PBE0-D3(BJ)/def2-SVP-SDD

**Figure S7.** Conformational searches for the first radical attack step at the ester-substituted site of disubstituted BCB **1m** at 338.15K using various functionals. Energies are presented in the unit of kcal mol$^{-1}$.

## Conformers for TS8:

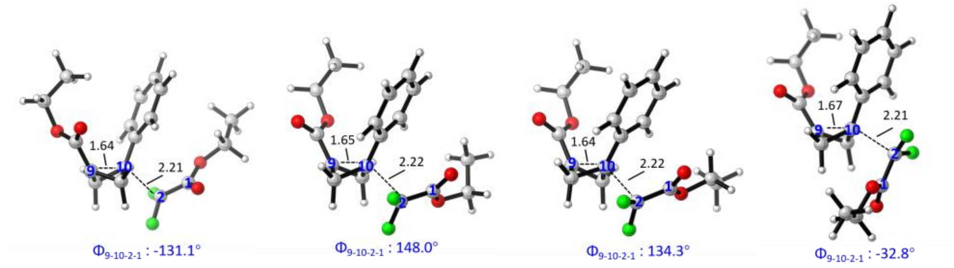


| | TS8-s1 | TS8-s2 | TS8-s3 | TS8-s4 |
|---|---|---|---|---|
| $\Delta G^{\ddagger}$ (kcal mol$^{-1}$) | 15.0 | 15.3 | 16.3 | 16.2 |
| $\Delta G^{\ddagger}$ (kcal mol$^{-1}$) | 15.9 | 16.2 | 17.2 | 17.1 |
| $\Delta G^{\ddagger}$ (kcal mol$^{-1}$) | 15.1 | 15.4 | 16.4 | 16.4 |
| $\Delta G^{\ddagger}$ (kcal mol$^{-1}$) | 16.3 | 16.7 | 17.6 | 17.6 |
| $\Delta G^{\ddagger}$ (kcal mol$^{-1}$) | 25.1 | 25.5 | 26.3 | 27.9 |

PBE0-D4/def2-TZVP-SDD-SMD(1,4-dioxane)//PBE0-D3(BJ)/def2-SVP-SDD
PBE0-D3/def2-TZVP-SDD-SMD(1,4-dioxane)//PBE0-D3(BJ)/def2-SVP-SDD
B3LYP-D4/def2-TZVP-SDD-SMD(1,4-dioxane)//PBE0-D3(BJ)/def2-SVP-SDD
B3LYP-D3/def2-TZVP-SDD-SMD(1,4-dioxane)//PBE0-D3(BJ)/def2-SVP-SDD
M06-D3/def2-TZVP-SDD-SMD(1,4-dioxane)//PBE0-D3(BJ)/def2-SVP-SDD

**Figure S8.** Conformational searches for the first radical attack step at the phenyl-substituted site of disubstituted BCB **1m** at 338.15K using various functionals. Energies are presented in the unit of kcal mol$^{-1}$.

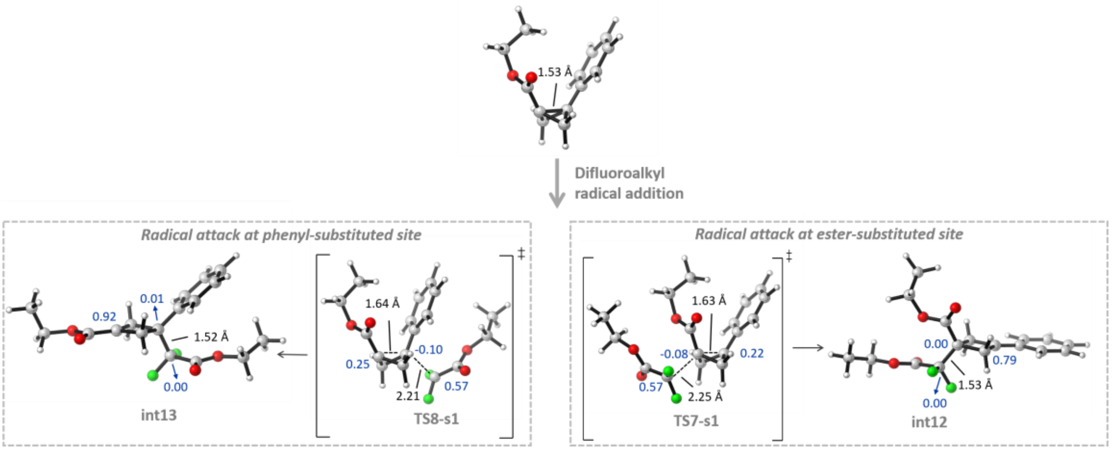


**Figure S9.** Optimized geometries and spin densities of computed structures in the first radical attack step with disubstituted BCB **1m**.

Upon the initial radical attack at the ester-substituted site of disubstituted BCB **1m**, the formed benzyl radical subsequently attacked the ruthenium(III) cyclometalated complex, giving rise to both *major*- and *minor*-isomers through **TS9$^{oss}$** and **TS10$^{oss}$**, respectively. In this process, five distinct conformers were identified, distinguished by different dihedral angles between the bridging C–C bond of the BCB core and the phenylpyridine plane, with consideration of attack orientations from both above and below the aromatic plane (**Figure S10-S11**).

## Conformers for TS9$^{oss}$:

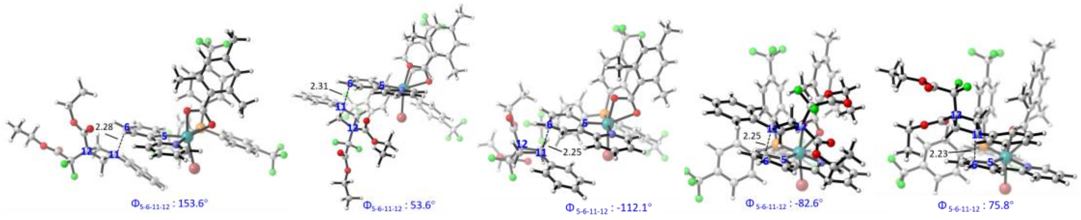


| | TS9-s1 | TS9-s2 | TS9-s3 | TS9-s4 | TS9-s5 |
|---|---|---|---|---|---|
| ΔG$^‡$ (kcal mol$^{-1}$) | 21.5 | 18.2 | 24.1 | 24.7 | 20.4 |
| ΔG$^‡$ (kcal mol$^{-1}$) | 21.3 | 18.2 | 24.1 | 23.9 | 19.8 |
| ΔG$^‡$ (kcal mol$^{-1}$) | 19.9 | 16.5 | 21.9 | 23.0 | 19.1 |
| ΔG$^‡$ (kcal mol$^{-1}$) | 19.4 | 16.2 | 21.6 | 21.7 | 18.2 |
| ΔG$^‡$ (kcal mol$^{-1}$) | 16.8 | 12.8 | 17.8 | 14.0 | 12.3 |

PBE0-D4/def2-TZVP-SDD-SMD(1,4-dioxane)//PBE0-D3(BJ)/def2-SVP-SDD
PBE0-D3/def2 TZVP-SDD-SMD(1,4-dioxane)//PBE0-D3(BJ)/def2-SVP-SDD
B3LYP-D4/def2-TZVP-SDD-SMD(1,4-dioxane)//PBE0-D3(BJ)/def2-SVP-SDD
B3LYP-D3/def2-TZVP-SDD-SMD(1,4-dioxane)//PBE0-D3(BJ)/def2-SVP-SDD
M06/def2-TZVP-SDD-SMD(1,4-dioxane)//PBE0-D3(BJ)/def2-SVP-SDD

**Figure S10.** Conformational searches for the second radical attack step at the arene moiety of the ruthenium complex at the open-shell singlet state for the generation of *major*-product at 338.15K using various functionals. Energies are presented in the unit of kcal mol$^{-1}$.

## Conformers for TS10$^{oss}$:

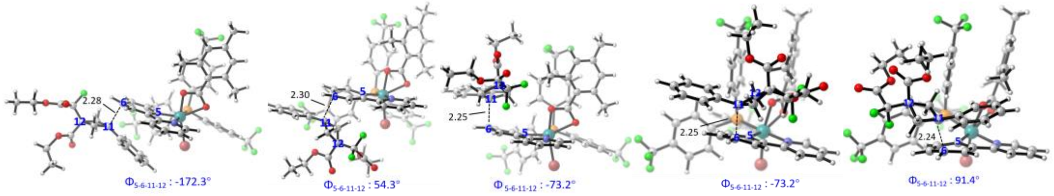


| | TS10-s1 | TS10-s2 | TS10-s3 | TS10-s4 | TS10-s5 |
|---|---|---|---|---|---|
| ΔG$^{‡}$ (kcal mol$^{-1}$) | 20.8 | 19.0 | 21.9 | 22.0 | 23.7 |
| ΔG$^{‡}$ (kcal mol$^{-1}$) | 20.8 | 18.7 | 22.3 | 21.4 | 23.0 |
| ΔG$^{‡}$ (kcal mol$^{-1}$) | 19.0 | 17.3 | 20.3 | 20.8 | 22.2 |
| ΔG$^{‡}$ (kcal mol$^{-1}$) | 18.7 | 16.7 | 20.7 | 19.9 | 21.1 |
| ΔG$^{‡}$ (kcal mol$^{-1}$) | 16.4 | 12.7 | 17.6 | 15.5 | 13.6 |

PBE0-D4/def2-TZVP-SDD-SMD(1,4-dioxane)//PBE0-D3(BJ)/def2-SVP-SDD
PBE0-D3/def2 TZVP-SDD-SMD(1,4-dioxane)//PBE0-D3(BJ)/def2-SVP-SDD
B3LYP-D4/def2-TZVP-SDD-SMD(1,4-dioxane)//PBE0-D3(BJ)/def2-SVP-SDD
B3LYP-D3/def2-TZVP-SDD-SMD(1,4-dioxane)//PBE0-D3(BJ)/def2-SVP-SDD
M06/def2-TZVP-SDD-SMD(1,4-dioxane)//PBE0-D3(BJ)/def2-SVP-SDD

**Figure S11.** Conformational searches for the second radical attack step at the arene moiety of the ruthenium complex at the open-shell singlet state for the generation of *minor*-product at 338.15K using various functionals. Energies are presented in the unit of kcal mol$^{-1}$.

Based on the Boltzmann distribution of these transition state conformers, Boltzmann-weighted Gibbs free energies $\Delta G_{\mathrm{ave}}$ for each diastereomer were calculated and presented in **Table S3.** The final Boltzmann populations of **TS9$^{oss}$** and **TS10$^{oss}$** with different functionals showed that both PBE0 and B3LYP functionals combined with D4 dispersion corrections provided a simulated ratio (3.3:1) closely aligning with the experimental ratio (2.8:1).

**Table S3.** Boltzmann-weighted Gibbs free energies and the corresponding ratios of the two diastereomers for the reaction with disubstituted BCB **1m** with different functionals.

| | PBE0-D4 | PBE0-D3 | PBE0-noD | B3LYP-D4 | B3LYP-D3 | B3LYP-noD | M06 |
|---|---|---|---|---|---|---|---|
| $\boldsymbol{\Delta G_w^{‡}}$(TS9$^{oss}$) | 18.3 | 18.4 | 51.3 | 16.6 | 16.3 | 43.0 | 12.6 |
| $\boldsymbol{\Delta G_w^{‡}}$(TS10$^{oss}$) | 19.1 | 18.9 | 52.2 | 17.4 | 16.8 | 42.7 | 12.9 |
| ΔΔG$^{‡}$ | -0.8 | -0.5 | -0.9 | -0.8 | -0.5 | 0.3 | -0.3 |
| Calculated ratio | **3.3/1** | 2.1/1 | 3.8/1 | **3.3/1** | 2.1/1 | 1/1.6 | 1.6/1 |
| Experimental ratio | | | | 2.8/1 | | | |

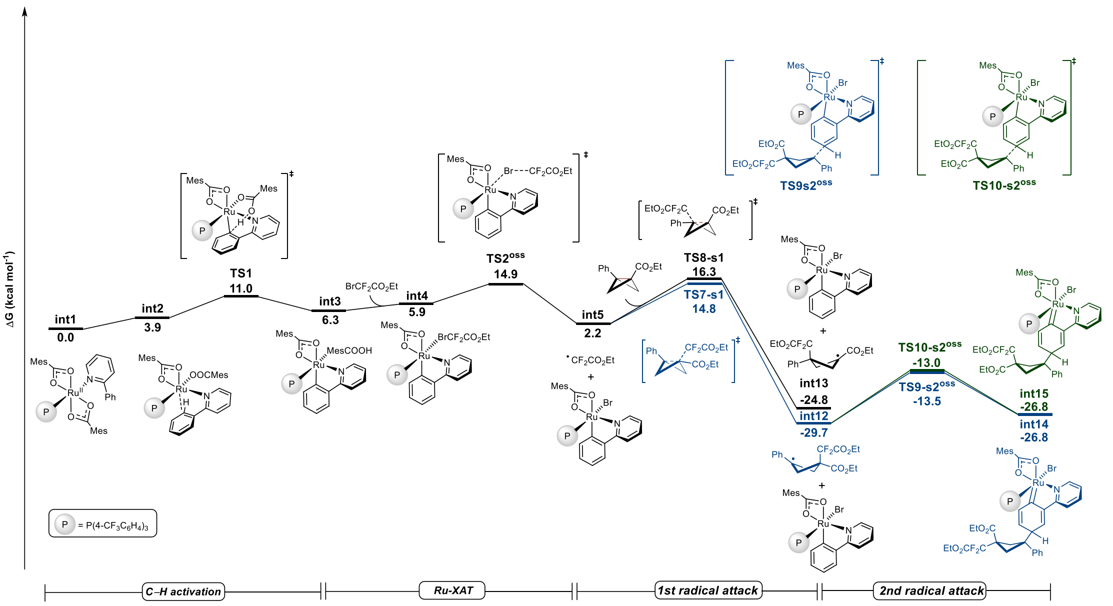


**Figure S12.** Computed relative Gibbs free energies ($\Delta G_{338.15}$) in kcal mol$^{-1}$ for ruthenium-catalyzed *meta*-C–H alkylation reaction with BCB **1m** at the B3LYP-D3/def2-TZVP-SMD(1,4-dioxane)//PBE0-D3(BJ)/def2-SVP level of theory.

### 7.2.3. Reaction with monosubstituted BCB 1k.

For substrates exhibiting substantial diastereoselectivity in product formation, such as compound **40**, computational studies were also employed to evaluate the two sequential radical attacks. As shown in **Figures S13** and **S14**, considering all relevant conformers involved, the radical attack at the unsubstituted site showed a higher preference over the attack at the amide-substituted position. This selectivity could be potentially attributed to the steric repulsions and electron-withdrawing nature of the difluoroalkyl radical. At this stage, the slightly elongated C–C bridge bond within the BCB backbone and relatively longer bond distance between the radical and BCB species in **TS11-s1**, indicated the radical attacked at the unsubstituted site involved an earlier transition state (**Figure S15**). Subsequently, the generated tertiary radical attack at the phenyl group of ruthenium(III) species showed a stronger preference for *cis*-product formation over *trans*-product formation analyzed with the PBE0, B3LYP, and M06 functionals (**Figure S16-S17**). Additionally, the calculated ratio, derived from Boltzmann-weighted Gibbs free energies $\Delta G_w^{\ddagger}$, considering the contributions of each conformer, further indicated that *cis*-diastereomer generation is energetically much more favorable over the *trans*-product formation (see **Table S4**). This theoretical

finding is consistent with experimental observation.

## Conformers for TS11:

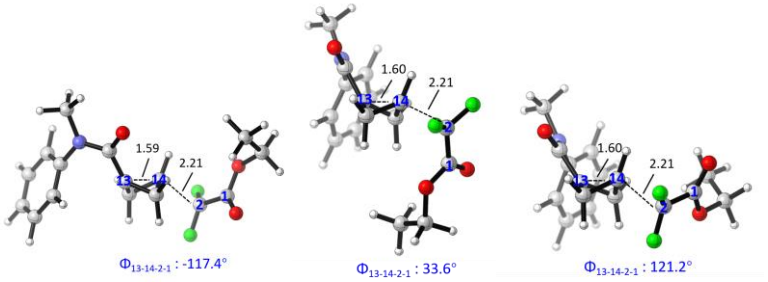


| | TS11-s1 | TS11-s2 | TS11-s3 |
|---|---|---|---|
| $\Delta G^{\ddagger}$ (kcal mol$^{-1}$) | 13.8 | 14.8 | 14.1 |
| $\Delta G^{\ddagger}$ (kcal mol$^{-1}$) | 14.5 | 15.4 | 14.7 |
| $\Delta G^{\ddagger}$ (kcal mol$^{-1}$) | 14.7 | 15.6 | 15.0 |
| $\Delta G^{\ddagger}$ (kcal mol$^{-1}$) | 15.7 | 16.4 | 15.8 |
| $\Delta G^{\ddagger}$ (kcal mol$^{-1}$) | 24.7 | 25.9 | 25.1 |

PBE0-D4/def2-TZVP-SDD-SMD(1,4-dioxane)//PBE0-D3(BJ)/def2-SVP-SDD
PBE0-D3/def2-TZVP-SDD-SMD(1,4-dioxane)//PBE0-D3(BJ)/def2-SVP-SDD
B3LYP-D4/def2-TZVP-SDD-SMD(1,4-dioxane)//PBE0-D3(BJ)/def2-SVP-SDD
B3LYP-D3/def2-TZVP-SDD-SMD(1,4-dioxane)//PBE0-D3(BJ)/def2-SVP-SDD
M06-D3/def2-TZVP-SDD-SMD(1,4-dioxane)//PBE0-D3(BJ)/def2-SVP-SDD

**Figure S13.** Conformational searches for the first radical attack step at the unsubstituted site of amide-functionalized BCB **1k** at 338.15K using various functionals. Energies are presented in the unit of kcal mol$^{-1}$.

## Conformers for TS12:

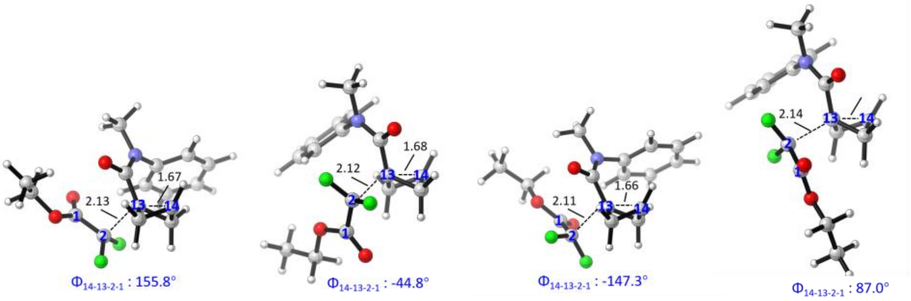


| | TS12-s1 | TS12-s2 | TS12-s3 | TS12-s4 |
|---|---|---|---|---|
| $\Delta G^{\ddagger}$ (kcal mol$^{-1}$) | 20.2 | 21.1 | 21.0 | 21.7 |
| $\Delta G^{\ddagger}$ (kcal mol$^{-1}$) | 21.4 | 22.0 | 22.0 | 22.7 |
| $\Delta G^{\ddagger}$ (kcal mol$^{-1}$) | 19.8 | 21.1 | 20.8 | 21.5 |
| $\Delta G^{\ddagger}$ (kcal mol$^{-1}$) | 21.4 | 22.3 | 22.1 | 22.9 |
| $\Delta G^{\ddagger}$ (kcal mol$^{-1}$) | 31.3 | 32.2 | 31.2 | 33.3 |

PBE0-D4/def2-TZVP-SDD-SMD(1,4-dioxane)//PBE0-D3(BJ)/def2-SVP-SDD
PBE0-D3/def2-TZVP-SDD-SMD(1,4-dioxane)//PBE0-D3(BJ)/def2-SVP-SDD
B3LYP-D4/def2-TZVP-SDD-SMD(1,4-dioxane)//PBE0-D3(BJ)/def2-SVP-SDD
B3LYP-D3/def2-TZVP-SDD-SMD(1,4-dioxane)//PBE0-D3(BJ)/def2-SVP-SDD
M06-D3/def2-TZVP-SDD-SMD(1,4-dioxane)//PBE0-D3(BJ)/def2-SVP-SDD

**Figure S14.** Conformational searches for the first radical attack step at the amide-substituted site of amide-functionalized BCB **1k** at 338.15K using various functionals. Energies are presented in the unit of kcal mol$^{-1}$.

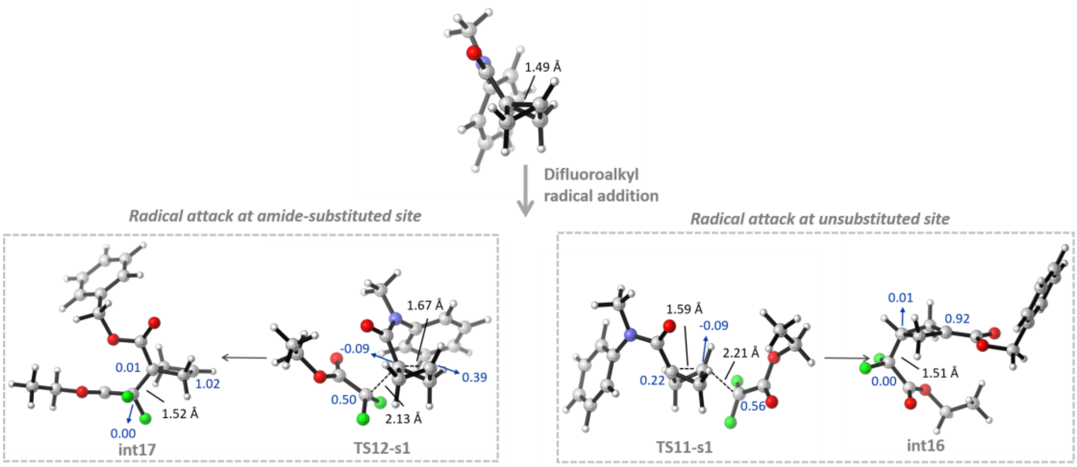


**Figure S15.** Optimized geometries and spin densities of computed structures in the first radical attack step considering amide-functionalized BCB **1k**.

## Conformers for TS13$^{oss}$:

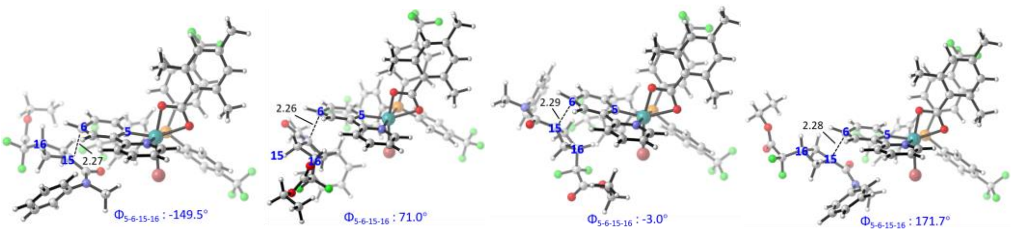


| | TS13-s1 | TS13-s2 | TS13-s3 | TS13-s4 |
|---|---|---|---|---|
| ΔG‡ (kcal mol$^{-1}$) | 23.5 | 19.4 | 20.2 | 20.9 |
| ΔG‡ (kcal mol$^{-1}$) | 23.7 | 18.9 | 19.4 | 20.7 |
| ΔG‡ (kcal mol$^{-1}$) | 21.8 | 18.5 | 18.5 | 19.4 |
| ΔG‡ (kcal mol$^{-1}$) | 21.8 | 17.5 | 17.5 | 19.0 |
| ΔG‡ (kcal mol$^{-1}$) | 17.8 | 14.8 | 12.5 | 15.3 |

PBE0-D4/def2-TZVP-SDD-SMD(1,4-dioxane)//PBE0-D3(BJ)/def2-SVP-SDD
PBE0-D3/def2 TZVP-SDD-SMD(1,4-dioxane)//PBE0-D3(BJ)/def2-SVP-SDD
B3LYP-D4/def2-TZVP-SDD-SMD(1,4-dioxane)//PBE0-D3(BJ)/def2-SVP-SDD
B3LYP-D3/def2-TZVP-SDD-SMD(1,4-dioxane)//PBE0-D3(BJ)/def2-SVP-SDD
M06-D3/def2-TZVP-SDD-SMD(1,4-dioxane)//PBE0-D3(BJ)/def2-SVP-SDD

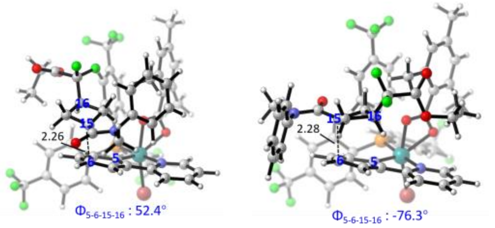


| TS13-s5 | TS13-s6 |
|---|---|
| 17.6 | 20.7 |
| 16.6 | 19.7 |
| 16.3 | 19.1 |
| 14.7 | 17.3 |
| 9.7 | 10.0 |

**Figure S16.** Conformational searches for the second radical attack step at the arene moiety of the ruthenium complex at the open-shell singlet state for the generation of *cis*-product at 338.15K using various functionals. Energies are presented in the unit of kcal mol$^{-1}$.

## Conformers for TS14$^{oss}$:

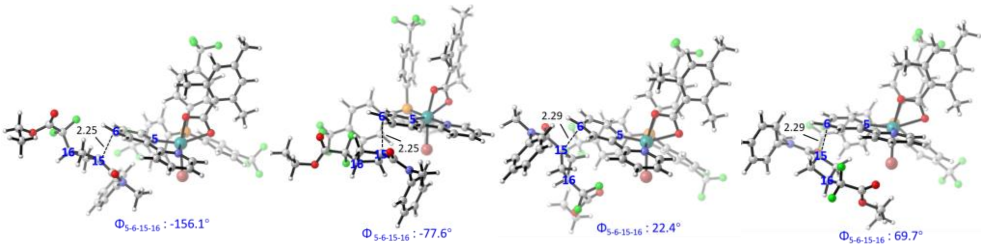


| | TS14-s1 | TS14-s2 | TS14-s3 | TS14-s4 |
|---|---|---|---|---|
| ΔG‡ (kcal mol$^{-1}$) | 22.0 | 20.2 | 23.0 | 20.7 |
| ΔG‡ (kcal mol$^{-1}$) | 21.9 | 20.5 | 22.9 | 20.8 |
| ΔG‡ (kcal mol$^{-1}$) | 20.5 | 18.5 | 22.0 | 18.2 |
| ΔG‡ (kcal mol$^{-1}$) | 20.1 | 18.7 | 21.7 | 18.2 |
| ΔG‡ (kcal mol$^{-1}$) | 16.7 | 14.4 | 17.3 | 14.7 |

PBE0-D4/def2-TZVP-SDD-SMD(1,4-dioxane)//PBE0-D3(BJ)/def2-SVP-SDD
PBE0-D3/def2 TZVP-SDD-SMD(1,4-dioxane)//PBE0-D3(BJ)/def2-SVP-SDD
B3LYP-D4/def2-TZVP-SDD-SMD(1,4-dioxane)//PBE0-D3(BJ)/def2-SVP-SDD
B3LYP-D3/def2-TZVP-SDD-SMD(1,4-dioxane)//PBE0-D3(BJ)/def2-SVP-SDD
M06-D3/def2-TZVP-SDD-SMD(1,4-dioxane)//PBE0-D3(BJ)/def2-SVP-SDD

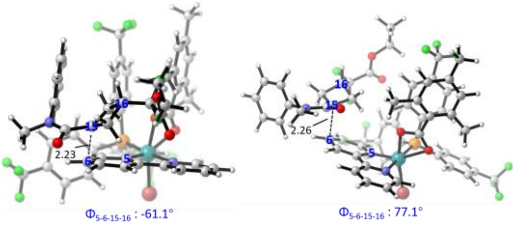


| TS14-s5 | TS14-s6 |
|---|---|
| 22.2 | 22.2 |
| 21.5 | 21.9 |
| 20.9 | 20.5 |
| 19.9 | 19.9 |
| 14.1 | 14.0 |

**Figure S17.** Conformational searches for the second radical attack step at the arene moiety of the ruthenium complex at the open-shell singlet state for the generation of *trans*-product at 338.15K using various functionals. Energies are presented in the unit of kcal mol$^{-1}$.

**Table S4.** Boltzmann-weighted Gibbs free energies and the corresponding ratios of the two diastereomers for the reaction with monosubstituted BCB **1k** with different functionals.

| | PBE0-D4 | PBE0-D3 | PBE0-noD | B3LYP-D4 | B3LYP-D3 | B3LYP-noD | M06 |
|---|---|---|---|---|---|---|---|
| $\boldsymbol{\Delta G_w^{\ddagger}}$(TS13$^{oss}$) | 17.8 | 16.7 | 35.0 | 16.5 | 14.9 | 41.4 | 9.8 |
| $\boldsymbol{\Delta G_w^{\ddagger}}$(TS14$^{oss}$) | 20.6 | 20.9 | 37.1 | 18.4 | 18.6 | 43.2 | 14.2 |
| ΔΔG‡ | -2.8 | -4.2 | -2.1 | -1.9 | -3.7 | -1.8 | -4.4 |
| Calculated ratio | 64.5/1 | 518/1 | 22.8/1 | 16.9/1 | 246/1 | 14.6/1 | 698/1 |
| Experimental ratio | | | | 20.0/1 | | | |

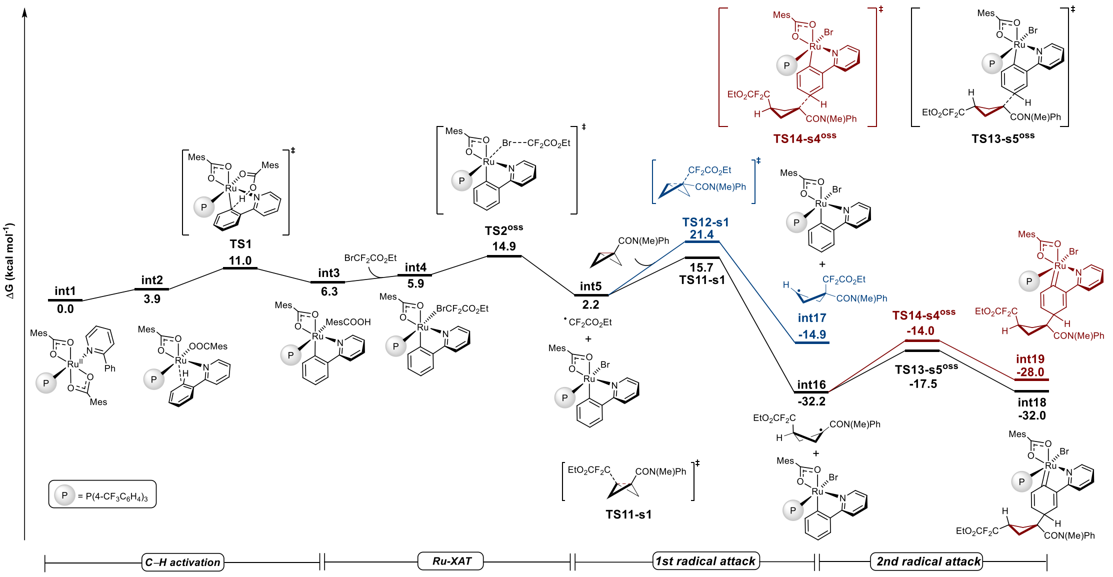


**Figure S18.** Computed relative Gibbs free energies ($\Delta G_{338.15}$) in kcal mol$^{-1}$ for ruthenium-catalyzed *meta*-C–H alkylation reaction with BCB **1k** at the B3LYP-D3/def2-TZVP-SMD(1,4-dioxane)//PBE0-D3(BJ)/def2-SVP level of theory.

### 7.2.4. Table of Energies

**Table S5.** Calculated electronic energies at the B3LYP-D3(BJ)/def2-TZVP-SMD (1,4-dioxane) level of theory and Gibbs free energies with dispersion corrections for all the structures in the reaction with mono-substituted BCB **1a**. (all in Hartree).

| Structure | Electronic Energy | Total Gibbs Free Energy | Frequency |
|---|---|---|---|
| **int1** | -3699.812482 | -3699.095851 | 8.7175 |
| **int2** | -3699.808679 | -3699.089557 | 12.0439 |
| **TS1** | -3699.792727 | -3699.078363 | -1006.2231 |
| **int3** | -3699.803608 | -3699.085756 | 9.6342 |
| **int4** | -3679.841729 | -3679.231584 | 5.9201 |
| **TS2$^{oss}$** | -3679.831286 | -3679.222120 | -270.3159 |
| **int5** | -3174.073686 | -3173.544095 | 8.0219 |
| **TS3-s1** | -1120.887695 | -1120.646676 | -820.9244 |
| **TS3-s2** | -1120.926142 | -1120.684933 | -460.5751 |
| **TS3-s3** | -1120.925003 | -1120.684809 | -463.5763 |
| **TS3-s4** | -1120.924853 | -1120.687170 | -537.3960 |
| **TS3-s5** | -1120.924931 | -1120.686535 | -538.5385 |
| **TS4-s1** | -1120.882538 | -1120.641229 | -703.7227 |
| **TS4-s2** | -1120.922732 | -1120.681960 | -657.1695 |
| **TS4-s3** | -1120.921295 | -1120.680564 | -638.2411 |
| **TS4-s4** | -1120.921460 | -1120.681130 | -666.0934 |
| **TS4-s5** | -1120.918991 | -1120.680360 | -693.1426 |
| **int6** | -1121.003236 | -1120.758452 | 16.5156 |
| **int7** | -1120.981791 | -1120.741679 | 8.0904 |

| | | | |
|---|---|---|---|
| **TS5-s1$^{oss}$** | -4295.088329 | -4294.279435 | -395.4788 |
| **TS5-s2$^{oss}$** | -4295.084035 | -4294.279299 | -403.2749 |
| **TS5-s3$^{oss}$** | -4295.087836 | -4294.281106 | -398.3302 |
| **TS5-s4$^{oss}$** | -4295.090129 | -4294.279649 | -408.5036 |
| **TS5-s5$^{oss}$** | -4295.089342 | -4294.281560 | -383.7089 |
| **TS6-s1$^{oss}$** | -4295.083733 | -4294.277826 | -366.1006 |
| **TS6-s2$^{oss}$** | -4295.085699 | -4294.276265 | -373.9054 |
| **TS6-s3$^{oss}$** | -4295.089659 | -4294.279353 | -331.3547 |
| **TS6-s4$^{oss}$** | -4295.086066 | -4294.276471 | -373.2975 |
| **TS6-s5$^{oss}$** | -4295.083084 | -4294.275786 | -380.4654 |
| **int8** | -4295.119077 | -4294.306255 | 10.2385 |
| **int9** | -4295.118344 | -4294.309331 | 7.9898 |
| **int10** | -4281.202347 | -4280.393220 | 8.4487 |
| **int11** | -4281.196320 | -4280.395212 | 7.6419 |
| **BrCF$_2$CO$_2$Et** | -519.0795953 | -519.0293513 | 39.1203 |
| **CF$_2$CO$_2$Et-radical** | -248.3669726 | -248.2413086 | 67.1204 |
| **MesCOOH** | -539.0324782 | -538.8793722 | 15.8559 |
| **BCB** | -615.176318 | -615.010776 | 21.1847 |

**Table S6.** Calculated electronic energies at the B3LYP-D3(BJ)/def2-TZVP-SMD (1,4-dioxane) level of theory and Gibbs free energies with dispersion corrections for all the structures in the reaction with disubstituted BCB **1m**. (all in Hartree).

| Structure | Electronic Energy | Total Gibbs Free Energy | Frequency |
|---|---|---|---|
| **TS7-s1** | -1160.275457 | -1160.007624 | -494.0650 |
| **TS7-s2** | -1160.273309 | -1160.005820 | -510.8990 |
| **TS7-s3** | -1160.274234 | -1160.006850 | -482.9898 |
| **TS7-s4** | -1160.271639 | -1160.004585 | -534.2686 |
| **TS8-s1** | -1160.274150 | -1160.005309 | -564.4964 |
| **TS8-s2** | -1160.271656 | -1160.004687 | -571.4633 |
| **TS8-s3** | -1160.270989 | -1160.003189 | -567.1970 |
| **TS8-s4** | -1160.269719 | -1160.003163 | -627.5734 |
| **int12** | -1160.346090 | -1160.078546 | 8.6069 |
| **int13** | -1160.340216 | -1160.070725 | 15.2284 |
| **TS9-s1$^{oss}$** | -4334.421295 | -4333.591768 | -405.6885 |
| **TS9-s2$^{oss}$** | -4334.428061 | -4333.596855 | -375.8676 |
| **TS9-s3$^{oss}$** | -4334.425185 | -4333.588289 | -434.6709 |
| **TS9-s4$^{oss}$** | -4334.421830 | -4333.588058 | -440.5622 |
| **TS9-s5$^{oss}$** | -4334.427750 | -4333.593693 | -448.8159 |
| **TS10-s1$^{oss}$** | -4334.420328 | -4333.592865 | -378.5693 |
| **TS10-s2$^{oss}$** | -4334.428563 | -4333.596018 | -360.2427 |
| **TS10-s3$^{oss}$** | -4334.420952 | -4333.589597 | -425.6857 |
| **TS10-s4$^{oss}$** | -4334.421279 | -4333.590913 | -420.7607 |

| | | | |
|---|---|---|---|
| **TS10-s5**$^{oss}$ | -4334.421397 | -4333.588938 | -441.5726 |
| **int14** | -4334.453883 | -4333.618045 | 9.4803 |
| **int15** | -4334.456412 | -4333.618010 | 9.5720 |
| **di-BCB** | -654.522062 | -654.3295230 | 35.0230 |

**Table S7.** Calculated electronic energies at the B3LYP-D3(BJ)/def2-TZVP-SMD (1,4-dioxane) level of theory and Gibbs free energies with dispersion corrections for all the structures in the reaction with mono- substituted BCB **1k**. (all in Hartree).

| **Structure** | **Electronic Energy** | **Total Gibbs Free Energy** | **Frequency** |
|---|---|---|---|
| **TS11-s1** | -1101.044552 | -1100.794343 | -513.7335 |
| **TS11-s2** | -1101.043680 | -1100.793158 | -553.1600 |
| **TS11-s3** | -1101.044944 | -1100.794037 | -515.9488 |
| **TS12-s1** | -1101.039227 | -1100.785136 | -671.1049 |
| **TS12-s2** | -1101.036408 | -1100.783685 | -713.5538 |
| **TS12-s3** | -1101.038491 | -1100.784005 | -685.3284 |
| **TS12-s4** | -1101.034556 | -1100.782841 | -695.3724 |
| **int16** | -1101.123129 | -1100.870565 | 15.4548 |
| **int17** | -1101.097235 | -1100.843054 | 14.8308 |
| **TS13-s1**$^{oss}$ | -4275.198231 | -4274.379865 | -263.3307 |
| **TS13-s2**$^{oss}$ | -4275.205765 | -4274.386803 | -413.2753 |
| **TS13-s3**$^{oss}$ | -4275.202208 | -4274.386797 | -428.9938 |
| **TS13-s4**$^{oss}$ | -4275.198777 | -4274.384427 | -424.6329 |
| **TS13-s5**$^{oss}$ | -4275.207492 | -4274.391221 | -434.9790 |
| **TS13-s6**$^{oss}$ | -4275.206389 | -4274.387074 | -416.8464 |
| **TS14-s1**$^{oss}$ | -4275.198318 | -4274.382673 | -451.8189 |
| **TS14-s2**$^{oss}$ | -4275.203161 | -4274.384910 | -432.8178 |
| **TS14-s3**$^{oss}$ | -4275.195933 | -4274.380002 | -373.2385 |
| **TS14-s4**$^{oss}$ | -4275.202394 | -4274.385645 | -406.2606 |
| **TS14-s5**$^{oss}$ | -4275.200877 | -4274.383001 | -455.0885 |
| **TS14-s6**$^{oss}$ | -4275.199677 | -4274.382905 | -420.3695 |
| **int18** | -4275.236131 | -4274.414380 | 10.3103 |
| **int19** | -4275.233234 | -4274.407955 | 9.3214 |
| **CON-BCB** | -595.2948546 | -595.1175486 | 37.7081 |

# 8. References


1. Agasti, S., Beltran, F., Pye, E. et al. A catalytic alkene insertion approach to bicyclo[2.1.1]hexane bioisosteres. *Nat. Chem.* **15**, 535–541 (2023).

2. Ni, D. et al. Intermolecular formal cycloaddition of indoles with bicyclo[1.1.0]butanes by Lewis acid catalysis. *Angew. Chem. Int. Ed.* **62**, e202308606 (2023).

3. Tang, L. et al. Silver-catalyzed dearomative [2π+2σ] cycloadditions of indoles with bicyclobutanes: access to indoline fused bicyclo[2.1.1]hexanes *Angew. Chem. Int. Ed.* **62**, e202310066 (2022).

4. Gianatassio, R. et al. Strain-release amination. *Science* **351**, 241–246 (2016).

5. Wang, Y., Chen, S., Chen, X., Zangarelli, A. & Ackermann, L. Photo-induced ruthenium-catalyzed double remote $C(sp^2)-H/C(sp^3)-H$ functionalizations by radical relay. *Angew. Chem. Int. Ed.* **61**, e202205562 (2022).

6. Tan, G., Schrader, M. L., Daniliuc, C., Strieth-Kalthoff, F. & Glorius, F. C–H activation based copper-catalyzed one-shot synthesis of N,O-bidentate organic difluoroboron complexes. *Angew. Chem. Int. Ed.* **59**, 21541–21545 (2020).

7. Kong, W., Yu, C., An, H. & Song, Q. Copper-catalyzed intermolecular reductive radical difluoroalkylation–thiolation of aryl alkenes. *Org. Lett.* **20**, 4975–4978 (2018).

8. Frisch, M. J., Trucks, G. W., Schlegel, H. B., Scuseria, G. E., Robb, M. A., Cheeseman, J. R., Scalmani, G., Barone, V., Petersson, G. A., Nakatsuji, H., Li, X., Caricato, M., Marenich, A. V., Bloino, J., Janesko, B. G., Gomperts, R., Mennucci, B., Hratchian, H. P., Ortiz, J. V., Izmaylov, A. F., Sonnenberg, J. L., Williams-Young, D., Ding, F., Lipparini, F., Egidi, F., Goings, J., Peng, B., Petrone, A., Henderson, T., Ranasinghe, D., Zakrzewski, V. G., Gao, J., Rega, N., Zheng, G., Liang, W., Hada, M., Ehara, M., Toyota, K., Fukuda, R., Hasegawa, J., Ishida, M., Nakajima, T., Honda, Y., Kitao, O., Nakai, H., Vreven, T., Throssell, K., Montgomery, Jr., J. A., Peralta, J. E., Ogliaro, F., Bearpark, M. J., Heyd, J. J., Brothers, E. N., Kudin, K. N., Staroverov, V. N., Keith, T. A., Kobayashi, R., Normand, J., Raghavachari, K., Rendell, A. P., Burant, J. C., Iyengar, S. S., Tomasi, J., Cossi, M., Millam, J. M., Klene, M., Adamo, C., Cammi, R., Ochterski, J. W., Martin, R. L., Morokuma, K., Farkas, O., Foresman, J. B. & Fox, D. J. Gaussian 16 Rev. A.03, Wallingford, CT, 2016.

9. Adamo, C. & Barone, V. Toward reliable density functional methods without adjustable parameters: The PBE0 model. *J. Chem. Phys.* **110**, 6158–6170 (1999).

10. Ernzerhof, M. & Scuseria, G. E. Assessment of the Perdew–Burke–Ernzerhof exchange-correlation functional. *J. Chem. Phys.* **110**, 5029–5036 (1999).

11. Grimme, S., Ehrlich, S. & Goerigk, L. Effect of the damping function in dispersion corrected density functional theory. *J. Comput. Chem.* **32**, 1456–1465 (2011).

12. Grimme, S., Antony, J., Ehrlich, S. & Krieg, H. A consistent and accurate ab initio parametrization of density functional dispersion correction (DFT-D) for the 94 elements H-Pu. *J. Chem. Phys.* **132**, 154104 (2010).

13. Weigend, F. Accurate Coulomb-fitting basis sets for H to Rn. *Phys. Chem. Chem. Phys.* **8**, 1057–1065 (2006).

14. Weigend, F. & Ahlrichs, R. Balanced basis sets of split valence, triple zeta valence and quadruple zeta valence quality for H to Rn: Design and assessment of accuracy. *Phys. Chem. Chem. Phys.* **7**, 3297–3305 (2005).

15. Martin, J. M. L. & Sundermann, Correlation consistent valence basis sets for use with the Stuttgart–Dresden–Bonn relativistic effective core potentials: The atoms Ga–Kr and In–Xe. *J. Chem. Phys.* **114**, 3408–3420 (2001).

16. Dolg, M., Wedig, U., Stoll, H. & Preuss, H. Energy-adjusted ab initio pseudopotentials for the first row transition elements. *J. Chem. Phys.* **86**, 866–872 (1987).

17. Becke, A. D. Density-Functional Thermochemistry. III. The Role of Exact Exchange. *J. Chem. Phys*. **98**, 5648–5652 (1993).

18. Lee, C., Yang, W. & Parr, R. G. Development of the Colle-Salvetti Correlation-Energy Formula into a Functional of the Electron Density. *Phys. Rev. B* **37**, 785–789 (1988).

19. Zhao, Y. & Truhlar, D. G. Density functionals with broad applicability in chemistry. *Acc. Chem. Res*. **41**, 157–167 (2008).

20. Marenich, A. V., Cramer, C. J. & Truhlar, D. G. Universal Solvation Model Based on Solute Electron Density and on a Continuum Model of the Solvent Defined by the Bulk Dielectric Constant and Atomic Surface Tensions. *J. Phys. Chem. B* **113**, 6378–6396 (2009).

# 9. NMR Spectra

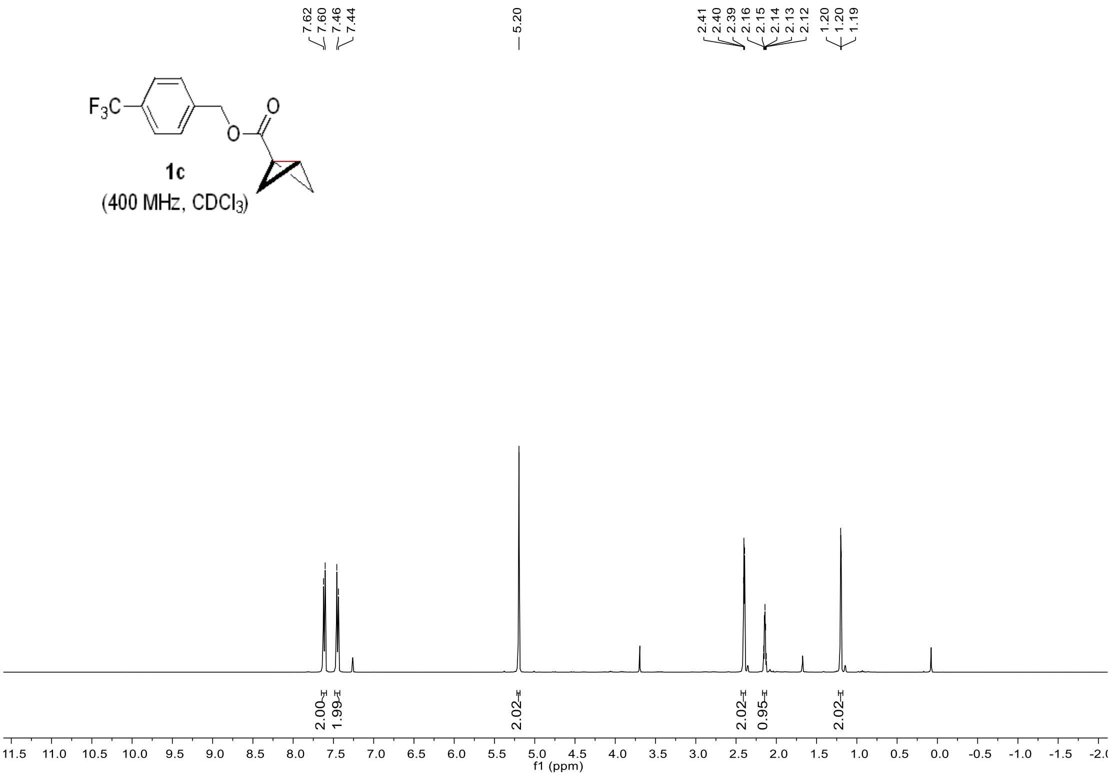


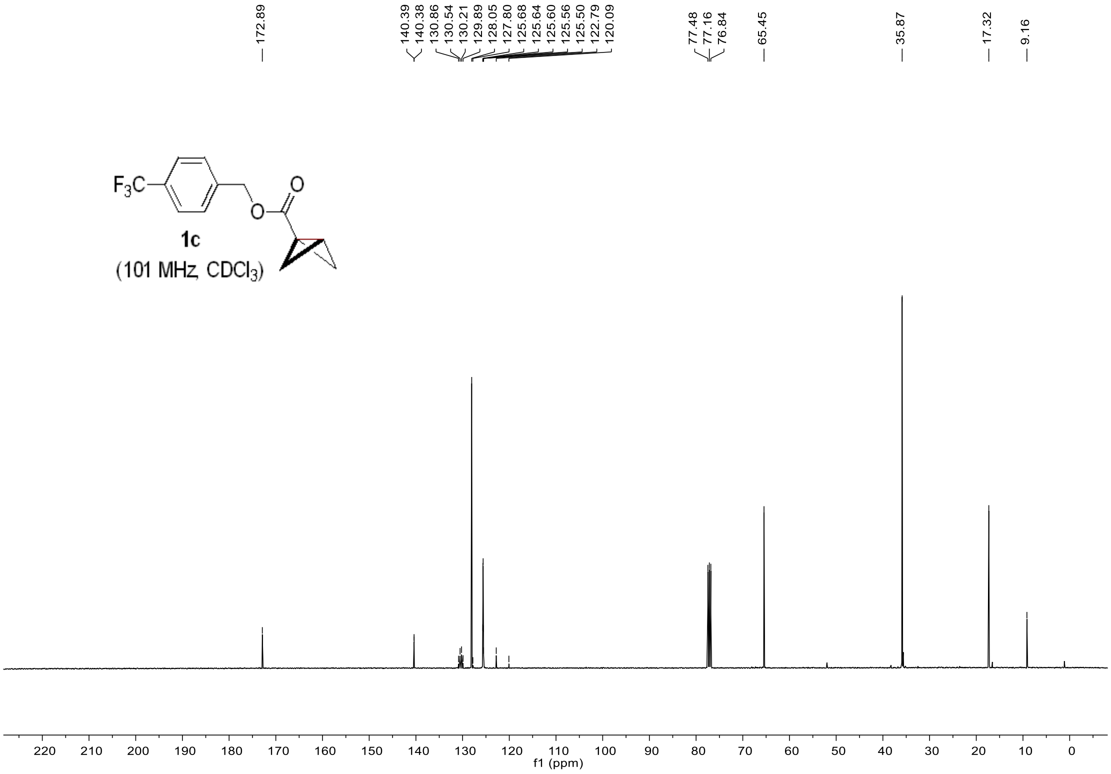

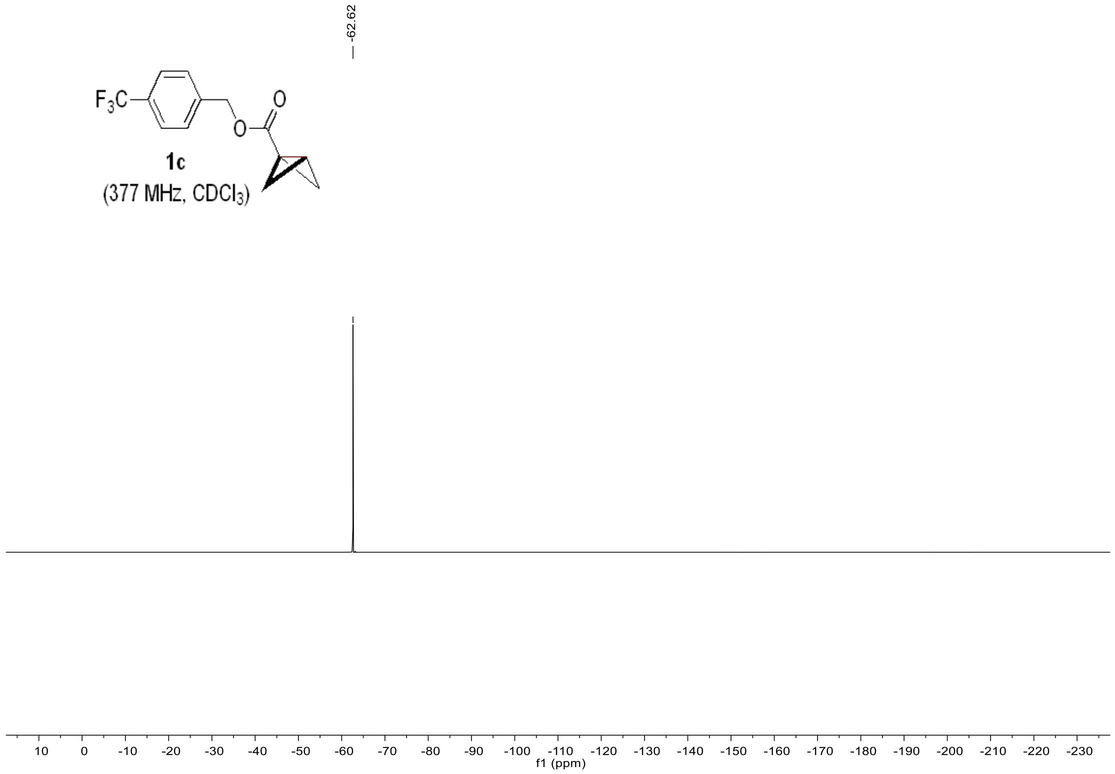
-62.62
$F_3C$
O
O
1c
(377 MHz, $CDCl_3$)
f1 (ppm)

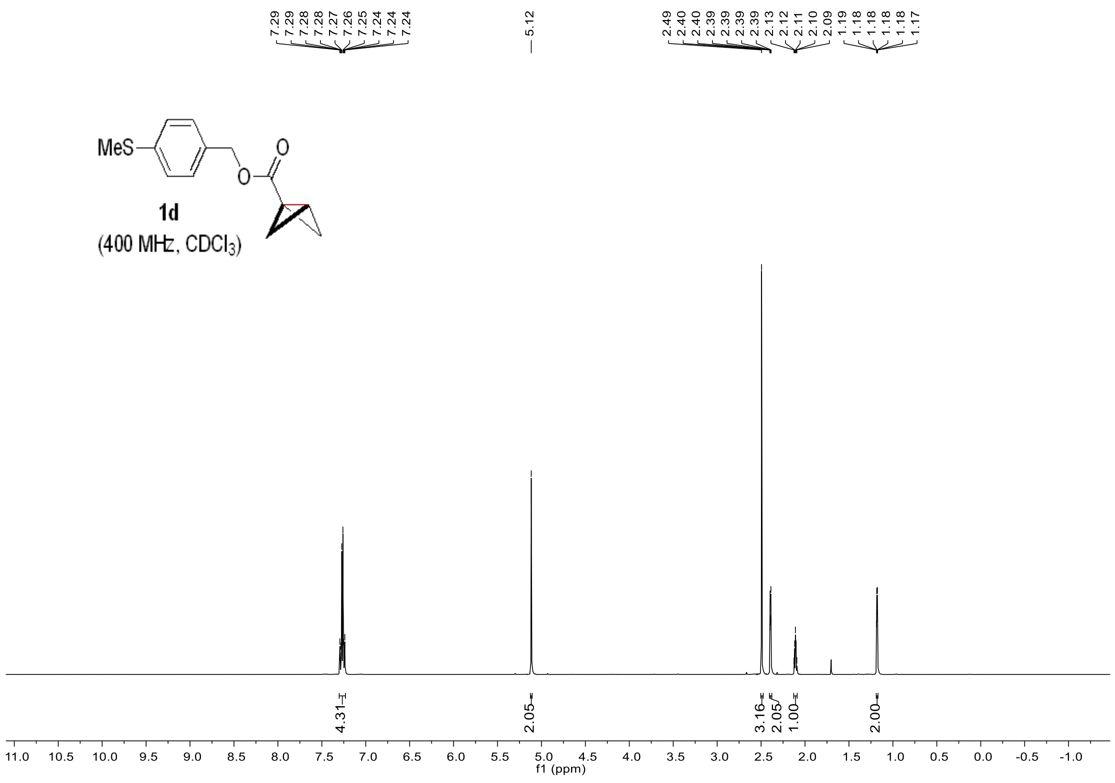
7.29 7.29 7.28 7.28 7.27 7.26 7.25 7.24 7.24 7.24
5.12
2.49 2.40 2.40 2.39 2.39 2.39 2.39 2.13 2.12 2.11 2.10 2.09 1.19 1.18 1.18 1.18 1.18 1.17
MeS
O
O
1d
(400 MHz, $CDCl_3$)
4.31
2.05
3.16
2.05
1.00
2.00
f1 (ppm)

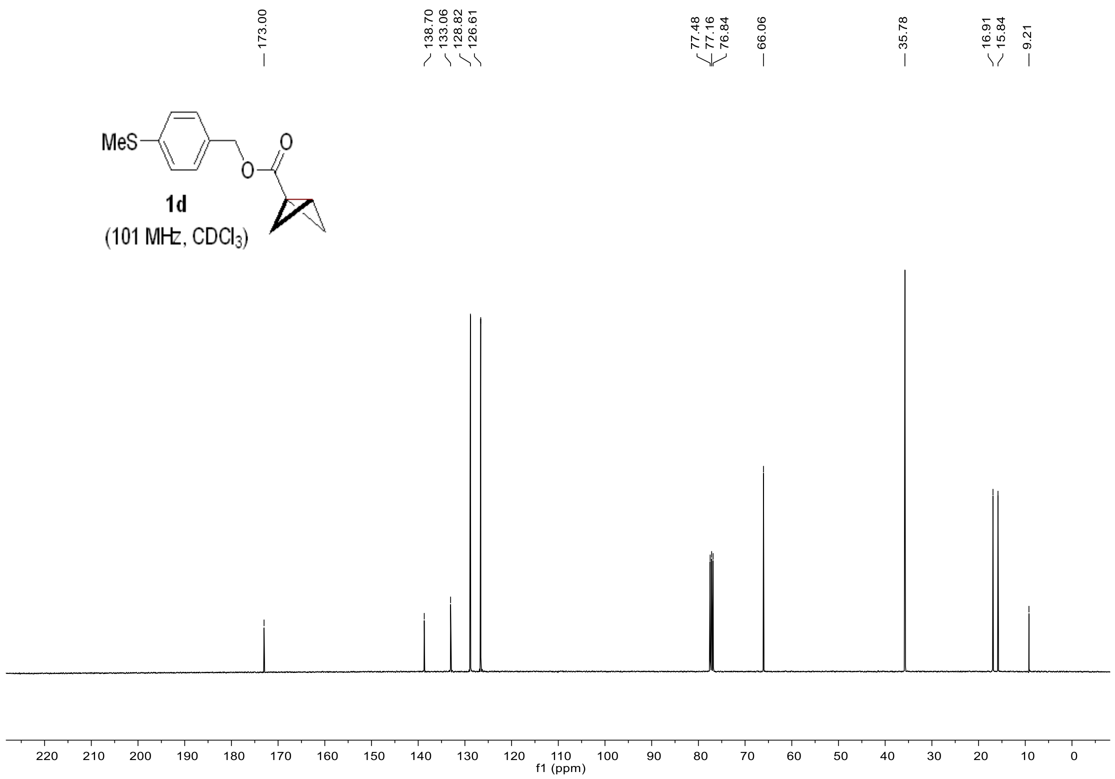


**1d**
(101 MHz, $CDCl_3$)

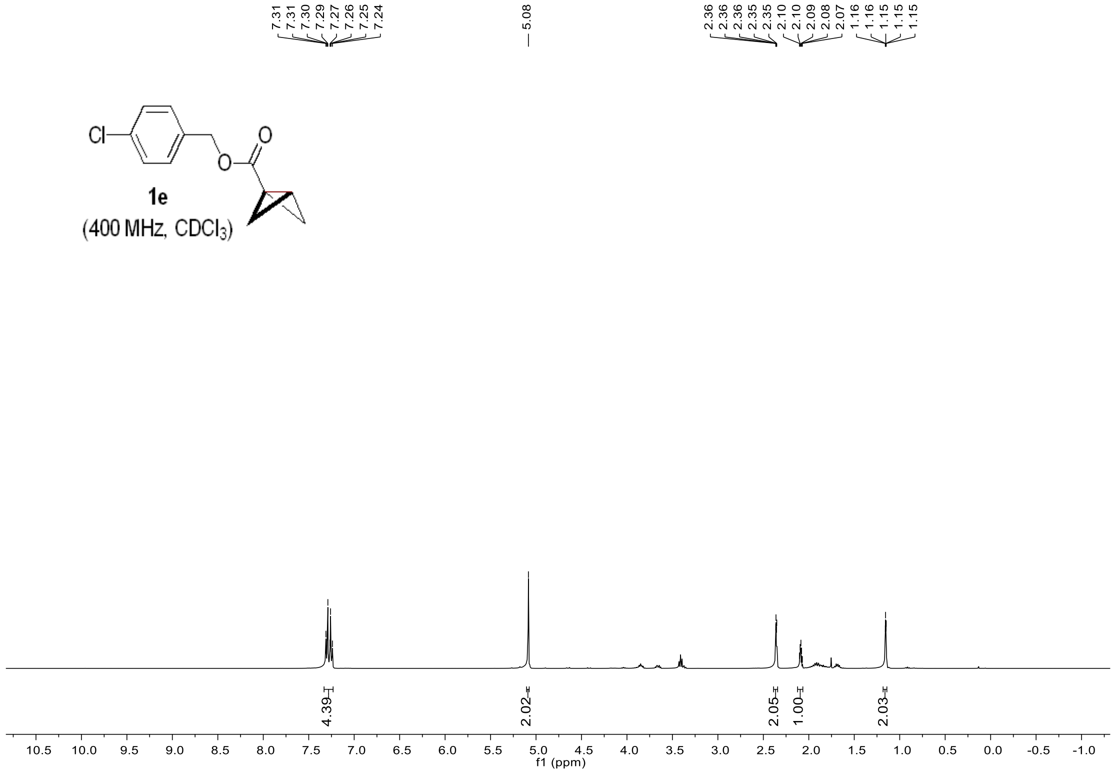


**1e**
(400 MHz, $CDCl_3$)

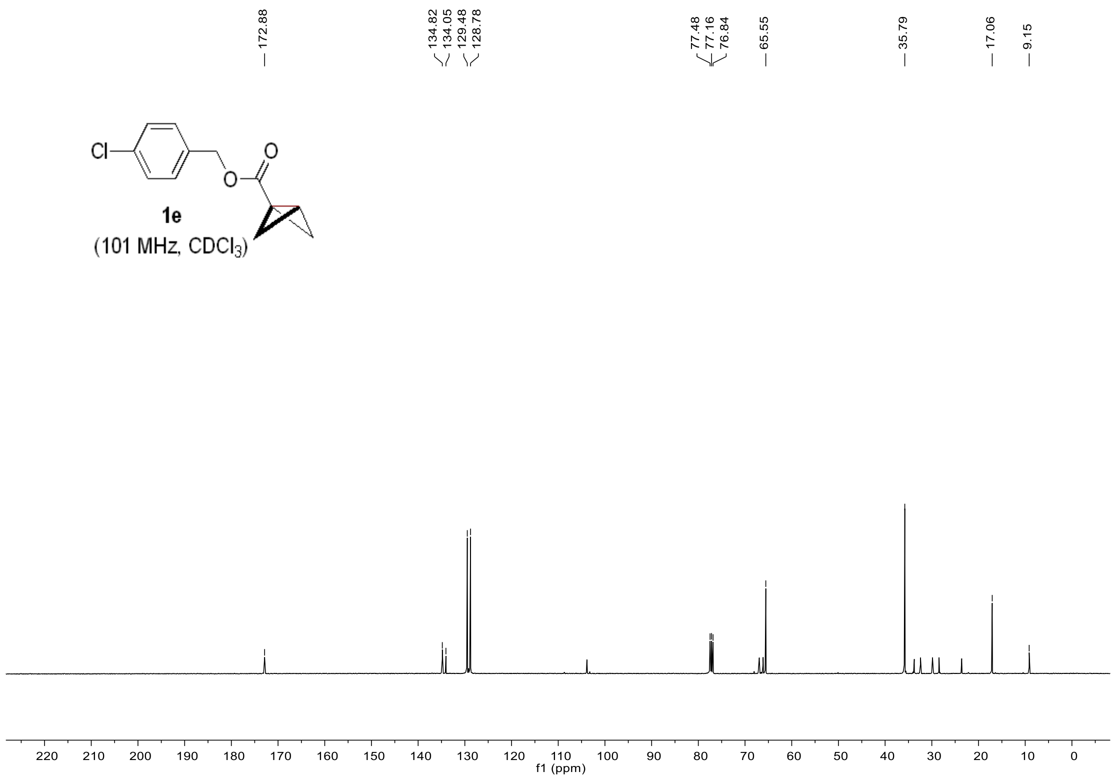
172.88
134.82
134.05
129.48
128.78
77.48
77.16
76.84
65.55
35.79
17.06
9.15
Cl
O
O
1e
(101 MHz, CDCl3)
f1 (ppm)

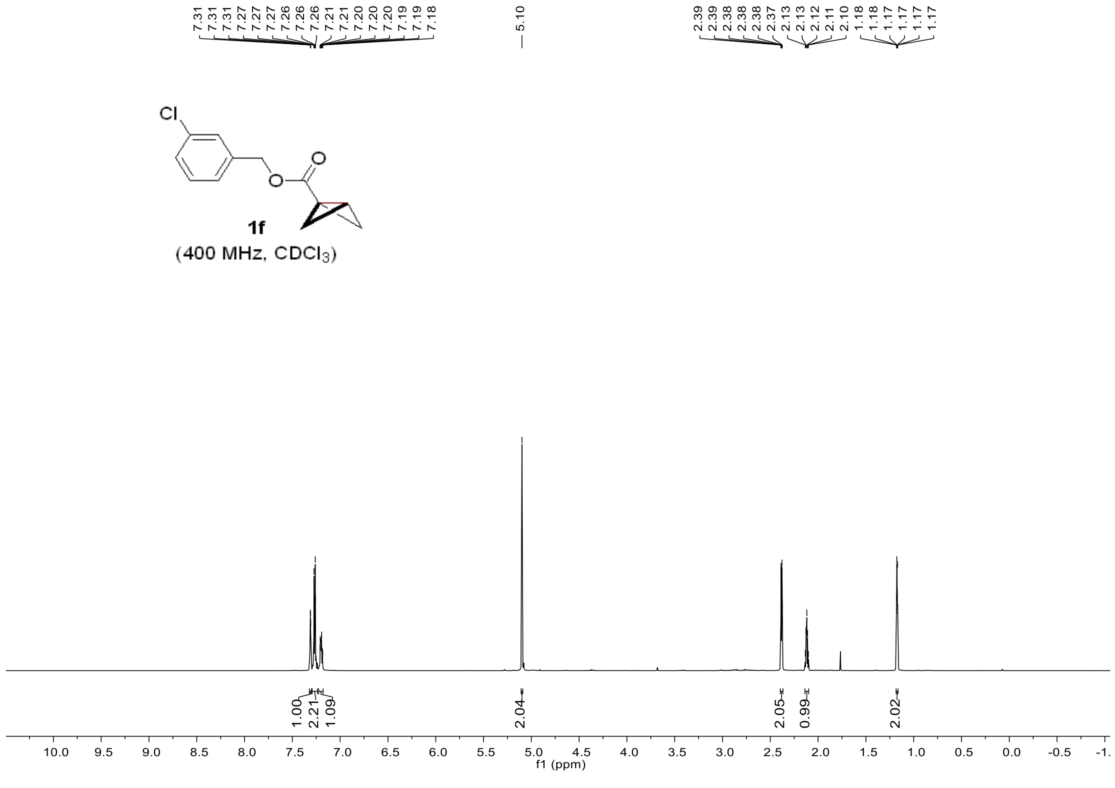
7.31 7.31 7.27 7.27 7.27 7.26 7.26 7.26 7.21 7.21 7.20 7.20 7.20 7.19 7.19 7.18
5.10
2.39 2.39 2.38 2.38 2.38 2.37 2.13 2.13 2.12 2.11 2.10 1.18 1.17 1.17 1.17 1.17
Cl
O
O
1f
(400 MHz, CDCl3)
1.00 2.21 1.09
2.04
2.05 0.99
2.02
f1 (ppm)

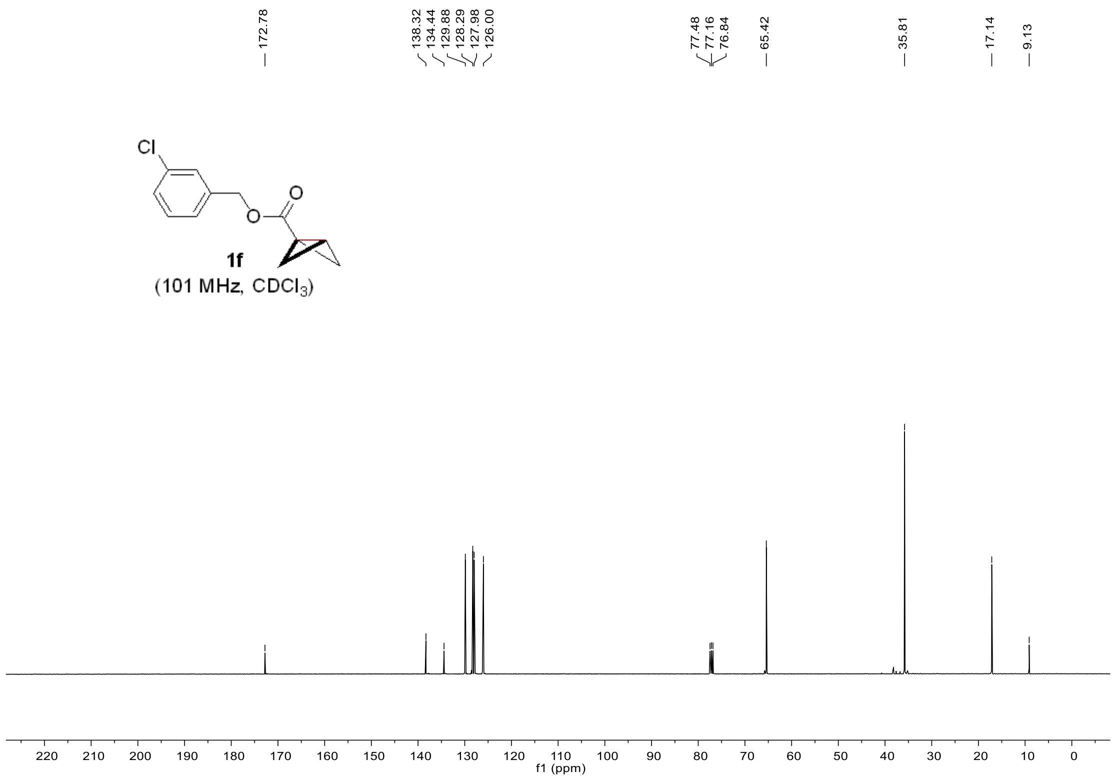
172.78
138.32
134.44
129.88
128.29
127.98
126.00
77.48
77.16
76.84
65.42
35.81
17.14
9.13
Cl
O
O
1f
(101 MHz, $CDCl_3$)
f1 (ppm)

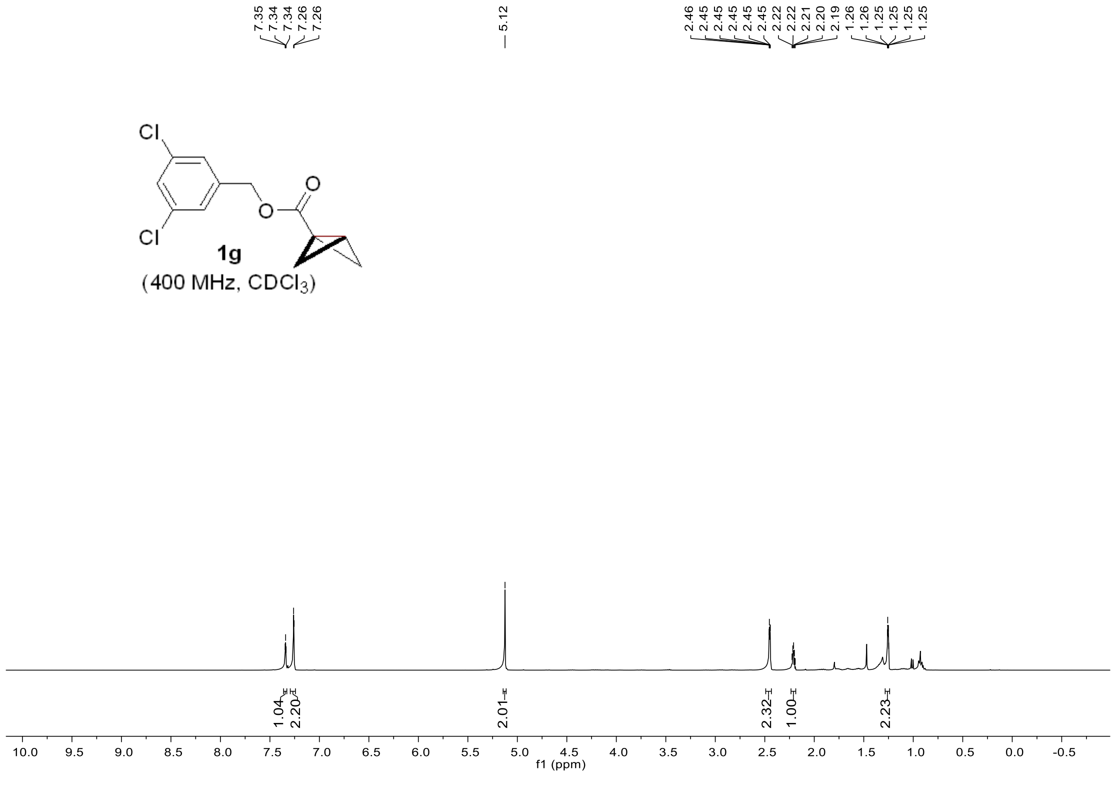
7.35
7.34
7.34
7.26
7.26
5.12
2.46
2.45
2.45
2.45
2.45
2.45
2.22
2.22
2.21
2.20
2.19
1.26
1.26
1.25
1.25
1.25
1.25
Cl
Cl
O
O
1g
(400 MHz, $CDCl_3$)
1.04
2.20
2.01
2.32
1.00
2.23
f1 (ppm)

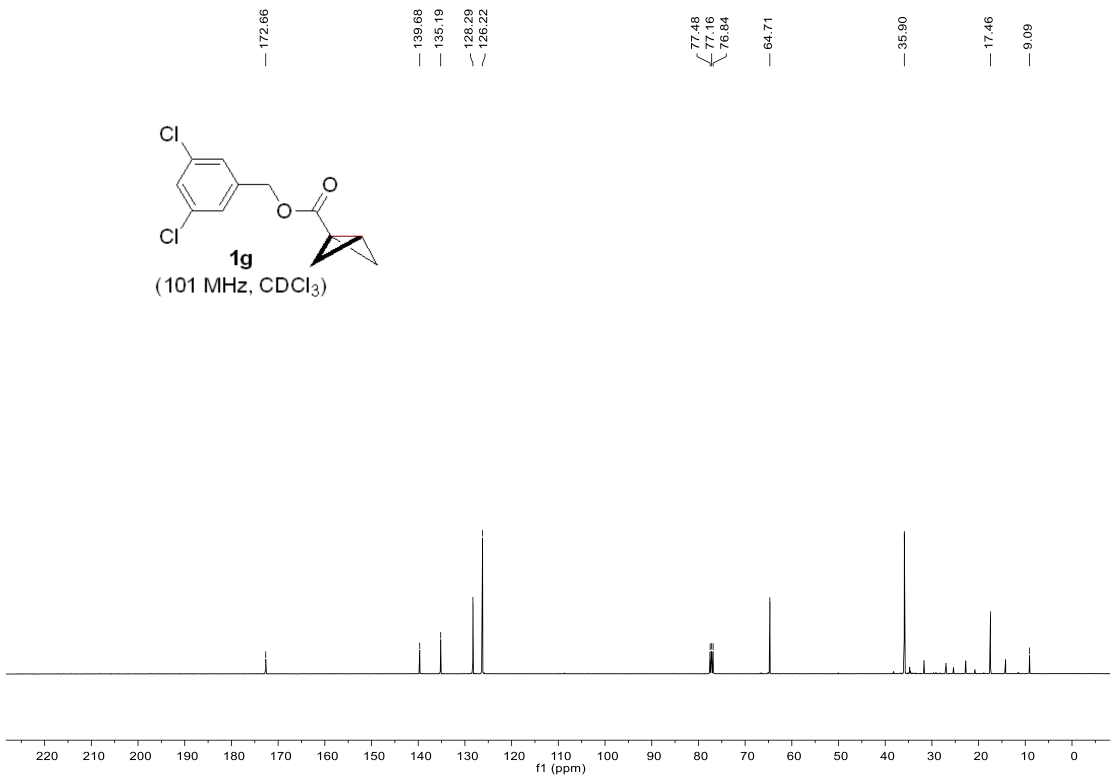
1g
(101 MHz, CDCl$_3$)
f1 (ppm)

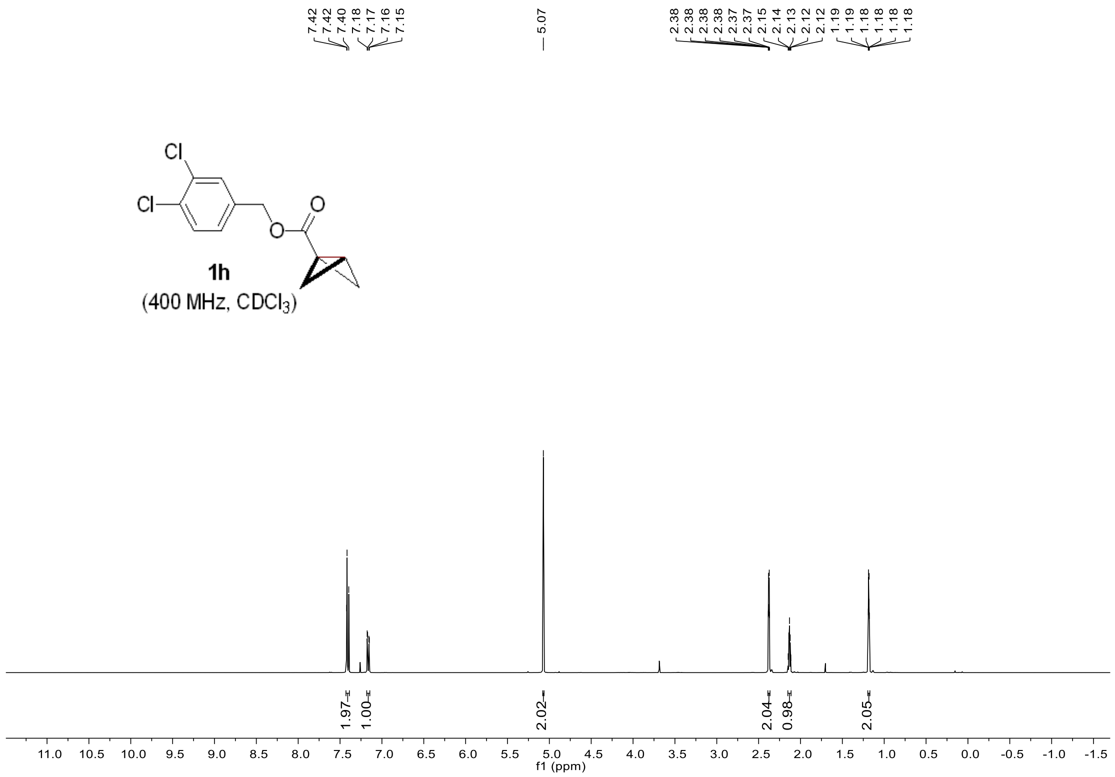
1h
(400 MHz, CDCl$_3$)
f1 (ppm)

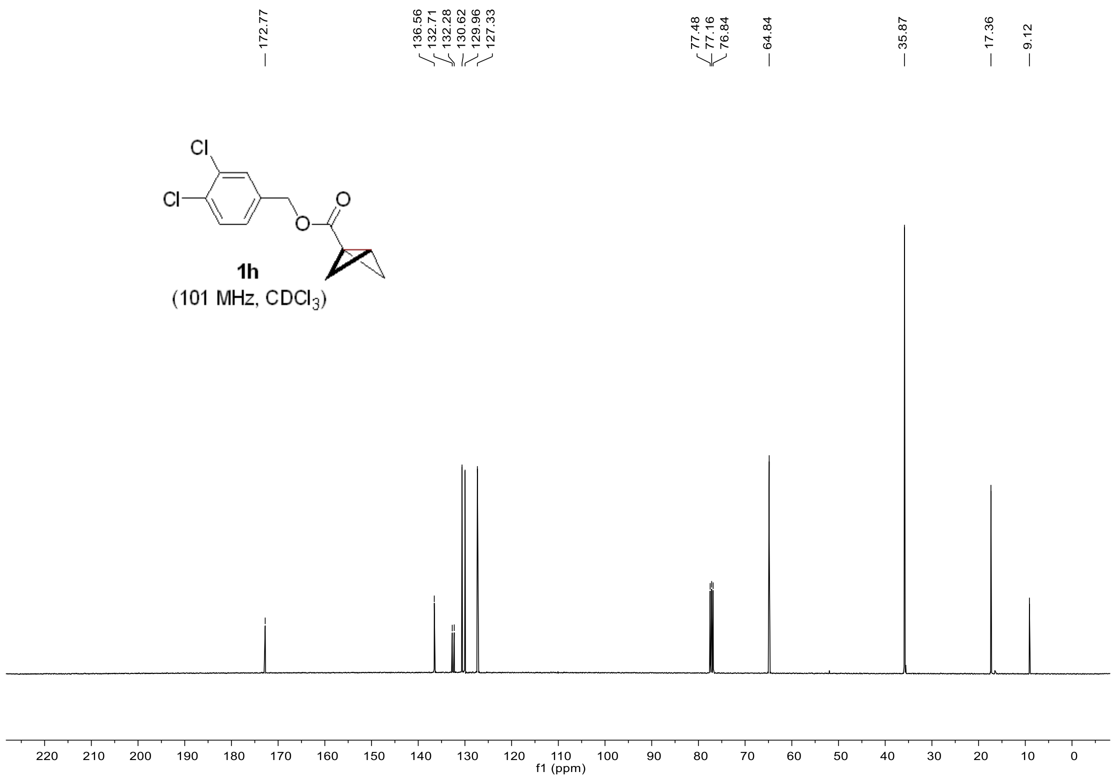
172.77
136.56
132.71
132.28
130.62
129.96
127.33
77.48
77.16
76.84
64.84
35.87
17.36
9.12
1h
(101 MHz, CDCl3)
f1 (ppm)

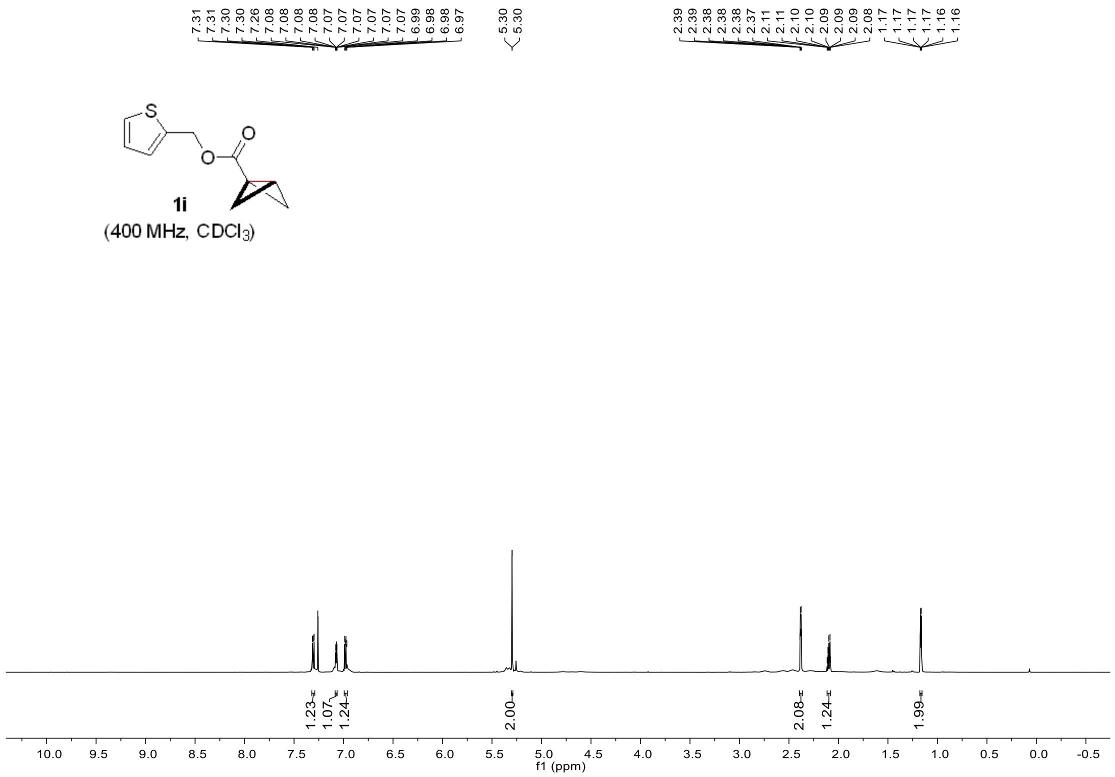
7.31
7.31
7.30
7.30
7.26
7.08
7.08
7.08
7.08
7.07
7.07
7.07
7.07
7.07
6.99
6.98
6.98
6.97
5.30
5.30
2.39
2.39
2.38
2.38
2.38
2.37
2.11
2.11
2.10
2.10
2.09
2.09
2.09
2.08
1.17
1.17
1.17
1.17
1.16
1.16
1i
(400 MHz, CDCl3)
1.23
1.07
1.24
2.00
2.08
1.24
1.99
f1 (ppm)

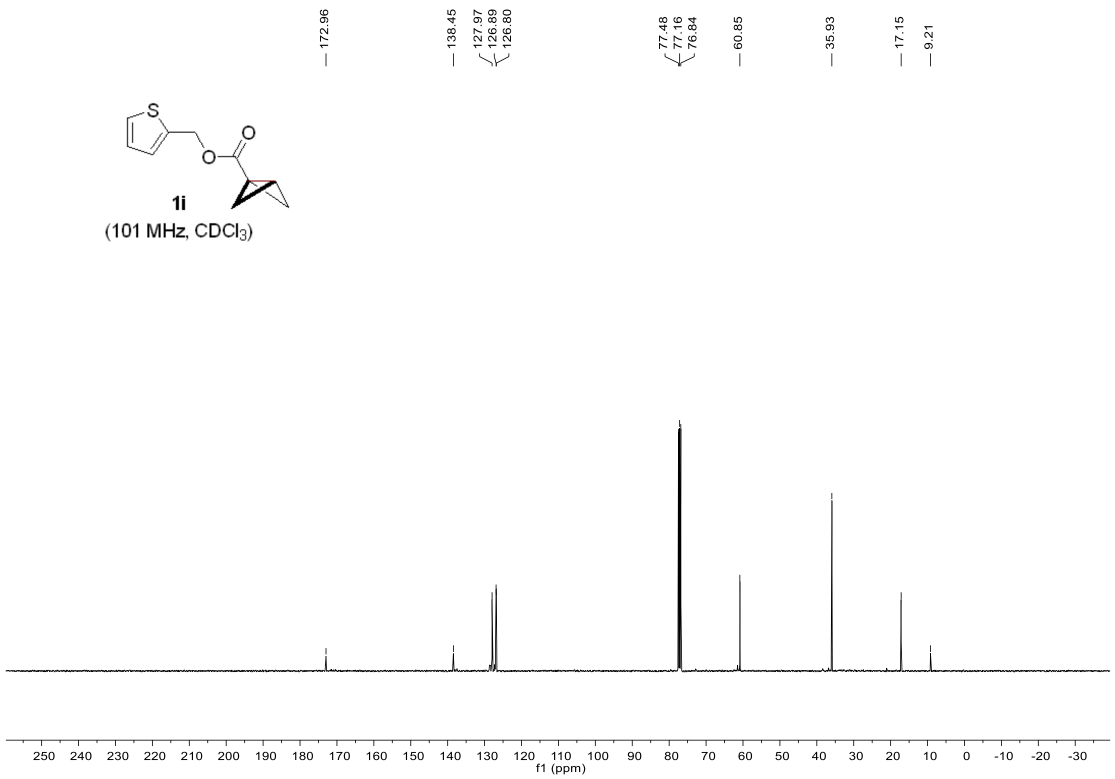
172.96
138.45
127.97
126.89
126.80
77.48
77.16
76.84
60.85
35.93
17.15
9.21
1i
(101 MHz, CDCl3)
250 240 230 220 210 200 190 180 170 160 150 140 130 120 110 100 90 80 70 60 50 40 30 20 10 0 -10 -20 -30
f1 (ppm)

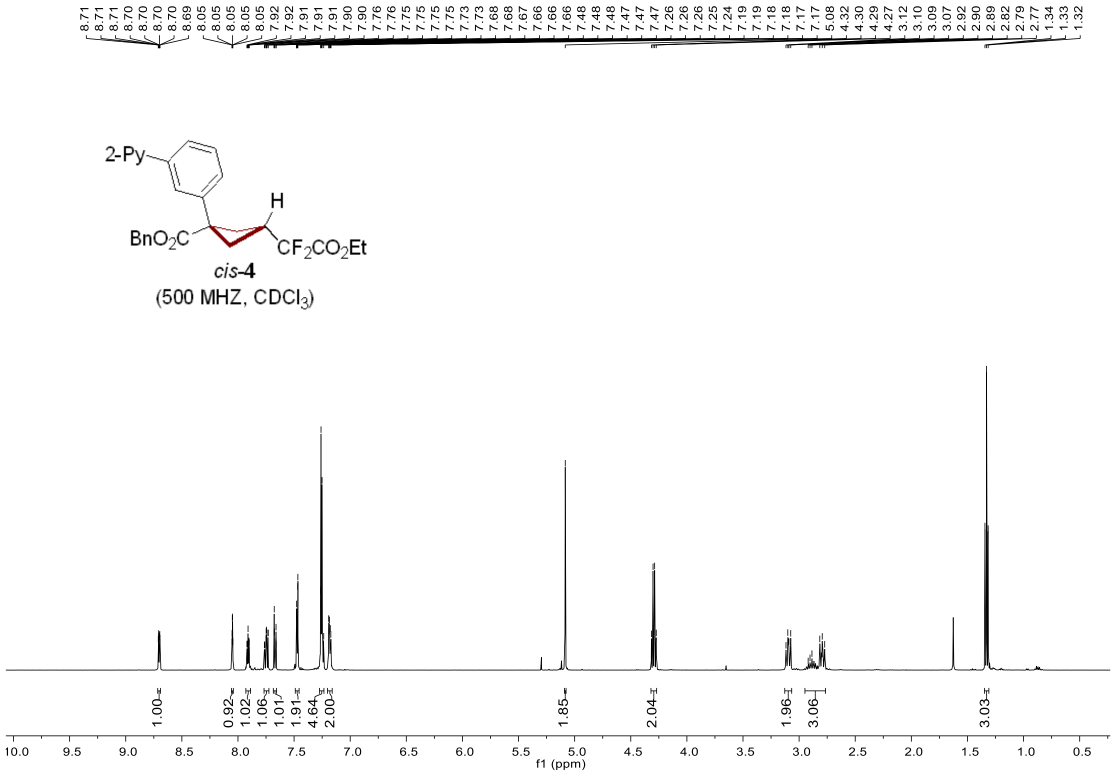
8.71 8.71 8.70 8.70 8.70 8.69 8.05 8.05 8.05 8.05 7.92 7.92 7.91 7.91 7.91 7.90 7.90 7.76 7.76 7.75 7.75 7.75 7.73 7.73 7.68 7.68 7.67 7.66 7.66 7.66 7.48 7.48 7.47 7.47 7.47 7.26 7.26 7.26 7.25 7.24 7.19 7.19 7.18 7.18 7.17 7.17 5.08 4.32 4.30 4.29 4.27 3.12 3.10 3.09 3.07 2.92 2.90 2.89 2.82 2.79 2.77 1.34 1.33 1.32
2-Py
H
BnO2C
CF2CO2Et
cis-4
(500 MHZ, CDCl3)
1.00
0.92
1.02
1.06
1.01
1.91
4.64
2.00
1.85
2.04
1.96
3.06
3.03
10.0 9.5 9.0 8.5 8.0 7.5 7.0 6.5 6.0 5.5 5.0 4.5 4.0 3.5 3.0 2.5 2.0 1.5 1.0 0.5
f1 (ppm)

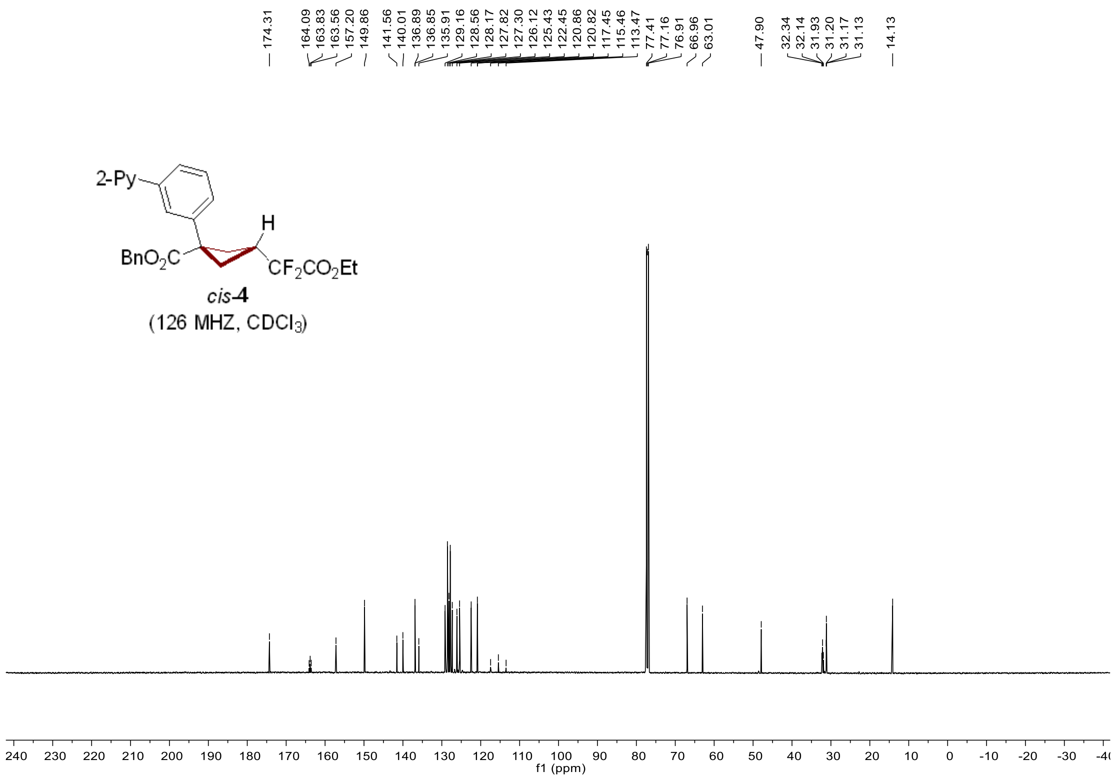

174.31
164.09
163.83
163.56
157.20
149.86
141.56
140.01
136.89
136.85
135.91
129.16
128.56
128.17
127.82
127.30
126.12
125.43
122.45
120.86
120.82
117.45
115.46
113.47
77.41
77.16
76.91
66.96
63.01
47.90
32.34
32.14
31.93
31.20
31.17
31.13
14.13
2-Py
H
$BnO_2C$
$CF_2CO_2Et$
*cis*-**4**
(126 MHZ, $CDCl_3$)
f1 (ppm)


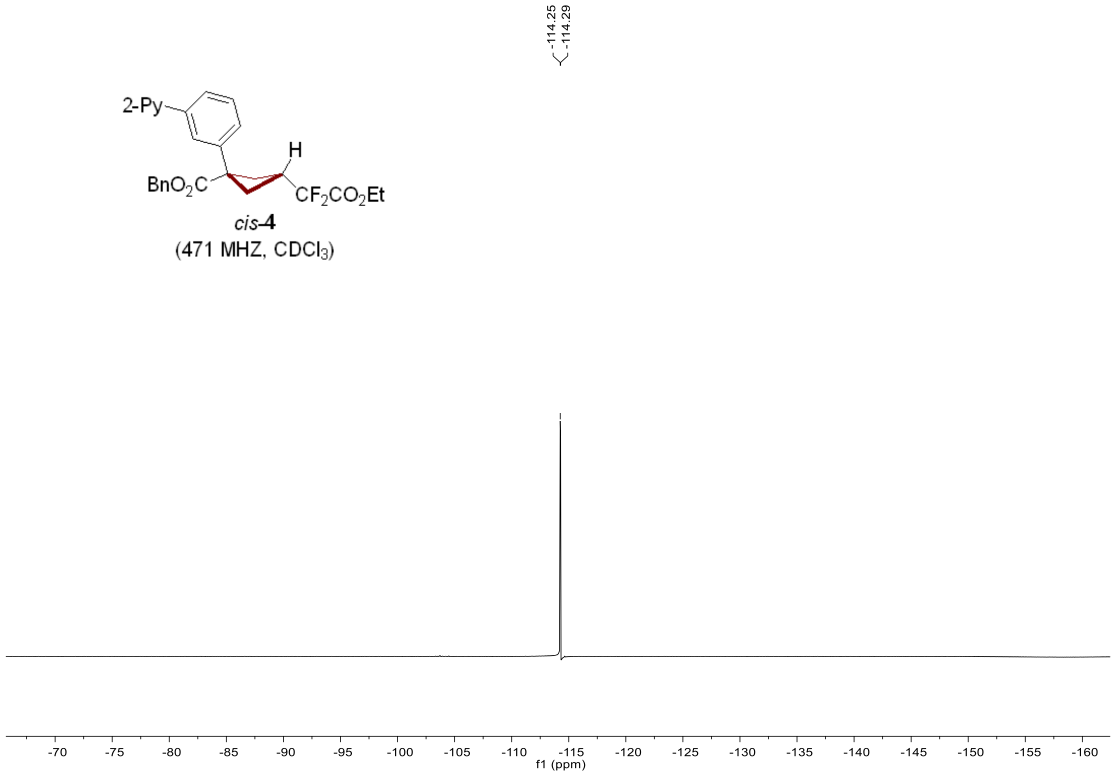

-114.25
-114.29
2-Py
H
$BnO_2C$
$CF_2CO_2Et$
*cis*-**4**
(471 MHZ, $CDCl_3$)
f1 (ppm)

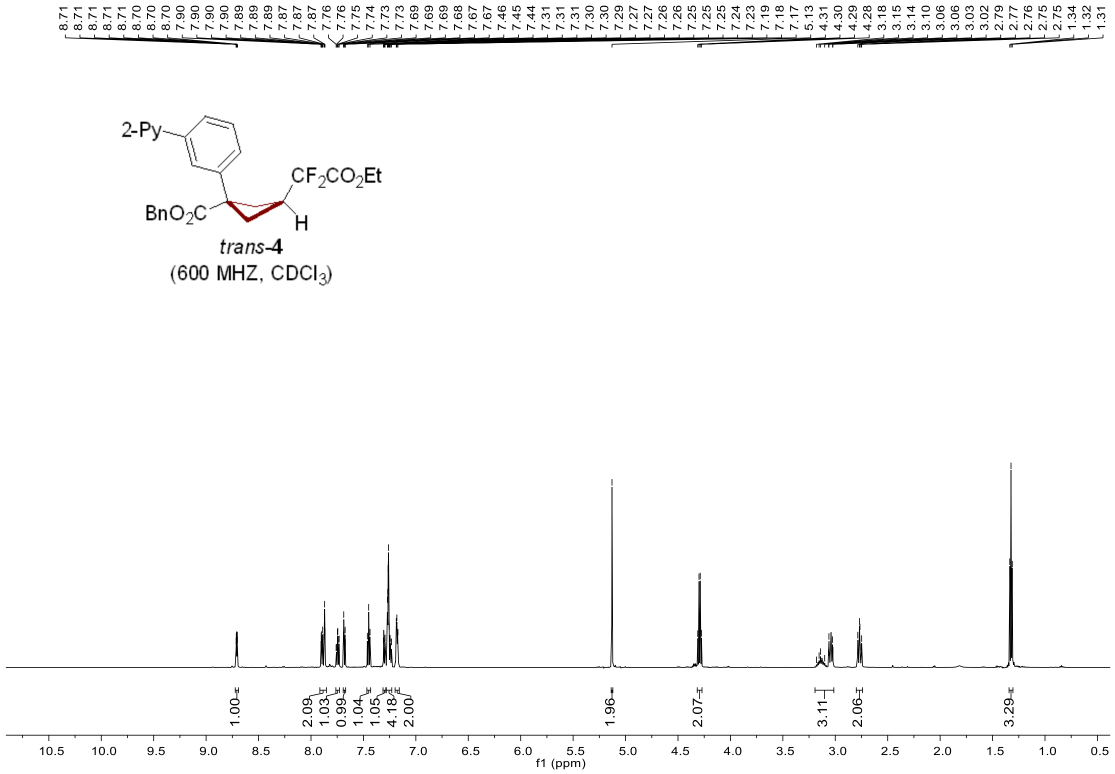
2-Py
CF2CO2Et
BnO2C
H
trans-4
(600 MHZ, CDCl3)
f1 (ppm)

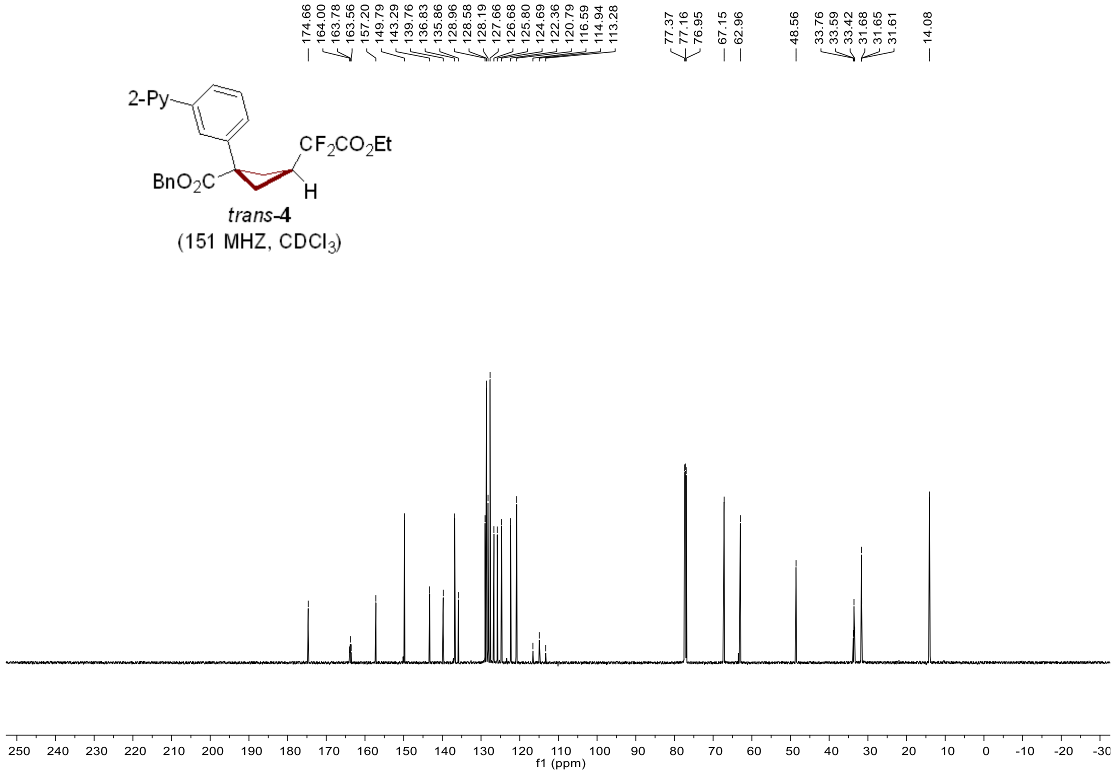
2-Py
CF2CO2Et
BnO2C
H
trans-4
(151 MHZ, CDCl3)
f1 (ppm)

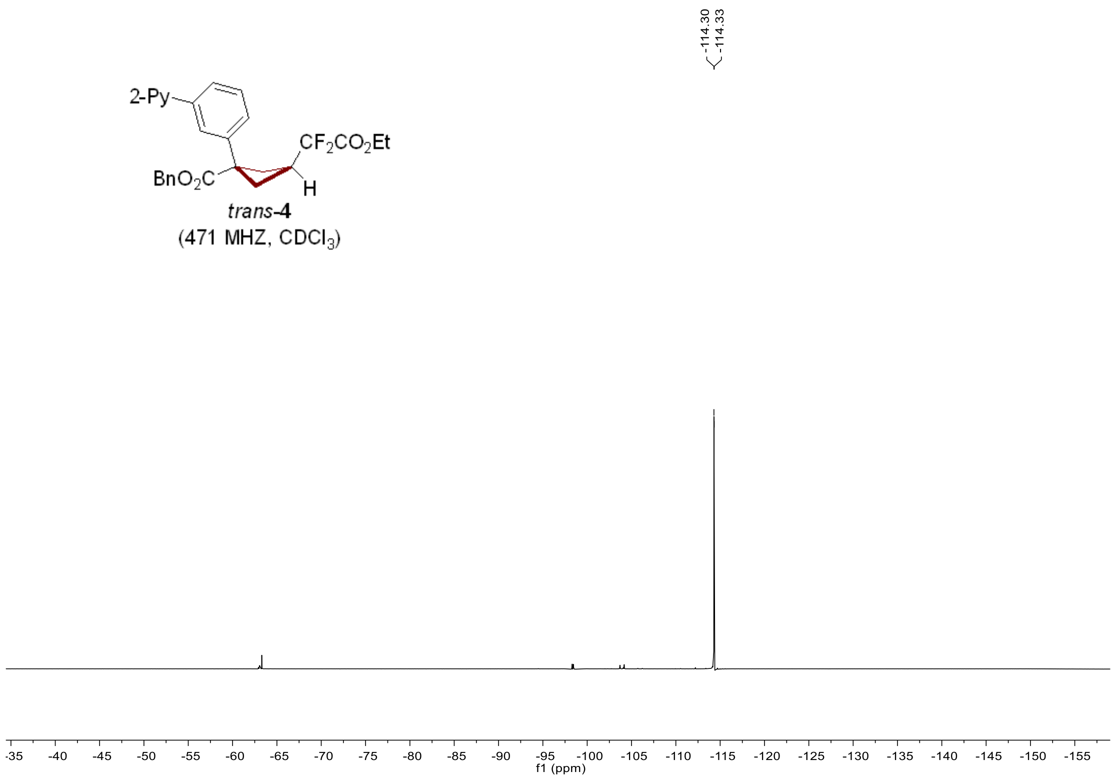
-114.30
-114.33
2-Py
$CF_2CO_2Et$
$BnO_2C$
H
*trans*-**4**
(471 MHZ, $CDCl_3$)
f1 (ppm)

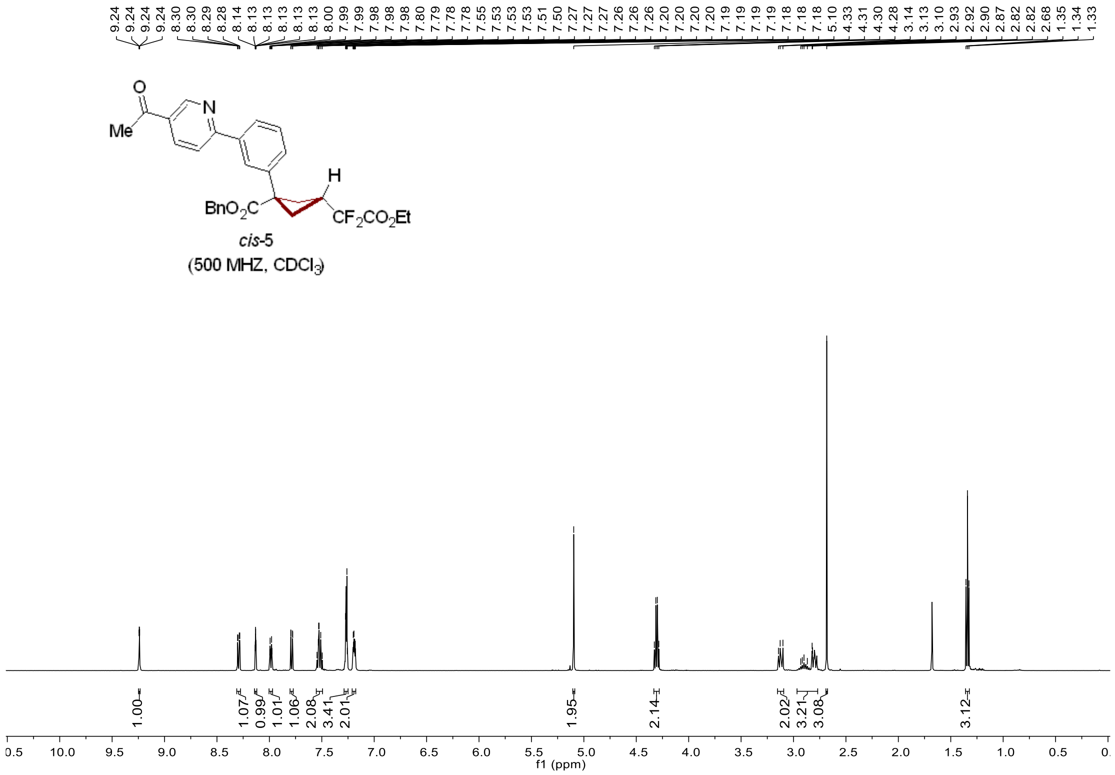
9.24 9.24 9.24 9.24 8.30 8.29 8.28 8.14 8.13 8.13 8.13 8.13 8.13 8.00 7.99 7.98 7.98 7.98 7.98 7.80 7.79 7.78 7.78 7.55 7.53 7.53 7.53 7.51 7.50 7.27 7.27 7.27 7.26 7.26 7.20 7.20 7.20 7.20 7.19 7.19 7.19 7.19 7.18 7.18 7.18 5.10 4.33 4.31 4.30 4.28 3.14 3.13 3.10 2.93 2.92 2.90 2.87 2.82 2.82 2.68 1.35 1.34 1.33
O
Me
N
H
$BnO_2C$
$CF_2CO_2Et$
*cis*-5
(500 MHZ, $CDCl_3$)
1.00 1.07 0.99 1.01 1.06 2.08 3.41 2.01 1.95 2.14 2.02 3.21 3.08 3.12
f1 (ppm)

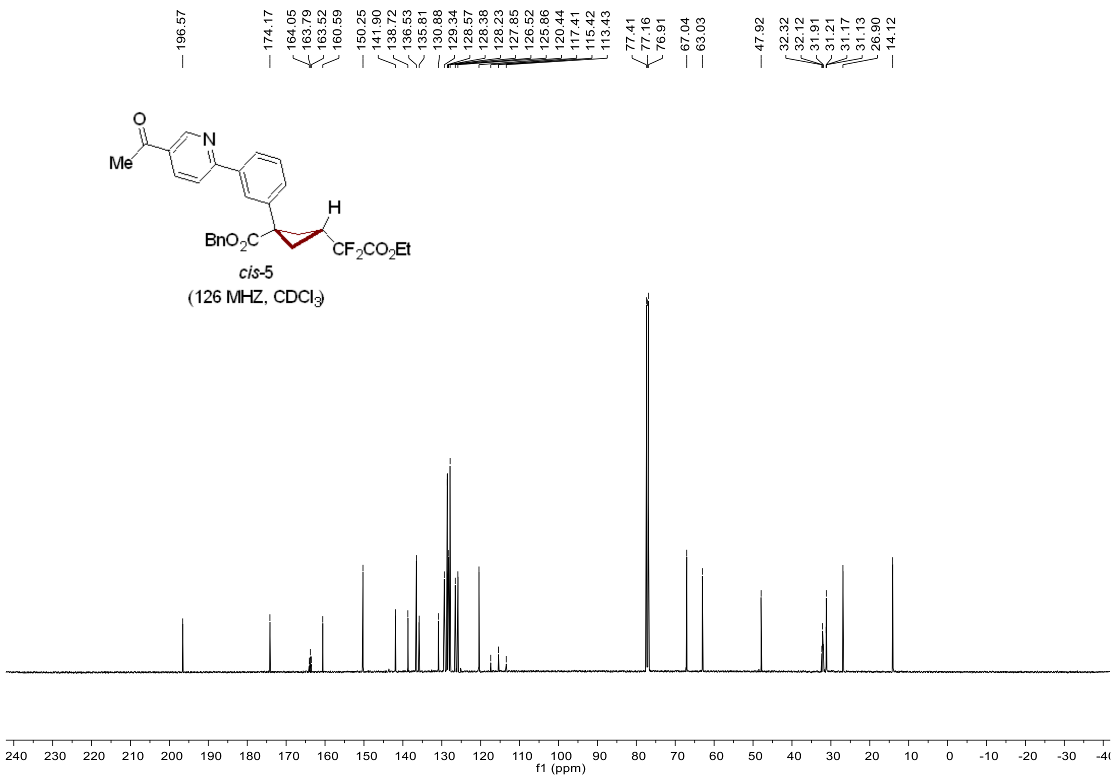
196.57
174.17
164.05
163.79
163.52
160.59
150.25
141.90
138.72
136.53
135.81
130.88
129.34
128.57
128.38
128.23
127.85
126.52
125.86
120.44
117.41
115.42
113.43
77.41
77.16
76.91
67.04
63.03
47.92
32.32
32.12
31.91
31.21
31.17
31.13
26.90
14.12
O
N
Me
H
BnO2C
CF2CO2Et
cis-5
(126 MHZ, CDCl3)
f1 (ppm)

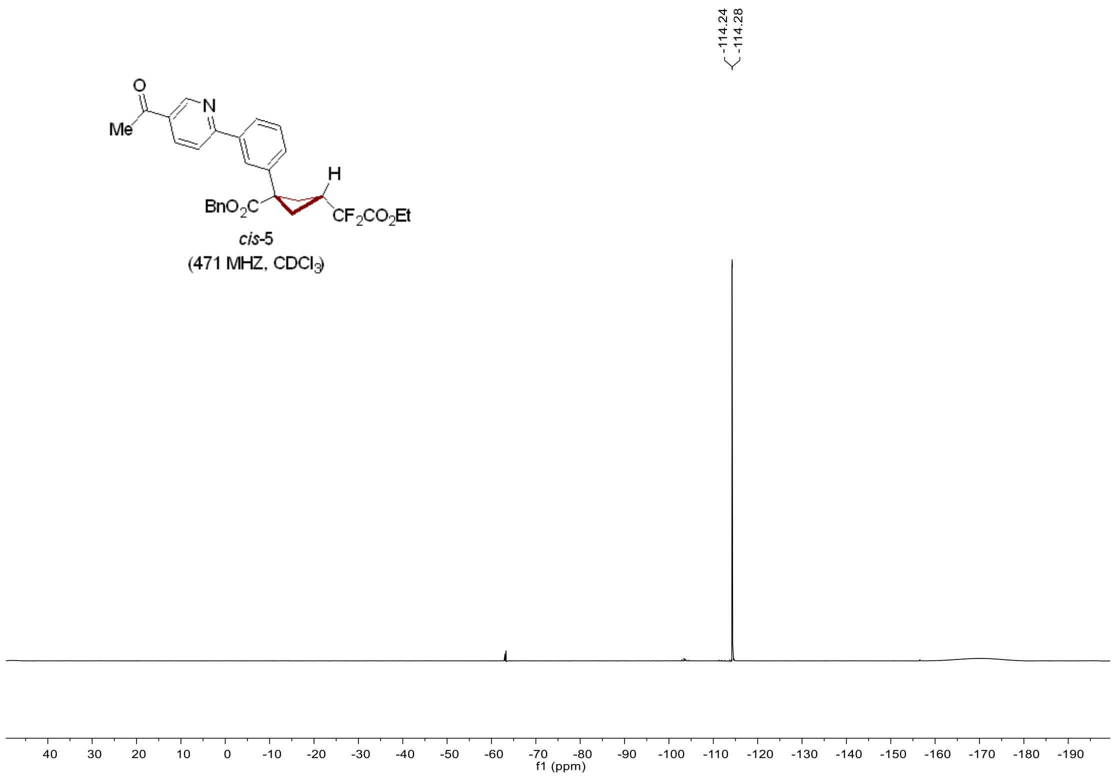
-114.24
-114.28
O
N
Me
H
BnO2C
CF2CO2Et
cis-5
(471 MHZ, CDCl3)
f1 (ppm)

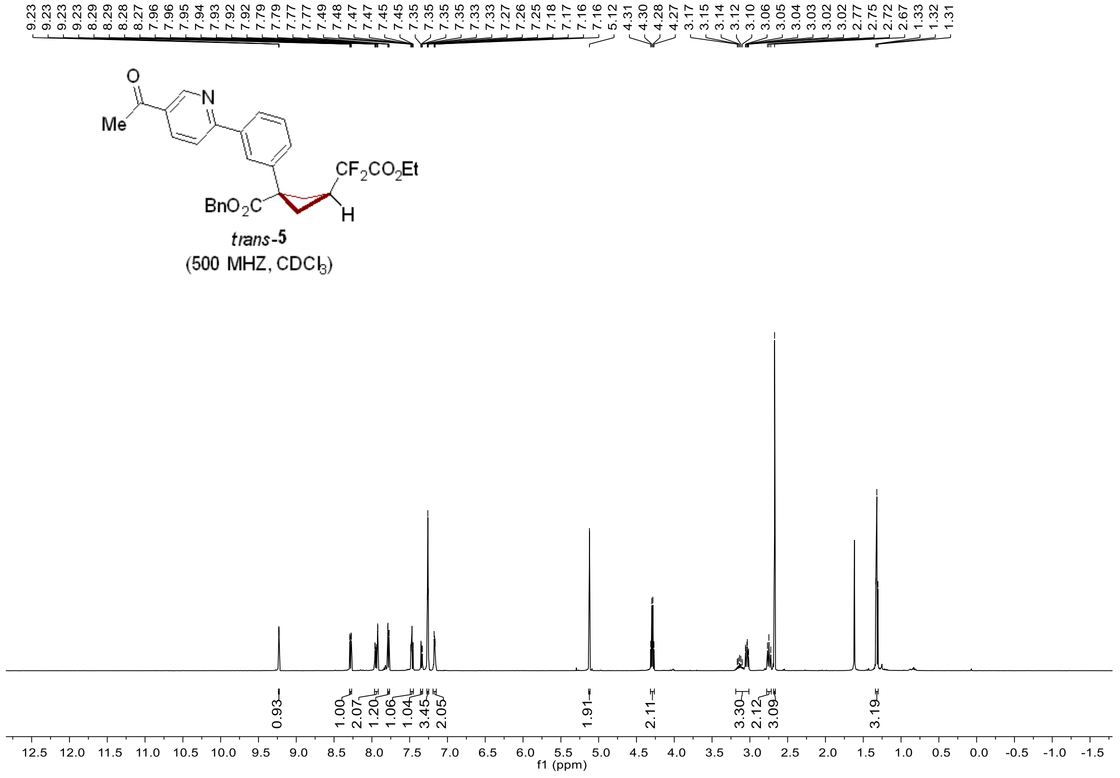
trans-5
(500 MHZ, CDCl3)
f1 (ppm)

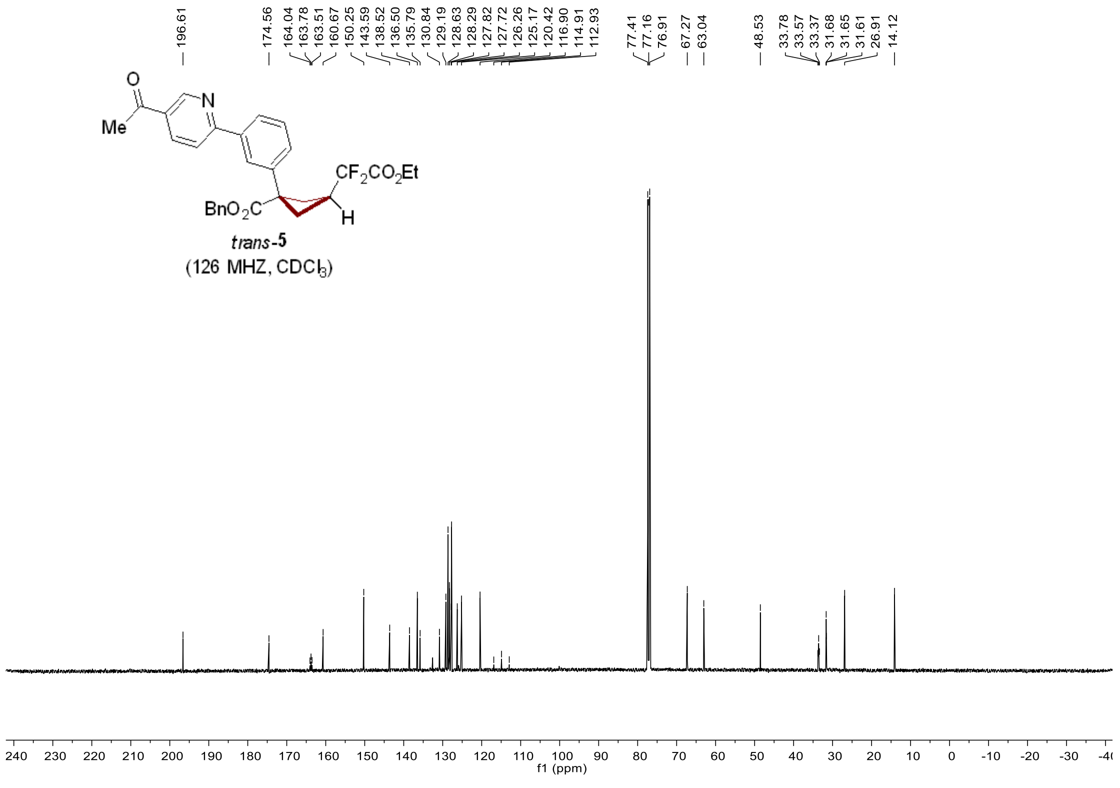
trans-5
(126 MHZ, CDCl3)
f1 (ppm)

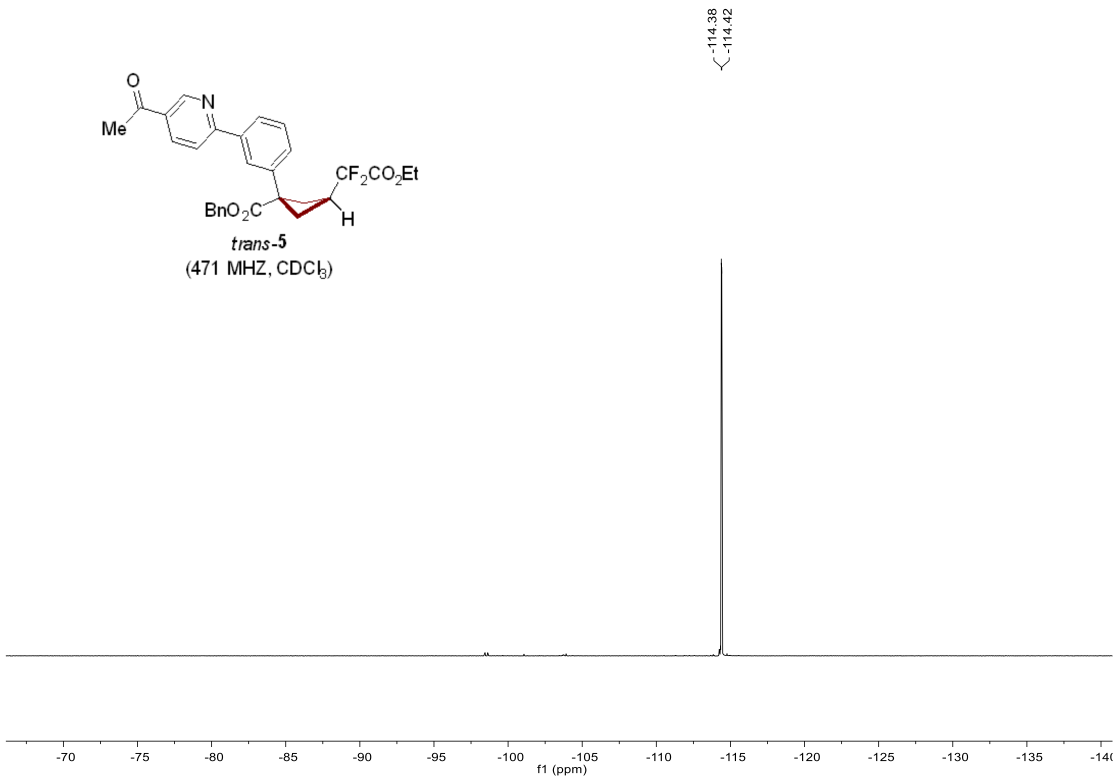

-114.38
-114.42
Me
O
N
$CF_2CO_2Et$
$BnO_2C$
H
*trans*-**5**
(471 MHZ, $CDCl_3$)
-70 -75 -80 -85 -90 -95 -100 -105 -110 -115 -120 -125 -130 -135 -140
f1 (ppm)


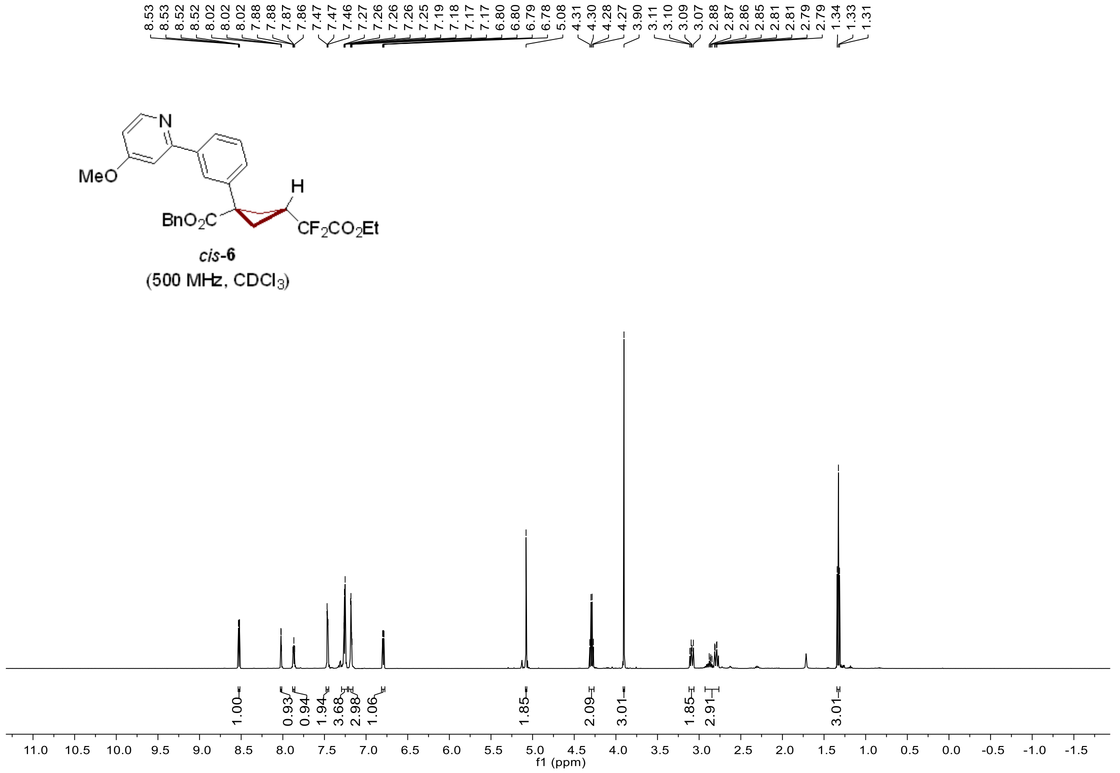

8.53 8.53 8.52 8.52 8.02 8.02 8.02 7.88 7.88 7.87 7.86 7.47 7.47 7.46 7.27 7.26 7.26 7.26 7.25 7.19 7.18 7.17 7.17 6.80 6.80 6.79 6.78 5.08 4.31 4.30 4.28 4.27 3.90 3.11 3.10 3.09 3.07 2.88 2.87 2.86 2.85 2.81 2.81 2.79 1.34 1.33 1.31
N
MeO
H
$BnO_2C$
$CF_2CO_2Et$
*cis*-**6**
(500 MHz, $CDCl_3$)
1.00 0.93 0.94 1.94 3.68 2.98 1.06 1.85 2.09 3.01 1.85 2.91 3.01
11.0 10.5 10.0 9.5 9.0 8.5 8.0 7.5 7.0 6.5 6.0 5.5 5.0 4.5 4.0 3.5 3.0 2.5 2.0 1.5 1.0 0.5 0.0 -0.5 -1.0 -1.5
f1 (ppm)

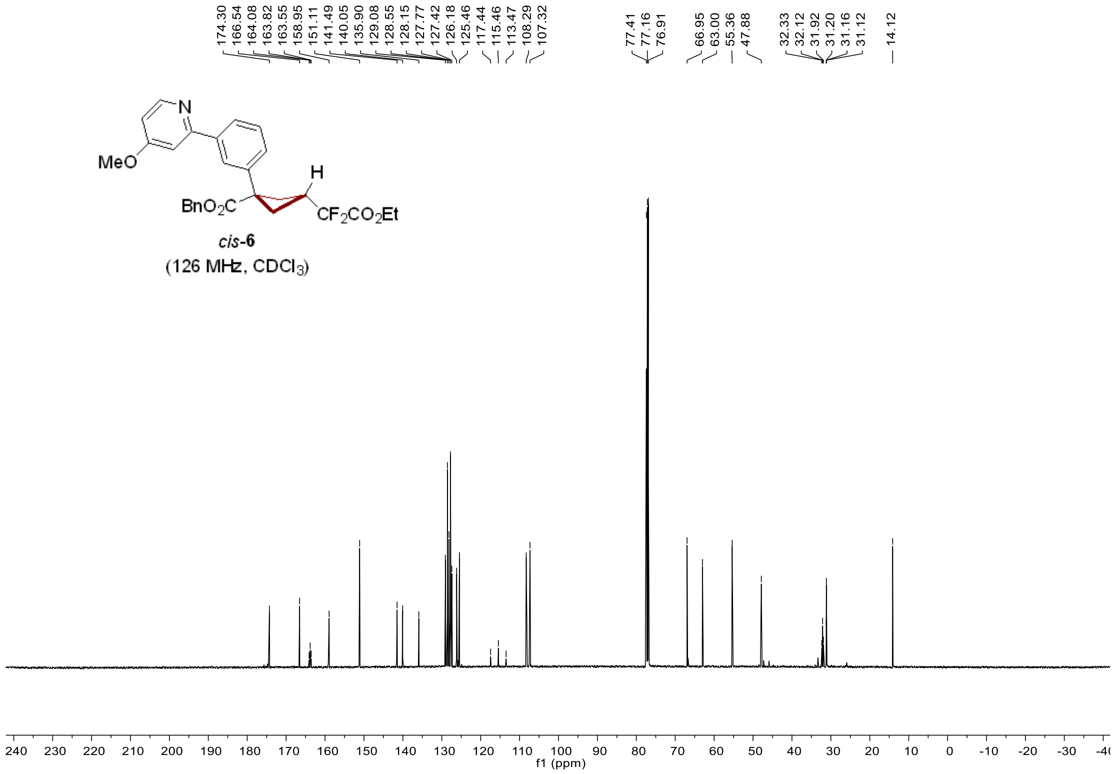
174.30
166.54
164.08
163.82
163.55
158.95
151.11
141.49
140.05
135.90
129.08
128.55
128.15
127.77
127.42
126.18
125.46
117.44
115.46
113.47
108.29
107.32
77.41
77.16
76.91
66.95
63.00
55.36
47.88
32.33
32.12
31.92
31.20
31.16
31.12
14.12
MeO
N
H
BnO2C
CF2CO2Et
cis-6
(126 MHz, CDCl3)
f1 (ppm)

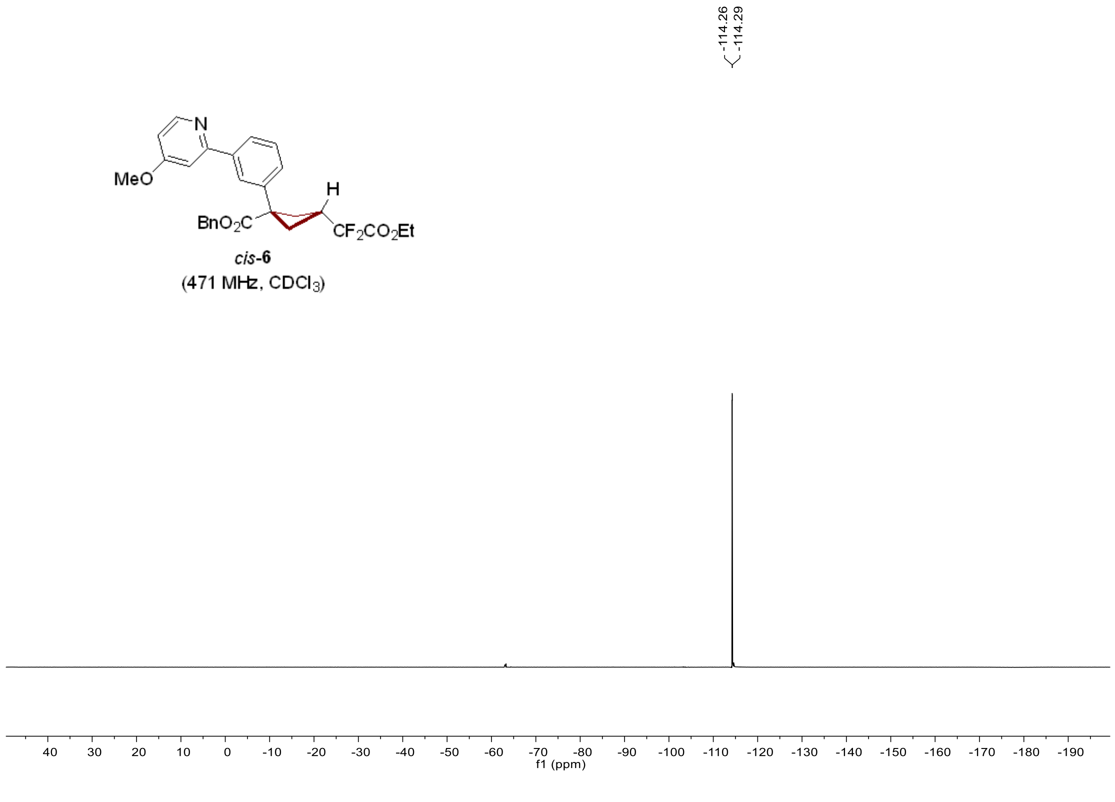
-114.26
-114.29
MeO
N
H
BnO2C
CF2CO2Et
cis-6
(471 MHz, CDCl3)
f1 (ppm)

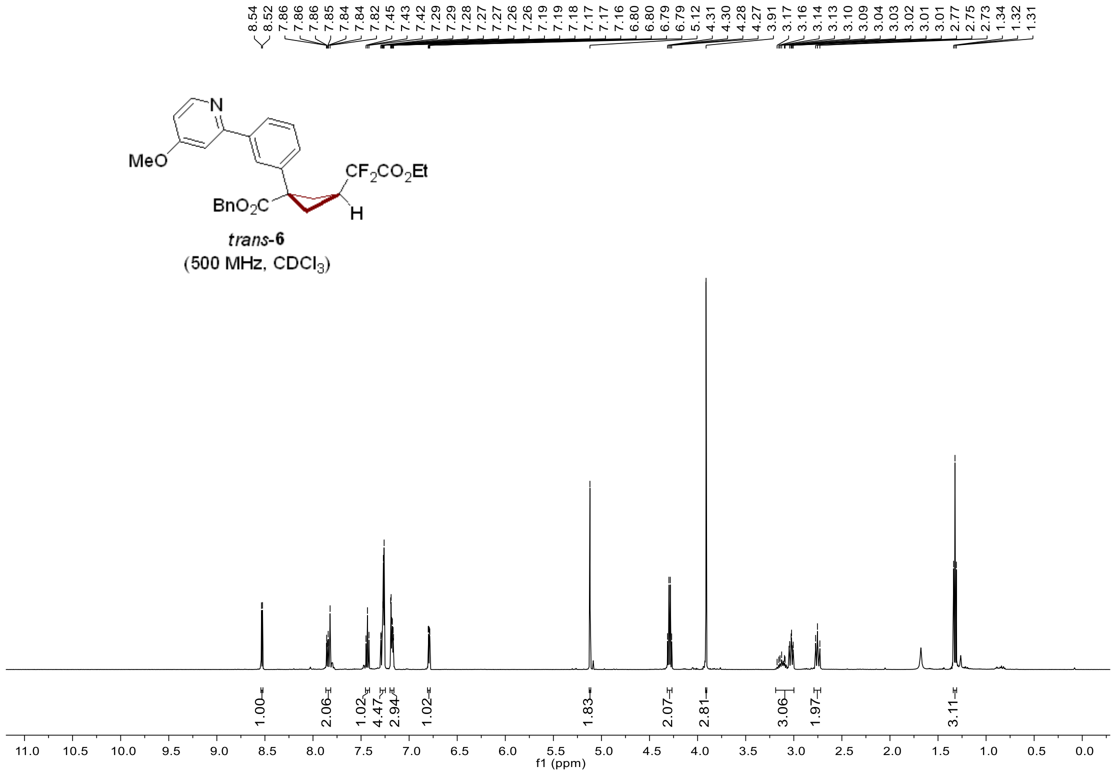
MeO
N
CF2CO2Et
BnO2C
H
trans-6
(500 MHz, CDCl3)
f1 (ppm)

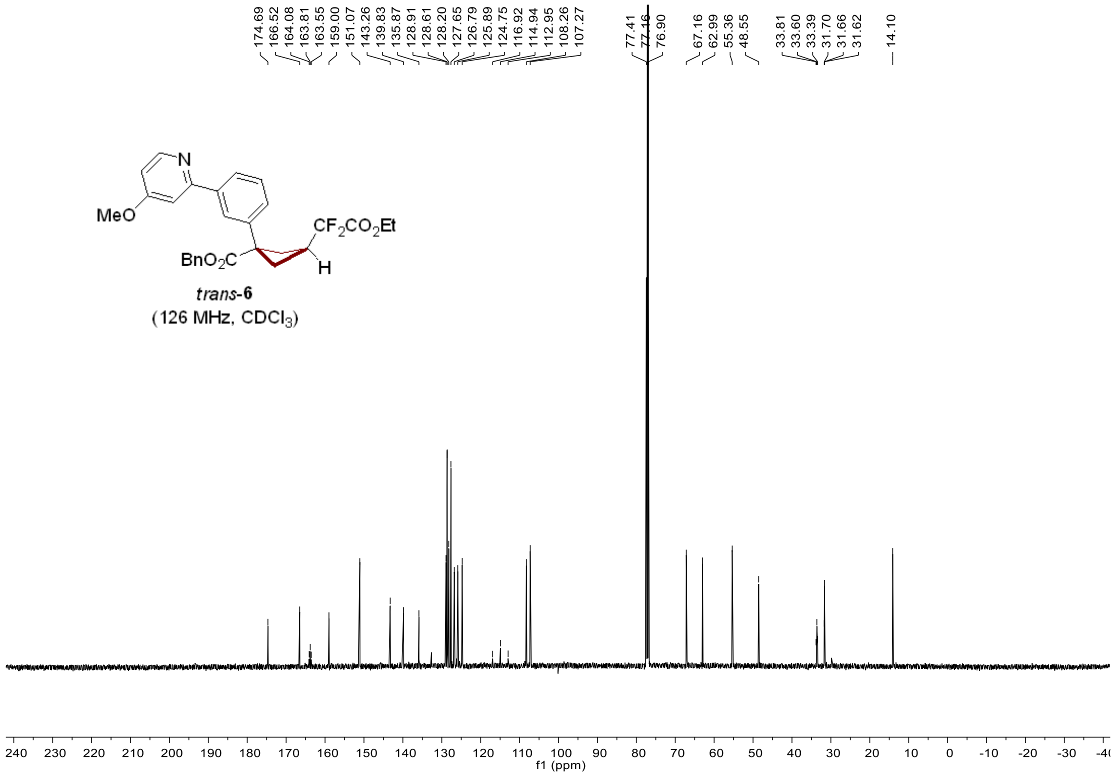
MeO
N
CF2CO2Et
BnO2C
H
trans-6
(126 MHz, CDCl3)
f1 (ppm)

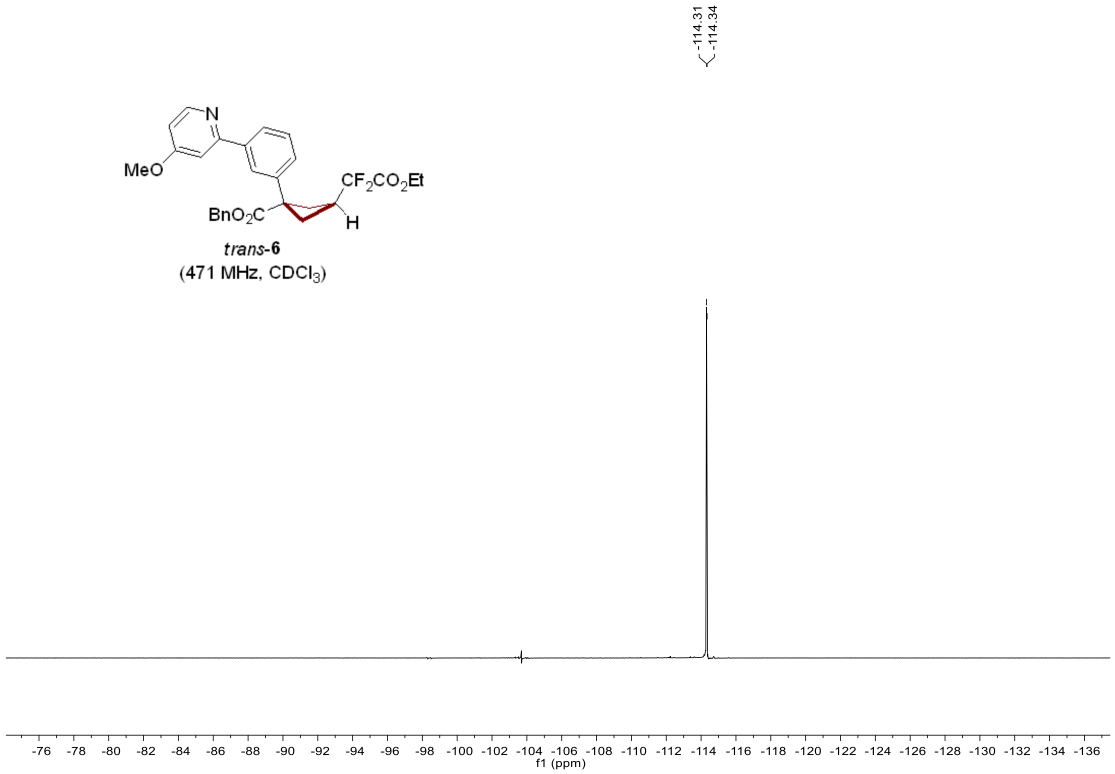

*trans*-**6**
(471 MHz, $CDCl_3$)

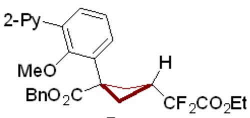

**7**
(500 MHz, $CDCl_3$)

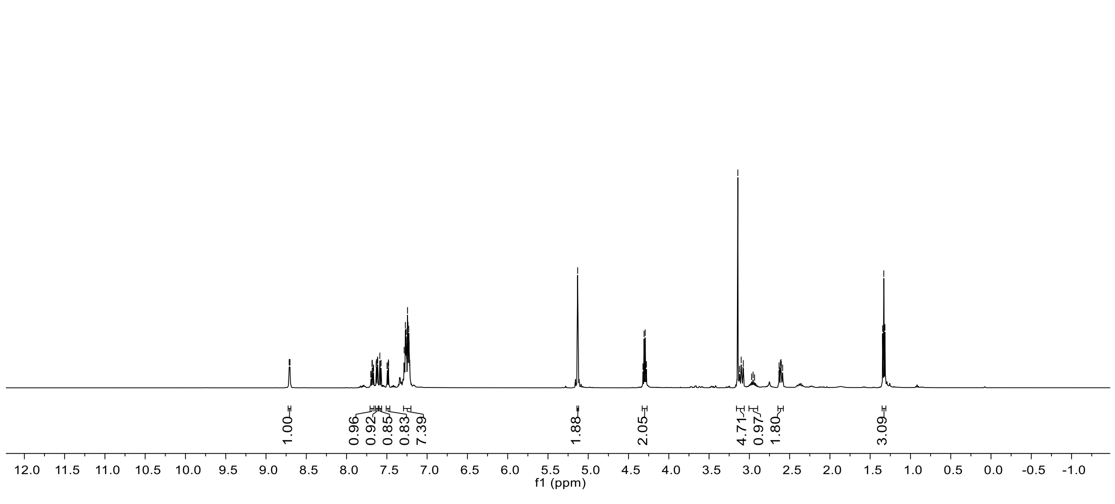

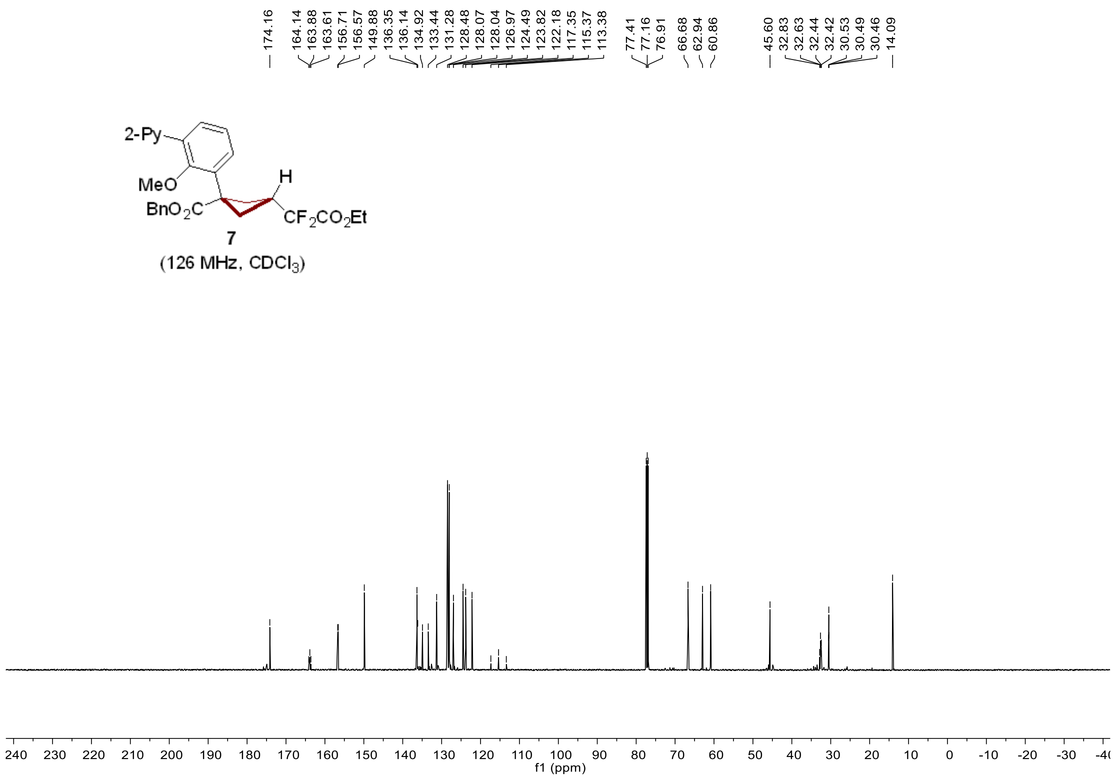
174.16
164.14
163.88
163.61
156.71
156.57
149.88
136.35
136.14
134.92
133.44
131.28
128.48
128.07
128.04
126.97
124.49
123.82
122.18
117.35
115.37
113.38
77.41
77.16
76.91
66.68
62.94
60.86
45.60
32.83
32.63
32.44
32.42
30.53
30.49
30.46
14.09
2-Py
MeO
$BnO_2C$
H
$CF_2CO_2Et$
7
(126 MHz, $CDCl_3$)
240 230 220 210 200 190 180 170 160 150 140 130 120 110 100 90 80 70 60 50 40 30 20 10 0 -10 -20 -30
f1 (ppm)

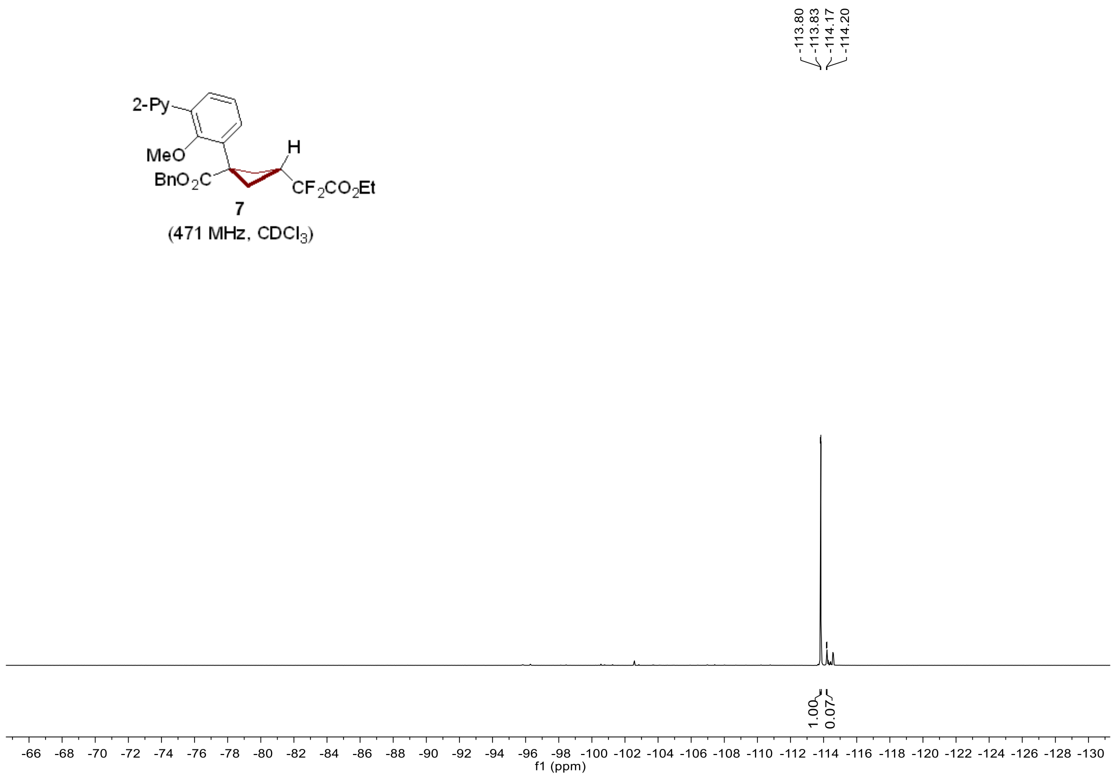
-113.80
-113.83
-114.17
-114.20
2-Py
MeO
$BnO_2C$
H
$CF_2CO_2Et$
7
(471 MHz, $CDCl_3$)
1.00
0.07
-66 -68 -70 -72 -74 -76 -78 -80 -82 -84 -86 -88 -90 -92 -94 -96 -98 -100 -102 -104 -106 -108 -110 -112 -114 -116 -118 -120 -122 -124 -126 -128 -130
f1 (ppm)

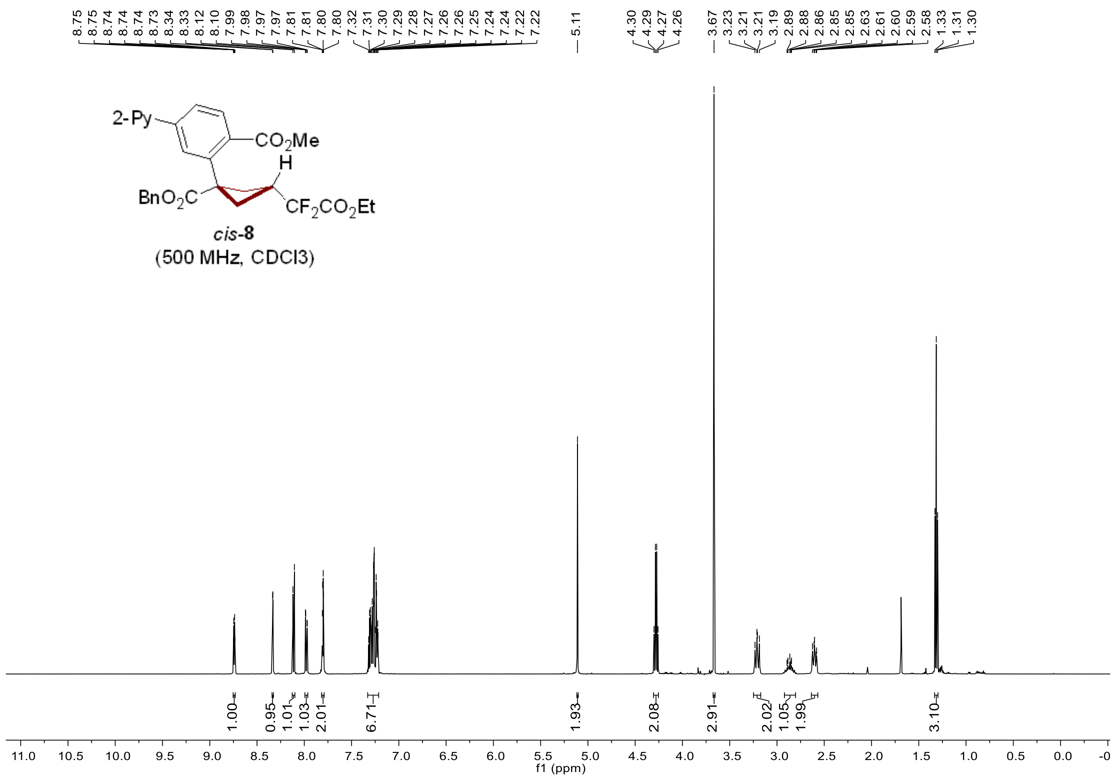

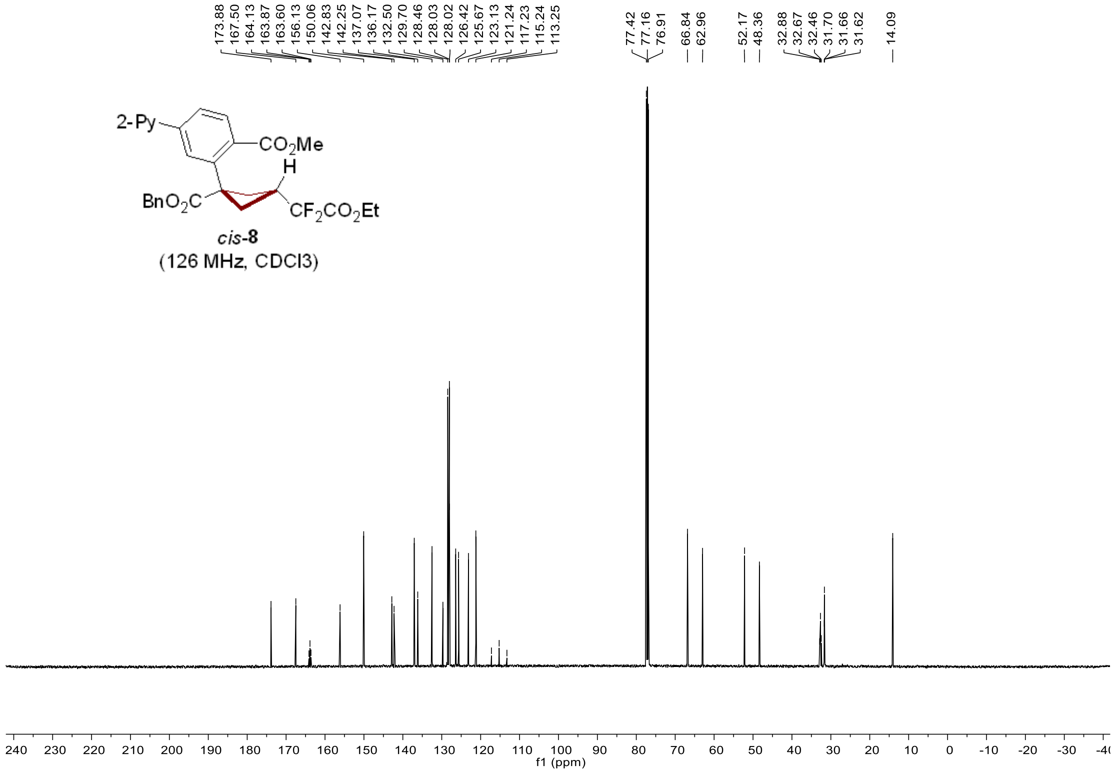

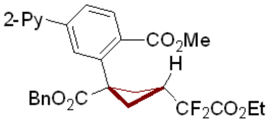


*cis*-**8**
(500 MHz, CDCl3)

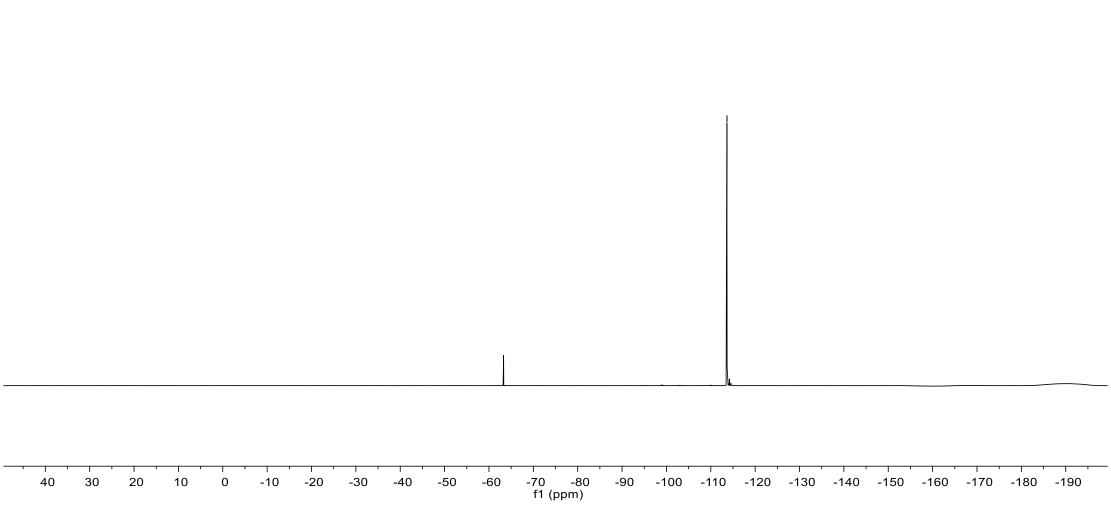


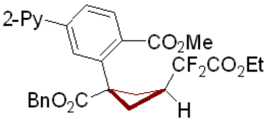


*trans*-**8**
(500 MHz, $CDCl_3$)

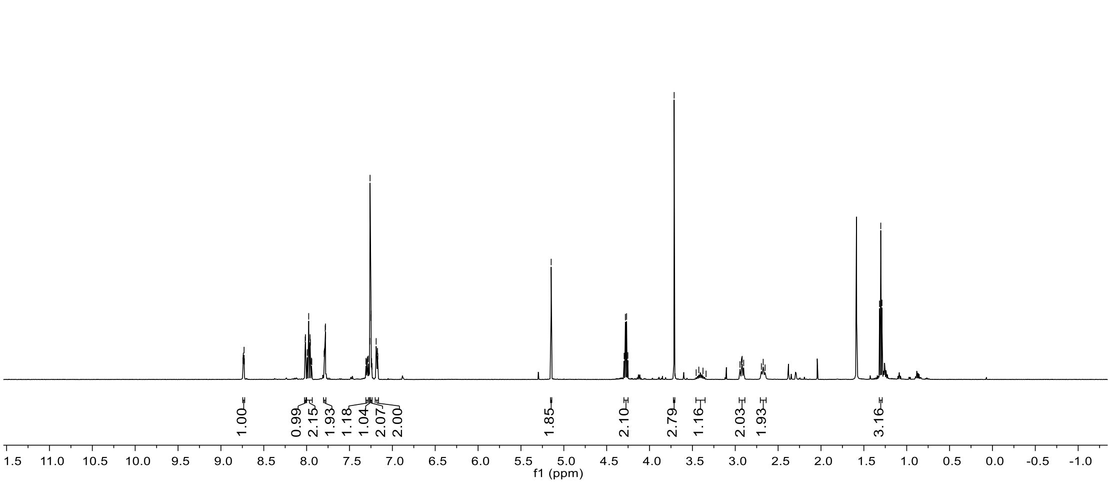

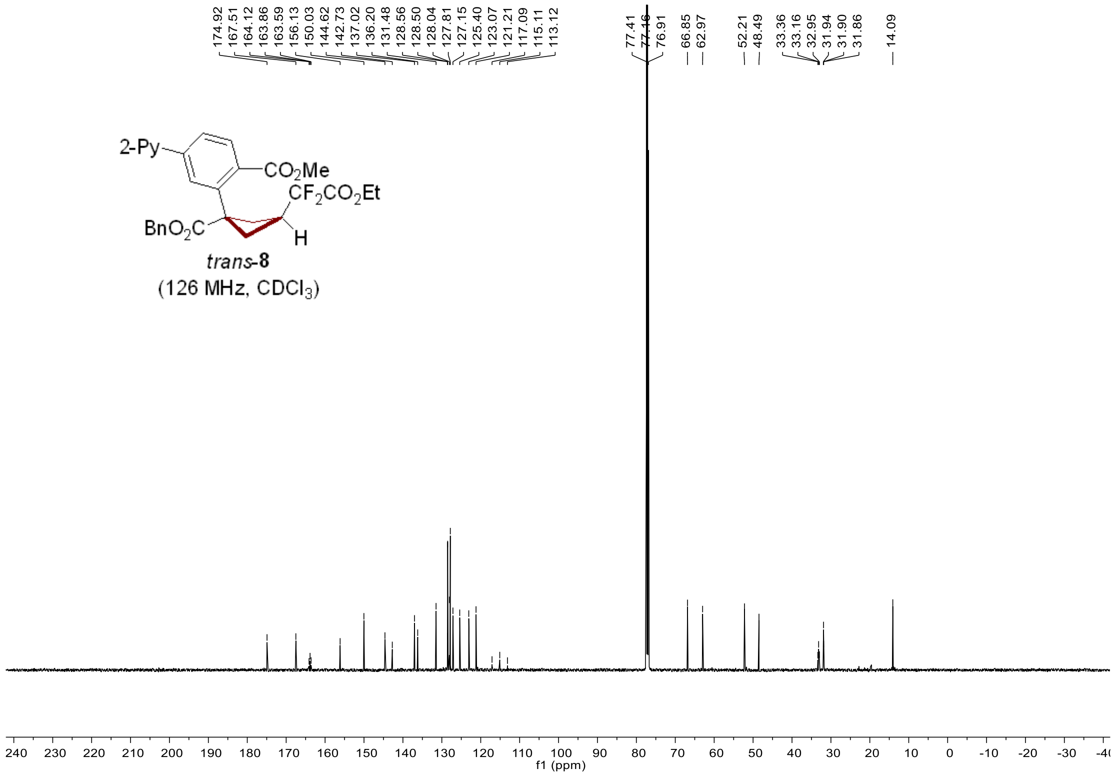
174.92
167.51
164.12
163.86
163.59
156.13
150.03
144.62
142.73
137.02
136.20
131.48
128.56
128.50
128.04
127.81
127.15
125.40
123.07
121.21
117.09
115.11
113.12
77.41
77.16
76.91
66.85
62.97
52.21
48.49
33.36
33.16
32.95
31.94
31.90
31.86
14.09
2-Py
CO2Me
CF2CO2Et
BnO2C
H
trans-8
(126 MHz, CDCl3)
f1 (ppm)

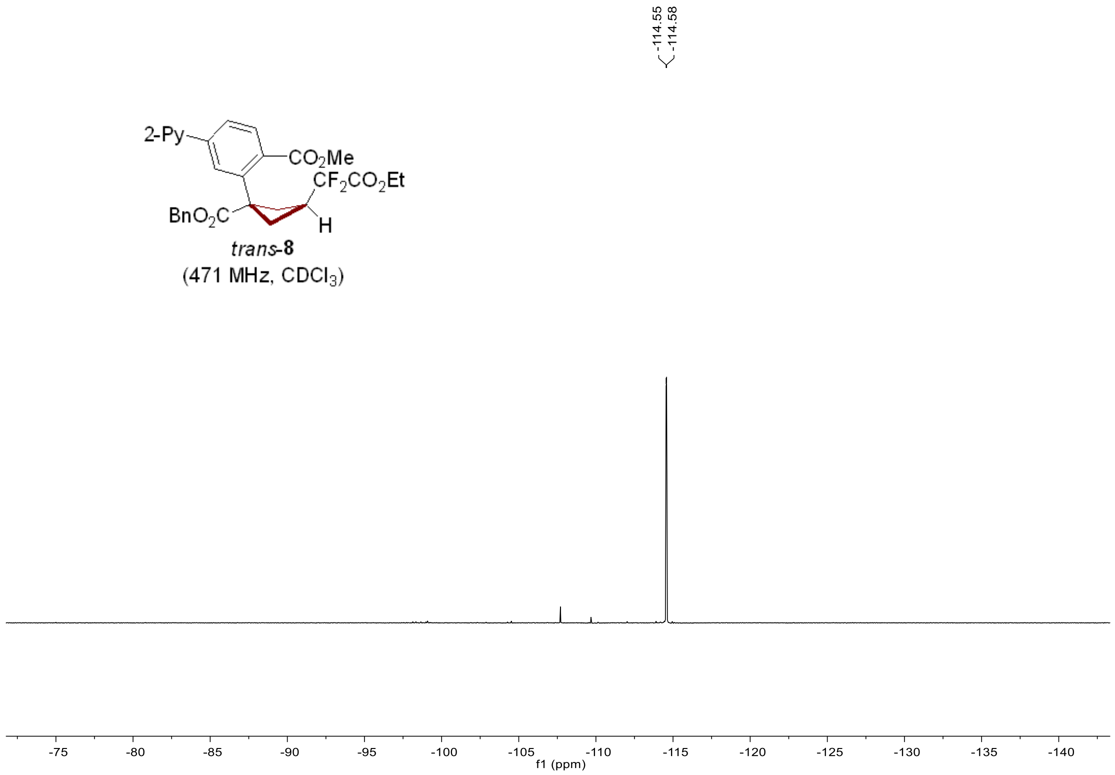
-114.55
-114.58
2-Py
CO2Me
CF2CO2Et
BnO2C
H
trans-8
(471 MHz, CDCl3)
f1 (ppm)

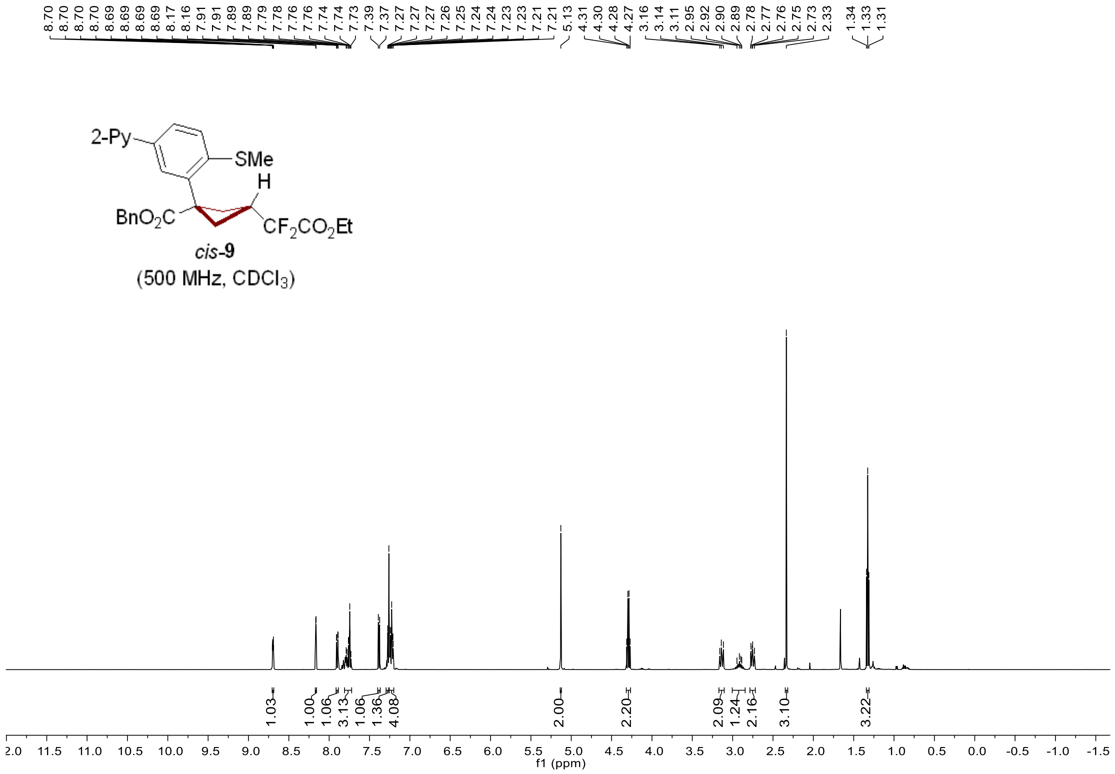
2-Py
SMe
H
BnO2C
CF2CO2Et
cis-9
(500 MHz, CDCl3)
f1 (ppm)

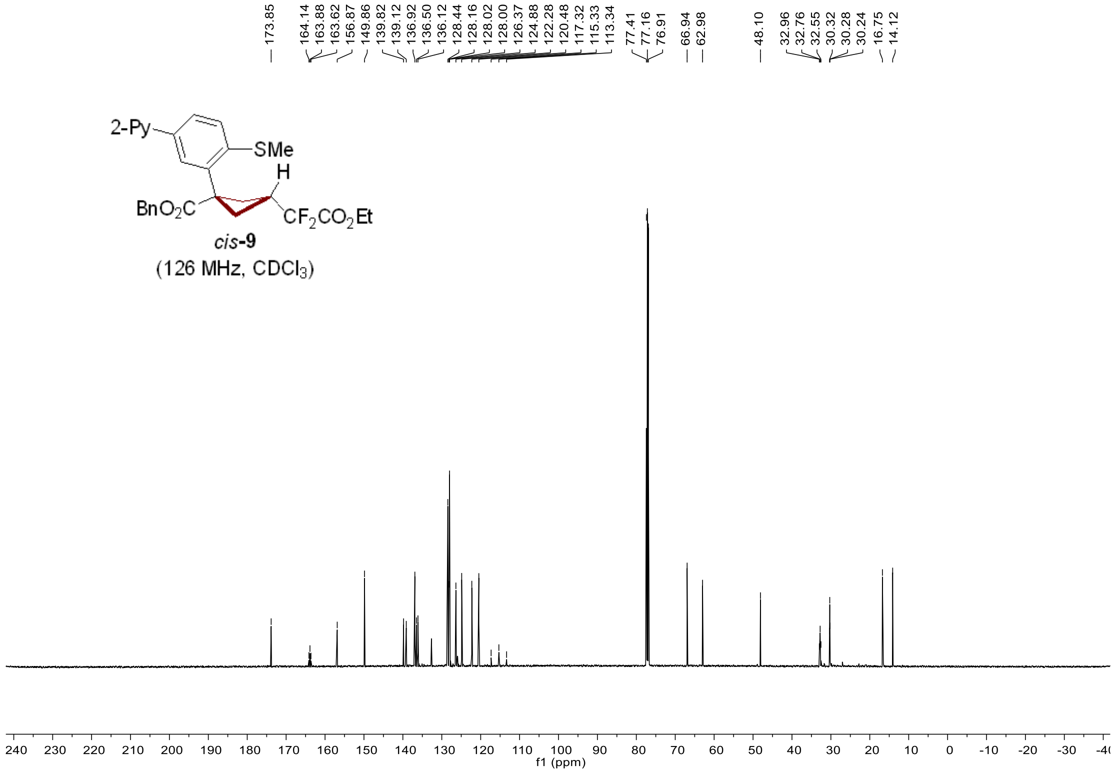
2-Py
SMe
H
BnO2C
CF2CO2Et
cis-9
(126 MHz, CDCl3)
f1 (ppm)

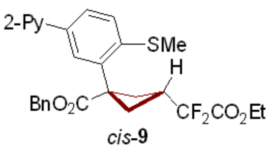


*cis*-**9**
(471 MHz, $CDCl_3$)

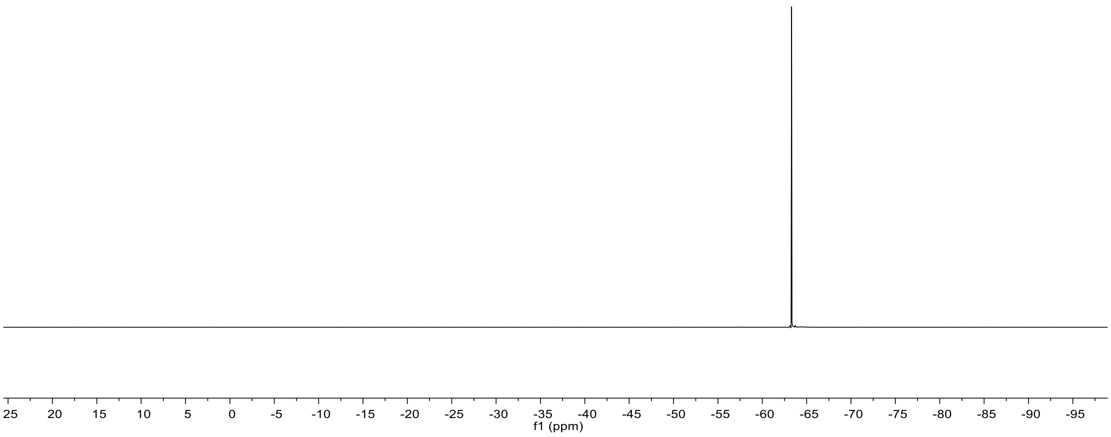


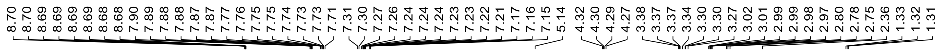


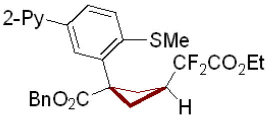


*trans*-**9**
(500 MHz, $CDCl_3$)

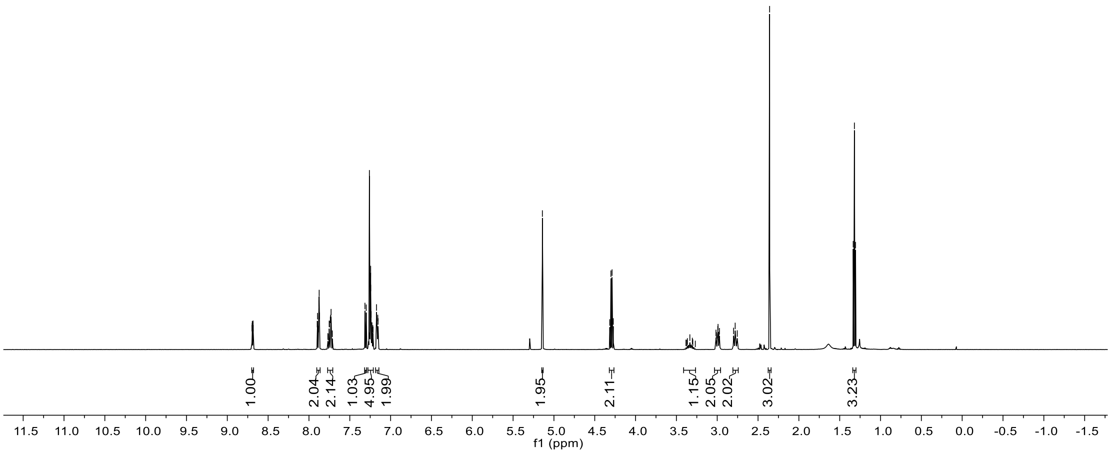

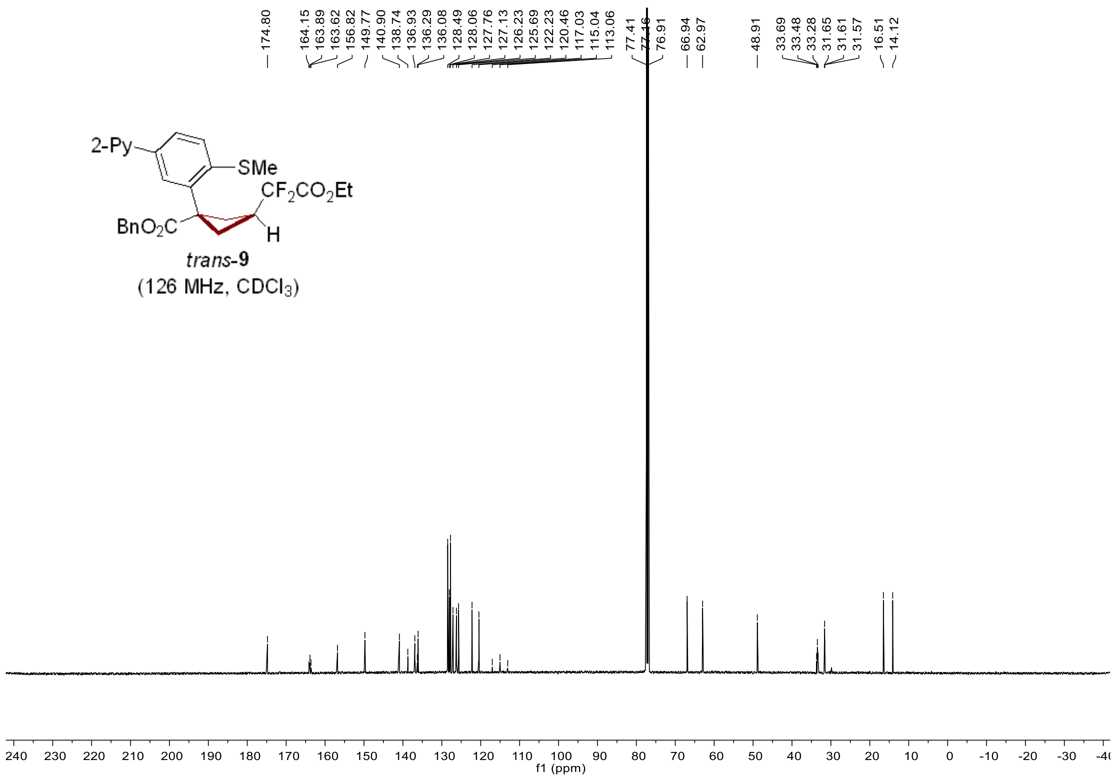
174.80
164.15
163.89
163.62
156.82
149.77
140.90
138.74
136.93
136.29
136.08
128.49
128.06
127.76
127.13
126.23
125.69
122.23
120.46
117.03
115.04
113.06
77.41
77.16
76.91
66.94
62.97
48.91
33.69
33.48
33.28
31.65
31.61
31.57
16.51
14.12
2-Py
SMe
$CF_2CO_2Et$
$BnO_2C$
H
*trans*-**9**
(126 MHz, $CDCl_3$)
f1 (ppm)

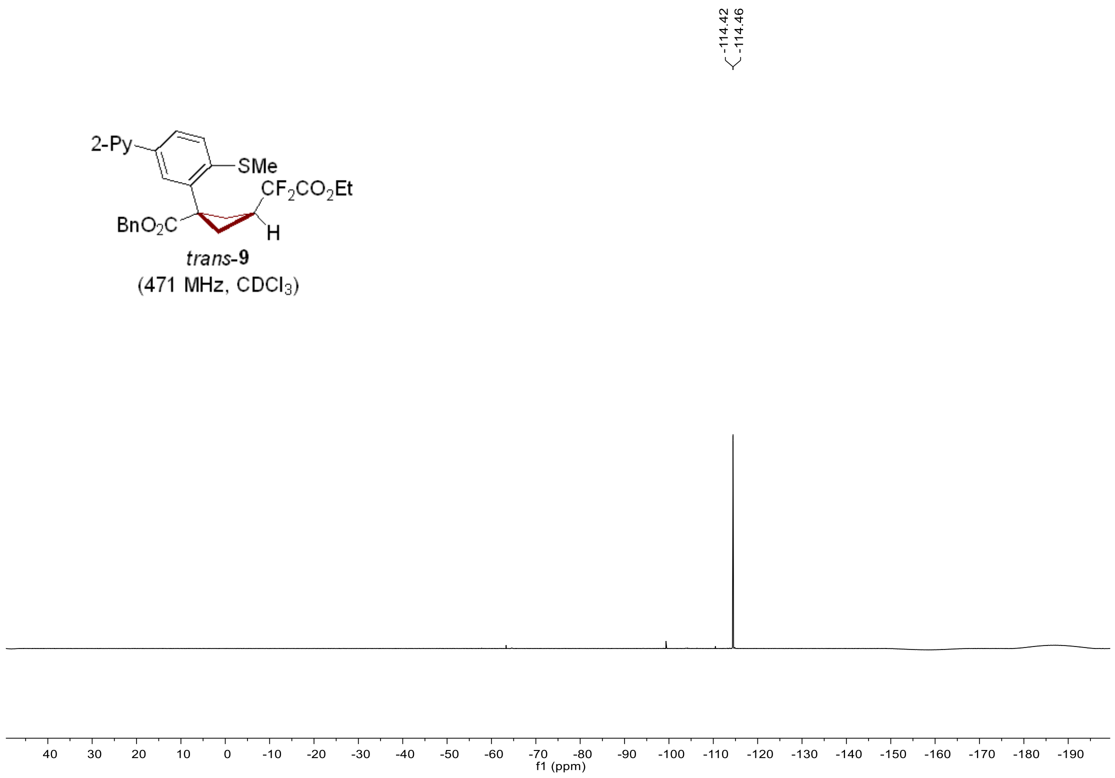
-114.42
-114.46
2-Py
SMe
$CF_2CO_2Et$
$BnO_2C$
H
*trans*-**9**
(471 MHz, $CDCl_3$)
f1 (ppm)

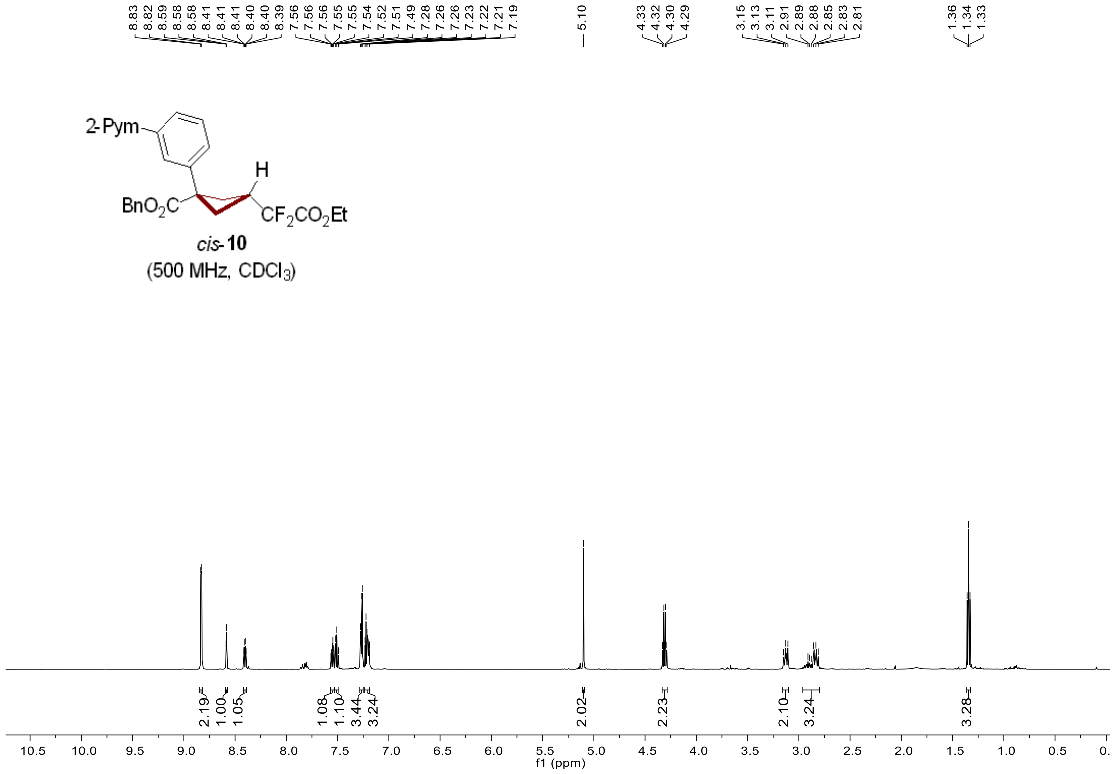


*cis*-**10**
(500 MHz, $CDCl_3$)

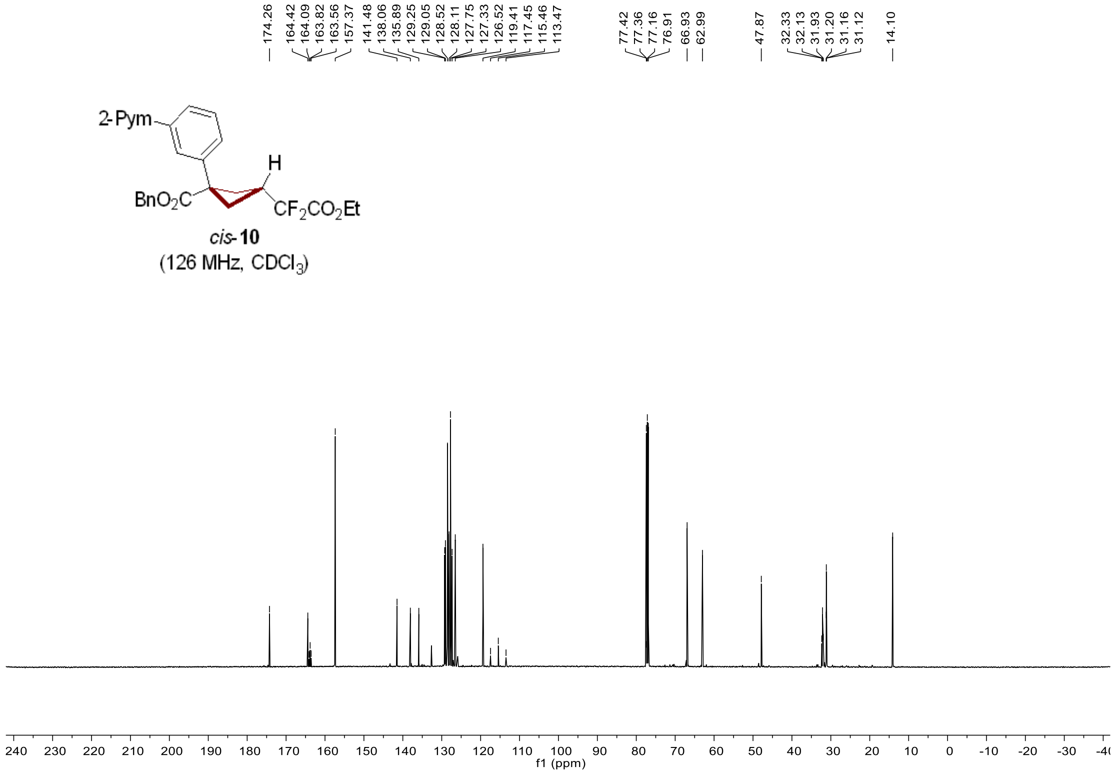


*cis*-**10**
(126 MHz, $CDCl_3$)

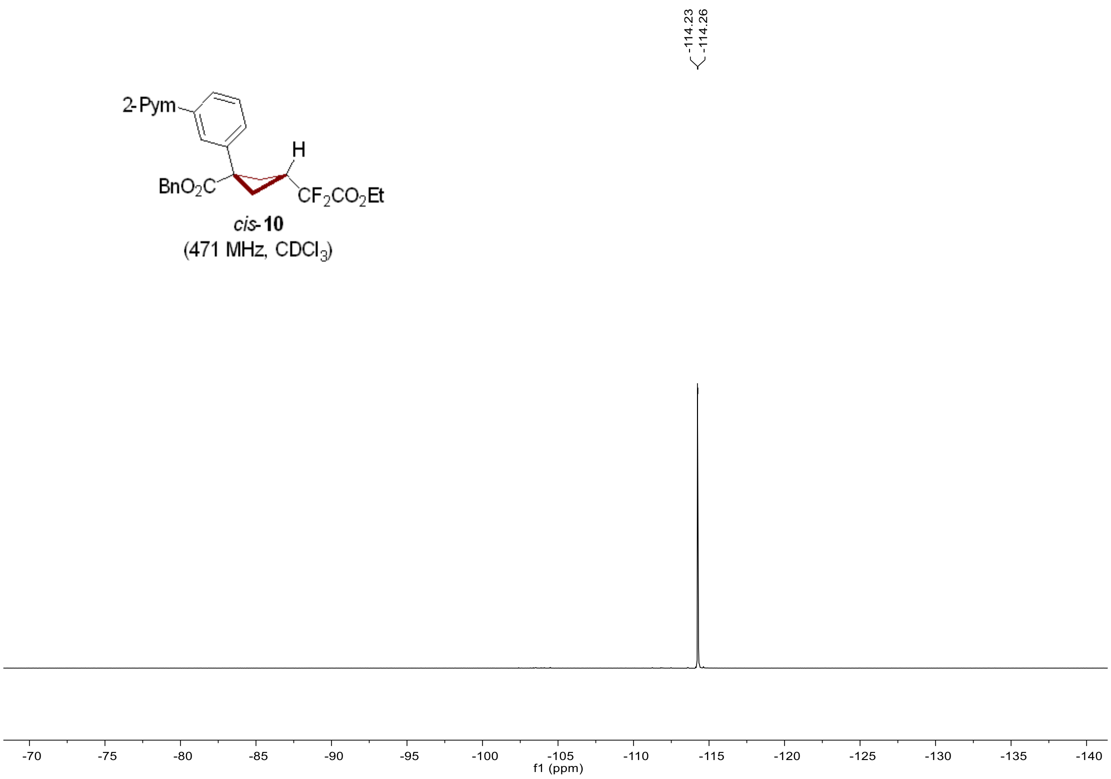

-114.23
-114.26
2-Pym
H
BnO$_2$C
CF$_2$CO$_2$Et
*cis*-**10**
(471 MHz, CDCl$_3$)
-70 -75 -80 -85 -90 -95 -100 -105 -110 -115 -120 -125 -130 -135 -140
f1 (ppm)


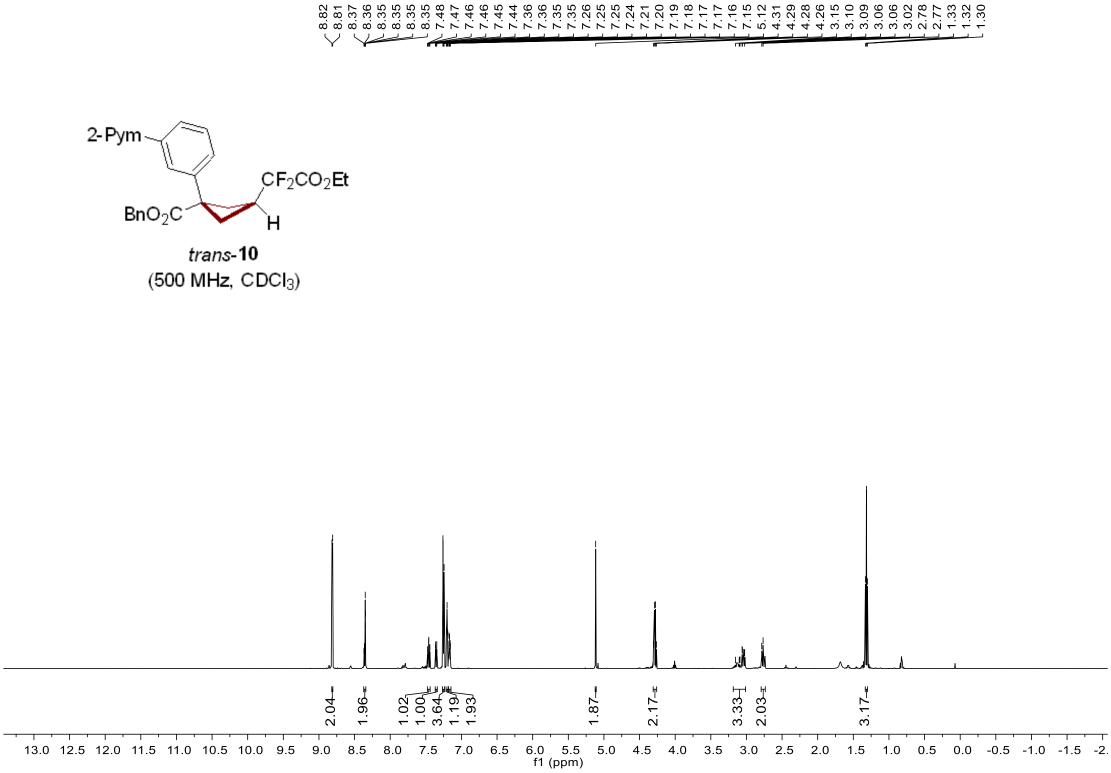

8.82 8.81 8.37 8.36 8.35 8.35 8.35 7.48 7.47 7.46 7.46 7.45 7.44 7.36 7.36 7.35 7.35 7.26 7.25 7.25 7.24 7.21 7.20 7.19 7.18 7.17 7.17 7.16 7.15 5.12 4.31 4.29 4.28 4.26 3.15 3.10 3.09 3.06 3.02 2.78 2.77 1.33 1.32 1.30
2-Pym
CF$_2$CO$_2$Et
BnO$_2$C
H
*trans*-**10**
(500 MHz, CDCl$_3$)
2.04 1.96 1.02 1.00 3.64 1.19 1.93 1.87 2.17 3.33 2.03 3.17
13.0 12.5 12.0 11.5 11.0 10.5 10.0 9.5 9.0 8.5 8.0 7.5 7.0 6.5 6.0 5.5 5.0 4.5 4.0 3.5 3.0 2.5 2.0 1.5 1.0 0.5 0.0 -0.5 -1.0 -1.5 -2.
f1 (ppm)

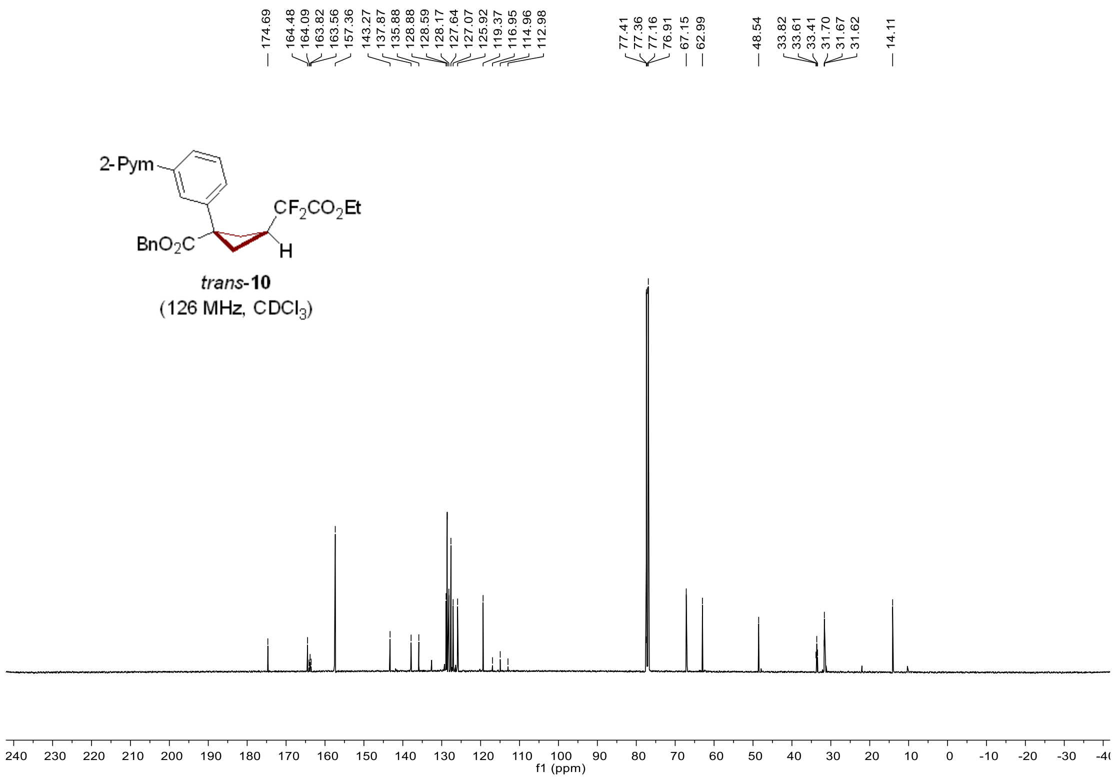
174.69
164.48
164.09
163.82
163.56
157.36
143.27
137.87
135.88
128.88
128.59
128.17
127.64
127.07
125.92
119.37
116.95
114.96
112.98
77.41
77.36
77.16
76.91
67.15
62.99
48.54
33.82
33.61
33.41
31.70
31.67
31.62
14.11
2-Pym
$CF_2CO_2Et$
$BnO_2C$
H
*trans*-**10**
(126 MHz, $CDCl_3$)
240 230 220 210 200 190 180 170 160 150 140 130 120 110 100 90 80 70 60 50 40 30 20 10 0 -10 -20 -30 -40
f1 (ppm)

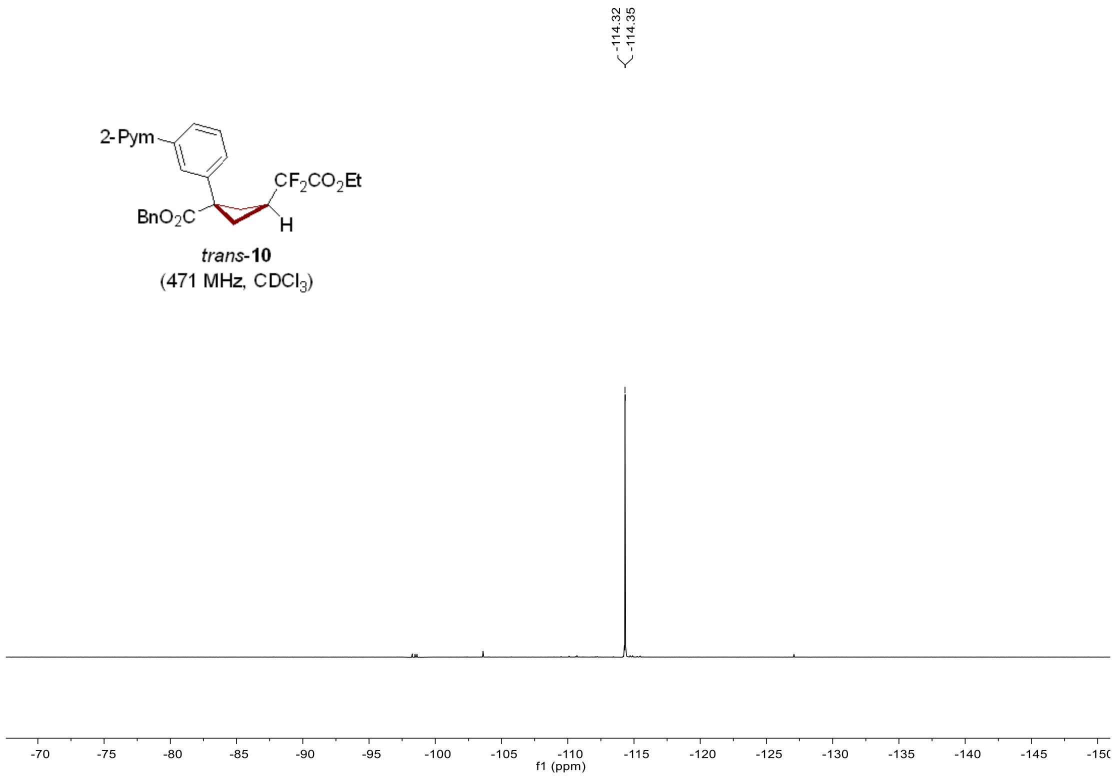
-114.32
-114.35
2-Pym
$CF_2CO_2Et$
$BnO_2C$
H
*trans*-**10**
(471 MHz, $CDCl_3$)
-70 -75 -80 -85 -90 -95 -100 -105 -110 -115 -120 -125 -130 -135 -140 -145 -150
f1 (ppm)

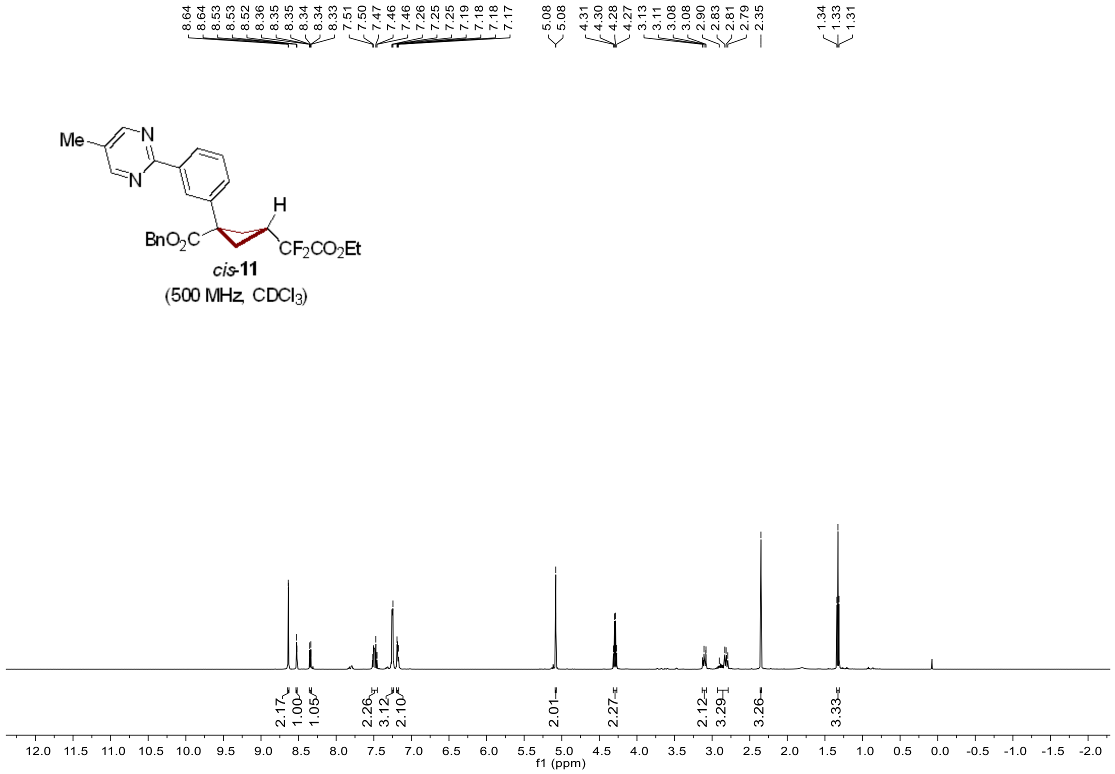
8.64 8.64 8.53 8.53 8.52 8.36 8.35 8.35 8.34 8.34 8.33 7.51 7.50 7.47 7.46 7.46 7.26 7.25 7.25 7.19 7.18 7.17
5.08 5.08 4.31 4.30 4.28 4.27 3.13 3.11 3.08 2.90 2.83 2.81 2.79 2.35 1.34 1.33 1.31
Me
N
N
H
BnO2C
CF2CO2Et
cis-11
(500 MHz, CDCl3)
2.17 1.00 1.05 2.26 3.12 2.10 2.01 2.27 2.12 3.29 3.26 3.33
12.0 11.5 11.0 10.5 10.0 9.5 9.0 8.5 8.0 7.5 7.0 6.5 6.0 5.5 5.0 4.5 4.0 3.5 3.0 2.5 2.0 1.5 1.0 0.5 0.0 -0.5 -1.0 -1.5 -2.0
f1 (ppm)

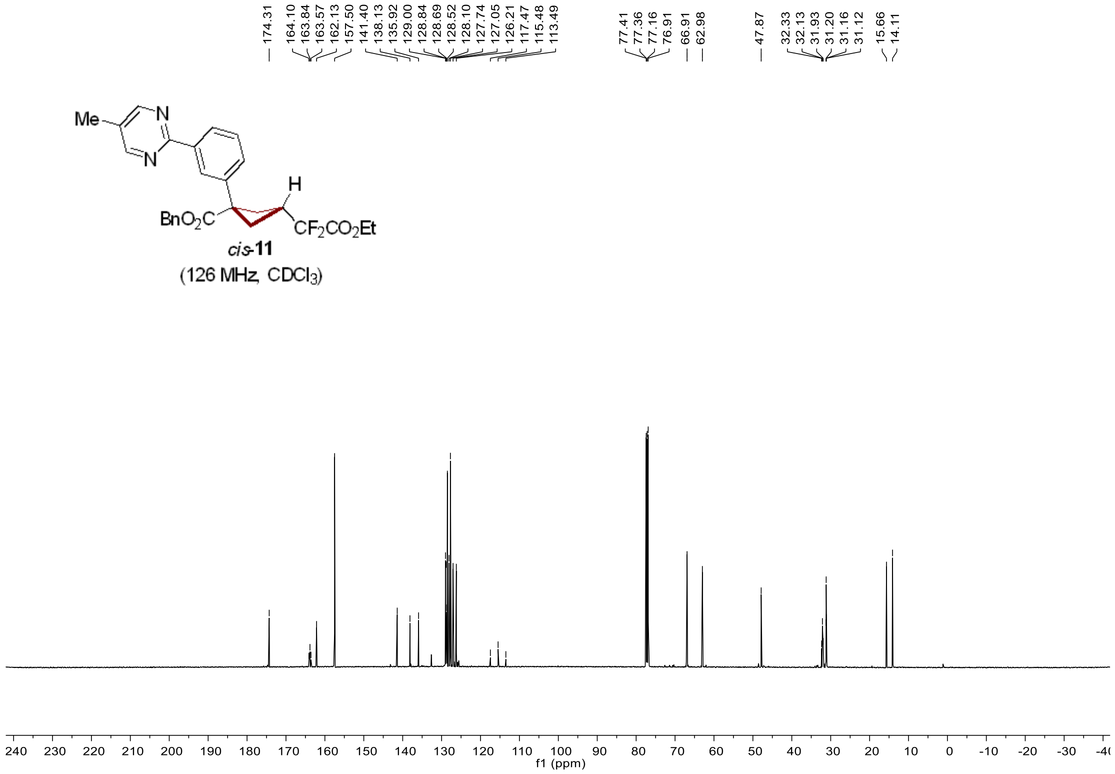
174.31 164.10 163.84 163.57 162.13 157.50 141.40 138.13 135.92 129.00 128.84 128.69 128.52 128.10 127.74 127.05 126.21 117.47 115.48 113.49
77.41 77.36 77.16 76.91 66.91 62.98 47.87 32.33 32.13 31.93 31.20 31.16 31.12 15.66 14.11
Me
N
N
H
BnO2C
CF2CO2Et
cis-11
(126 MHz, CDCl3)
240 230 220 210 200 190 180 170 160 150 140 130 120 110 100 90 80 70 60 50 40 30 20 10 0 -10 -20 -30
f1 (ppm)

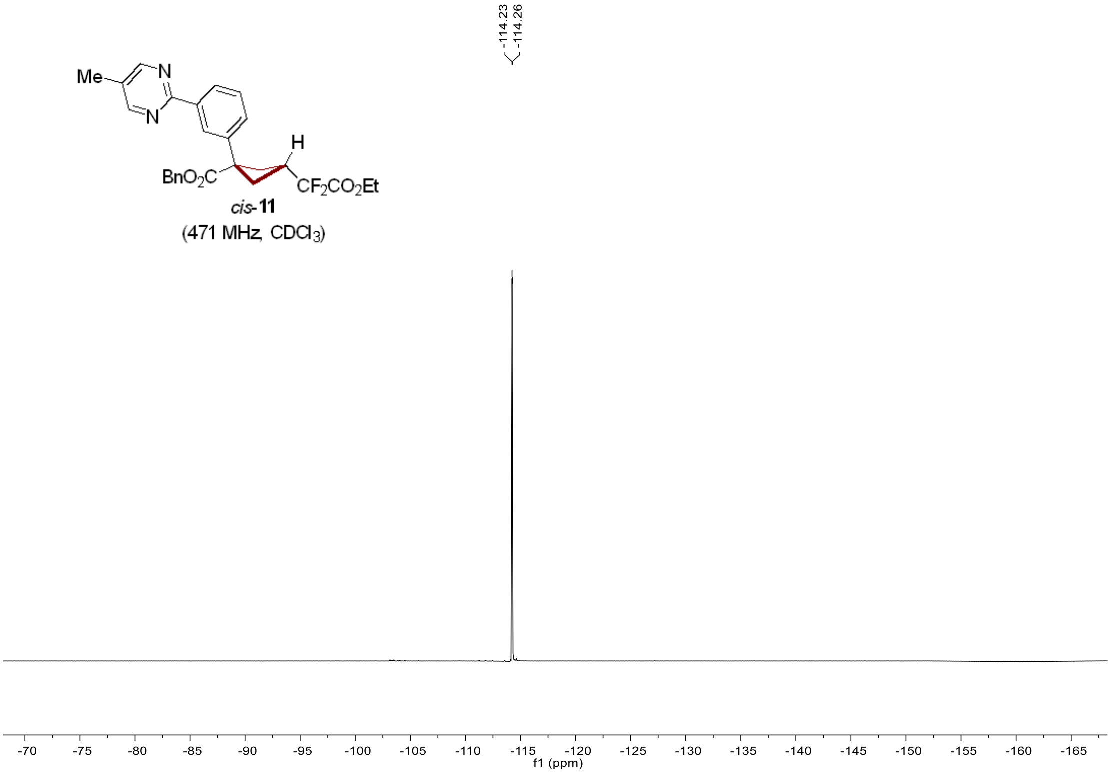


*cis*-**11**
(471 MHz, $CDCl_3$)

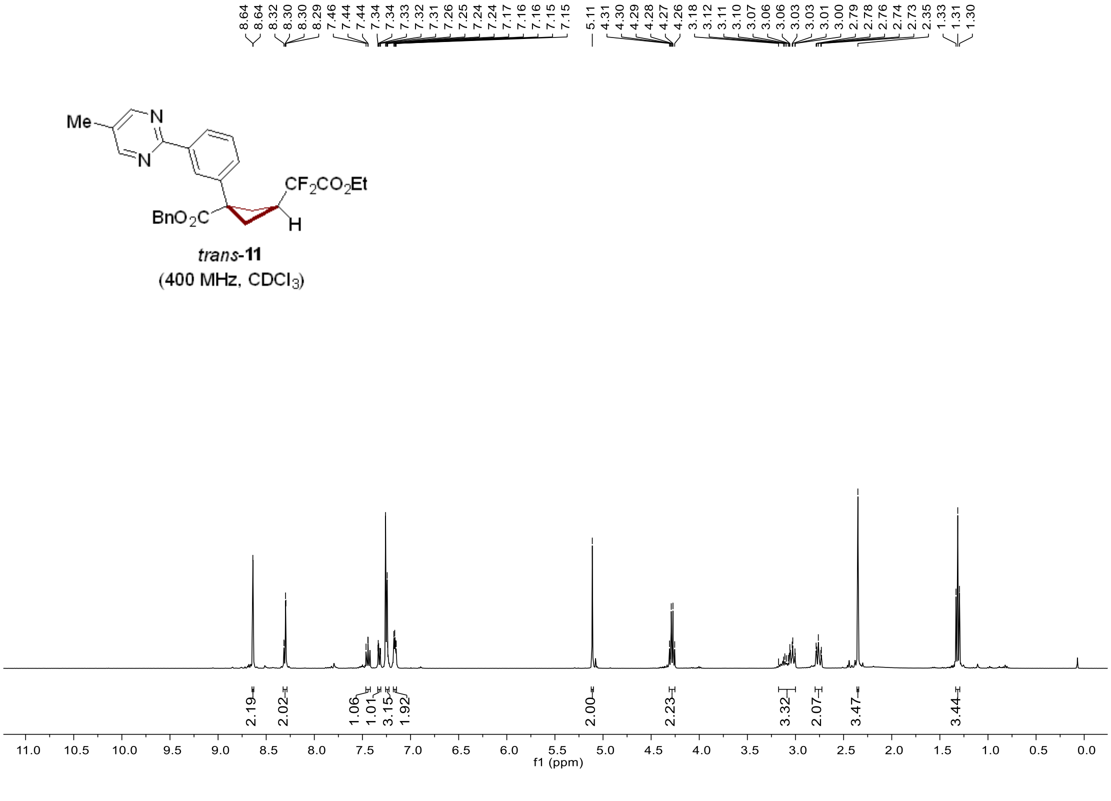


*trans*-**11**
(400 MHz, $CDCl_3$)

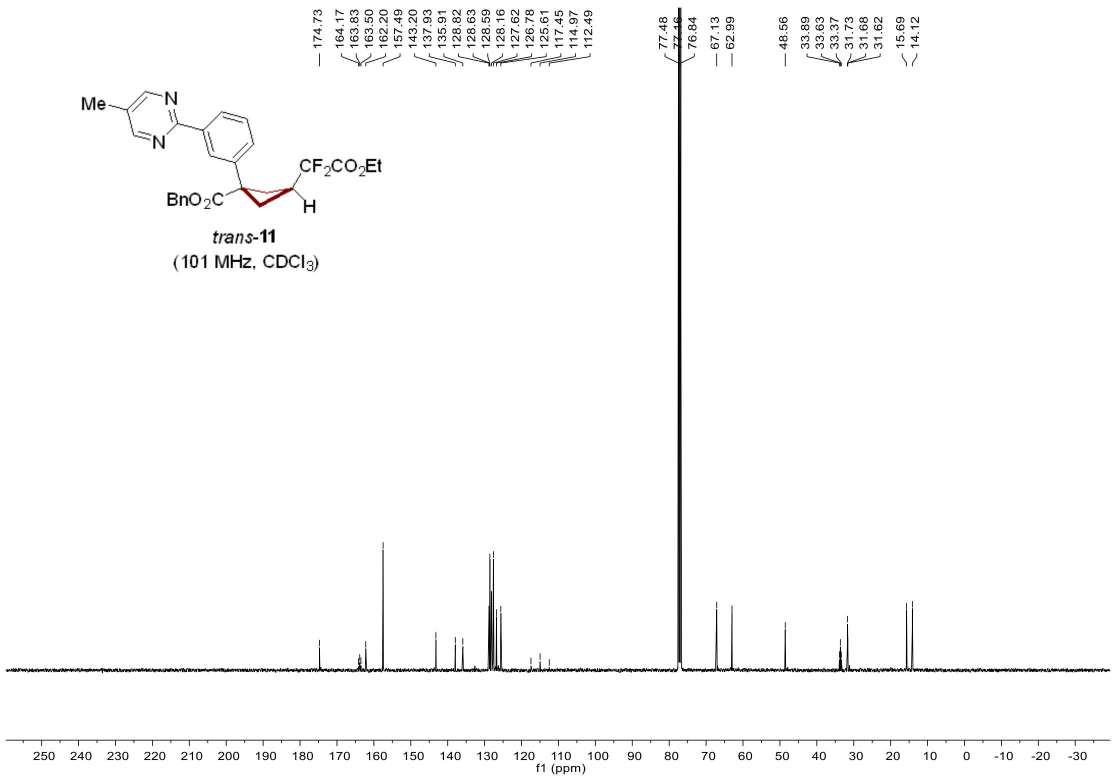
174.73
164.17
163.83
163.50
162.20
157.49
143.20
137.93
135.91
128.82
128.63
128.59
128.16
127.62
126.78
125.61
117.45
114.97
112.49
77.48
77.16
76.84
67.13
62.99
48.56
33.89
33.63
33.37
31.73
31.68
31.62
15.69
14.12
Me
N
N
$CF_2CO_2Et$
$BnO_2C$
H
*trans*-**11**
(101 MHz, $CDCl_3$)
f1 (ppm)

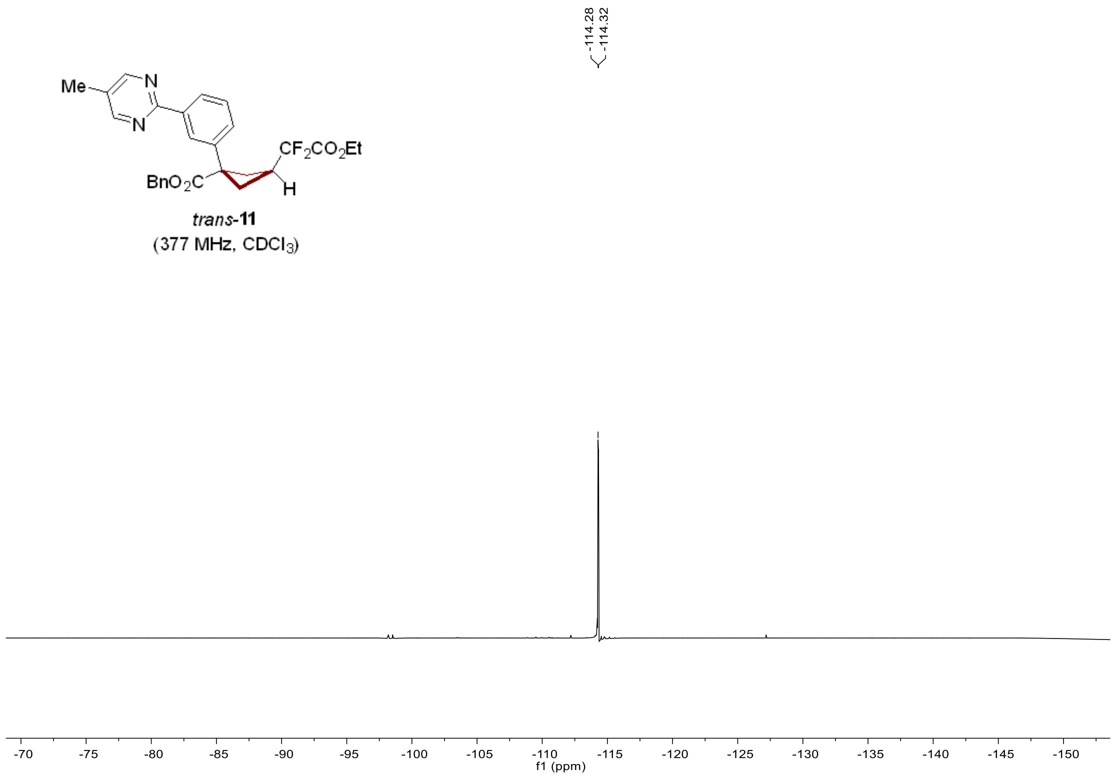
-114.28
-114.32
Me
N
N
$CF_2CO_2Et$
$BnO_2C$
H
*trans*-**11**
(377 MHz, $CDCl_3$)
f1 (ppm)

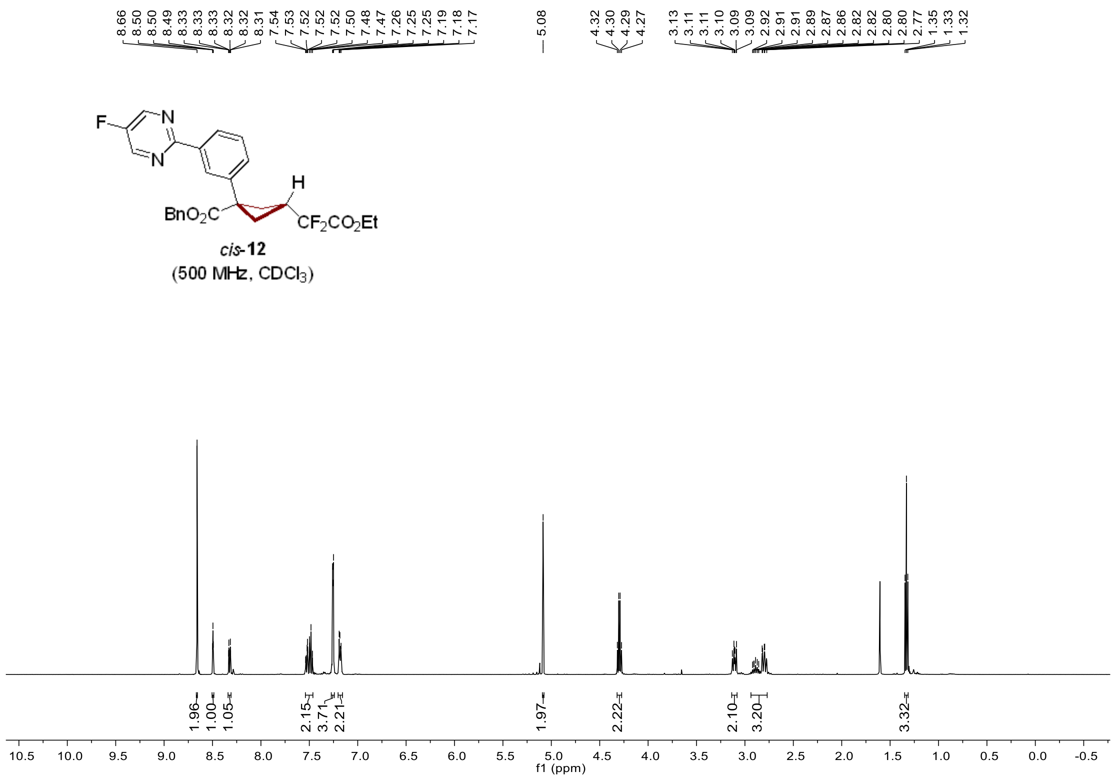
8.66 8.50 8.50 8.49 8.33 8.33 8.33 8.32 8.32 8.31 7.54 7.53 7.52 7.52 7.50 7.48 7.47 7.26 7.25 7.25 7.19 7.18 7.17
5.08
4.32 4.30 4.29 4.27
3.13 3.11 3.11 3.10 3.09 3.09 2.92 2.91 2.91 2.89 2.87 2.86 2.82 2.82 2.80 2.80 2.77 1.35 1.33 1.32
F
N
N
H
$BnO_2C$
$CF_2CO_2Et$
*cis*-**12**
(500 MHz, $CDCl_3$)
1.96 1.00 1.05 2.15 3.71 2.21 1.97 2.22 2.10 3.20 3.32
10.5 10.0 9.5 9.0 8.5 8.0 7.5 7.0 6.5 6.0 5.5 5.0 4.5 4.0 3.5 3.0 2.5 2.0 1.5 1.0 0.5 0.0 -0.5
f1 (ppm)

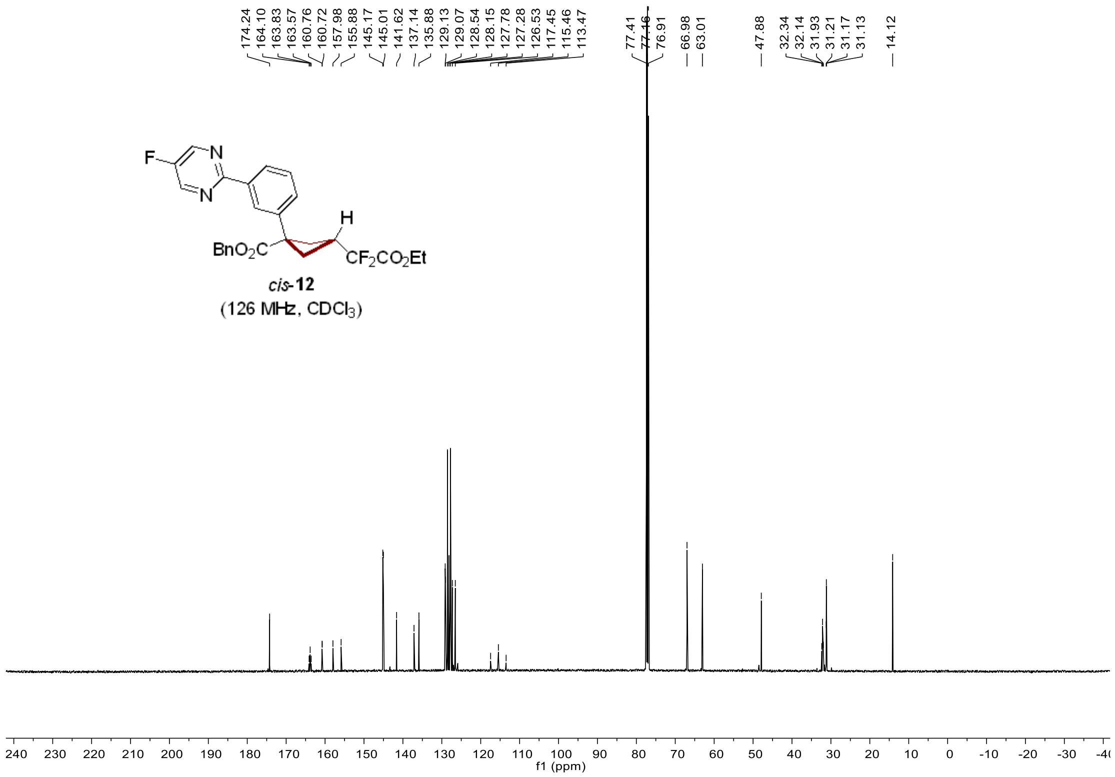
174.24 164.10 163.83 163.57 160.76 160.72 157.98 155.88 145.17 145.01 141.62 137.14 135.88 129.13 129.07 128.54 128.15 127.78 127.28 126.53 117.45 115.46 113.47
77.41 77.16 76.91
66.98 63.01
47.88
32.34 32.14 31.93 31.21 31.17 31.13
14.12
F
N
N
H
$BnO_2C$
$CF_2CO_2Et$
*cis*-**12**
(126 MHz, $CDCl_3$)
240 230 220 210 200 190 180 170 160 150 140 130 120 110 100 90 80 70 60 50 40 30 20 10 0 -10 -20 -30 -40
f1 (ppm)

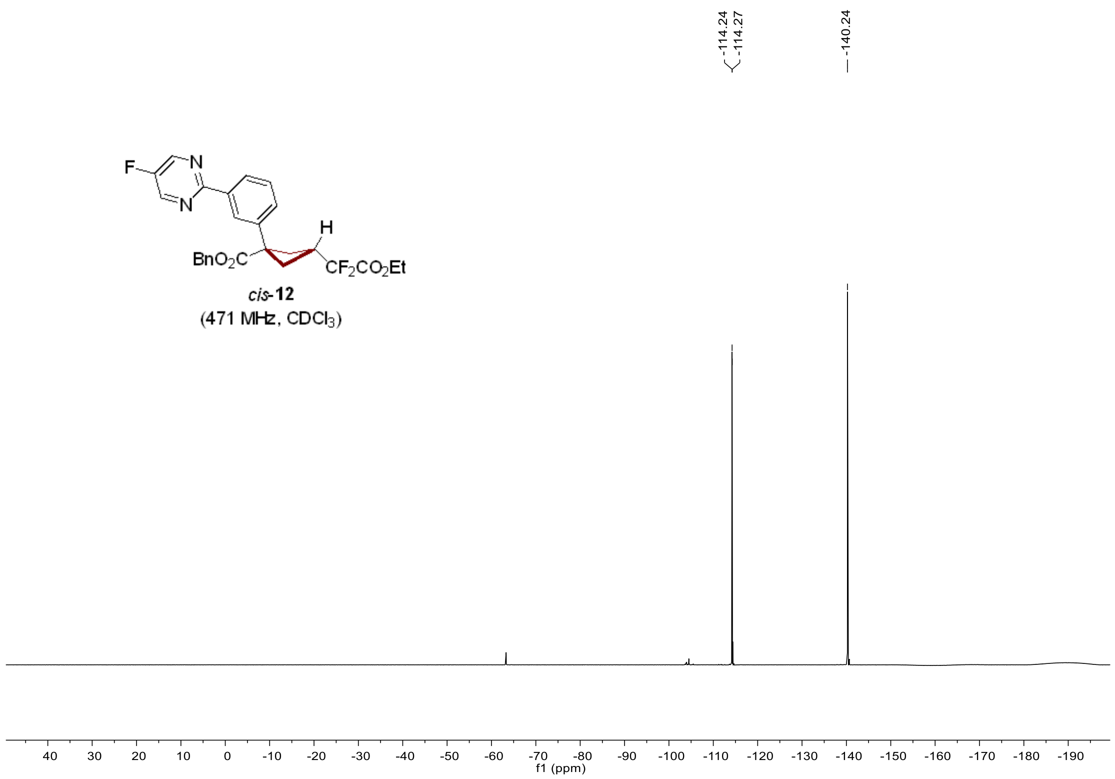
-114.24
-114.27
-140.24
cis-12
(471 MHz, CDCl3)
f1 (ppm)

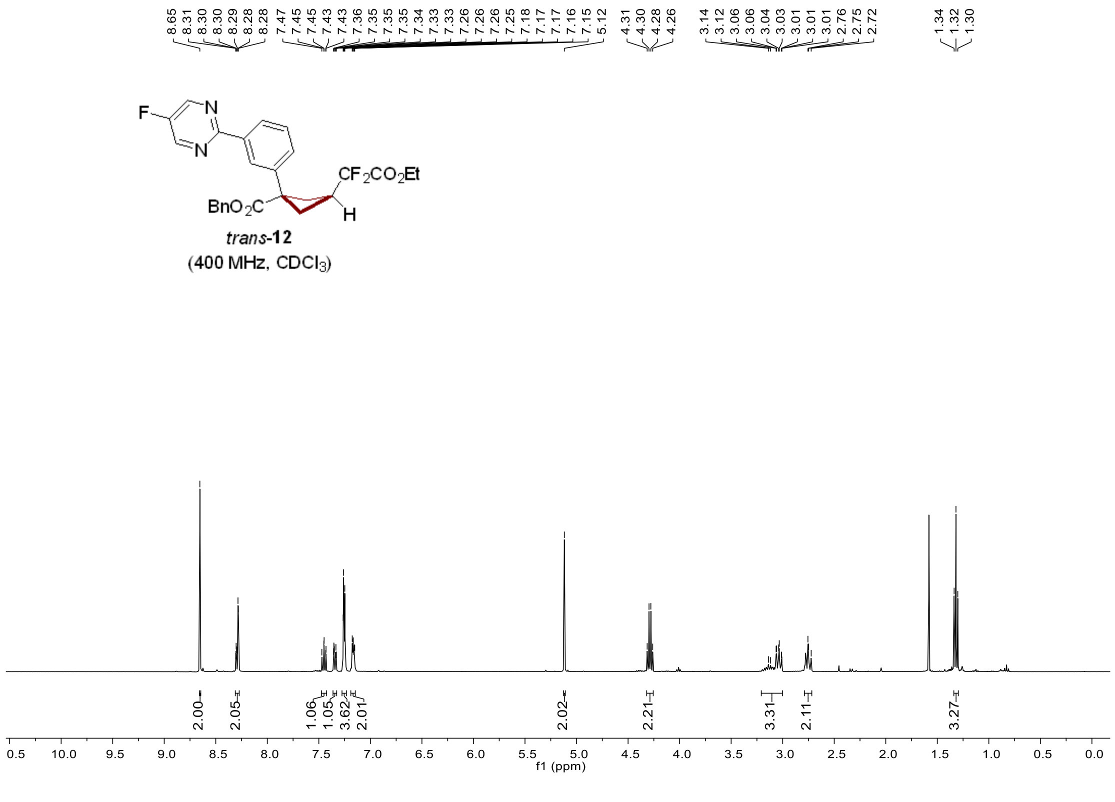
trans-12
(400 MHz, CDCl3)
f1 (ppm)

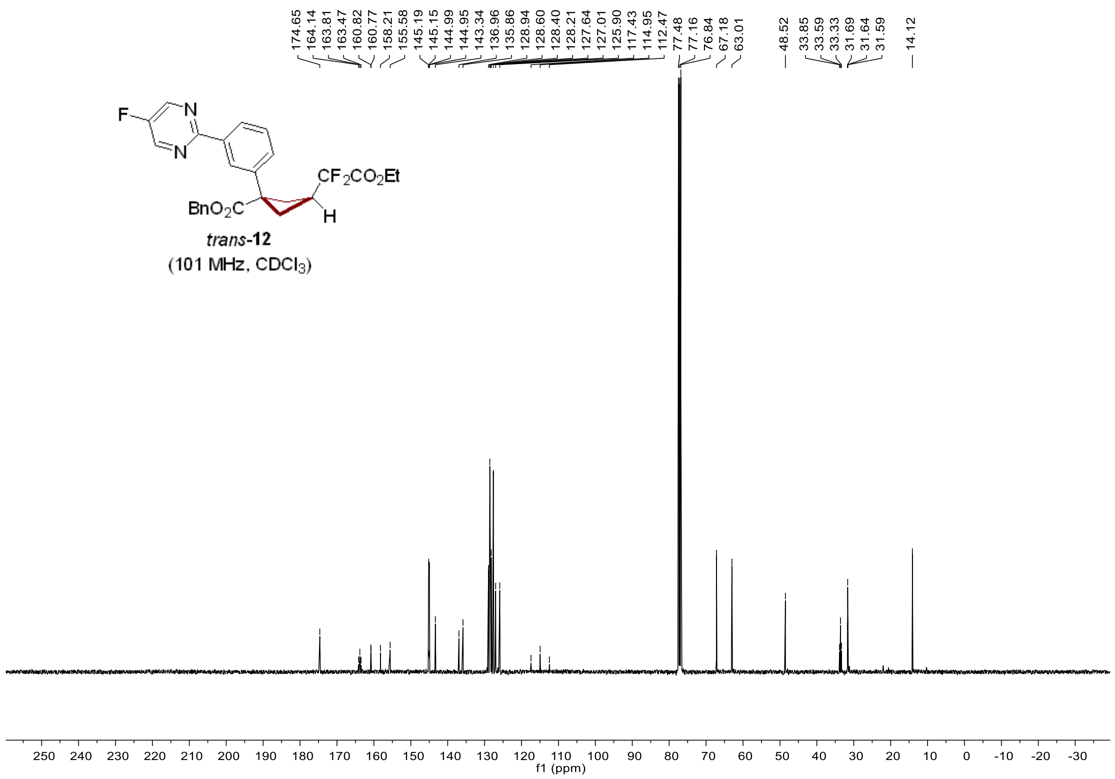
174.65
164.14
163.81
163.47
160.82
160.77
158.21
155.58
145.19
145.15
144.99
144.95
143.34
136.96
135.86
128.94
128.60
128.40
128.21
127.64
127.01
125.90
117.43
114.95
112.47
77.48
77.16
76.84
67.18
63.01
48.52
33.85
33.59
33.33
31.69
31.64
31.59
14.12
F
N
N
$CF_2CO_2Et$
$BnO_2C$
H
*trans*-**12**
(101 MHz, $CDCl_3$)
250 240 230 220 210 200 190 180 170 160 150 140 130 120 110 100 90 80 70 60 50 40 30 20 10 0 -10 -20 -30
f1 (ppm)

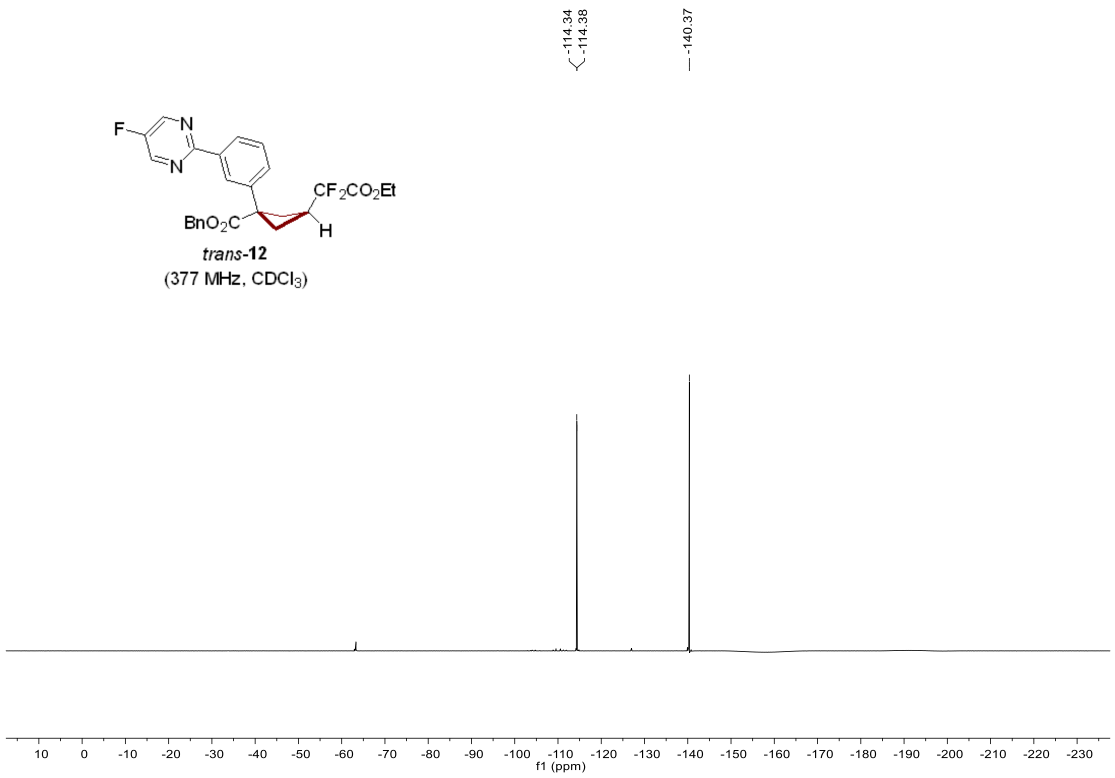
-114.34
-114.38
-140.37
F
N
N
$CF_2CO_2Et$
$BnO_2C$
H
*trans*-**12**
(377 MHz, $CDCl_3$)
10 0 -10 -20 -30 -40 -50 -60 -70 -80 -90 -100 -110 -120 -130 -140 -150 -160 -170 -180 -190 -200 -210 -220 -230
f1 (ppm)

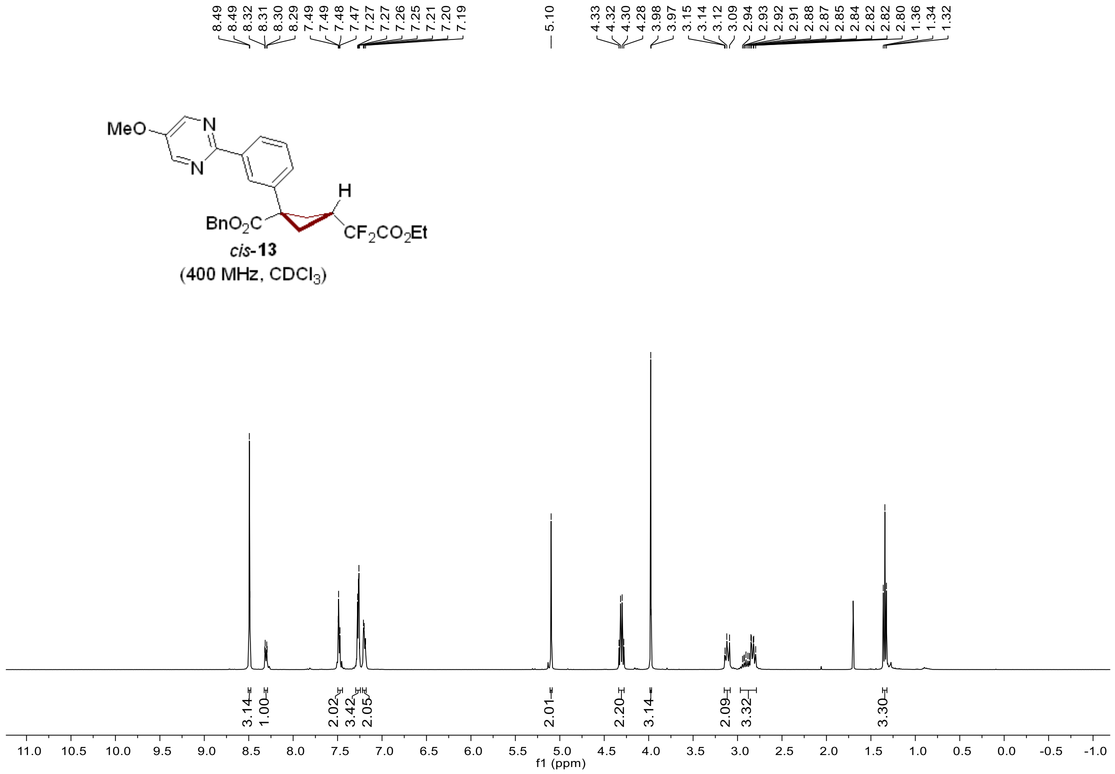
MeO
N
BnO2C
H
CF2CO2Et
cis-13
(400 MHz, CDCl3)
f1 (ppm)

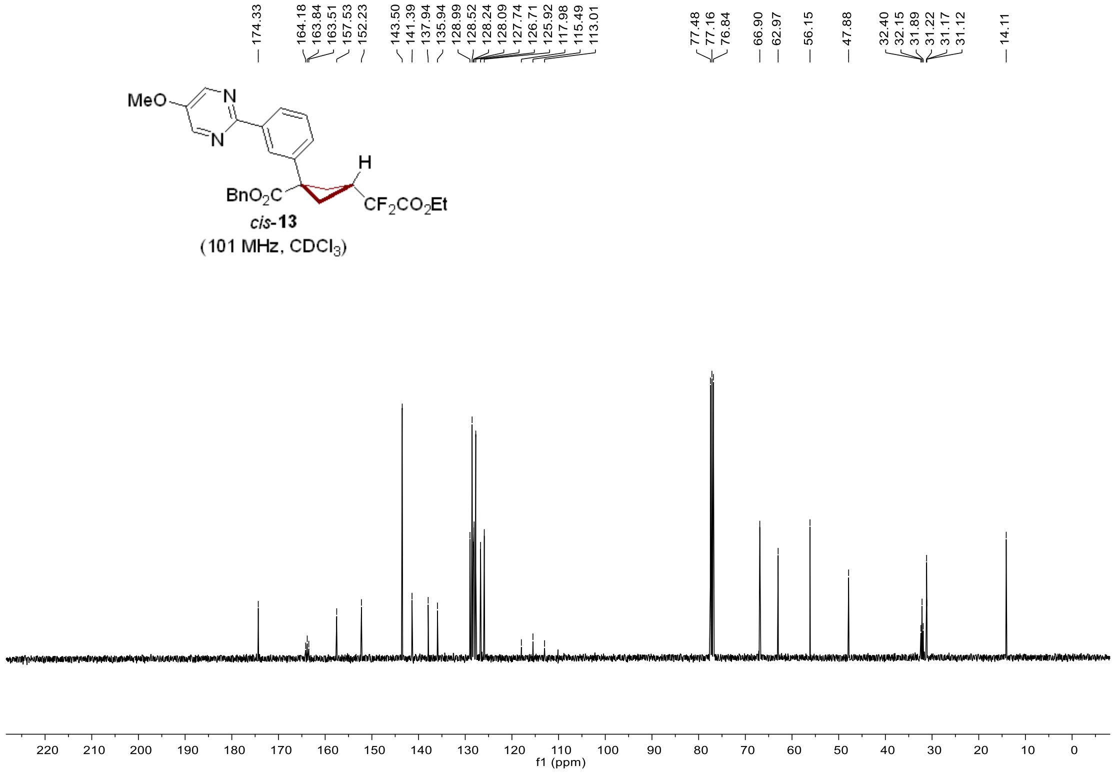
MeO
N
BnO2C
H
CF2CO2Et
cis-13
(101 MHz, CDCl3)
f1 (ppm)

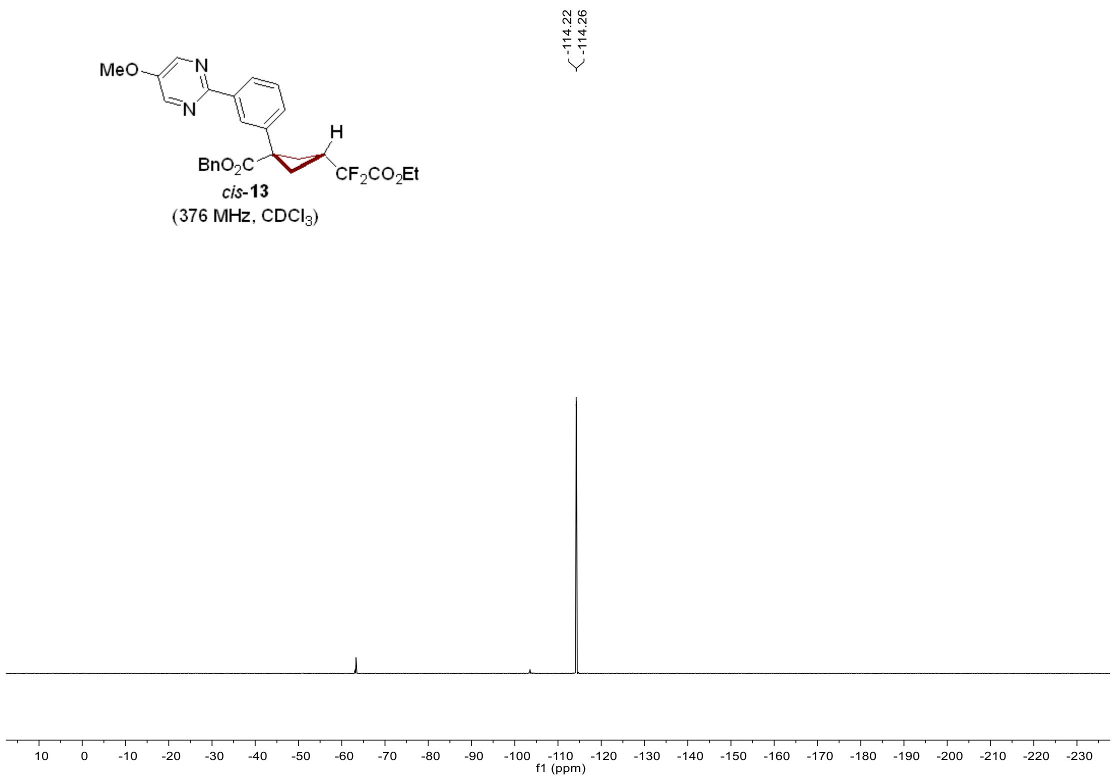

-114.22
-114.26
MeO
N
N
H
BnO2C
CF2CO2Et
cis-13
(376 MHz, CDCl3)
f1 (ppm)


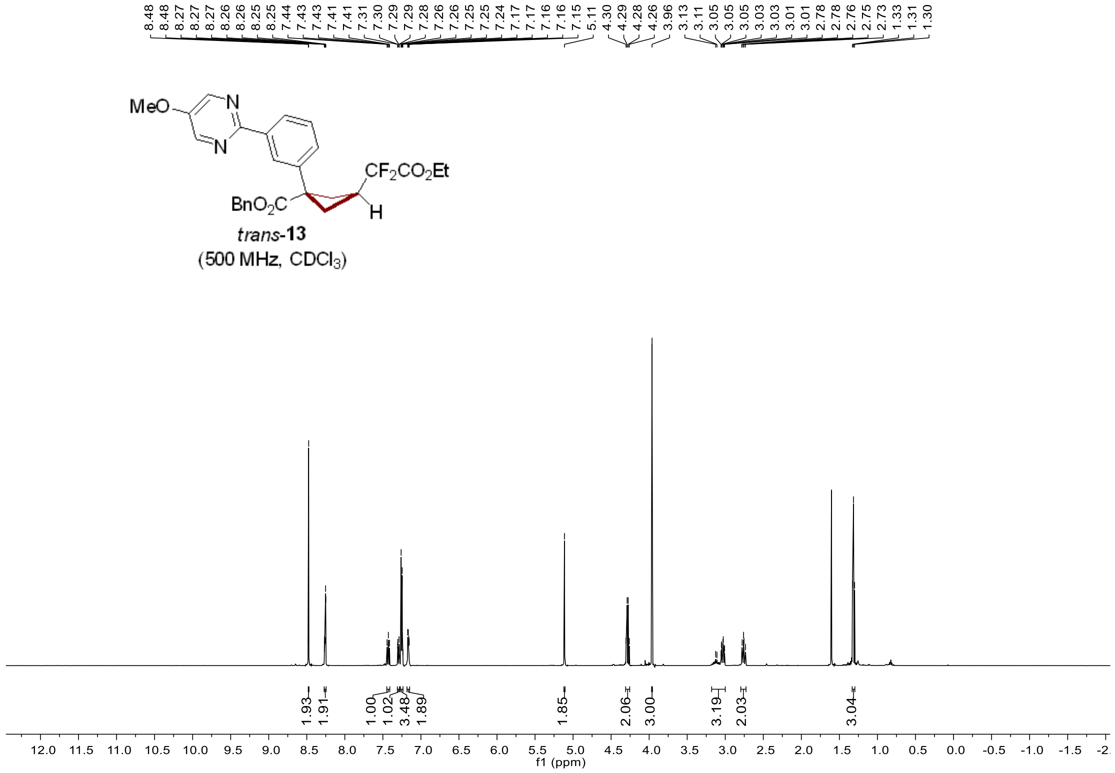

MeO
N
N
CF2CO2Et
BnO2C
H
trans-13
(500 MHz, CDCl3)
f1 (ppm)

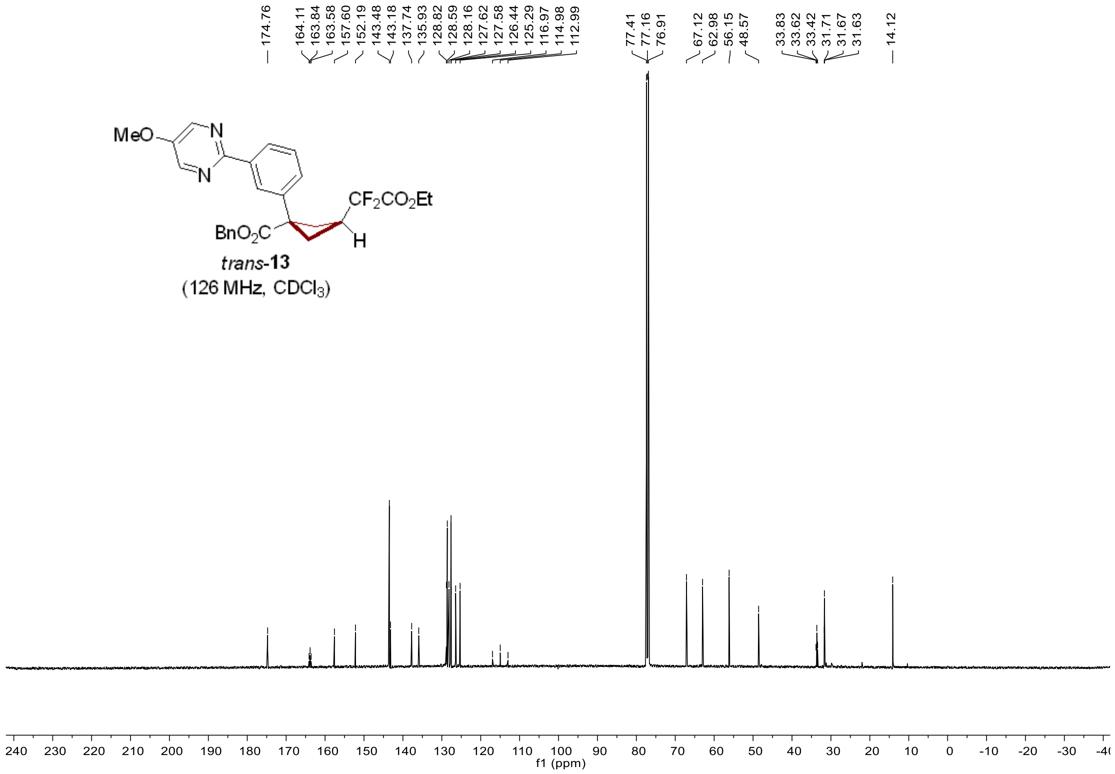
174.76
164.11
163.84
163.58
157.60
152.19
143.48
143.18
137.74
135.93
128.82
128.59
128.16
127.62
127.58
126.44
125.29
116.97
114.98
112.99
77.41
77.16
76.91
67.12
62.98
56.15
48.57
33.83
33.62
33.42
31.71
31.67
31.63
14.12
MeO
N
N
CF2CO2Et
BnO2C
H
trans-13
(126 MHz, CDCl3)
f1 (ppm)

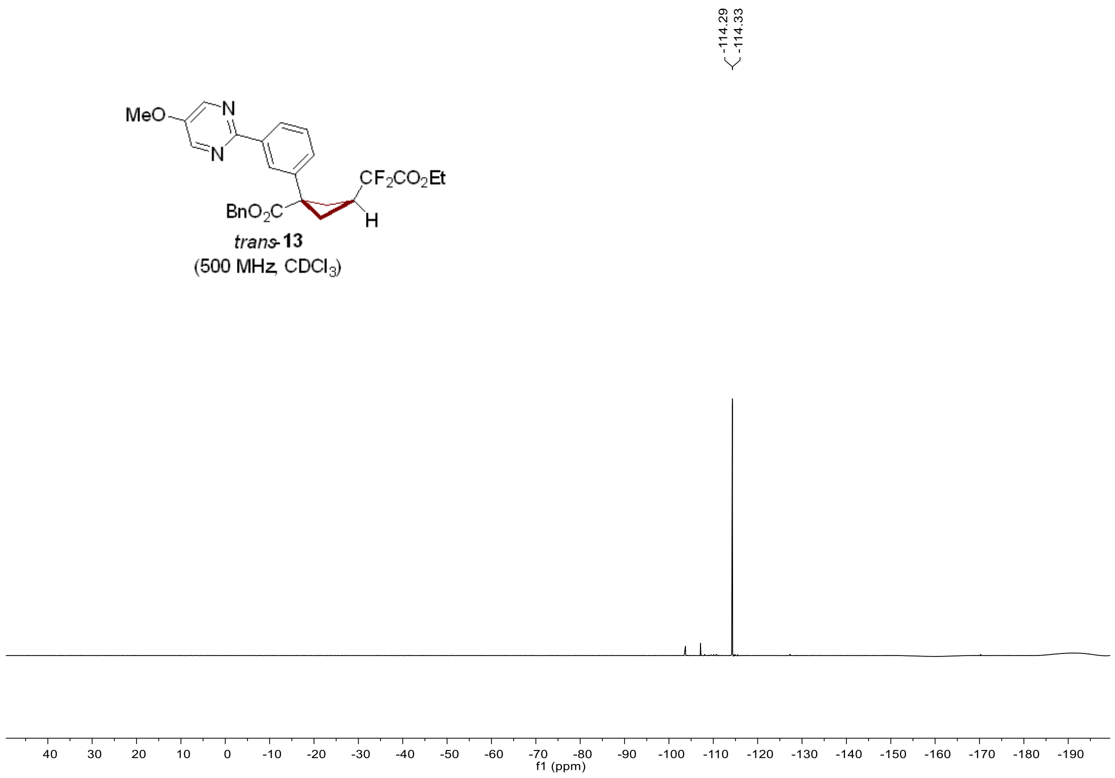
-114.29
-114.33
MeO
N
N
CF2CO2Et
BnO2C
H
trans-13
(500 MHz, CDCl3)
f1 (ppm)

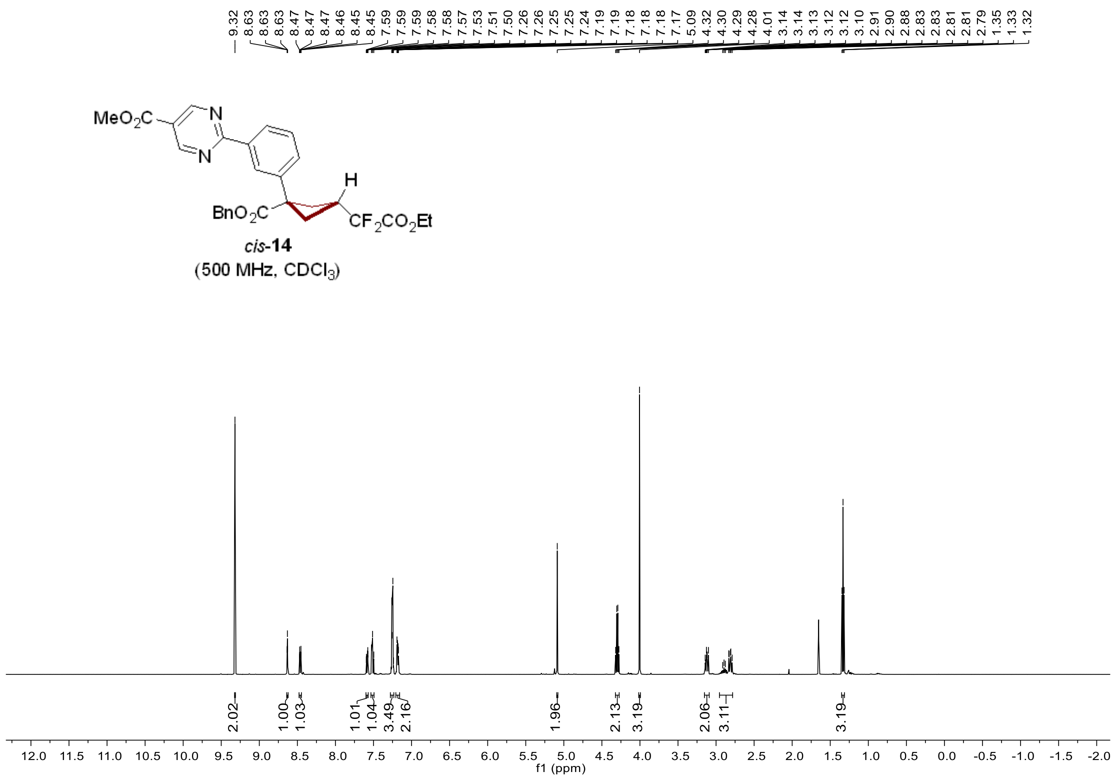

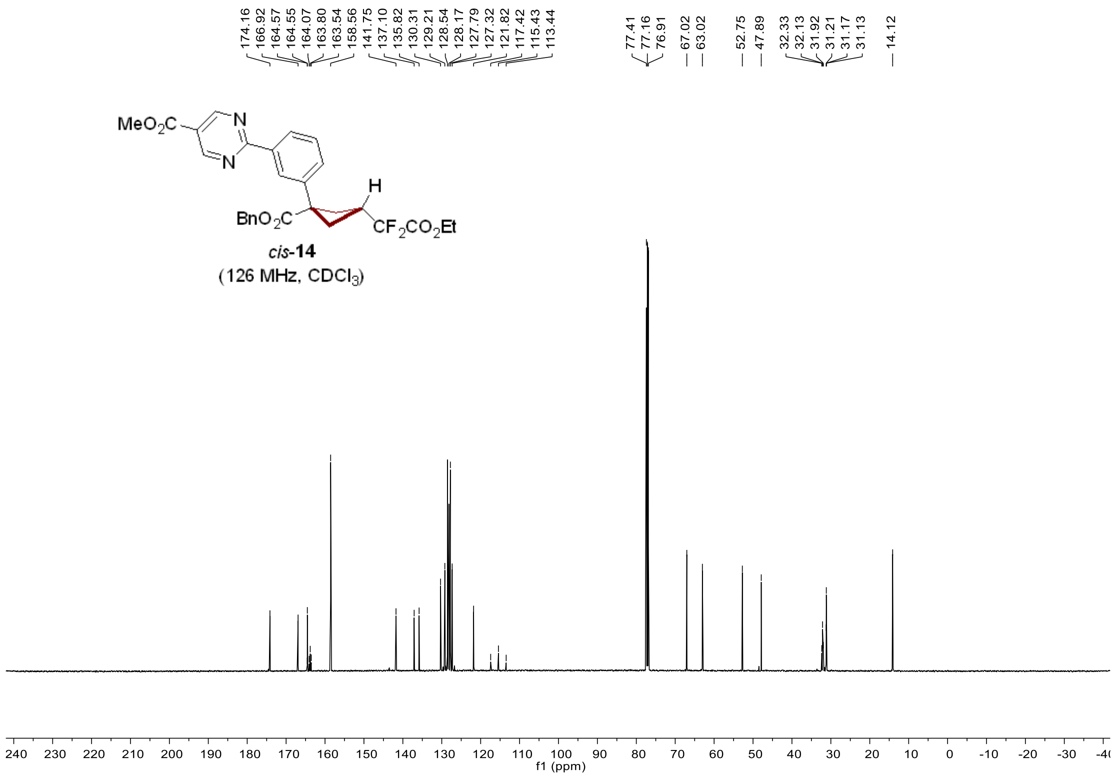

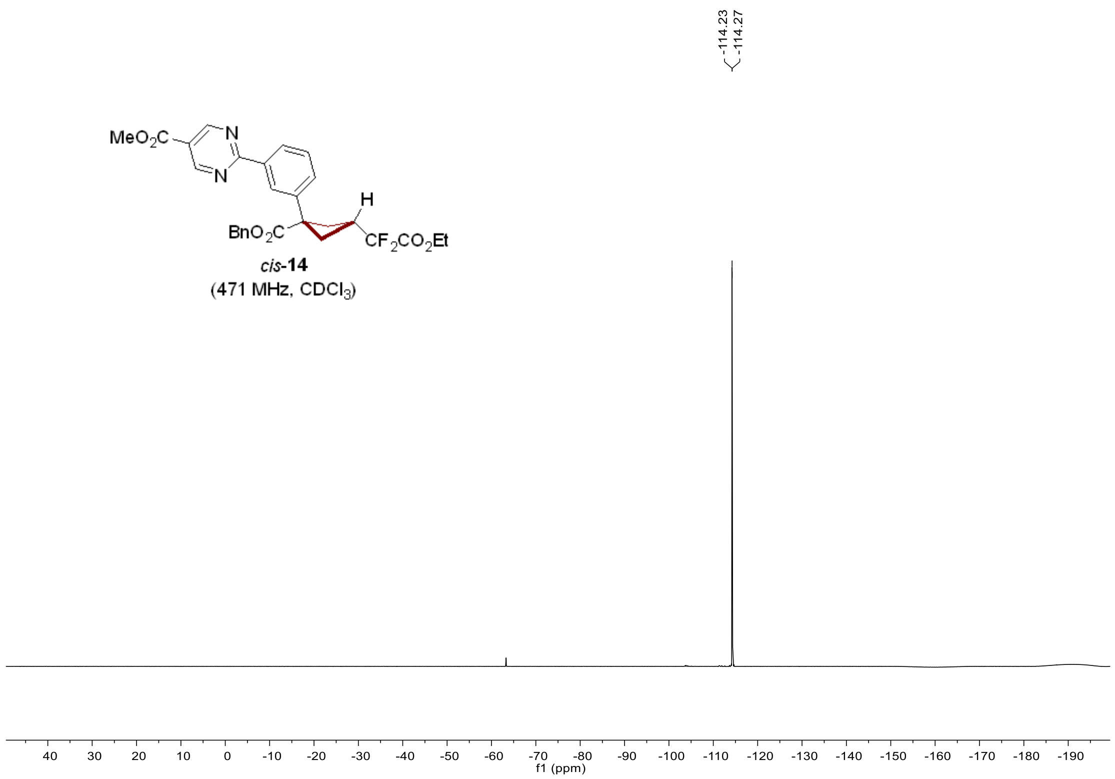


*cis*-**14**
(471 MHz, $CDCl_3$)

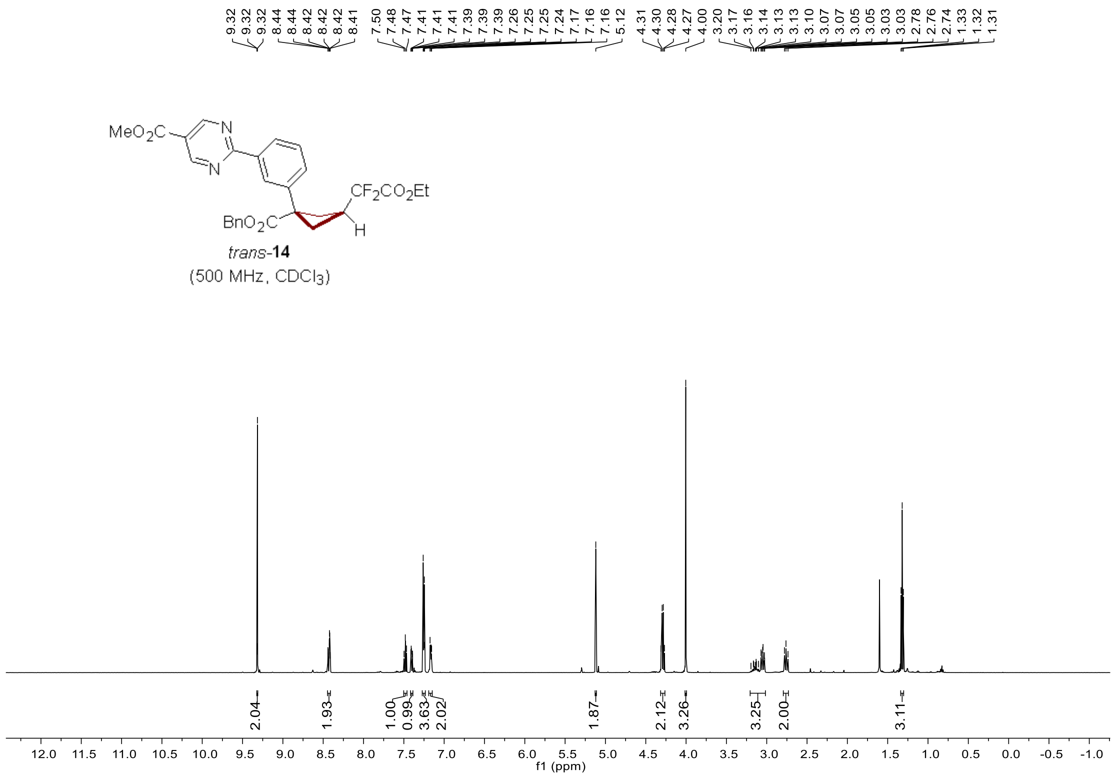


*trans*-**14**
(500 MHz, $CDCl_3$)

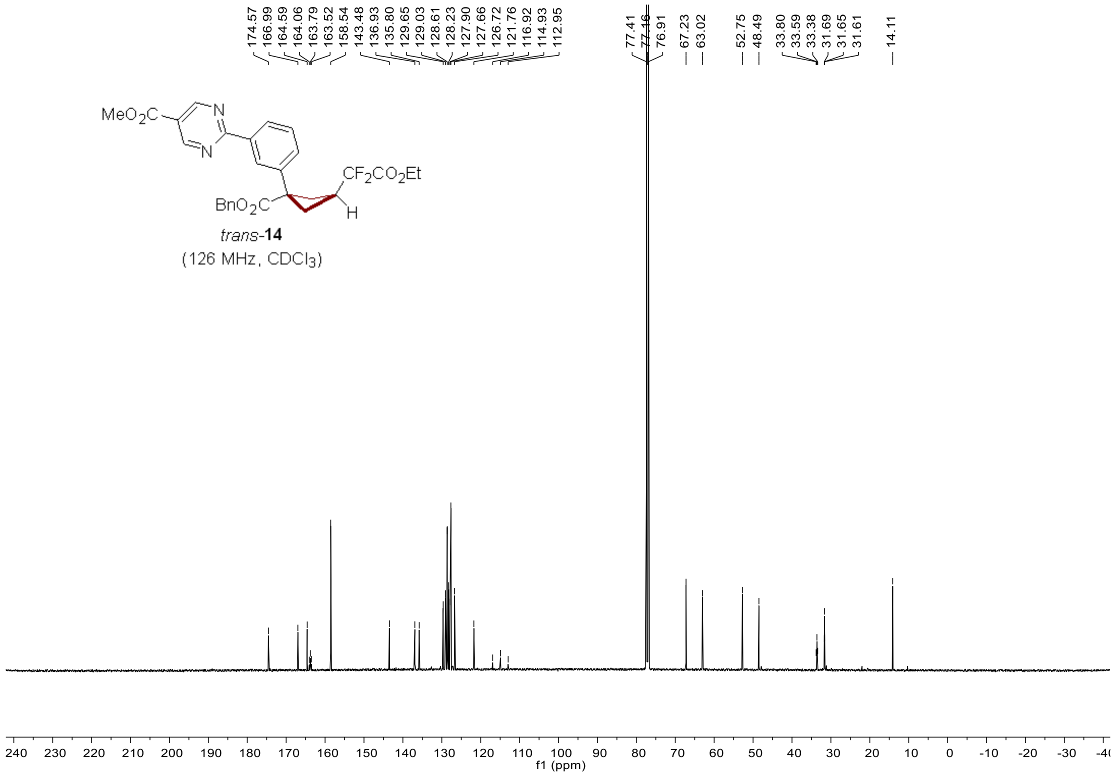
174.57
166.99
164.59
164.06
163.79
163.52
158.54
143.48
136.93
135.80
129.65
129.03
128.61
128.23
127.90
127.66
126.72
121.76
116.92
114.93
112.95
77.41
77.16
76.91
67.23
63.02
52.75
48.49
33.80
33.59
33.38
31.69
31.65
31.61
14.11
$MeO_2C$
N
N
$CF_2CO_2Et$
$BnO_2C$
H
*trans*-**14**
(126 MHz, $CDCl_3$)
f1 (ppm)

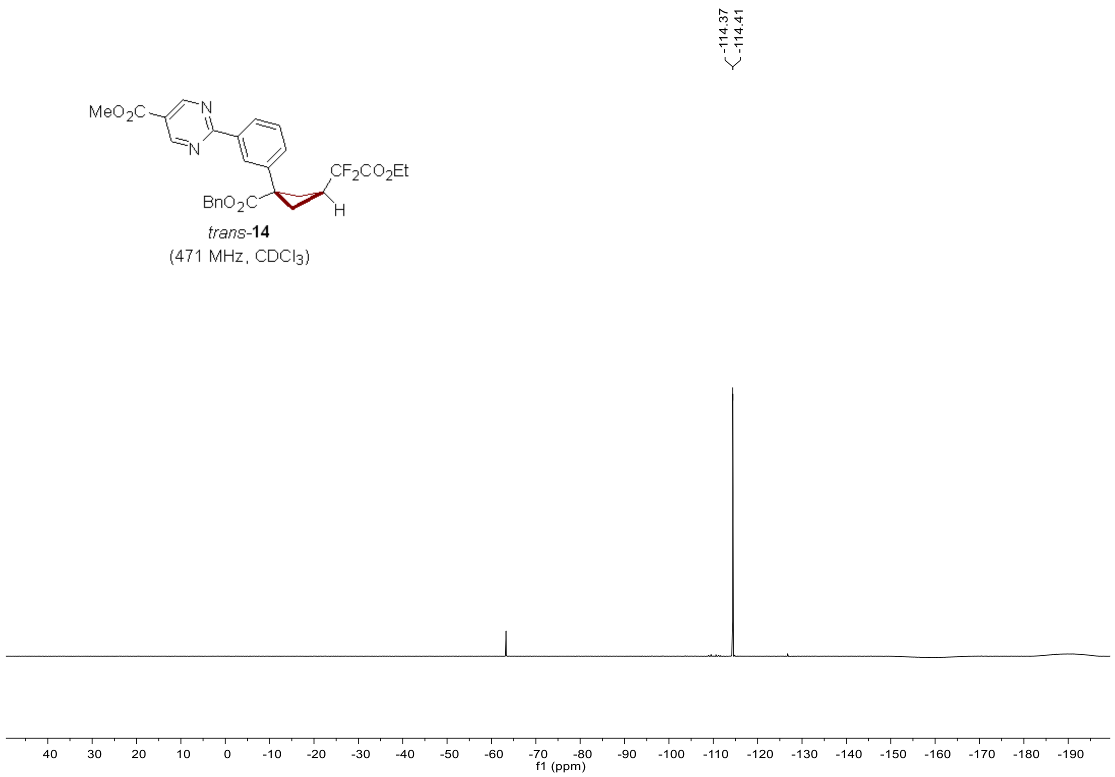
-114.37
-114.41
$MeO_2C$
N
N
$CF_2CO_2Et$
$BnO_2C$
H
*trans*-**14**
(471 MHz, $CDCl_3$)
f1 (ppm)

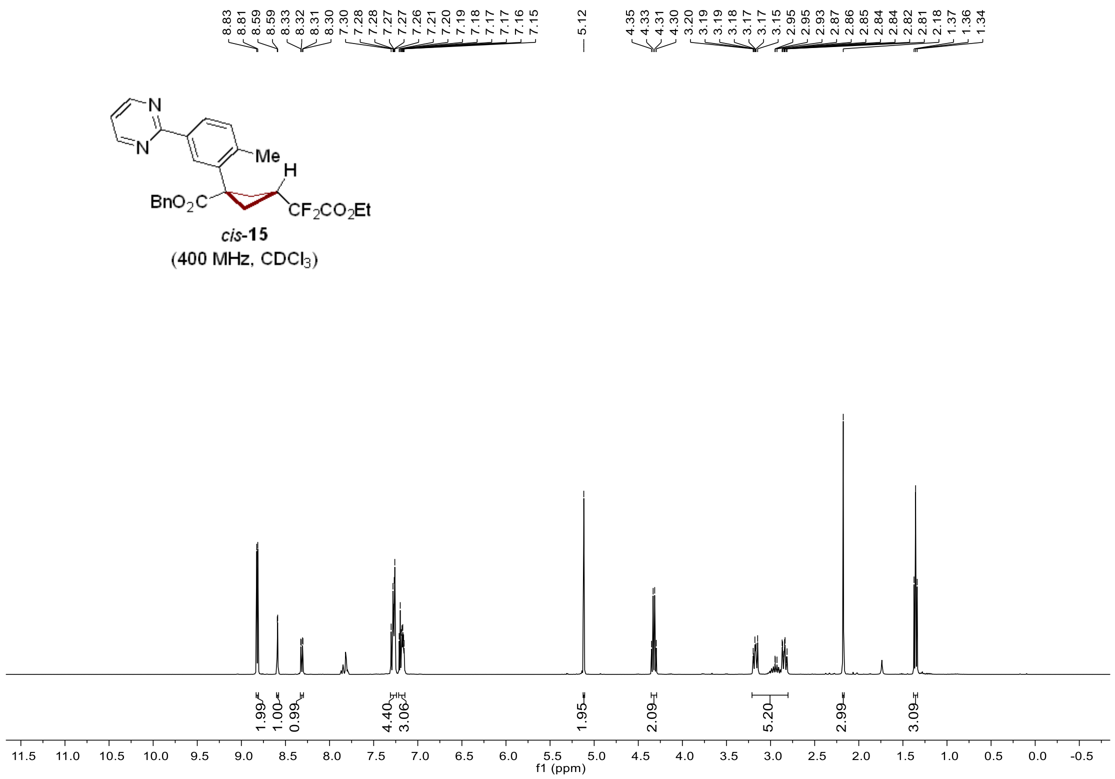
8.83 8.81 8.59 8.59 8.33 8.32 8.31 8.30 7.30 7.28 7.27 7.27 7.26 7.21 7.20 7.19 7.18 7.17 7.17 7.16 7.15
5.12
4.35 4.33 4.31 4.30 3.20 3.19 3.18 3.17 3.17 3.15 2.95 2.95 2.93 2.87 2.86 2.85 2.84 2.84 2.82 2.81 2.18 1.37 1.36 1.34
N
N
Me
H
$BnO_2C$
$CF_2CO_2Et$
*cis*-**15**
(400 MHz, $CDCl_3$)
1.99 1.00 0.99 4.40 3.06 1.95 2.09 5.20 2.99 3.09
11.5 11.0 10.5 10.0 9.5 9.0 8.5 8.0 7.5 7.0 6.5 6.0 5.5 5.0 4.5 4.0 3.5 3.0 2.5 2.0 1.5 1.0 0.5 0.0 -0.5
f1 (ppm)

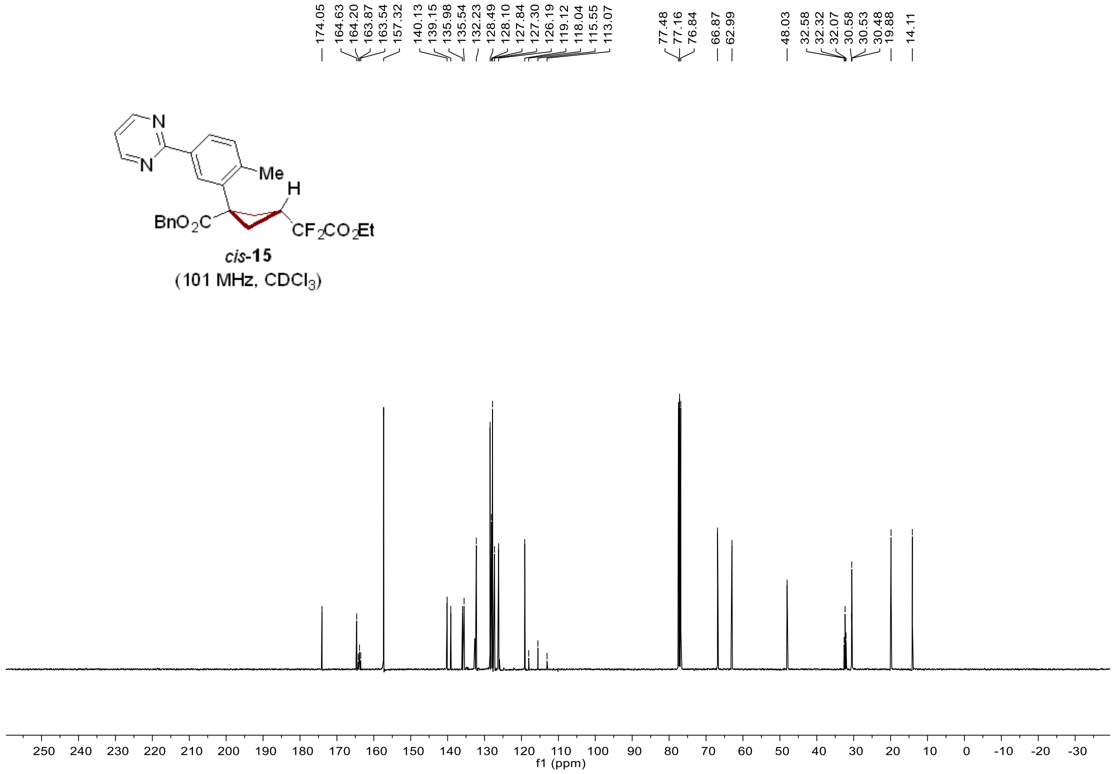
174.05 164.63 164.20 163.87 163.54 157.32 140.13 139.15 135.98 135.54 132.23 128.49 128.10 127.84 127.30 126.19 119.12 118.04 115.55 113.07
77.48 77.16 76.84 66.87 62.99
48.03 32.58 32.32 32.07 30.58 30.53 30.48 19.88 14.11
N
N
Me
H
$BnO_2C$
$CF_2CO_2Et$
*cis*-**15**
(101 MHz, $CDCl_3$)
250 240 230 220 210 200 190 180 170 160 150 140 130 120 110 100 90 80 70 60 50 40 30 20 10 0 -10 -20 -30
f1 (ppm)

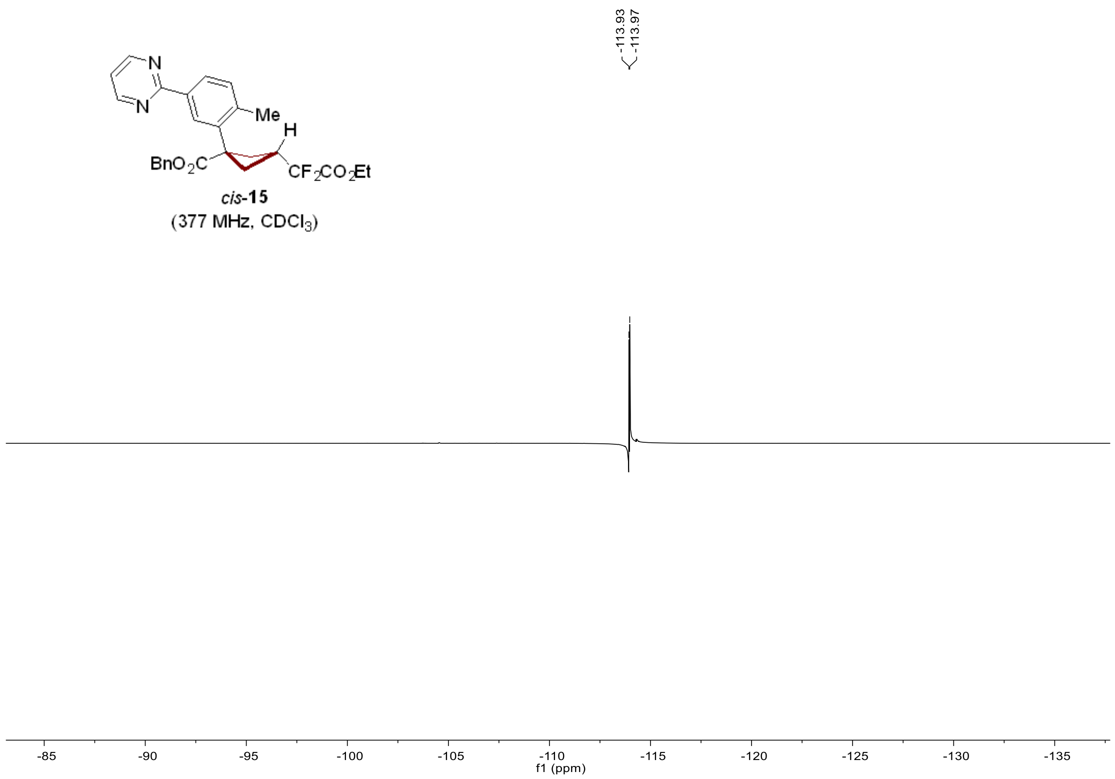


*cis*-**15**
(377 MHz, $CDCl_3$)

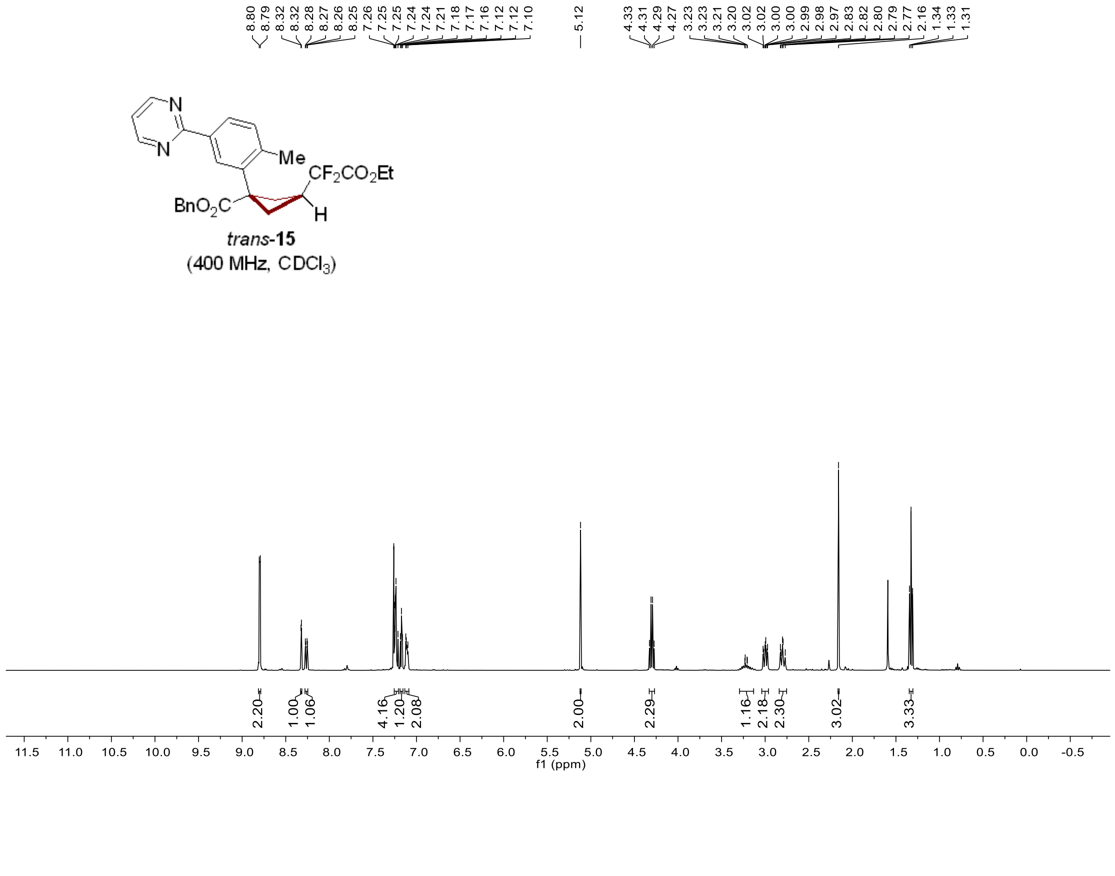


*trans*-**15**
(400 MHz, $CDCl_3$)

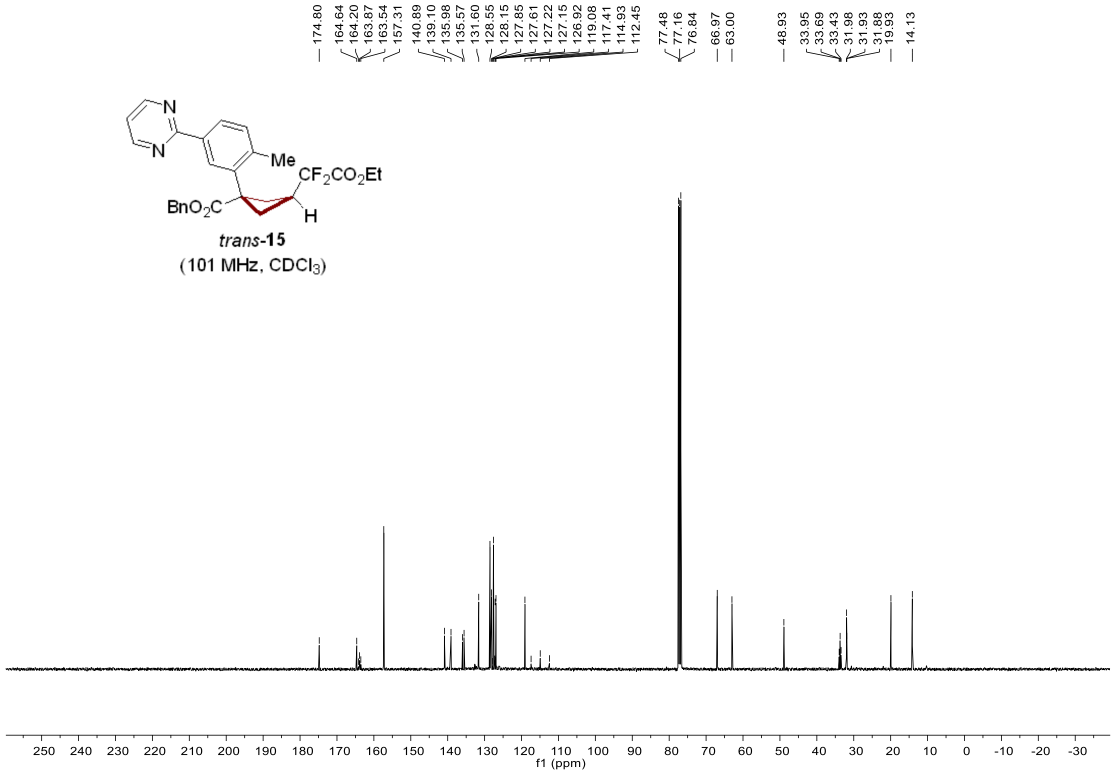
174.80
164.64
164.20
163.87
163.54
157.31
140.89
139.10
135.98
135.57
131.60
128.55
128.15
127.85
127.61
127.22
127.15
126.92
119.08
117.41
114.93
112.45
77.48
77.16
76.84
66.97
63.00
48.93
33.95
33.69
33.43
31.98
31.93
31.88
19.93
14.13
N
N
Me
$CF_2CO_2Et$
$BnO_2C$
H
*trans*-**15**
(101 MHz, $CDCl_3$)
f1 (ppm)

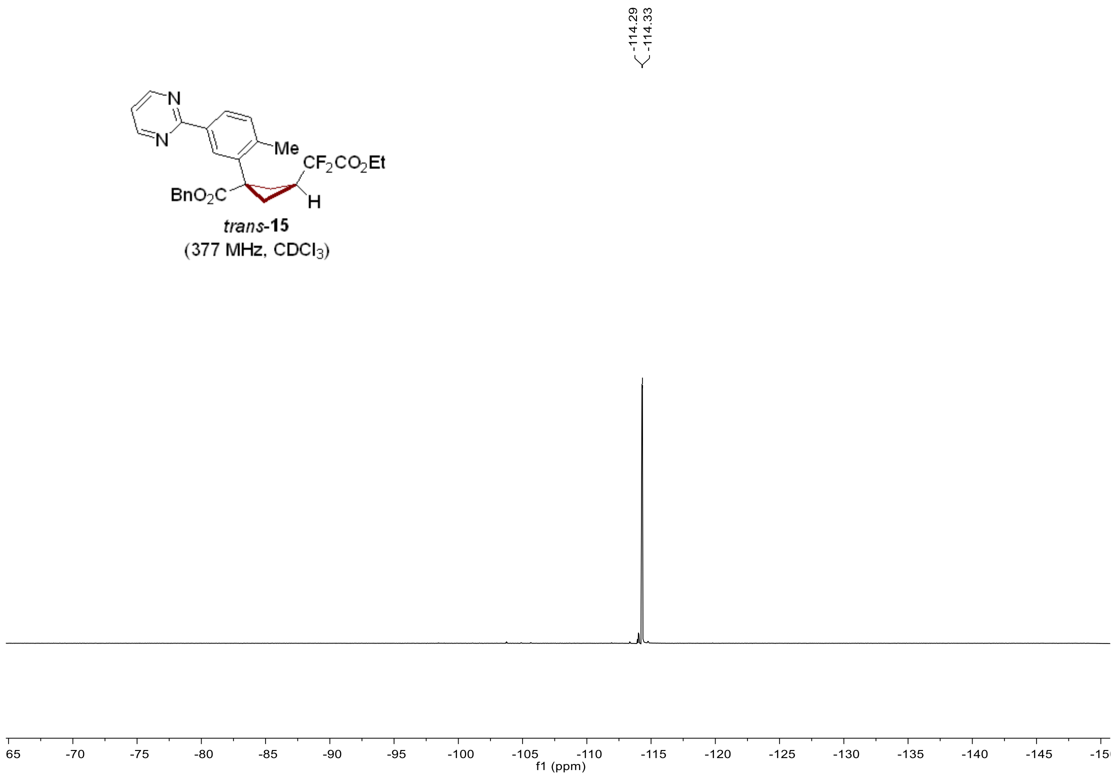
-114.29
-114.33
N
N
Me
$CF_2CO_2Et$
$BnO_2C$
H
*trans*-**15**
(377 MHz, $CDCl_3$)
f1 (ppm)

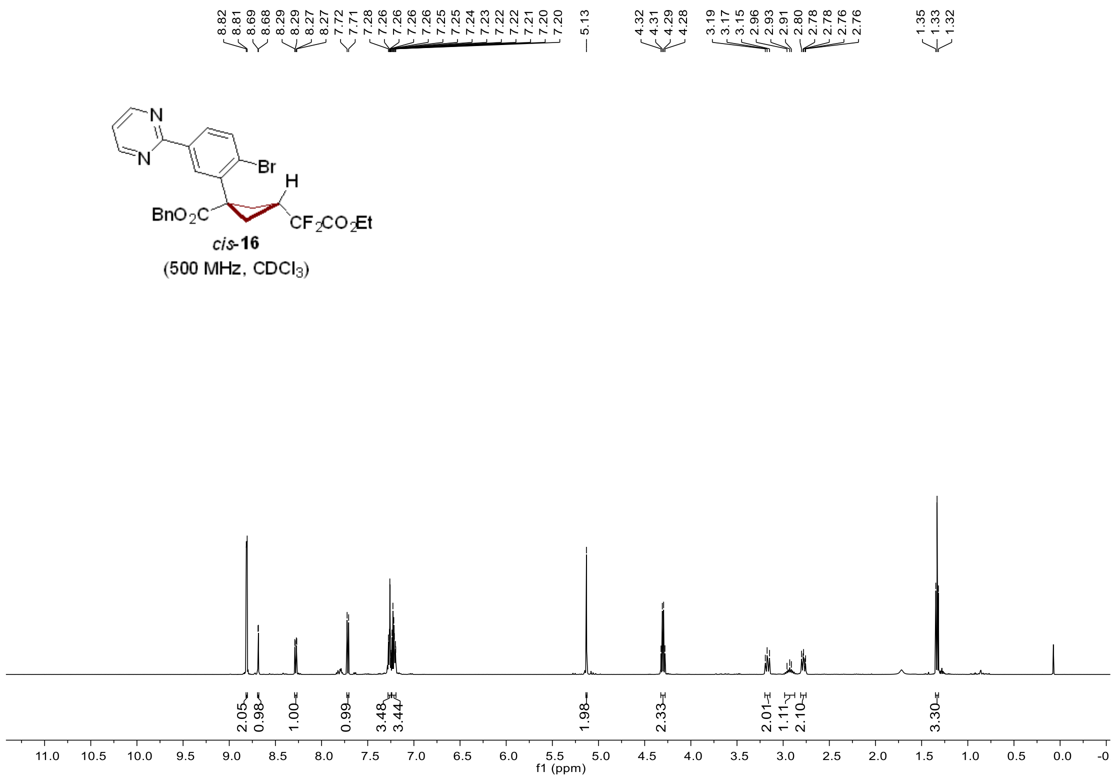

cis-16
(500 MHz, CDCl3)
f1 (ppm)


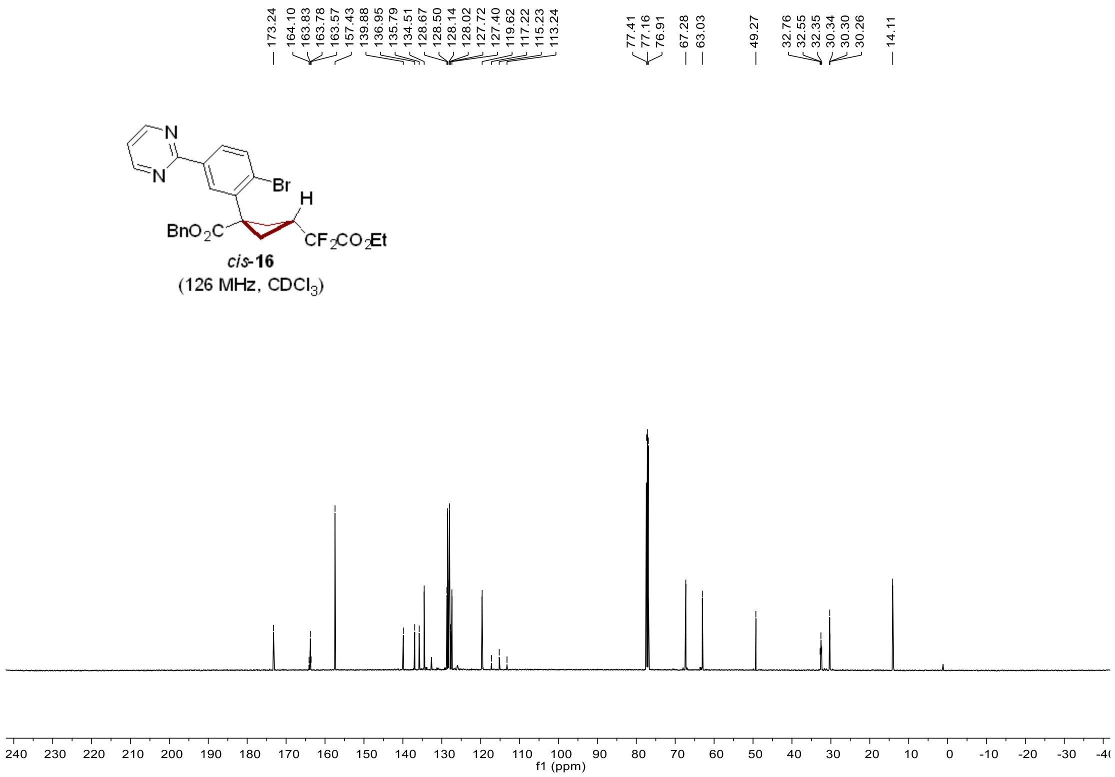

cis-16
(126 MHz, CDCl3)
f1 (ppm)

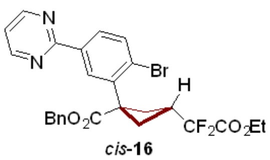

*cis*-**16**
(471 MHz, CDCl$_3$)

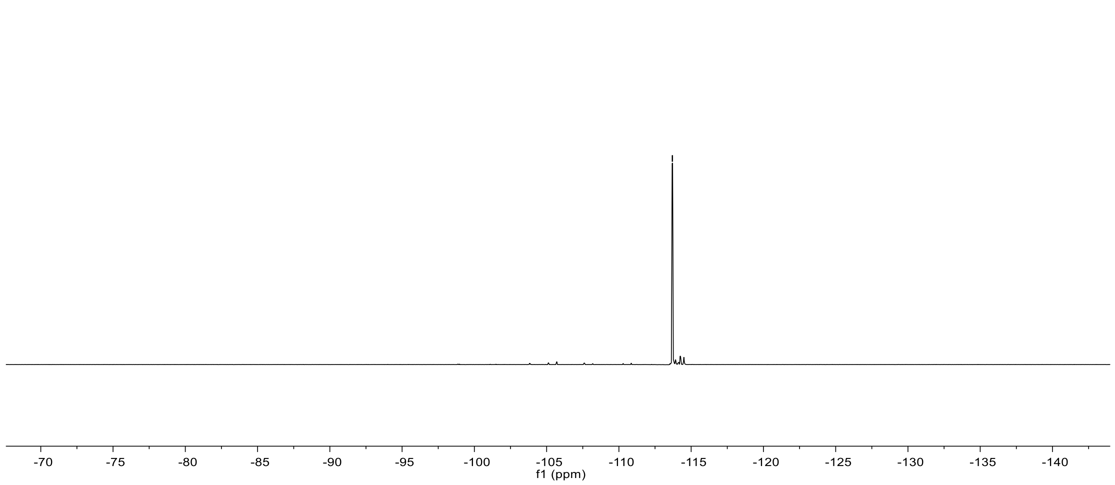

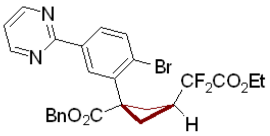

*trans*-**16**
(400 MHz, CDCl$_3$)

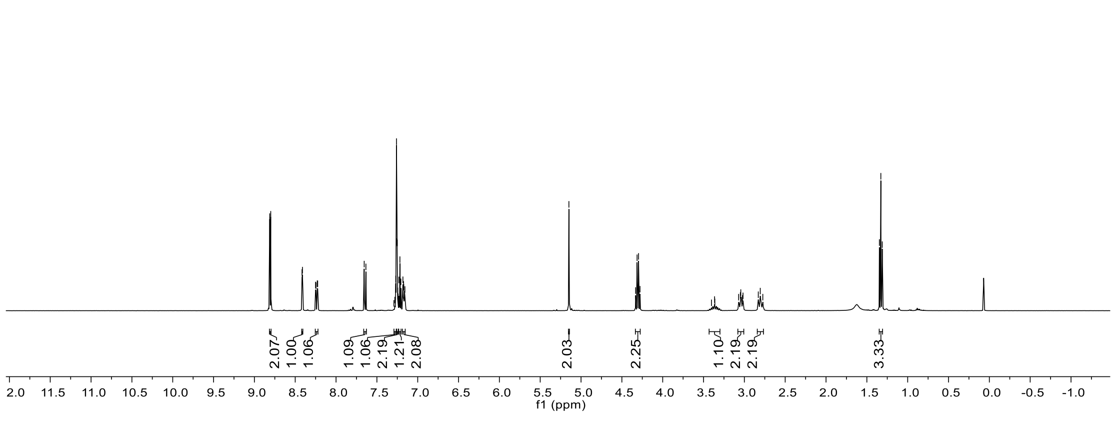

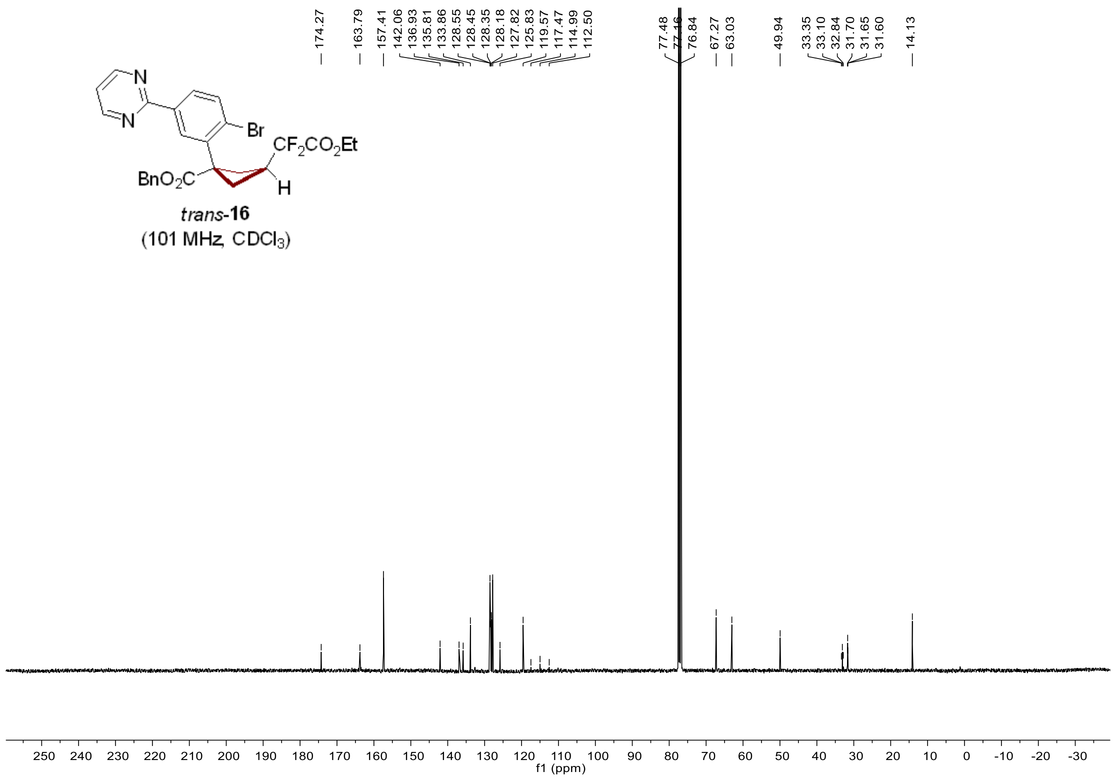
174.27
163.79
157.41
142.06
136.93
135.81
133.86
128.55
128.45
128.35
128.18
127.82
125.83
119.57
117.47
114.99
112.50
77.48
77.16
76.84
67.27
63.03
49.94
33.35
33.10
32.84
31.70
31.65
31.60
14.13
Br
$CF_2CO_2Et$
$BnO_2C$
H
*trans*-**16**
(101 MHz, $CDCl_3$)
f1 (ppm)

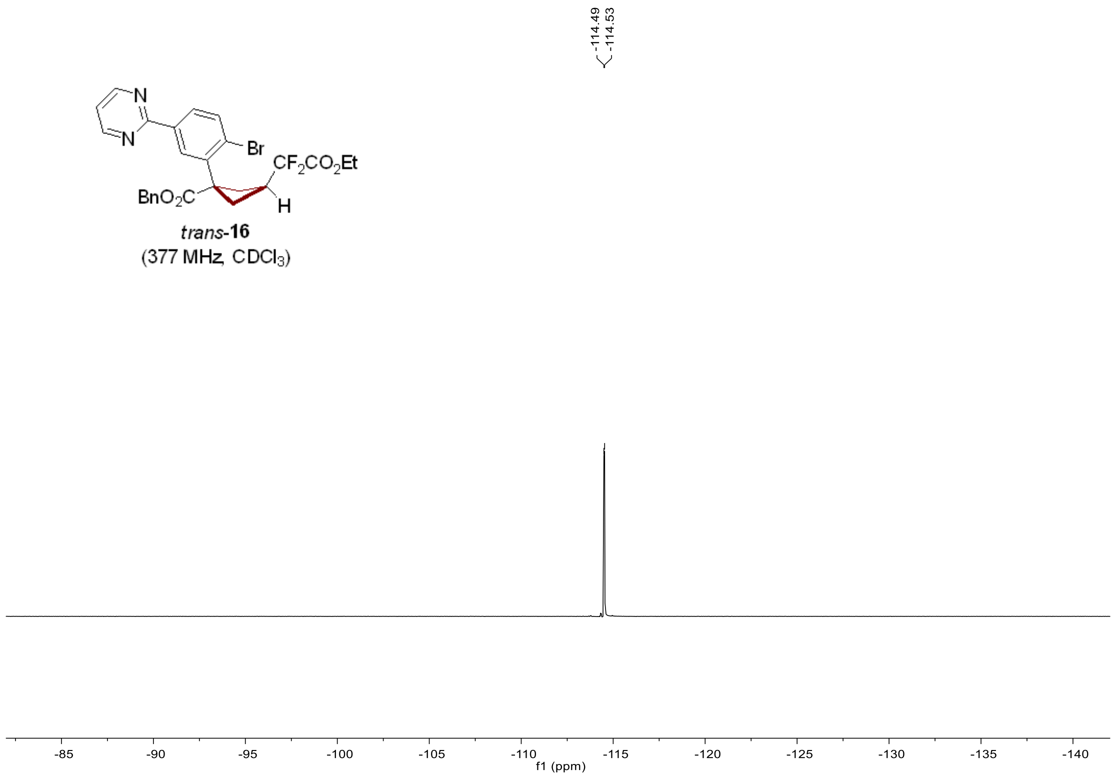
-114.49
-114.53
Br
$CF_2CO_2Et$
$BnO_2C$
H
*trans*-**16**
(377 MHz, $CDCl_3$)
f1 (ppm)

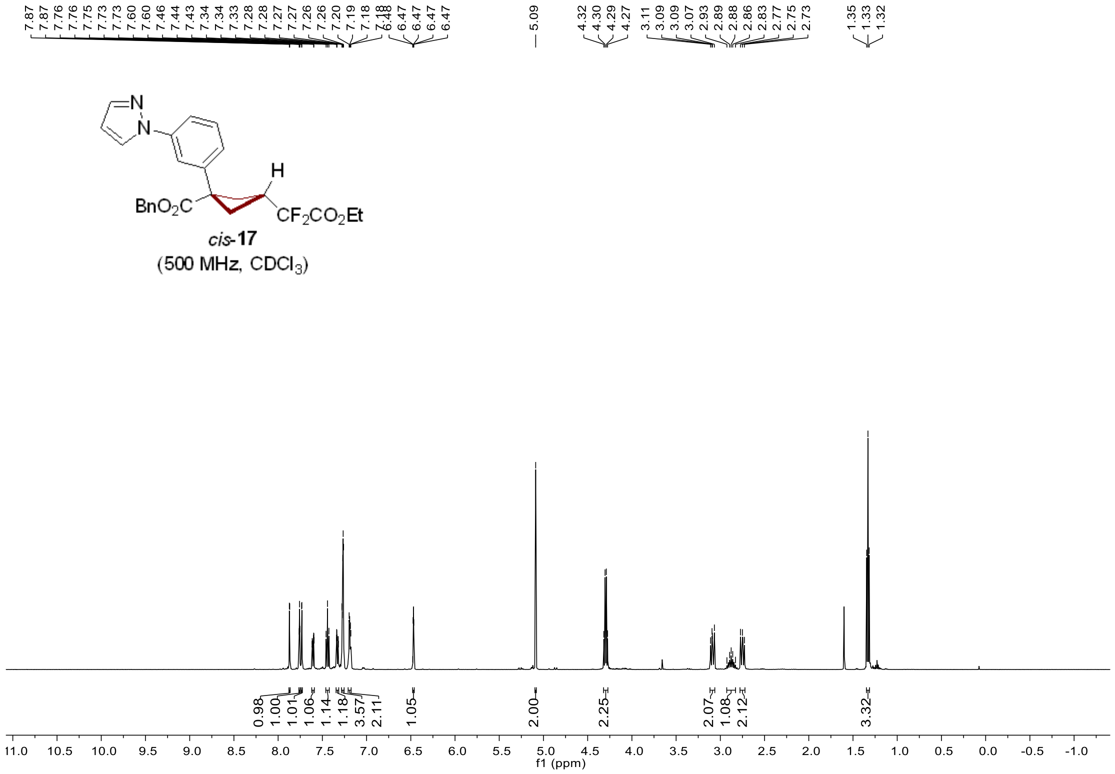

BnO$_2$C
CF$_2$CO$_2$Et
*cis*-**17**
(500 MHz, CDCl$_3$)
f1 (ppm)


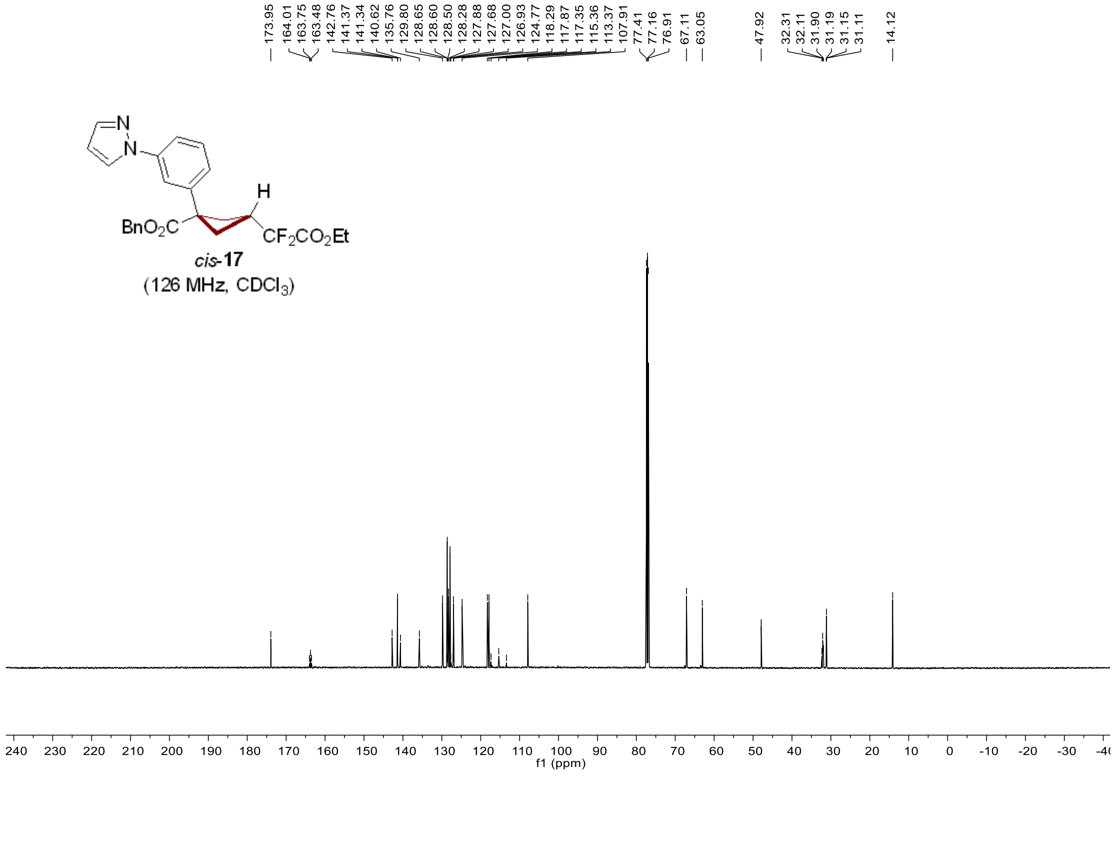

BnO$_2$C
CF$_2$CO$_2$Et
*cis*-**17**
(126 MHz, CDCl$_3$)
f1 (ppm)

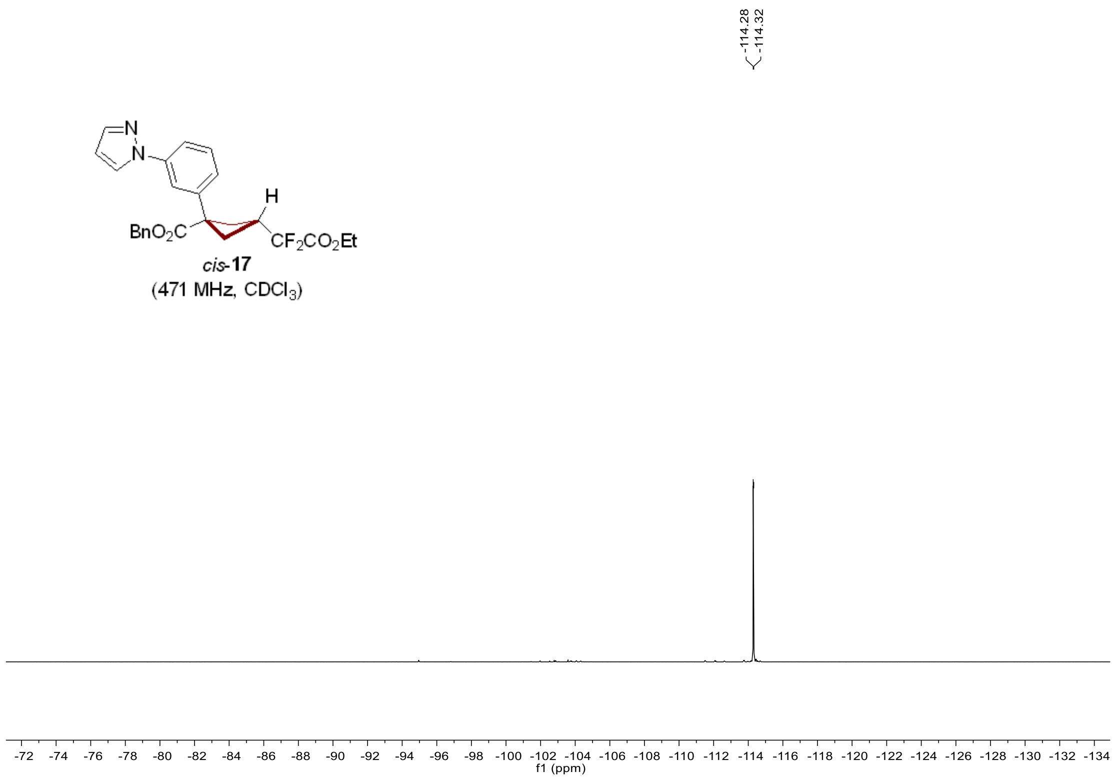


*cis*-**17**
(471 MHz, $CDCl_3$)

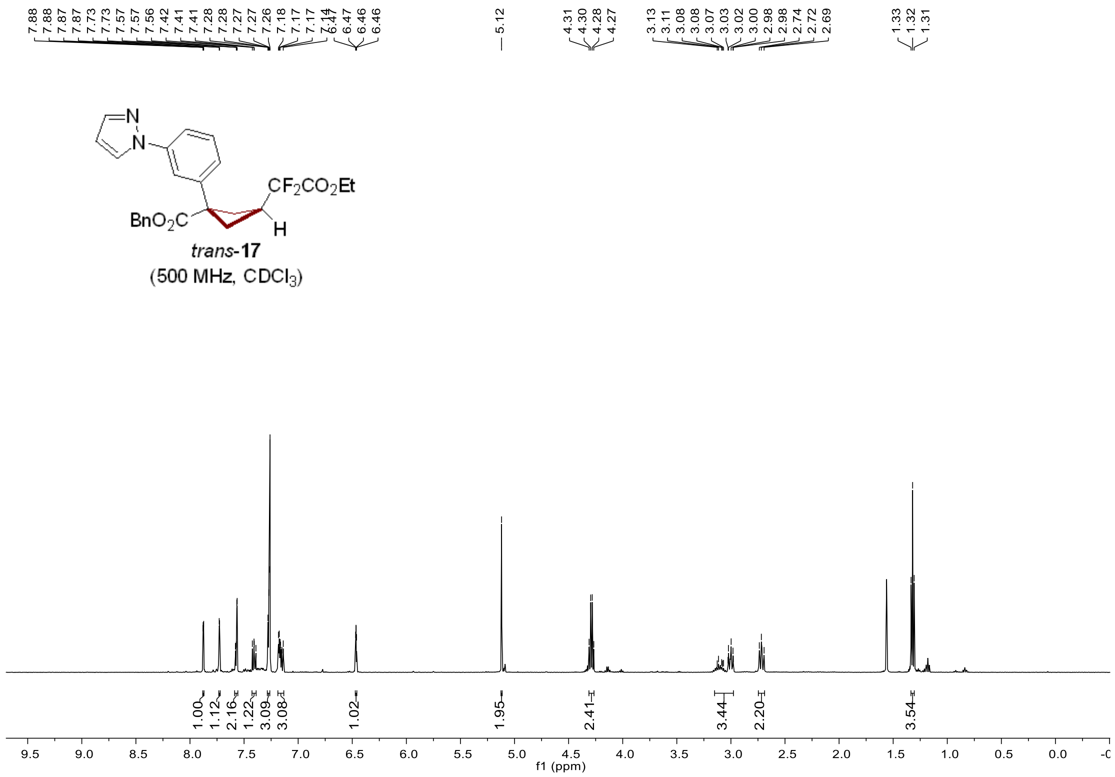


*trans*-**17**
(500 MHz, $CDCl_3$)

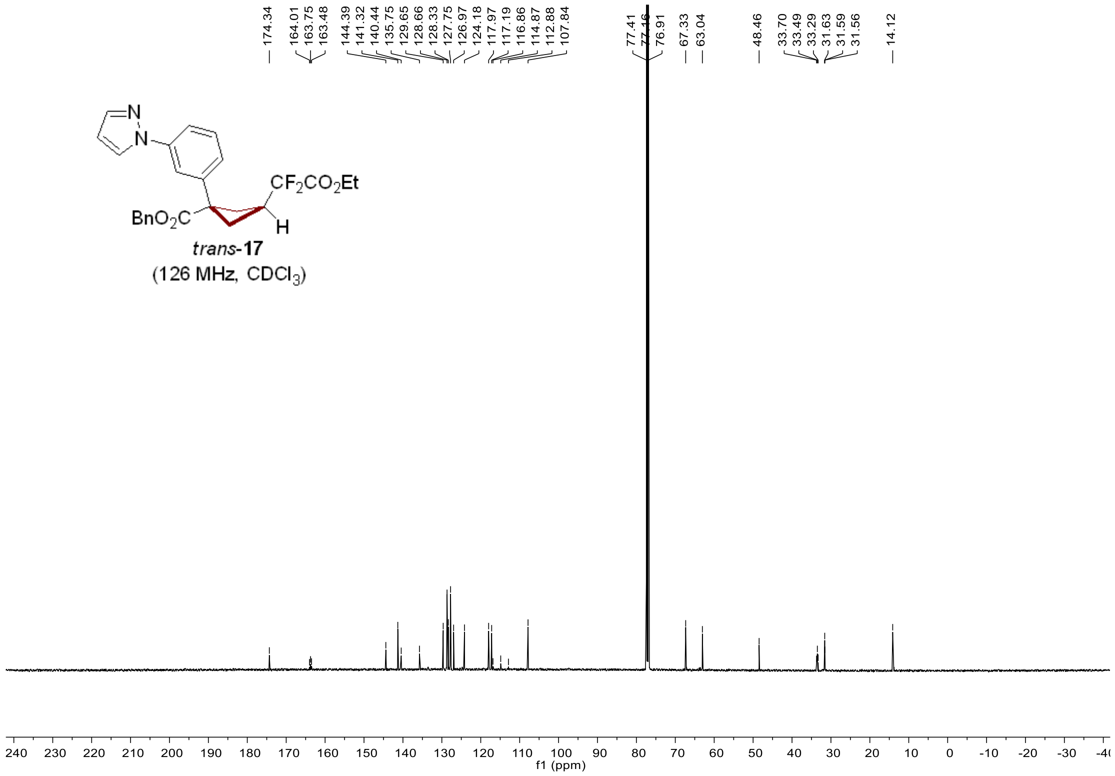
174.34
164.01
163.75
163.48
144.39
141.32
140.44
135.75
129.65
128.66
128.33
127.75
126.97
124.18
117.97
117.19
116.86
114.87
112.88
107.84
77.41
77.16
76.91
67.33
63.04
48.46
33.70
33.49
33.29
31.63
31.59
31.56
14.12
N
N
CF2CO2Et
BnO2C
H
trans-17
(126 MHz, CDCl3)
f1 (ppm)

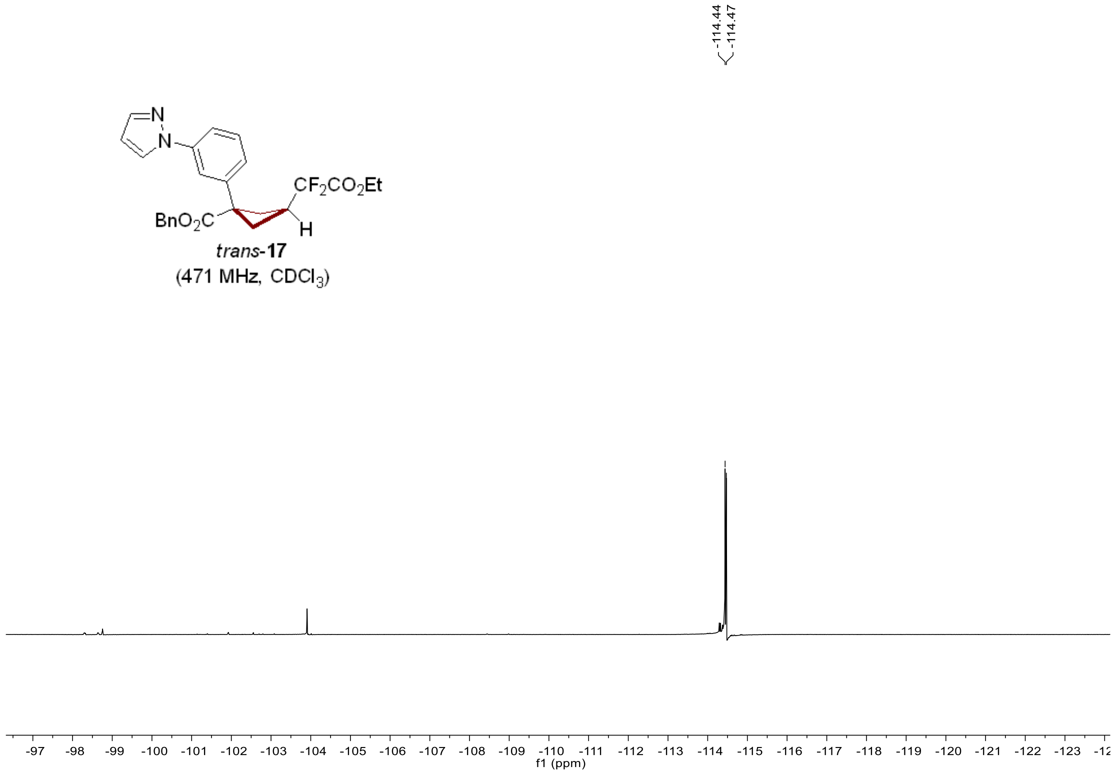
-114.44
-114.47
N
N
CF2CO2Et
BnO2C
H
trans-17
(471 MHz, CDCl3)
f1 (ppm)

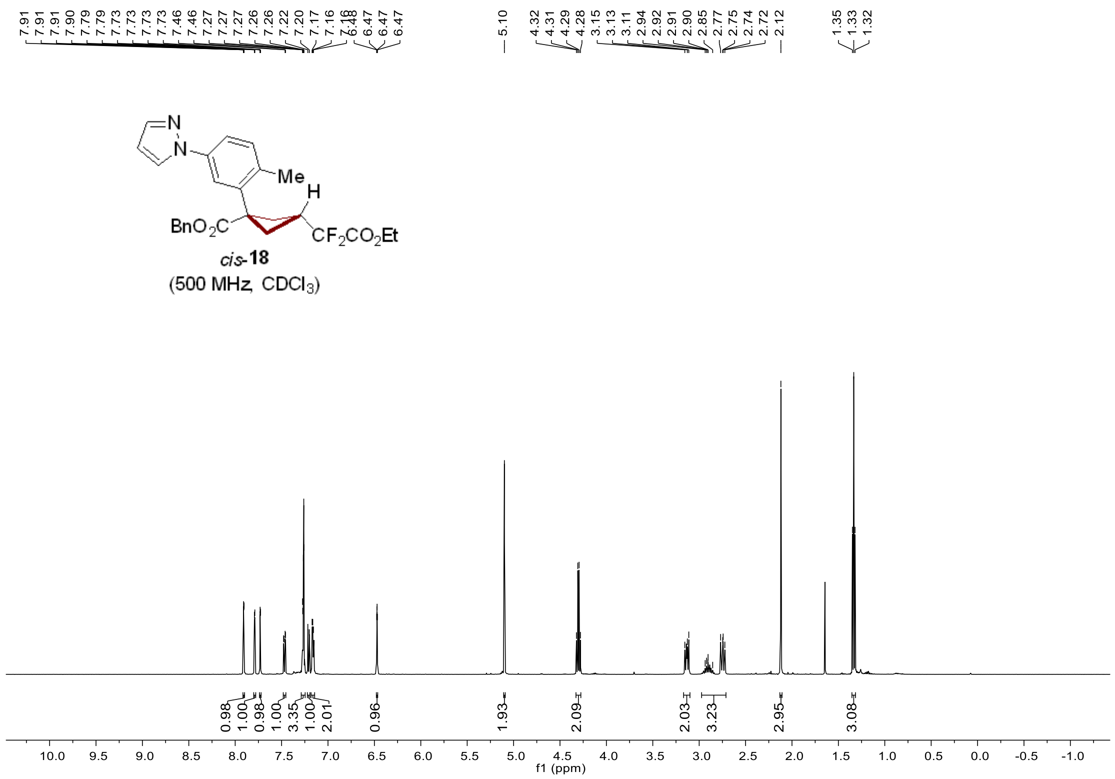
cis-18
(500 MHz, CDCl3)
f1 (ppm)

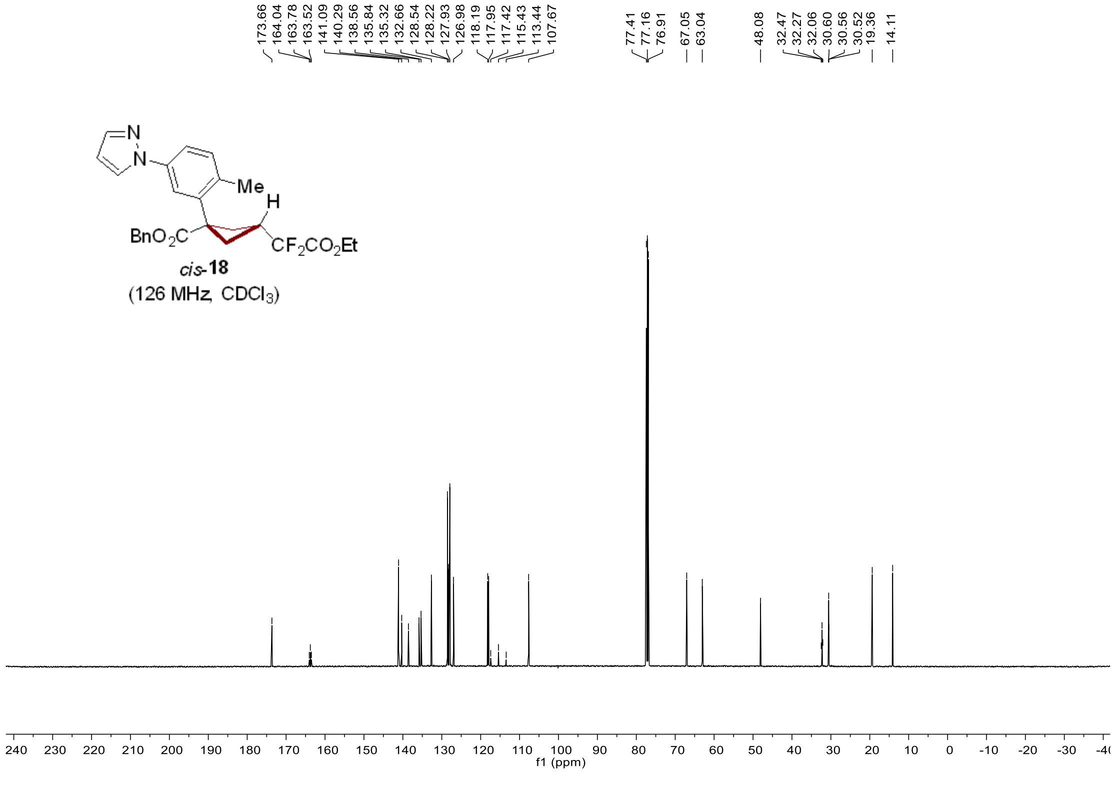
cis-18
(126 MHz, CDCl3)
f1 (ppm)

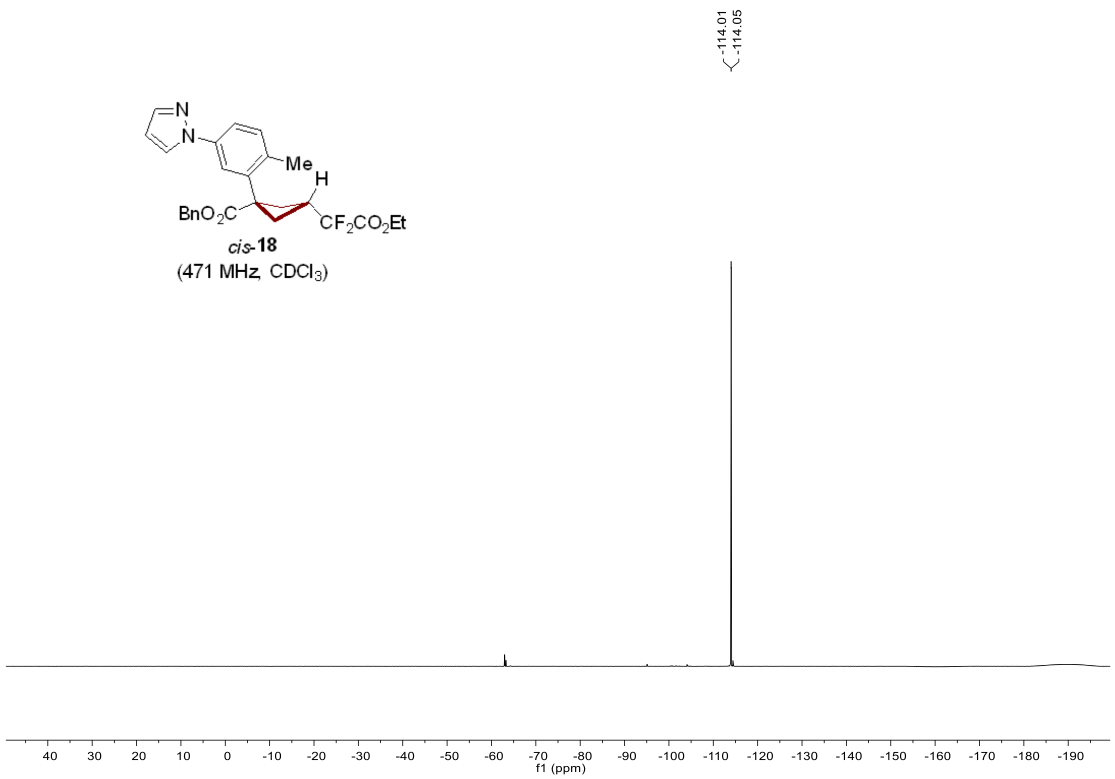
-114.01
-114.05
cis-18
(471 MHz, CDCl3)
f1 (ppm)

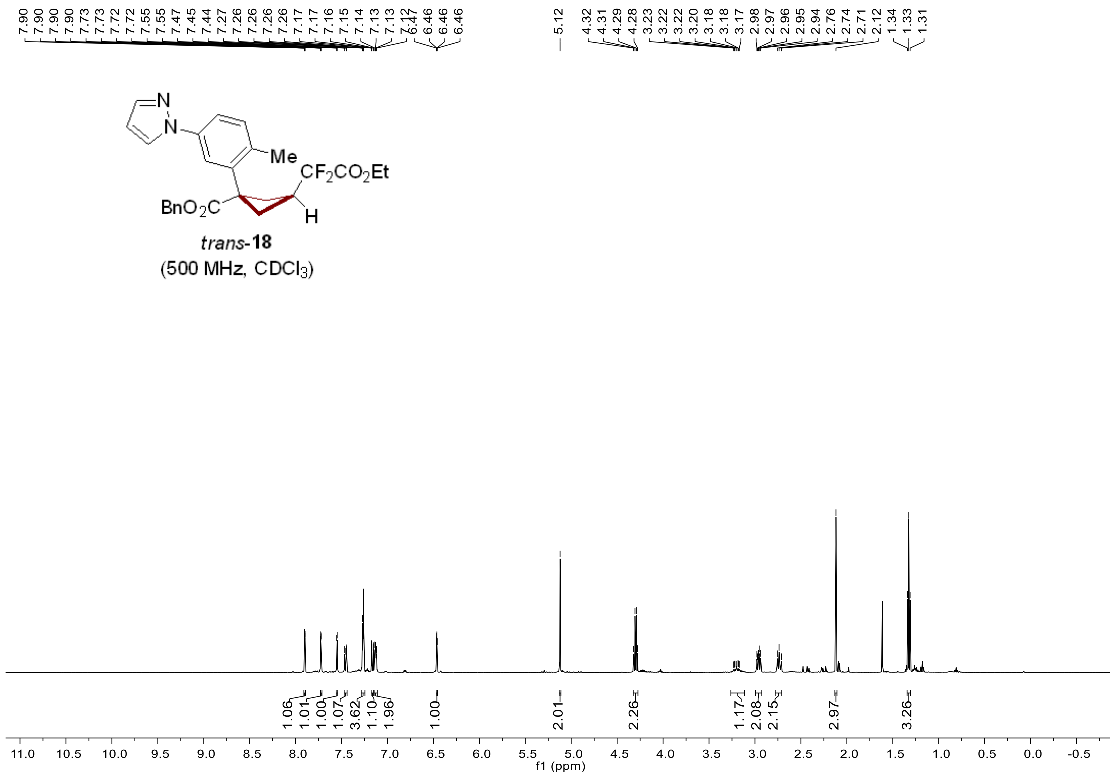
trans-18
(500 MHz, CDCl3)
f1 (ppm)

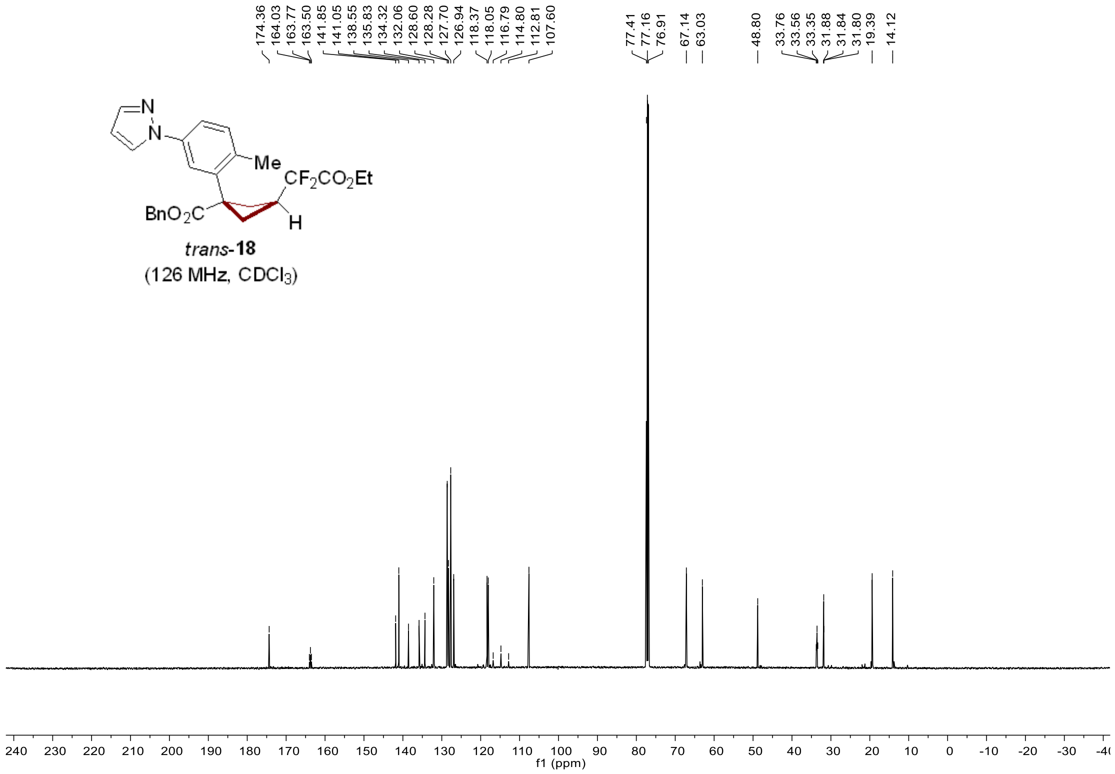
174.36
164.03
163.77
163.50
141.85
141.05
138.55
135.83
134.32
132.06
128.60
128.28
127.70
126.94
118.37
118.05
116.79
114.80
112.81
107.60
77.41
77.16
76.91
67.14
63.03
48.80
33.76
33.56
33.35
31.88
31.84
31.80
19.39
14.12
Me
CF2CO2Et
BnO2C
H
trans-18
(126 MHz, CDCl3)
f1 (ppm)

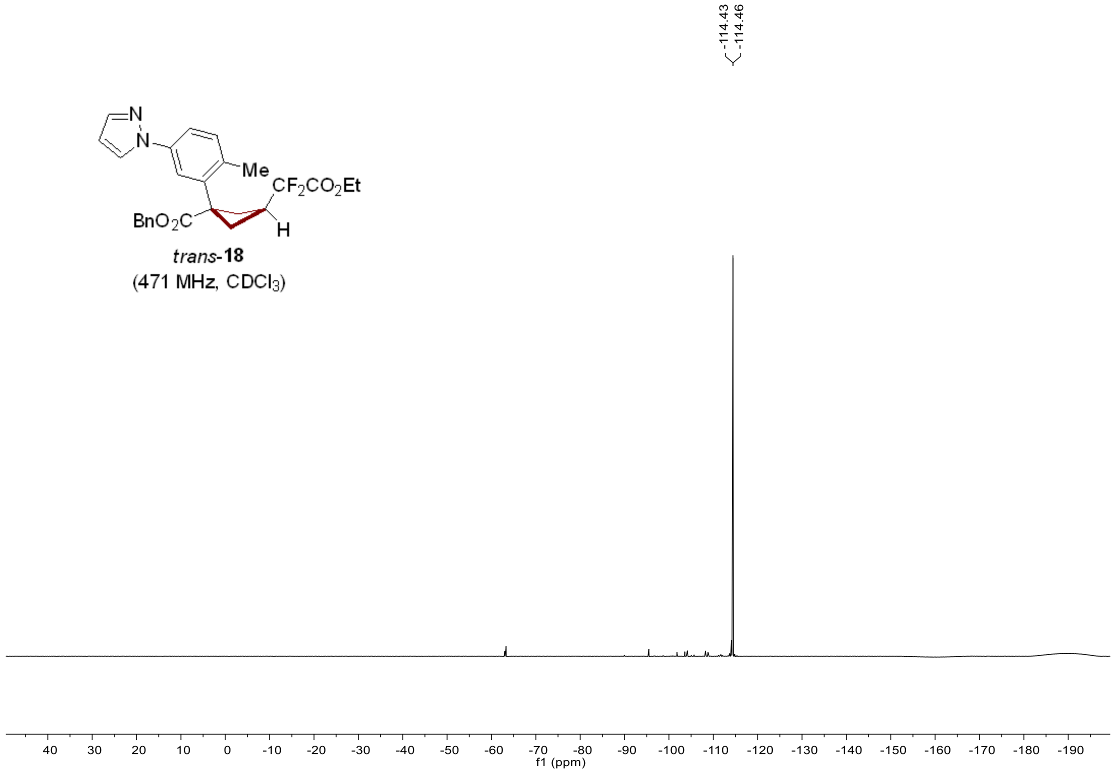
-114.43
-114.46
Me
CF2CO2Et
BnO2C
H
trans-18
(471 MHz, CDCl3)
f1 (ppm)

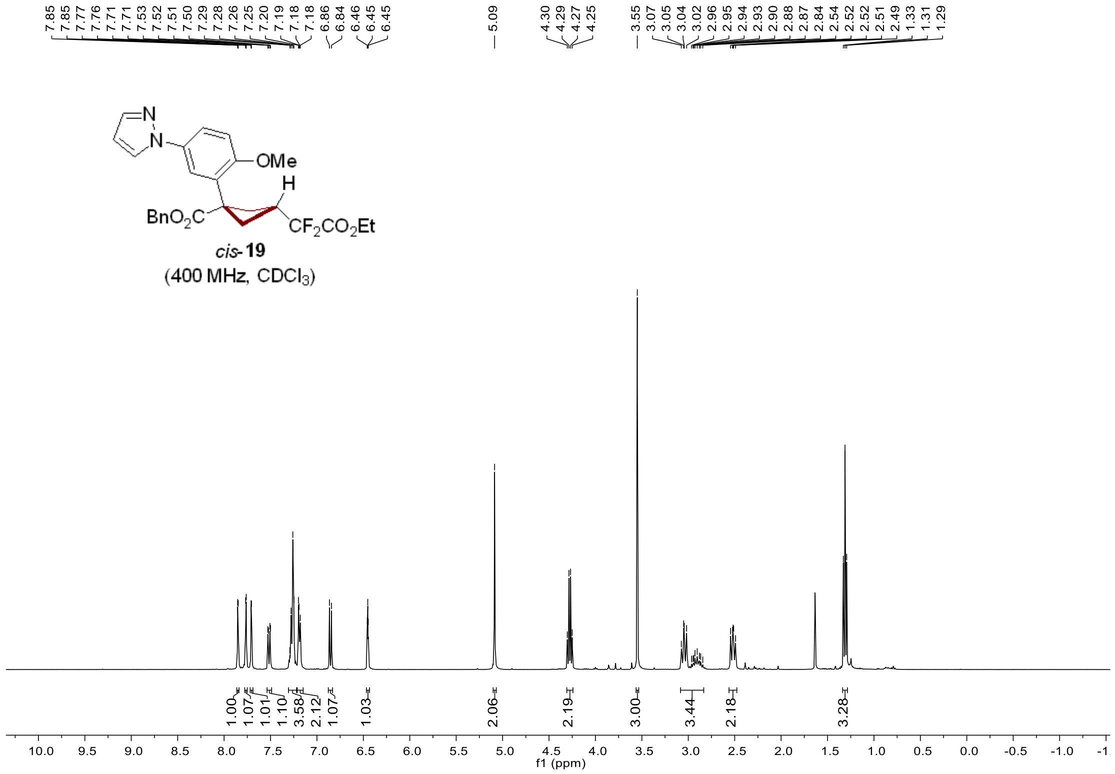
7.85 7.85 7.77 7.76 7.71 7.71 7.53 7.52 7.51 7.50 7.29 7.28 7.26 7.25 7.20 7.19 7.18 6.86 6.84 6.46 6.45 6.45
5.09
4.30 4.29 4.27 4.25
3.55 3.07 3.05 3.04 3.02 2.96 2.95 2.94 2.93 2.90 2.88 2.87 2.84 2.54 2.52 2.52 2.51 2.49 1.33 1.31 1.29
OMe
H
$BnO_2C$
$CF_2CO_2Et$
*cis*-**19**
(400 MHz, $CDCl_3$)
1.00 1.07 1.01 1.10 3.58 2.12 1.07 1.03 2.06 2.19 3.00 3.44 2.18 3.28
f1 (ppm)

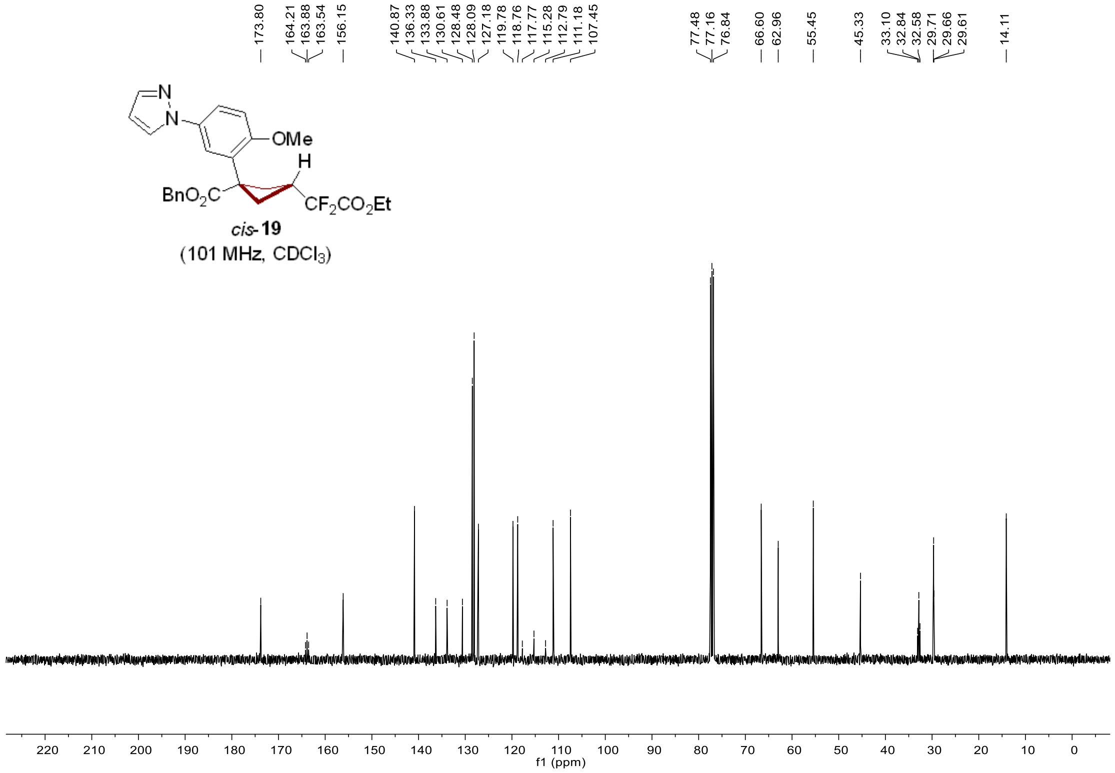
173.80
164.21 163.88 163.54
156.15
140.87 136.33 133.88 130.61 128.48 128.09 127.18 119.78 118.76 117.77 115.28 112.79 111.18 107.45
77.48 77.16 76.84
66.60 62.96
55.45
45.33
33.10 32.84 32.58 29.71 29.66 29.61
14.11
OMe
H
$BnO_2C$
$CF_2CO_2Et$
*cis*-**19**
(101 MHz, $CDCl_3$)
f1 (ppm)

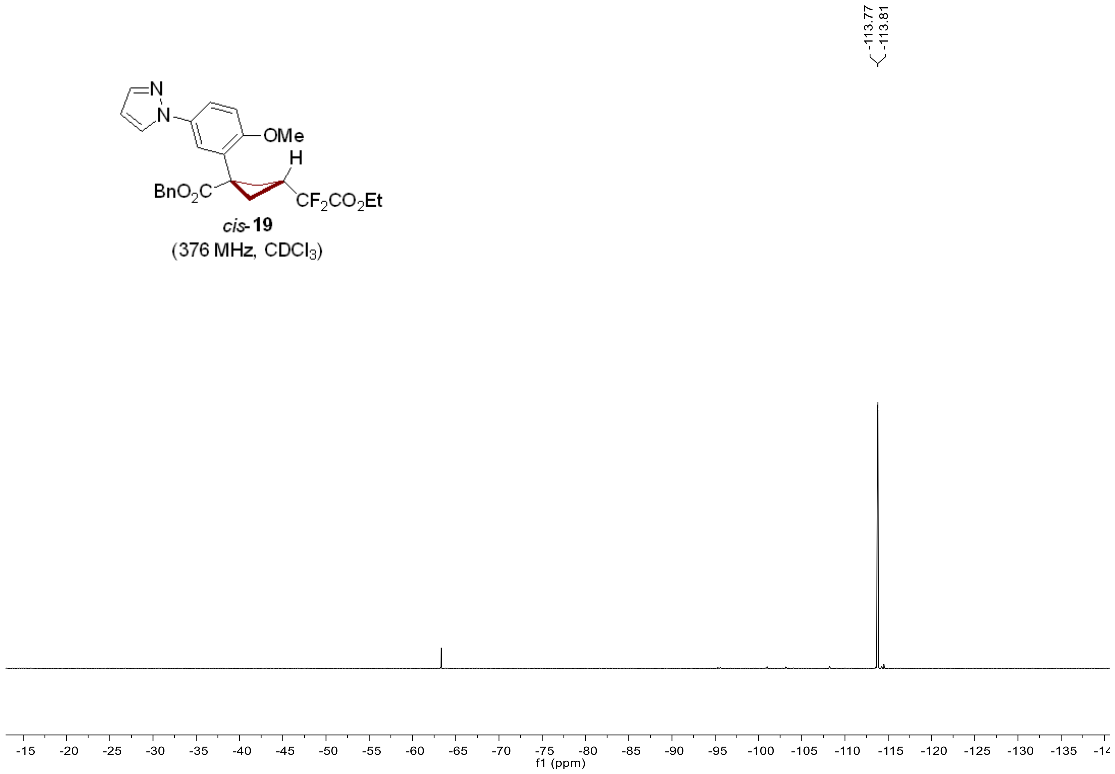

cis-19
(376 MHz, CDCl3)
-113.77
-113.81
f1 (ppm)


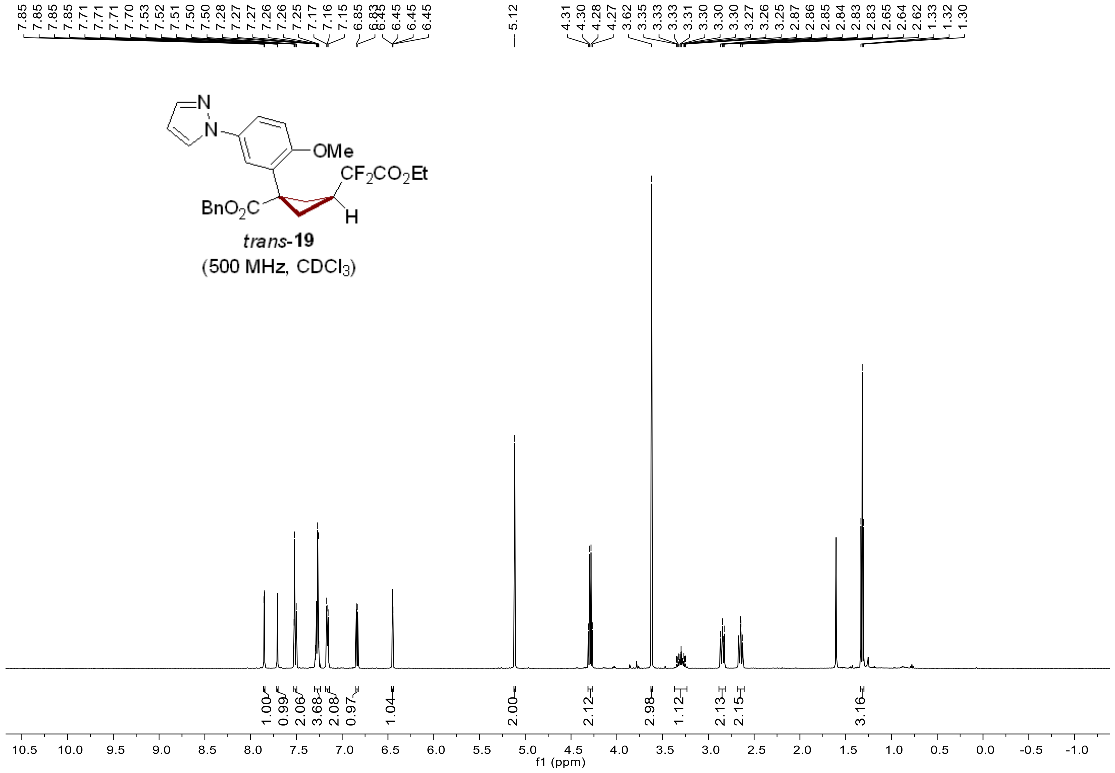

trans-19
(500 MHz, CDCl3)
f1 (ppm)

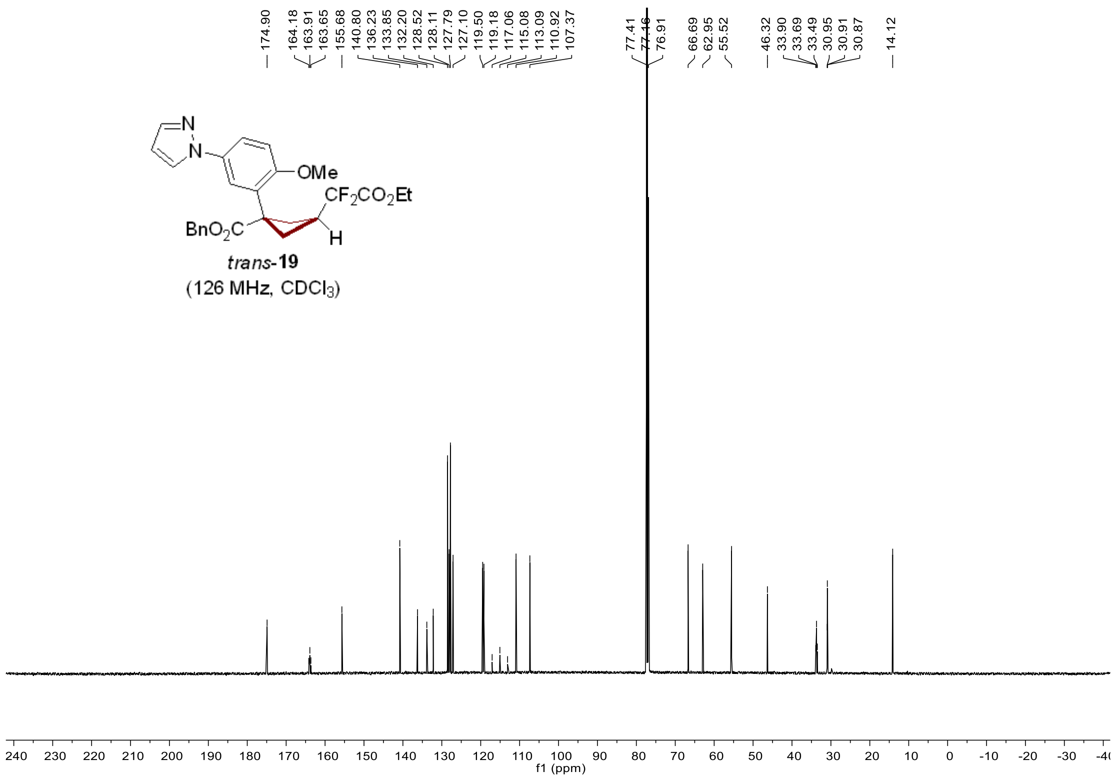
174.90
164.18
163.91
163.65
155.68
140.80
136.23
133.85
132.20
128.52
128.11
127.79
127.10
119.50
119.18
117.06
115.08
113.09
110.92
107.37
77.41
77.16
76.91
66.69
62.95
55.52
46.32
33.90
33.69
33.49
30.95
30.91
30.87
14.12
N
N
OMe
$CF_2CO_2Et$
$BnO_2C$
H
*trans*-**19**
(126 MHz, $CDCl_3$)
240 230 220 210 200 190 180 170 160 150 140 130 120 110 100 90 80 70 60 50 40 30 20 10 0 -10 -20 -30
f1 (ppm)

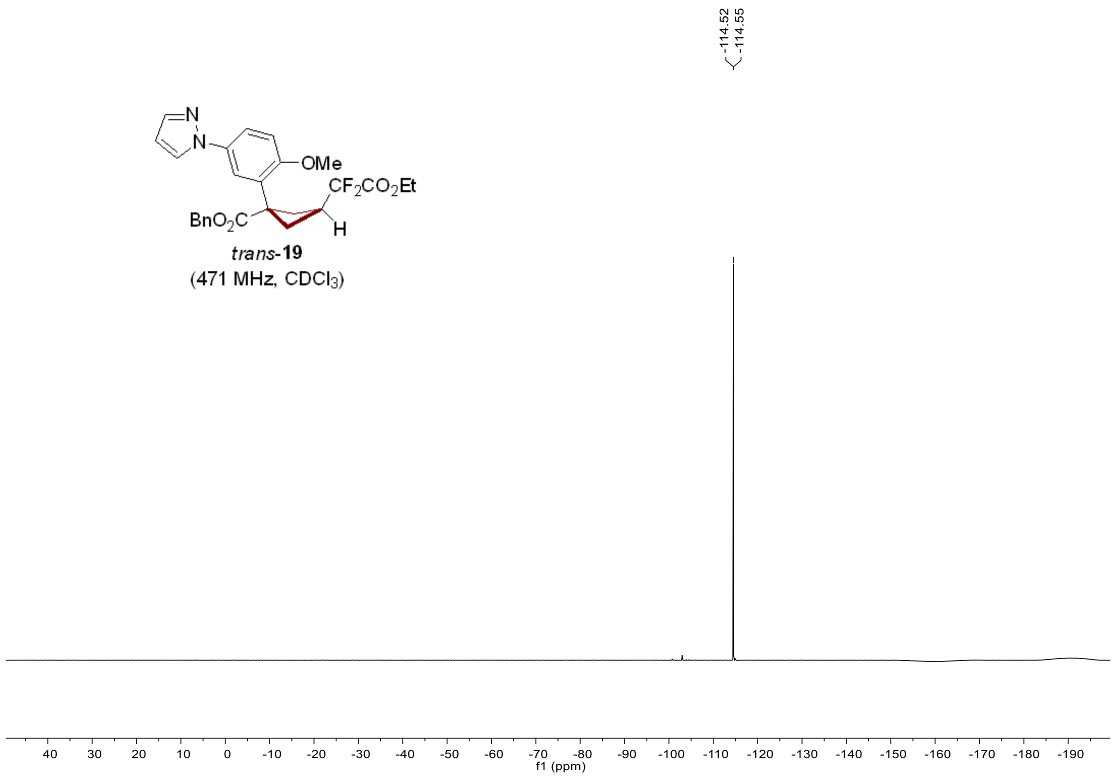
-114.52
-114.55
N
N
OMe
$CF_2CO_2Et$
$BnO_2C$
H
*trans*-**19**
(471 MHz, $CDCl_3$)
40 30 20 10 0 -10 -20 -30 -40 -50 -60 -70 -80 -90 -100 -110 -120 -130 -140 -150 -160 -170 -180 -190
f1 (ppm)

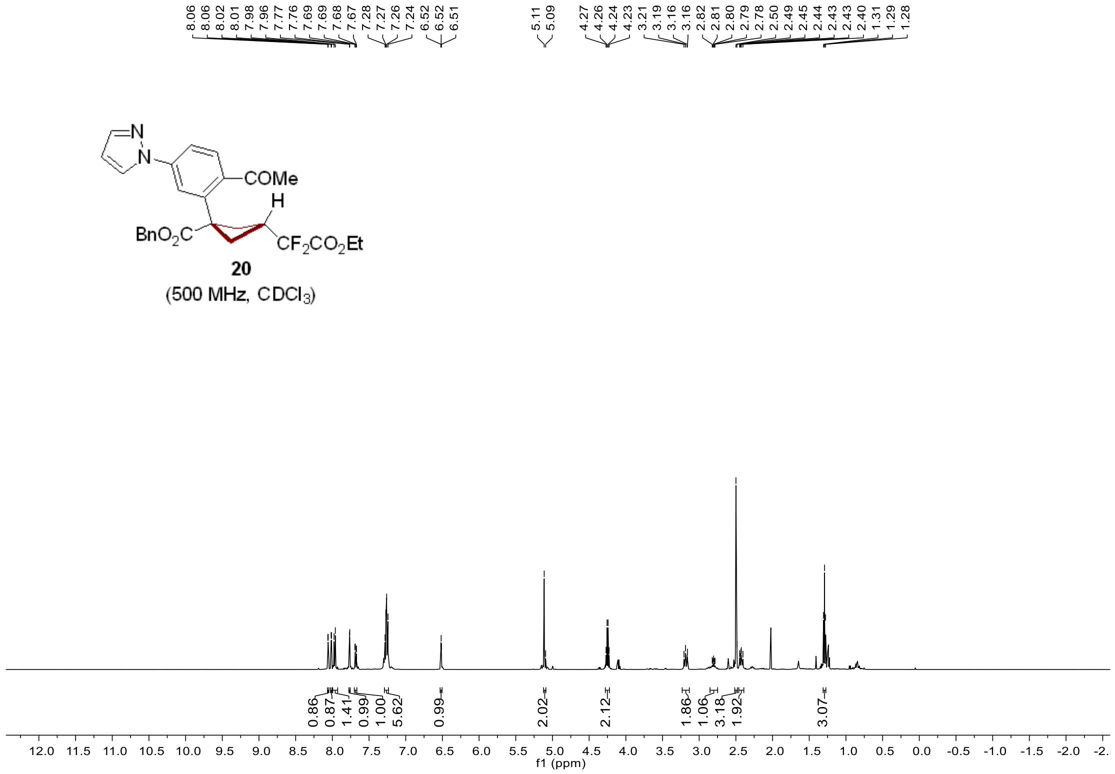

N
N
COMe
H
BnO2C
CF2CO2Et
20
(500 MHz, CDCl3)


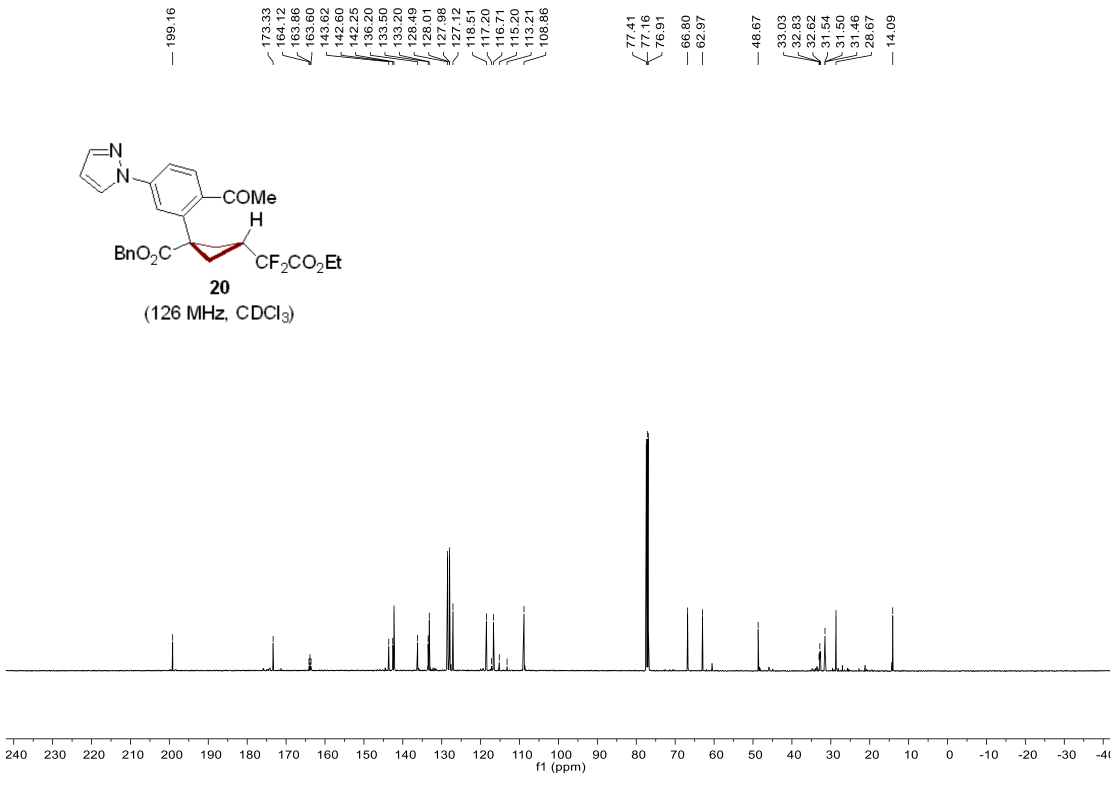

N
N
COMe
H
BnO2C
CF2CO2Et
20
(126 MHz, CDCl3)

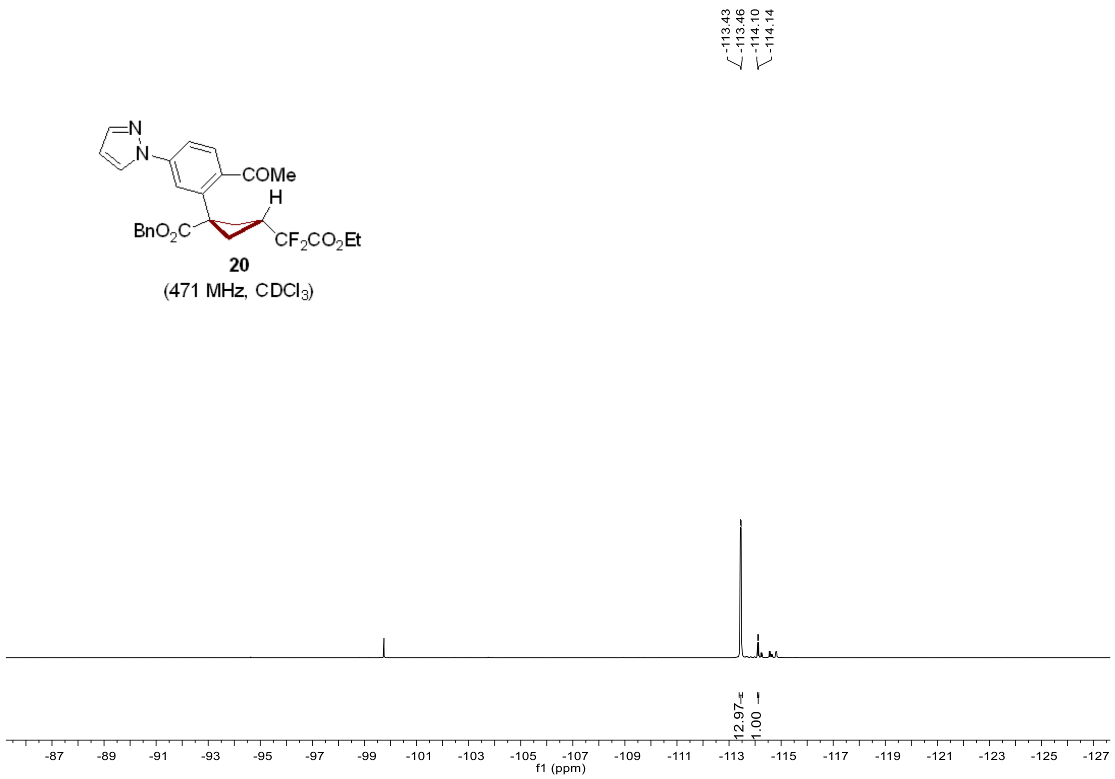


**20**
(471 MHz, $CDCl_3$)

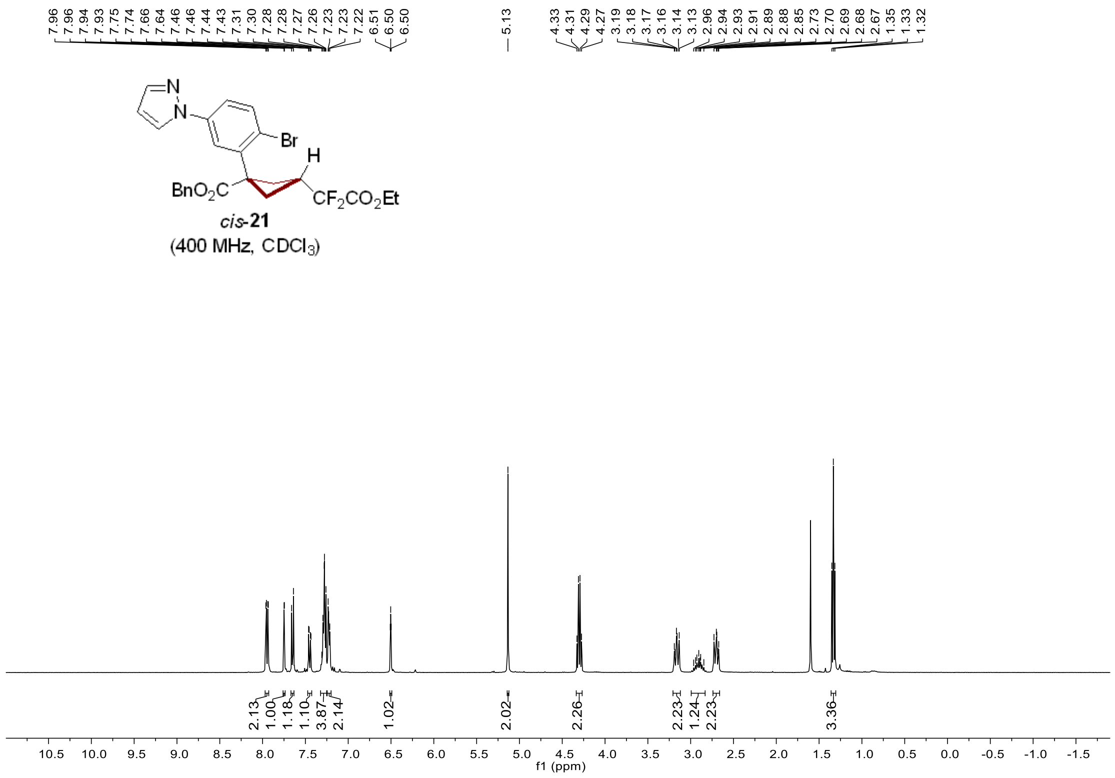


*cis*-**21**
(400 MHz, $CDCl_3$)

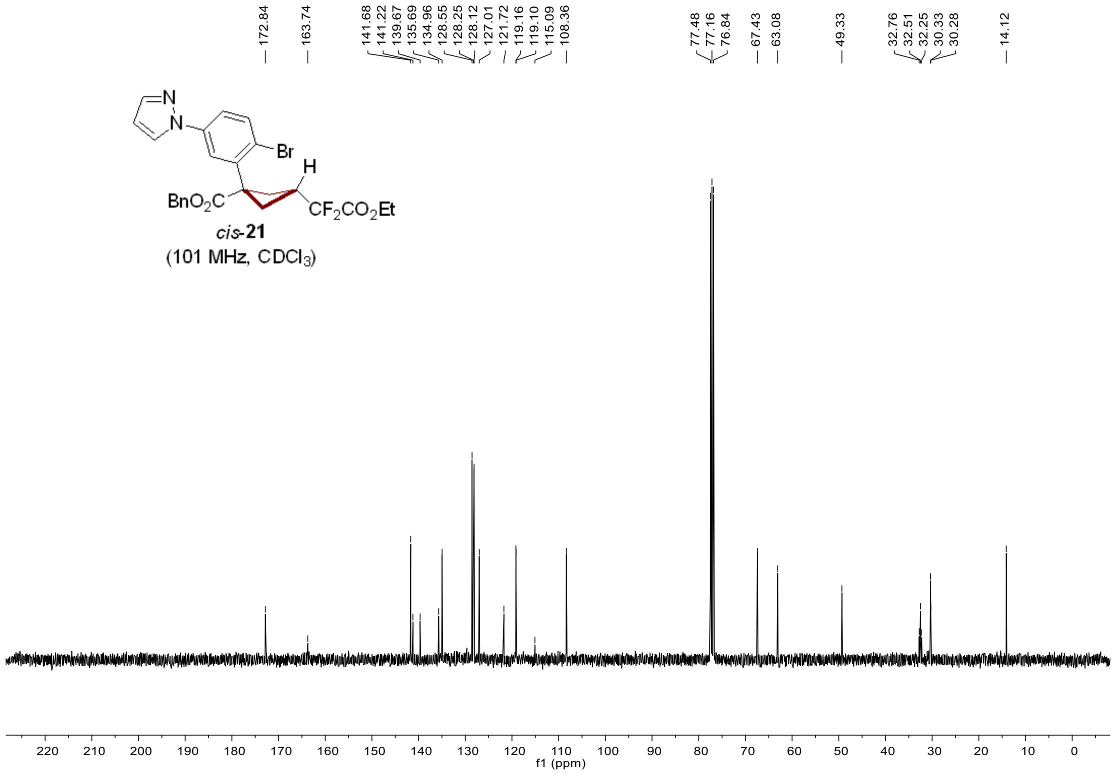

172.84
163.74
141.68
141.22
139.67
135.69
134.96
128.55
128.25
128.12
127.01
121.72
119.16
119.10
115.09
108.36
77.48
77.16
76.84
67.43
63.08
49.33
32.76
32.51
32.25
30.33
30.28
14.12
N
N
Br
H
$BnO_2C$
$CF_2CO_2Et$
*cis*-**21**
(101 MHz, $CDCl_3$)
220 210 200 190 180 170 160 150 140 130 120 110 100 90 80 70 60 50 40 30 20 10 0
f1 (ppm)


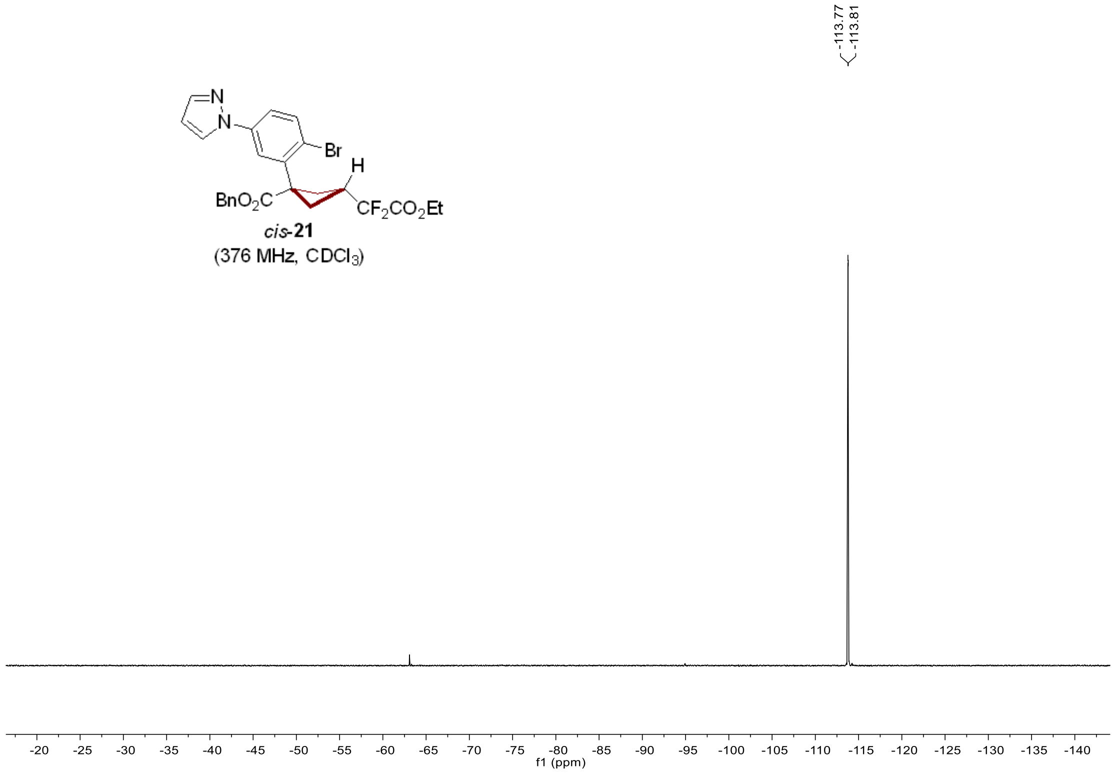

-113.77
-113.81
N
N
Br
H
$BnO_2C$
$CF_2CO_2Et$
*cis*-**21**
(376 MHz, $CDCl_3$)
-20 -25 -30 -35 -40 -45 -50 -55 -60 -65 -70 -75 -80 -85 -90 -95 -100 -105 -110 -115 -120 -125 -130 -135 -140
f1 (ppm)

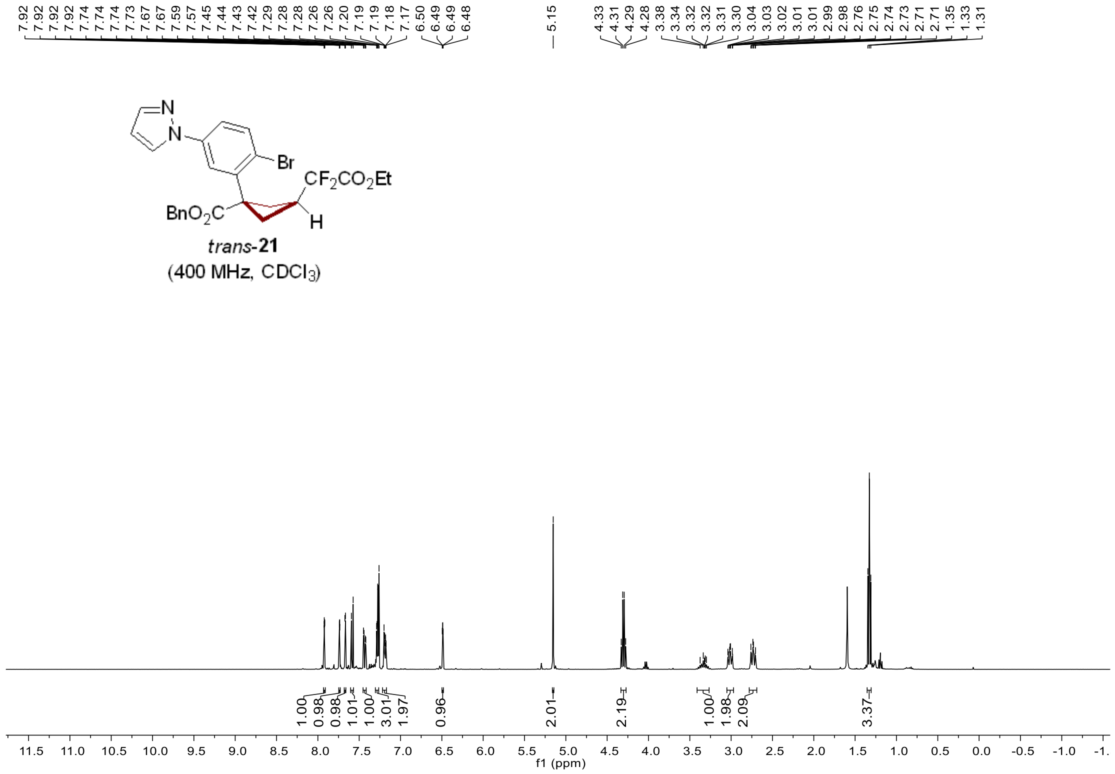
Br
CF2CO2Et
BnO2C
H
trans-21
(400 MHz, CDCl3)
f1 (ppm)

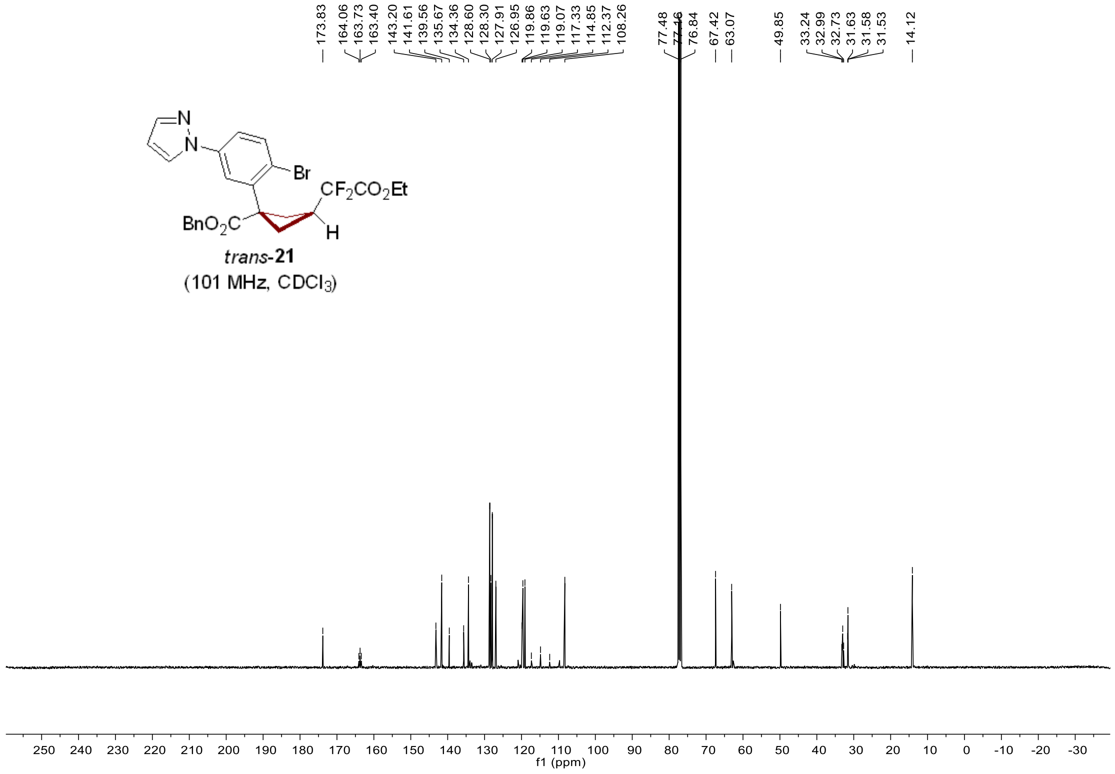
Br
CF2CO2Et
BnO2C
H
trans-21
(101 MHz, CDCl3)
f1 (ppm)

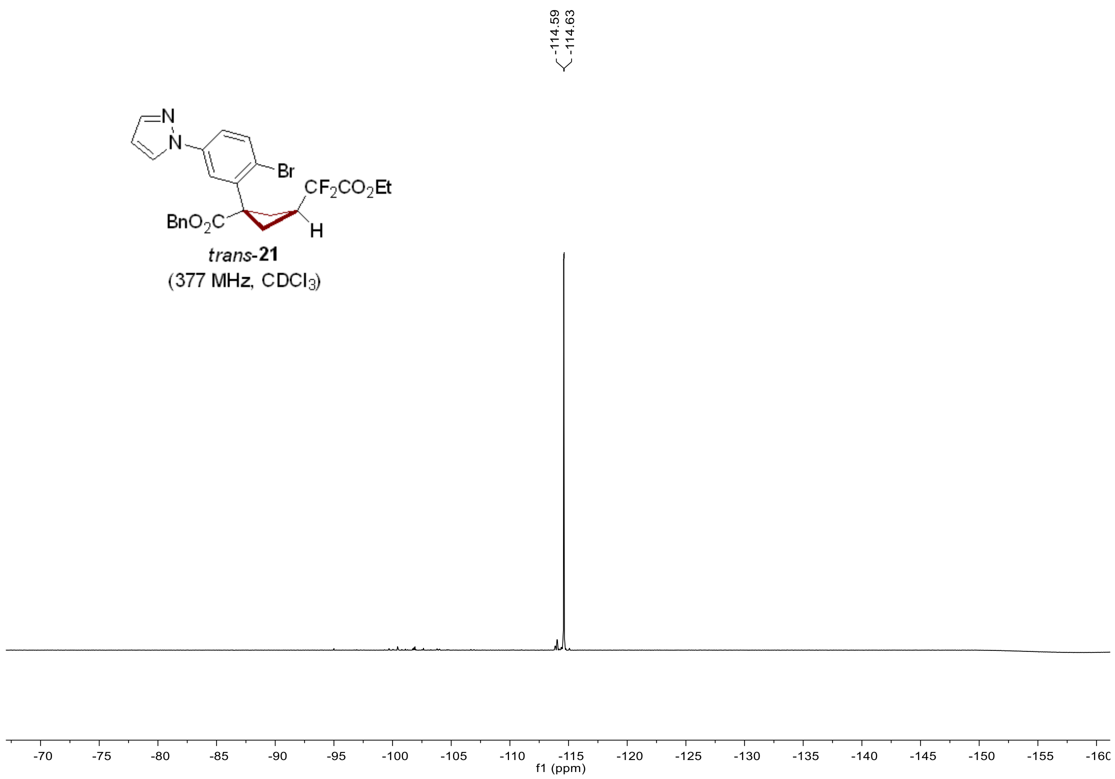
-114.59
-114.63
trans-21
(377 MHz, CDCl3)
f1 (ppm)

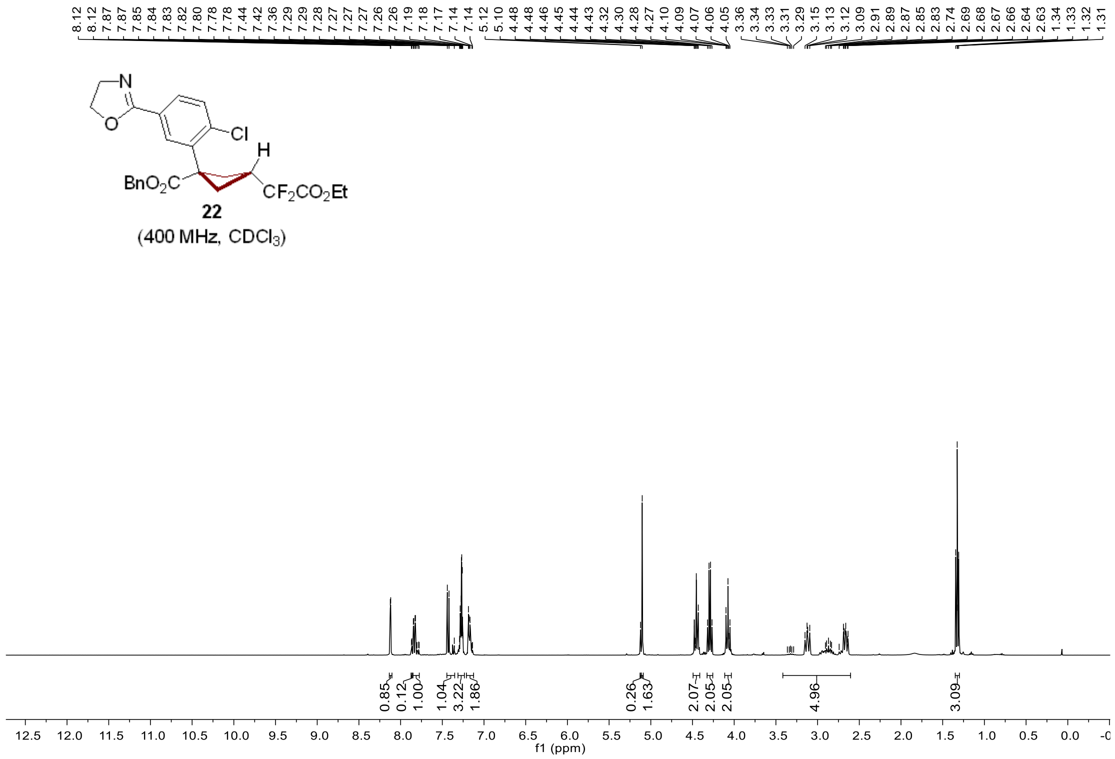
8.12 8.12 7.87 7.87 7.85 7.84 7.83 7.82 7.80 7.78 7.44 7.42 7.36 7.29 7.29 7.28 7.27 7.27 7.27 7.26 7.19 7.18 7.17 7.17 7.14 7.14 5.12 5.10 4.48 4.46 4.45 4.44 4.43 4.32 4.30 4.28 4.27 4.10 4.09 4.07 4.06 4.05 3.36 3.34 3.33 3.31 3.29 3.15 3.13 3.12 3.09 2.91 2.89 2.87 2.85 2.83 2.74 2.69 2.68 2.67 2.66 2.64 2.63 1.34 1.33 1.32 1.31
22
(400 MHz, CDCl3)
0.85 0.12 1.00 1.04 3.22 1.86 0.26 1.63 2.07 2.05 2.05 4.96 3.09
f1 (ppm)

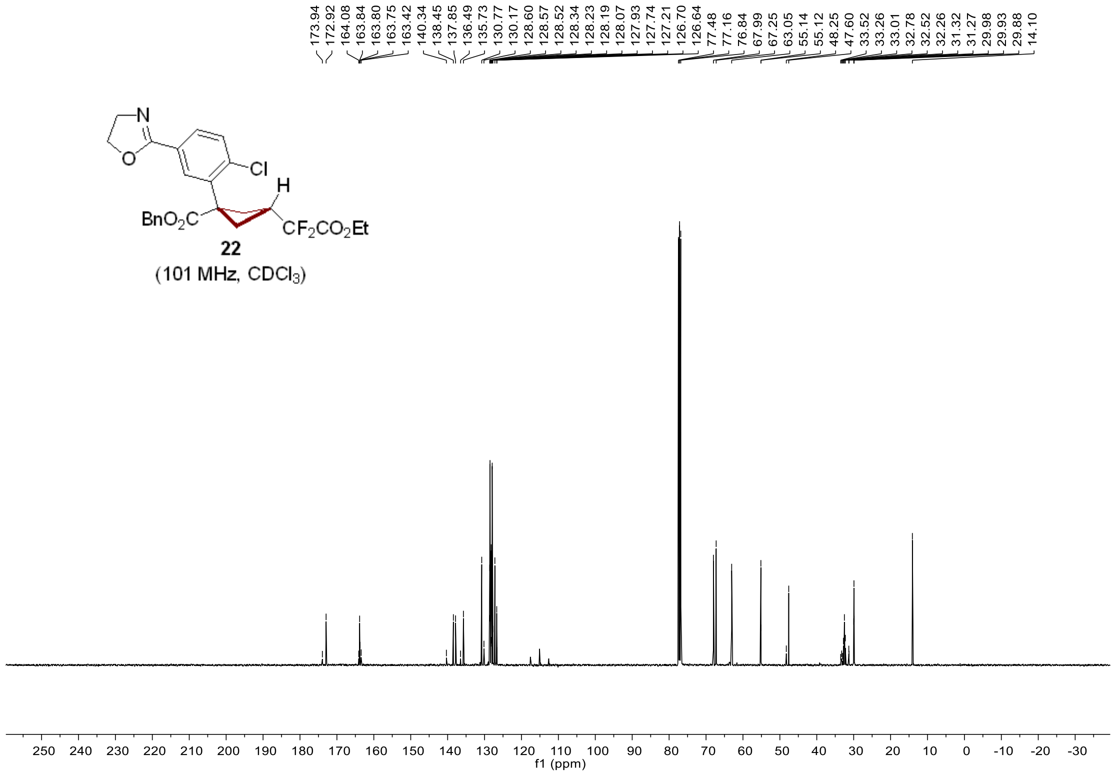
173.94
172.92
164.08
163.84
163.80
163.75
163.42
140.34
138.45
137.85
136.49
135.73
130.77
130.17
128.60
128.57
128.52
128.34
128.23
128.19
128.07
127.93
127.74
127.21
126.70
126.64
77.48
77.16
76.84
67.99
67.25
63.05
55.14
55.12
48.25
47.60
33.52
33.26
33.01
32.78
32.52
32.26
31.32
31.27
29.98
29.93
29.88
14.10
N
O
Cl
H
BnO2C
CF2CO2Et
22
(101 MHz, CDCl3)
f1 (ppm)

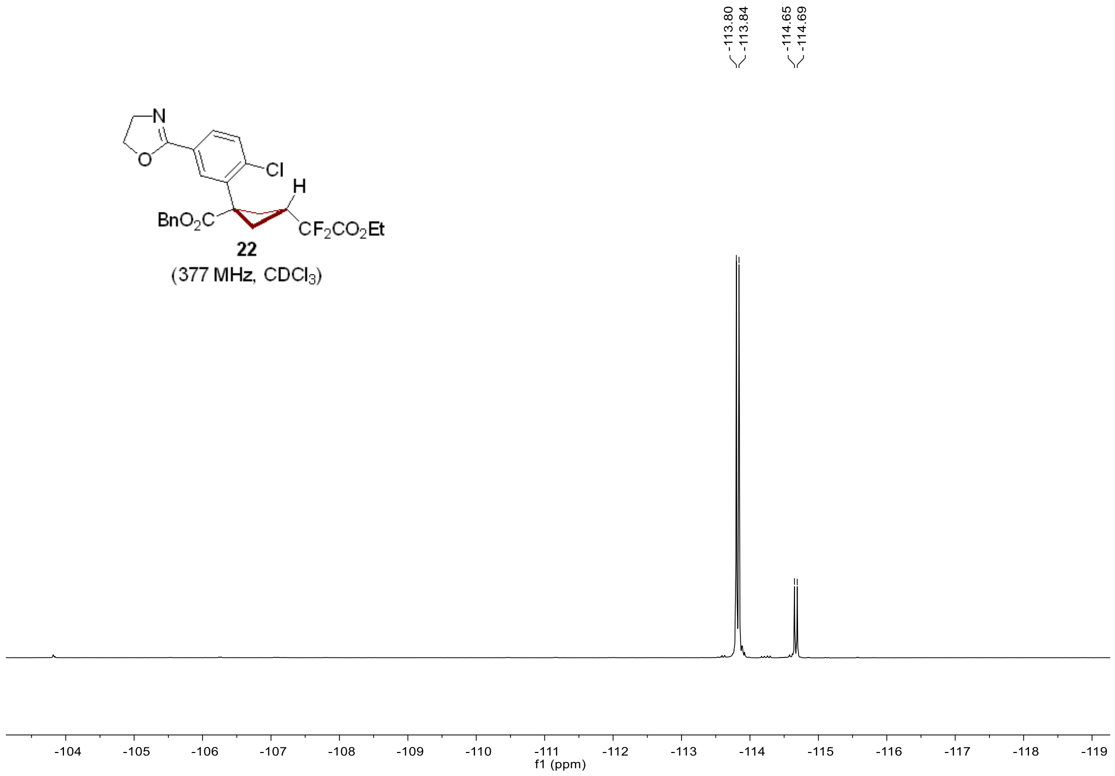
-113.80
-113.84
-114.65
-114.69
N
O
Cl
H
BnO2C
CF2CO2Et
22
(377 MHz, CDCl3)
f1 (ppm)

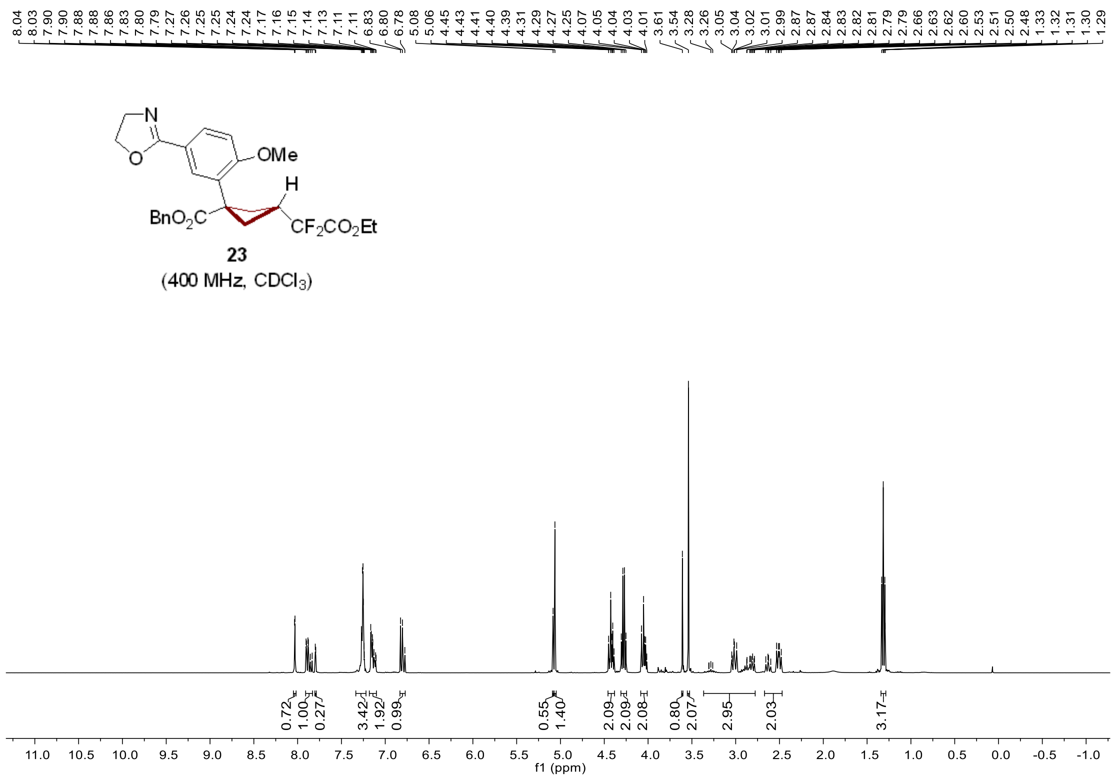


**23**
(400 MHz, $CDCl_3$)

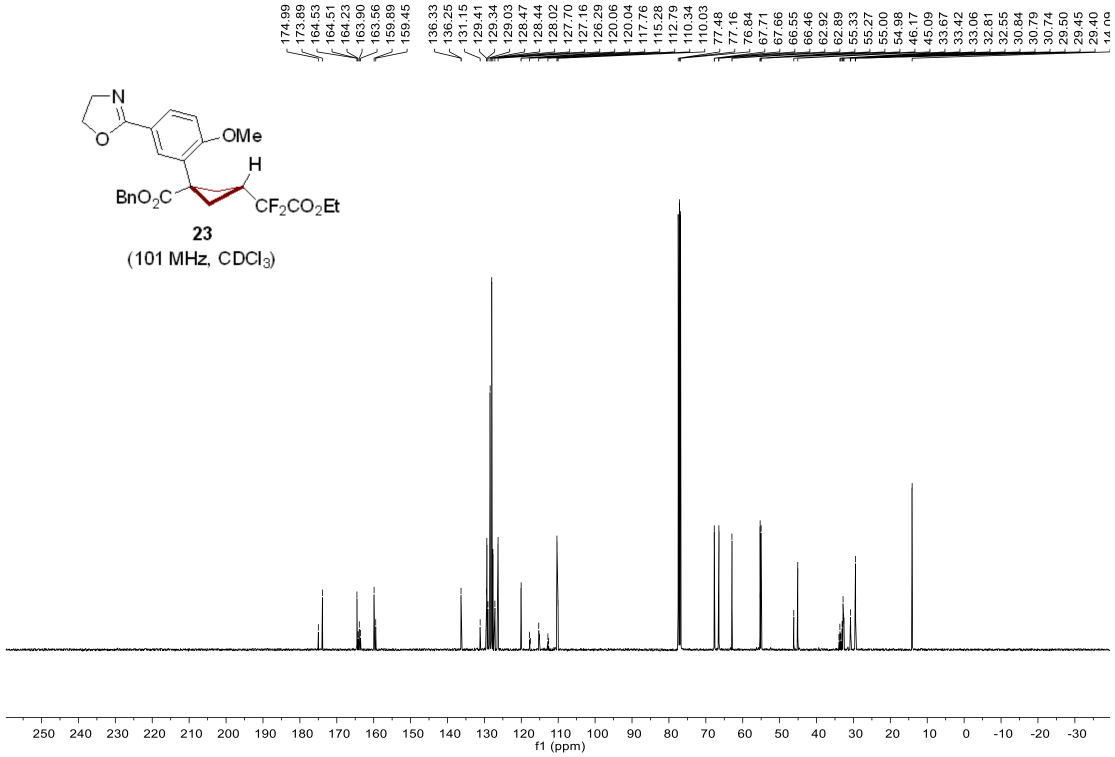


**23**
(101 MHz, $CDCl_3$)

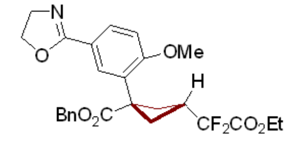

**23**
(377 MHz, $CDCl_3$)

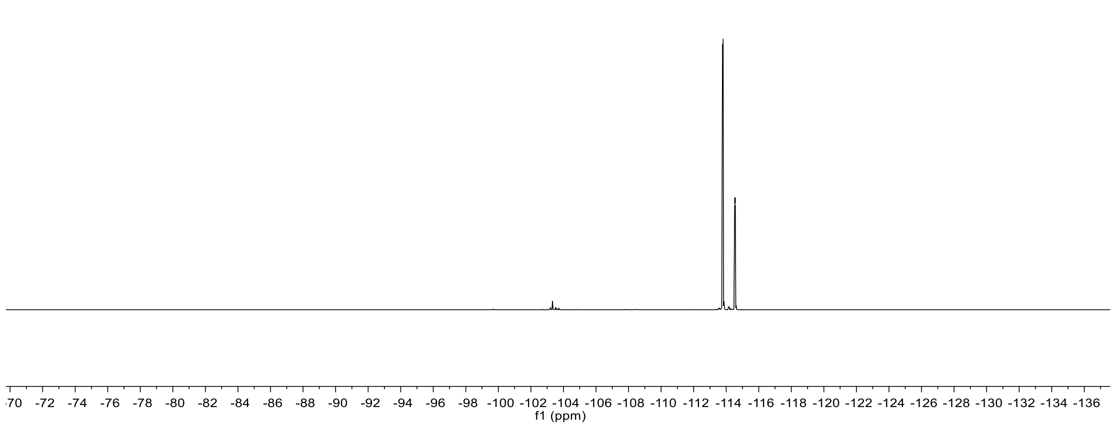


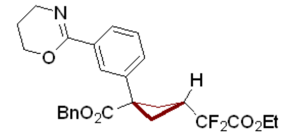

**24**
(400 MHz, $CDCl_3$)

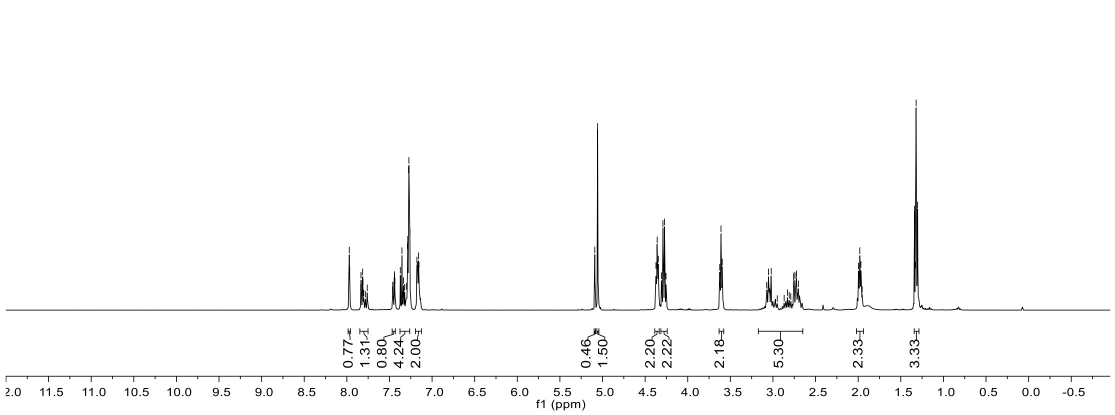

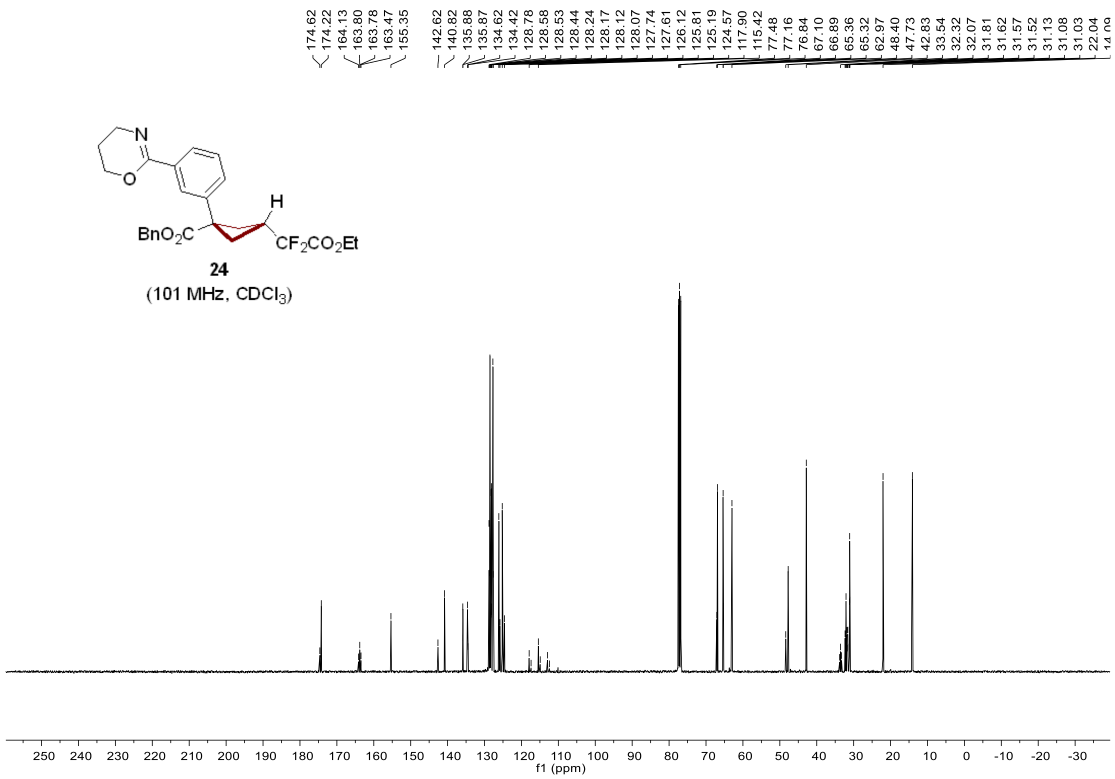
BnO2C
CF2CO2Et
H
N
O
24
(101 MHz, CDCl3)
174.62
174.22
164.13
163.80
163.78
163.47
155.35
142.62
140.82
135.88
135.87
134.62
134.42
128.78
128.58
128.53
128.44
128.24
128.17
128.12
128.07
127.74
127.61
126.12
125.81
125.19
124.57
117.90
115.42
77.48
77.16
76.84
67.10
66.89
65.36
65.32
62.97
48.40
47.73
42.83
33.54
32.32
32.07
31.81
31.62
31.57
31.52
31.13
31.08
31.03
22.04
f1 (ppm)

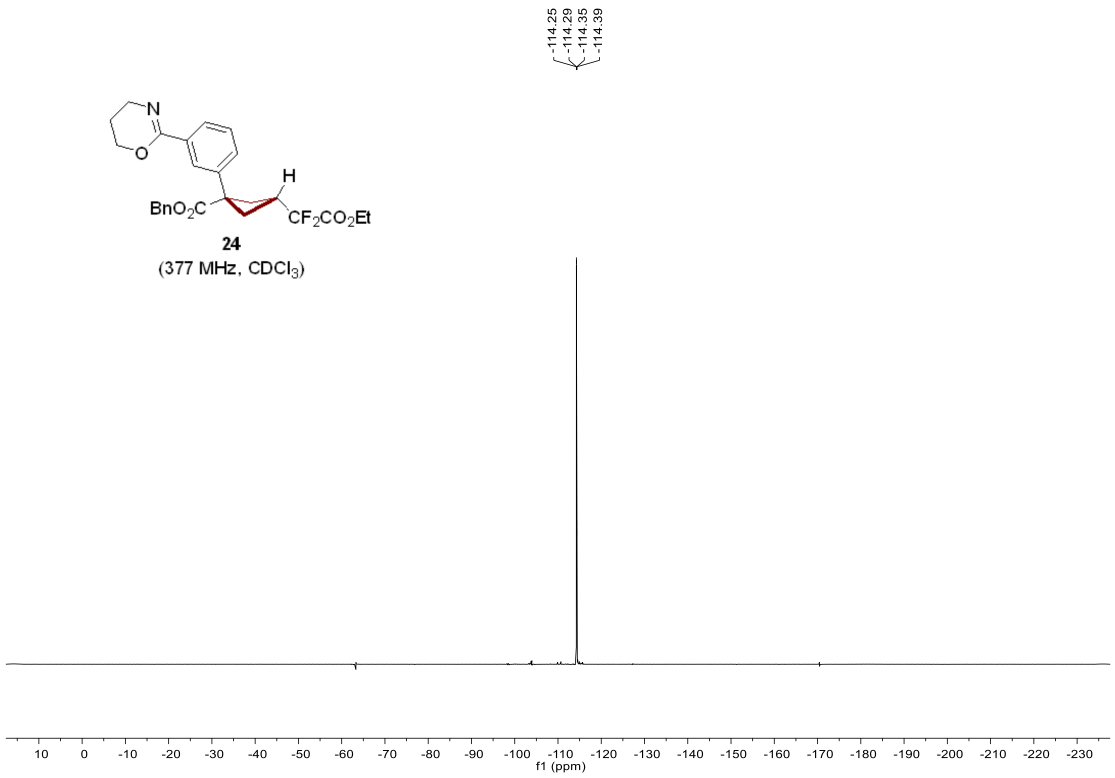
BnO2C
CF2CO2Et
H
N
O
24
(377 MHz, CDCl3)
-114.25
-114.29
-114.35
-114.39
f1 (ppm)

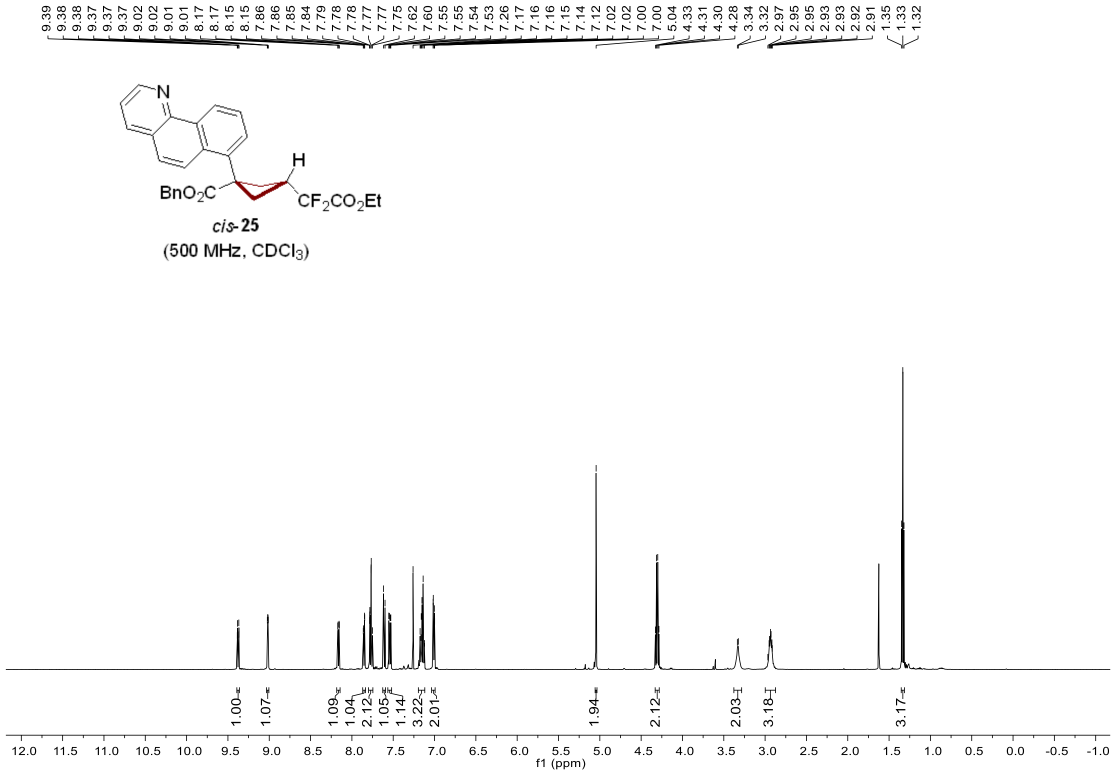


*cis*-**25**
(500 MHz, $CDCl_3$)

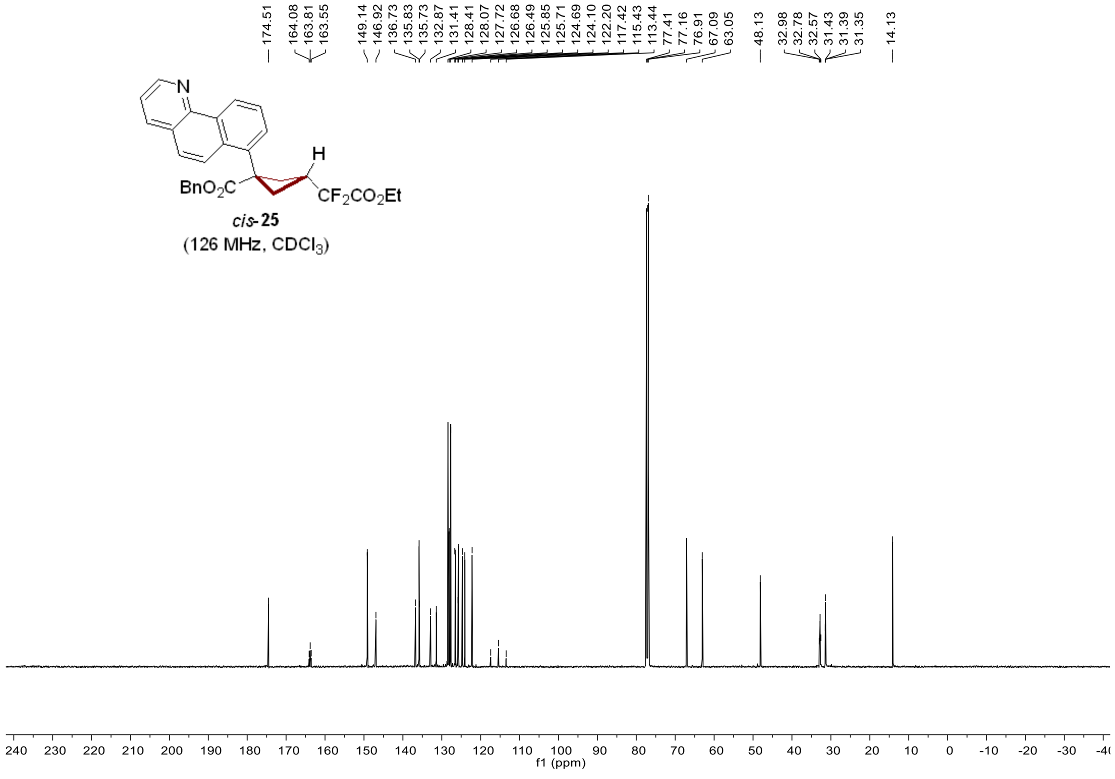


*cis*-**25**
(126 MHz, $CDCl_3$)

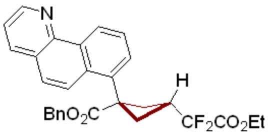

*cis*-**25**
(471 MHz, $CDCl_3$)

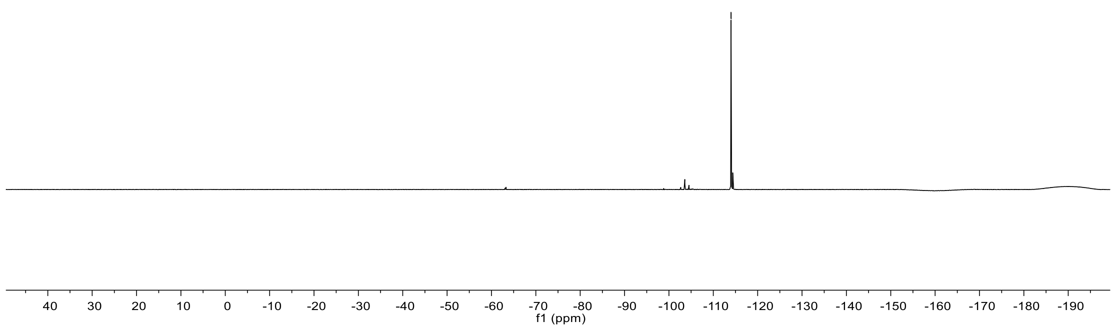


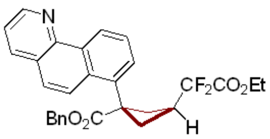

*trans*-**25**
(500 MHz, $CDCl_3$)

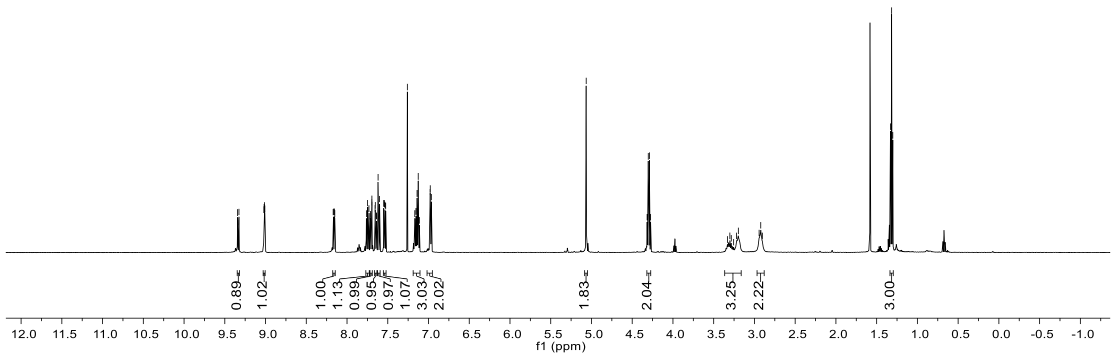

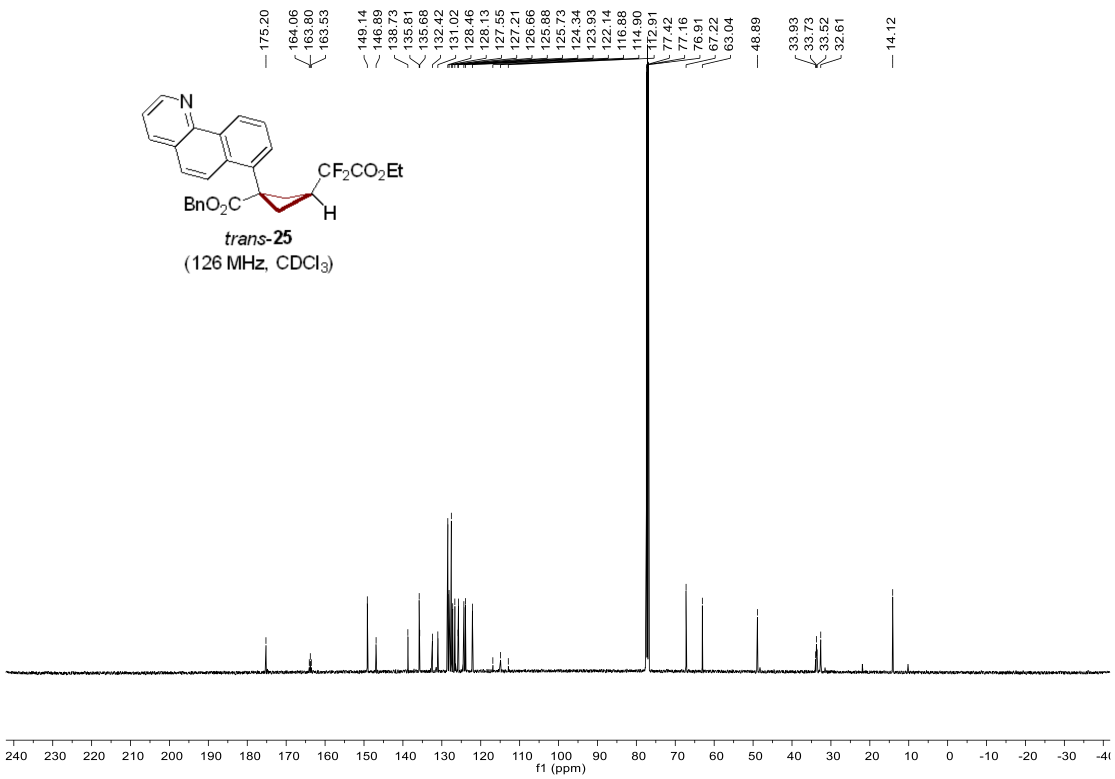
175.20
164.06
163.80
163.53
149.14
146.89
138.73
135.81
135.68
132.42
131.02
128.46
128.13
127.55
127.21
126.66
125.88
125.73
124.34
123.93
122.14
116.88
114.90
112.91
77.42
77.16
76.91
67.22
63.04
48.89
33.93
33.73
33.52
32.61
14.12
CF2CO2Et
BnO2C
H
N
trans-25
(126 MHz, CDCl3)
f1 (ppm)

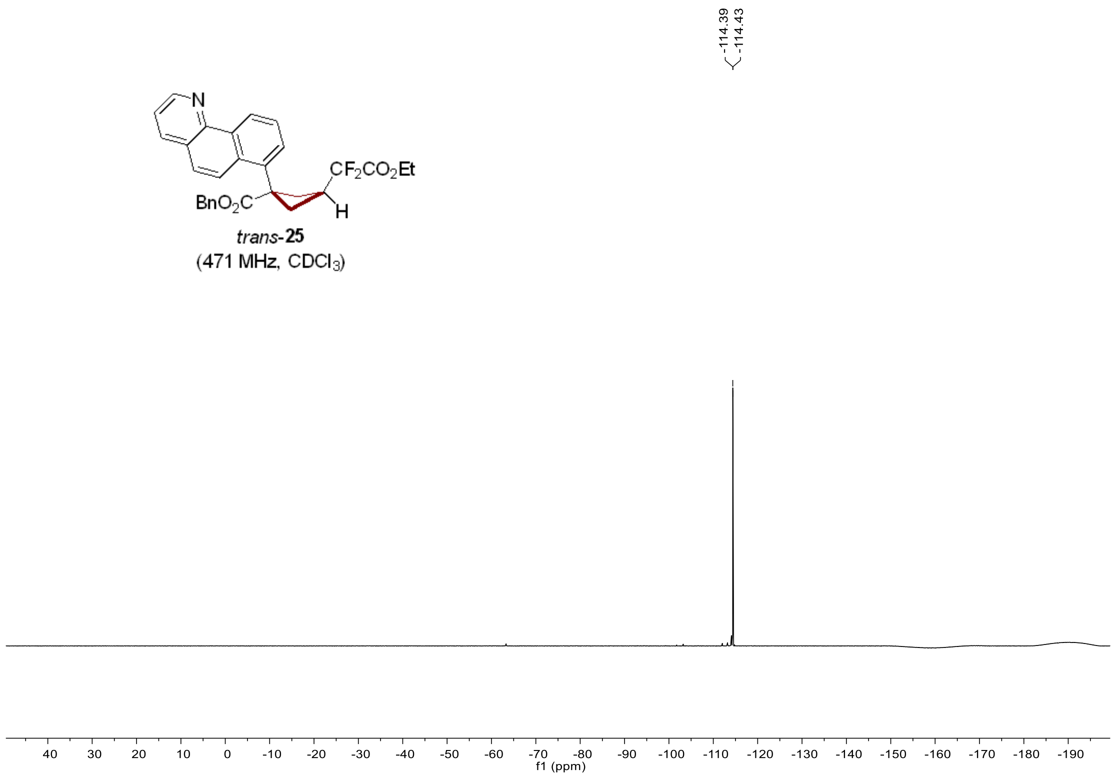
-114.39
-114.43
CF2CO2Et
BnO2C
H
N
trans-25
(471 MHz, CDCl3)
f1 (ppm)

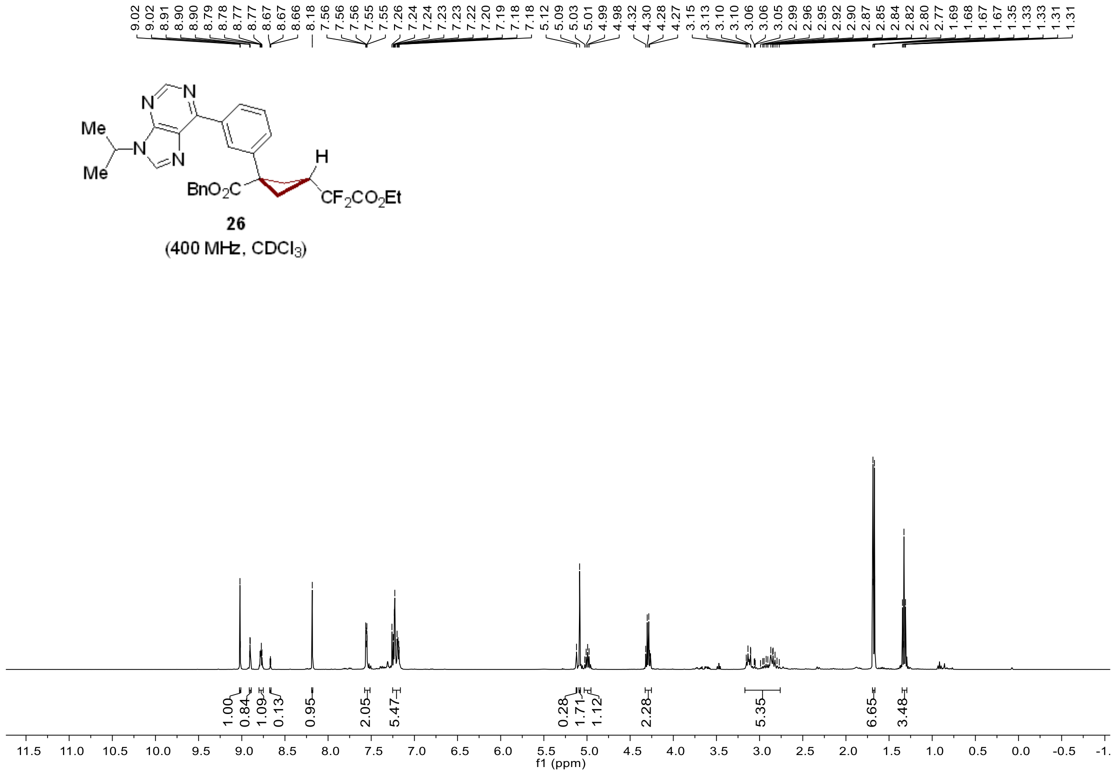

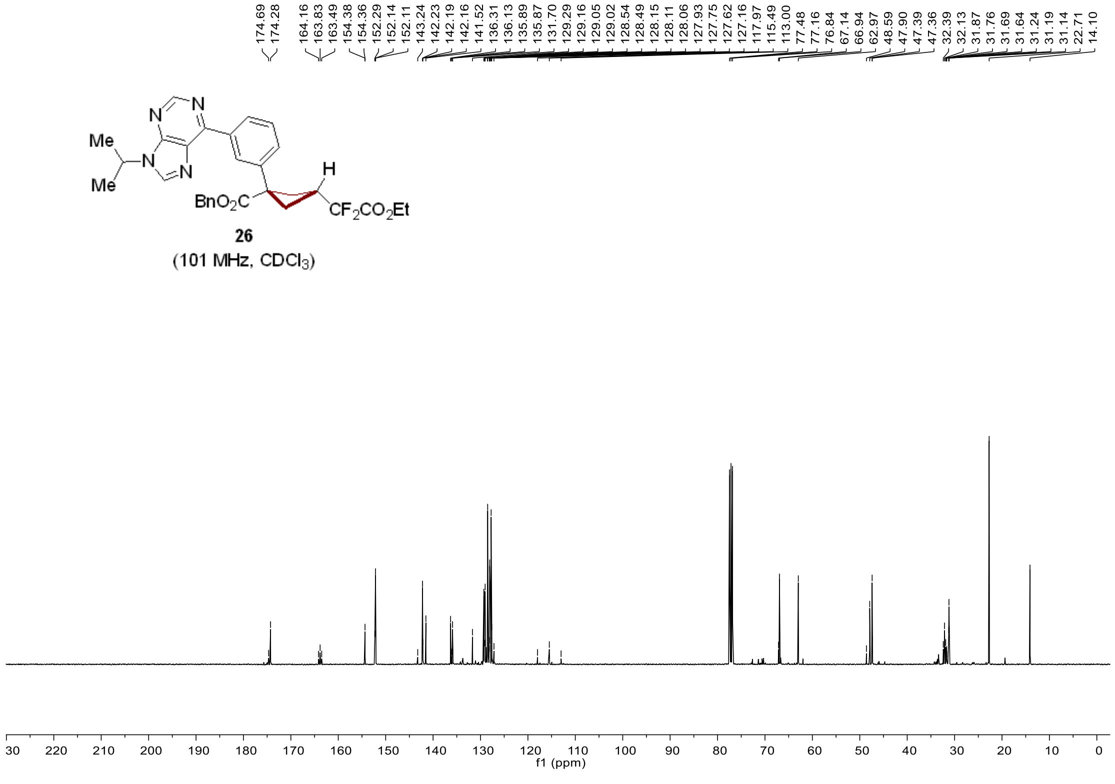

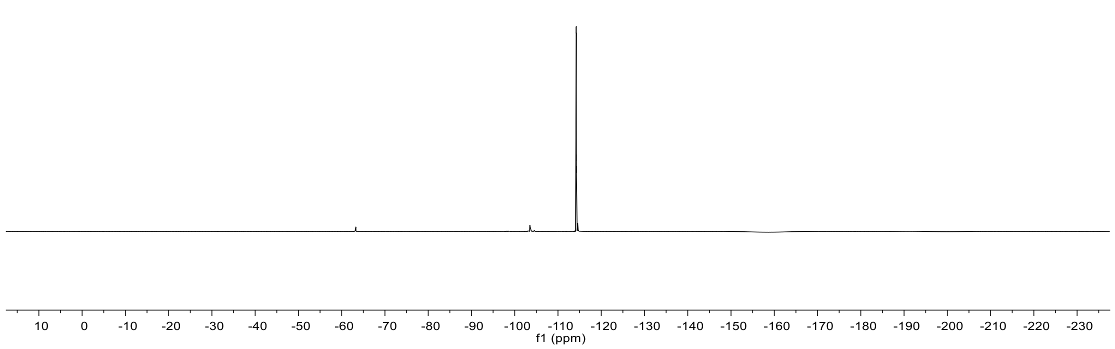


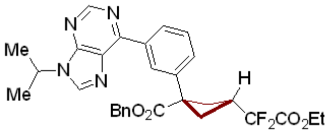


**26**

(377 MHz, $CDCl_3$)

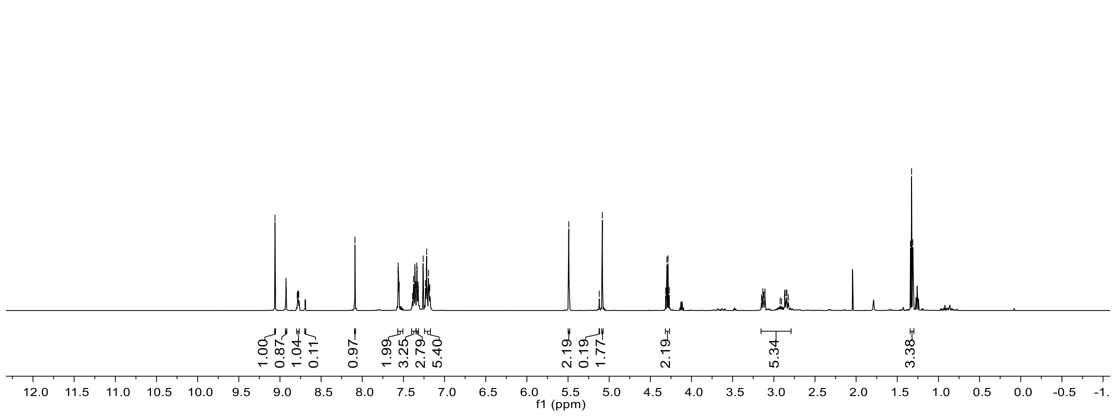


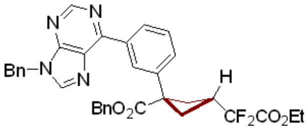


**27**

(500 MHz, $CDCl_3$)

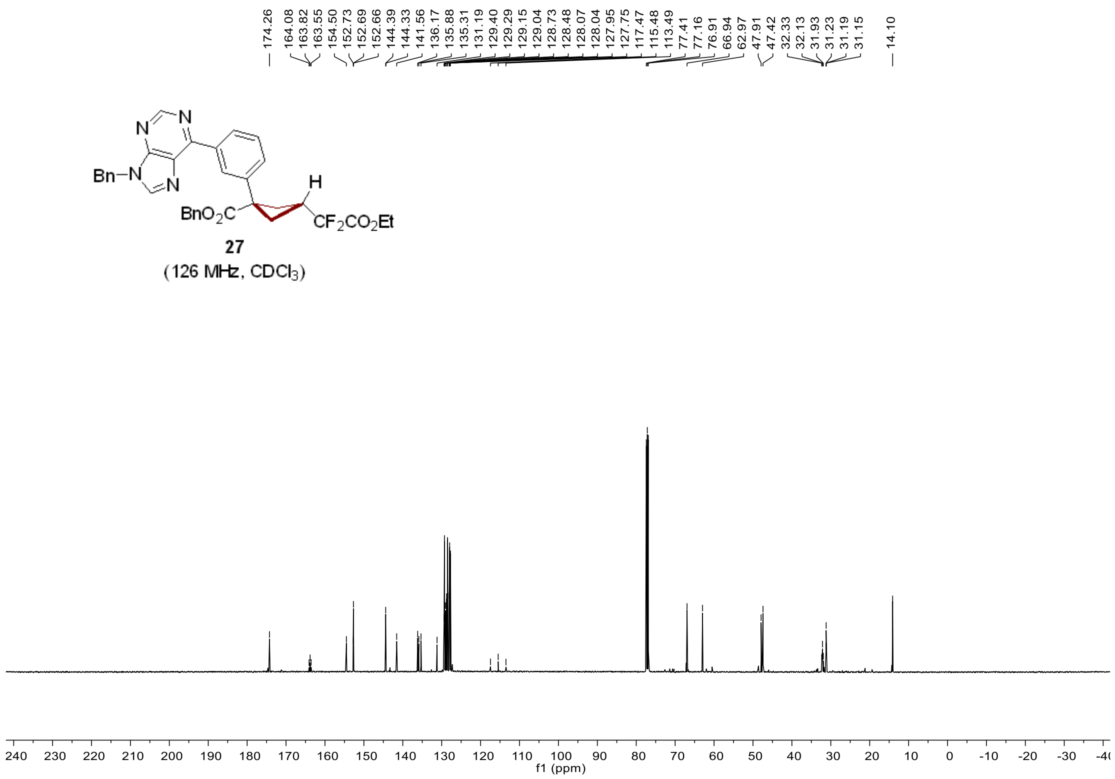


**27**
(126 MHz, $CDCl_3$)

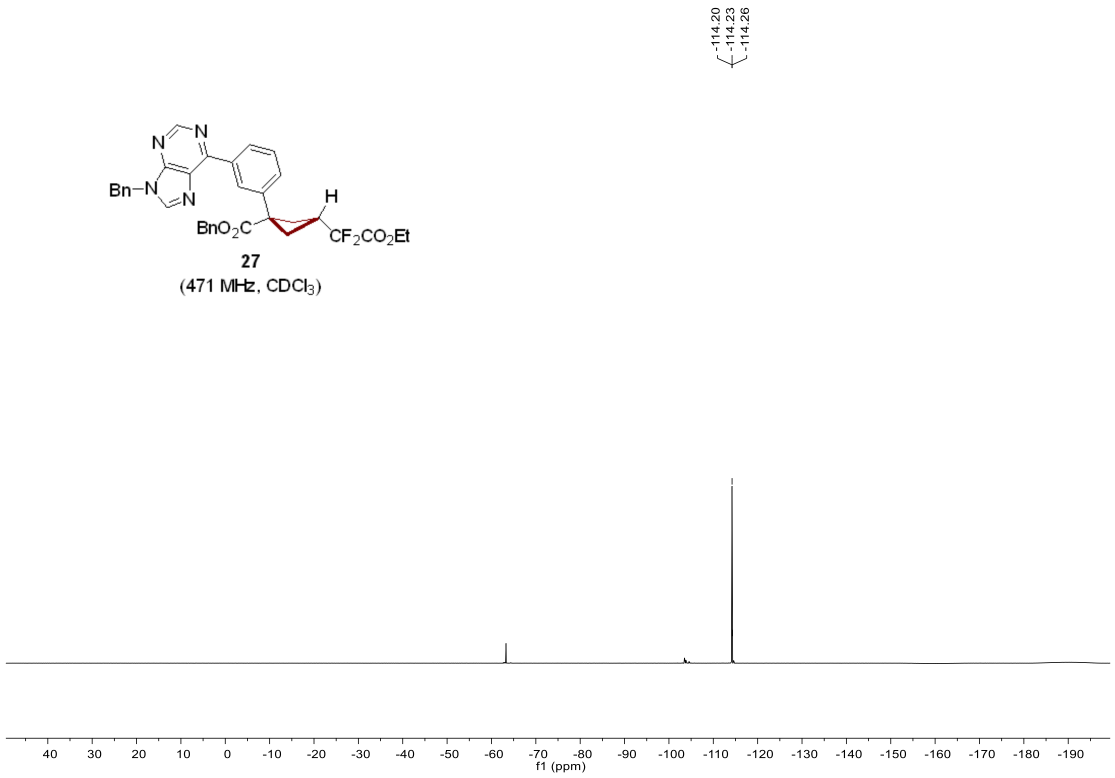


**27**
(471 MHz, $CDCl_3$)

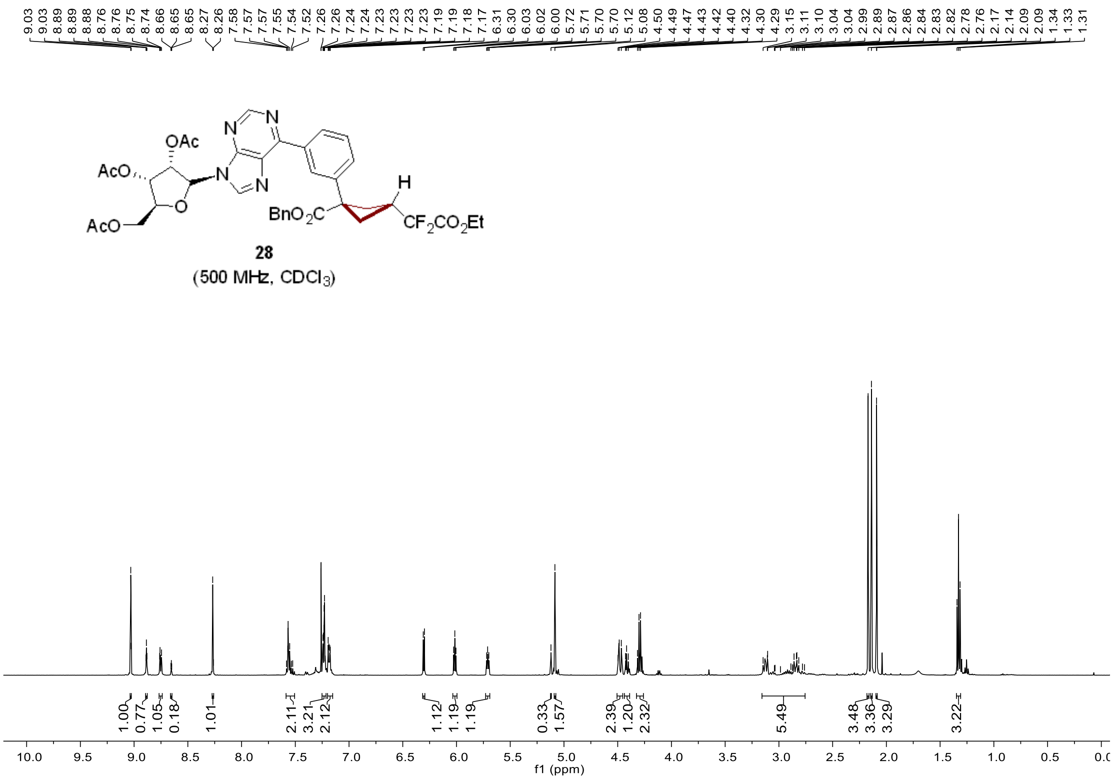

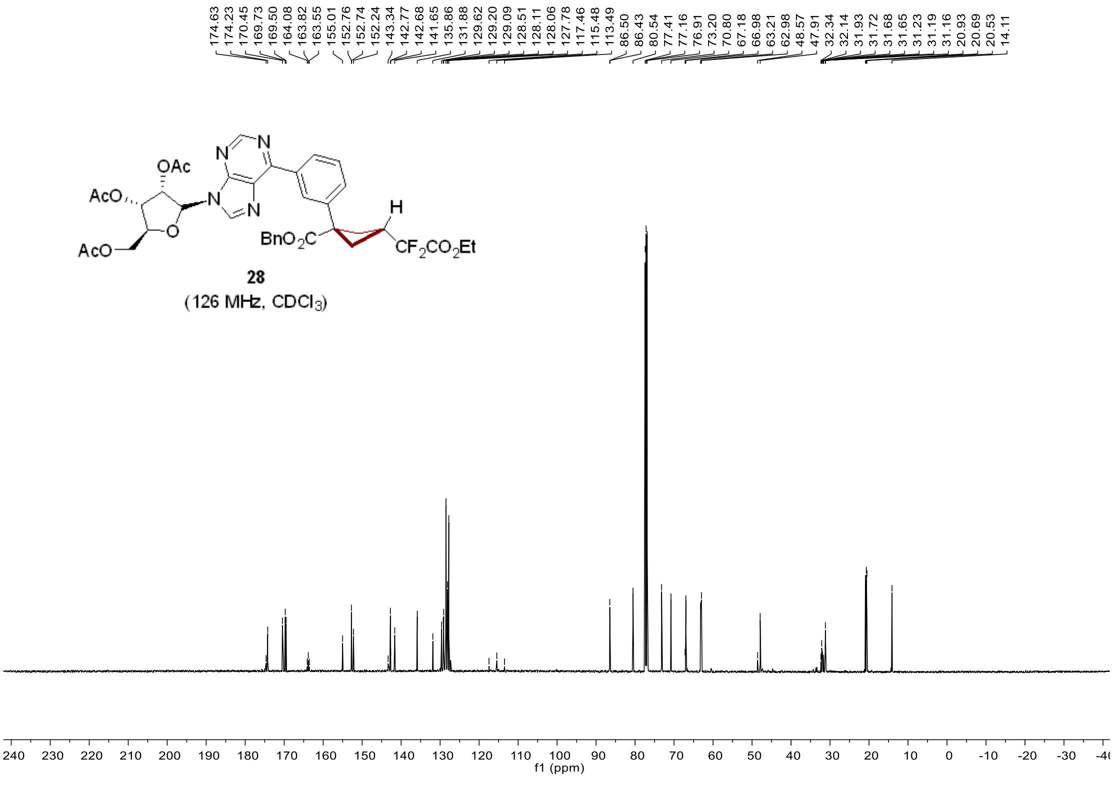

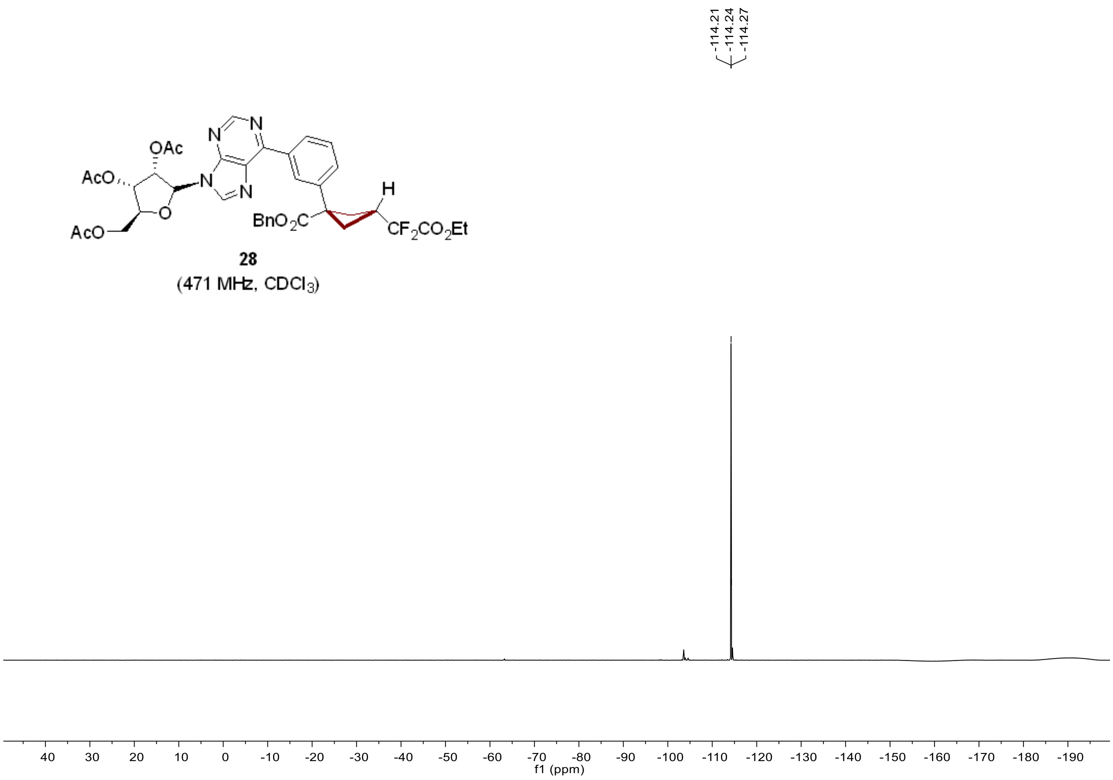


**28**
(471 MHz, $CDCl_3$)

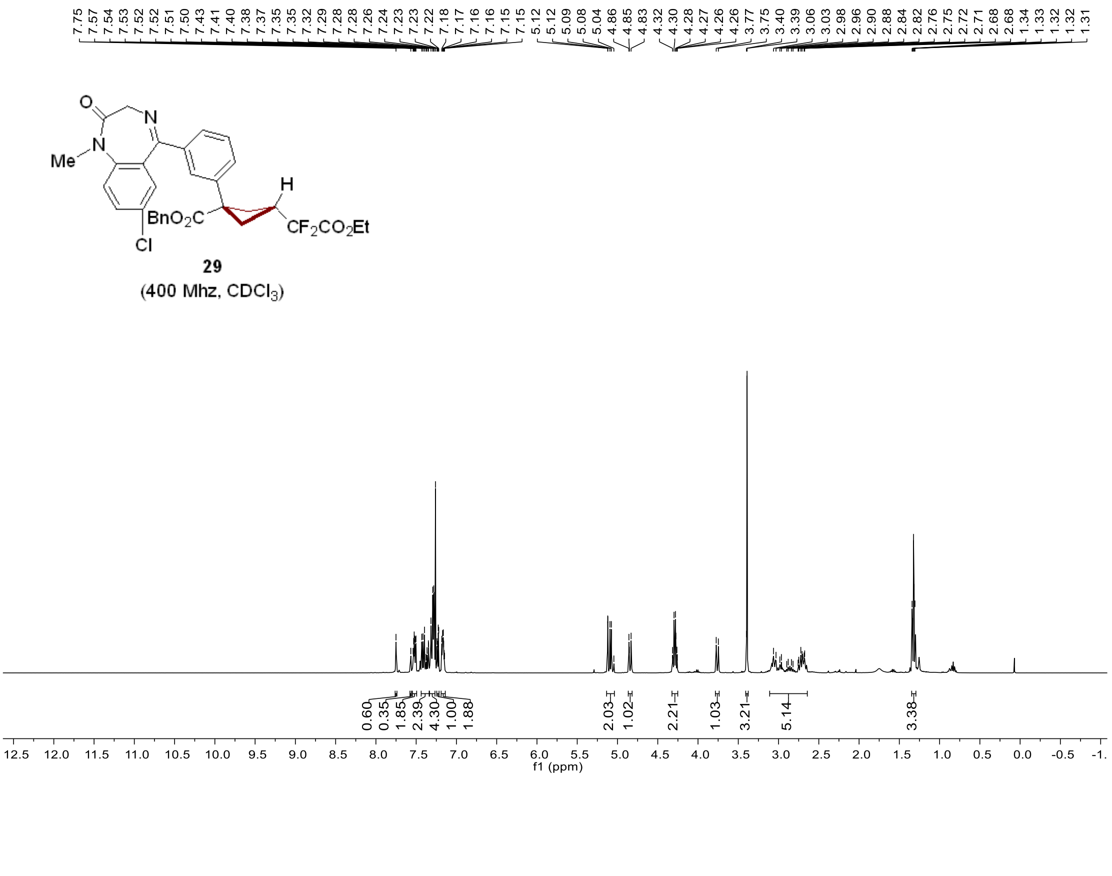


**29**
(400 Mhz, $CDCl_3$)

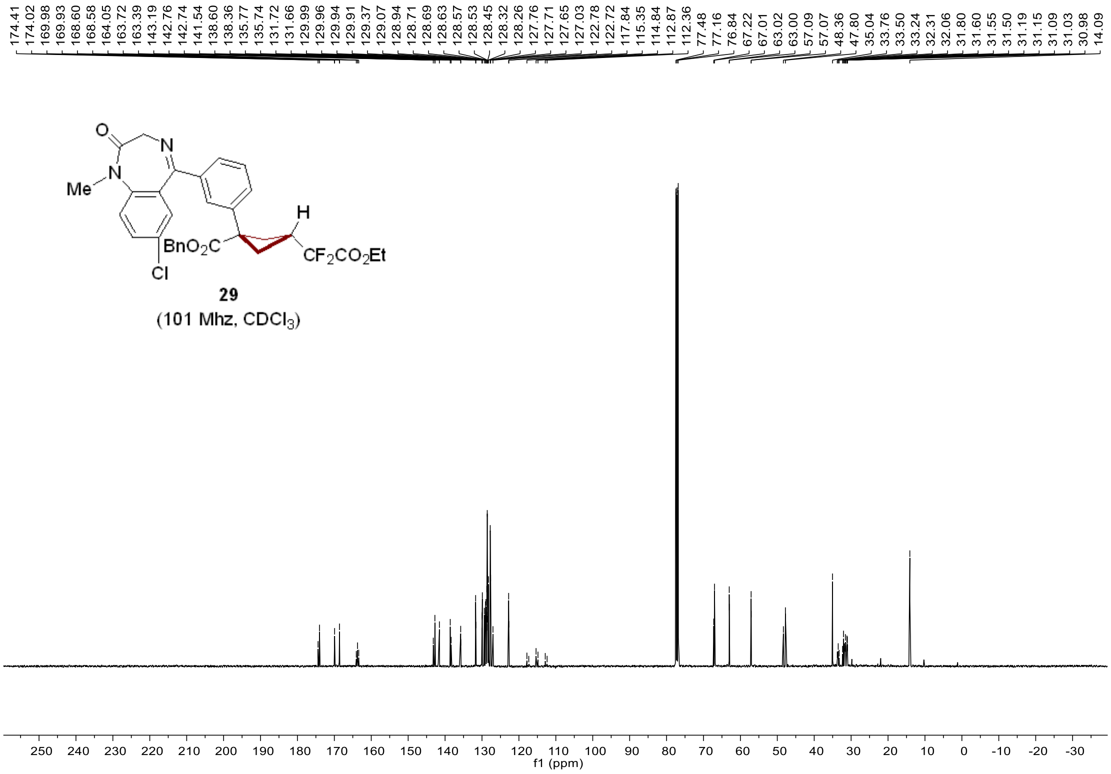
174.41
174.02
169.98
169.93
168.60
168.58
164.05
163.72
163.39
143.19
142.76
142.74
141.54
138.60
138.36
135.77
135.74
131.72
131.66
129.99
129.96
129.94
129.91
129.37
129.07
128.94
128.71
128.69
128.63
128.57
128.53
128.45
128.32
128.26
127.76
127.71
127.65
127.03
122.78
122.72
117.84
115.35
114.84
112.87
112.36
77.48
77.16
76.84
67.22
67.01
63.02
63.00
57.09
57.07
48.36
47.80
35.04
33.76
33.50
33.24
32.31
32.06
31.80
31.60
31.55
31.50
31.19
31.15
31.09
31.03
30.98
14.09
29
(101 Mhz, CDCl3)
f1 (ppm)

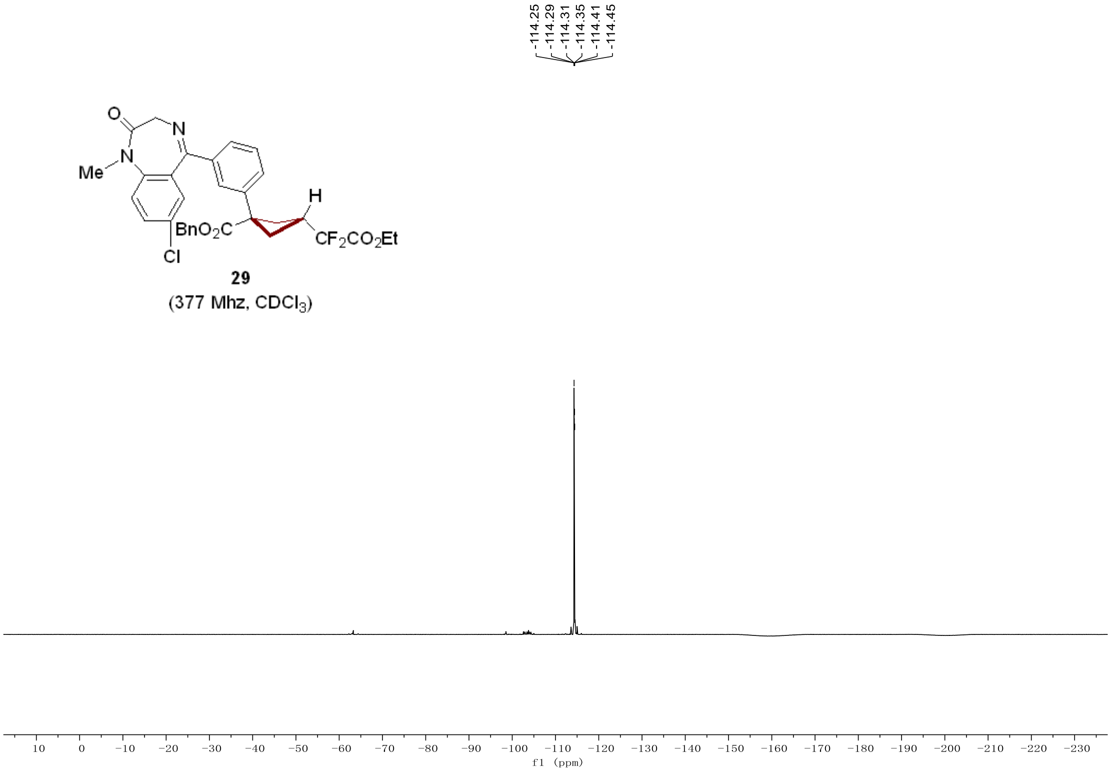
-114.25
-114.29
-114.31
-114.35
-114.41
-114.45
29
(377 Mhz, CDCl3)
f1 (ppm)

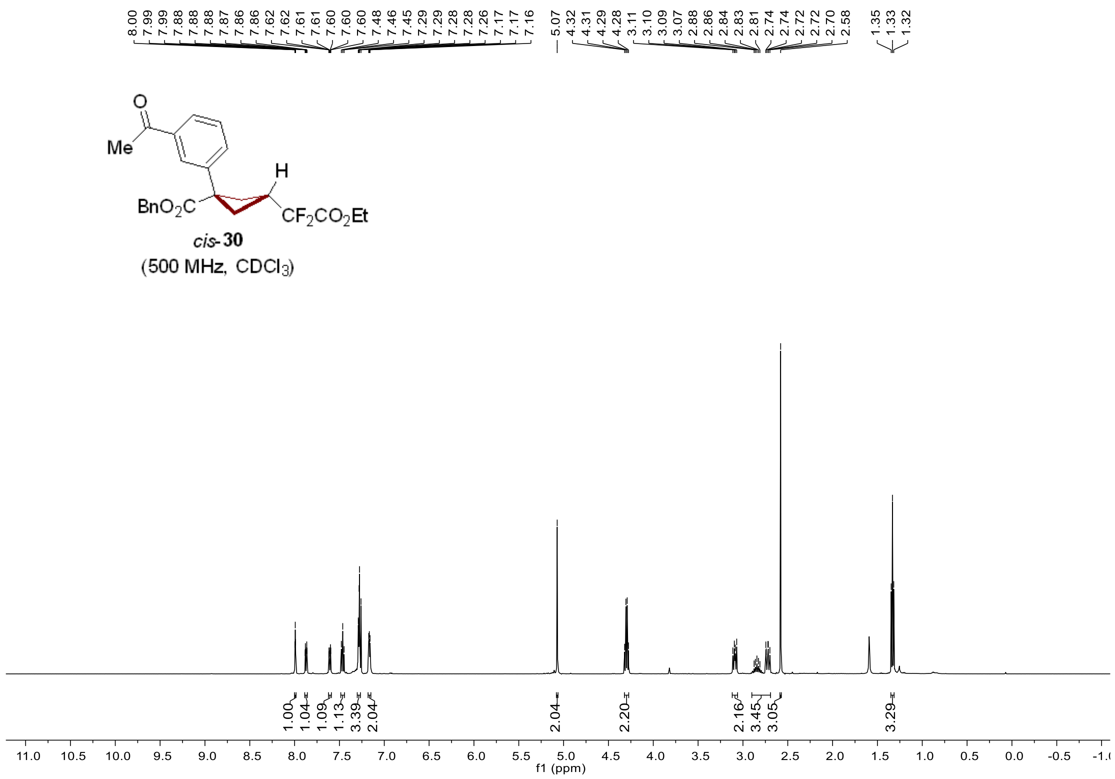
cis-30
(500 MHz, CDCl3)
f1 (ppm)

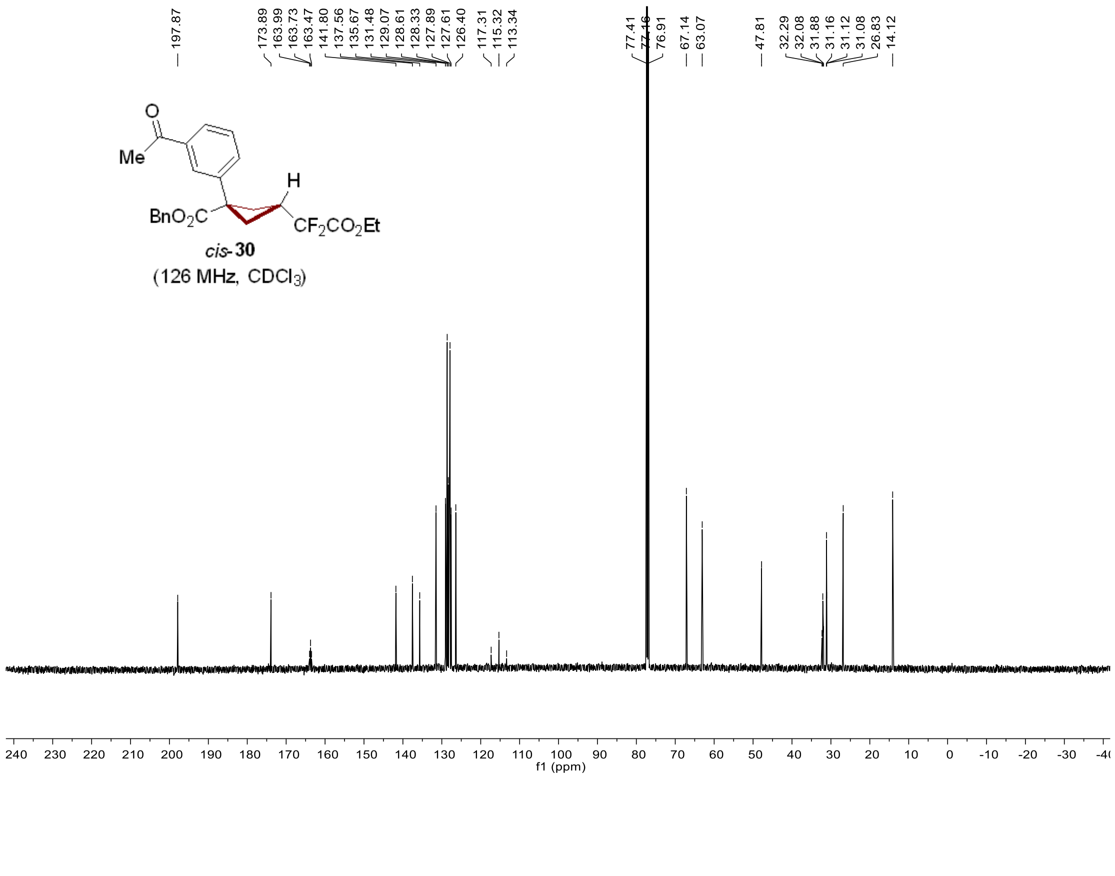
cis-30
(126 MHz, CDCl3)
f1 (ppm)

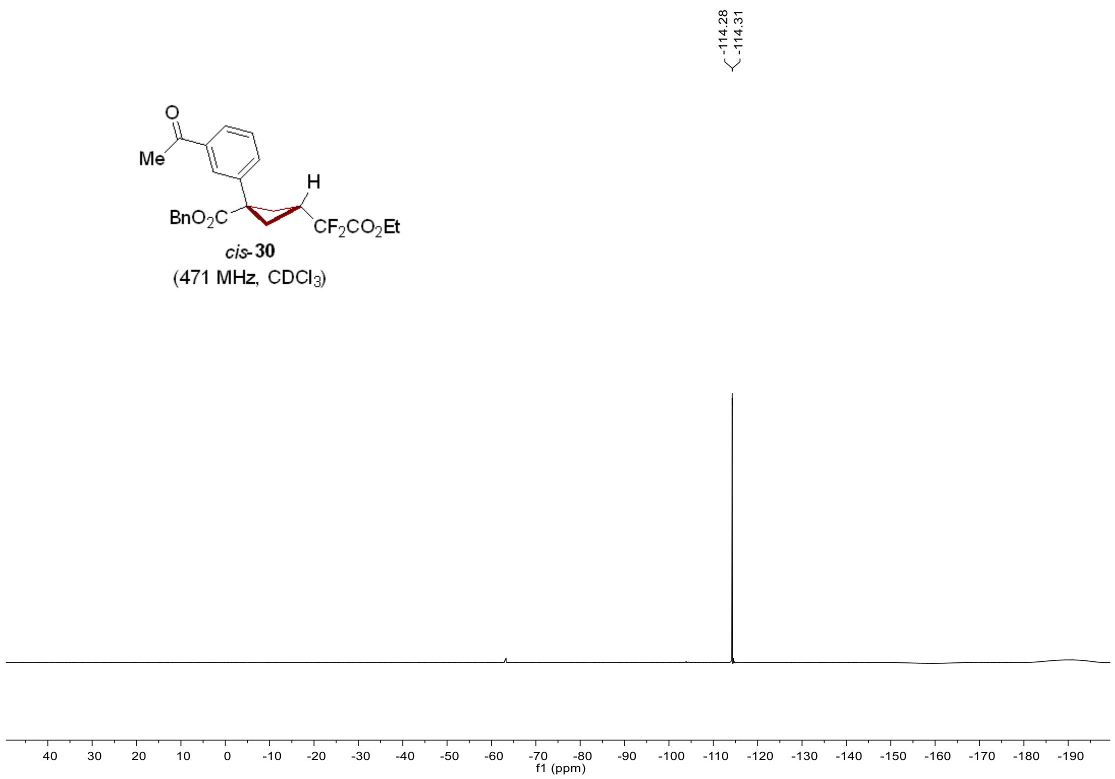

O
Me
H
BnO2C
CF2CO2Et
cis-30
(471 MHz, CDCl3)
-114.28
-114.31
f1 (ppm)


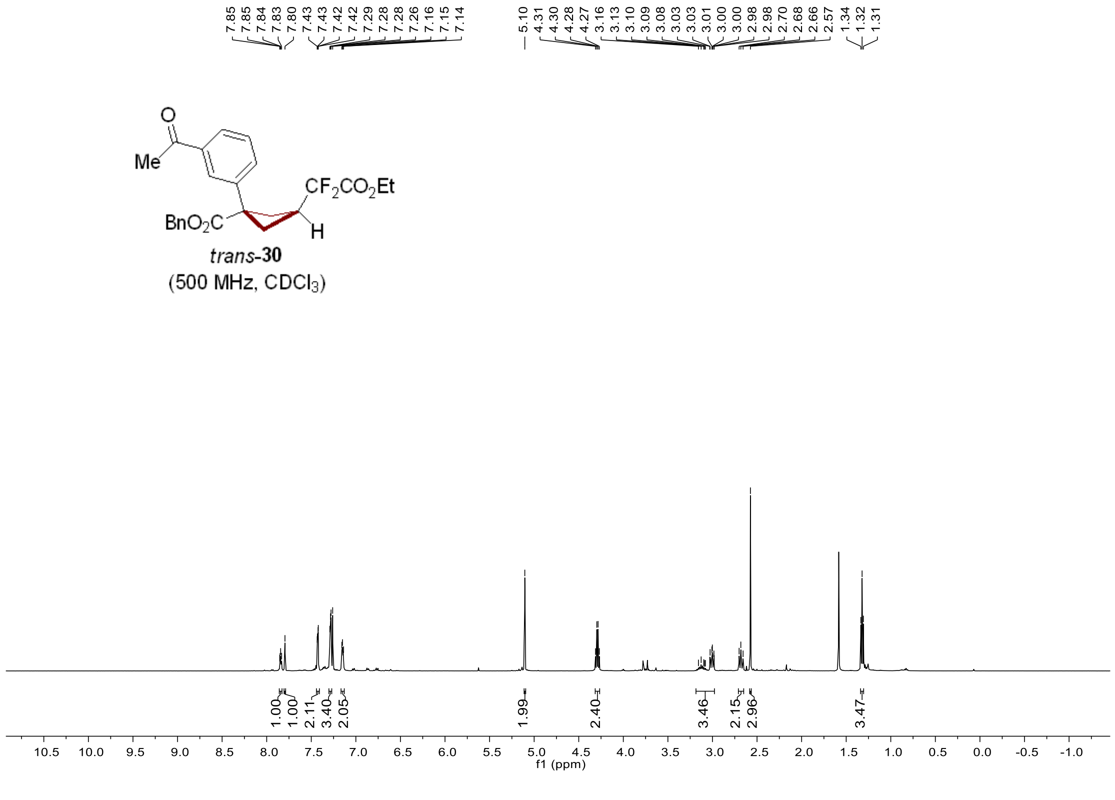

O
Me
CF2CO2Et
BnO2C
H
trans-30
(500 MHz, CDCl3)
f1 (ppm)

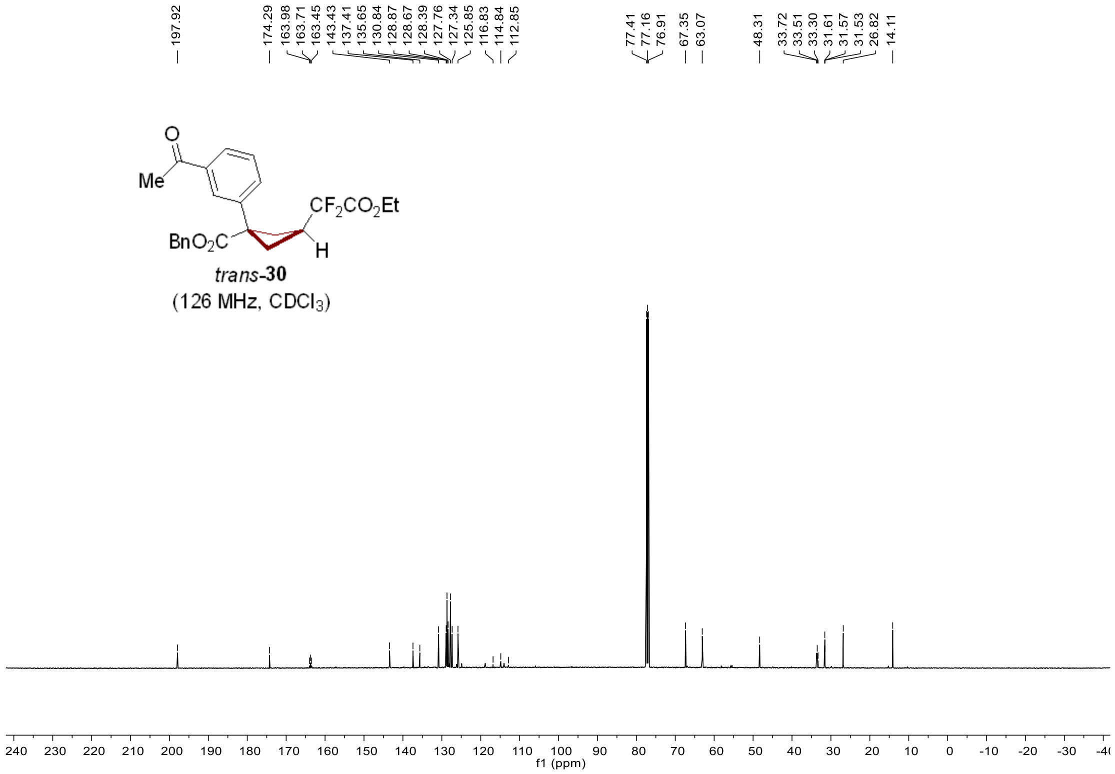
197.92
174.29
163.98
163.71
163.45
143.43
137.41
135.65
130.84
128.87
128.67
128.39
127.76
127.34
125.85
116.83
114.84
112.85
77.41
77.16
76.91
67.35
63.07
48.31
33.72
33.51
33.30
31.61
31.57
31.53
26.82
14.11
O
Me
CF2CO2Et
BnO2C
H
trans-30
(126 MHz, CDCl3)
f1 (ppm)

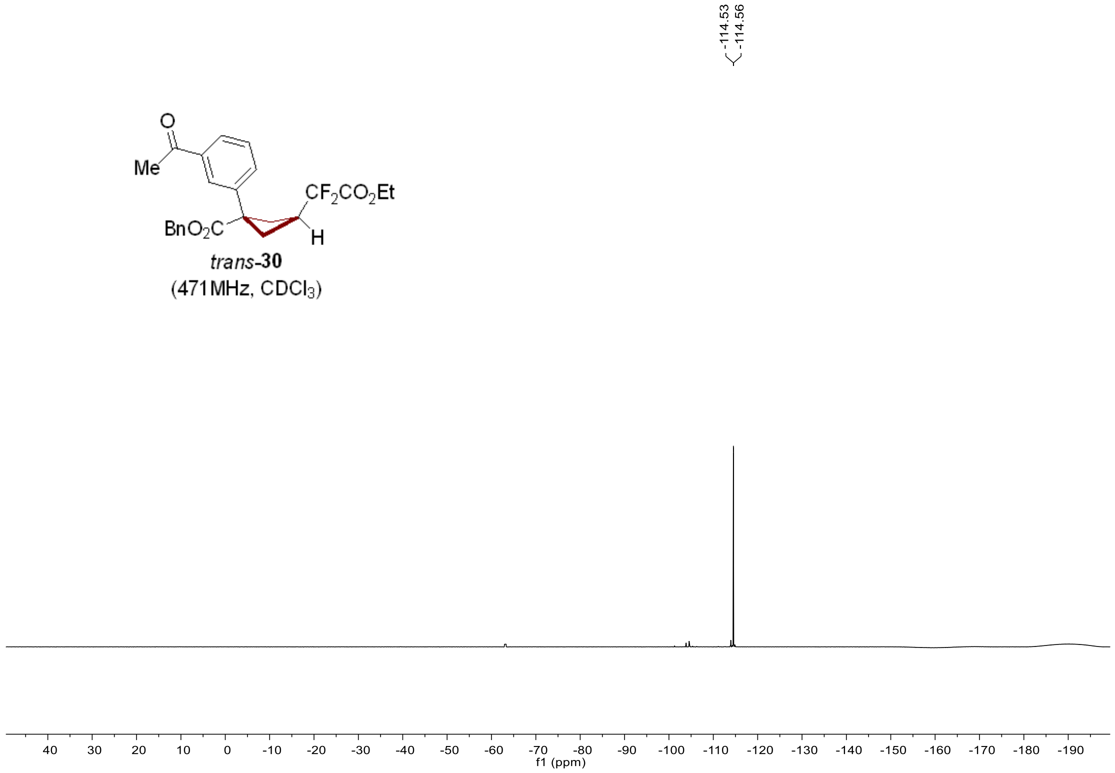
-114.53
-114.56
O
Me
CF2CO2Et
BnO2C
H
trans-30
(471MHz, CDCl3)
f1 (ppm)

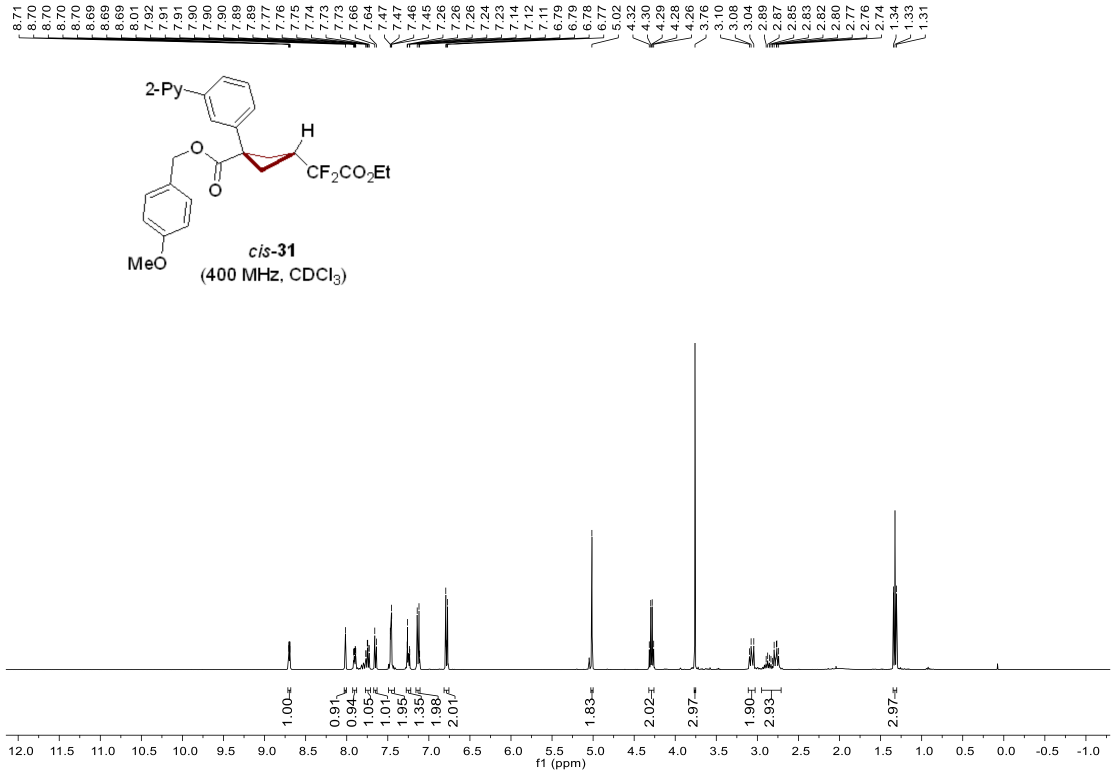
8.71 8.70 8.70 8.70 8.69 8.69 8.69 8.01 7.92 7.91 7.91 7.90 7.90 7.89 7.89 7.77 7.76 7.75 7.74 7.73 7.73 7.66 7.64 7.47 7.47 7.46 7.45 7.26 7.26 7.24 7.23 7.14 7.12 7.11 6.79 6.79 6.78 6.77 5.02 4.32 4.30 4.29 4.28 4.26 3.76 3.10 3.08 3.04 2.89 2.87 2.85 2.83 2.82 2.80 2.77 2.76 2.74 1.34 1.33 1.31
2-Py
H
O
CF2CO2Et
O
MeO
cis-31
(400 MHz, CDCl3)
1.00 0.91 0.94 1.05 1.01 1.95 1.35 1.98 2.01 1.83 2.02 2.97 1.90 2.93 2.97
12.0 11.5 11.0 10.5 10.0 9.5 9.0 8.5 8.0 7.5 7.0 6.5 6.0 5.5 5.0 4.5 4.0 3.5 3.0 2.5 2.0 1.5 1.0 0.5 0.0 -0.5 -1.0
f1 (ppm)

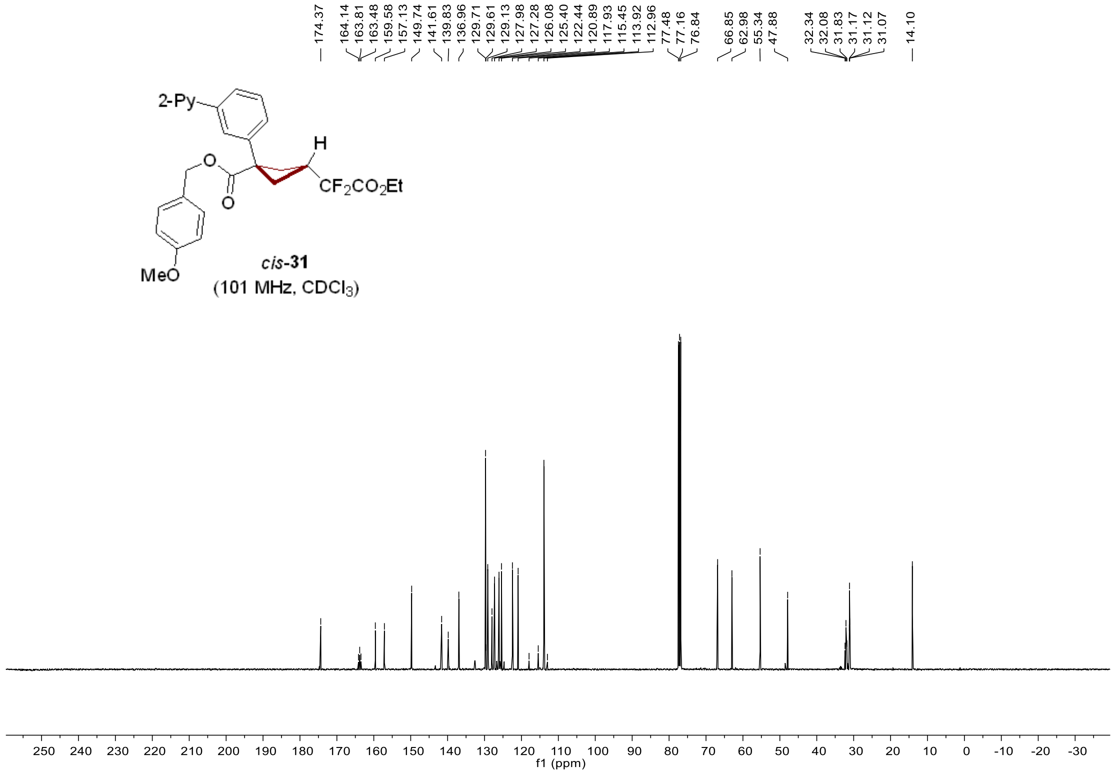
174.37 164.14 163.81 163.48 159.58 157.13 149.74 141.61 139.83 136.96 129.71 129.61 129.13 127.98 127.28 126.08 125.40 122.44 120.89 117.93 115.45 113.92 112.96 77.48 77.16 76.84 66.85 62.98 55.34 47.88 32.34 32.08 31.83 31.17 31.12 31.07 14.10
2-Py
H
O
CF2CO2Et
O
MeO
cis-31
(101 MHz, CDCl3)
250 240 230 220 210 200 190 180 170 160 150 140 130 120 110 100 90 80 70 60 50 40 30 20 10 0 -10 -20 -30
f1 (ppm)

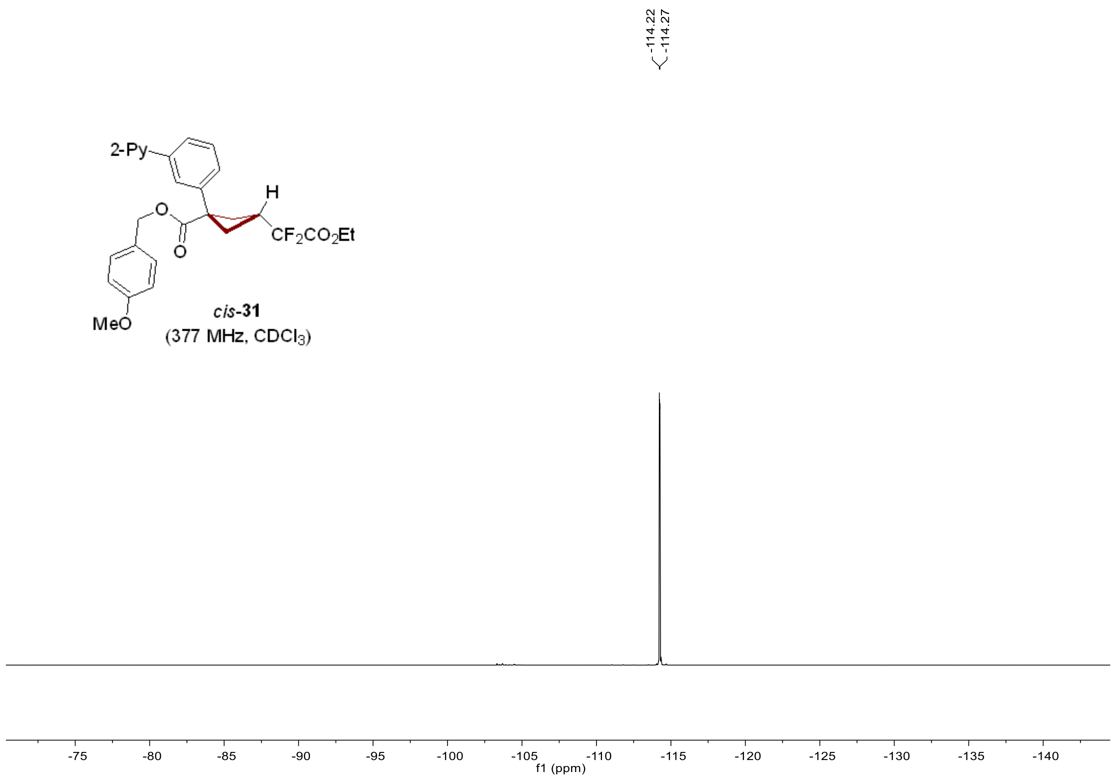
-114.22
-114.27
2-Py
H
O
CF2CO2Et
O
MeO
cis-31
(377 MHz, CDCl3)
-75 -80 -85 -90 -95 -100 -105 -110 -115 -120 -125 -130 -135 -140
f1 (ppm)

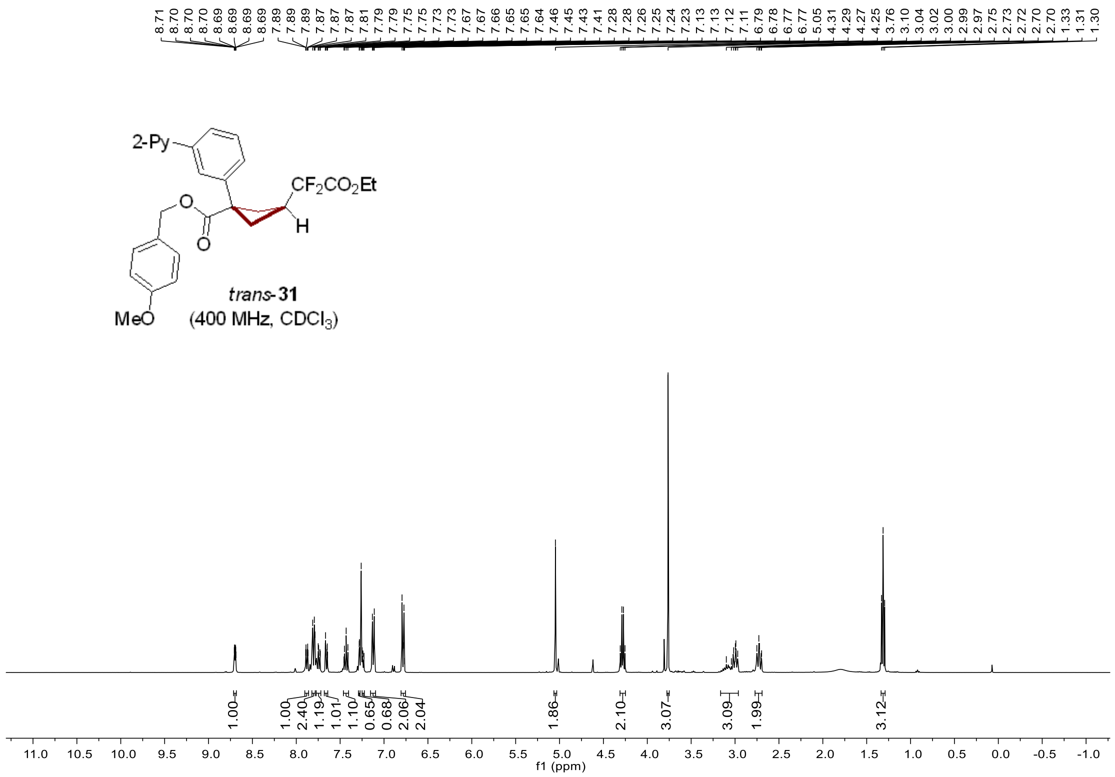
8.71 8.70 8.70 8.70 8.69 8.69 8.69 8.69 7.89 7.89 7.87 7.87 7.87 7.81 7.79 7.79 7.75 7.75 7.73 7.73 7.67 7.67 7.66 7.65 7.65 7.64 7.46 7.45 7.43 7.41 7.28 7.26 7.25 7.25 7.24 7.23 7.13 7.13 7.12 7.11 6.79 6.78 6.77 6.77 5.05 4.31 4.29 4.27 4.25 3.76 3.10 3.04 3.02 3.00 2.99 2.97 2.75 2.73 2.72 2.70 1.33 1.31 1.30
2-Py
CF2CO2Et
O
H
O
MeO
trans-31
(400 MHz, CDCl3)
1.00 1.00 2.40 1.19 1.01 1.10 0.65 0.68 2.06 2.04 1.86 2.10 3.07 3.09 1.99 3.12
11.0 10.5 10.0 9.5 9.0 8.5 8.0 7.5 7.0 6.5 6.0 5.5 5.0 4.5 4.0 3.5 3.0 2.5 2.0 1.5 1.0 0.5 0.0 -0.5 -1.0
f1 (ppm)

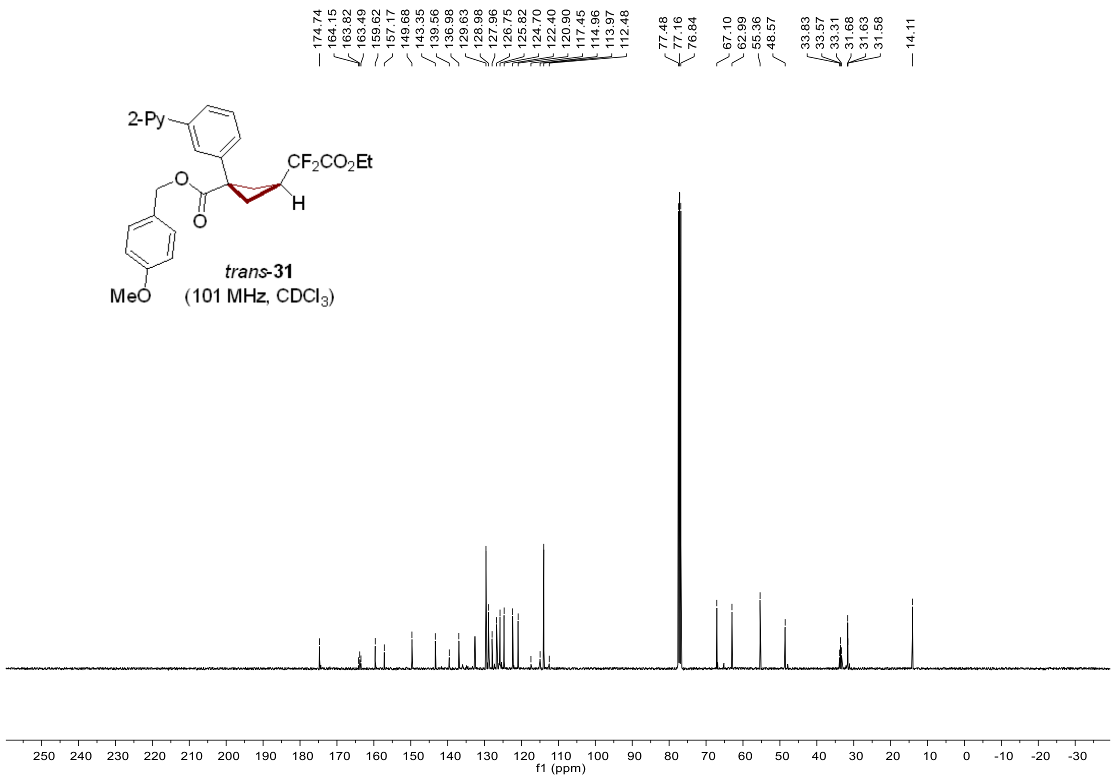
174.74
164.15
163.82
163.49
159.62
157.17
149.68
143.35
139.56
136.98
129.63
128.98
127.96
126.75
125.82
124.70
122.40
120.90
117.45
114.96
113.97
112.48
77.48
77.16
76.84
67.10
62.99
55.36
48.57
33.83
33.57
33.31
31.68
31.63
31.58
14.11
2-Py
$CF_2CO_2Et$
O
H
O
MeO
*trans*-**31**
(101 MHz, $CDCl_3$)
250 240 230 220 210 200 190 180 170 160 150 140 130 120 110 100 90 80 70 60 50 40 30 20 10 0 -10 -20 -30
f1 (ppm)

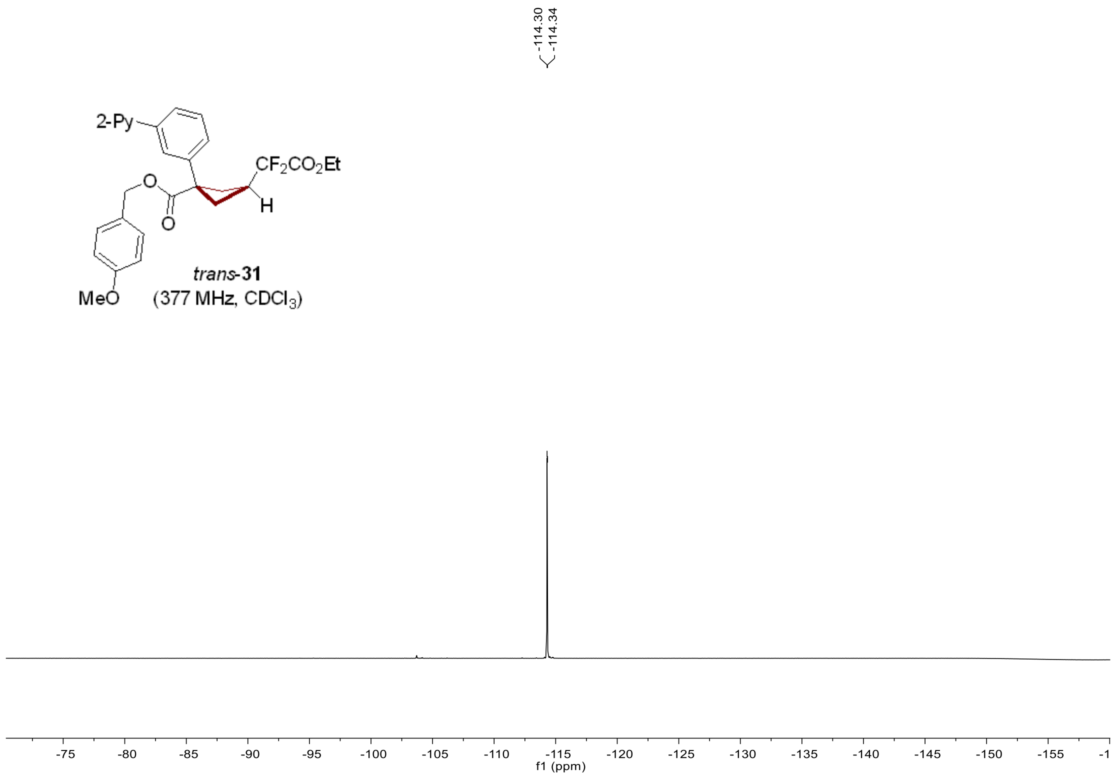
-114.30
-114.34
2-Py
$CF_2CO_2Et$
O
H
O
MeO
*trans*-**31**
(377 MHz, $CDCl_3$)
-75 -80 -85 -90 -95 -100 -105 -110 -115 -120 -125 -130 -135 -140 -145 -150 -155 -1
f1 (ppm)

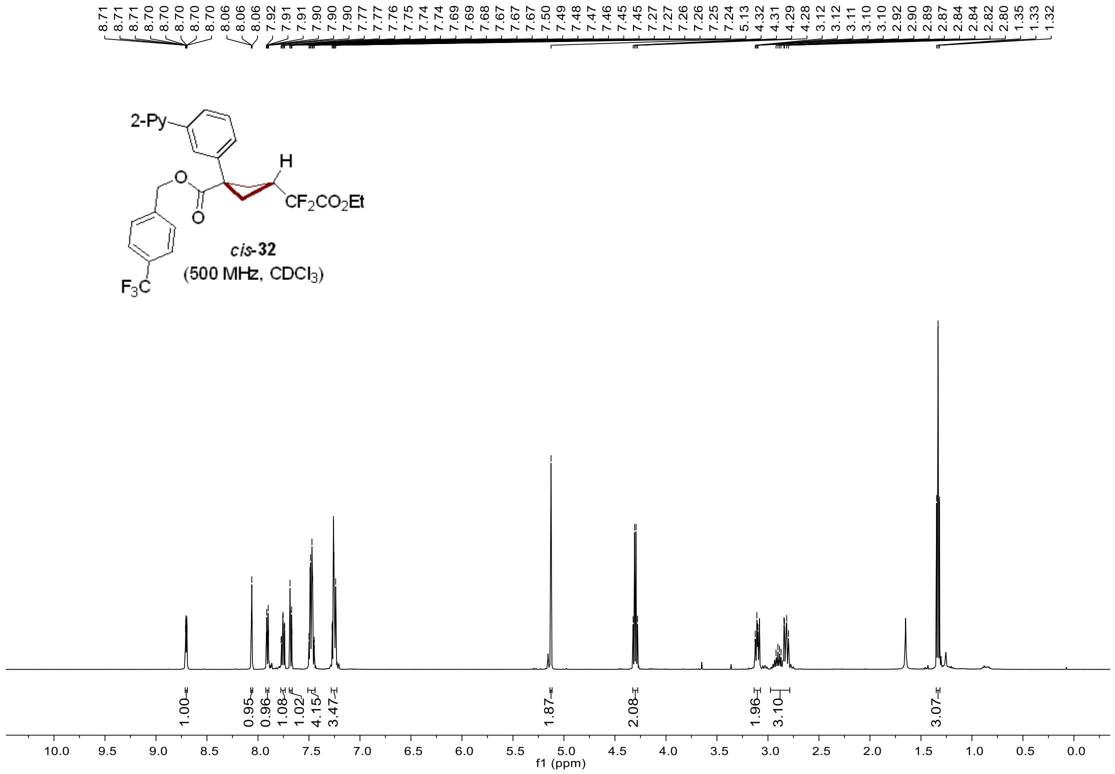
8.71 8.71 8.71 8.70 8.70 8.70 8.70 8.06 8.06 7.92 7.91 7.91 7.90 7.90 7.90 7.77 7.77 7.76 7.75 7.74 7.74 7.69 7.69 7.68 7.67 7.67 7.50 7.49 7.48 7.47 7.46 7.45 7.45 7.27 7.27 7.26 7.26 7.25 7.24 5.13 4.32 4.31 4.29 4.28 3.12 3.12 3.11 3.10 3.10 2.92 2.90 2.89 2.87 2.84 2.84 2.82 2.80 1.35 1.33 1.32
2-Py
O
H
$CF_2CO_2Et$
$F_3C$
*cis*-**32**
(500 MHz, $CDCl_3$)
1.00 0.95 0.96 1.08 1.02 4.15 3.47 1.87 2.08 1.96 3.10 3.07
10.0 9.5 9.0 8.5 8.0 7.5 7.0 6.5 6.0 5.5 5.0 4.5 4.0 3.5 3.0 2.5 2.0 1.5 1.0 0.5 0.0
f1 (ppm)

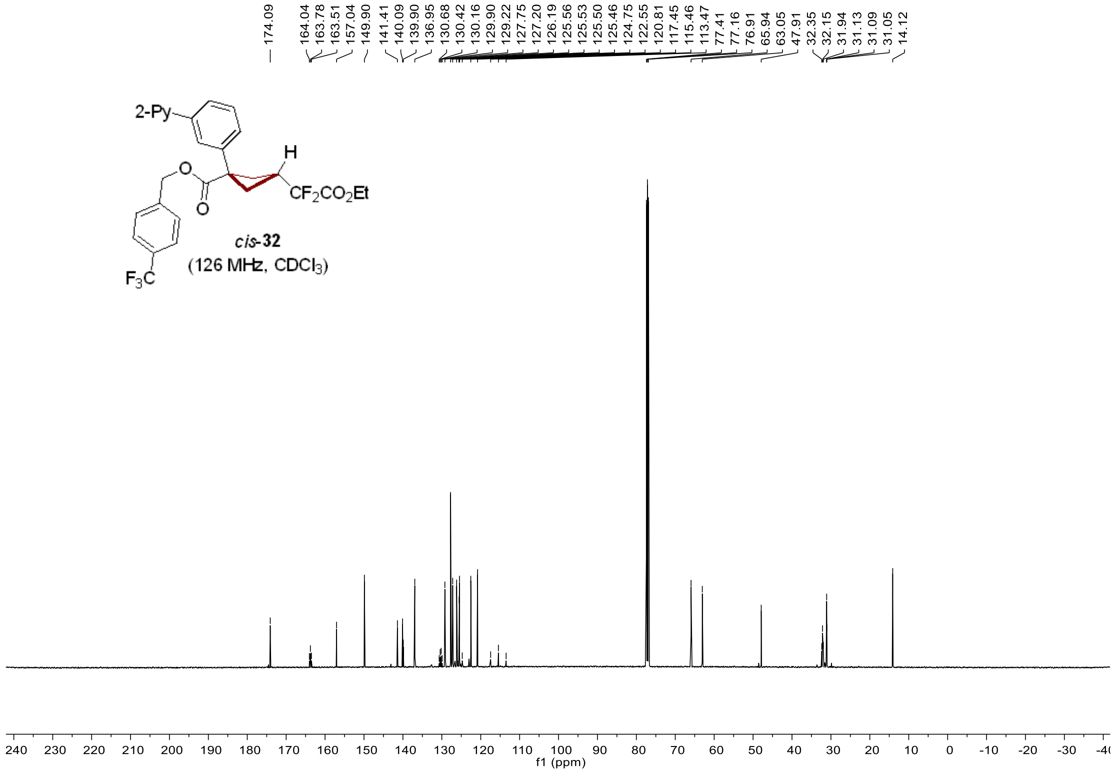
174.09 164.04 163.78 163.51 157.04 149.90 141.41 140.09 139.90 136.95 130.68 130.42 130.16 129.90 129.22 127.75 127.20 126.19 125.56 125.53 125.50 125.46 124.75 122.55 120.81 117.45 115.46 113.47 77.41 77.16 76.91 65.94 63.05 47.91 32.35 32.15 31.94 31.13 31.09 31.05 14.12
2-Py
O
H
$CF_2CO_2Et$
$F_3C$
*cis*-**32**
(126 MHz, $CDCl_3$)
240 230 220 210 200 190 180 170 160 150 140 130 120 110 100 90 80 70 60 50 40 30 20 10 0 -10 -20 -30
f1 (ppm)

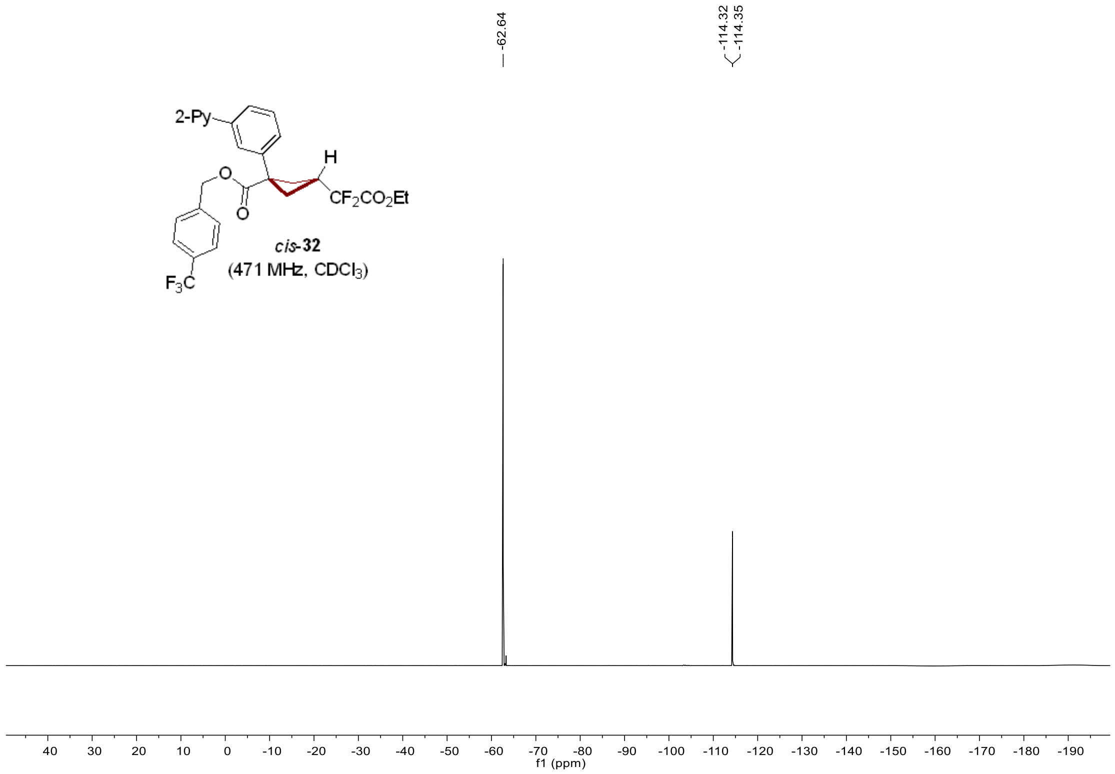
-62.64
-114.32
-114.35
2-Py
H
O
CF2CO2Et
O
cis-32
(471 MHz, CDCl3)
F3C
f1 (ppm)

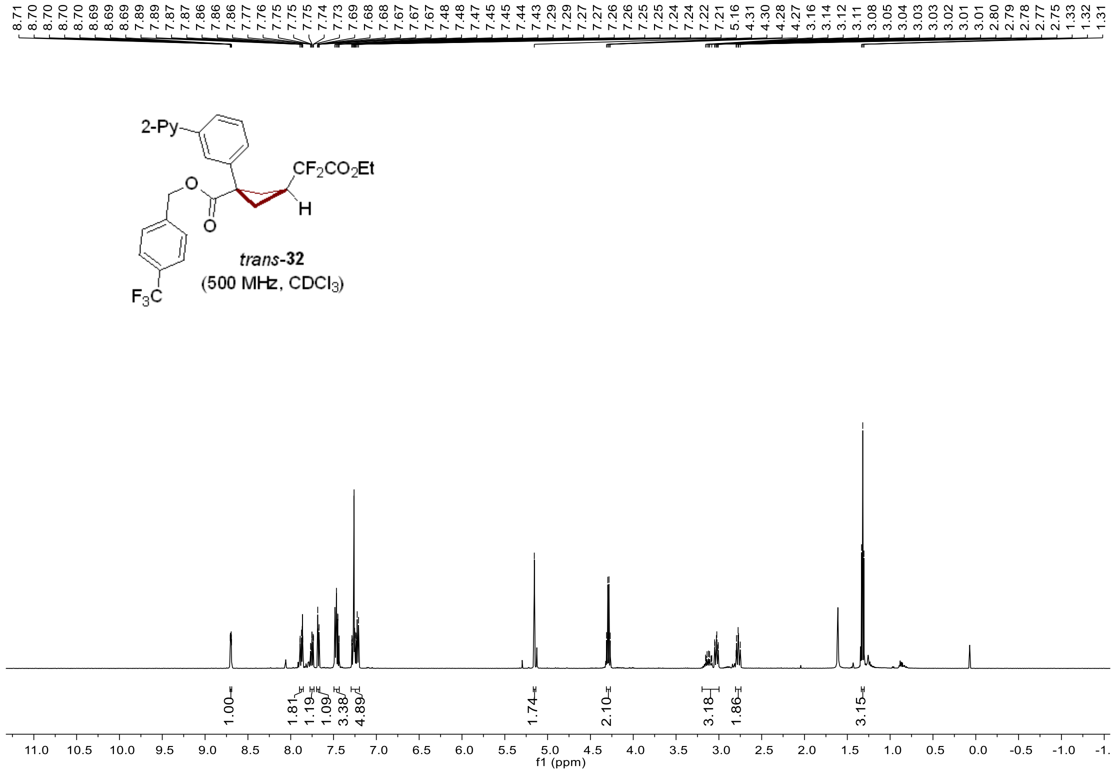
8.71 8.70 8.70 8.70 8.69 8.69 8.69 7.89 7.89 7.87 7.87 7.86 7.86 7.77 7.76 7.75 7.75 7.74 7.73 7.69 7.68 7.67 7.67 7.67 7.48 7.48 7.47 7.45 7.45 7.44 7.43 7.29 7.29 7.27 7.27 7.26 7.26 7.25 7.25 7.24 7.24 7.22 7.21 5.16 4.31 4.30 4.28 4.27 3.16 3.14 3.12 3.11 3.08 3.05 3.04 3.03 3.03 3.02 3.01 2.80 2.79 2.78 2.77 2.75 1.33 1.32 1.31
2-Py
CF2CO2Et
O
H
O
trans-32
(500 MHz, CDCl3)
F3C
1.00
1.81
1.19
1.09
3.38
4.89
1.74
2.10
3.18
1.86
3.15
f1 (ppm)

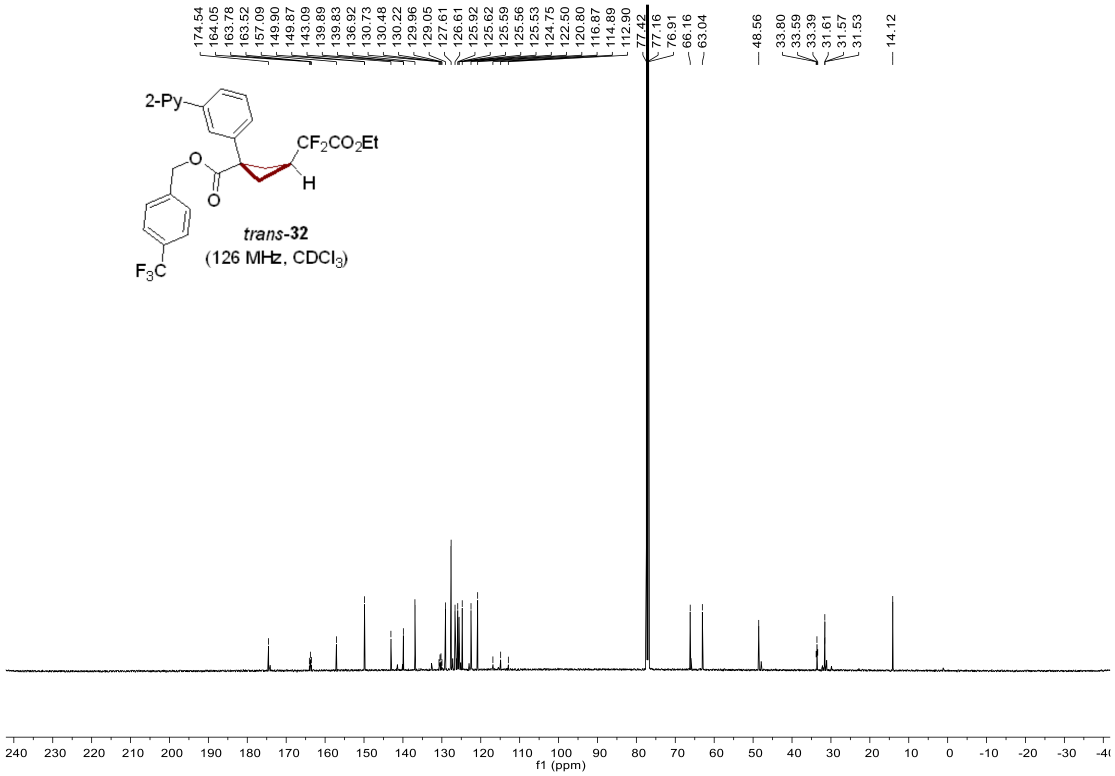

174.54
164.05
163.78
163.52
157.09
149.90
149.87
143.09
139.89
139.83
136.92
130.73
130.48
130.22
129.96
129.05
127.61
126.61
125.92
125.62
125.59
125.56
125.53
124.75
122.50
120.80
116.87
114.89
112.90
77.42
77.16
76.91
66.16
63.04
48.56
33.80
33.59
33.39
31.61
31.57
31.53
14.12
2-Py
CF2CO2Et
O
O
H
F3C
trans-32
(126 MHz, CDCl3)
240 230 220 210 200 190 180 170 160 150 140 130 120 110 100 90 80 70 60 50 40 30 20 10 0 -10 -20 -30
f1 (ppm)


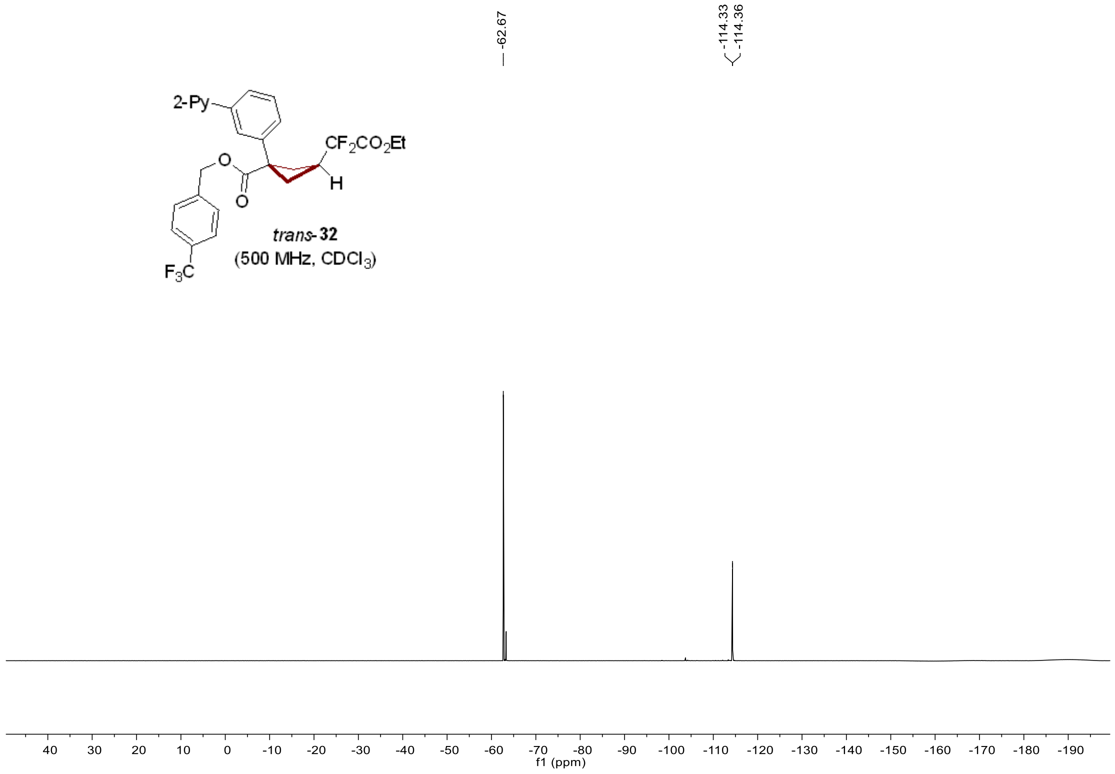

-62.67
-114.33
-114.36
2-Py
CF2CO2Et
O
O
H
F3C
trans-32
(500 MHz, CDCl3)
40 30 20 10 0 -10 -20 -30 -40 -50 -60 -70 -80 -90 -100 -110 -120 -130 -140 -150 -160 -170 -180 -190
f1 (ppm)

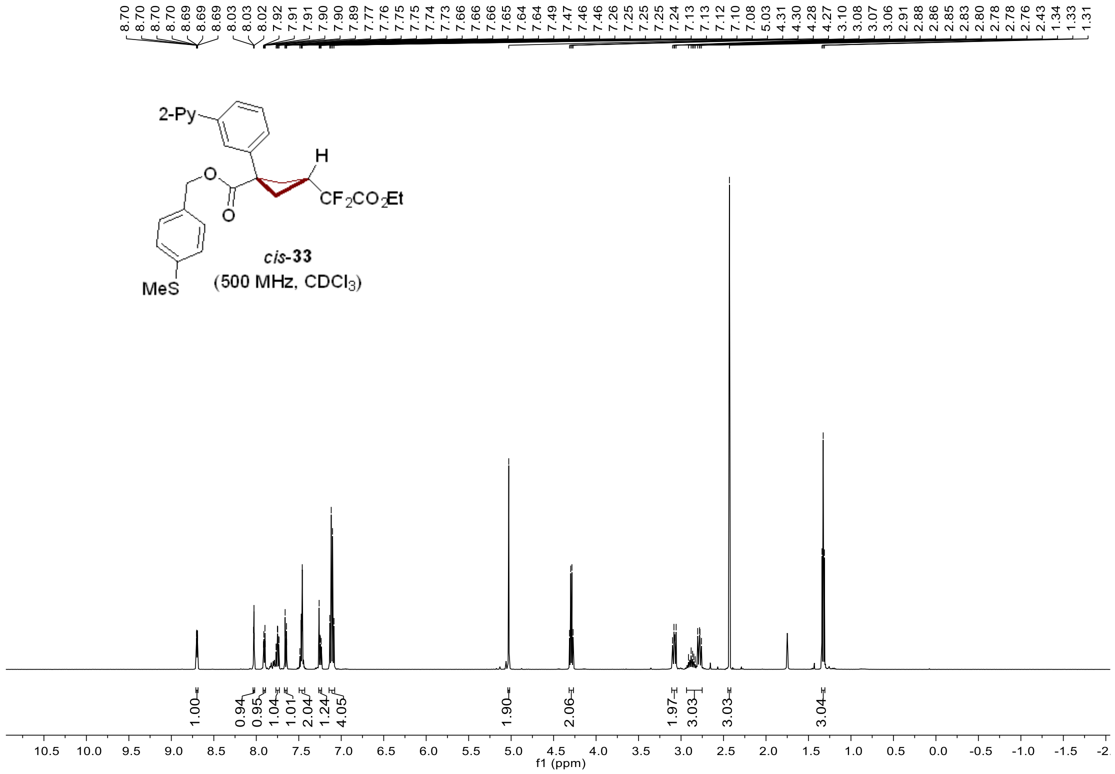

8.70 8.70 8.70 8.69 8.69 8.69 8.03 8.03 8.02 7.92 7.91 7.91 7.90 7.89 7.77 7.76 7.75 7.75 7.74 7.73 7.66 7.66 7.65 7.64 7.64 7.49 7.47 7.46 7.26 7.25 7.25 7.24 7.13 7.13 7.12 7.10 7.08 5.03 4.31 4.30 4.28 4.27 3.10 3.08 3.07 3.06 2.91 2.88 2.86 2.85 2.83 2.80 2.78 2.78 2.76 2.43 1.34 1.33 1.31
2-Py
H
O
O
CF2CO2Et
MeS
cis-33
(500 MHz, CDCl3)
1.00 0.94 0.95 1.04 1.01 2.04 1.24 4.05 1.90 2.06 1.97 3.03 3.03 3.04
f1 (ppm)


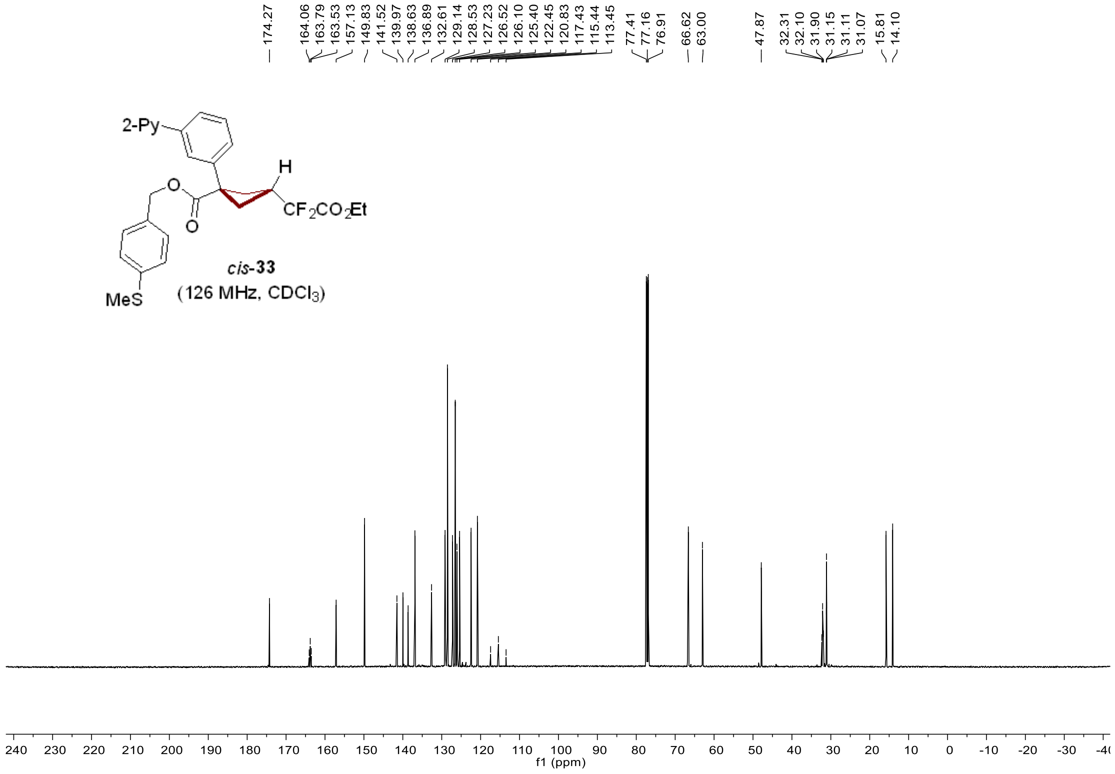

174.27 164.06 163.79 163.53 157.13 149.83 141.52 139.97 138.63 136.89 132.61 129.14 128.53 127.23 126.52 126.10 125.40 122.45 120.83 117.43 115.44 113.45 77.41 77.16 76.91 66.62 63.00 47.87 32.31 32.10 31.90 31.15 31.11 31.07 15.81 14.10
2-Py
H
O
O
CF2CO2Et
MeS
cis-33
(126 MHz, CDCl3)
f1 (ppm)

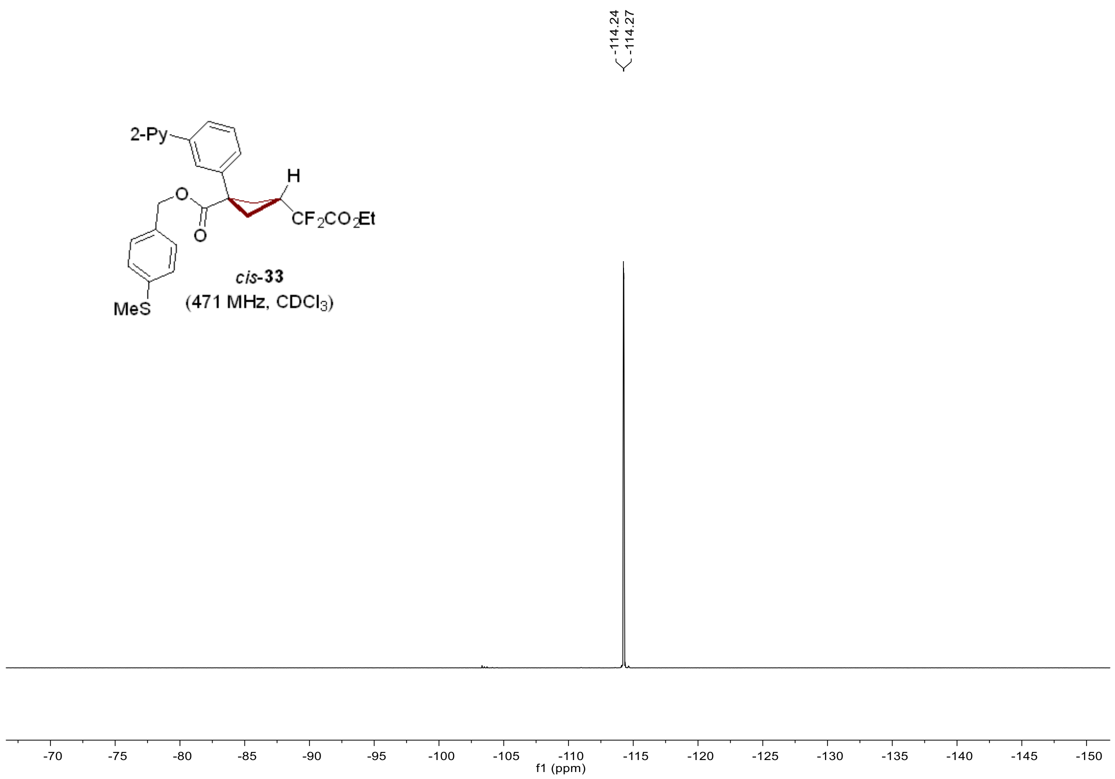
-114.24
-114.27
2-Py
H
$CF_2CO_2Et$
O
O
MeS
*cis*-**33**
(471 MHz, $CDCl_3$)
f1 (ppm)

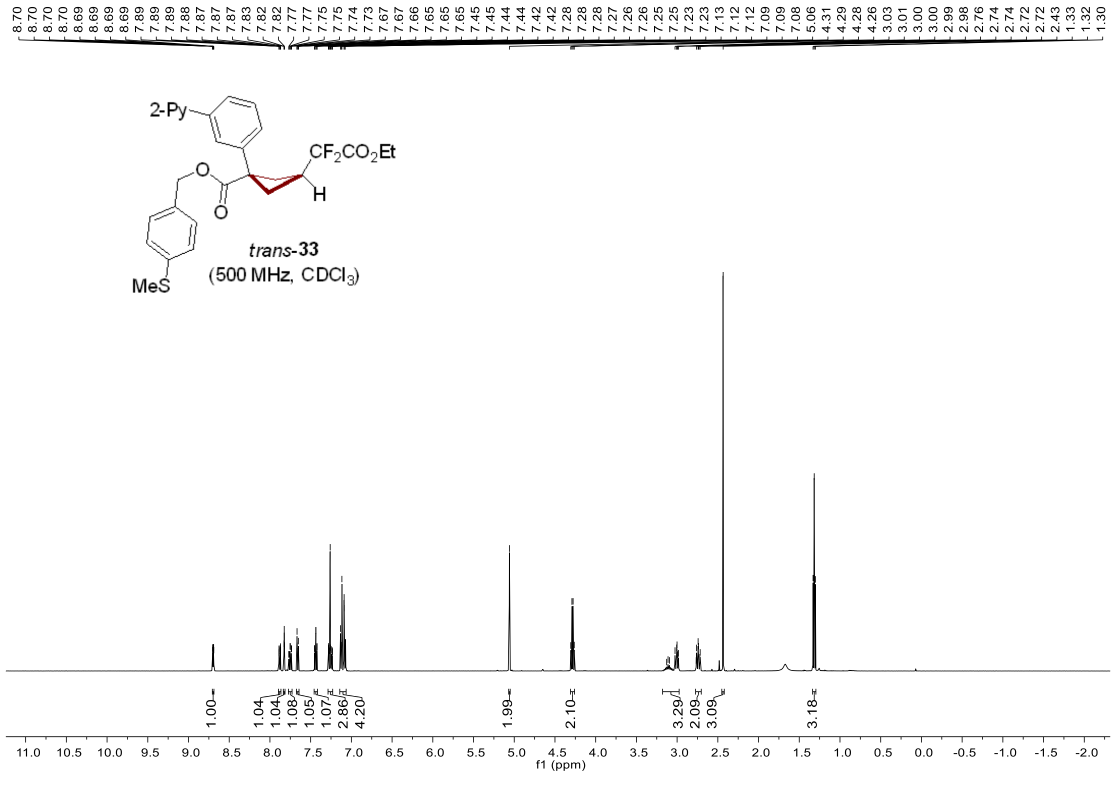
2-Py
$CF_2CO_2Et$
H
O
O
MeS
*trans*-**33**
(500 MHz, $CDCl_3$)
f1 (ppm)

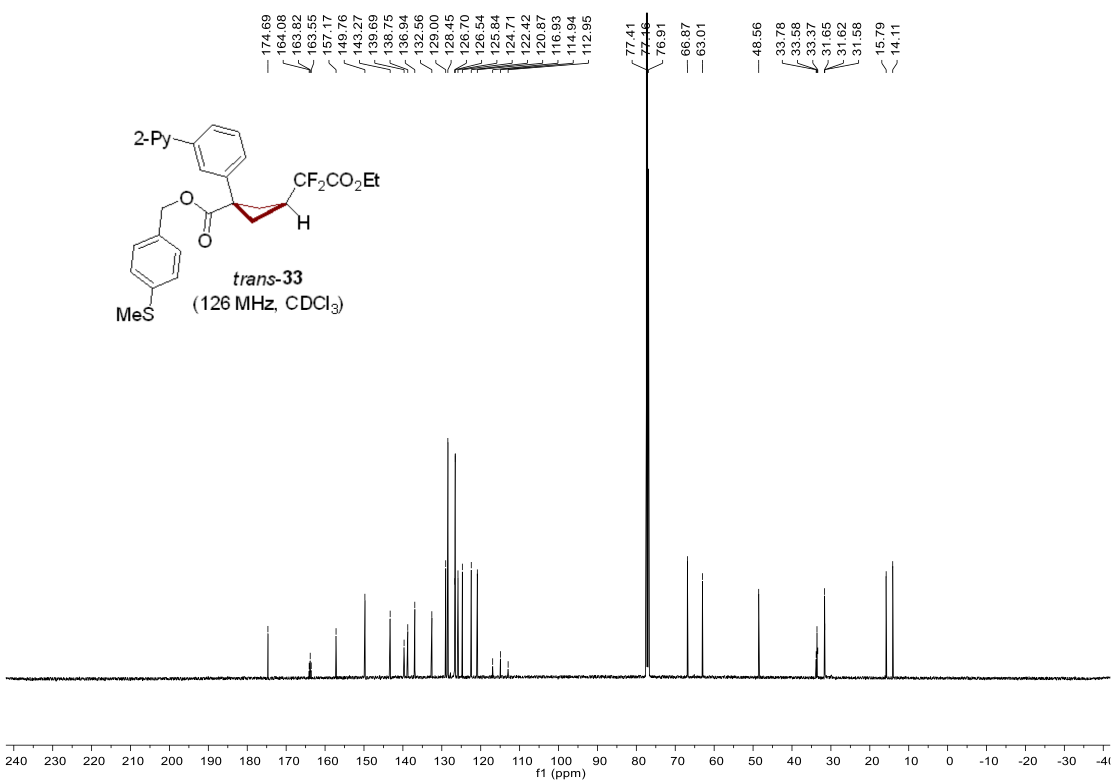
174.69
164.08
163.82
163.55
157.17
149.76
143.27
139.69
138.75
136.94
132.56
129.00
128.45
126.70
126.54
125.84
124.71
122.42
120.87
116.93
114.94
112.95
77.41
77.16
76.91
66.87
63.01
48.56
33.78
33.58
33.37
31.65
31.62
31.58
15.79
14.11
2-Py
$CF_2CO_2Et$
O
O
H
MeS
*trans*-**33**
(126 MHz, $CDCl_3$)
f1 (ppm)

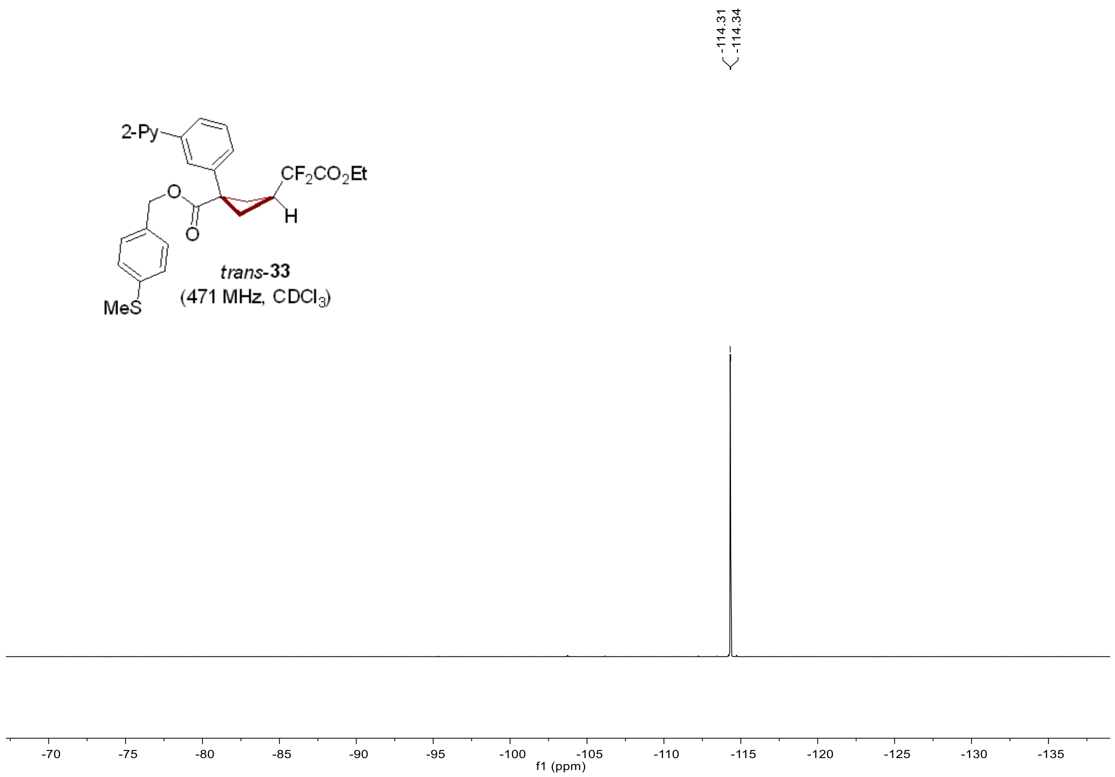
-114.31
-114.34
2-Py
$CF_2CO_2Et$
O
O
H
MeS
*trans*-**33**
(471 MHz, $CDCl_3$)
f1 (ppm)

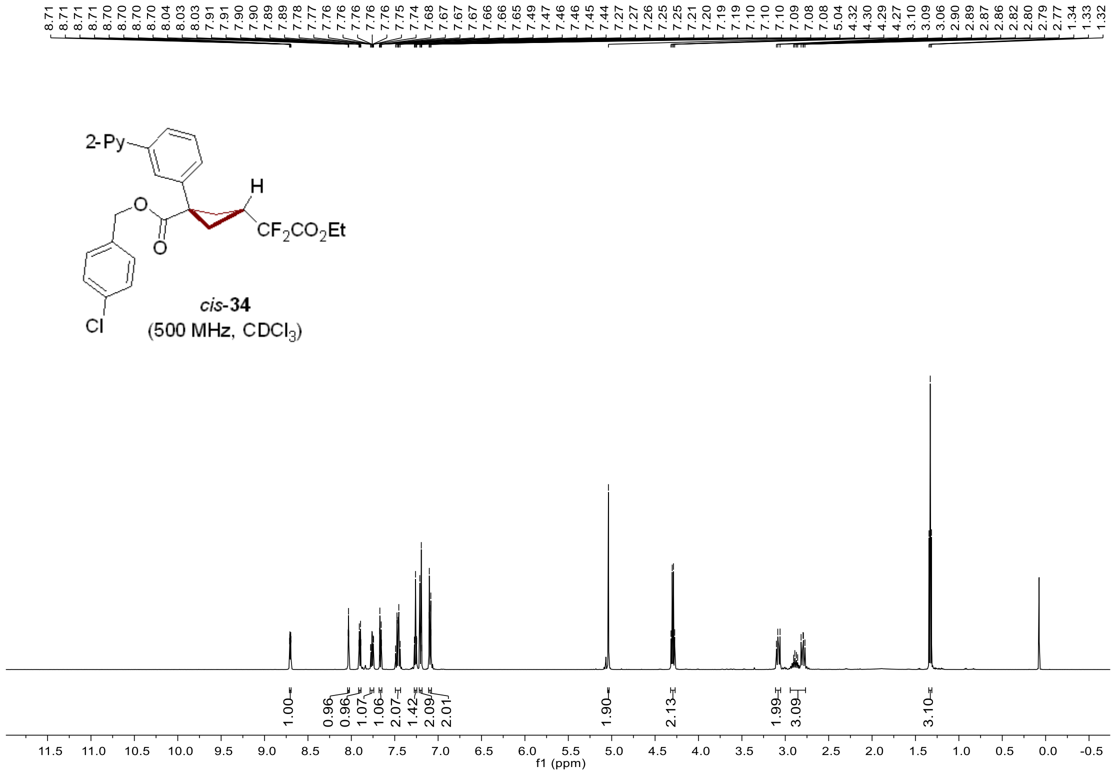
8.71 8.71 8.71 8.70 8.70 8.70 8.04 8.03 7.91 7.91 7.90 7.89 7.78 7.77 7.76 7.76 7.76 7.76 7.75 7.74 7.68 7.67 7.67 7.66 7.65 7.49 7.47 7.46 7.46 7.45 7.44 7.27 7.27 7.26 7.25 7.25 7.21 7.20 7.19 7.19 7.10 7.10 7.09 7.09 7.08 5.04 4.32 4.30 4.29 4.27 3.10 3.09 3.06 2.90 2.89 2.87 2.86 2.82 2.80 2.79 2.77 1.34 1.33 1.32
2-Py
H
O
$CF_2CO_2Et$
Cl
*cis*-**34**
(500 MHz, $CDCl_3$)
1.00 0.96 0.96 1.07 1.06 2.07 1.42 2.09 2.01 1.90 2.13 1.99 3.09 3.10
f1 (ppm)

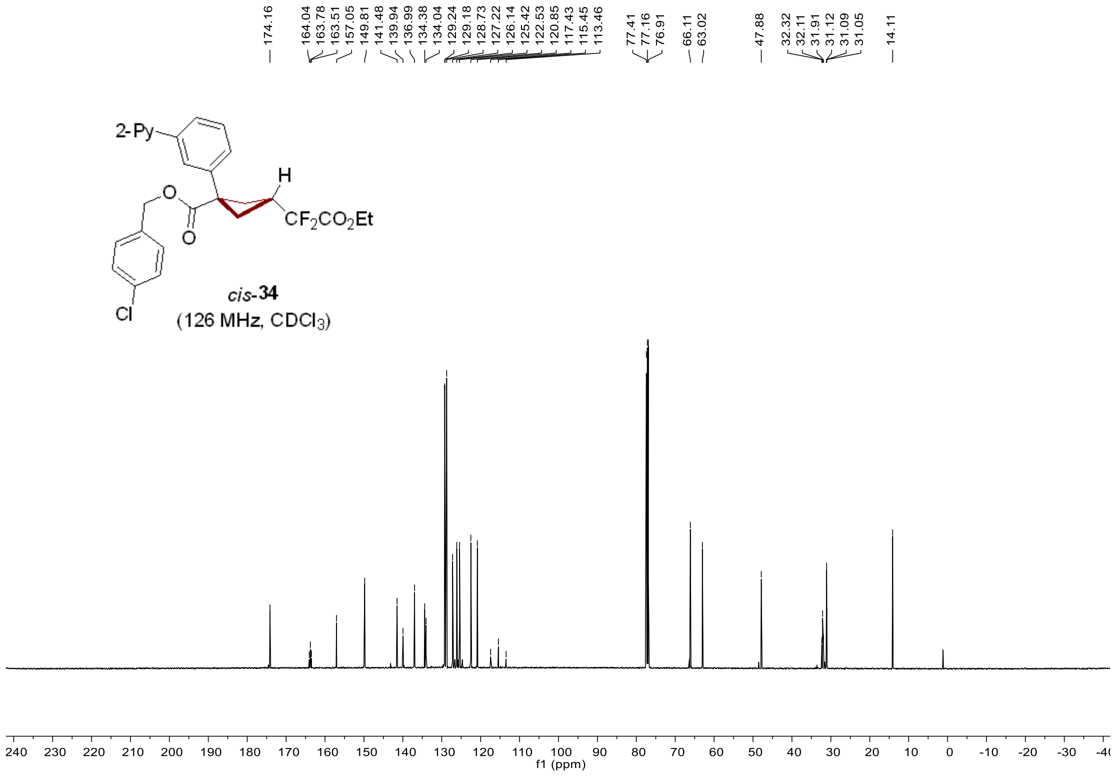
174.16 164.04 163.78 163.51 157.05 149.81 141.48 139.94 136.99 134.38 134.04 129.24 129.18 128.73 127.22 126.14 125.42 122.53 120.85 117.43 115.45 113.46 77.41 77.16 76.91 66.11 63.02 47.88 32.32 32.11 31.91 31.12 31.09 31.05 14.11
2-Py
H
O
$CF_2CO_2Et$
Cl
*cis*-**34**
(126 MHz, $CDCl_3$)
f1 (ppm)

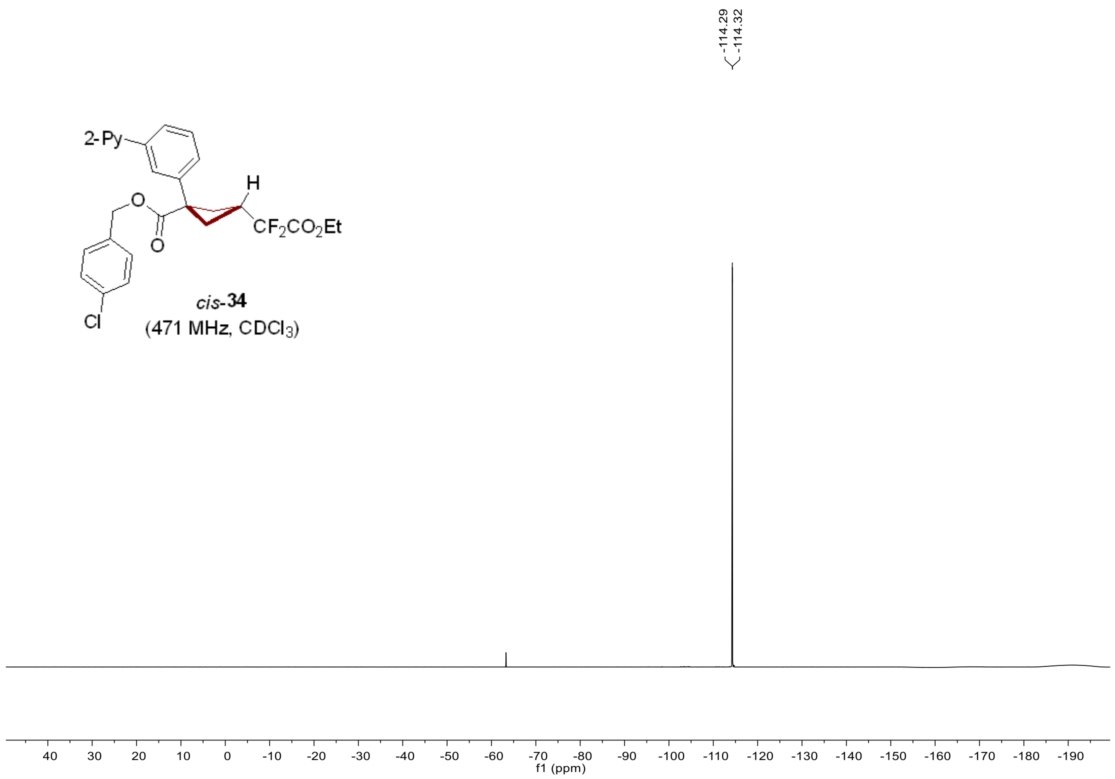
-114.29
-114.32
2-Py
H
O
CF2CO2Et
Cl
cis-34
(471 MHz, CDCl3)
f1 (ppm)

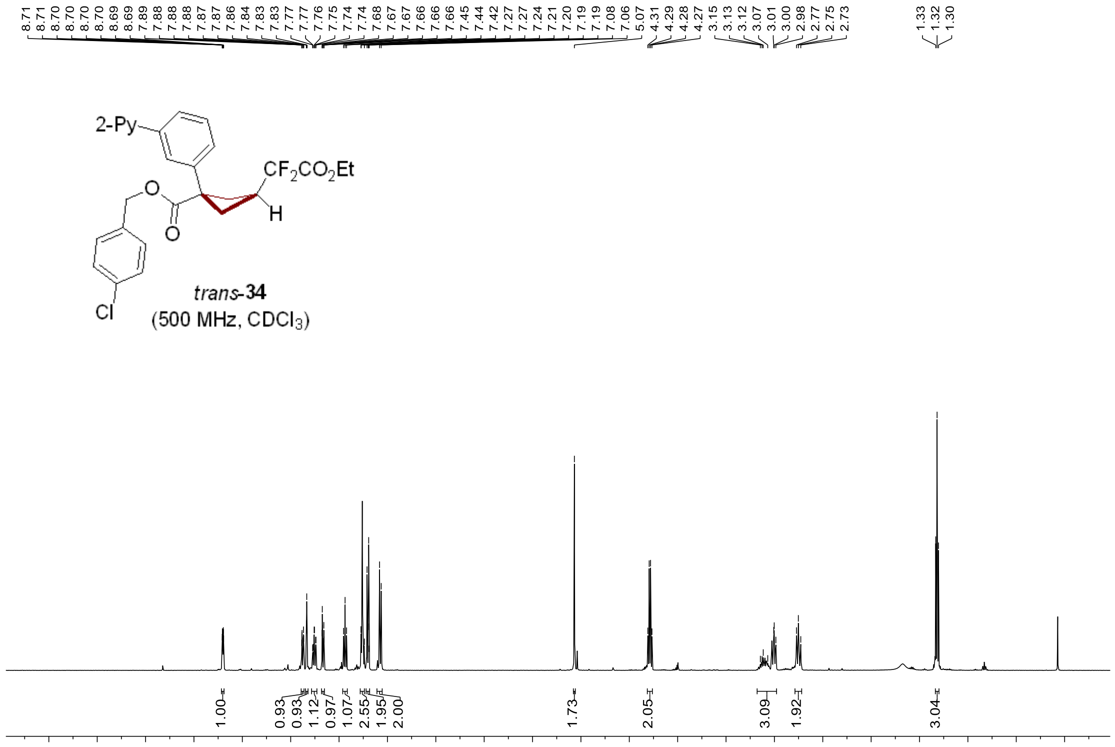
8.71 8.71 8.70 8.70 8.70 8.69 7.89 7.88 7.88 7.87 7.86 7.84 7.83 7.77 7.76 7.75 7.74 7.68 7.67 7.66 7.45 7.44 7.42 7.27 7.24 7.21 7.20 7.19 7.08 7.06 5.07 4.31 4.29 4.28 4.27 3.15 3.13 3.12 3.07 3.01 3.00 2.98 2.77 2.75 2.73 1.33 1.32 1.30
2-Py
CF2CO2Et
O
H
Cl
trans-34
(500 MHz, CDCl3)
1.00 0.93 0.93 1.12 0.97 1.07 2.55 1.95 2.00 1.73 2.05 3.09 1.92 3.04
f1 (ppm)

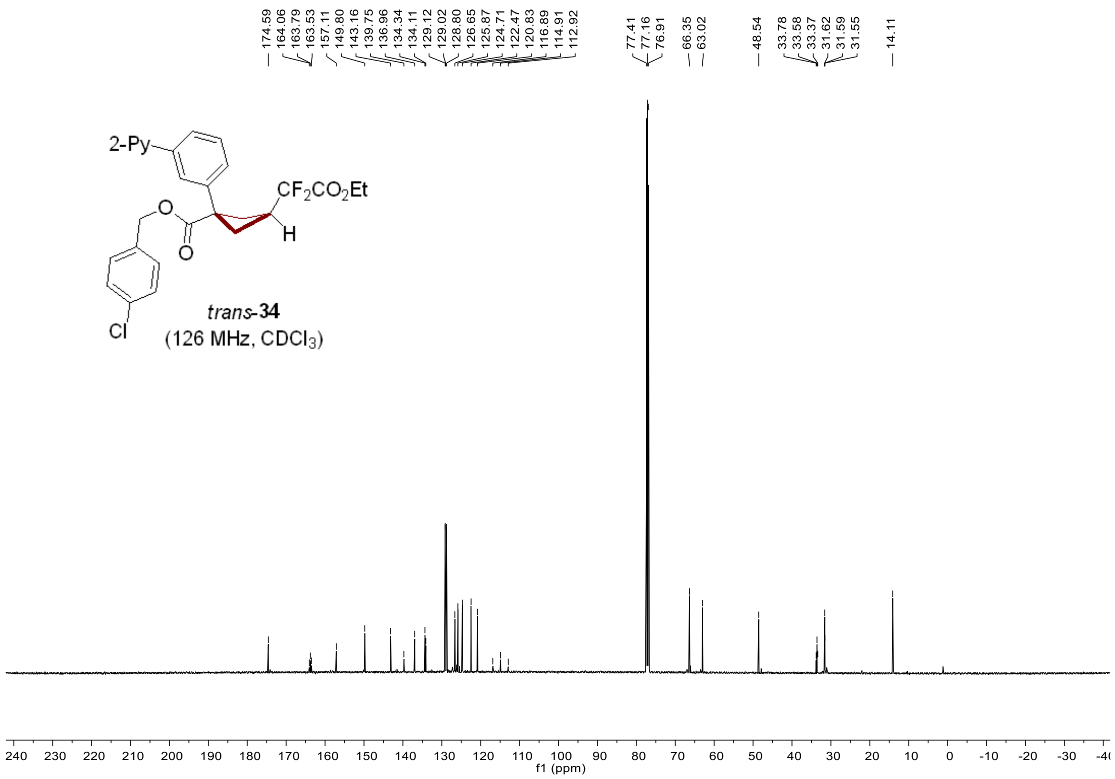
174.59
164.06
163.79
163.53
157.11
149.80
143.16
139.75
136.96
134.34
134.11
129.12
129.02
128.80
126.65
125.87
124.71
122.47
120.83
116.89
114.91
112.92
77.41
77.16
76.91
66.35
63.02
48.54
33.78
33.58
33.37
31.62
31.59
31.55
14.11
2-Py
CF2CO2Et
H
O
O
Cl
trans-34
(126 MHz, CDCl3)
f1 (ppm)

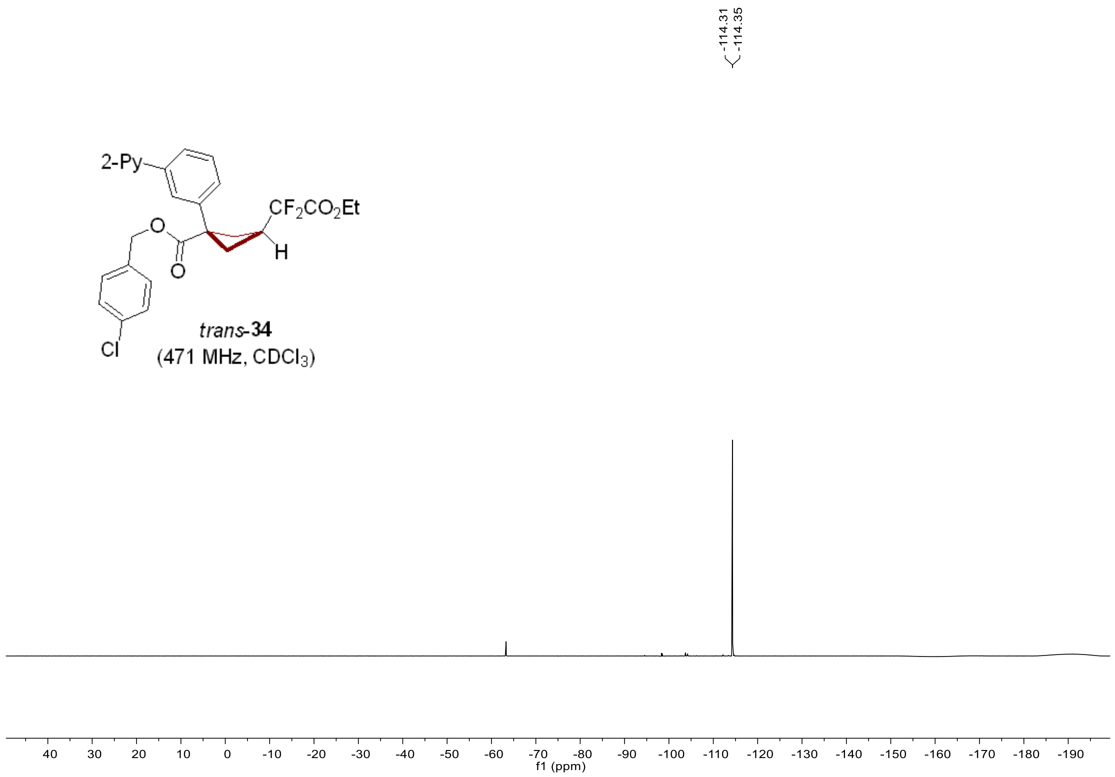
-114.31
-114.35
2-Py
CF2CO2Et
H
O
O
Cl
trans-34
(471 MHz, CDCl3)
f1 (ppm)

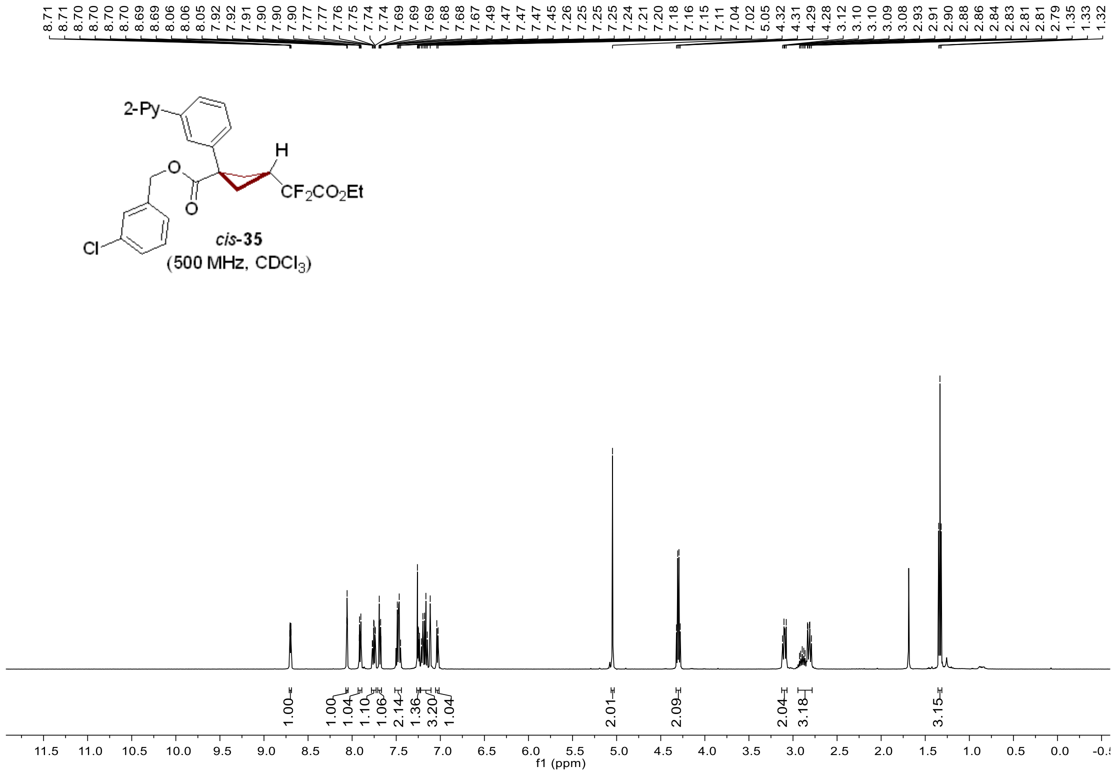
2-Py
H
O
O
CF2CO2Et
Cl
cis-35
(500 MHz, CDCl3)
f1 (ppm)

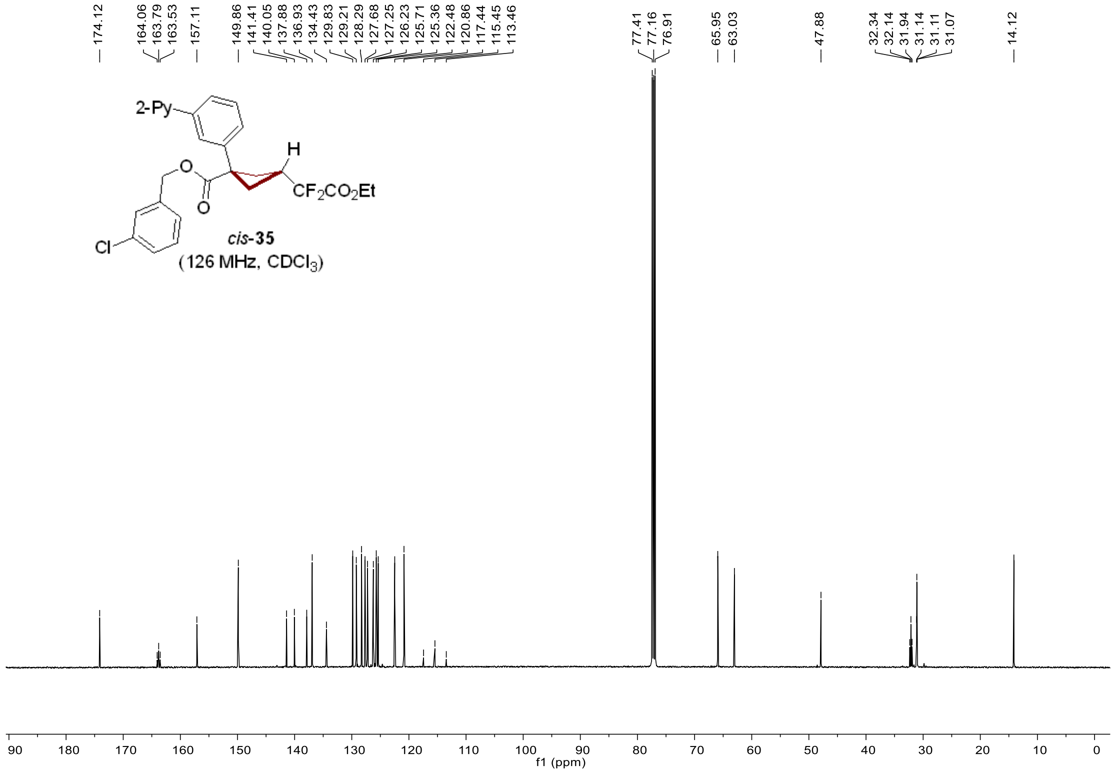
2-Py
H
O
O
CF2CO2Et
Cl
cis-35
(126 MHz, CDCl3)
f1 (ppm)

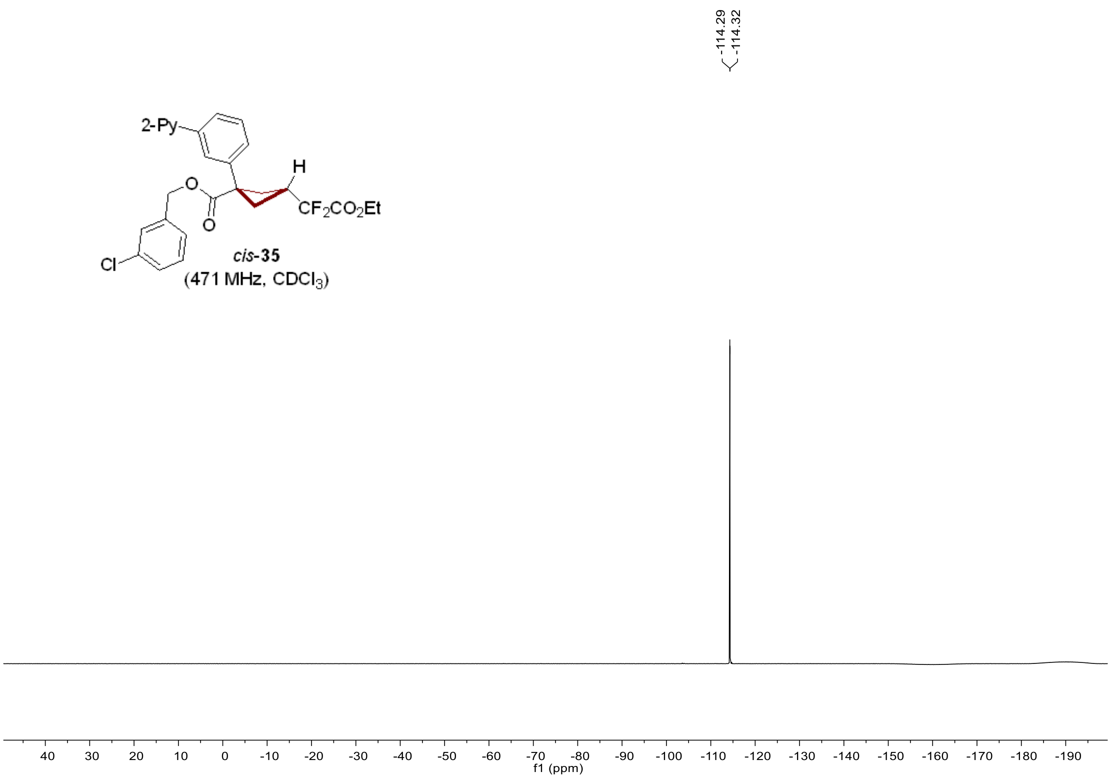
-114.29
-114.32
2-Py
H
O
CF2CO2Et
O
Cl
cis-35
(471 MHz, CDCl3)
f1 (ppm)

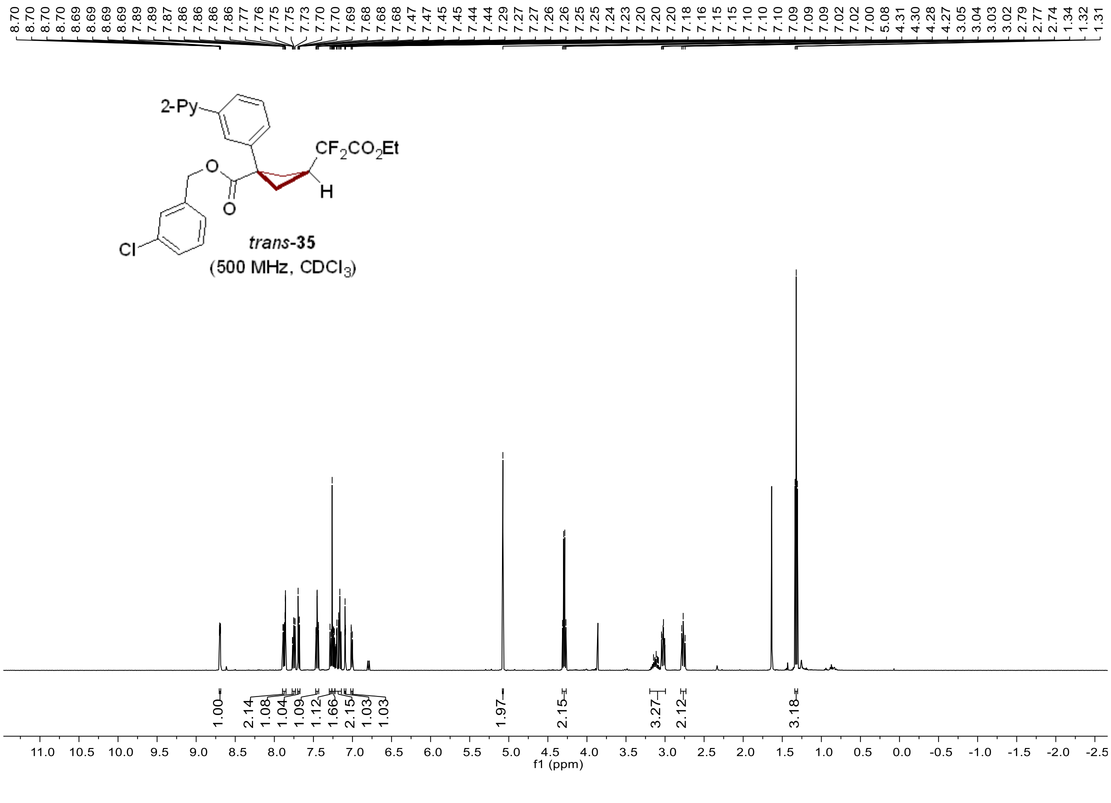
2-Py
CF2CO2Et
O
O
H
Cl
trans-35
(500 MHz, CDCl3)
1.00
2.14
1.08
1.04
1.09
1.12
1.66
2.15
1.03
1.03
1.97
2.15
3.27
2.12
3.18
f1 (ppm)

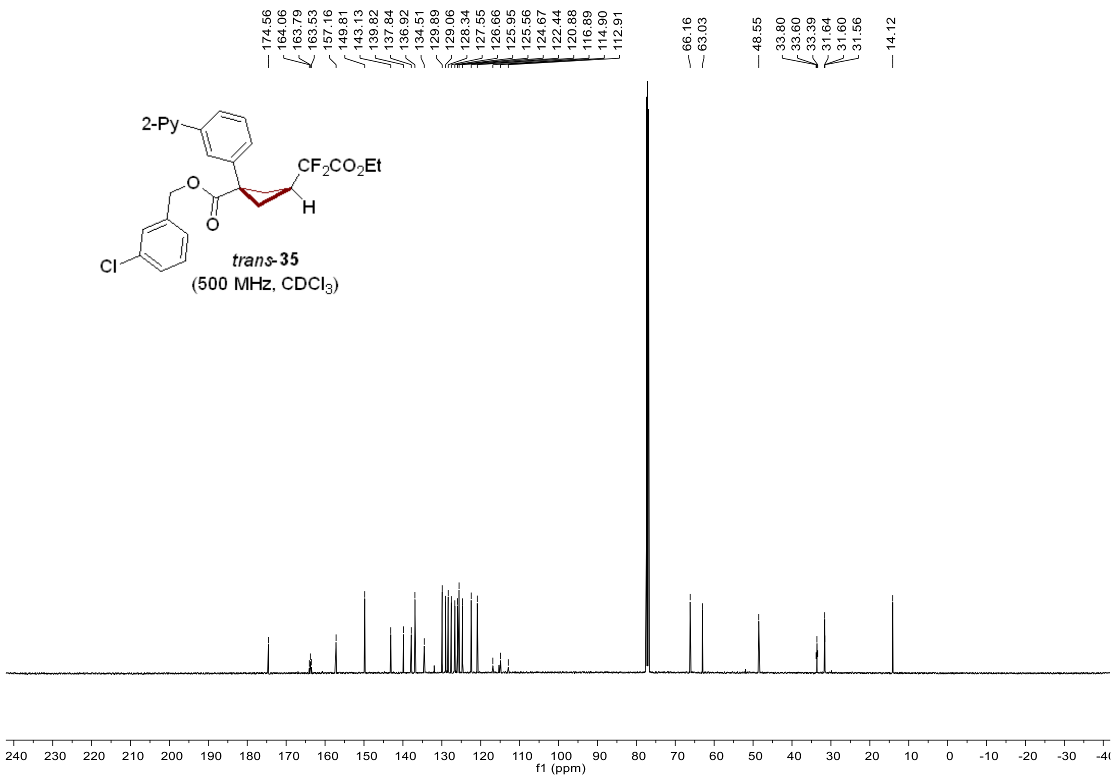
174.56
164.06
163.79
163.53
157.16
149.81
143.13
139.82
137.84
136.92
134.51
129.89
129.06
128.34
127.55
126.66
125.95
125.56
124.67
122.44
120.88
116.89
114.90
112.91
66.16
63.03
48.55
33.80
33.60
33.39
31.64
31.60
31.56
14.12
2-Py
CF2CO2Et
O
O
H
Cl
trans-35
(500 MHz, CDCl3)
f1 (ppm)

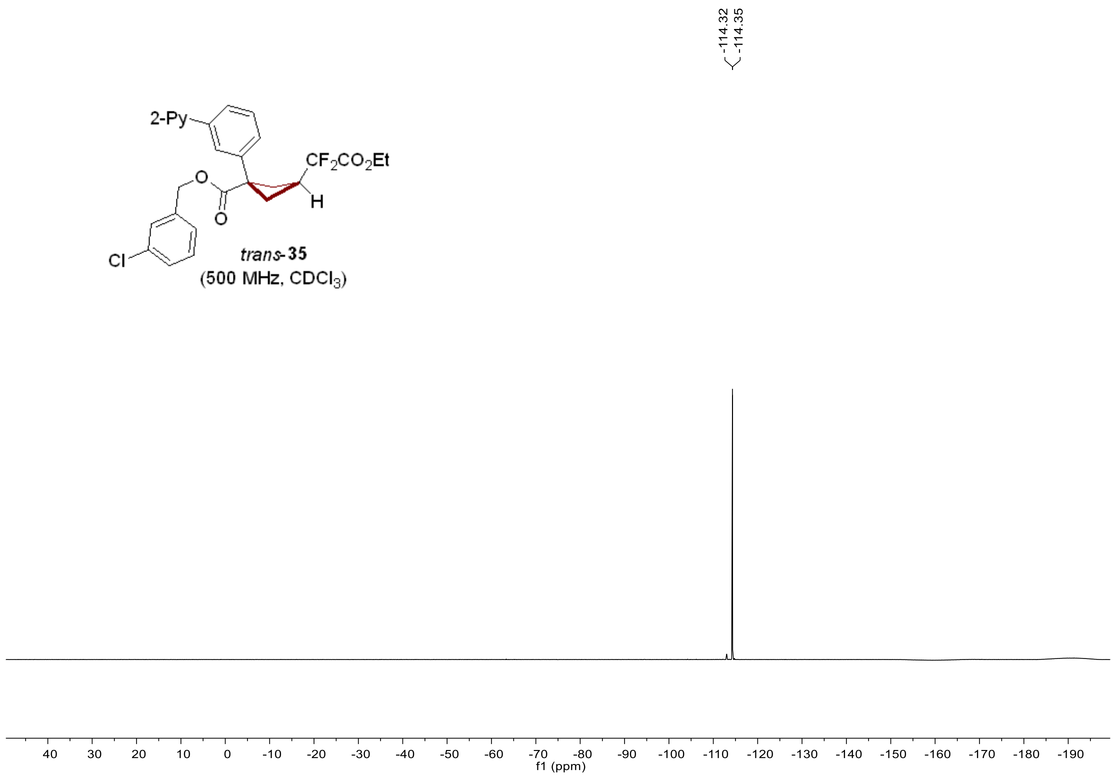
-114.32
-114.35
2-Py
CF2CO2Et
O
O
H
Cl
trans-35
(500 MHz, CDCl3)
f1 (ppm)

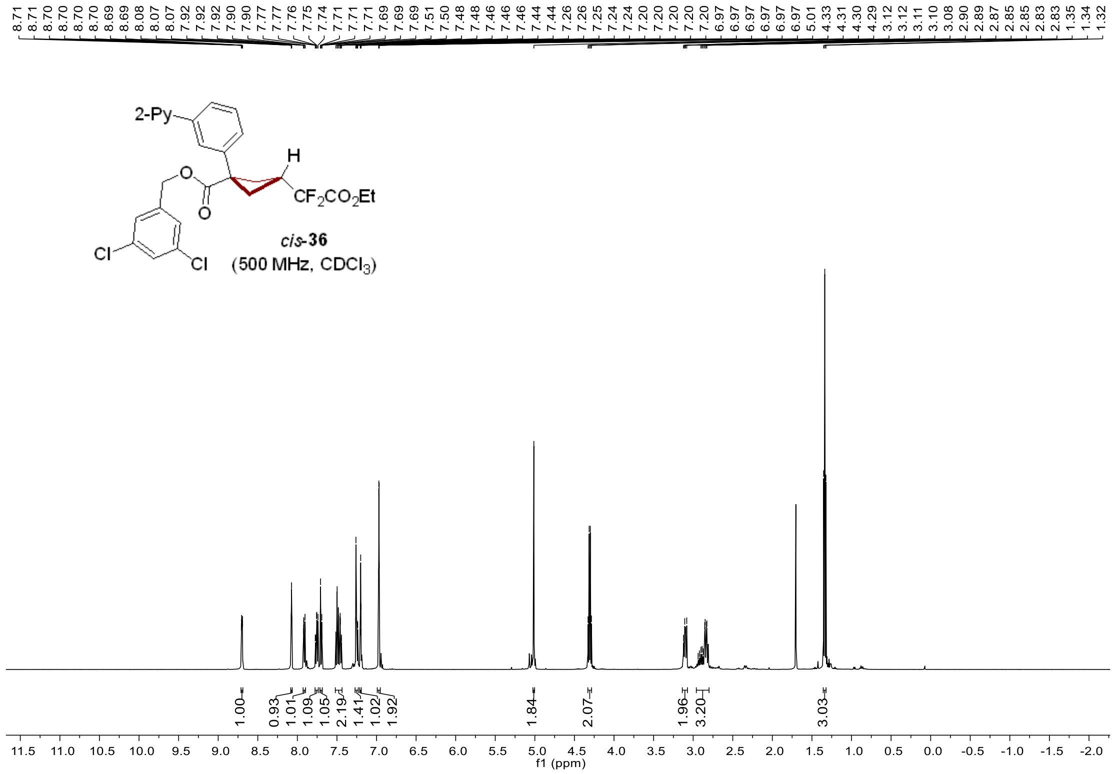

2-Py
H
CF2CO2Et
O
O
Cl
Cl
cis-36
(500 MHz, CDCl3)
f1 (ppm)


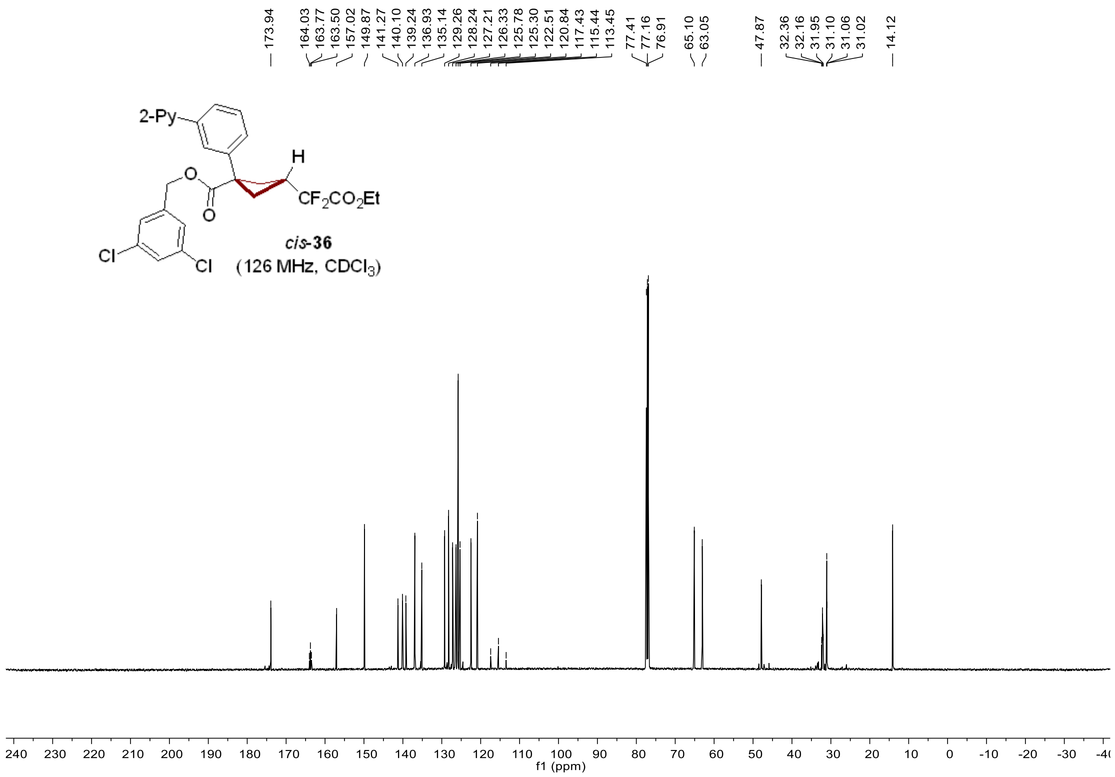

2-Py
H
CF2CO2Et
O
O
Cl
Cl
cis-36
(126 MHz, CDCl3)
f1 (ppm)

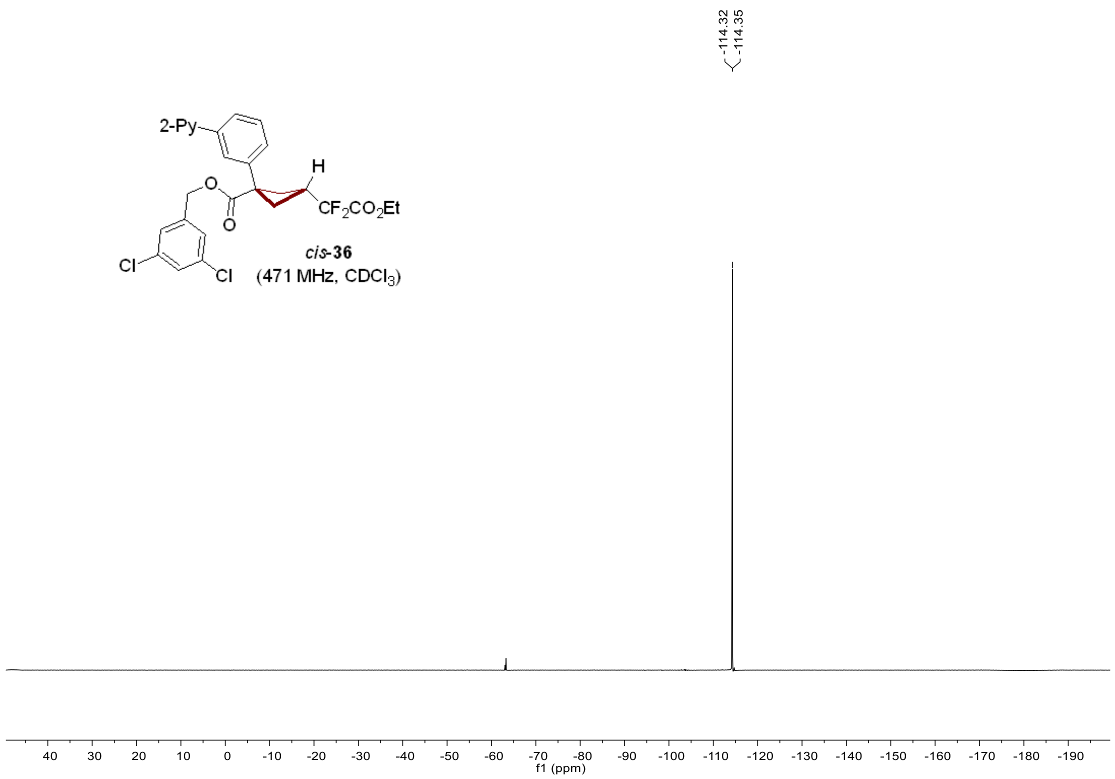

-114.32
-114.35
2-Py
H
CF2CO2Et
O
O
Cl
Cl
cis-36
(471 MHz, CDCl3)
f1 (ppm)


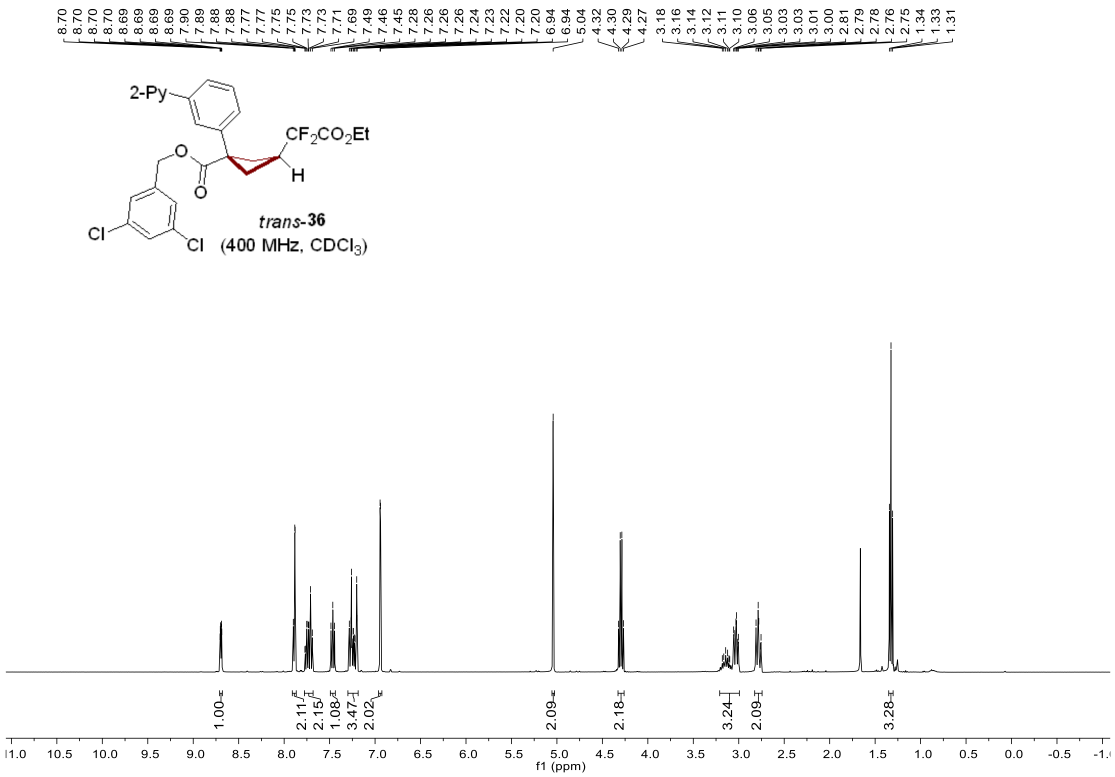

2-Py
CF2CO2Et
H
O
O
Cl
Cl
trans-36
(400 MHz, CDCl3)
1.00
2.11
2.15
1.08
3.47
2.02
2.09
2.18
3.24
2.09
3.28
f1 (ppm)

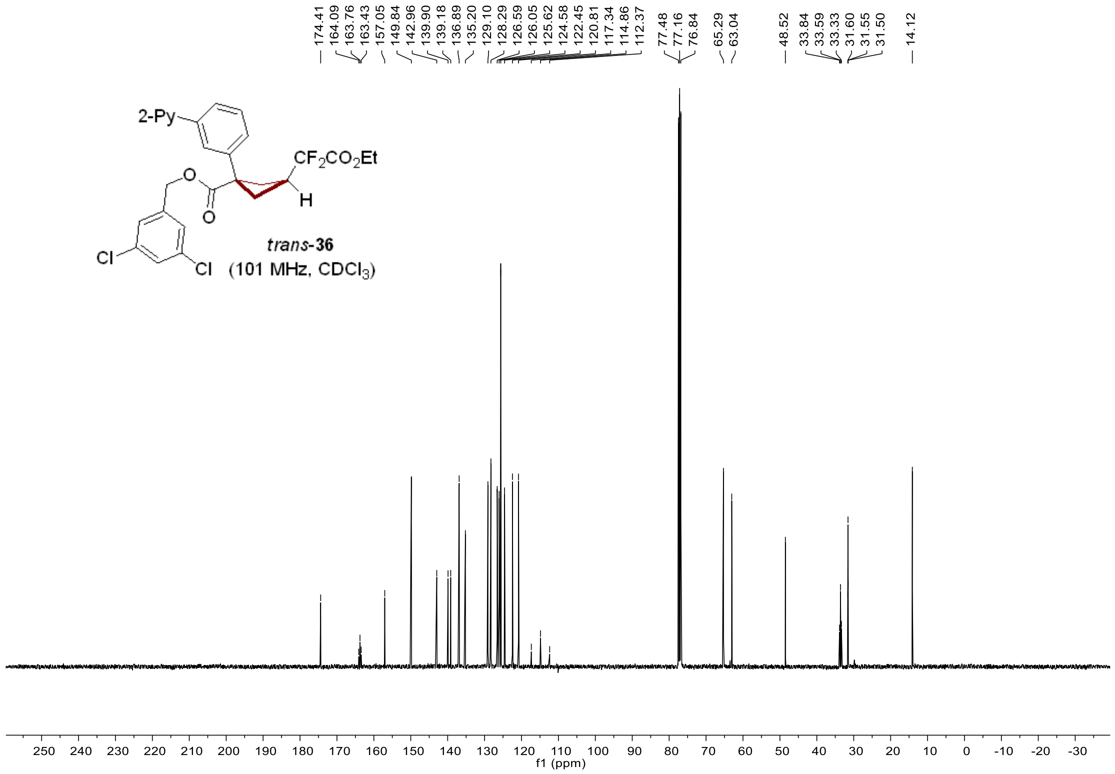
174.41
164.09
163.76
163.43
157.05
149.84
142.96
139.90
139.18
136.89
135.20
129.10
128.29
126.59
126.05
125.62
124.58
122.45
120.81
117.34
114.86
112.37
77.48
77.16
76.84
65.29
63.04
48.52
33.84
33.59
33.33
31.60
31.55
31.50
14.12
2-Py
CF2CO2Et
O
O
H
Cl
Cl
trans-36
(101 MHz, CDCl3)
250 240 230 220 210 200 190 180 170 160 150 140 130 120 110 100 90 80 70 60 50 40 30 20 10 0 -10 -20 -30
f1 (ppm)

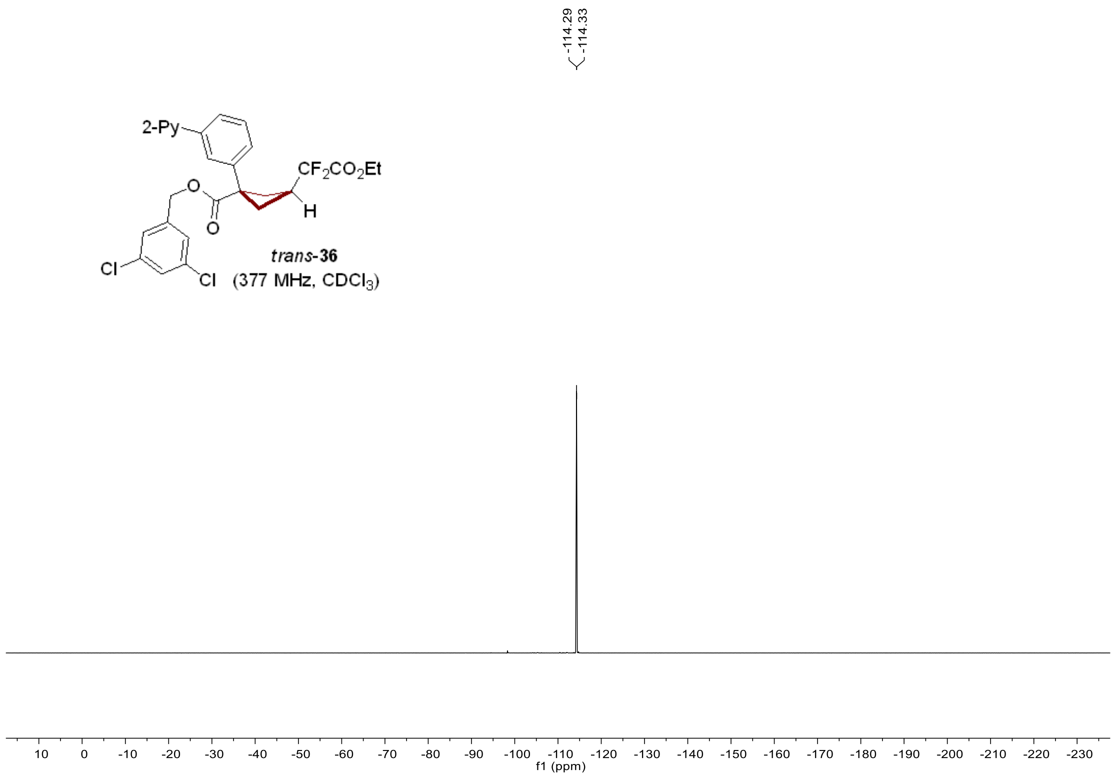
-114.29
-114.33
2-Py
CF2CO2Et
O
O
H
Cl
Cl
trans-36
(377 MHz, CDCl3)
10 0 -10 -20 -30 -40 -50 -60 -70 -80 -90 -100 -110 -120 -130 -140 -150 -160 -170 -180 -190 -200 -210 -220 -230
f1 (ppm)

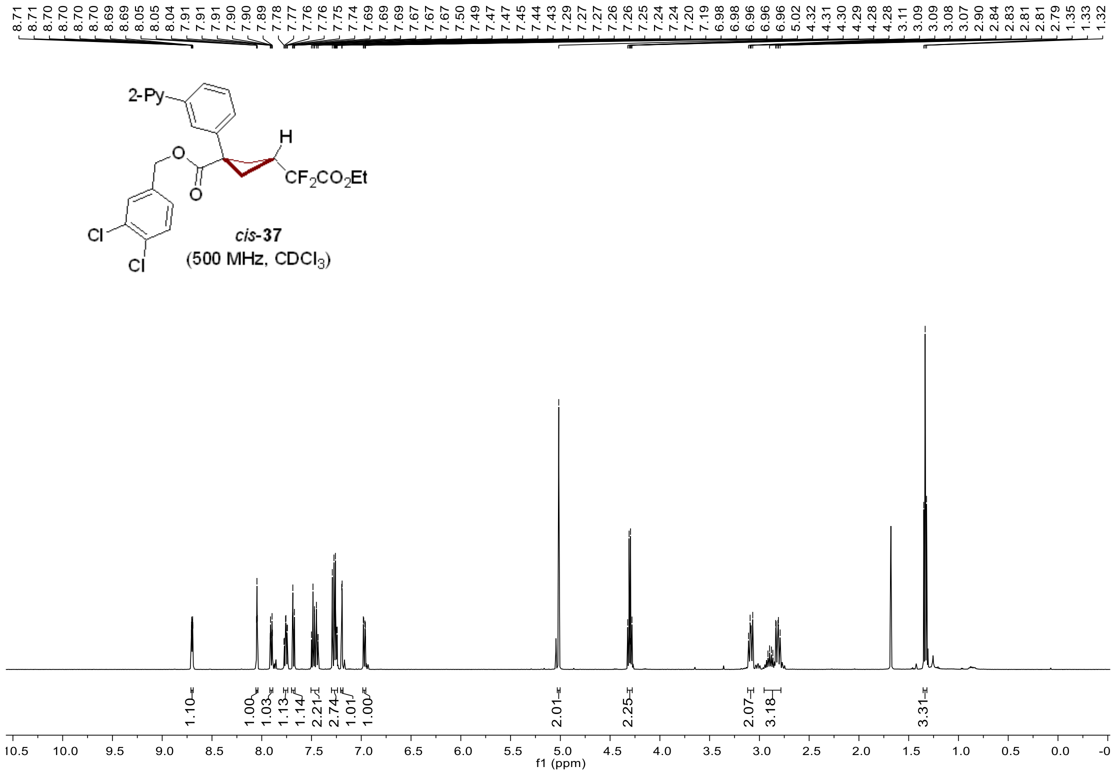
2-Py
H
CF2CO2Et
Cl
Cl
O
cis-37
(500 MHz, CDCl3)
f1 (ppm)

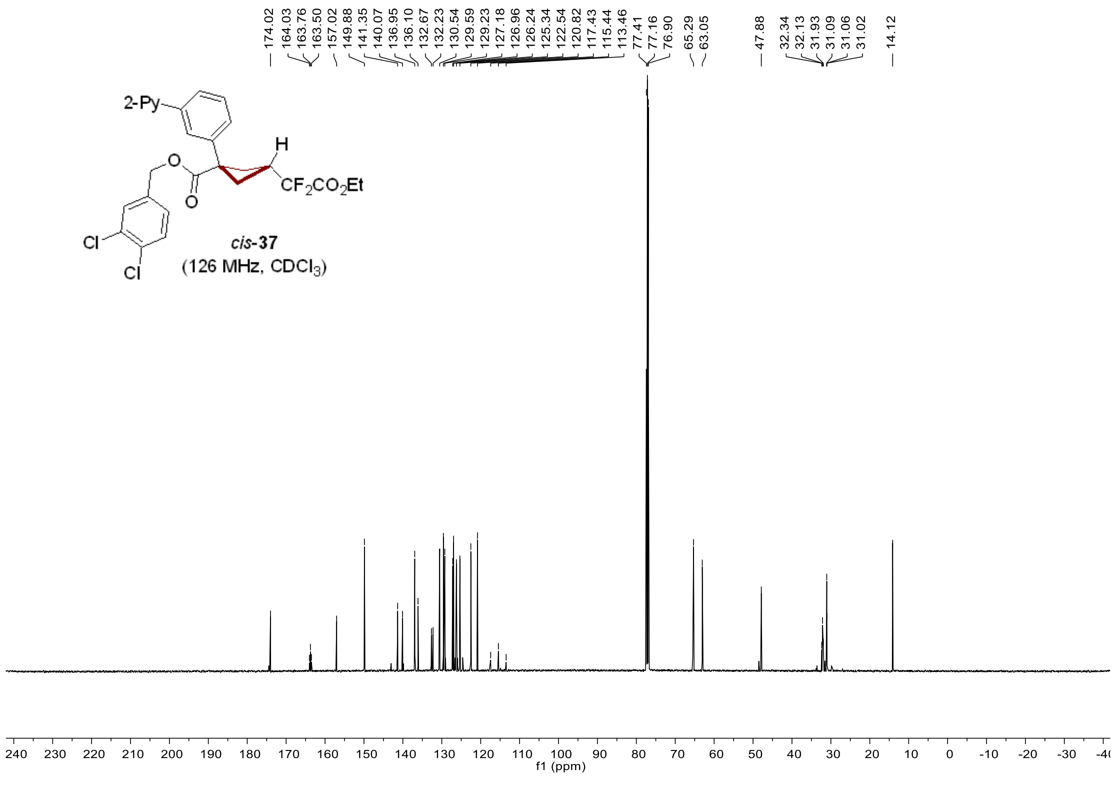
2-Py
H
CF2CO2Et
Cl
Cl
O
cis-37
(126 MHz, CDCl3)
f1 (ppm)

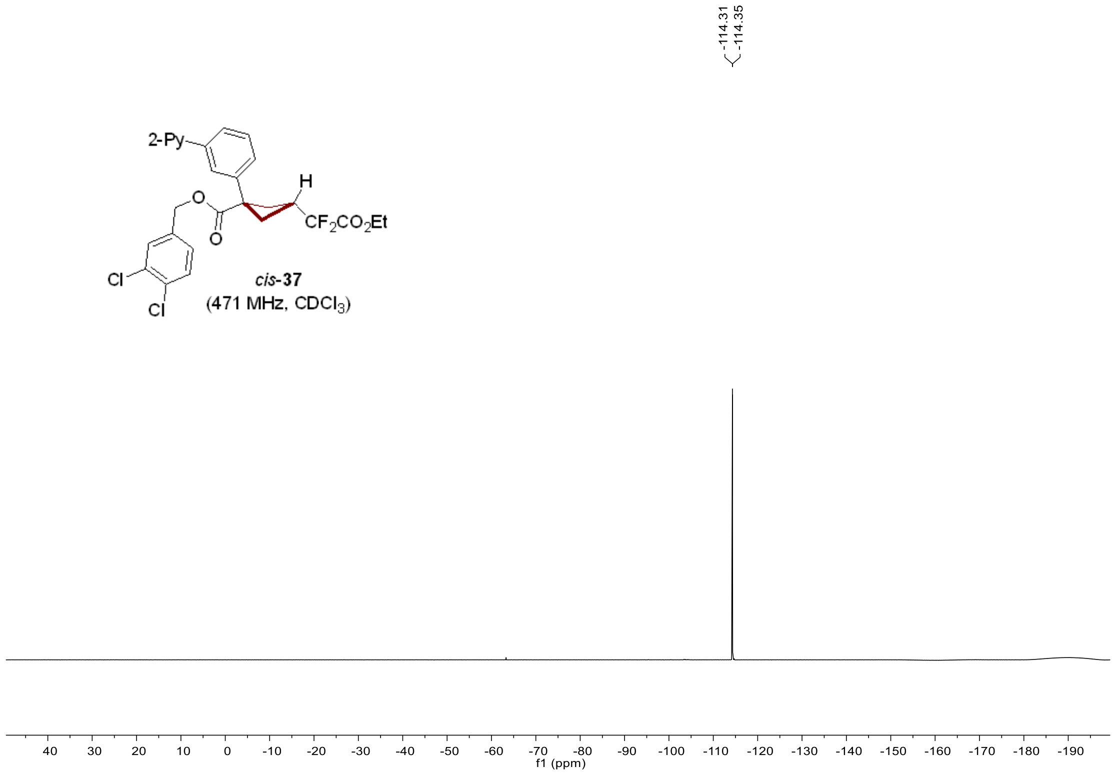
-114.31
-114.35
2-Py
H
O
CF2CO2Et
O
Cl
Cl
cis-37
(471 MHz, CDCl3)
40 30 20 10 0 -10 -20 -30 -40 -50 -60 -70 -80 -90 -100 -110 -120 -130 -140 -150 -160 -170 -180 -190
f1 (ppm)

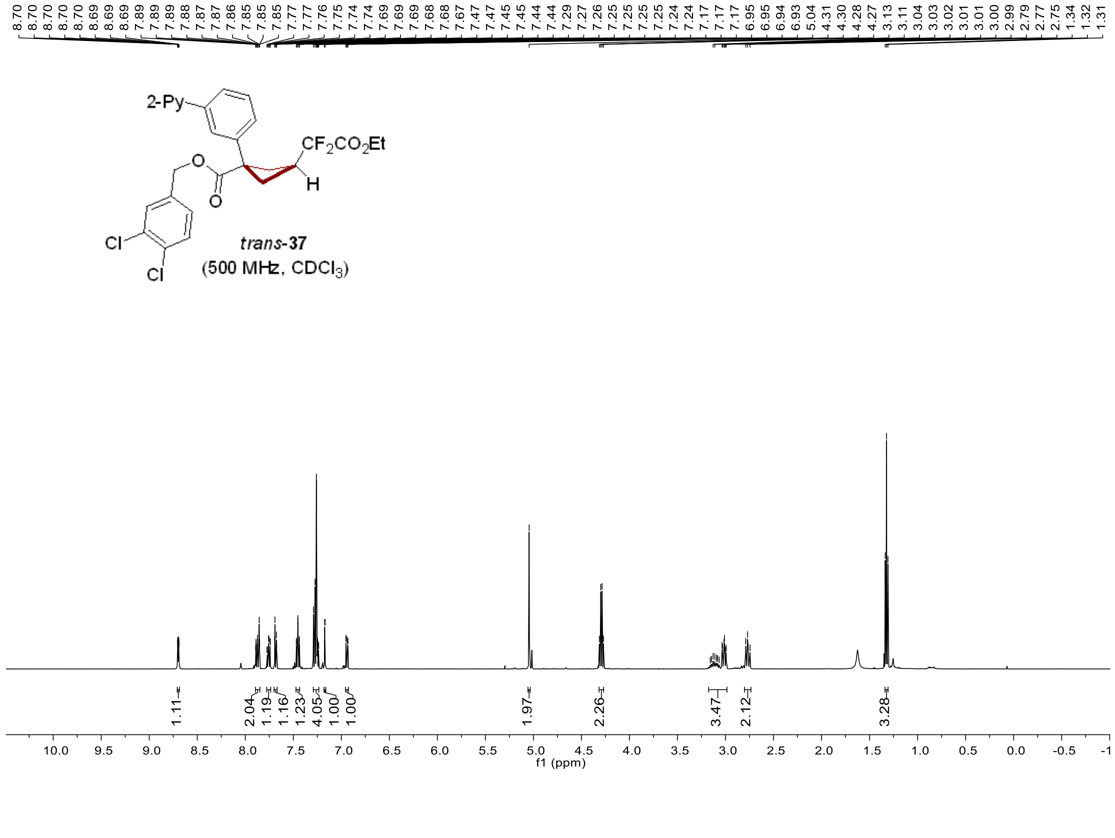
8.70 8.70 8.70 8.70 8.69 8.69 8.69 7.89 7.89 7.88 7.87 7.87 7.86 7.85 7.85 7.85 7.77 7.77 7.76 7.75 7.74 7.74 7.69 7.69 7.69 7.68 7.68 7.67 7.47 7.47 7.45 7.45 7.44 7.29 7.27 7.26 7.25 7.25 7.25 7.25 7.24 7.24 7.17 7.17 7.17 6.95 6.95 6.94 6.93 5.04 4.31 4.30 4.28 4.27 3.13 3.11 3.04 3.03 3.02 3.01 3.00 2.99 2.79 2.77 2.75 1.34 1.32 1.31
2-Py
CF2CO2Et
O
O
H
Cl
Cl
trans-37
(500 MHz, CDCl3)
1.11 2.04 1.19 1.16 1.23 4.05 1.00 1.00 1.97 2.26 3.47 2.12 3.28
10.0 9.5 9.0 8.5 8.0 7.5 7.0 6.5 6.0 5.5 5.0 4.5 4.0 3.5 3.0 2.5 2.0 1.5 1.0 0.5 0.0 -0.5 -1
f1 (ppm)

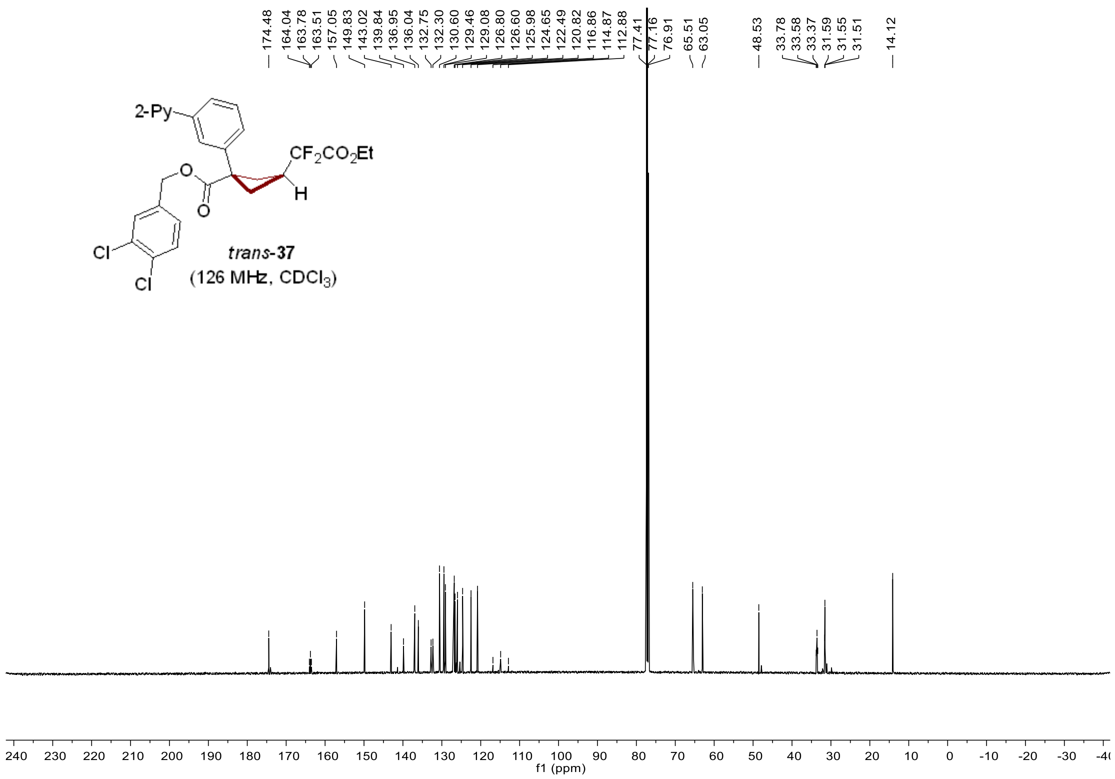

174.48
164.04
163.78
163.51
157.05
149.83
143.02
139.84
136.95
136.04
132.75
132.30
130.60
129.46
129.08
126.80
126.60
125.98
124.65
122.49
120.82
116.86
114.87
112.88
77.41
77.16
76.91
65.51
63.05
48.53
33.78
33.58
33.37
31.59
31.55
31.51
14.12
2-Py
$CF_2CO_2Et$
H
O
O
Cl
Cl
*trans*-**37**
(126 MHz, $CDCl_3$)
240 230 220 210 200 190 180 170 160 150 140 130 120 110 100 90 80 70 60 50 40 30 20 10 0 -10 -20 -30 -40
f1 (ppm)


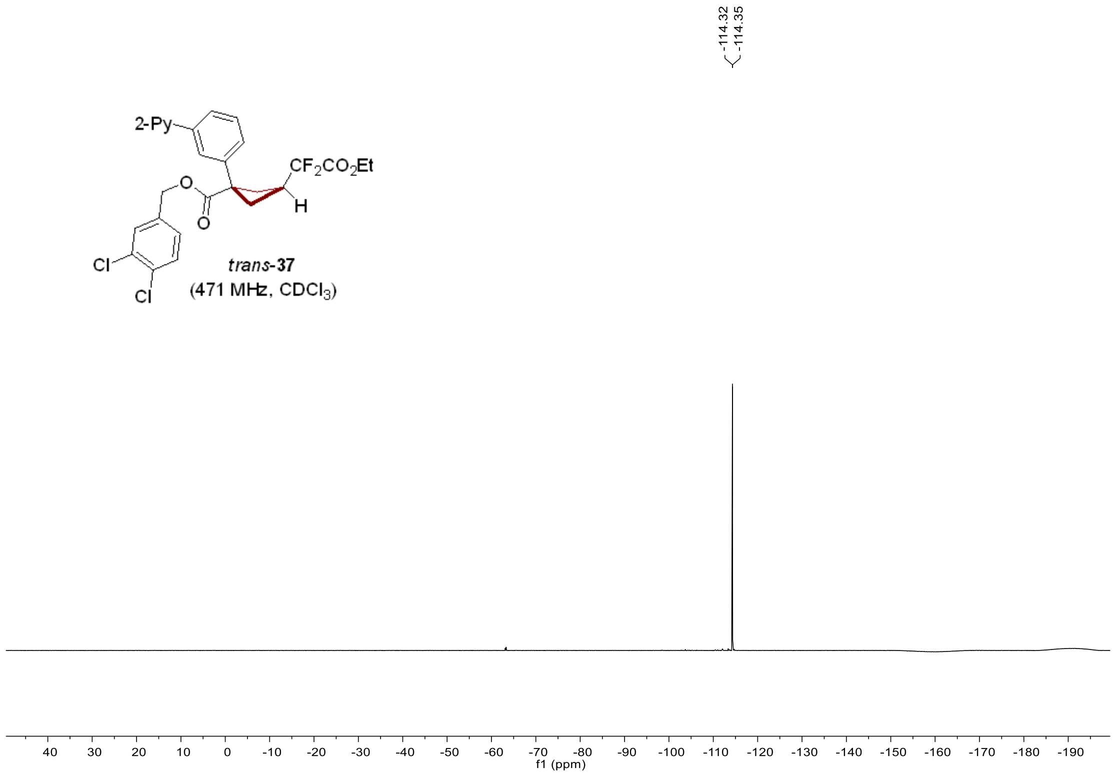

-114.32
-114.35
2-Py
$CF_2CO_2Et$
H
O
O
Cl
Cl
*trans*-**37**
(471 MHz, $CDCl_3$)
40 30 20 10 0 -10 -20 -30 -40 -50 -60 -70 -80 -90 -100 -110 -120 -130 -140 -150 -160 -170 -180 -190
f1 (ppm)

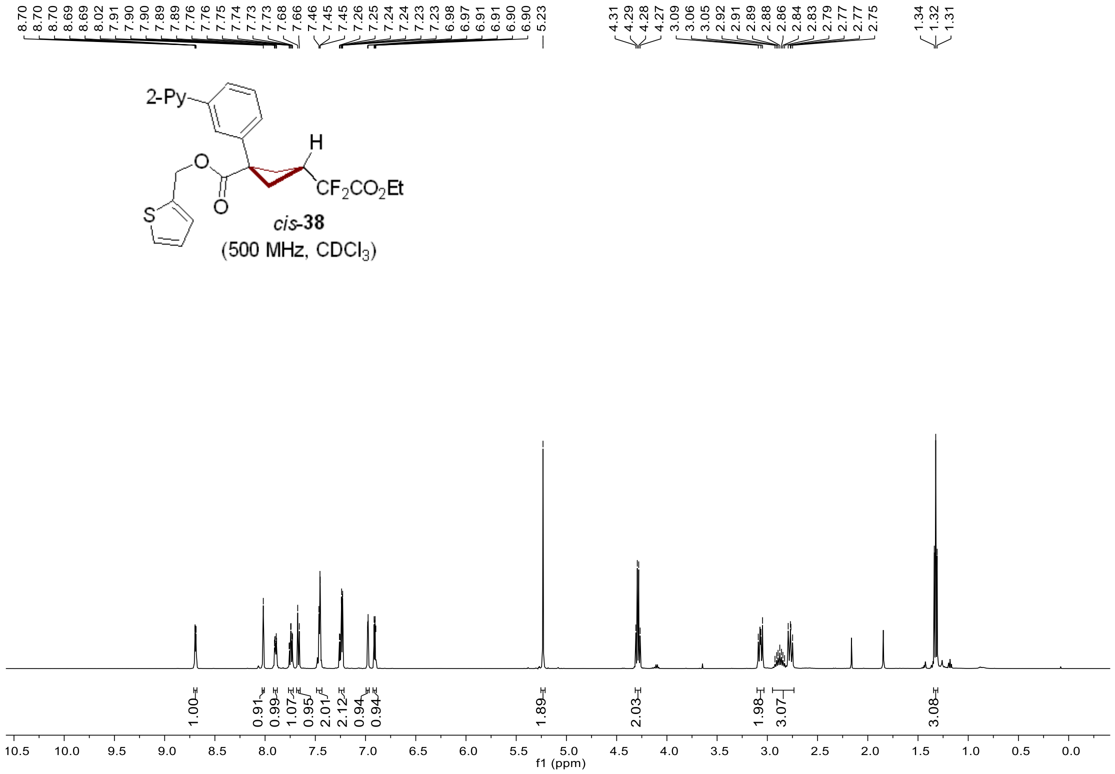
8.70 8.70 8.69 8.69 8.02 7.91 7.90 7.90 7.89 7.89 7.76 7.76 7.75 7.74 7.73 7.73 7.68 7.66 7.46 7.45 7.45 7.26 7.25 7.24 7.24 7.23 7.23 6.98 6.97 6.91 6.91 6.90 6.90 5.23
4.31 4.29 4.28 4.27 3.09 3.06 3.05 2.92 2.91 2.89 2.88 2.86 2.84 2.83 2.79 2.77 2.77 2.75
1.34 1.32 1.31
2-Py
H
O
O
S
$CF_2CO_2Et$
*cis*-**38**
(500 MHz, $CDCl_3$)
1.00 0.91 0.99 1.07 0.95 2.01 2.12 0.94 0.94 1.89 2.03 1.98 3.07 3.08
f1 (ppm)

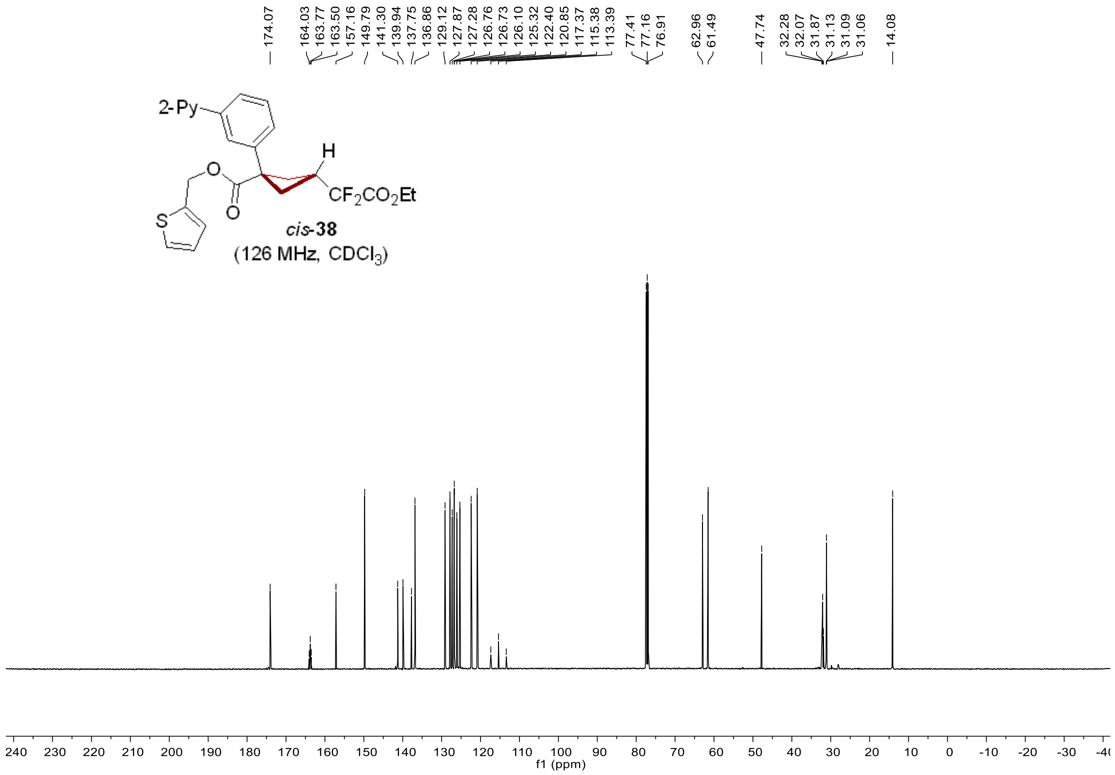
174.07
164.03 163.77 163.50 157.16 149.79 141.30 139.94 137.75 136.86 129.12 127.87 127.28 126.76 126.73 126.10 125.32 122.40 120.85 117.37 115.38 113.39
77.41 77.16 76.91
62.96 61.49
47.74
32.28 32.07 31.87 31.13 31.09 31.06
14.08
2-Py
H
O
O
S
$CF_2CO_2Et$
*cis*-**38**
(126 MHz, $CDCl_3$)
f1 (ppm)

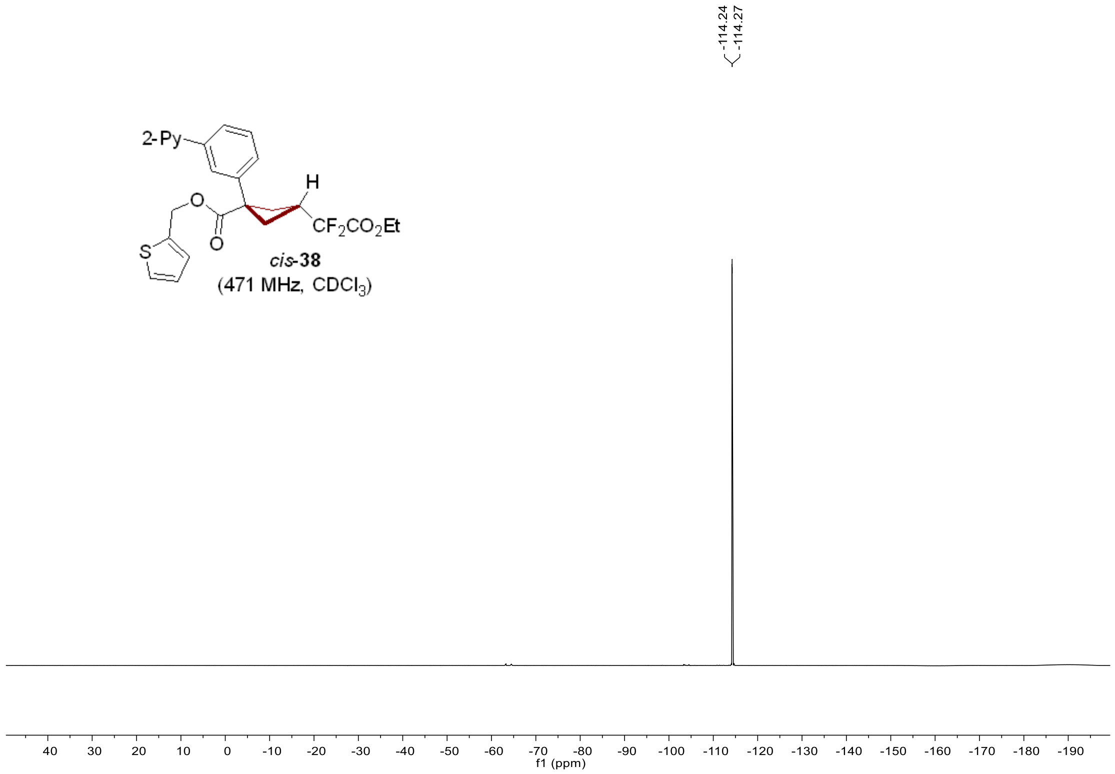
-114.24
-114.27
2-Py
H
CF2CO2Et
O
O
S
cis-38
(471 MHz, CDCl3)
40 30 20 10 0 -10 -20 -30 -40 -50 -60 -70 -80 -90 -100 -110 -120 -130 -140 -150 -160 -170 -180 -190
f1 (ppm)

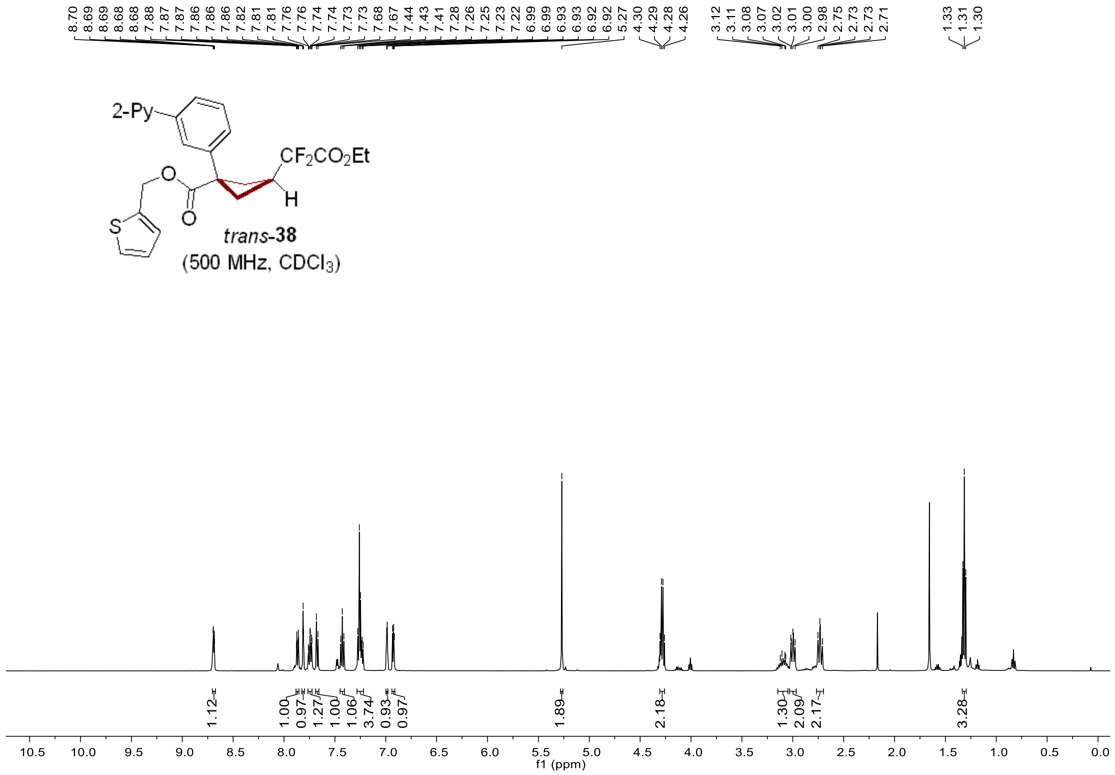
8.70 8.69 8.69 8.68 8.68 7.88 7.87 7.87 7.86 7.86 7.82 7.81 7.81 7.76 7.76 7.74 7.74 7.73 7.73 7.68 7.67 7.44 7.43 7.41 7.28 7.26 7.25 7.23 7.22 6.99 6.99 6.93 6.93 6.92 6.92 5.27 4.30 4.29 4.28 4.26 3.12 3.11 3.08 3.07 3.02 3.01 3.00 2.98 2.75 2.73 2.73 2.71 1.33 1.31 1.30
2-Py
CF2CO2Et
H
O
O
S
trans-38
(500 MHz, CDCl3)
1.12
1.00 0.97 1.27 1.00 1.06 3.74 0.93 0.97
1.89
2.18
1.30 2.09 2.17
3.28
10.5 10.0 9.5 9.0 8.5 8.0 7.5 7.0 6.5 6.0 5.5 5.0 4.5 4.0 3.5 3.0 2.5 2.0 1.5 1.0 0.5 0.0
f1 (ppm)

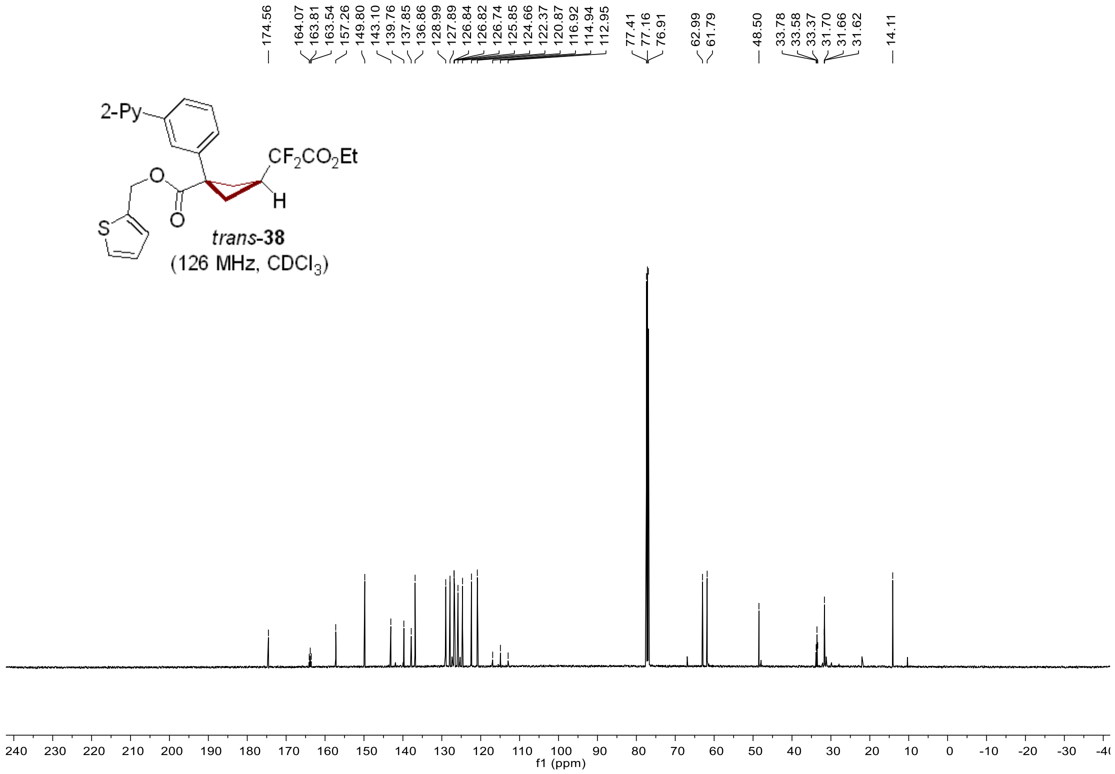
174.56
164.07
163.81
163.54
157.26
149.80
143.10
139.76
137.85
136.86
128.99
127.89
126.84
126.82
126.74
125.85
124.66
122.37
120.87
116.92
114.94
112.95
77.41
77.16
76.91
62.99
61.79
48.50
33.78
33.58
33.37
31.70
31.66
31.62
14.11
2-Py
CF2CO2Et
O
H
S
*trans*-**38**
(126 MHz, CDCl3)
f1 (ppm)

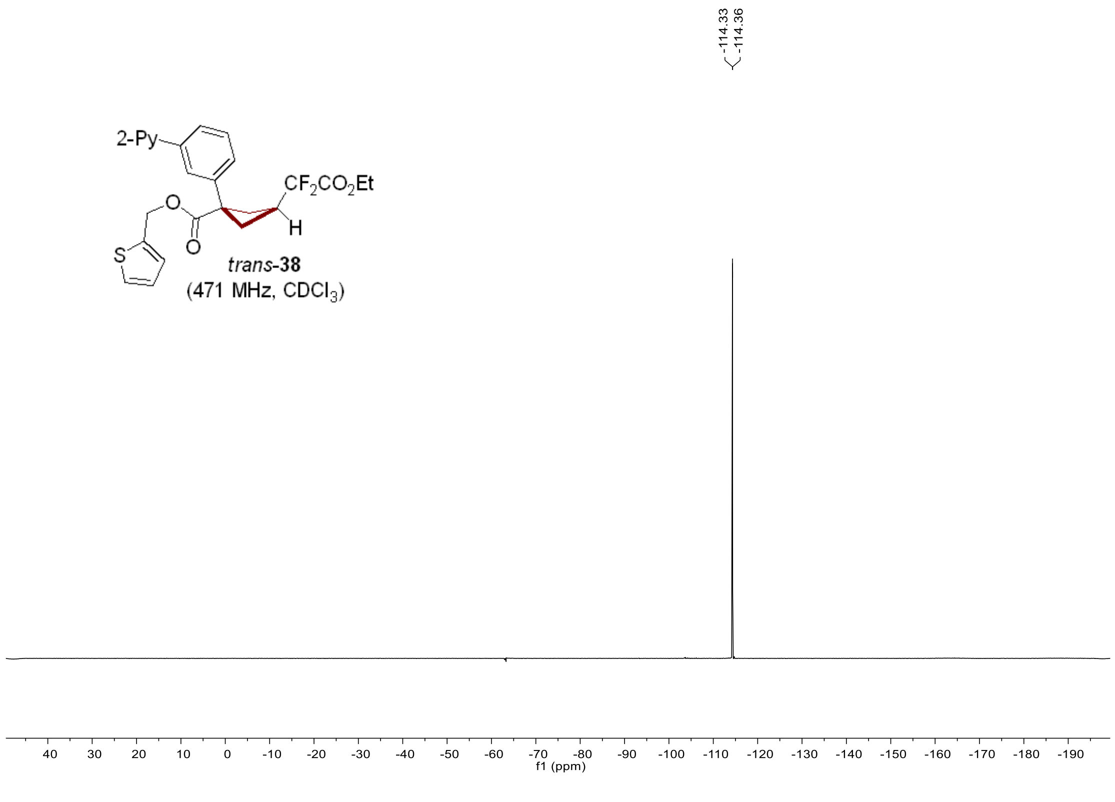
-114.33
-114.36
2-Py
CF2CO2Et
O
H
S
*trans*-**38**
(471 MHz, CDCl3)
f1 (ppm)

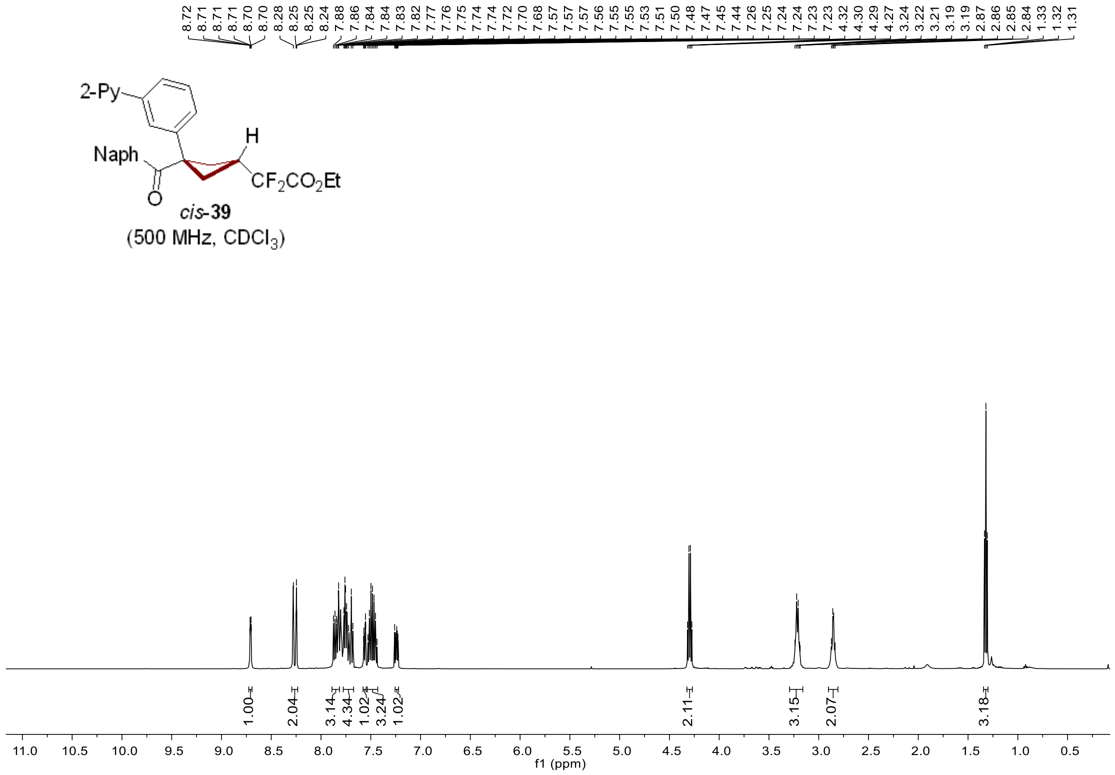


*cis*-**39**
(500 MHz, $CDCl_3$)

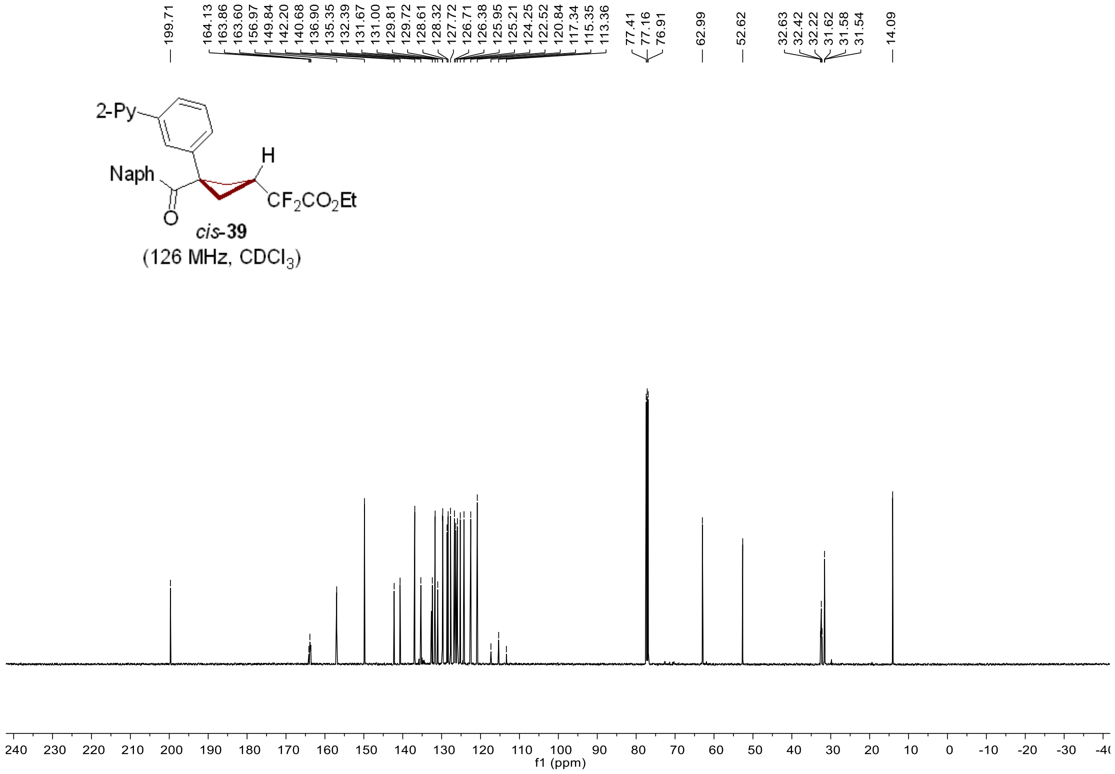


*cis*-**39**
(126 MHz, $CDCl_3$)

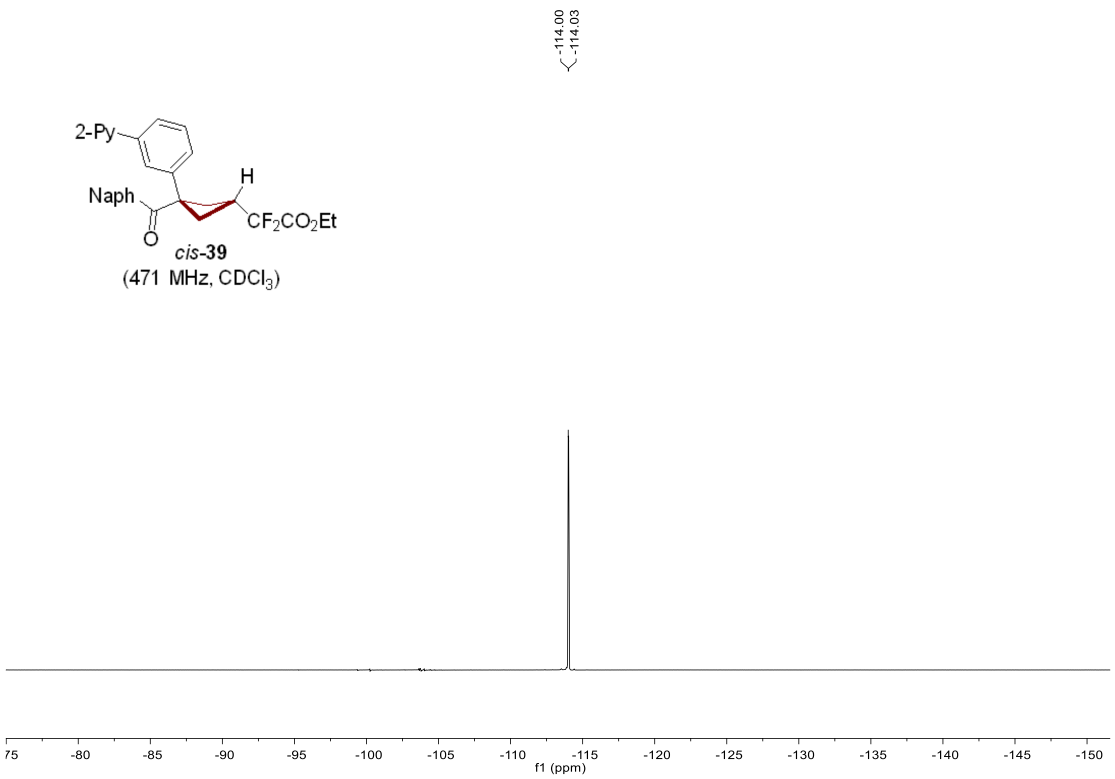
-114.00
-114.03
2-Py
Naph
H
CF2CO2Et
O
cis-39
(471 MHz, CDCl3)
f1 (ppm)

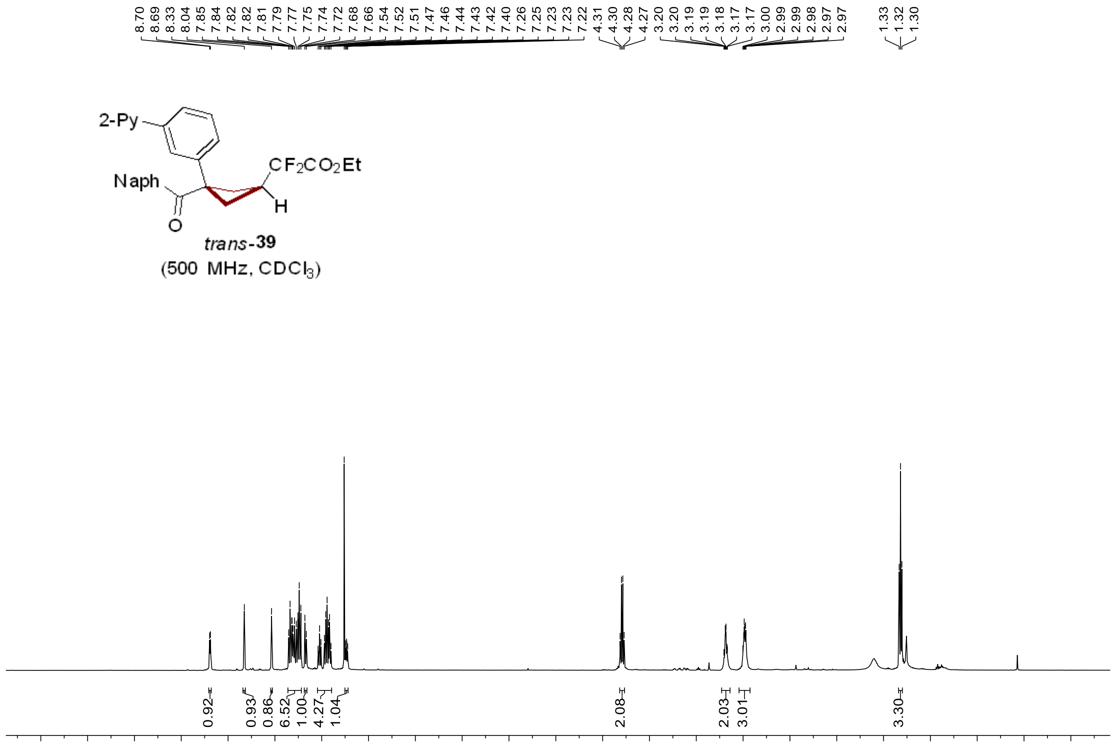
2-Py
Naph
CF2CO2Et
H
O
trans-39
(500 MHz, CDCl3)
f1 (ppm)

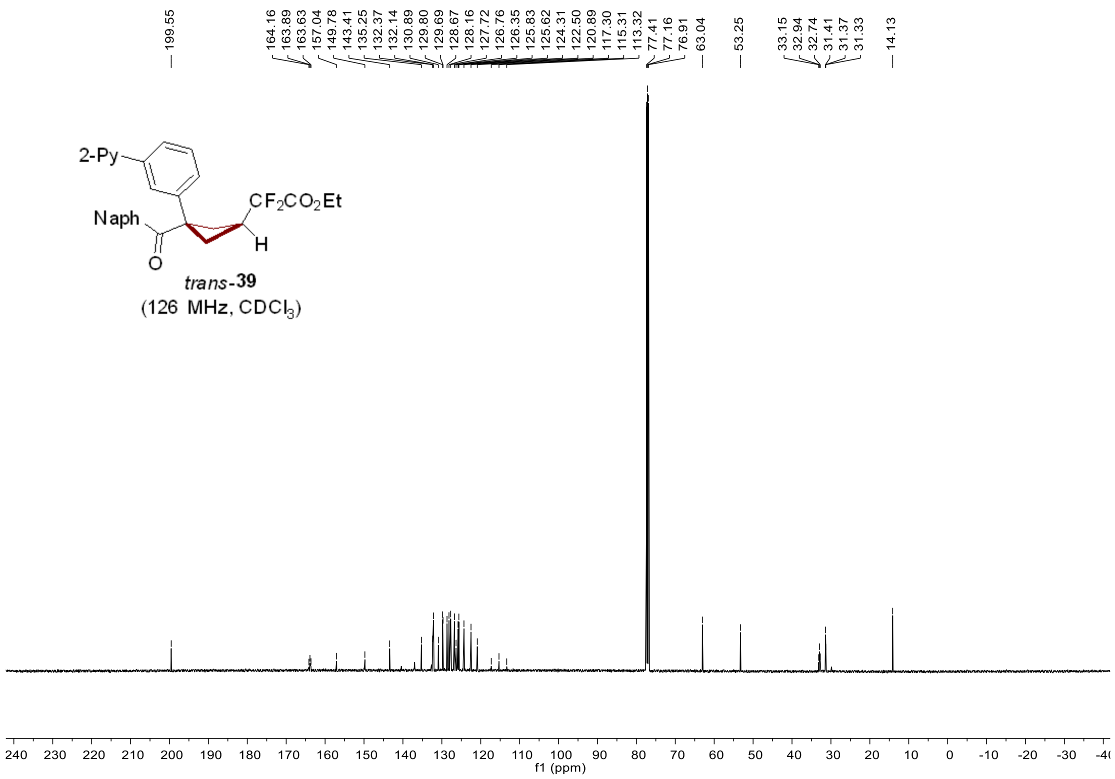
2-Py
Naph
CF2CO2Et
H
O
trans-39
(126 MHz, CDCl3)
199.55
164.16
163.89
163.63
157.04
149.78
143.41
135.25
132.37
132.14
130.89
129.80
129.69
128.67
128.16
127.72
126.76
126.35
125.83
125.62
124.31
122.50
120.89
117.30
115.31
113.32
77.41
77.16
76.91
63.04
53.25
33.15
32.94
32.74
31.41
31.37
31.33
14.13
f1 (ppm)

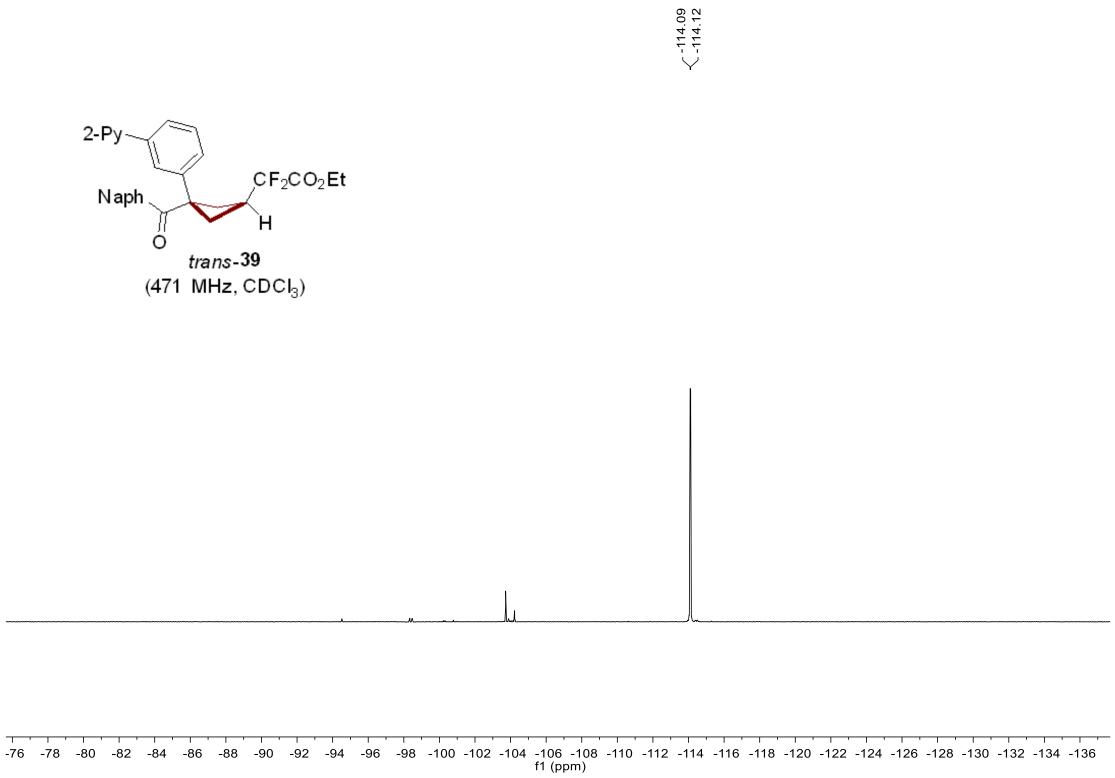
-114.09
-114.12
2-Py
Naph
CF2CO2Et
H
O
trans-39
(471 MHz, CDCl3)
f1 (ppm)

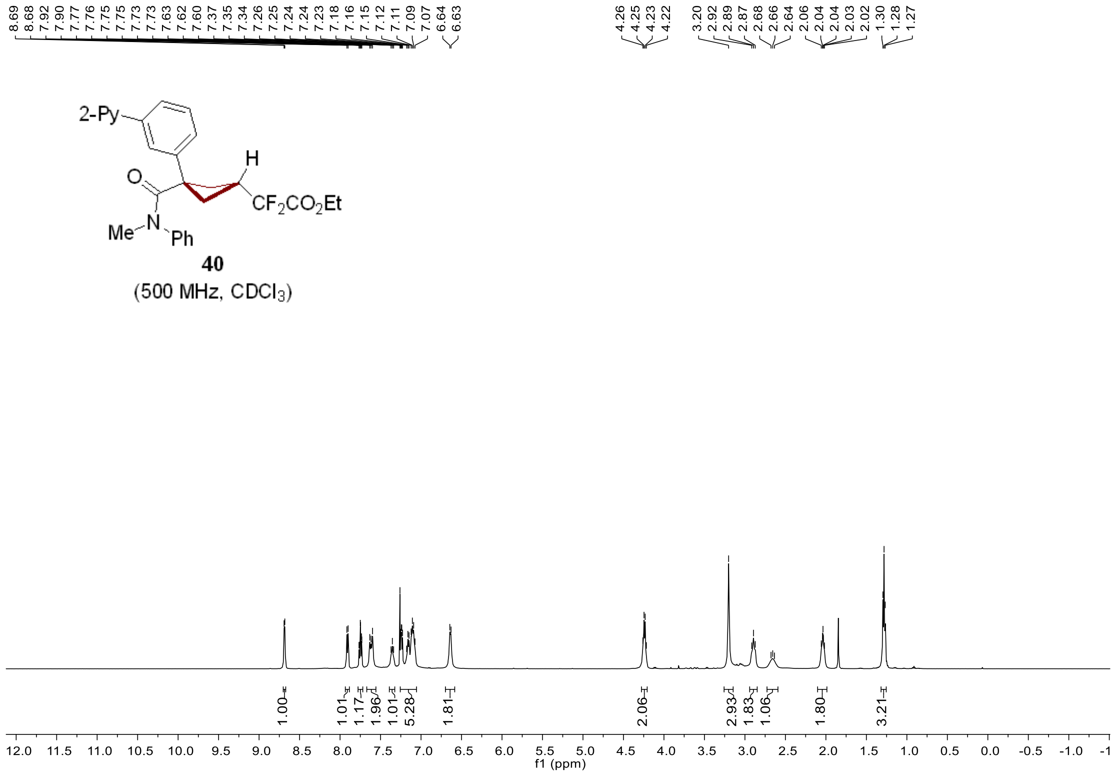
8.69 8.68 7.92 7.90 7.77 7.76 7.75 7.75 7.73 7.73 7.63 7.62 7.60 7.37 7.35 7.34 7.26 7.25 7.24 7.24 7.23 7.18 7.16 7.15 7.12 7.11 7.09 7.07 6.64 6.63
4.26 4.25 4.23 4.22 3.20 2.92 2.89 2.87 2.68 2.66 2.64 2.06 2.04 2.04 2.03 2.02 1.30 1.28 1.27
2-Py
O
H
N
CF2CO2Et
Me
Ph
40
(500 MHz, CDCl3)
1.00 1.01 1.17 1.96 1.01 5.28 1.81 2.06 2.93 1.83 1.06 1.80 3.21
12.0 11.5 11.0 10.5 10.0 9.5 9.0 8.5 8.0 7.5 7.0 6.5 6.0 5.5 5.0 4.5 4.0 3.5 3.0 2.5 2.0 1.5 1.0 0.5 0.0 -0.5 -1.0 -1
f1 (ppm)

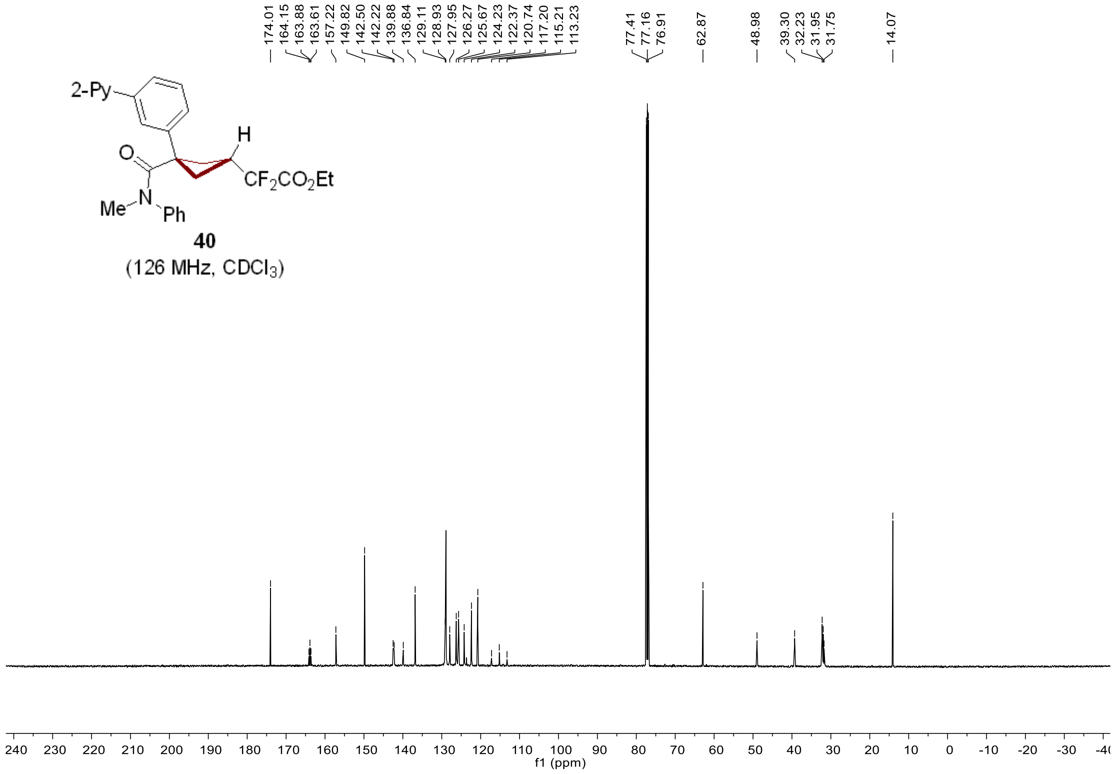
174.01 164.15 163.88 163.61 157.22 149.82 142.50 142.22 139.88 136.84 129.11 128.93 127.95 126.27 125.67 124.23 122.37 120.74 117.20 115.21 113.23
77.41 77.16 76.91 62.87 48.98 39.30 32.23 31.95 31.75 14.07
2-Py
O
H
N
CF2CO2Et
Me
Ph
40
(126 MHz, CDCl3)
240 230 220 210 200 190 180 170 160 150 140 130 120 110 100 90 80 70 60 50 40 30 20 10 0 -10 -20 -30 -40
f1 (ppm)

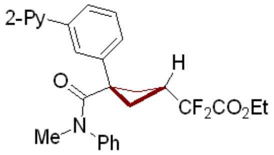


**40**
(471 MHz, $CDCl_3$)

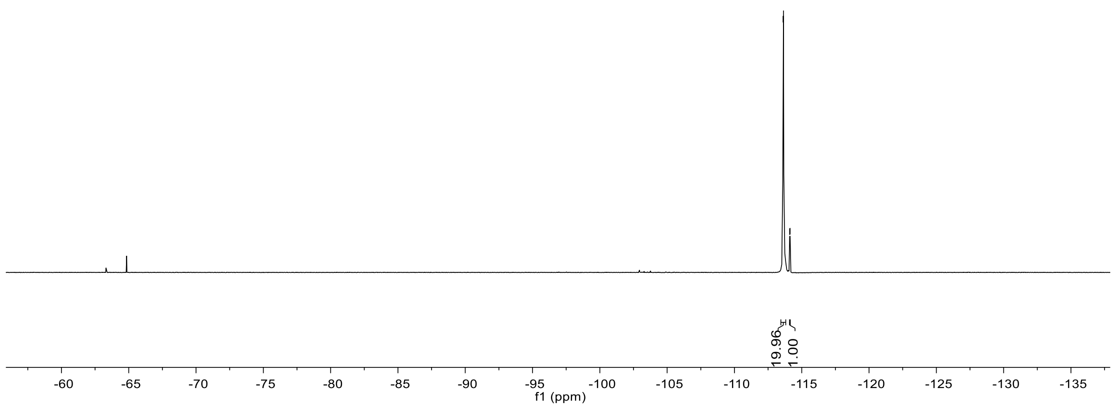


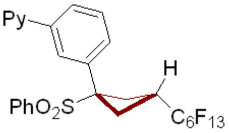


*cis*-**41**
(500 MHz, $CDCl_3$)

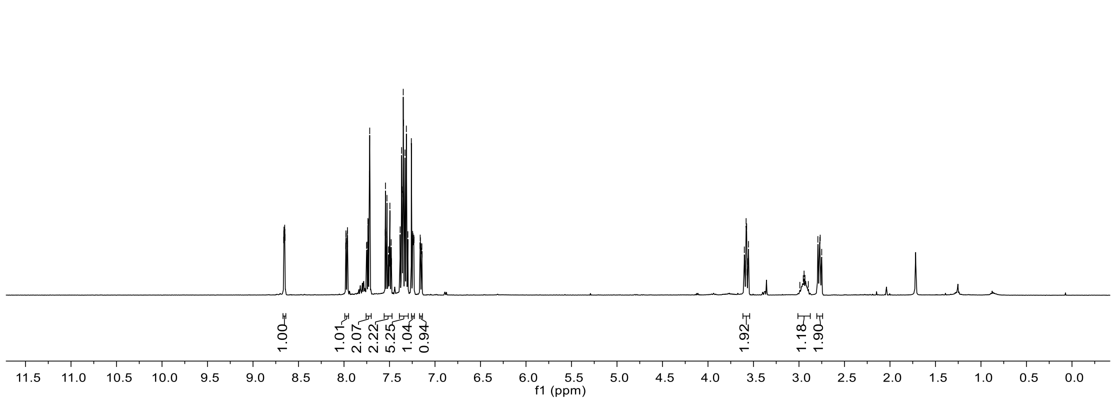

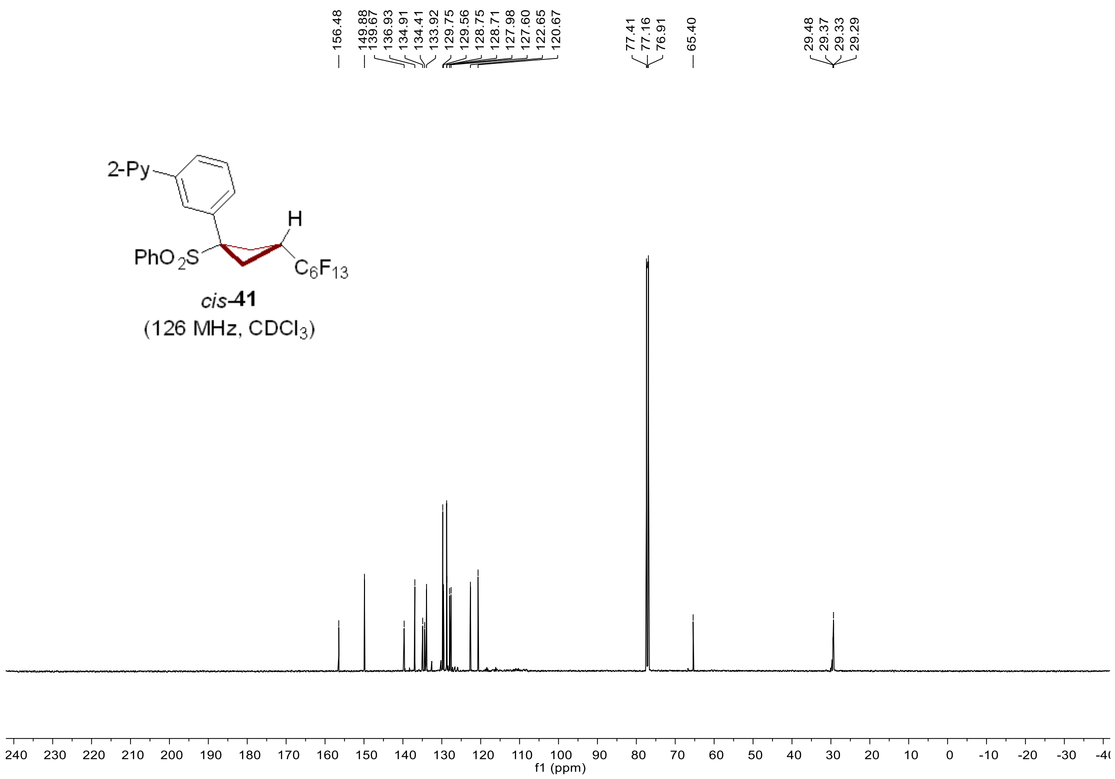
156.48
149.88
139.67
136.93
134.91
134.41
133.92
129.75
129.56
128.75
128.71
127.98
127.60
122.65
120.67
77.41
77.16
76.91
65.40
29.48
29.37
29.33
29.29
2-Py
H
PhO2S
C6F13
cis-41
(126 MHz, CDCl3)
f1 (ppm)

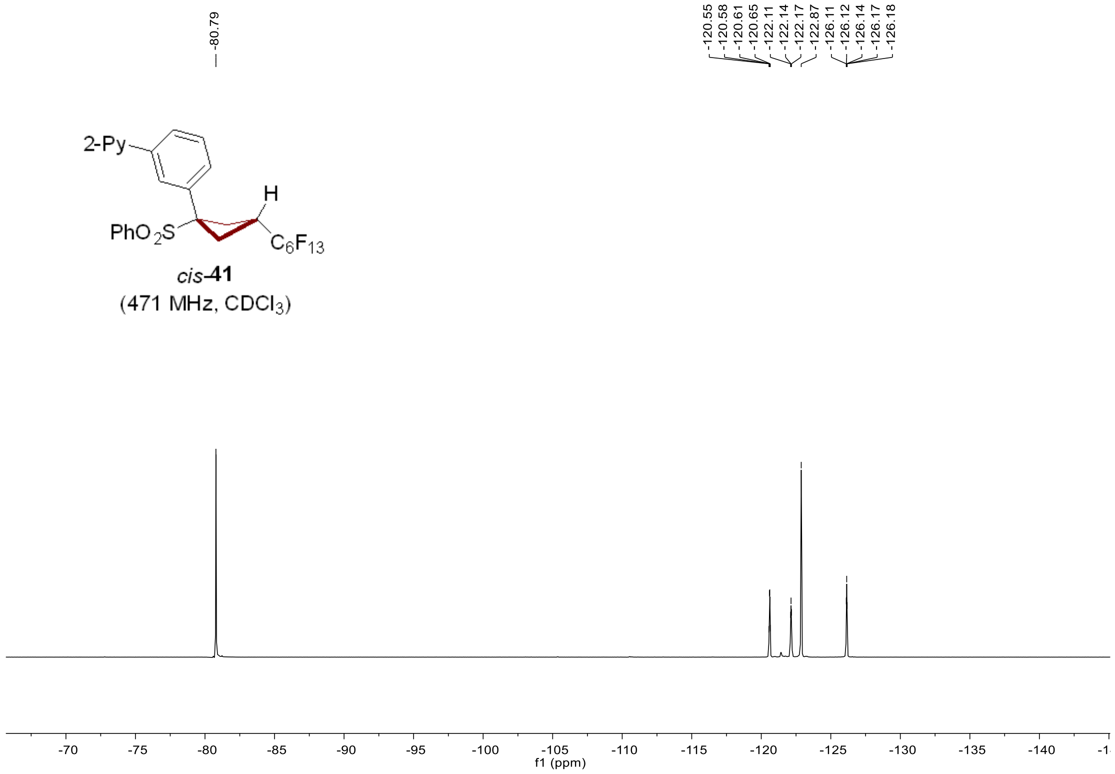
-80.79
-120.55
-120.58
-120.61
-120.65
-122.11
-122.14
-122.17
-122.87
-126.11
-126.12
-126.14
-126.17
-126.18
2-Py
H
PhO2S
C6F13
cis-41
(471 MHz, CDCl3)
f1 (ppm)

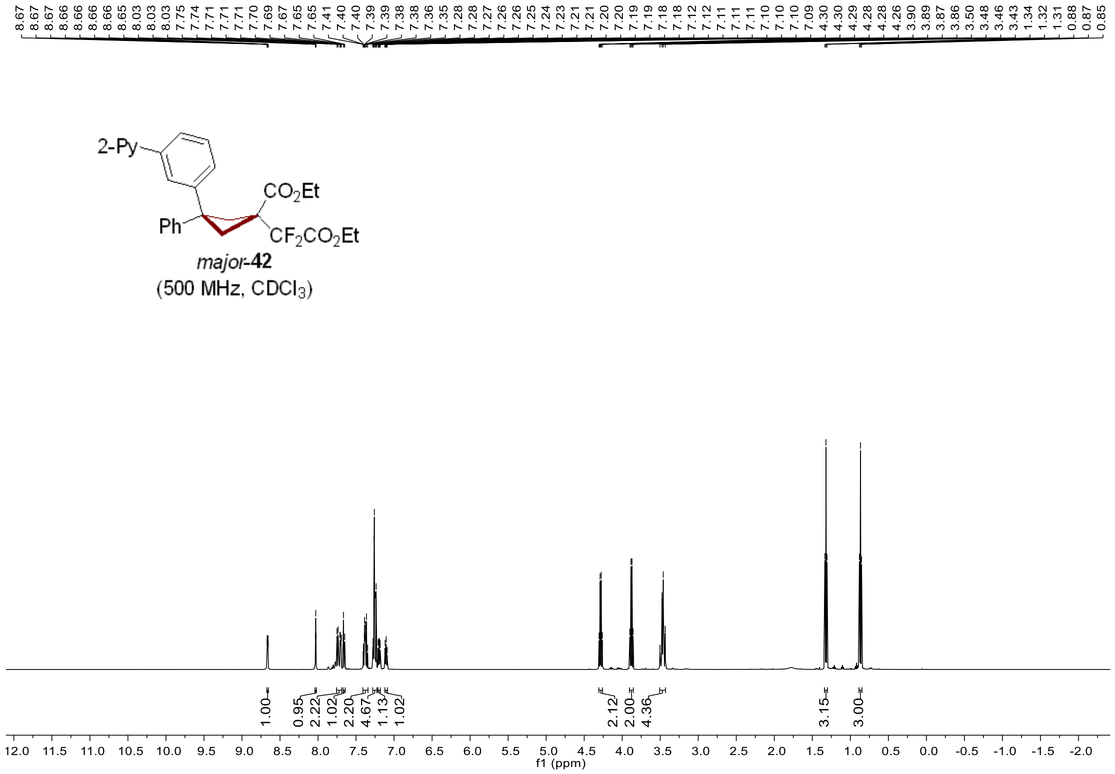
2-Py
CO2Et
Ph
CF2CO2Et
major-42
(500 MHz, CDCl3)
f1 (ppm)

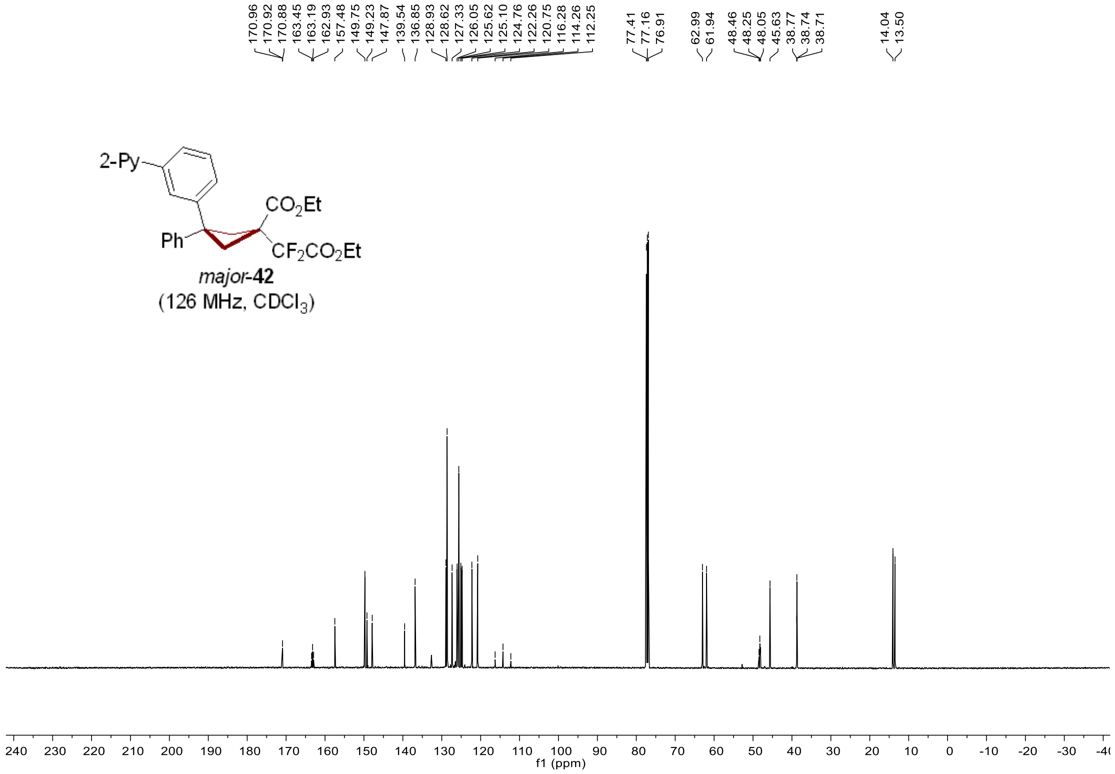
2-Py
CO2Et
Ph
CF2CO2Et
major-42
(126 MHz, CDCl3)
f1 (ppm)

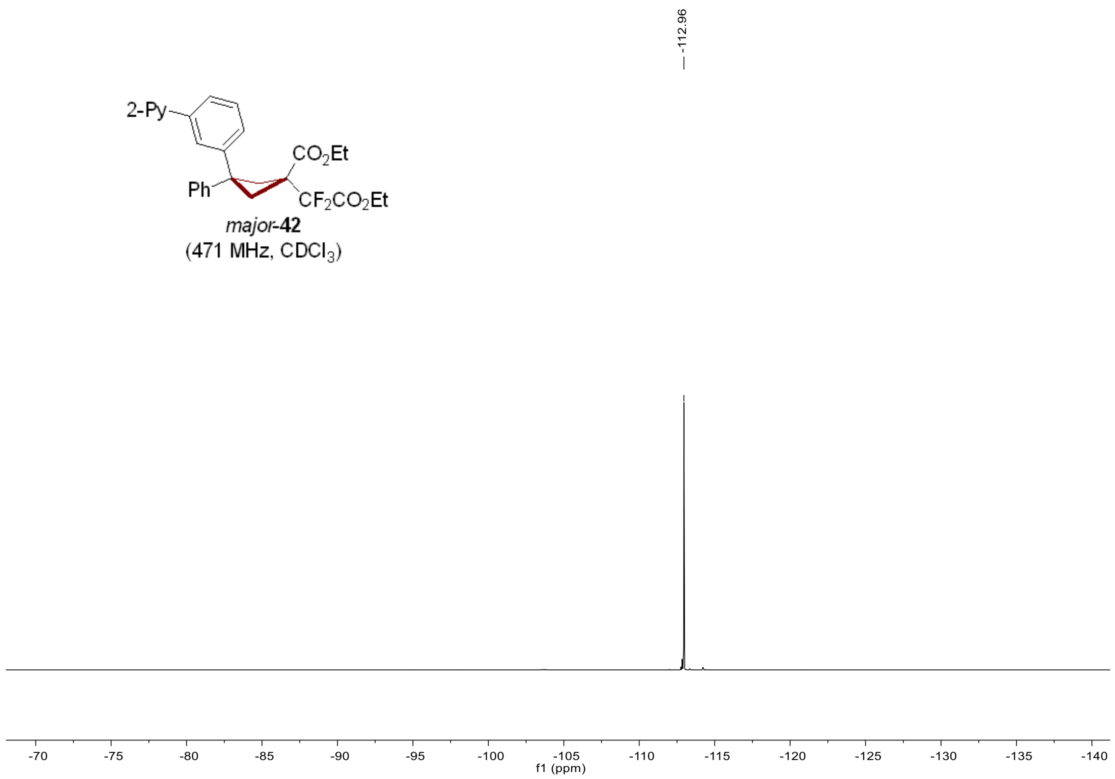
-112.96
2-Py
CO2Et
Ph
CF2CO2Et
major-42
(471 MHz, CDCl3)
f1 (ppm)

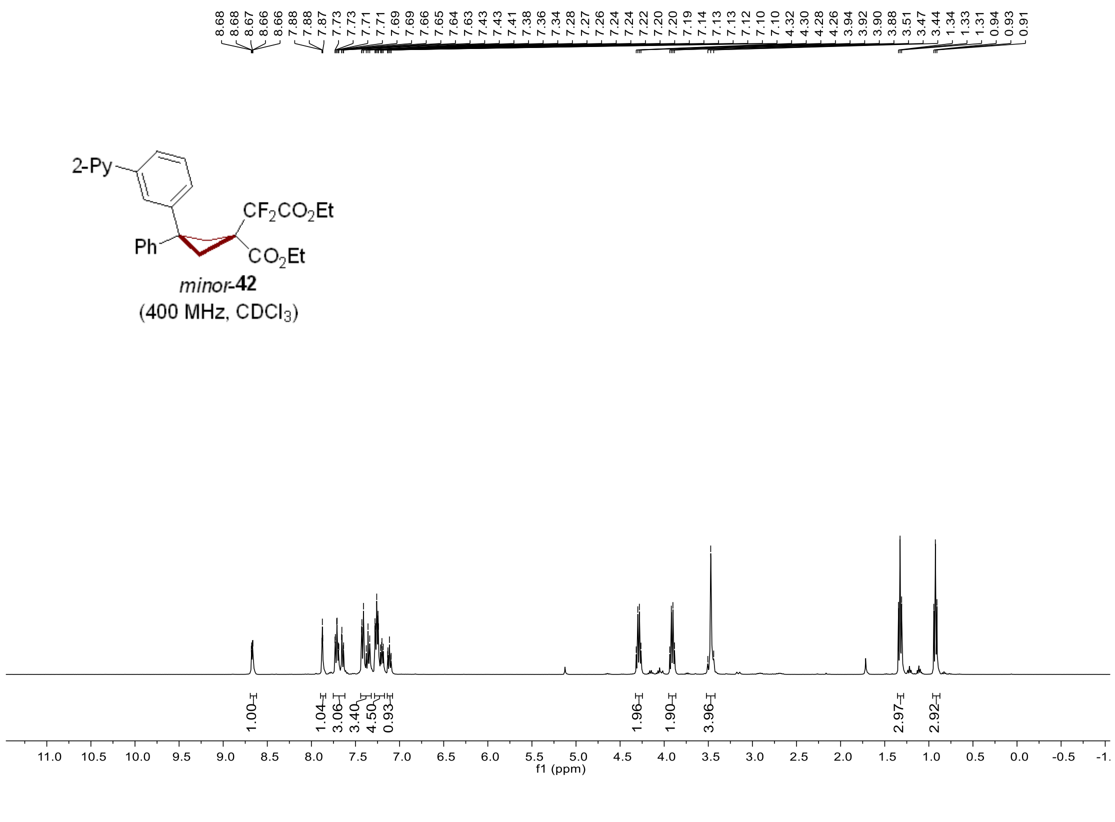
2-Py
CF2CO2Et
Ph
CO2Et
minor-42
(400 MHz, CDCl3)
1.00
1.04
3.06
3.40
4.50
0.93
1.96
1.90
3.96
2.97
2.92
f1 (ppm)

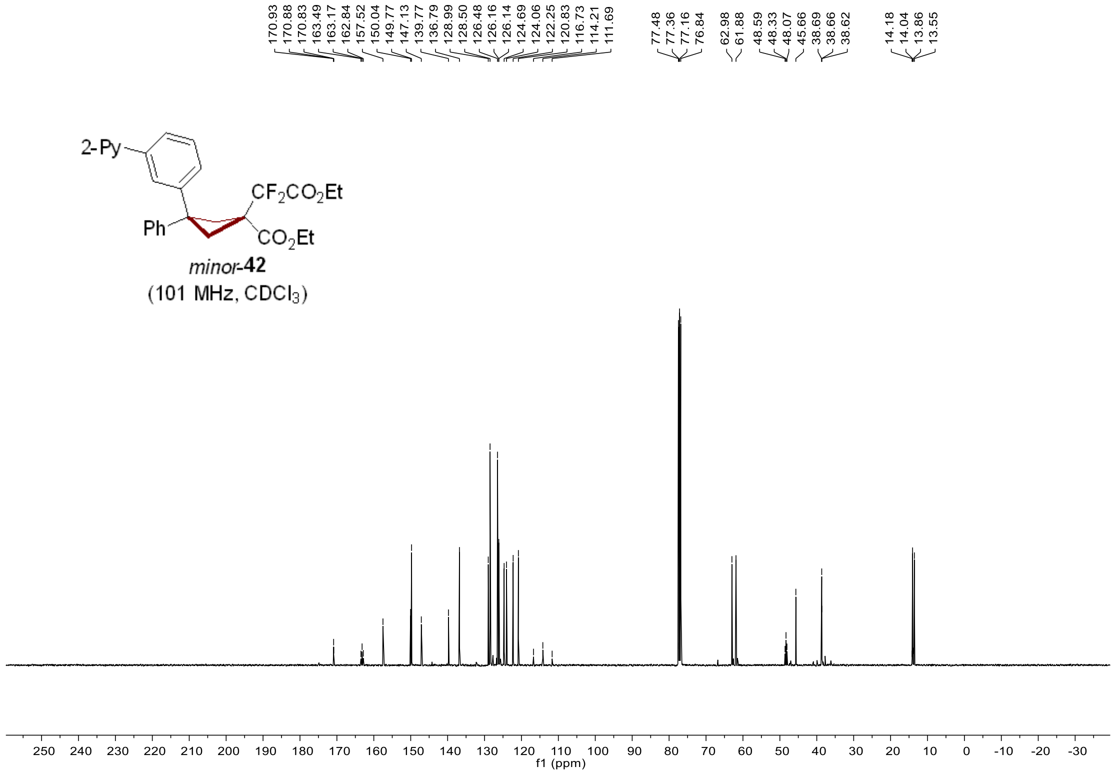
170.93
170.88
170.83
163.49
163.17
162.84
157.52
150.04
149.77
147.13
139.77
136.79
128.99
128.50
126.48
126.16
126.14
124.69
124.06
122.25
120.83
116.73
114.21
111.69
77.48
77.36
77.16
76.84
62.98
61.88
48.59
48.33
48.07
45.66
38.69
38.66
38.62
14.18
14.04
13.86
13.55
2-Py
CF2CO2Et
Ph
CO2Et
minor-42
(101 MHz, CDCl3)
f1 (ppm)

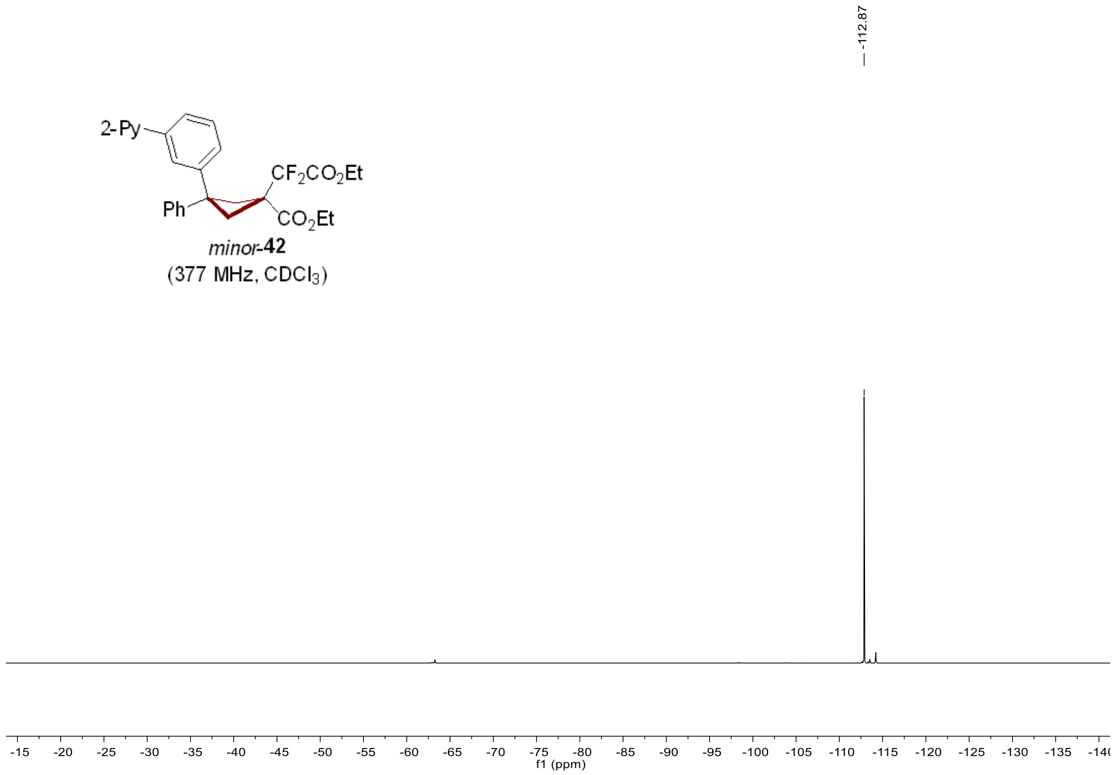
-112.87
2-Py
CF2CO2Et
Ph
CO2Et
minor-42
(377 MHz, CDCl3)
f1 (ppm)

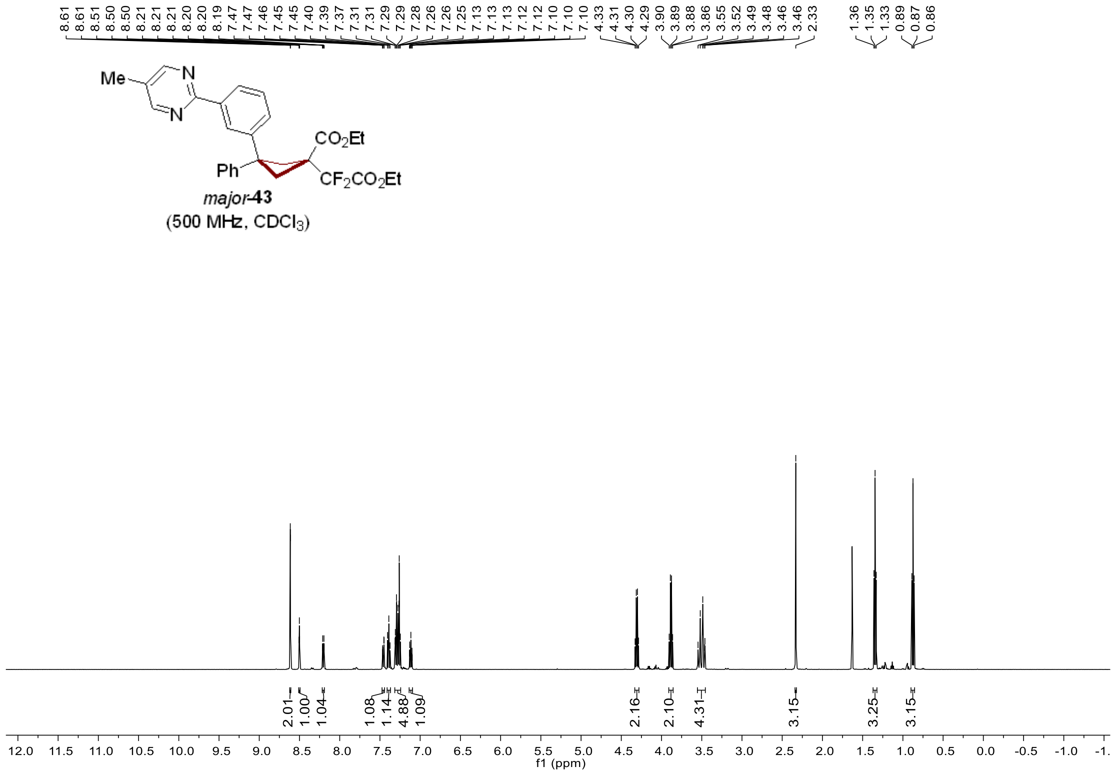
8.61 8.61 8.51 8.50 8.21 8.21 8.20 8.19 7.47 7.46 7.45 7.45 7.40 7.39 7.37 7.31 7.31 7.29 7.29 7.28 7.26 7.25 7.13 7.13 7.13 7.12 7.10 7.10 4.33 4.31 4.30 4.29 3.90 3.89 3.88 3.86 3.55 3.52 3.49 3.48 3.46 2.33 1.36 1.35 1.33 0.89 0.87 0.86
Me
N
N
$CO_2Et$
Ph
$CF_2CO_2Et$
*major*-**43**
(500 MHz, $CDCl_3$)
2.01 1.00 1.04 1.08 1.14 4.88 1.09 2.16 2.10 4.31 3.15 3.25 3.15
f1 (ppm)

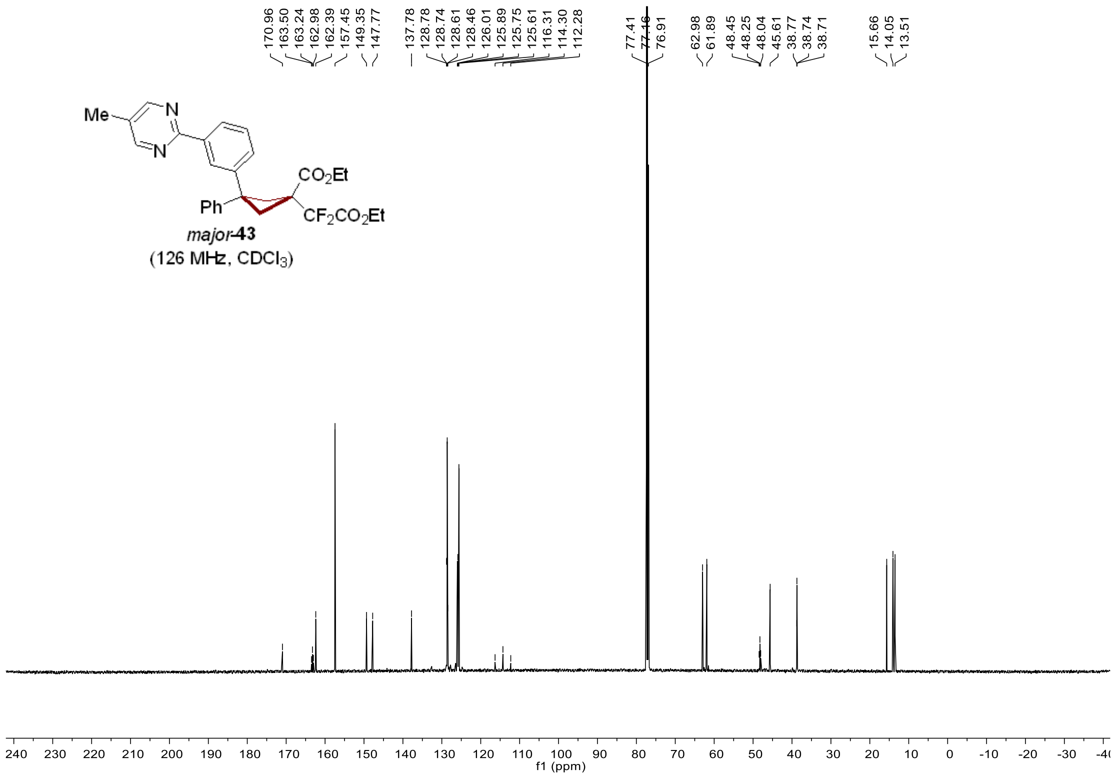
170.96 163.50 163.24 162.98 162.39 157.45 149.35 147.77 137.78 128.78 128.74 128.61 128.46 126.01 125.89 125.75 125.61 116.31 114.30 112.28 77.41 77.16 76.91 62.98 61.89 48.45 48.25 48.04 45.61 38.77 38.74 38.71 15.66 14.05 13.51
Me
N
N
$CO_2Et$
Ph
$CF_2CO_2Et$
*major*-**43**
(126 MHz, $CDCl_3$)
f1 (ppm)

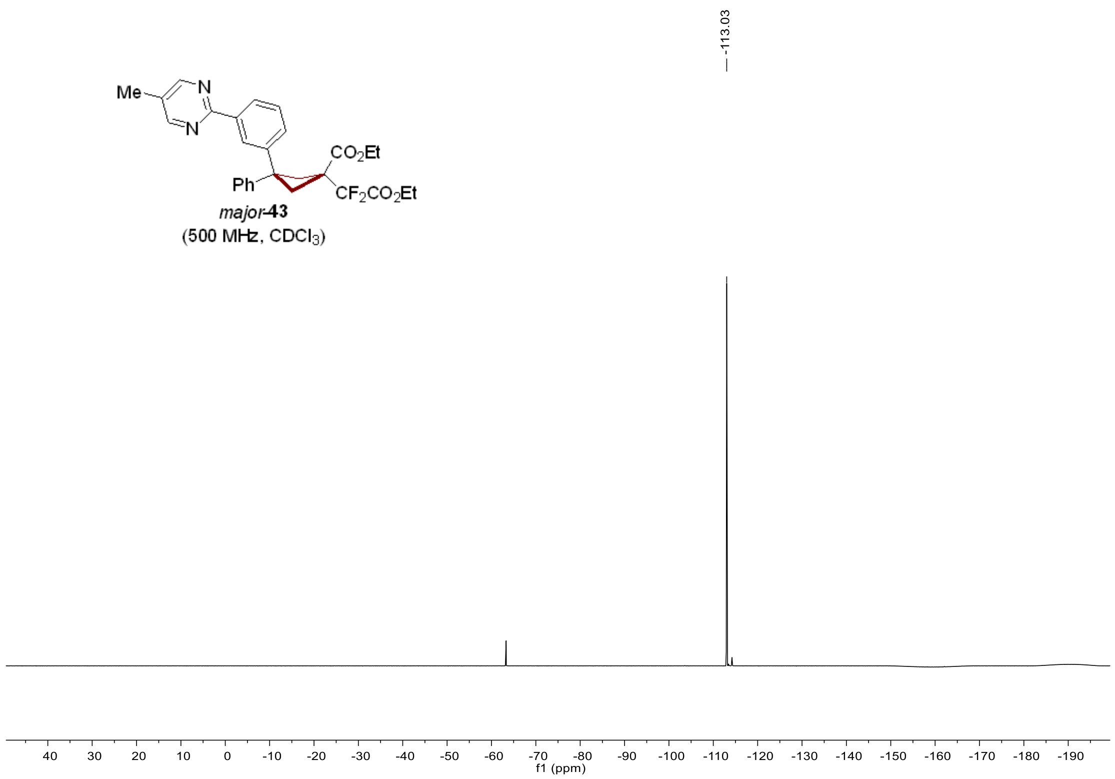
-113.03
Me
N
N
CO2Et
Ph
CF2CO2Et
major-43
(500 MHz, CDCl3)
40 30 20 10 0 -10 -20 -30 -40 -50 -60 -70 -80 -90 -100 -110 -120 -130 -140 -150 -160 -170 -180 -190
f1 (ppm)

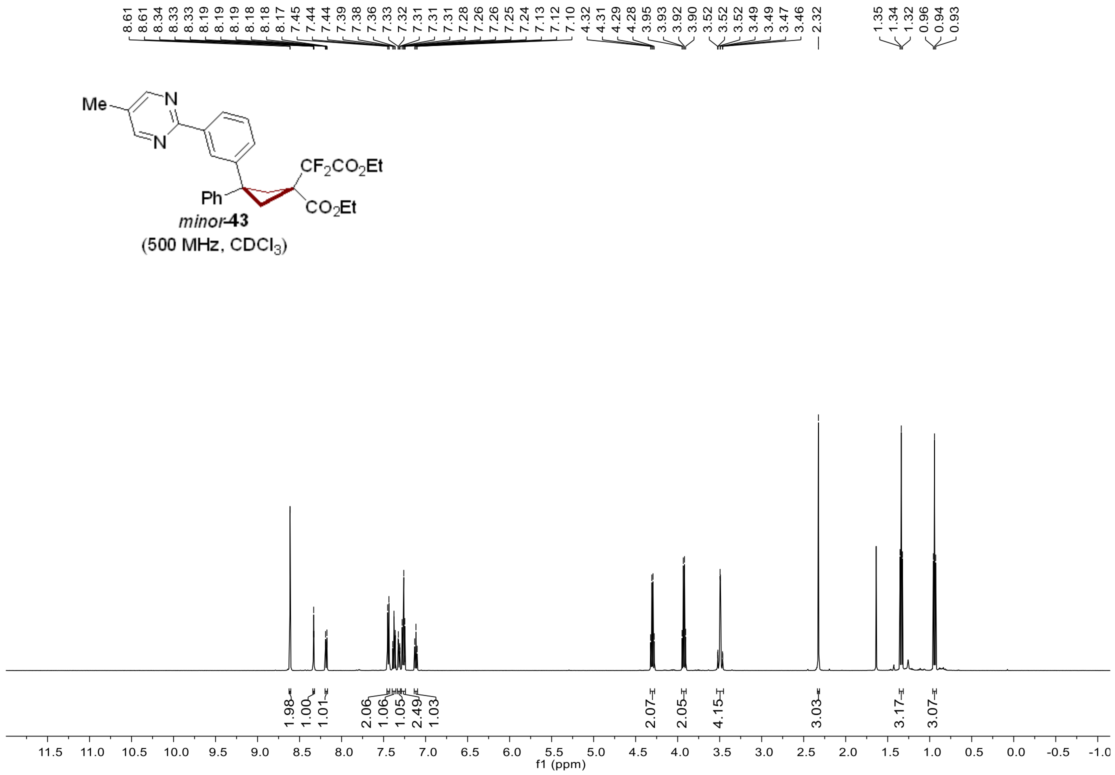
8.61 8.61 8.34 8.33 8.33 8.19 8.19 8.19 8.18 8.18 8.17 7.45 7.44 7.44 7.39 7.38 7.36 7.33 7.32 7.31 7.31 7.28 7.26 7.26 7.25 7.24 7.13 7.12 7.10 4.32 4.31 4.29 4.28 3.95 3.93 3.92 3.90 3.52 3.52 3.52 3.49 3.47 3.46 2.32 1.35 1.34 1.32 0.96 0.94 0.93
Me
N
N
CF2CO2Et
Ph
CO2Et
minor-43
(500 MHz, CDCl3)
1.98 1.00 1.01 2.06 1.06 1.05 2.49 1.03 2.07 2.05 4.15 3.03 3.17 3.07
11.5 11.0 10.5 10.0 9.5 9.0 8.5 8.0 7.5 7.0 6.5 6.0 5.5 5.0 4.5 4.0 3.5 3.0 2.5 2.0 1.5 1.0 0.5 0.0 -0.5 -1.0
f1 (ppm)

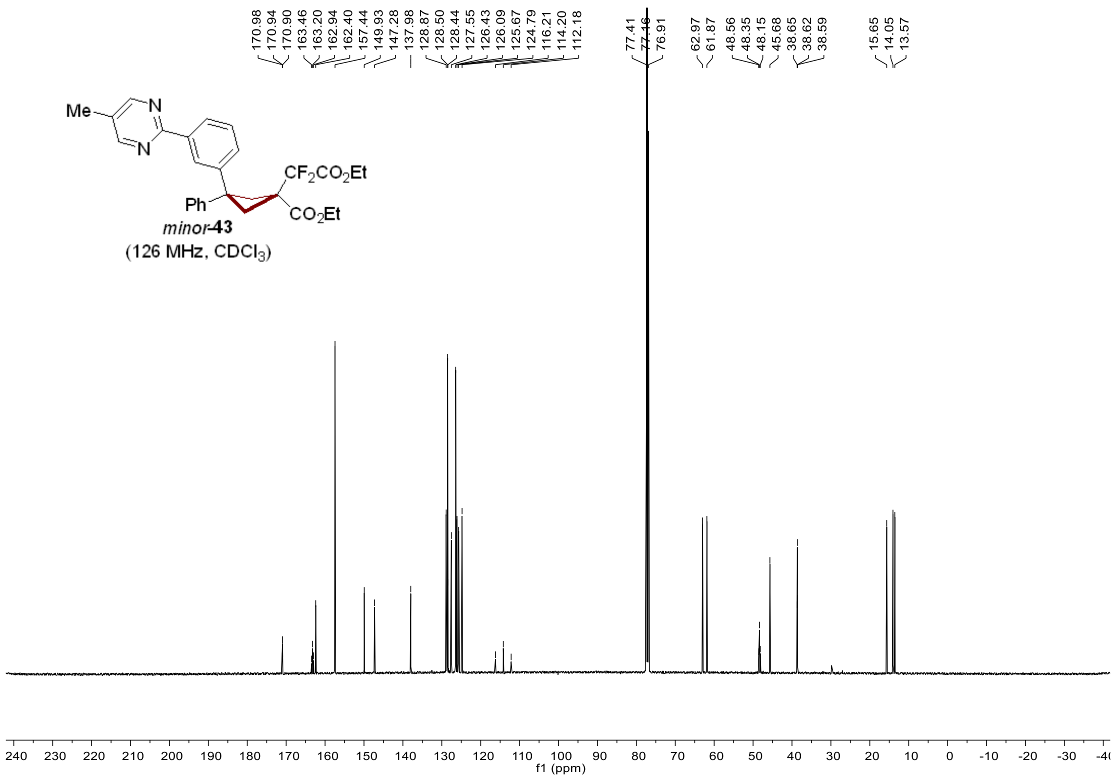

170.98
170.94
170.90
163.46
163.20
162.94
162.40
157.44
149.93
147.28
137.98
128.87
128.50
128.44
127.55
126.43
126.09
125.67
124.79
116.21
114.20
112.18
77.41
77.16
76.91
62.97
61.87
48.56
48.35
48.15
45.68
38.65
38.62
38.59
15.65
14.05
13.57
Me
N
N
CF2CO2Et
Ph
CO2Et
minor-43
(126 MHz, CDCl3)
f1 (ppm)


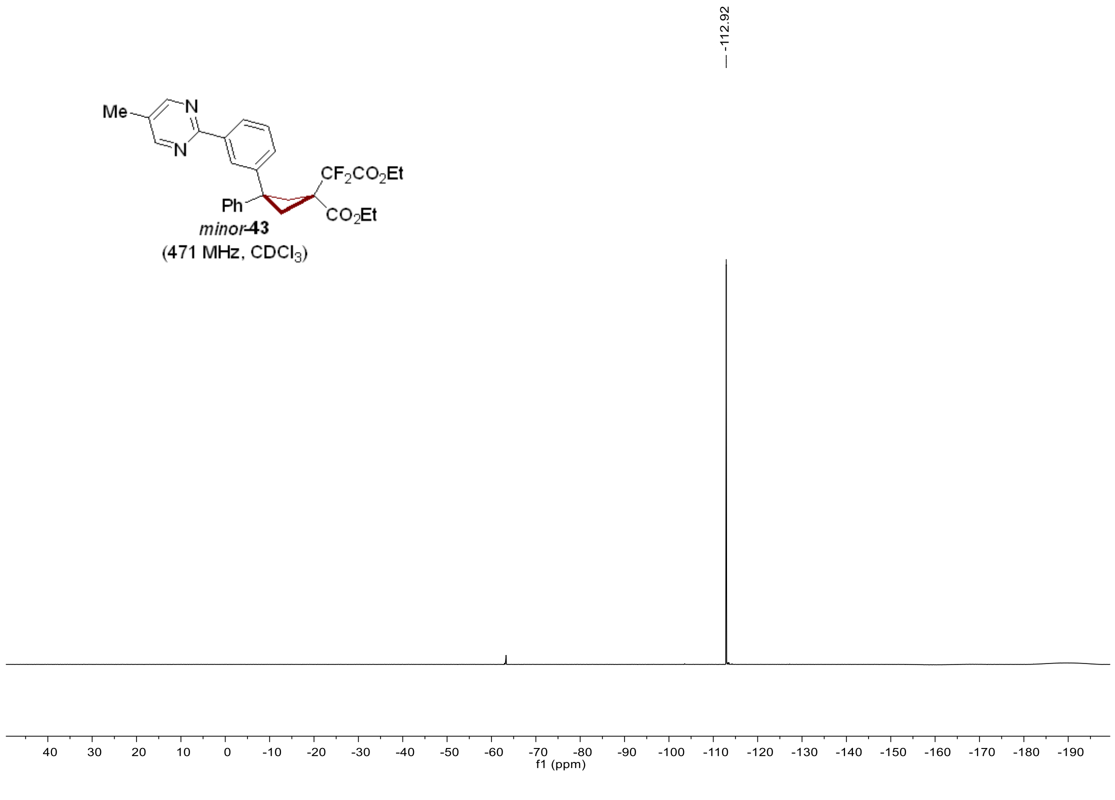

-112.92
Me
N
N
CF2CO2Et
Ph
CO2Et
minor-43
(471 MHz, CDCl3)
f1 (ppm)

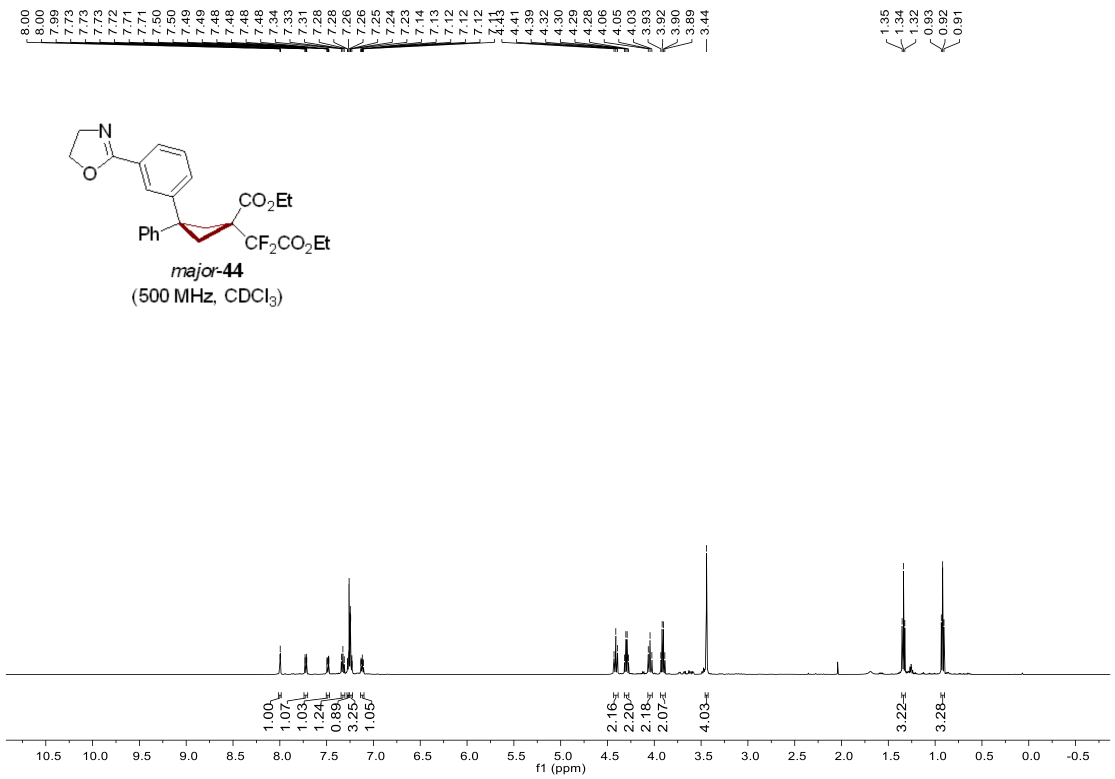
8.00 8.00 7.99 7.73 7.73 7.73 7.72 7.71 7.50 7.50 7.49 7.48 7.48 7.48 7.34 7.33 7.31 7.28 7.28 7.26 7.26 7.25 7.24 7.23 7.14 7.13 7.12 7.12 7.12 7.11 4.43 4.41 4.39 4.32 4.30 4.29 4.28 4.06 4.05 4.03 3.93 3.92 3.90 3.89 3.44
1.35 1.34 1.32 0.93 0.92 0.91
N
O
CO2Et
Ph
CF2CO2Et
major-44
(500 MHz, CDCl3)
1.00 1.07 1.03 1.24 0.89 3.25 1.05
2.16 2.20 2.18 2.07
4.03
3.22
3.28
10.5 10.0 9.5 9.0 8.5 8.0 7.5 7.0 6.5 6.0 5.5 5.0 4.5 4.0 3.5 3.0 2.5 2.0 1.5 1.0 0.5 0.0 -0.5
f1 (ppm)

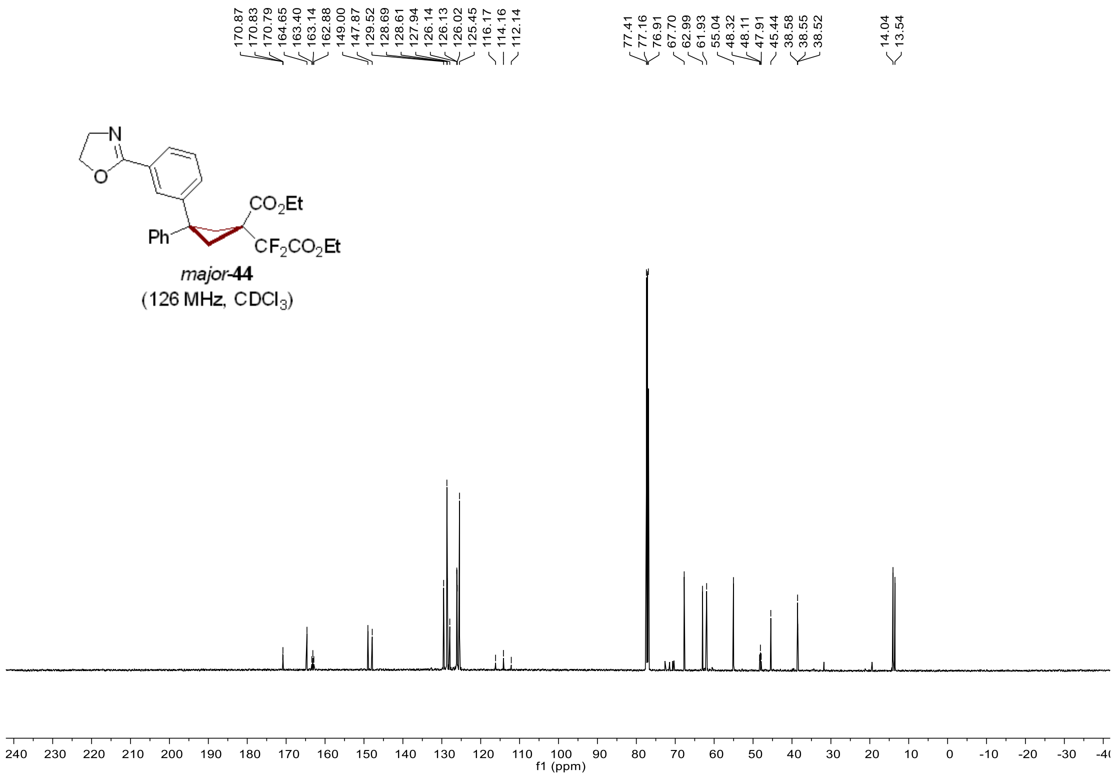
170.87 170.83 170.79 164.65 163.40 163.14 162.88 149.00 147.87 129.52 128.69 128.61 127.94 126.14 126.13 126.02 125.45 116.17 114.16 112.14
77.41 77.16 76.91 67.70 62.99 61.93 55.04 48.32 48.11 47.91 45.44 38.58 38.55 38.52
14.04 13.54
N
O
CO2Et
Ph
CF2CO2Et
major-44
(126 MHz, CDCl3)
240 230 220 210 200 190 180 170 160 150 140 130 120 110 100 90 80 70 60 50 40 30 20 10 0 -10 -20 -30 -40
f1 (ppm)

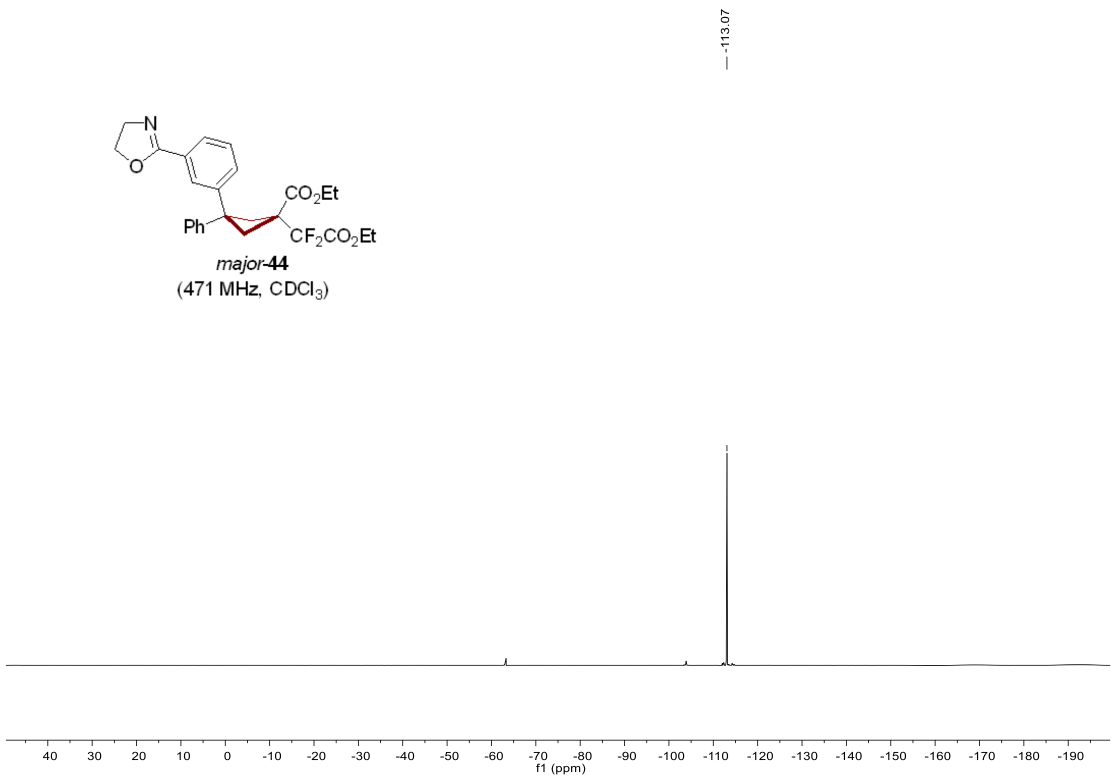
CO2Et
Ph
CF2CO2Et
major-44
(471 MHz, CDCl3)
-113.07
f1 (ppm)

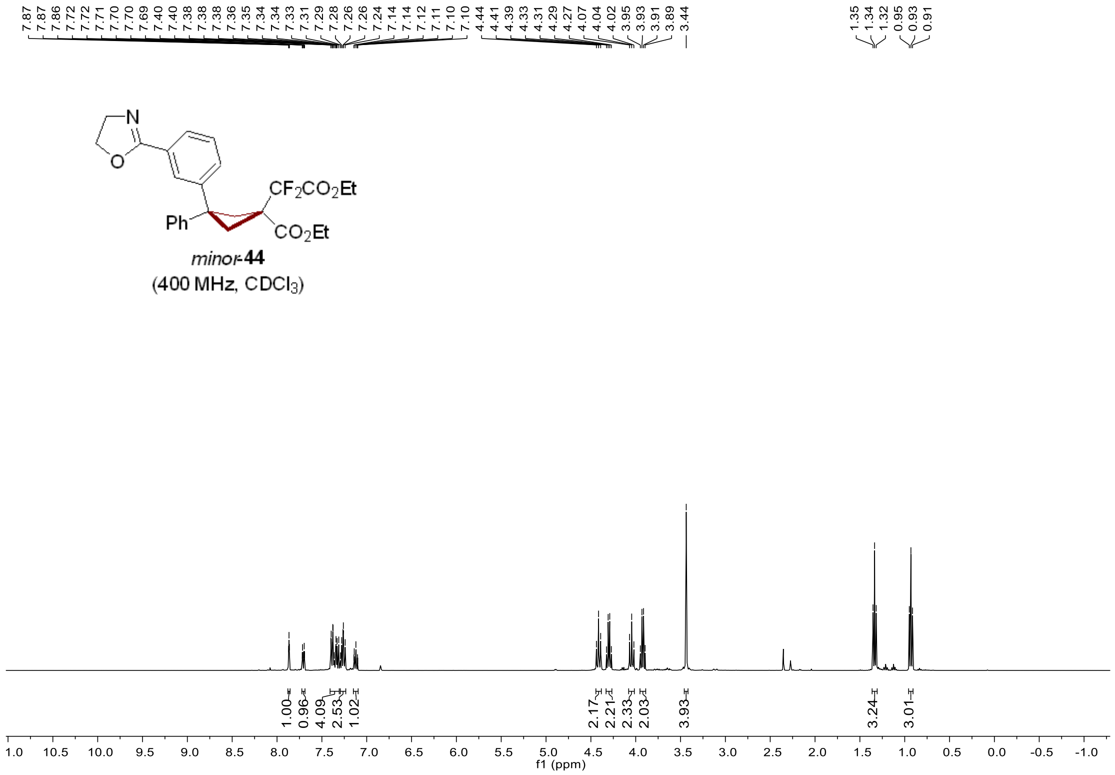
CF2CO2Et
Ph
CO2Et
minor-44
(400 MHz, CDCl3)
f1 (ppm)

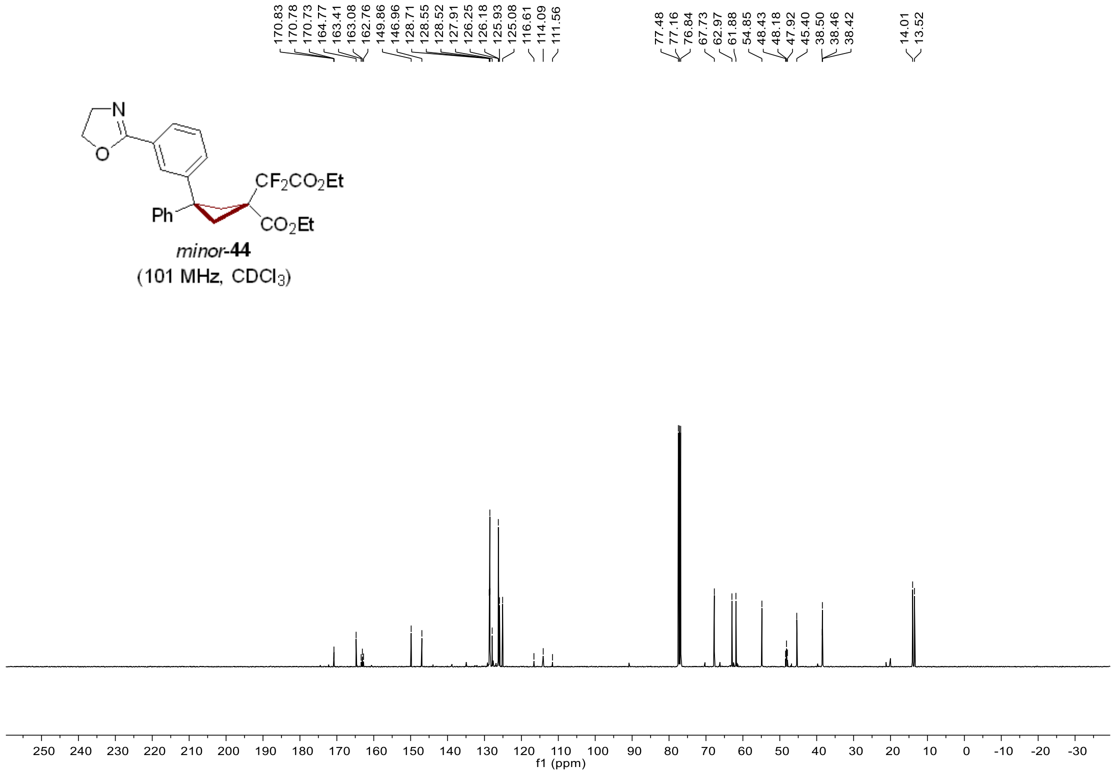
170.83
170.78
170.73
164.77
163.41
163.08
162.76
149.86
146.96
128.71
128.55
128.52
127.91
126.25
126.18
125.93
125.08
116.61
114.09
111.56
77.48
77.16
76.84
67.73
62.97
61.88
54.85
48.43
48.18
47.92
45.40
38.50
38.46
38.42
14.01
13.52
N
O
CF2CO2Et
Ph
CO2Et
minor-44
(101 MHz, CDCl3)
f1 (ppm)

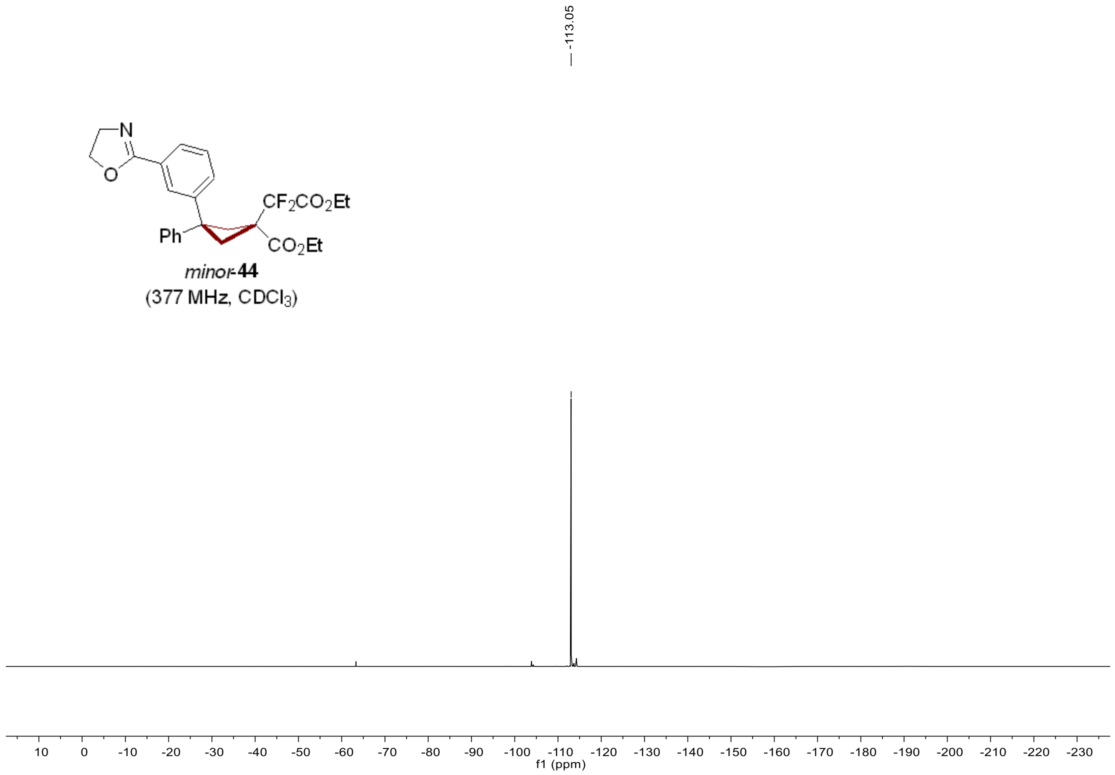
-113.05
N
O
CF2CO2Et
Ph
CO2Et
minor-44
(377 MHz, CDCl3)
f1 (ppm)

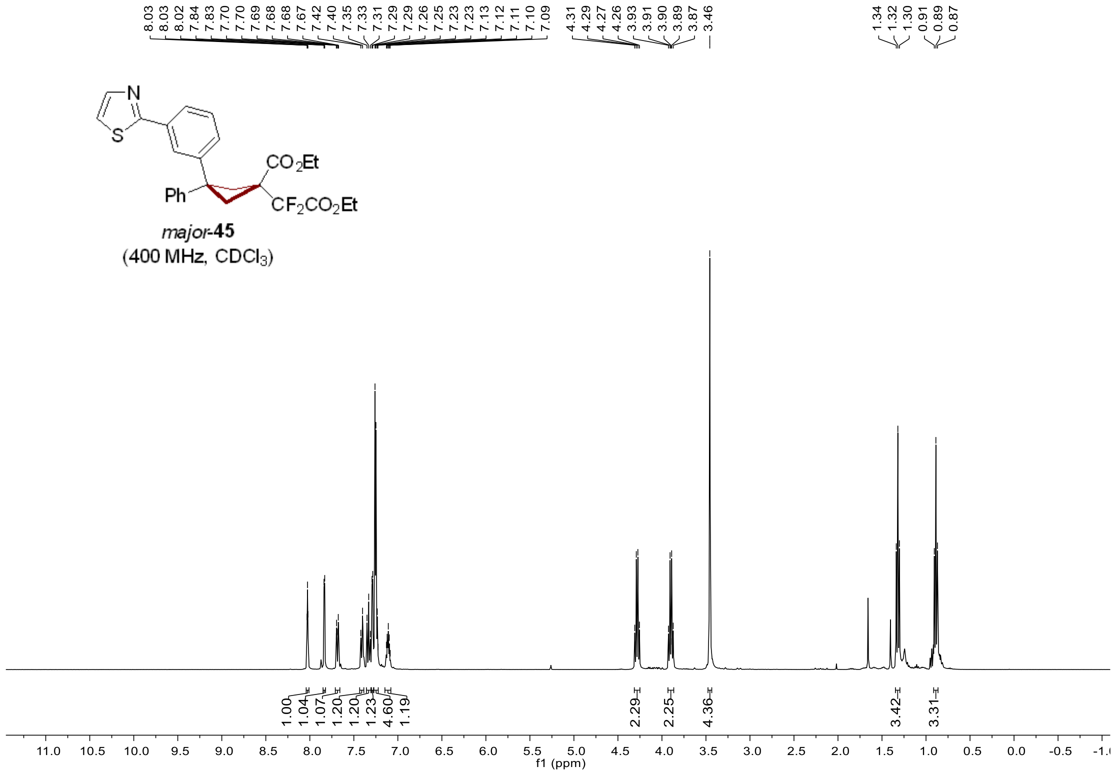
8.03 8.03 8.02 7.84 7.83 7.70 7.70 7.69 7.68 7.68 7.67 7.42 7.40 7.35 7.33 7.31 7.29 7.29 7.26 7.25 7.23 7.23 7.13 7.12 7.11 7.10 7.09
4.31 4.29 4.27 4.26 3.93 3.91 3.90 3.89 3.87 3.46
1.34 1.32 1.30 0.91 0.89 0.87
N
S
CO2Et
Ph
CF2CO2Et
major-45
(400 MHz, CDCl3)
1.00 1.04 1.07 1.20 1.20 1.23 4.60 1.19
2.29 2.25 4.36
3.42 3.31
11.0 10.5 10.0 9.5 9.0 8.5 8.0 7.5 7.0 6.5 6.0 5.5 5.0 4.5 4.0 3.5 3.0 2.5 2.0 1.5 1.0 0.5 0.0 -0.5
f1 (ppm)

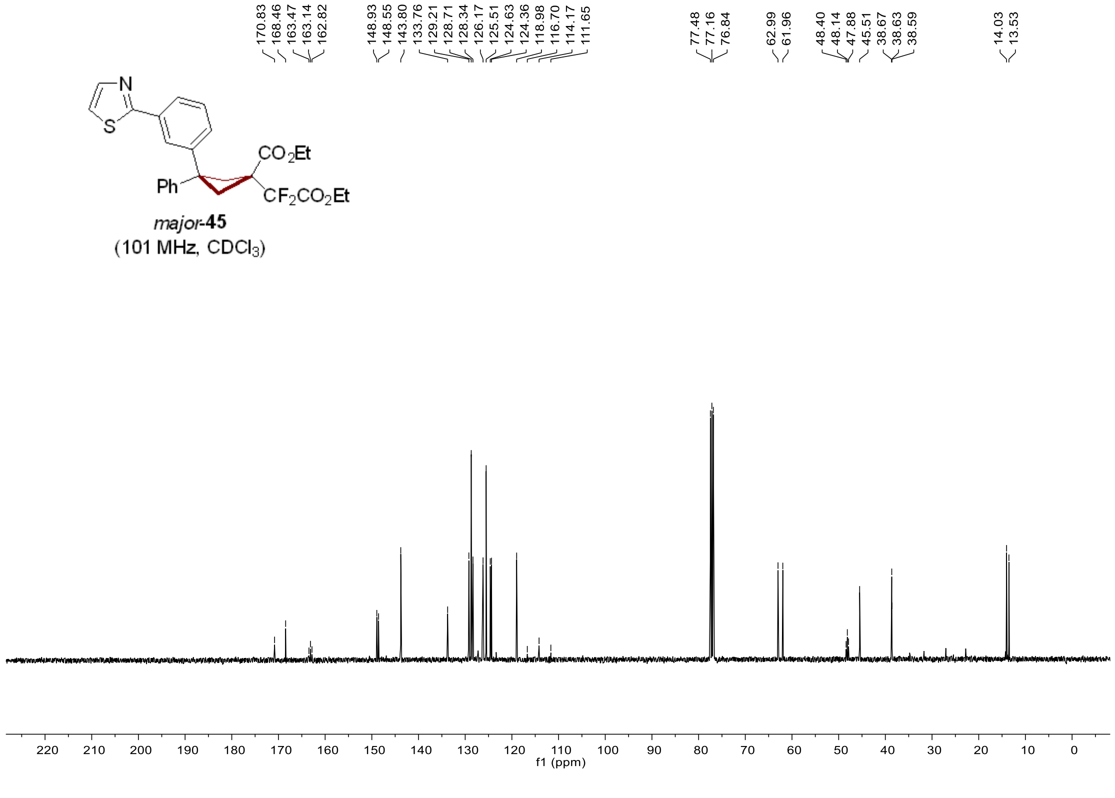
170.83 168.46 163.47 163.14 162.82
148.93 148.55 143.80 133.76 129.21 128.71 128.34 126.17 125.51 124.63 124.36 118.98 116.70 114.17 111.65
77.48 77.16 76.84
62.99 61.96
48.40 48.14 47.88 45.51 38.67 38.63 38.59
14.03 13.53
N
S
CO2Et
Ph
CF2CO2Et
major-45
(101 MHz, CDCl3)
220 210 200 190 180 170 160 150 140 130 120 110 100 90 80 70 60 50 40 30 20 10 0
f1 (ppm)

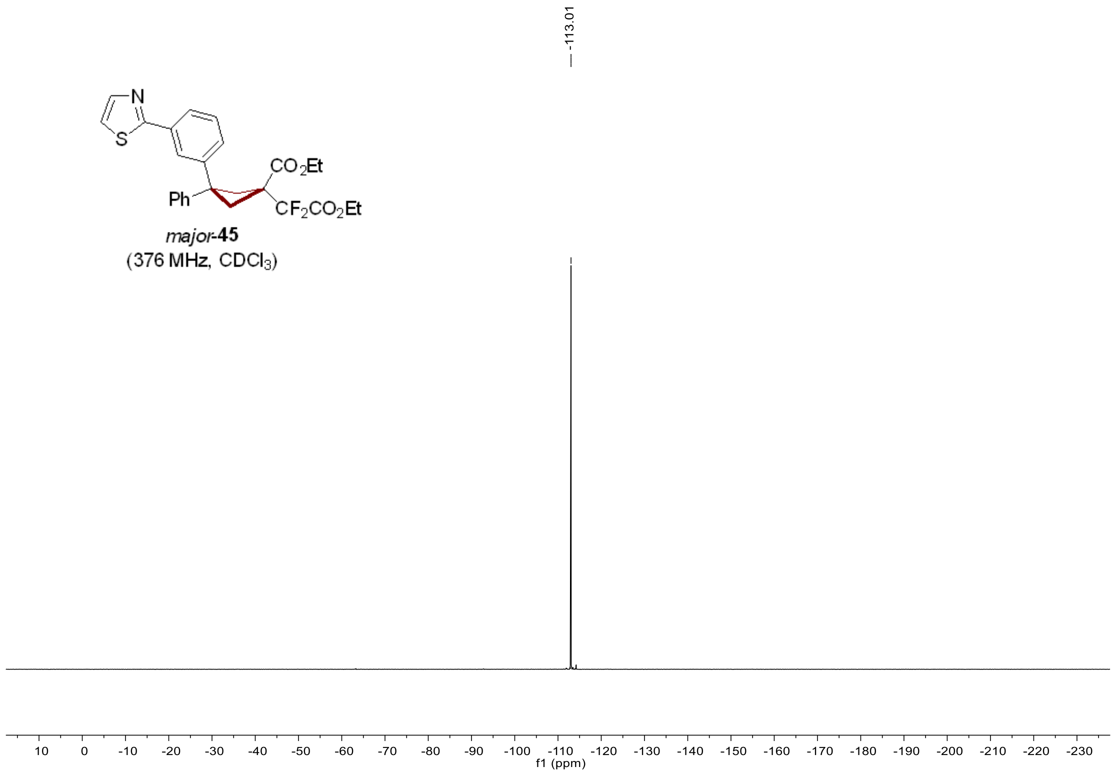

major-45
(376 MHz, CDCl3)
-113.01
f1 (ppm)


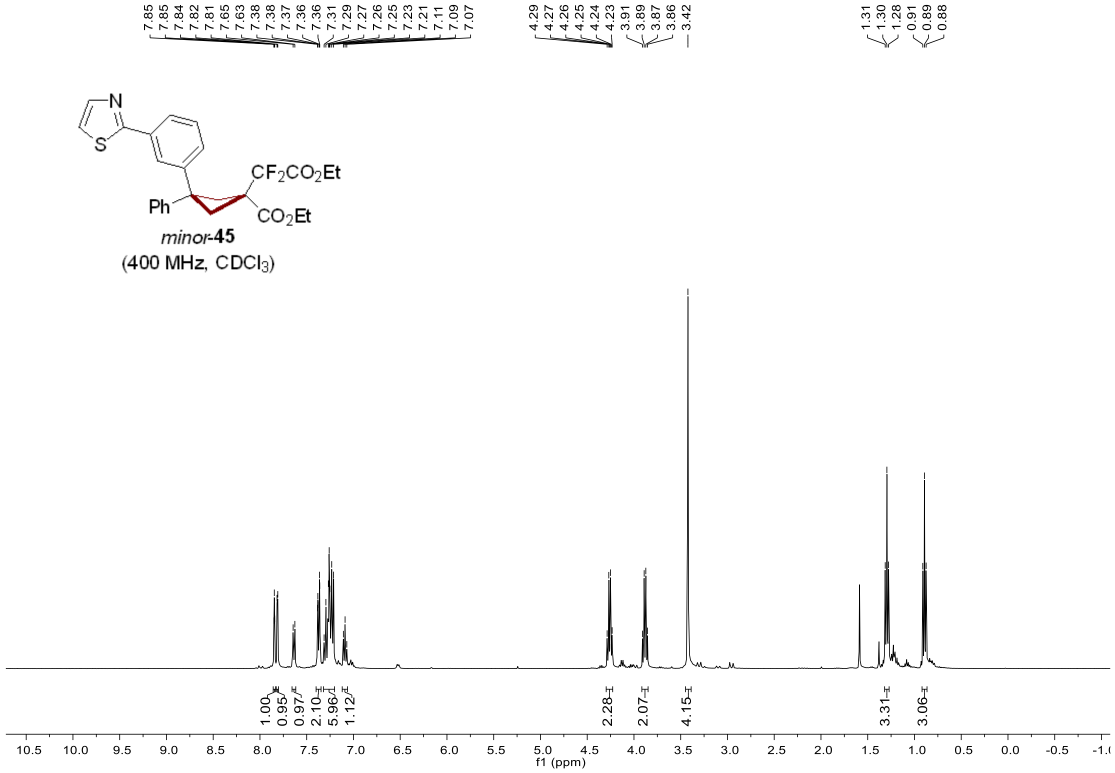

minor-45
(400 MHz, CDCl3)
7.85 7.85 7.84 7.82 7.81 7.65 7.63 7.38 7.38 7.37 7.36 7.36 7.31 7.29 7.27 7.26 7.25 7.23 7.21 7.11 7.09 7.07
4.29 4.27 4.26 4.25 4.24 4.23 3.91 3.89 3.87 3.86 3.42
1.31 1.30 1.28 0.91 0.89 0.88
1.00 0.95 0.97 2.10 5.96 1.12 2.28 2.07 4.15 3.31 3.06
f1 (ppm)

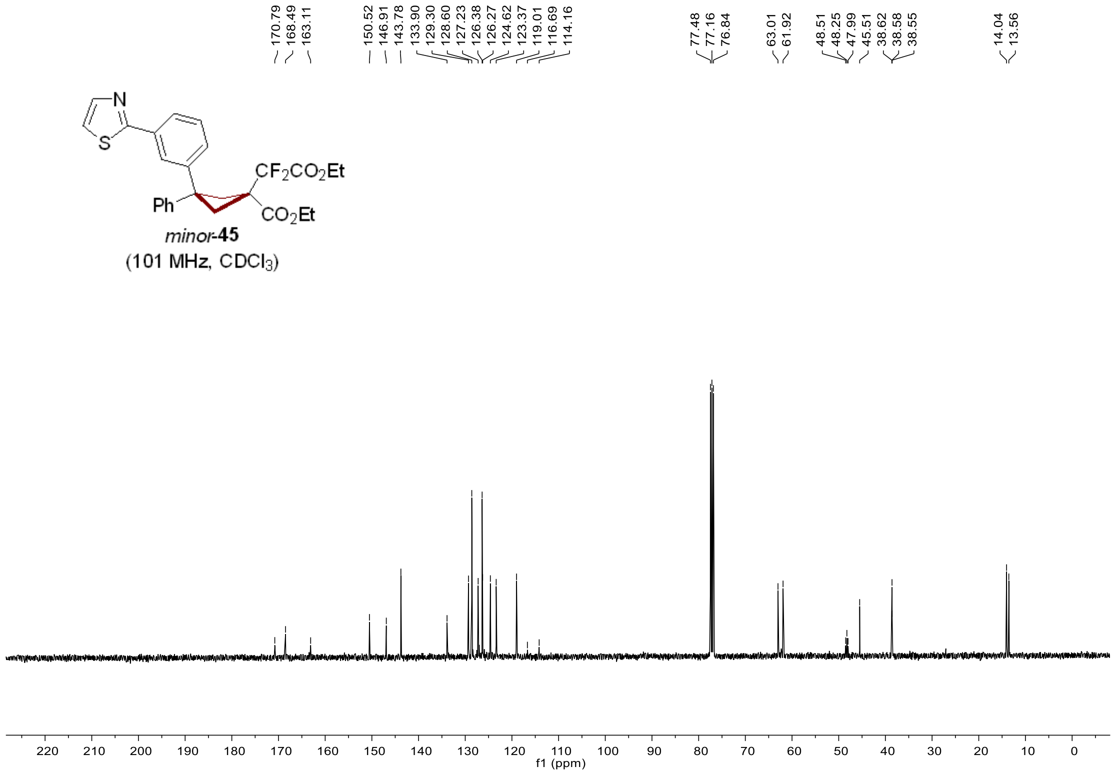
170.79
168.49
163.11
150.52
146.91
143.78
133.90
129.30
128.60
127.23
126.38
126.27
124.62
123.37
119.01
116.69
114.16
77.48
77.16
76.84
63.01
61.92
48.51
48.25
47.99
45.51
38.62
38.58
38.55
14.04
13.56
N
S
Ph
$CF_2CO_2Et$
$CO_2Et$
*minor*-**45**
(101 MHz, $CDCl_3$)
220 210 200 190 180 170 160 150 140 130 120 110 100 90 80 70 60 50 40 30 20 10 0
f1 (ppm)

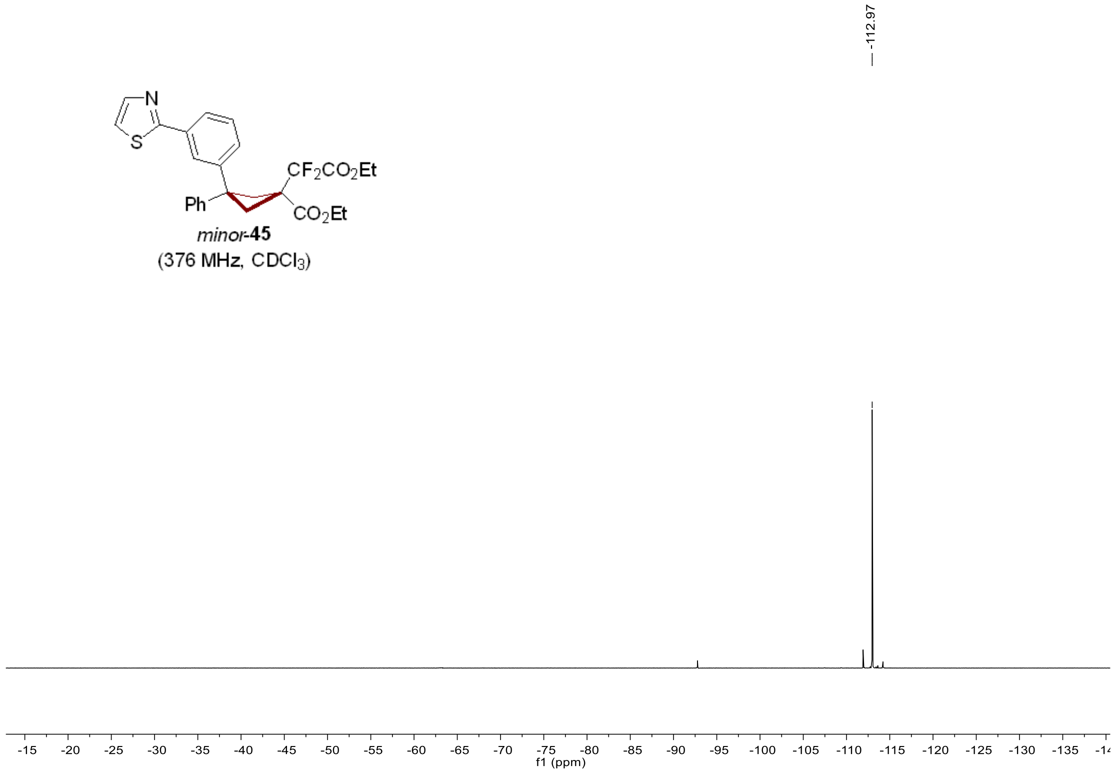
-112.97
N
S
Ph
$CF_2CO_2Et$
$CO_2Et$
*minor*-**45**
(376 MHz, $CDCl_3$)
-15 -20 -25 -30 -35 -40 -45 -50 -55 -60 -65 -70 -75 -80 -85 -90 -95 -100 -105 -110 -115 -120 -125 -130 -135
f1 (ppm)

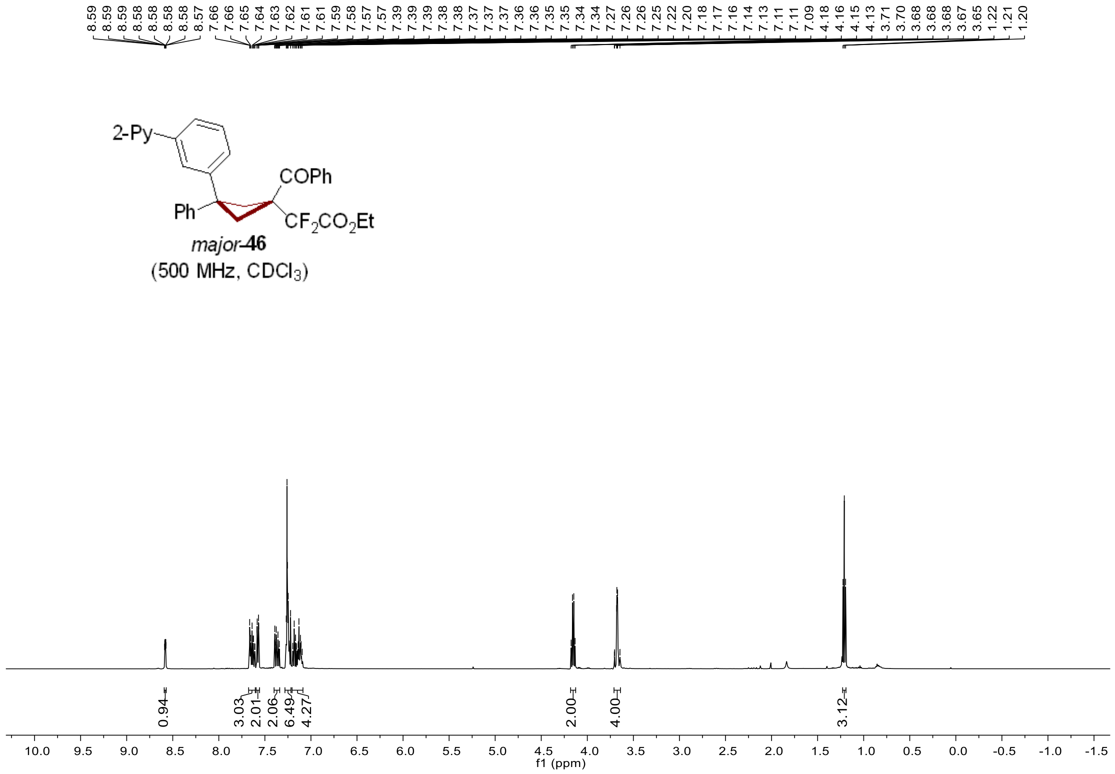
8.59 8.59 8.59 8.58 8.58 8.58 8.57 7.66 7.65 7.64 7.63 7.62 7.61 7.61 7.59 7.58 7.57 7.57 7.39 7.39 7.39 7.38 7.38 7.37 7.37 7.37 7.36 7.36 7.35 7.35 7.34 7.34 7.27 7.26 7.26 7.25 7.22 7.20 7.18 7.17 7.16 7.14 7.13 7.11 7.11 7.09 4.18 4.16 4.15 4.13 3.71 3.70 3.68 3.68 3.68 3.67 3.65 1.22 1.21 1.20
2-Py
COPh
Ph
$CF_2CO_2Et$
*major*-**46**
(500 MHz, $CDCl_3$)
0.94
3.03
2.01
2.06
6.49
4.27
2.00
4.00
3.12
10.0 9.5 9.0 8.5 8.0 7.5 7.0 6.5 6.0 5.5 5.0 4.5 4.0 3.5 3.0 2.5 2.0 1.5 1.0 0.5 0.0 -0.5 -1.0 -1.5
f1 (ppm)

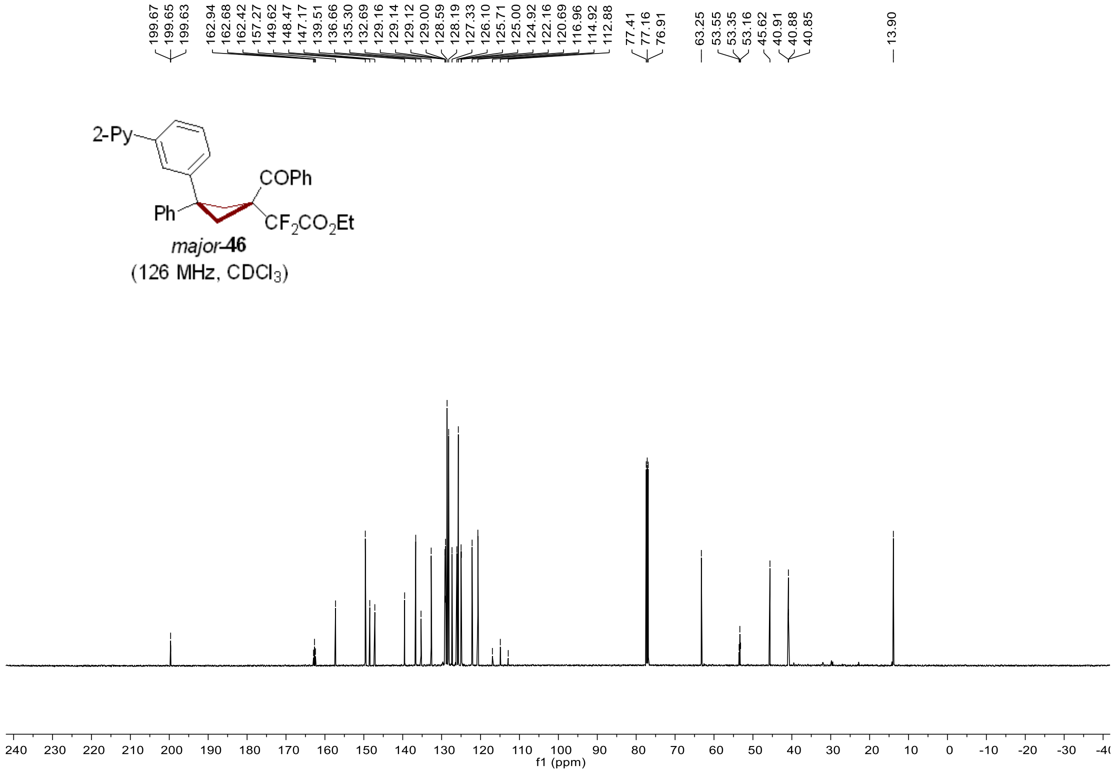
199.67 199.65 199.63 162.94 162.68 162.42 157.27 149.62 148.47 147.17 139.51 136.66 135.30 132.69 129.16 129.14 129.12 129.00 128.59 128.19 127.33 126.10 125.71 125.00 124.92 122.16 120.69 116.96 114.92 112.88 77.41 77.16 76.91 63.25 53.55 53.35 53.16 45.62 40.91 40.88 40.85 13.90
2-Py
COPh
Ph
$CF_2CO_2Et$
*major*-**46**
(126 MHz, $CDCl_3$)
240 230 220 210 200 190 180 170 160 150 140 130 120 110 100 90 80 70 60 50 40 30 20 10 0 -10 -20 -30 -4
f1 (ppm)

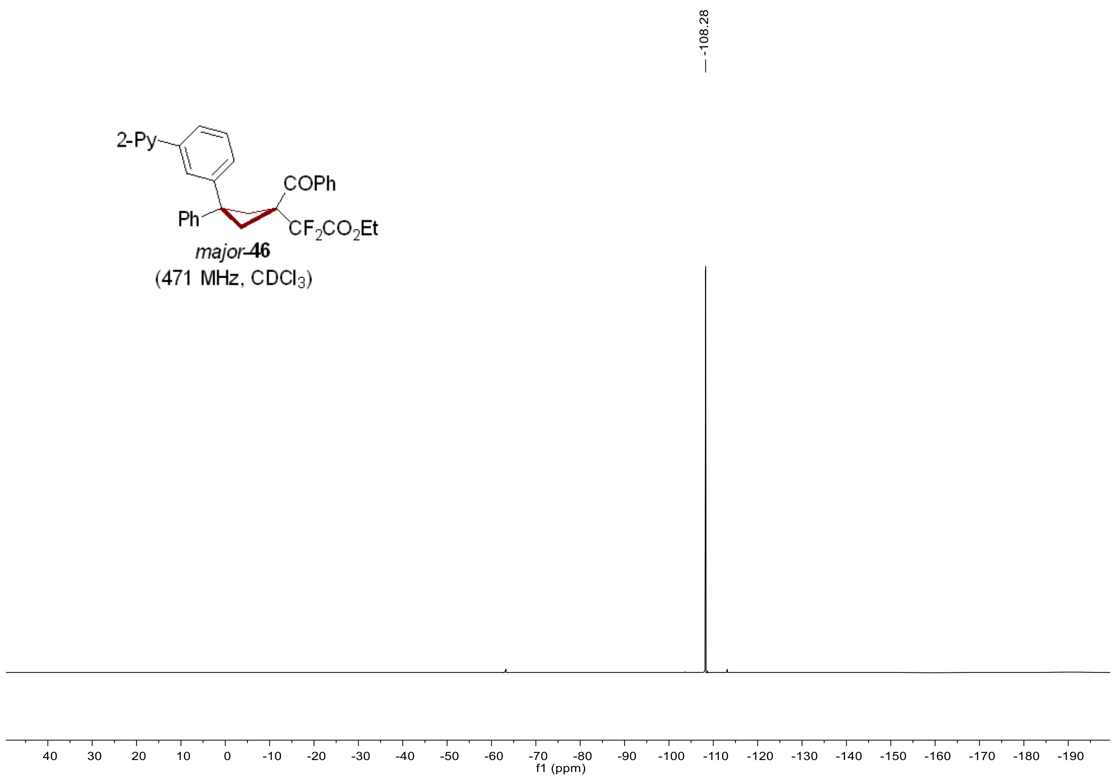
-108.28
2-Py
COPh
Ph
$CF_2CO_2Et$
*major*-**46**
(471 MHz, $CDCl_3$)
f1 (ppm)

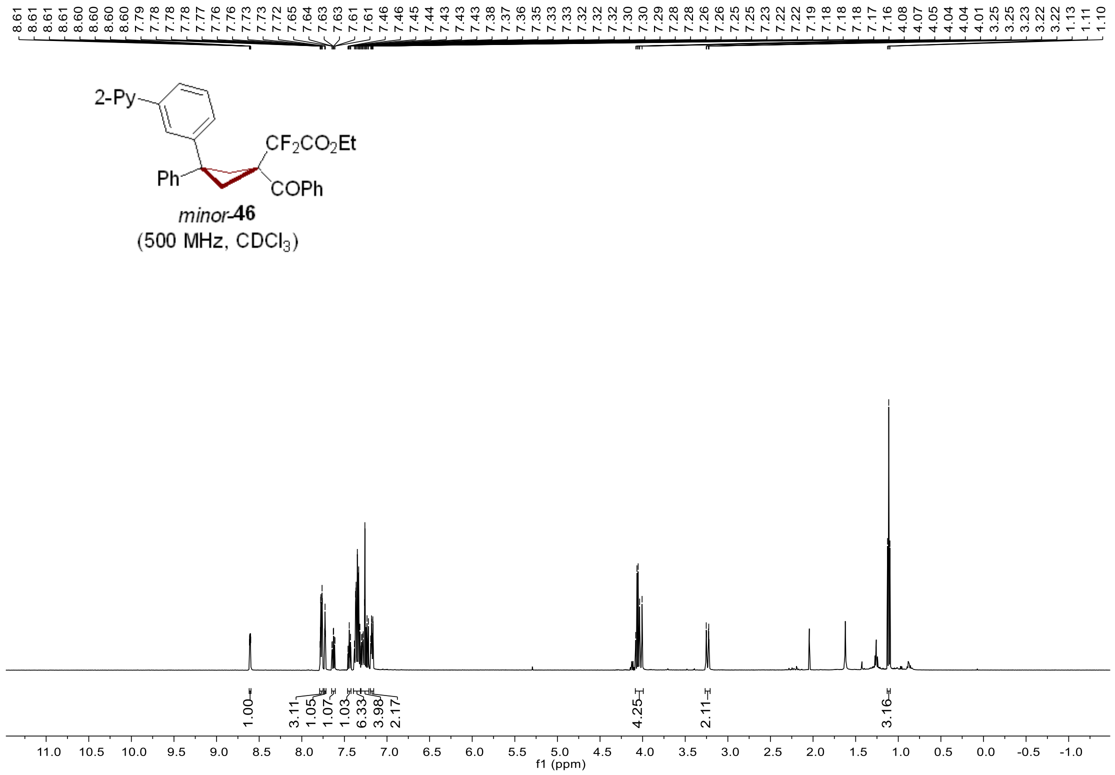
2-Py
$CF_2CO_2Et$
Ph
COPh
*minor*-**46**
(500 MHz, $CDCl_3$)
f1 (ppm)

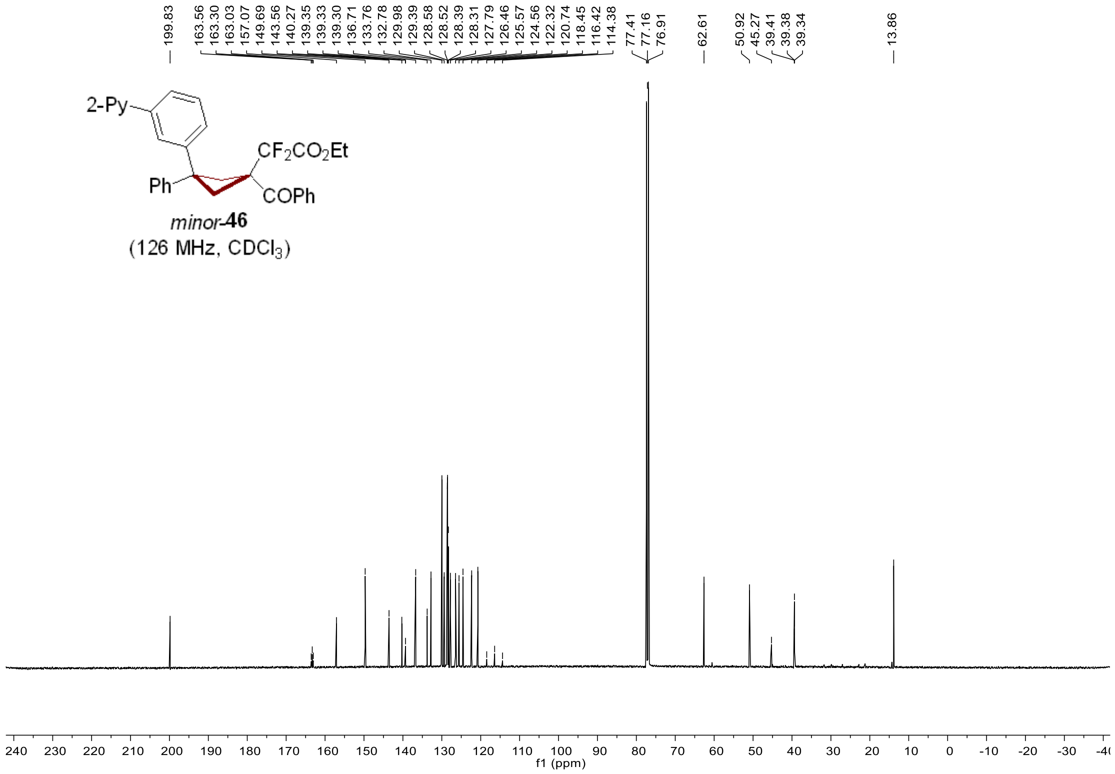

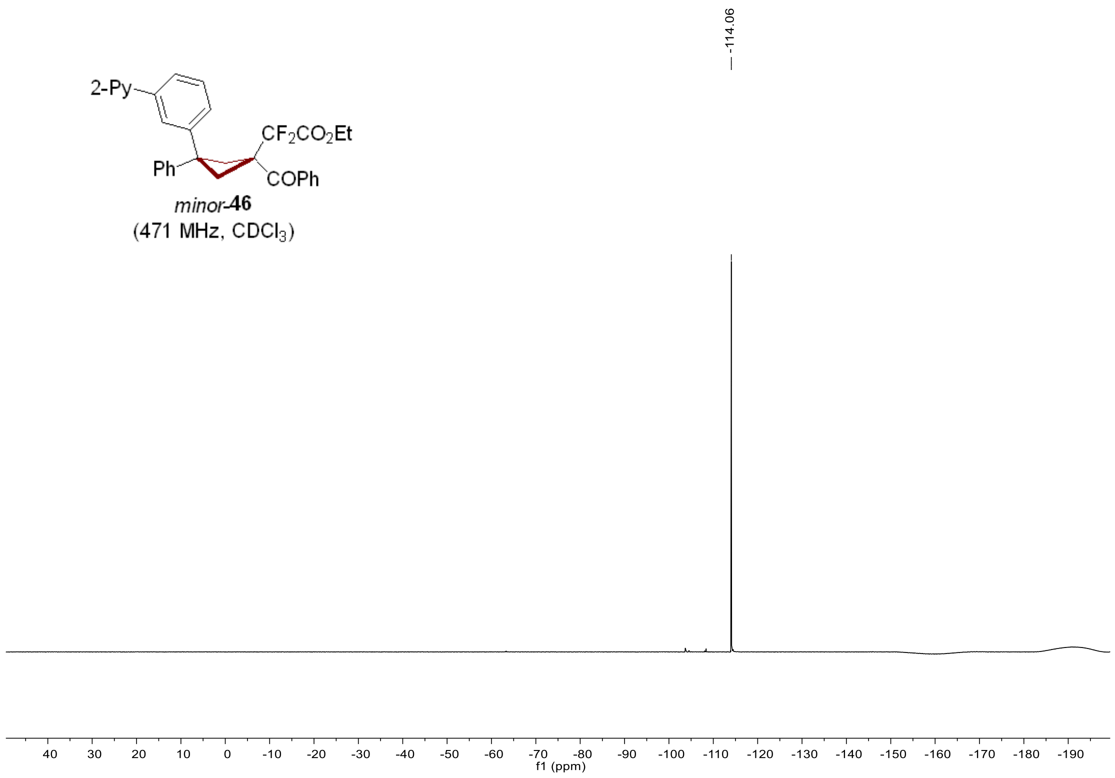

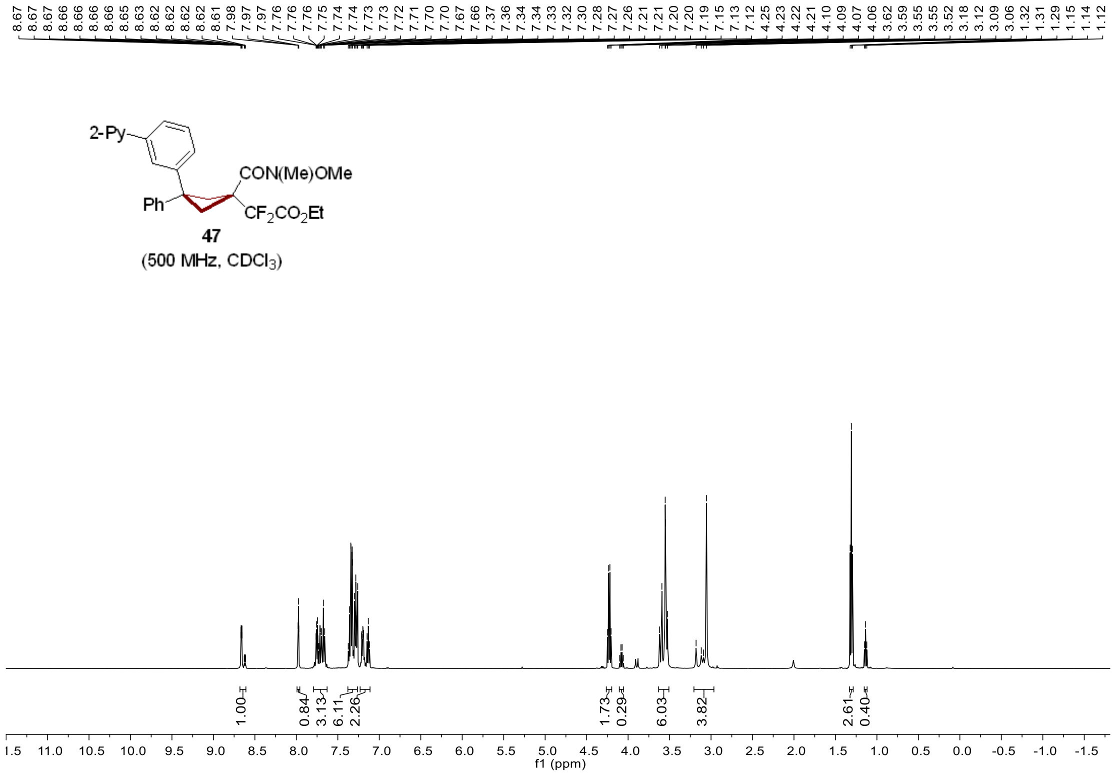
8.67 8.67 8.66 8.66 8.66 8.65 8.63 8.62 8.62 8.62 8.61 7.98 7.97 7.76 7.76 7.75 7.74 7.74 7.73 7.73 7.72 7.71 7.70 7.67 7.66 7.37 7.36 7.34 7.33 7.32 7.30 7.28 7.27 7.26 7.21 7.21 7.20 7.19 7.15 7.13 7.12 4.25 4.23 4.22 4.21 4.10 4.09 4.07 4.06 3.62 3.59 3.55 3.55 3.52 3.18 3.12 3.09 3.06 1.32 1.31 1.29 1.15 1.14 1.12
2-Py
CON(Me)OMe
Ph
CF2CO2Et
47
(500 MHz, CDCl3)
1.00 0.84 3.13 6.11 2.26 1.73 0.29 6.03 3.82 2.61 0.40
f1 (ppm)

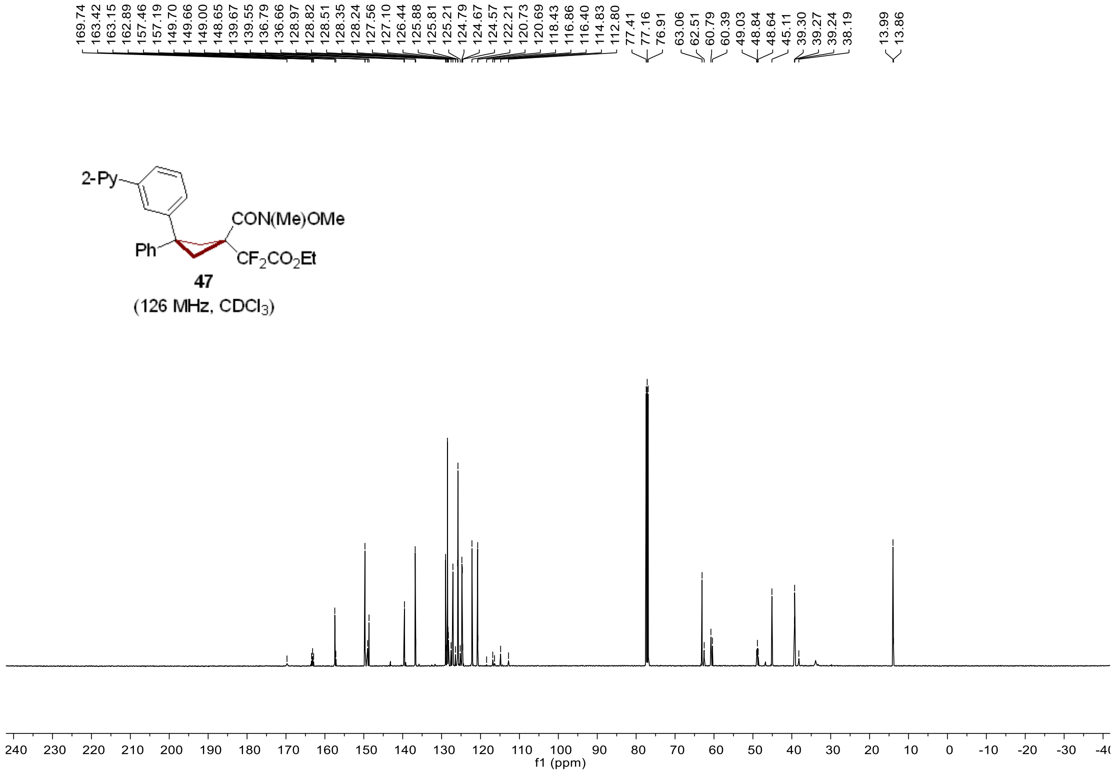
169.74 163.42 163.15 162.89 157.46 157.19 149.70 149.66 149.00 148.65 139.67 139.55 136.79 136.66 128.97 128.82 128.51 128.35 128.24 127.56 127.10 126.44 125.88 125.81 125.21 124.79 124.67 124.57 122.21 120.73 120.69 118.43 116.86 116.40 114.83 112.80 77.41 77.16 76.91 63.06 62.51 60.79 60.39 49.03 48.84 48.64 45.11 39.30 39.27 39.24 38.19 13.99 13.86
2-Py
CON(Me)OMe
Ph
CF2CO2Et
47
(126 MHz, CDCl3)
f1 (ppm)

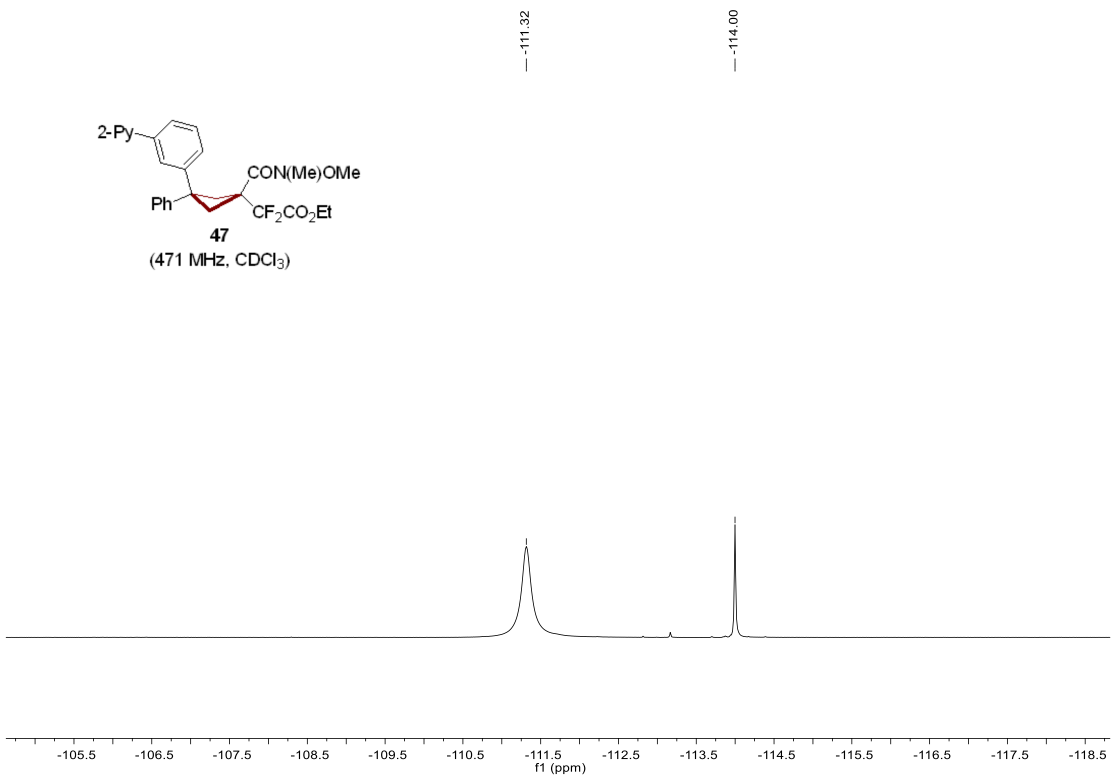


**47**
(471 MHz, $CDCl_3$)

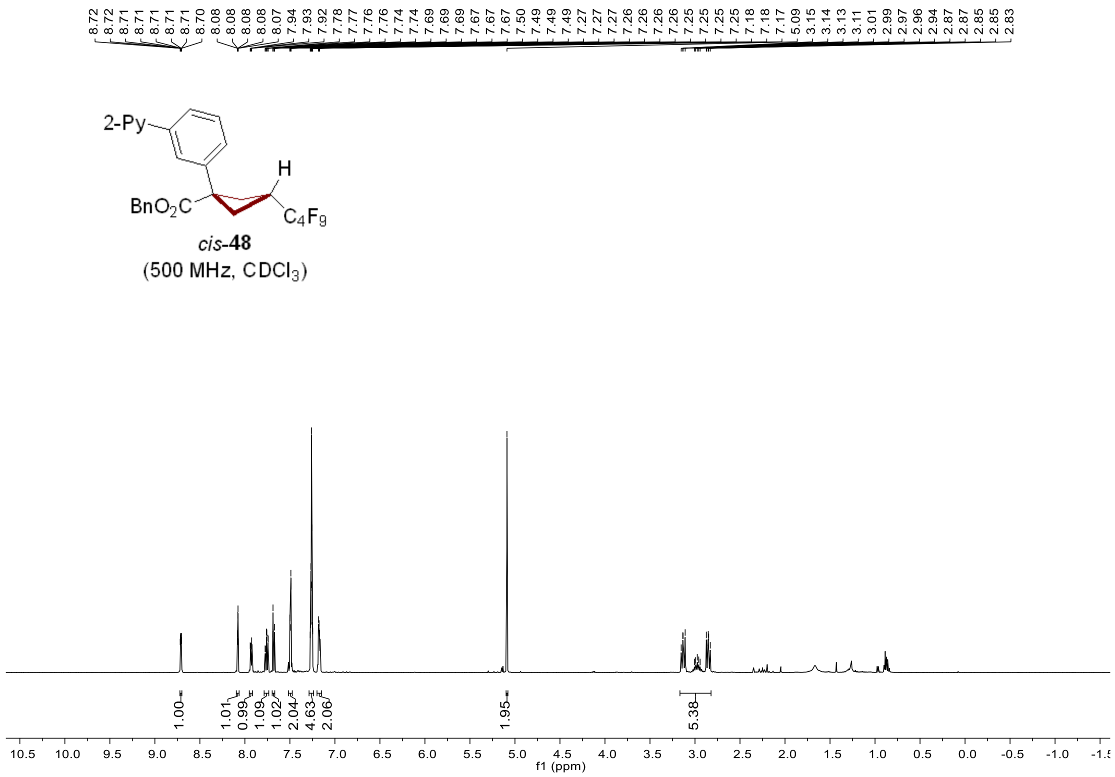


*cis*-**48**
(500 MHz, $CDCl_3$)

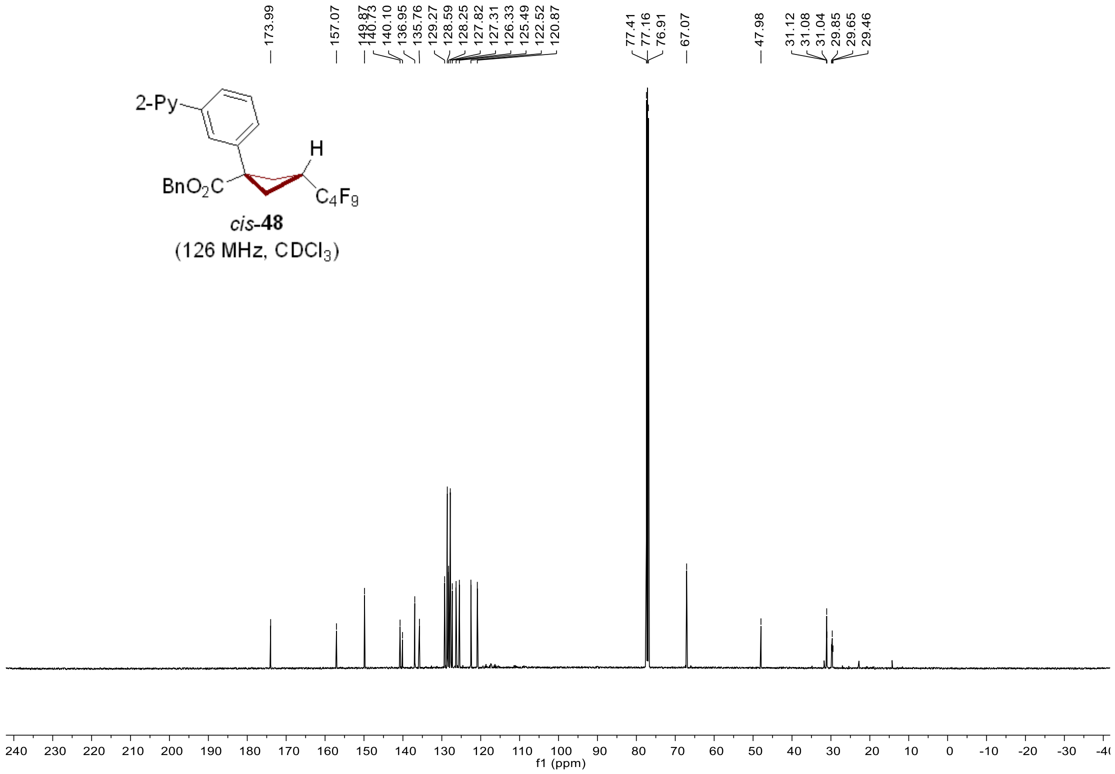
173.99
157.07
149.87
140.73
140.10
136.95
135.76
129.27
128.59
128.25
127.82
127.31
126.33
125.49
122.52
120.87
77.41
77.16
76.91
67.07
47.98
31.12
31.08
31.04
29.85
29.65
29.46
2-Py
H
$BnO_2C$
$C_4F_9$
*cis*-**48**
(126 MHz, $CDCl_3$)
f1 (ppm)

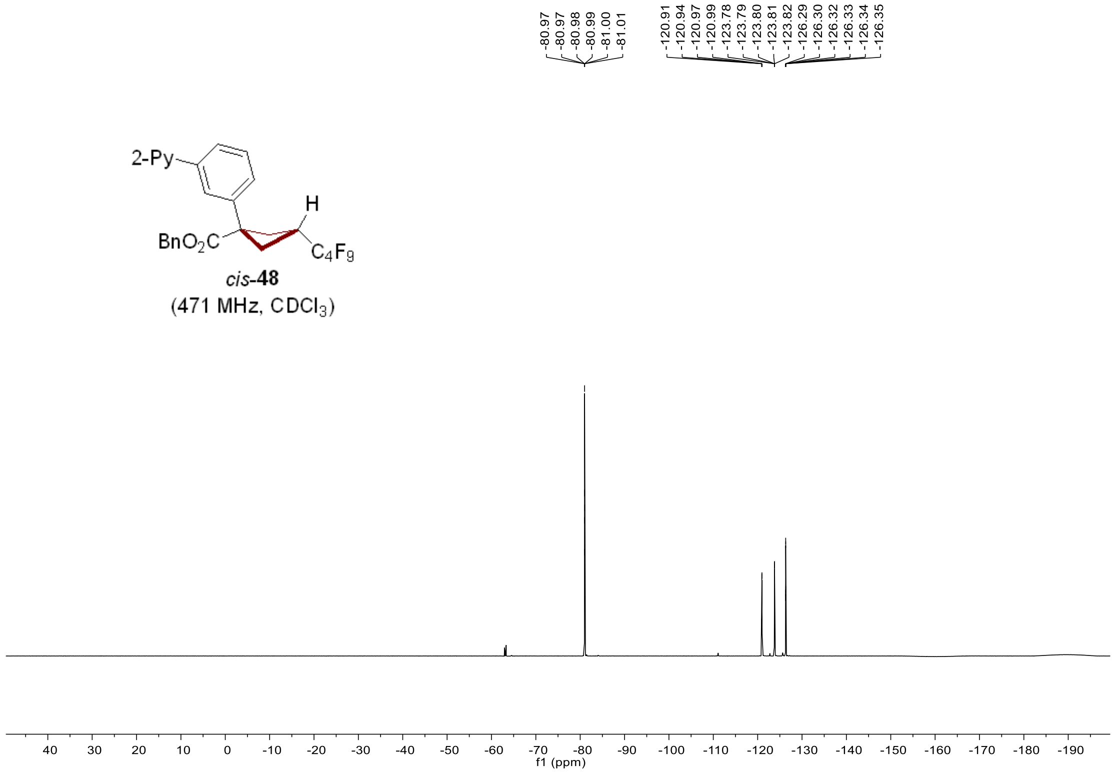
-80.97
-80.97
-80.98
-80.99
-81.00
-81.01
-120.91
-120.94
-120.97
-120.99
-123.78
-123.79
-123.80
-123.81
-123.82
-126.29
-126.30
-126.32
-126.33
-126.34
-126.35
2-Py
H
$BnO_2C$
$C_4F_9$
*cis*-**48**
(471 MHz, $CDCl_3$)
f1 (ppm)

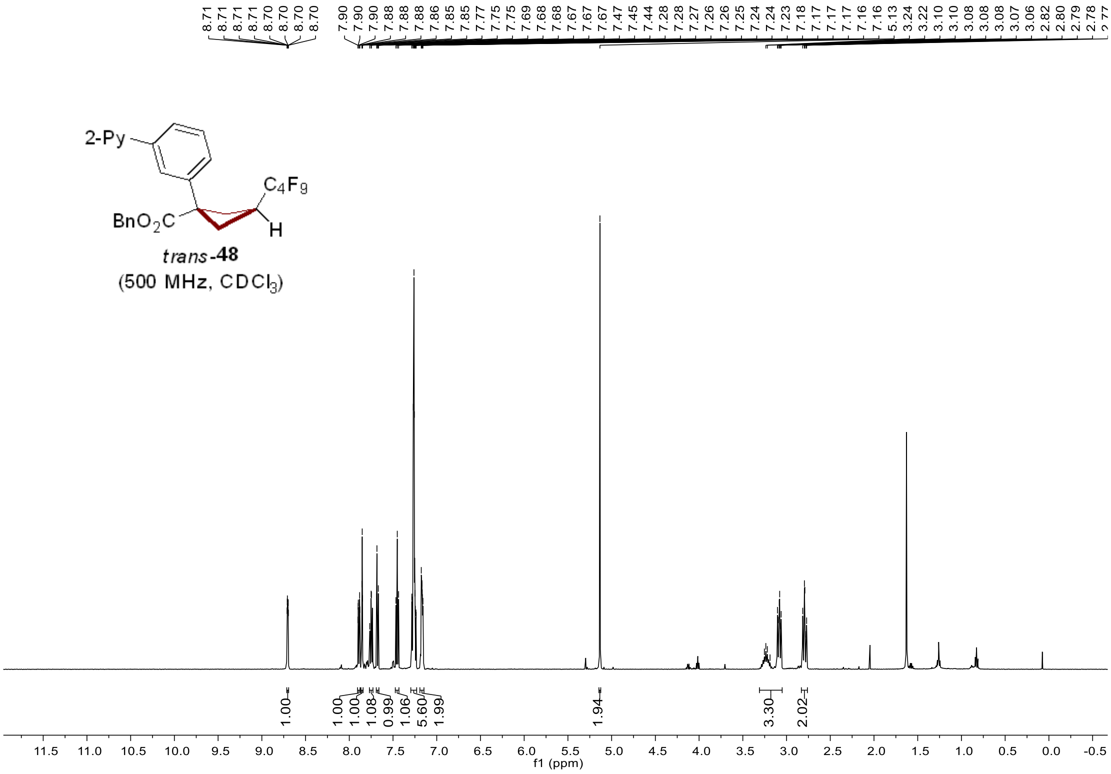
8.71 8.71 8.71 8.70 8.70 8.70 8.70 7.90 7.90 7.90 7.88 7.88 7.86 7.85 7.85 7.77 7.75 7.75 7.69 7.68 7.68 7.67 7.67 7.67 7.47 7.45 7.44 7.28 7.28 7.27 7.26 7.26 7.25 7.24 7.24 7.23 7.18 7.17 7.17 7.17 7.16 5.13 3.24 3.22 3.10 3.10 3.08 3.08 3.07 3.06 2.82 2.80 2.79 2.78
2-Py
C4F9
BnO2C
H
trans-48
(500 MHz, CDCl3)
1.00 1.00 1.00 1.08 0.99 1.06 5.60 1.99 1.94 3.30 2.02
f1 (ppm)

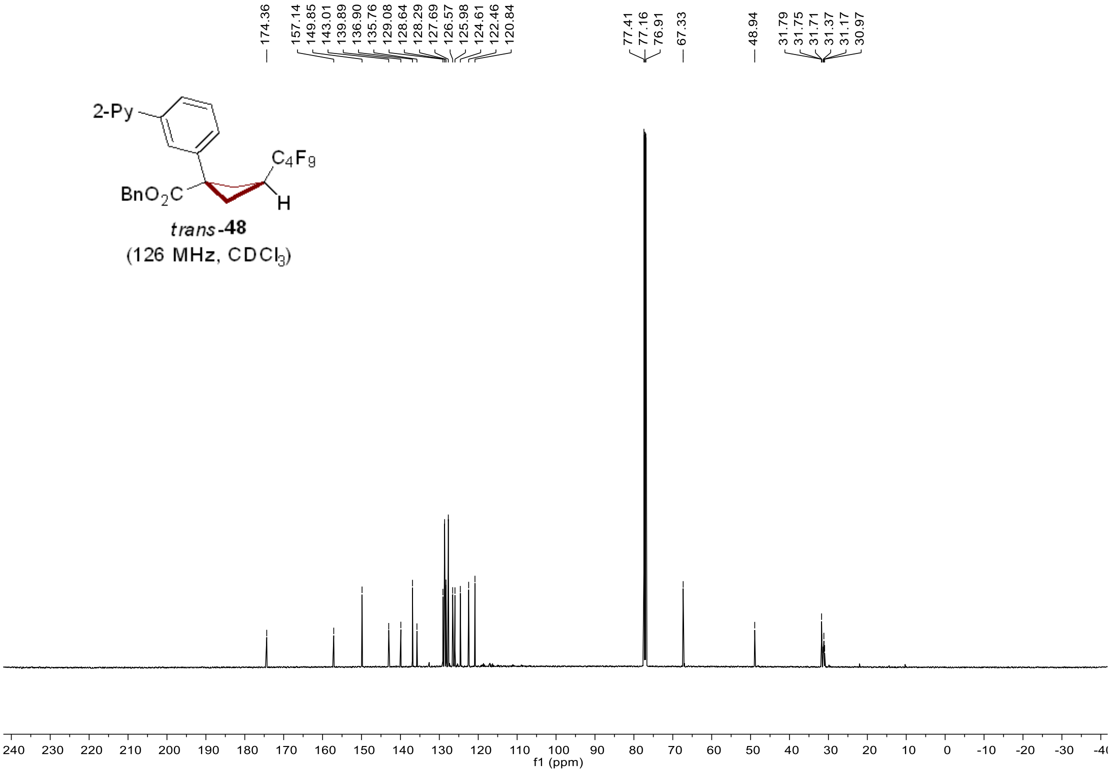
174.36 157.14 149.85 143.01 139.89 136.90 135.76 129.08 128.64 128.29 127.69 126.57 125.98 124.61 122.46 120.84 77.41 77.16 76.91 67.33 48.94 31.79 31.75 31.71 31.37 31.17 30.97
2-Py
C4F9
BnO2C
H
trans-48
(126 MHz, CDCl3)
f1 (ppm)

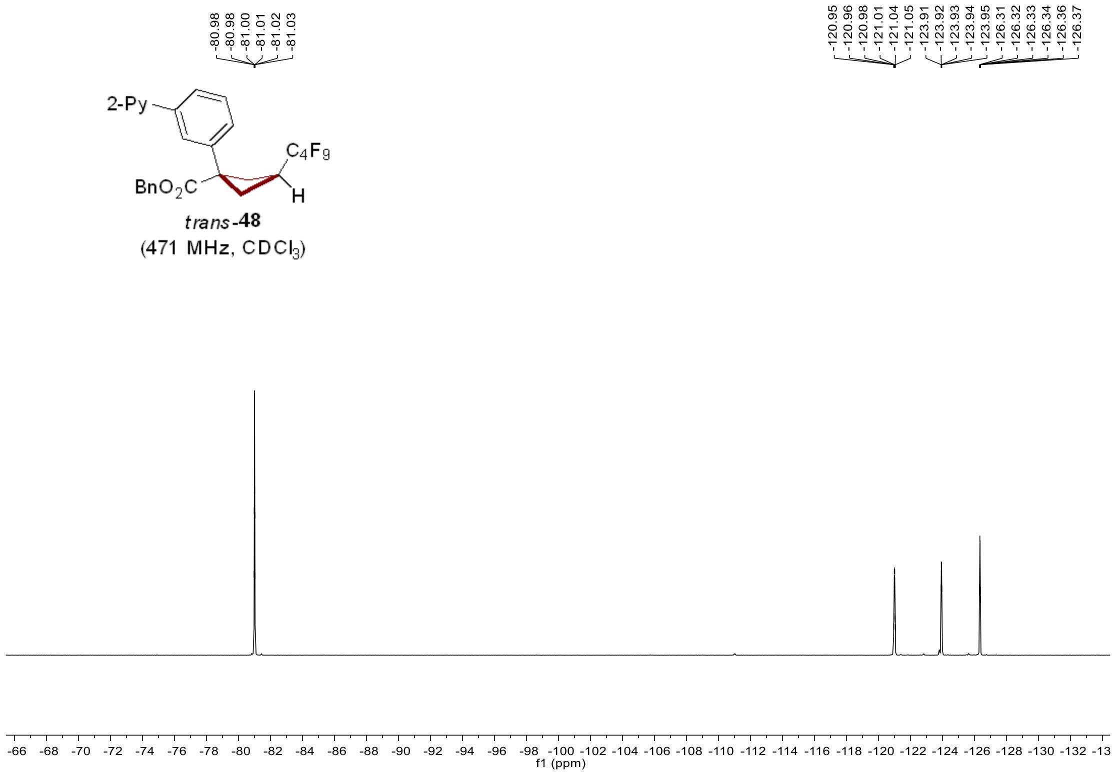


*trans*-**48**
(471 MHz, $CDCl_3$)

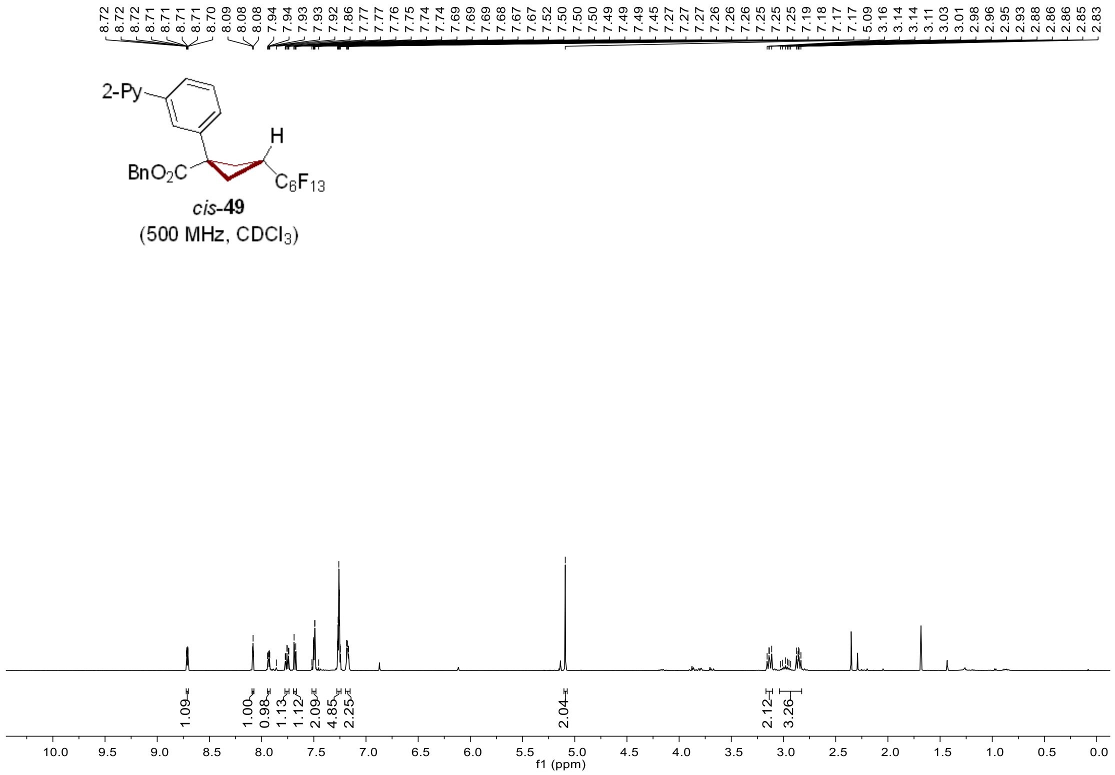


*cis*-**49**
(500 MHz, $CDCl_3$)

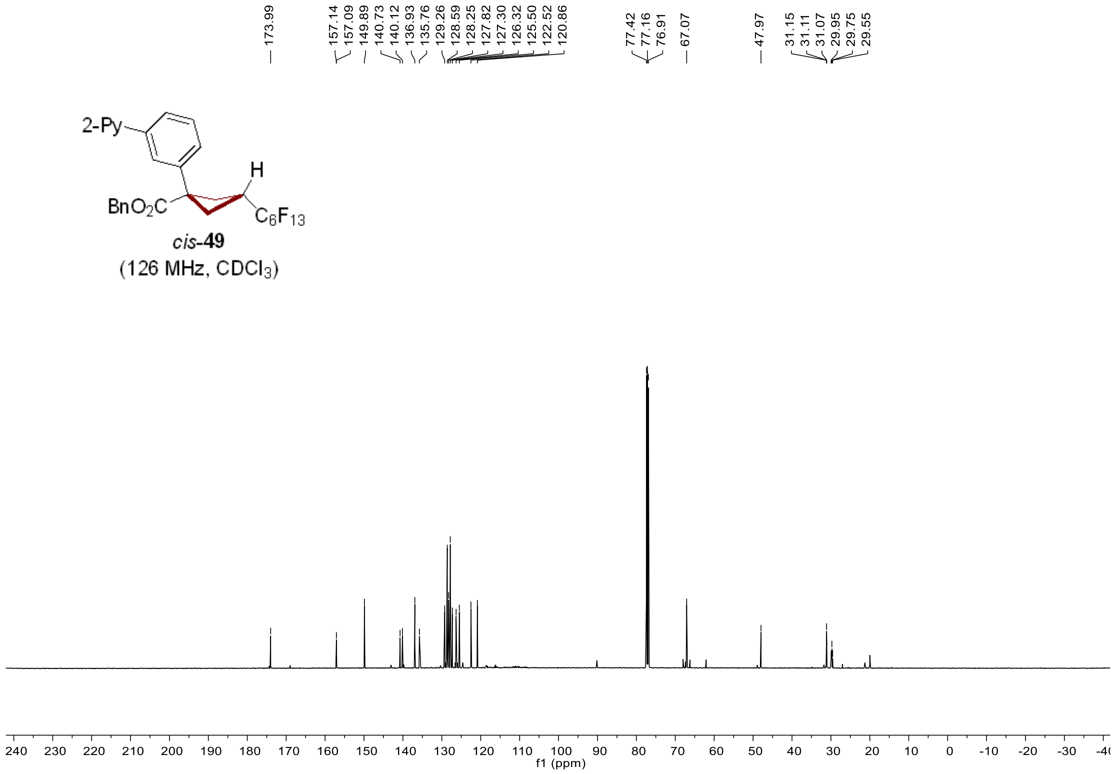
173.99
157.14
157.09
149.89
140.73
140.12
136.93
135.76
129.26
128.59
128.25
127.82
127.30
126.32
125.50
122.52
120.86
77.42
77.16
76.91
67.07
47.97
31.15
31.11
31.07
29.95
29.75
29.55
2-Py
H
$BnO_2C$
$C_6F_{13}$
*cis*-**49**
(126 MHz, $CDCl_3$)
f1 (ppm)

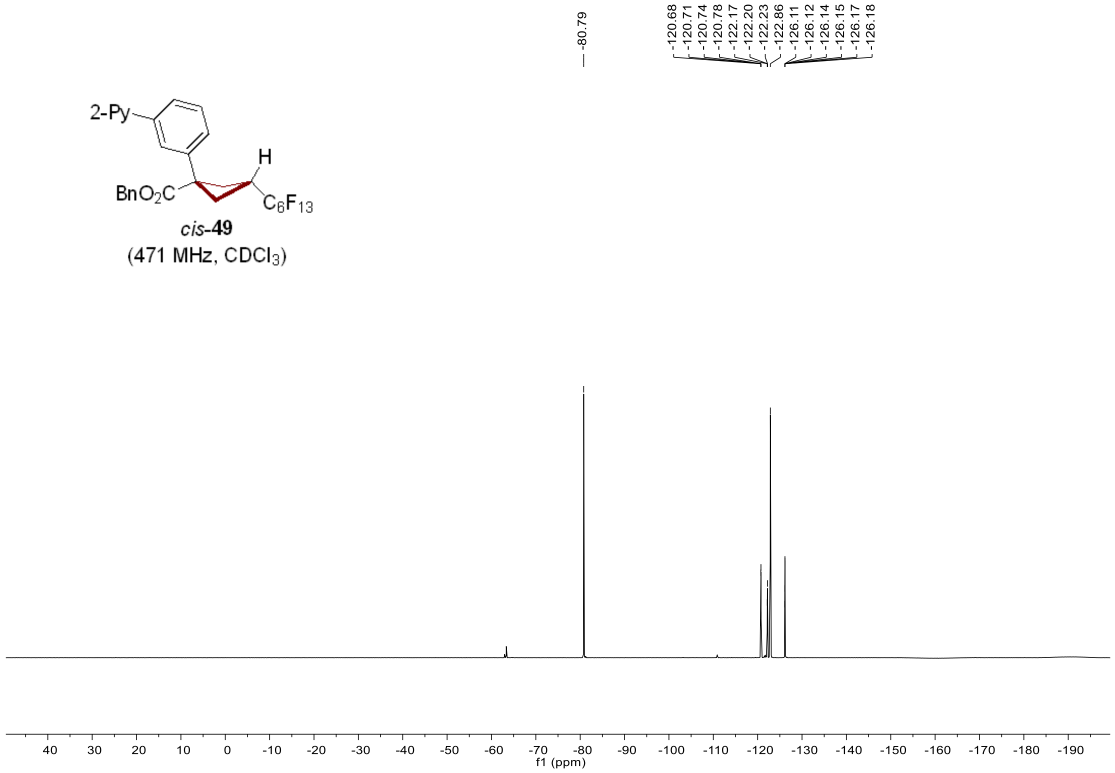
-80.79
-120.68
-120.71
-120.74
-120.78
-122.17
-122.20
-122.23
-122.86
-126.11
-126.12
-126.14
-126.15
-126.17
-126.18
2-Py
H
$BnO_2C$
$C_6F_{13}$
*cis*-**49**
(471 MHz, $CDCl_3$)
f1 (ppm)

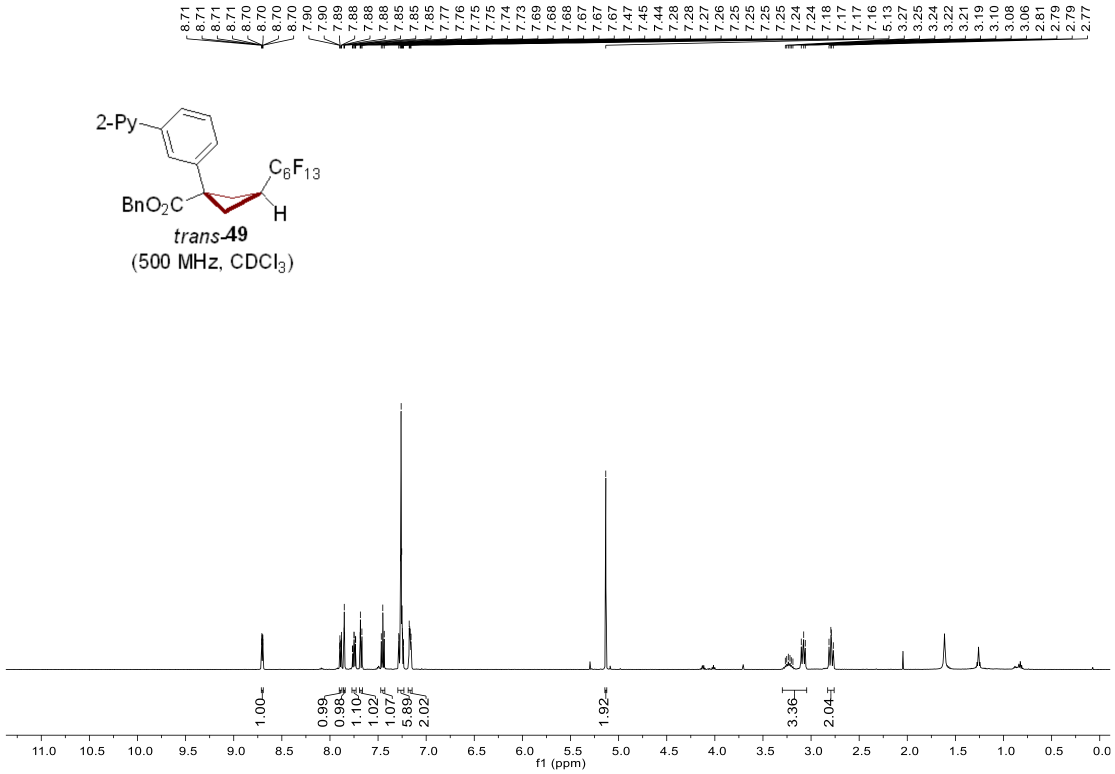
8.71 8.71 8.71 8.71 8.70 8.70 8.70 7.90 7.89 7.88 7.88 7.88 7.85 7.85 7.77 7.76 7.75 7.75 7.74 7.73 7.69 7.68 7.68 7.67 7.67 7.47 7.45 7.44 7.28 7.27 7.26 7.25 7.25 7.25 7.24 7.24 7.18 7.17 7.16 5.13 3.27 3.25 3.24 3.22 3.21 3.19 3.10 3.08 3.06 2.81 2.79 2.77
2-Py
$C_6F_{13}$
$BnO_2C$
H
trans-49
(500 MHz, $CDCl_3$)
1.00 0.99 0.98 1.10 1.02 1.07 5.89 2.02 1.92 3.36 2.04
f1 (ppm)

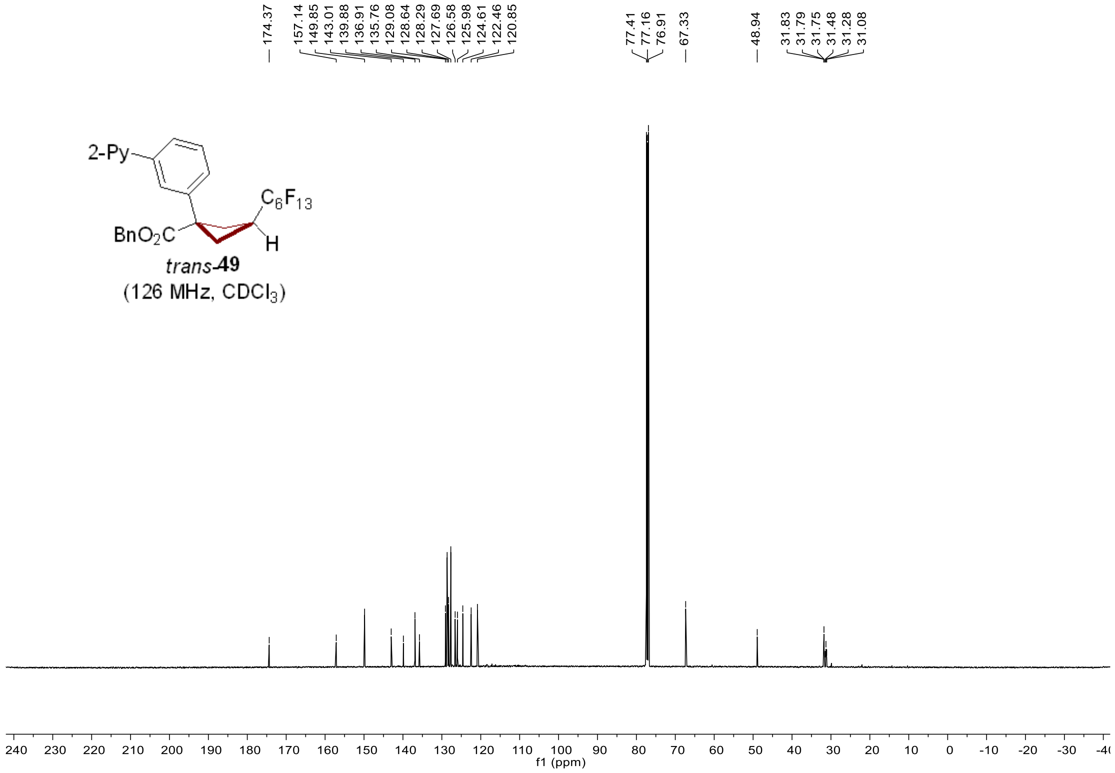
174.37 157.14 149.85 143.01 139.88 136.91 135.76 129.08 128.64 128.29 127.69 126.58 125.98 124.61 122.46 120.85 77.41 77.16 76.91 67.33 48.94 31.83 31.79 31.75 31.48 31.28 31.08
2-Py
$C_6F_{13}$
$BnO_2C$
H
trans-49
(126 MHz, $CDCl_3$)
f1 (ppm)

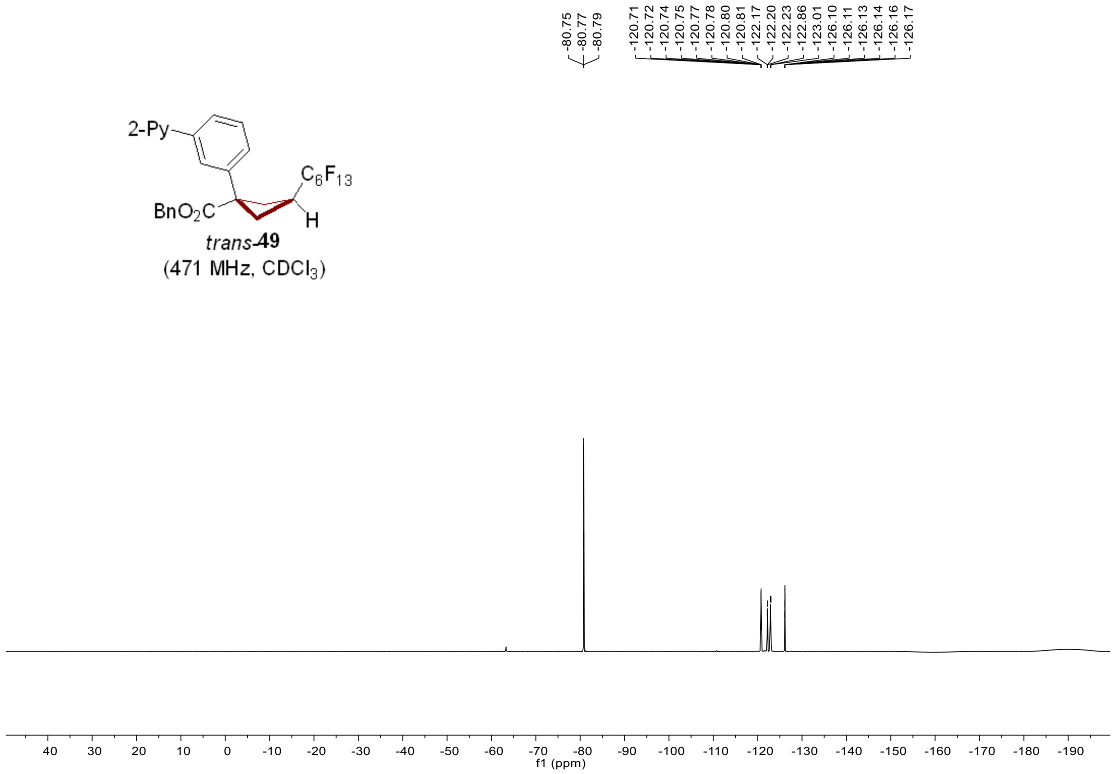
-80.75
-80.77
-80.79
-120.71
-120.72
-120.74
-120.75
-120.77
-120.78
-120.80
-120.81
-122.17
-122.20
-122.23
-122.86
-123.01
-126.10
-126.11
-126.13
-126.14
-126.16
-126.17
2-Py
$C_6F_{13}$
$BnO_2C$
H
*trans*-**49**
(471 MHz, $CDCl_3$)
f1 (ppm)

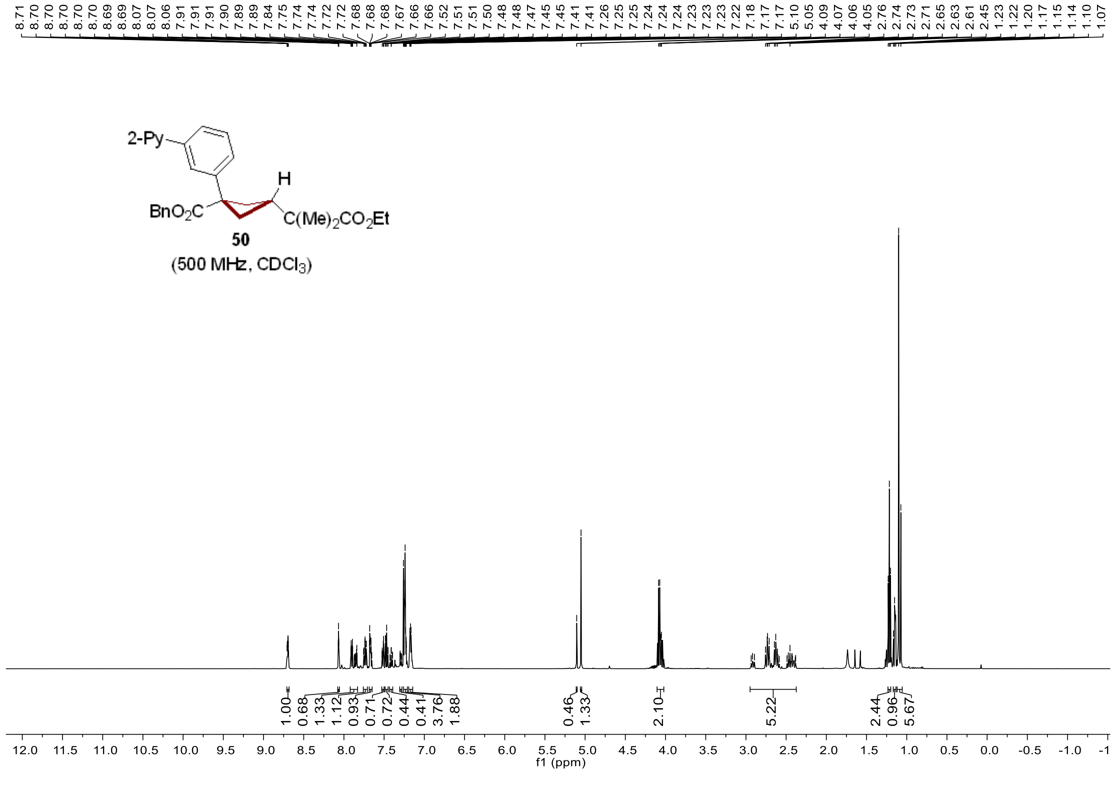
2-Py
H
$BnO_2C$
$C(Me)_2CO_2Et$
**50**
(500 MHz, $CDCl_3$)
1.00
0.68
1.33
1.12
0.93
0.71
0.72
0.44
0.41
3.76
1.88
0.46
1.33
2.10
5.22
2.44
0.96
5.67
f1 (ppm)

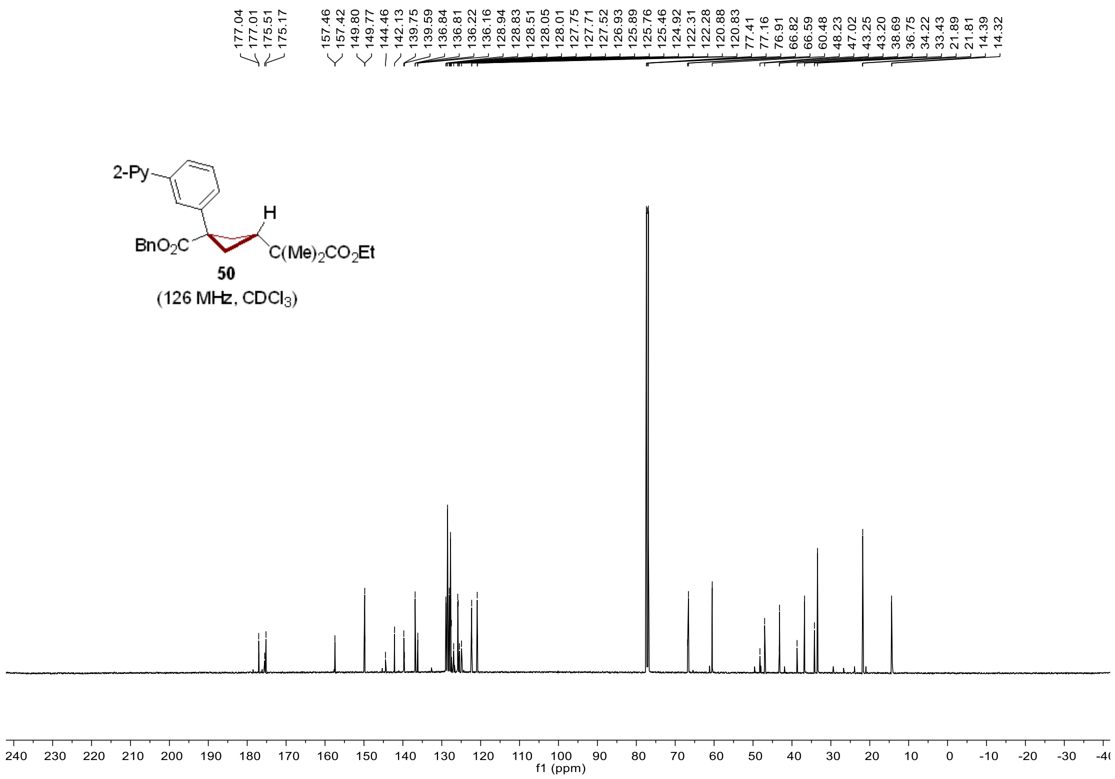


Note: product **51** was obtained from a prochiral radical species, only two isomers were observed in this case, i.e *cis*-**51** and *trans*-**51**.

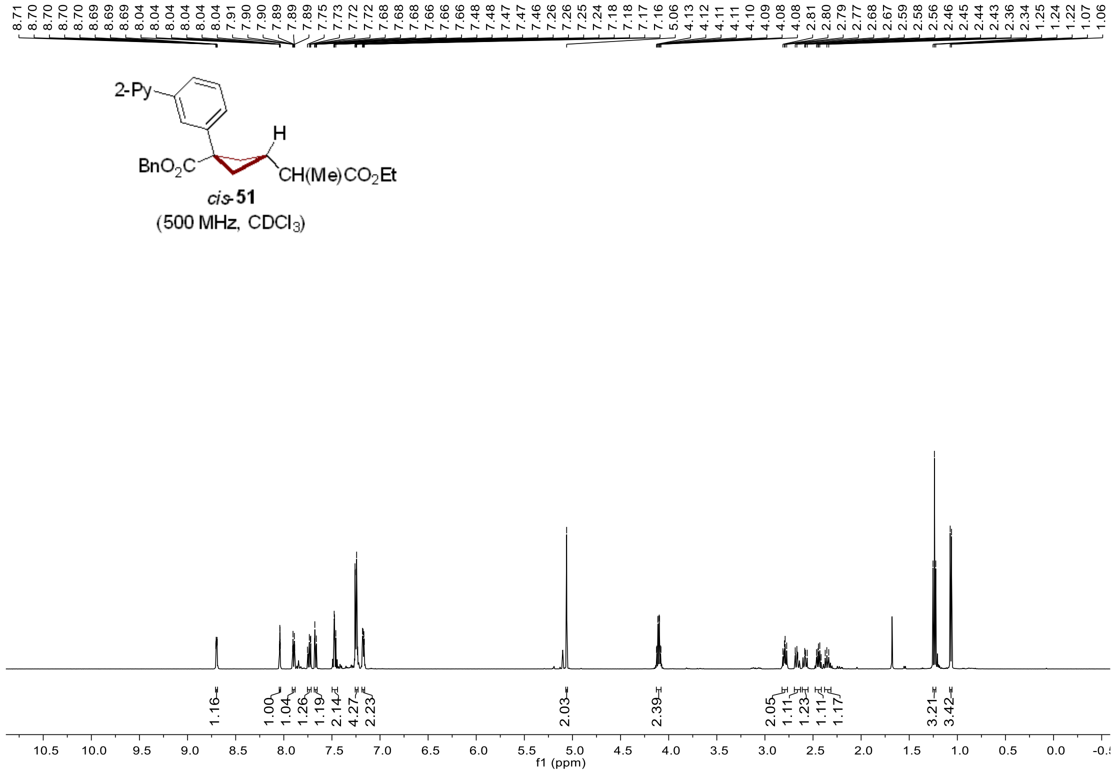

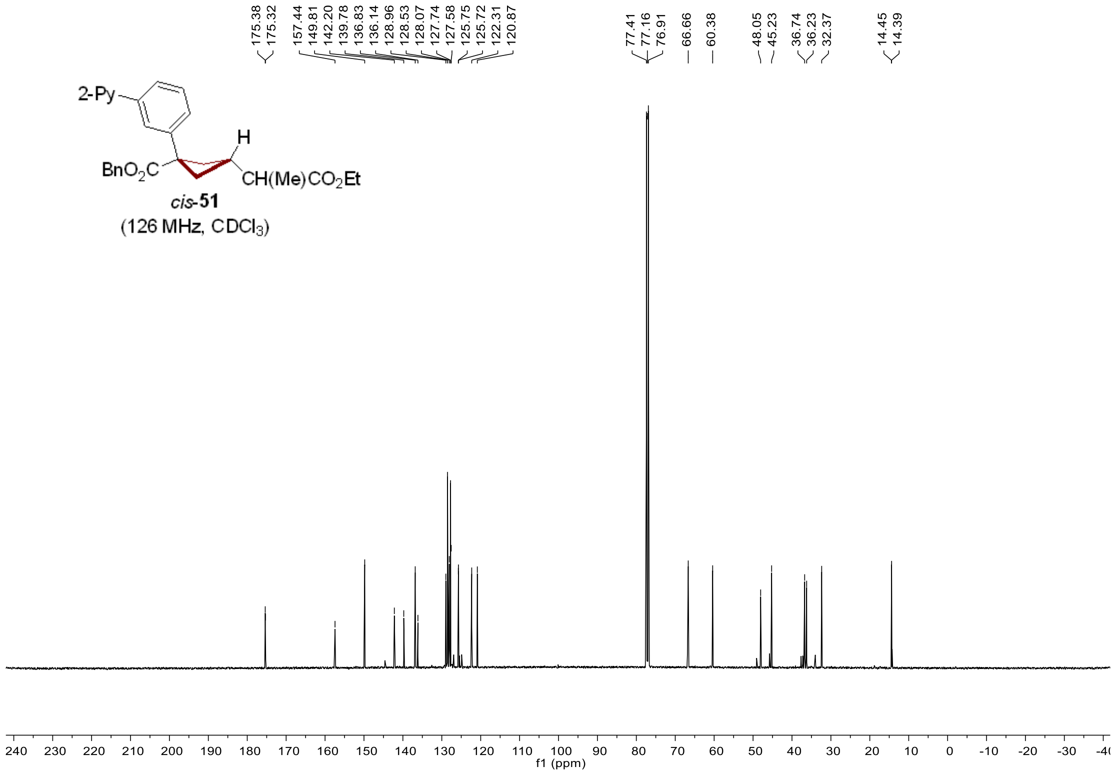
2-Py
H
BnO2C
CH(Me)CO2Et
cis-51
(126 MHz, CDCl3)
f1 (ppm)

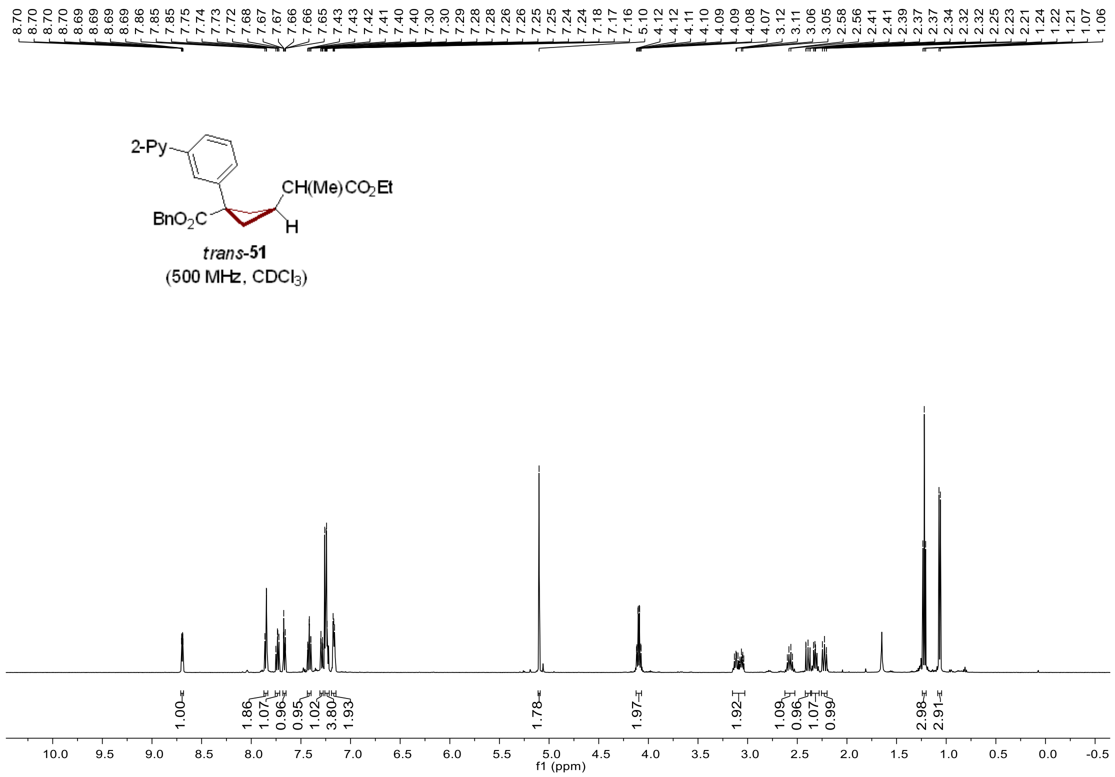
2-Py
CH(Me)CO2Et
BnO2C
H
trans-51
(500 MHz, CDCl3)
f1 (ppm)

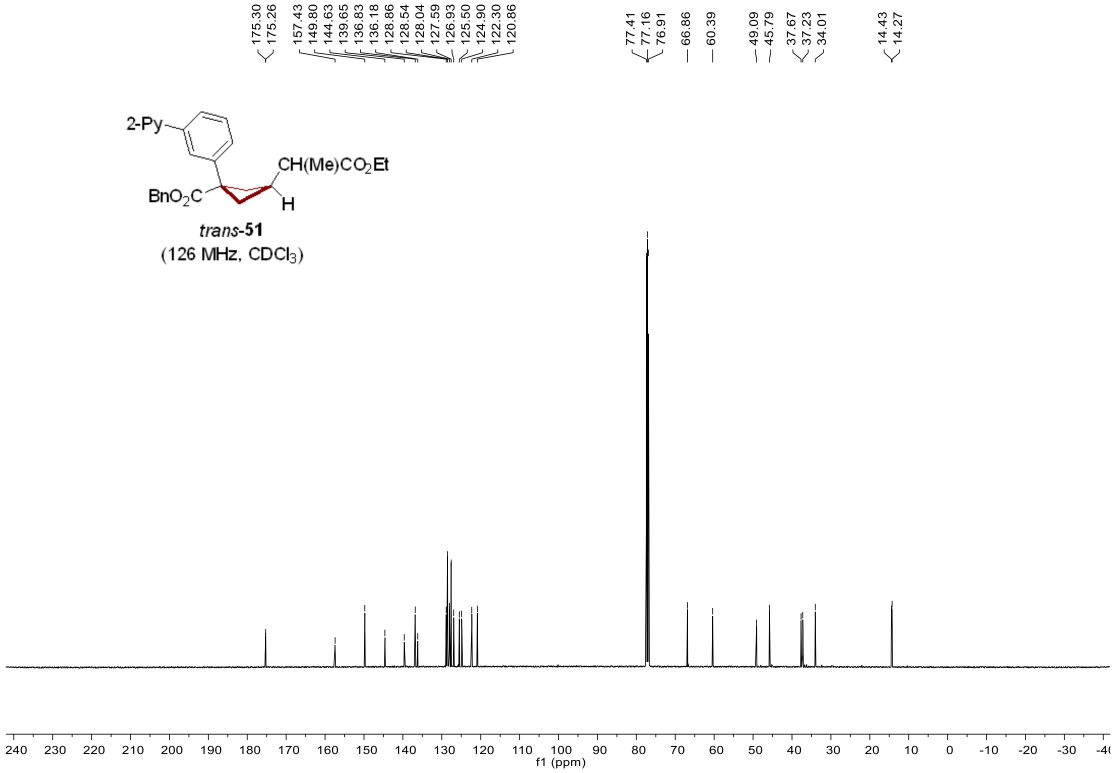
175.30
175.26
157.43
149.80
144.63
139.65
136.83
136.18
128.86
128.54
128.04
127.59
126.93
125.50
124.90
122.30
120.86
77.41
77.16
76.91
66.86
60.39
49.09
45.79
37.67
37.23
34.01
14.43
14.27
2-Py
CH(Me)CO2Et
BnO2C
H
trans-51
(126 MHz, CDCl3)
f1 (ppm)

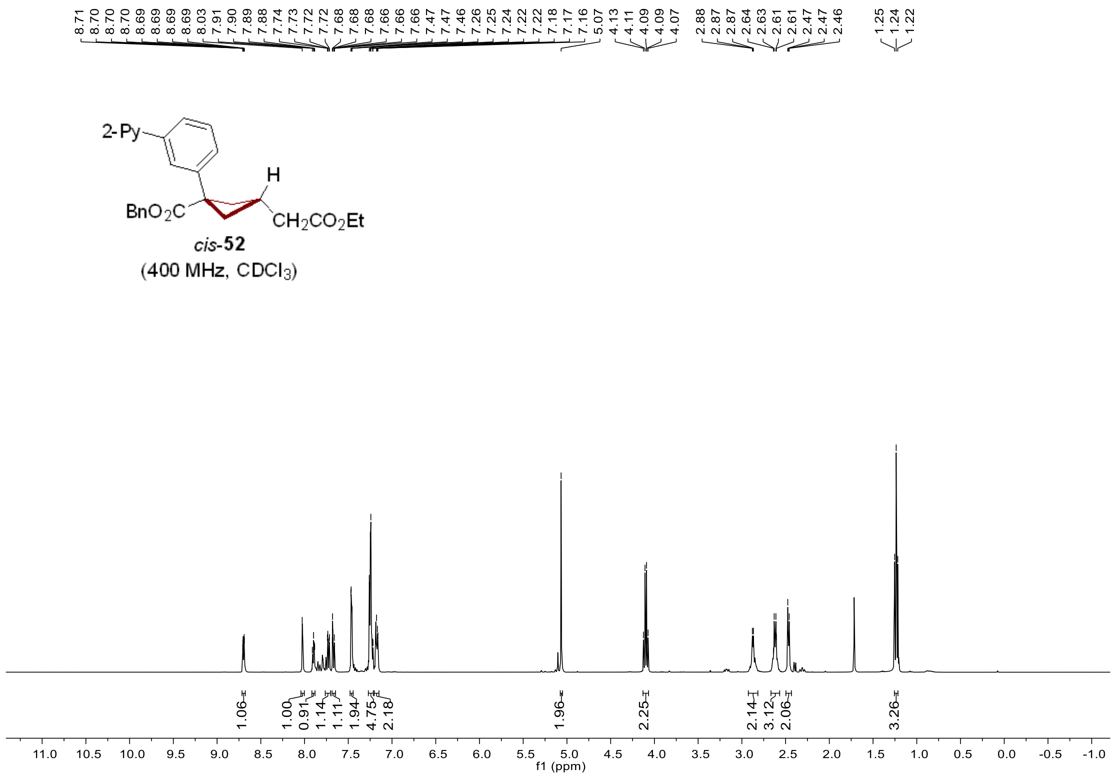
8.71
8.70
8.70
8.69
8.69
8.69
8.03
7.91
7.90
7.89
7.88
7.74
7.73
7.72
7.72
7.68
7.68
7.68
7.66
7.66
7.47
7.47
7.46
7.26
7.25
7.24
7.22
7.22
7.18
7.17
7.16
5.07
4.13
4.11
4.09
4.09
4.07
2.88
2.87
2.87
2.64
2.63
2.61
2.61
2.47
2.47
2.46
1.25
1.24
1.22
2-Py
H
BnO2C
CH2CO2Et
cis-52
(400 MHz, CDCl3)
1.06
1.00
0.91
1.14
1.11
1.94
4.75
2.18
1.96
2.25
2.14
3.12
2.06
3.26
f1 (ppm)

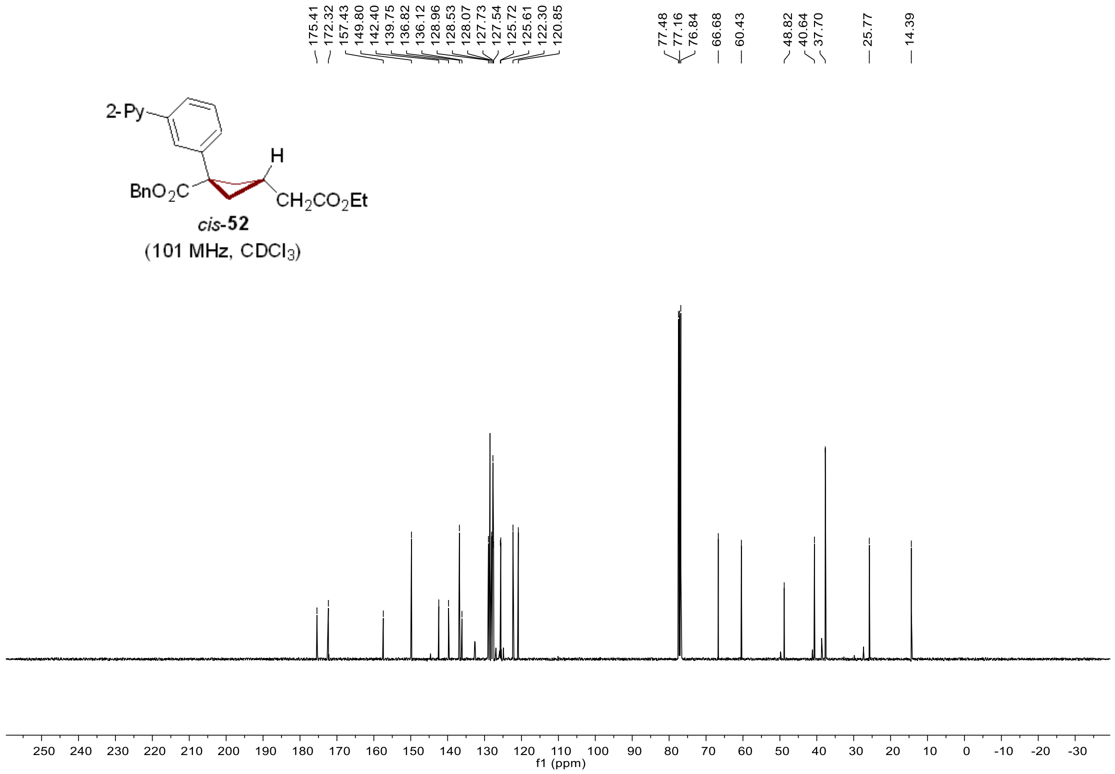
175.41
172.32
157.43
149.80
142.40
139.75
136.82
136.12
128.96
128.53
128.07
127.73
127.54
125.72
125.61
122.30
120.85
77.48
77.16
76.84
66.68
60.43
48.82
40.64
37.70
25.77
14.39
2-Py
H
$BnO_2C$
$CH_2CO_2Et$
*cis*-**52**
(101 MHz, $CDCl_3$)
250 240 230 220 210 200 190 180 170 160 150 140 130 120 110 100 90 80 70 60 50 40 30 20 10 0 -10 -20 -30
f1 (ppm)

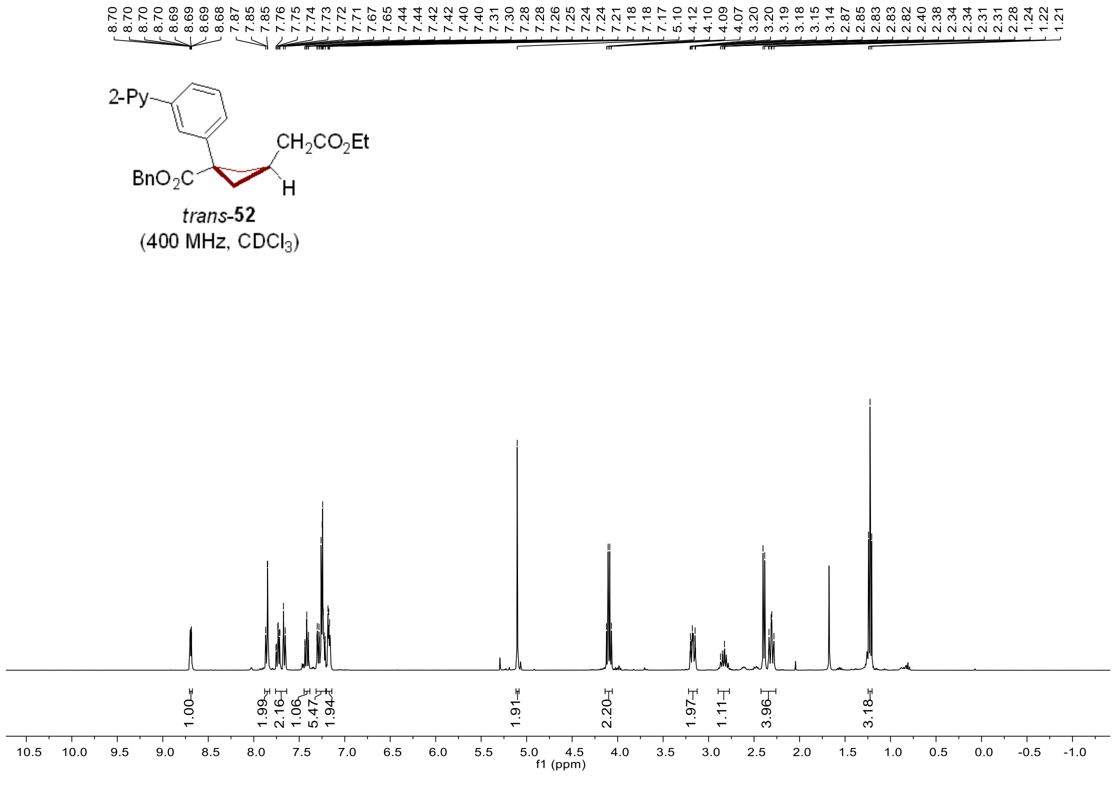
8.70
8.70
8.70
8.69
8.69
8.69
8.68
7.87
7.85
7.85
7.76
7.75
7.74
7.73
7.72
7.71
7.67
7.65
7.44
7.44
7.42
7.42
7.40
7.40
7.31
7.30
7.28
7.28
7.26
7.25
7.24
7.24
7.21
7.18
7.18
7.17
5.10
4.12
4.10
4.09
4.07
3.20
3.20
3.19
3.18
3.15
3.14
2.87
2.85
2.83
2.83
2.82
2.40
2.38
2.34
2.31
2.31
2.28
1.24
1.22
1.21
2-Py
$CH_2CO_2Et$
$BnO_2C$
H
*trans*-**52**
(400 MHz, $CDCl_3$)
1.00
1.99
2.16
1.06
5.47
1.94
1.91
2.20
1.97
1.11
3.96
3.18
10.5 10.0 9.5 9.0 8.5 8.0 7.5 7.0 6.5 6.0 5.5 5.0 4.5 4.0 3.5 3.0 2.5 2.0 1.5 1.0 0.5 0.0 -0.5 -1.0
f1 (ppm)

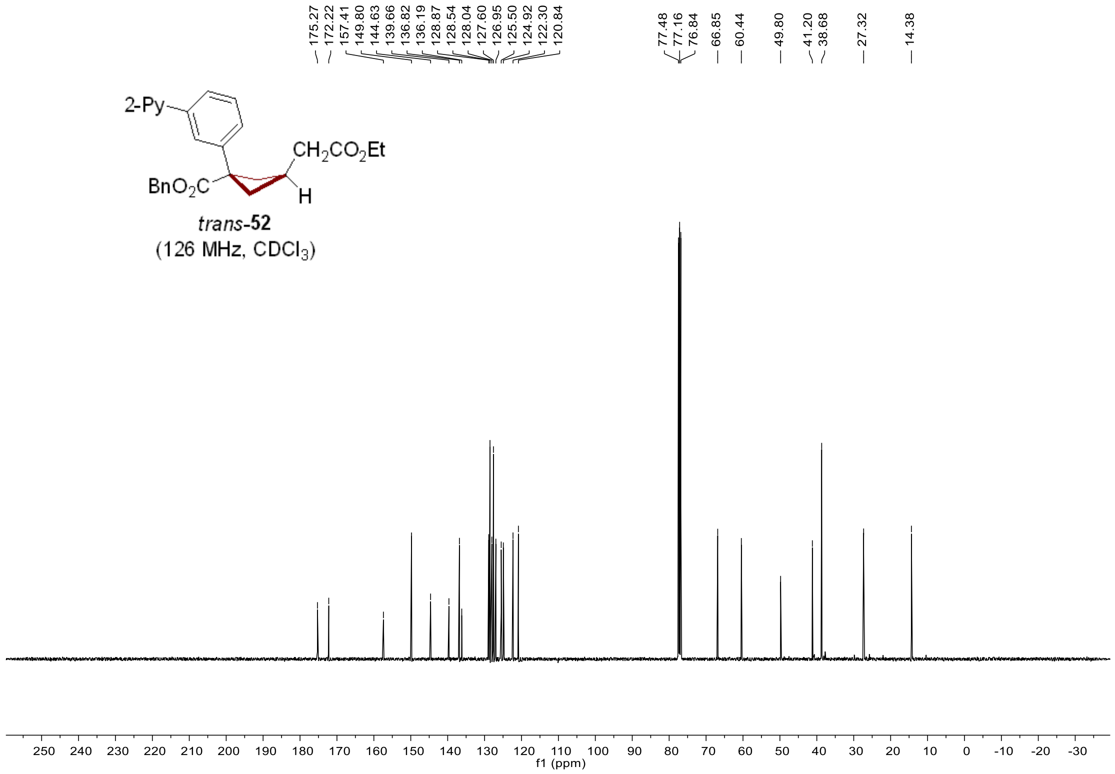

trans-**52**
(126 MHz, $CDCl_3$)

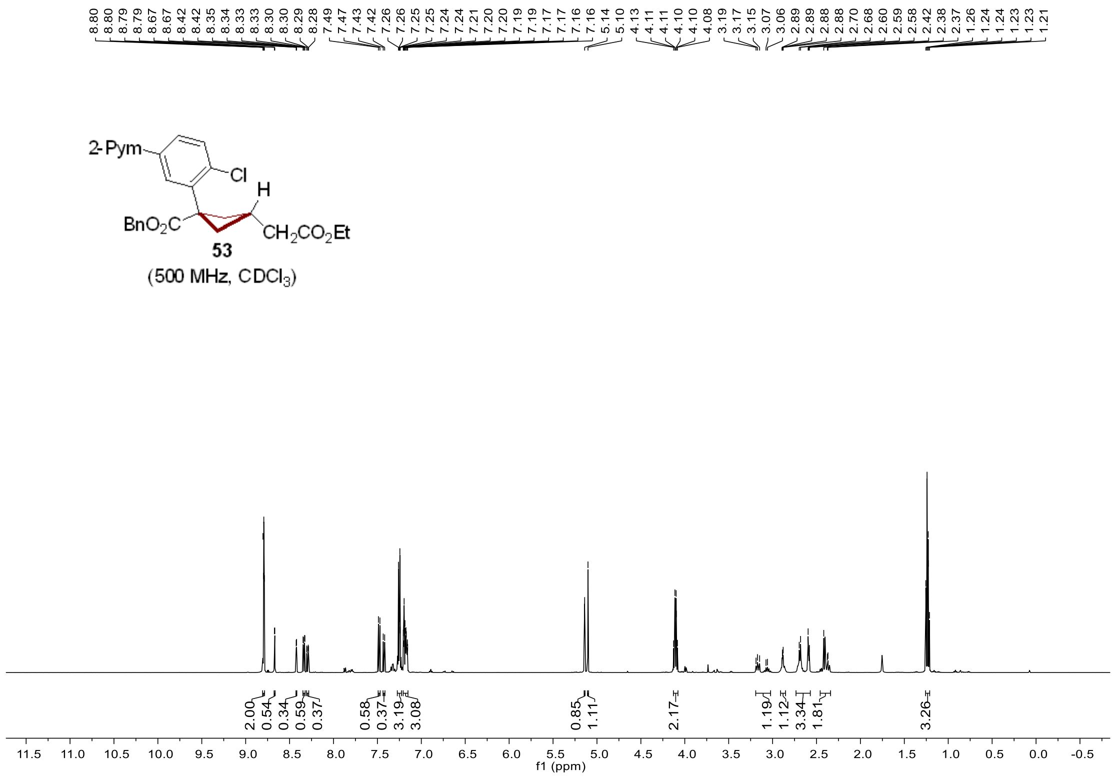

**53**
(500 MHz, $CDCl_3$)

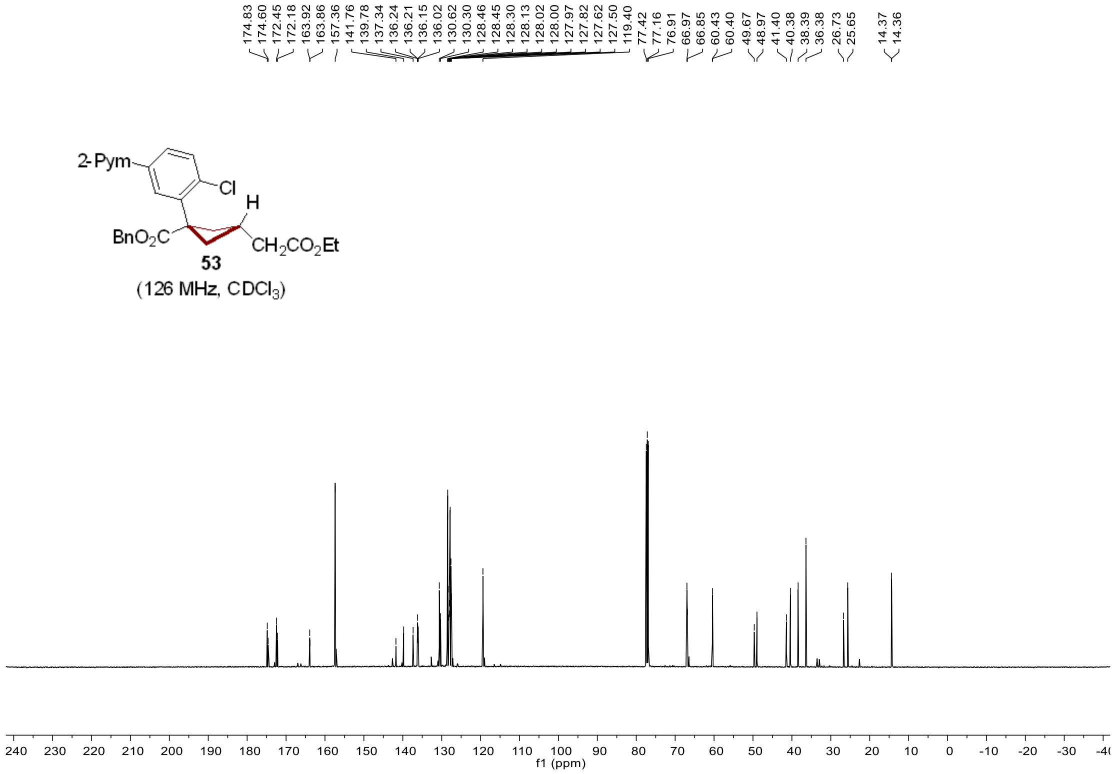
174.83
174.60
172.45
172.18
163.92
163.86
157.36
141.76
139.78
137.34
136.24
136.21
136.15
136.02
130.62
130.30
128.46
128.45
128.30
128.13
128.02
128.00
127.97
127.82
127.62
127.50
119.40
77.42
77.16
76.91
66.97
66.85
60.43
60.40
49.67
48.97
41.40
40.38
38.39
36.38
26.73
25.65
14.37
14.36
2-Pym
Cl
H
$BnO_2C$
$CH_2CO_2Et$
53
(126 MHz, $CDCl_3$)
f1 (ppm)

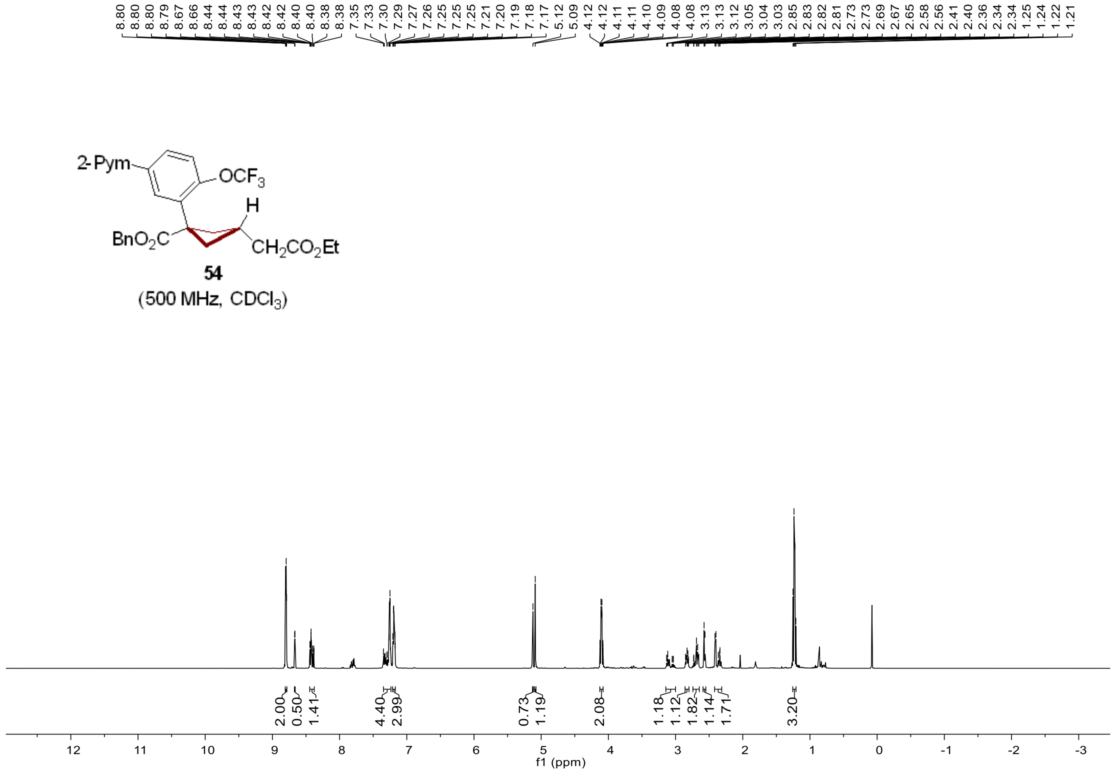
8.80
8.80
8.79
8.67
8.66
8.44
8.44
8.43
8.42
8.42
8.40
8.38
8.38
7.35
7.33
7.30
7.29
7.27
7.26
7.25
7.25
7.21
7.20
7.19
7.18
7.17
5.12
5.09
4.12
4.12
4.11
4.10
4.09
4.08
4.08
3.13
3.13
3.12
3.05
3.04
3.03
2.85
2.83
2.82
2.81
2.73
2.69
2.67
2.65
2.58
2.56
2.41
2.40
2.36
2.34
1.25
1.24
1.22
1.21
2-Pym
$OCF_3$
H
$BnO_2C$
$CH_2CO_2Et$
54
(500 MHz, $CDCl_3$)
2.00
0.50
1.41
4.40
2.99
0.73
1.19
2.08
1.18
1.12
1.82
1.14
1.71
3.20
f1 (ppm)

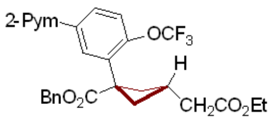


**54**
(126 MHz, $CDCl_3$)

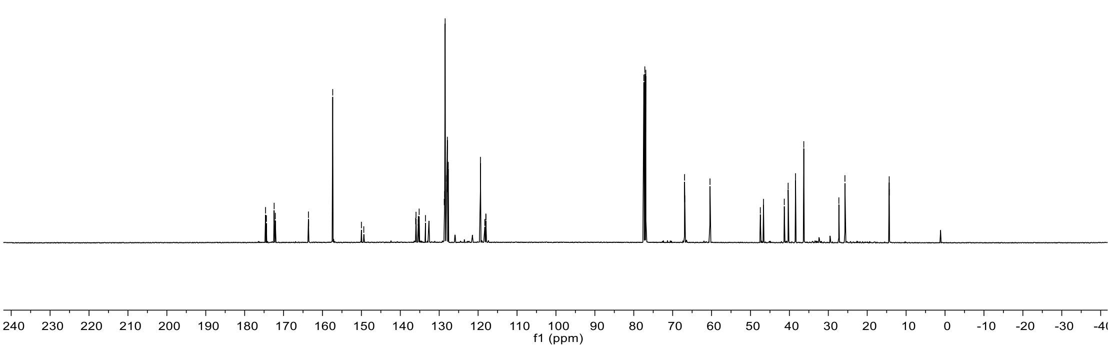


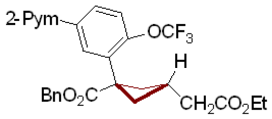


**54**
(471 MHz, $CDCl_3$)

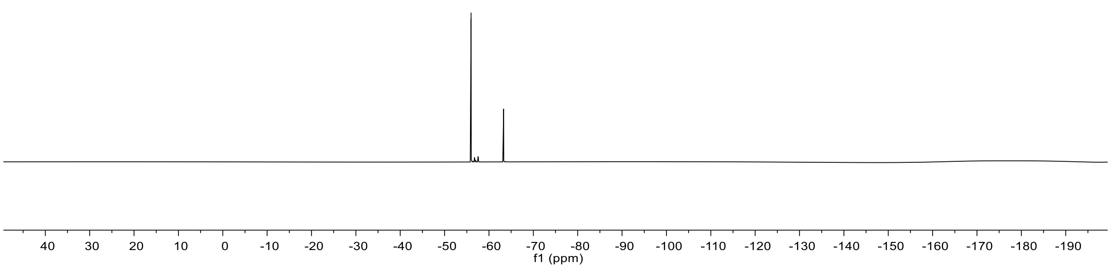

Note: product **55** was obtained from a prochiral radical species, only two isomers were observed in this case, i.e *cis*-**55** and *trans*-**55**.

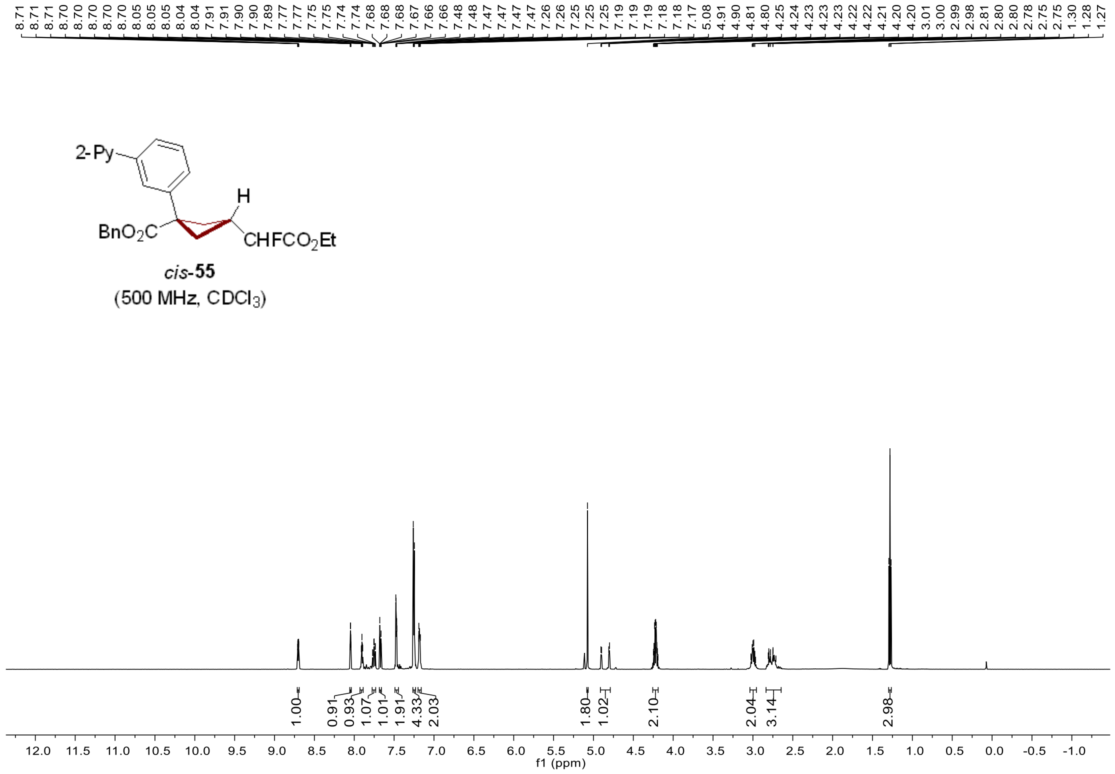


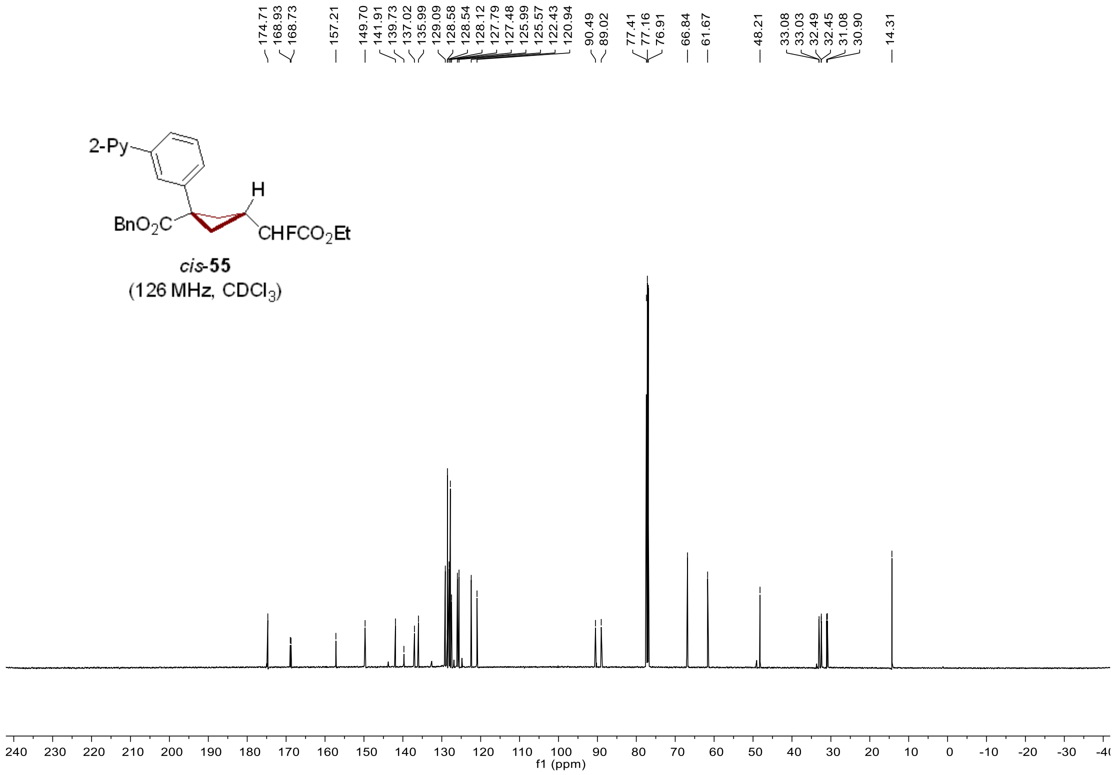

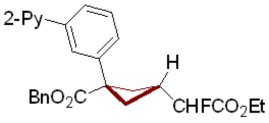

*cis*-**55**
(282 MHz, $CDCl_3$)

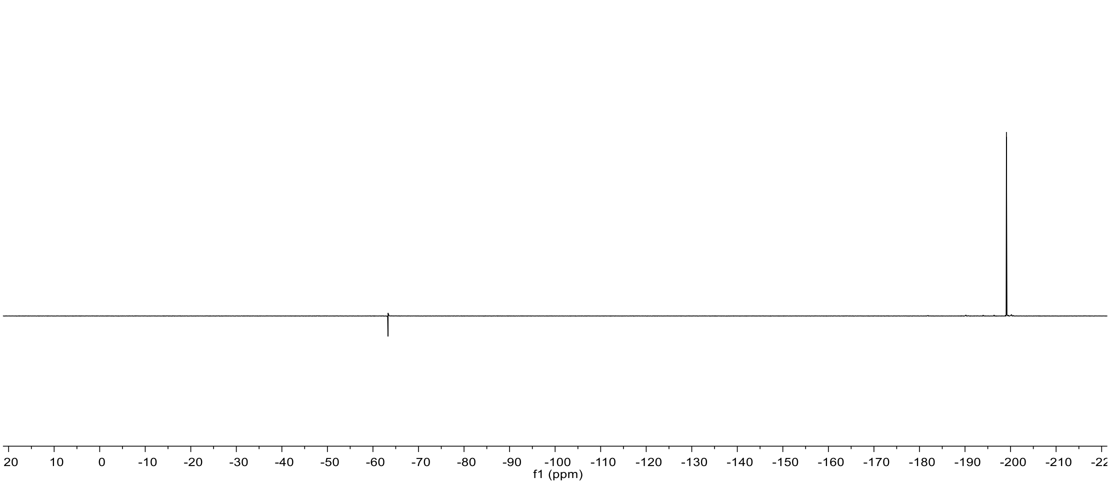


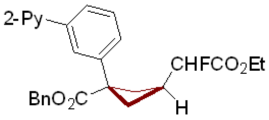

*trans*-**55**
(500 MHz, $CDCl_3$)

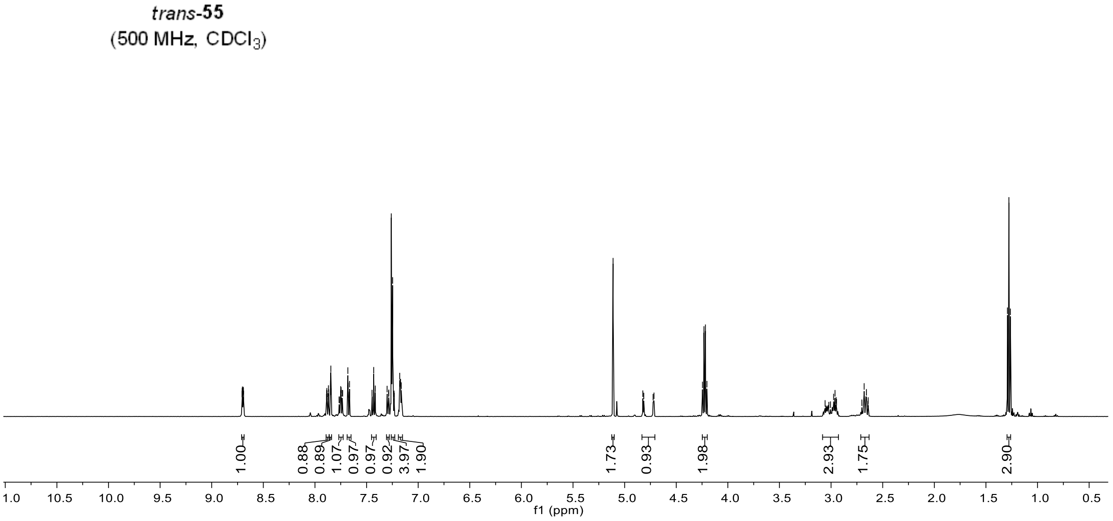

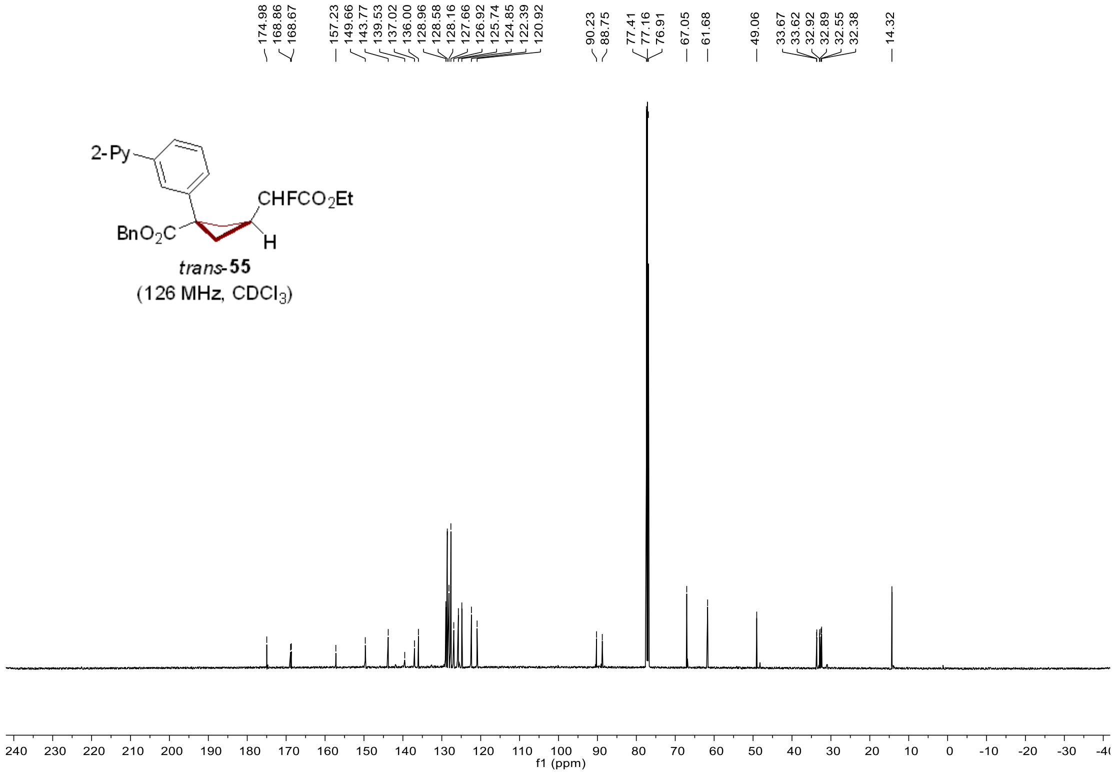
174.98
168.86
168.67
157.23
149.66
143.77
139.53
137.02
136.00
128.96
128.58
128.16
127.66
126.92
125.74
124.85
122.39
120.92
90.23
88.75
77.41
77.16
76.91
67.05
61.68
49.06
33.67
33.62
32.92
32.89
32.55
32.38
14.32
2-Py
CHFCO2Et
BnO2C
H
trans-55
(126 MHz, CDCl3)
f1 (ppm)

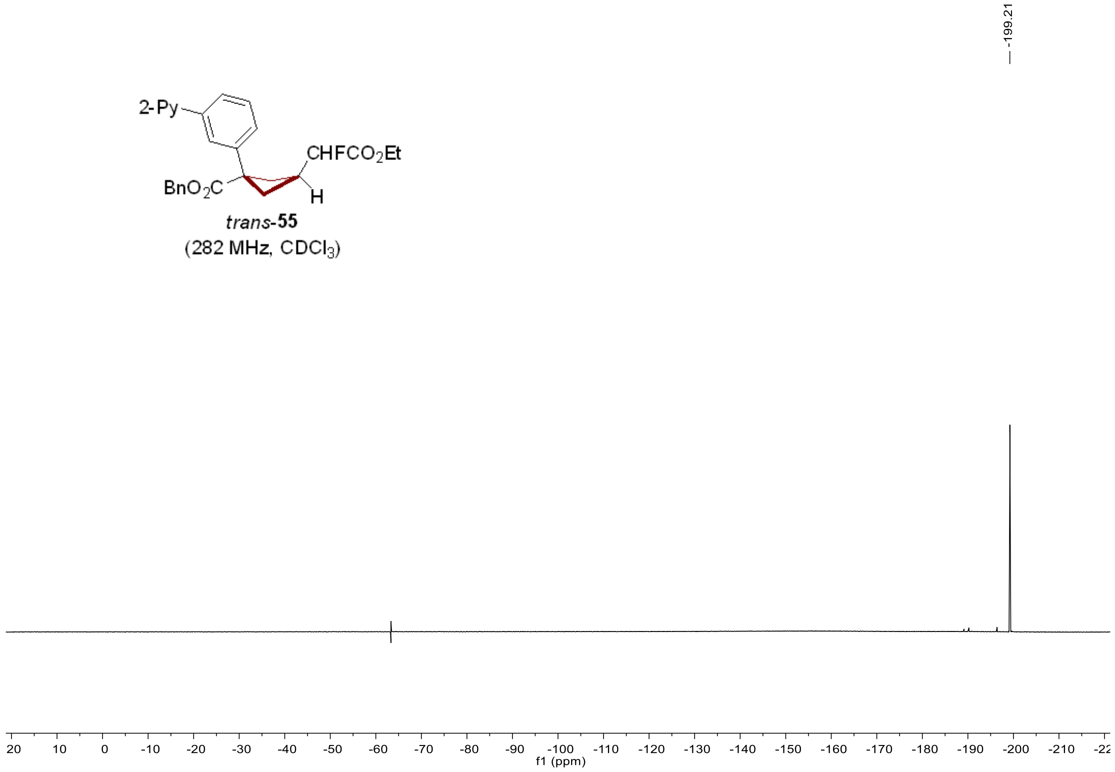
-199.21
2-Py
CHFCO2Et
BnO2C
H
trans-55
(282 MHz, CDCl3)
f1 (ppm)

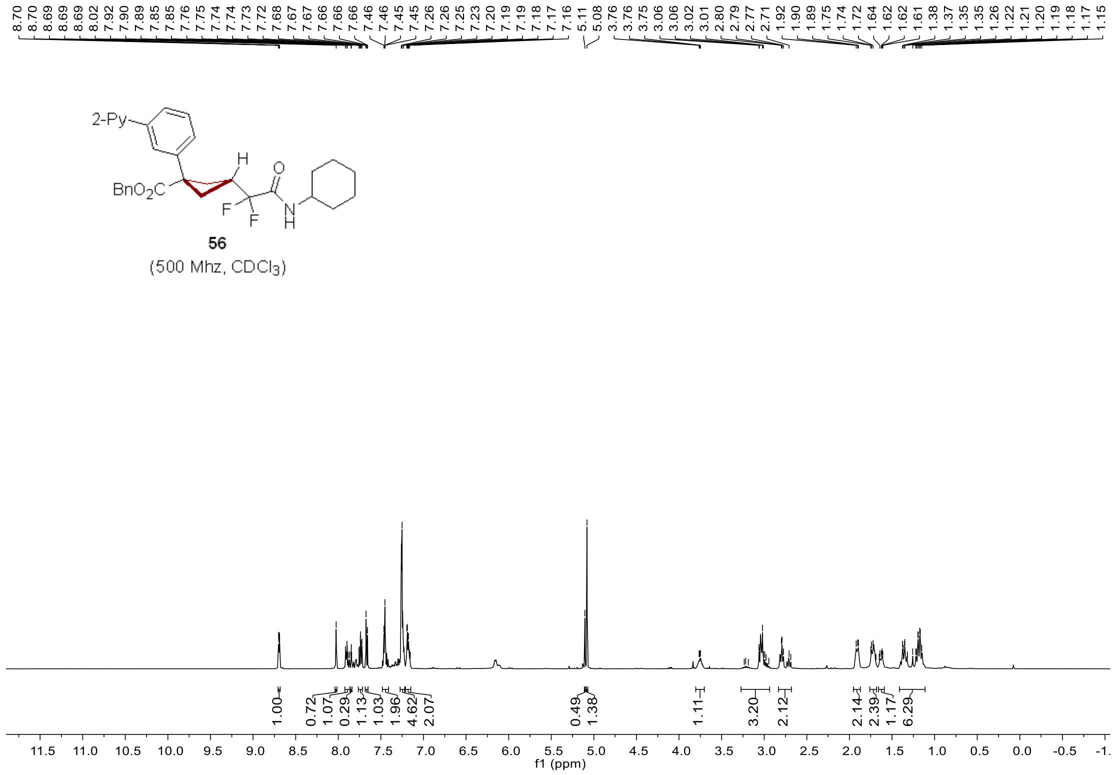


**56**

(500 Mhz, $CDCl_3$)

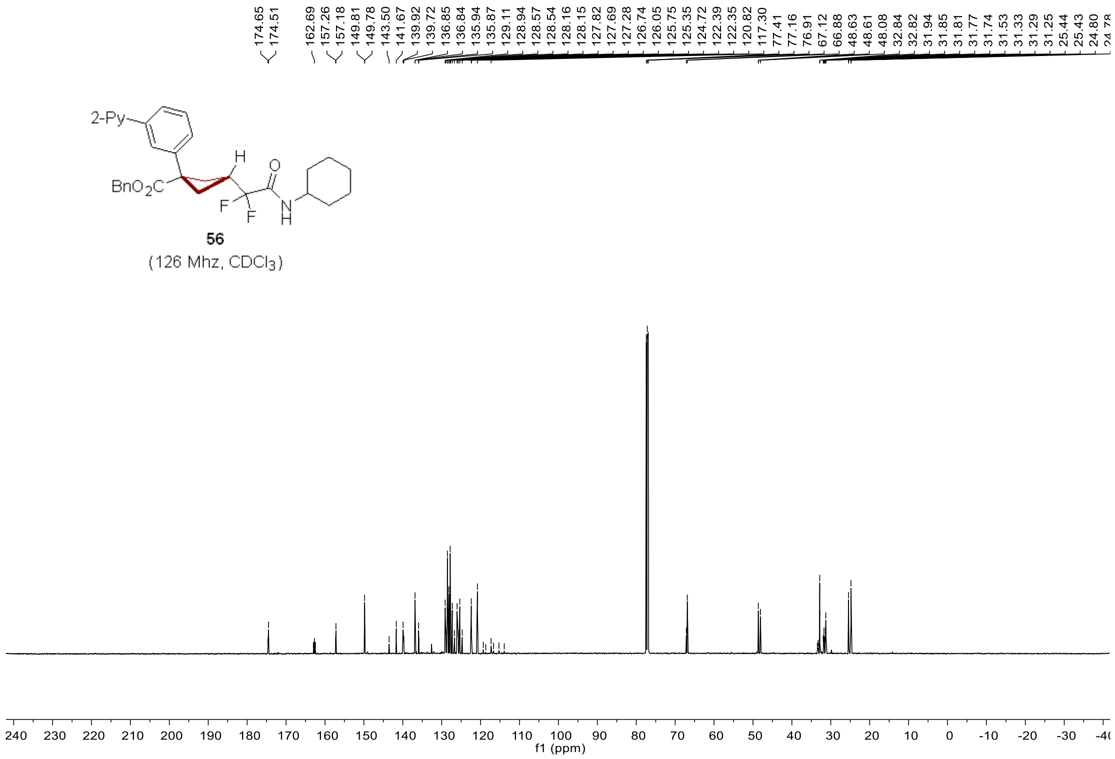


**56**

(126 Mhz, $CDCl_3$)

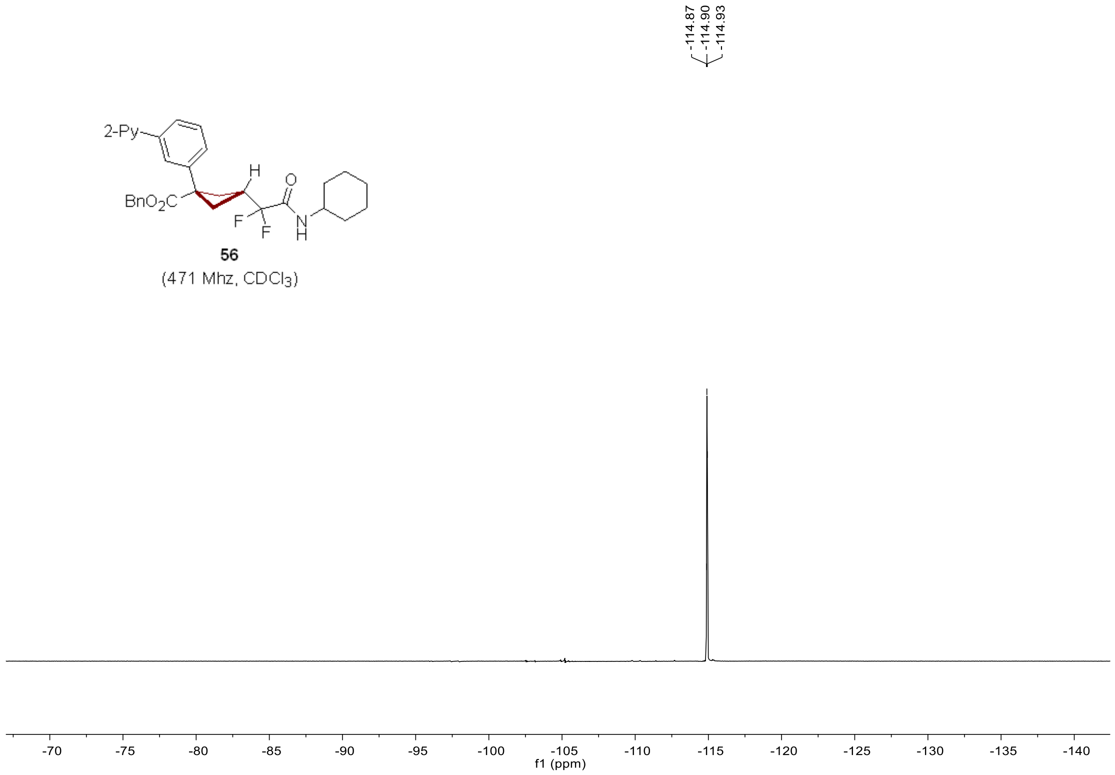


**56**
(471 Mhz, $CDCl_3$)

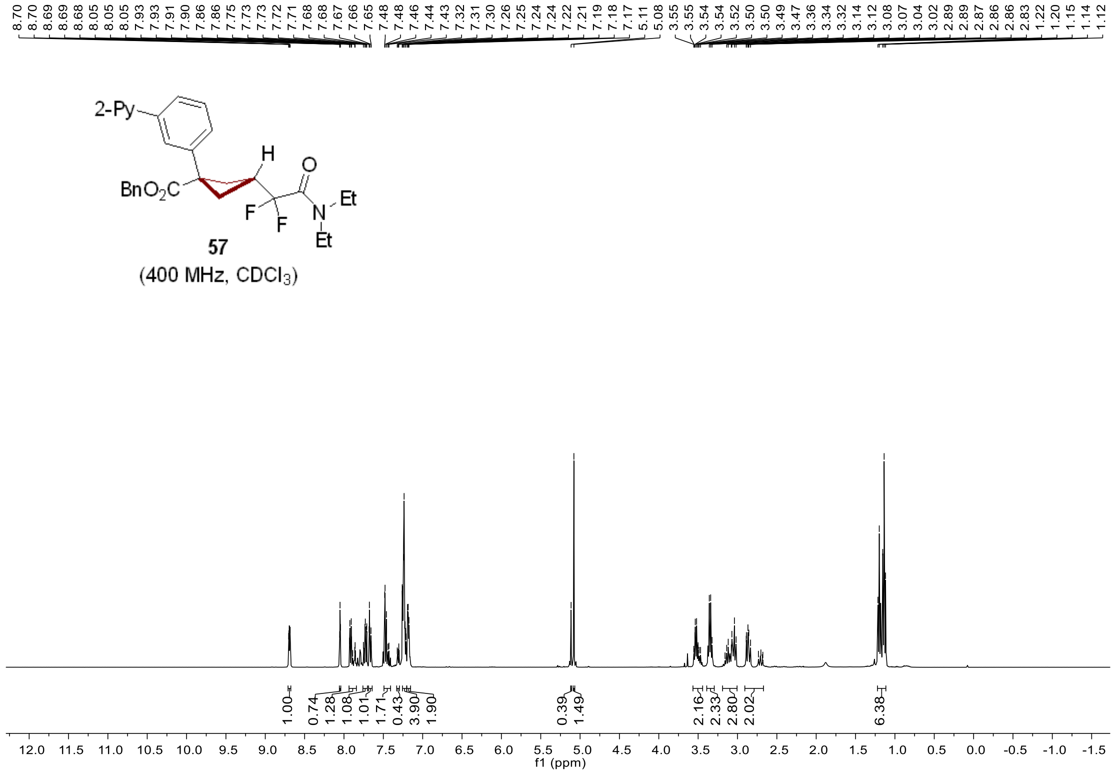


**57**
(400 MHz, $CDCl_3$)

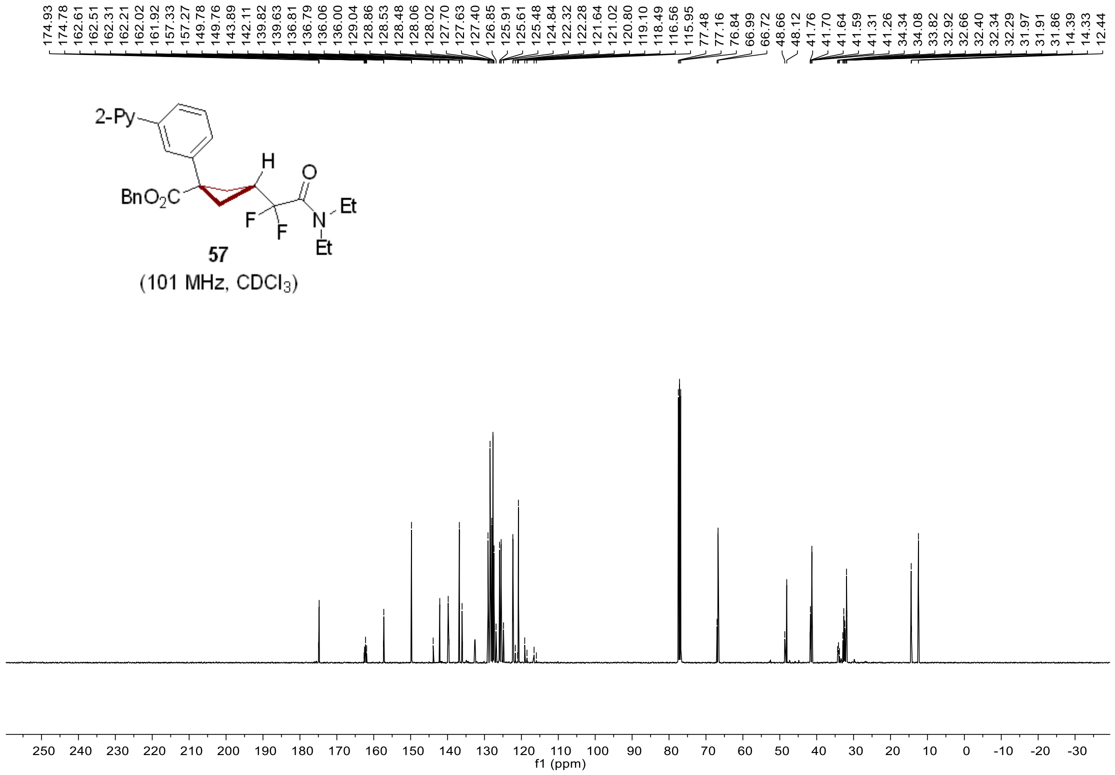
174.93
174.78
162.61
162.51
162.31
162.21
162.02
161.92
157.33
157.27
149.78
149.76
143.89
142.11
139.82
139.63
136.81
136.79
136.06
136.00
129.04
128.86
128.53
128.48
128.06
128.02
127.70
127.63
127.40
126.85
125.91
125.61
125.48
124.84
122.32
122.28
121.64
121.02
120.80
119.10
118.49
116.56
115.95
77.48
77.16
76.84
66.99
66.72
48.66
48.12
41.76
41.70
41.64
41.59
41.31
41.26
34.34
34.08
33.82
32.92
32.66
32.40
32.34
32.29
31.97
31.91
31.86
14.39
14.33
12.44
2-Py
H
O
$BnO_2C$
F
F
N
Et
Et
57
(101 MHz, $CDCl_3$)
250 240 230 220 210 200 190 180 170 160 150 140 130 120 110 100 90 80 70 60 50 40 30 20 10 0 -10 -20 -30
f1 (ppm)

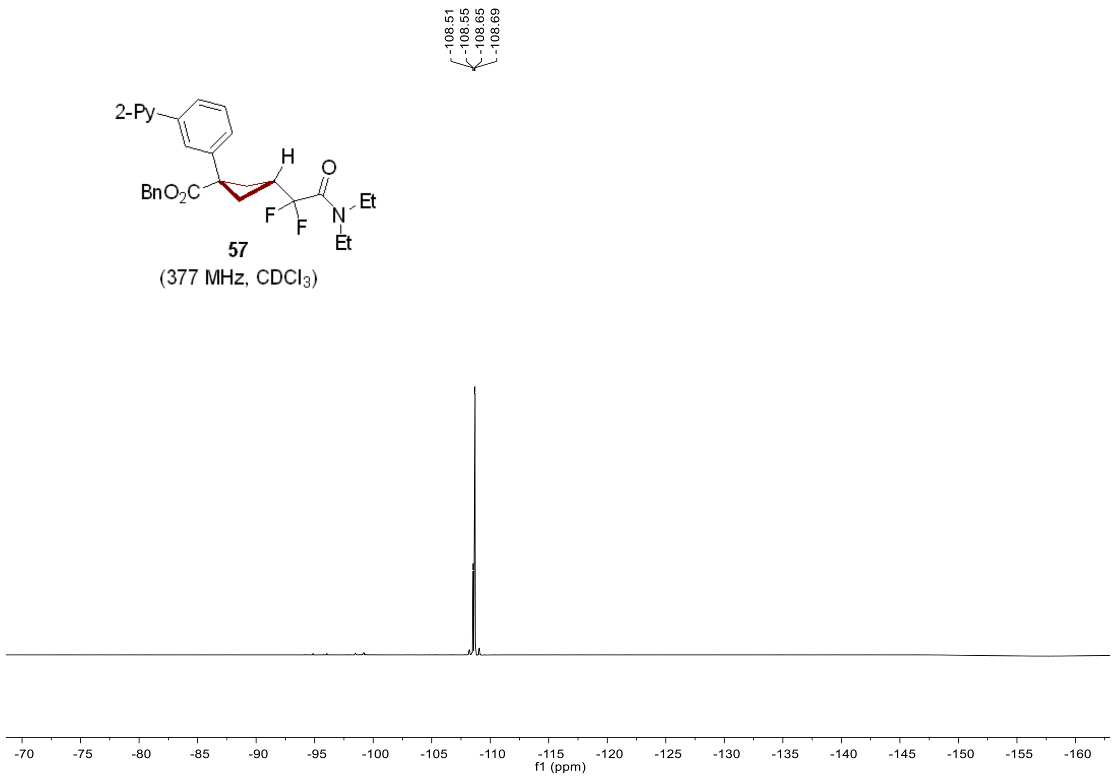
-108.51
-108.55
-108.65
-108.69
2-Py
H
O
$BnO_2C$
F
F
N
Et
Et
57
(377 MHz, $CDCl_3$)
-70 -75 -80 -85 -90 -95 -100 -105 -110 -115 -120 -125 -130 -135 -140 -145 -150 -155 -160
f1 (ppm)

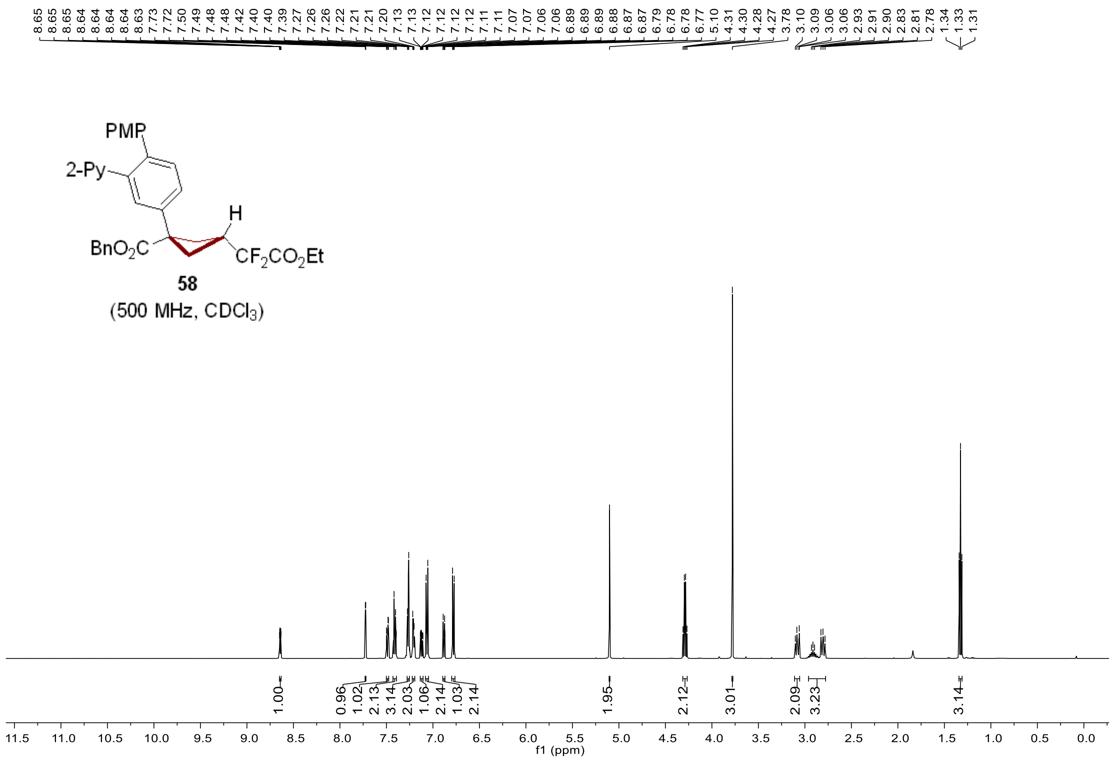
PMP
2-Py
H
BnO2C
CF2CO2Et
58
(500 MHz, CDCl3)
f1 (ppm)

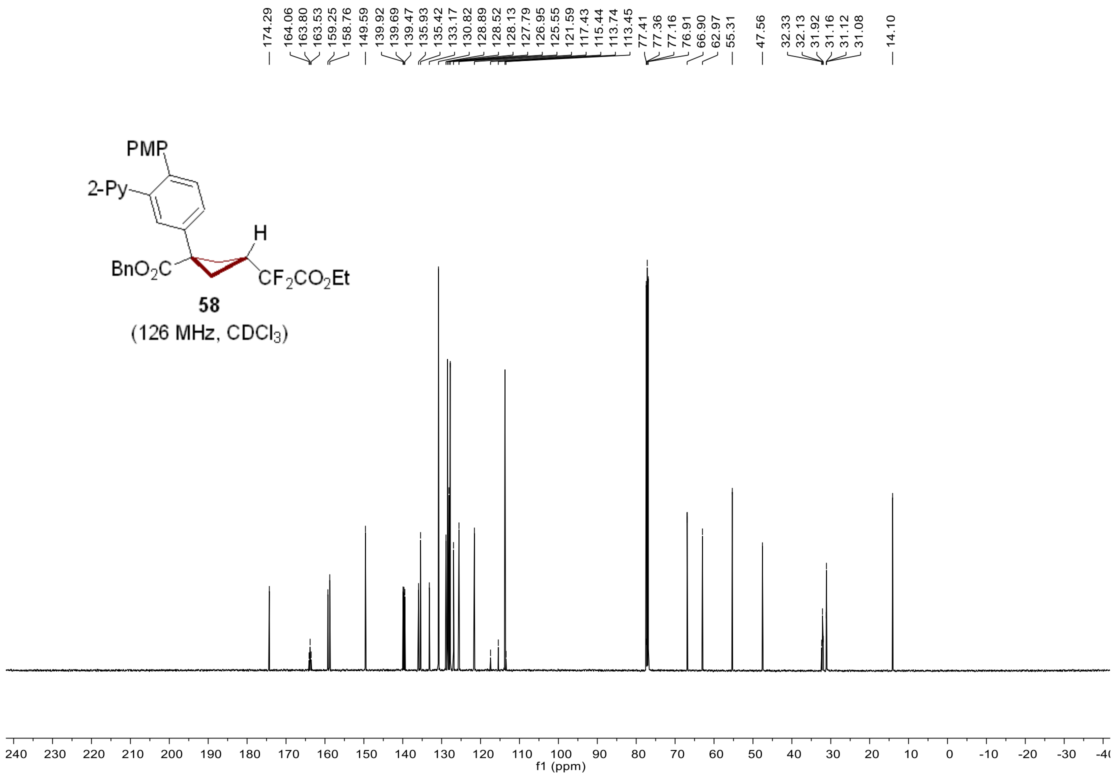
PMP
2-Py
H
BnO2C
CF2CO2Et
58
(126 MHz, CDCl3)
f1 (ppm)

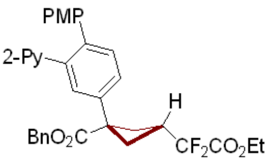


**58**

(471 MHz, $CDCl_3$)

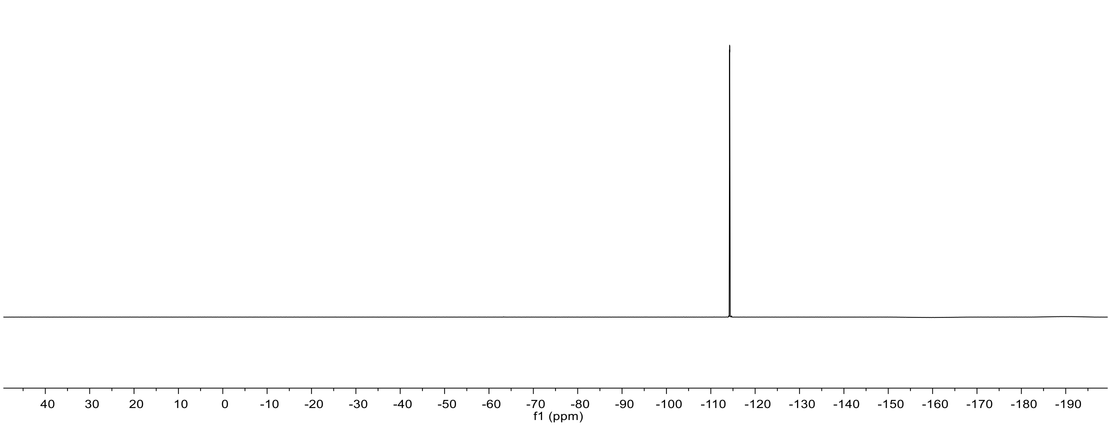


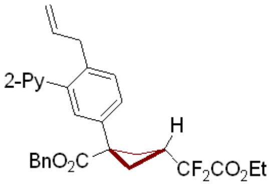


**59**

(500 MHz, $CDCl_3$)

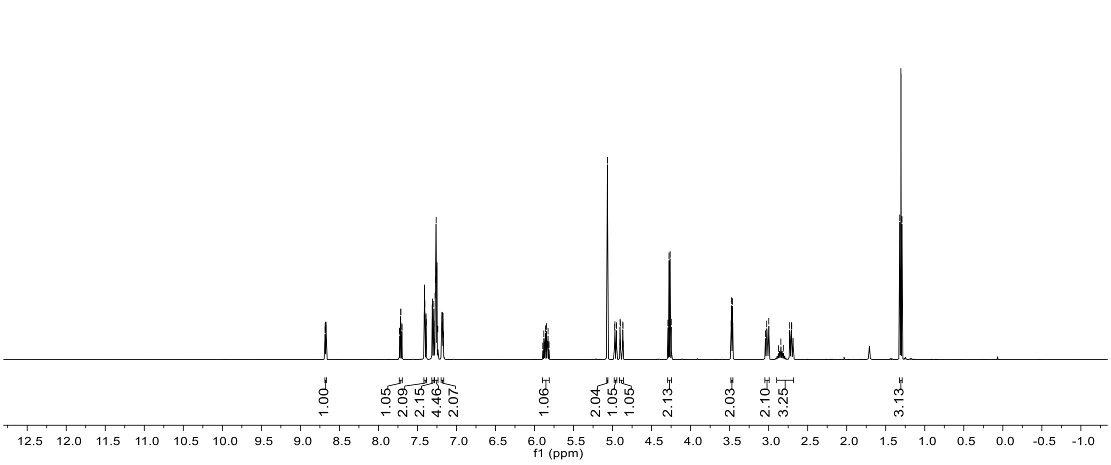

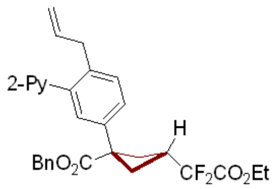


**59**

(126 MHz, $CDCl_3$)

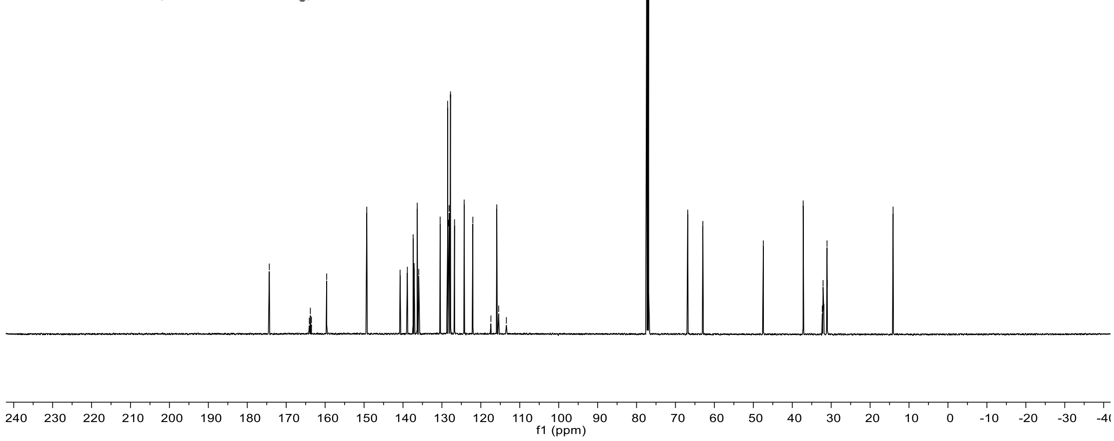


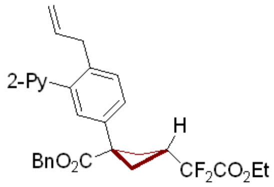


**59**

(471 MHz, $CDCl_3$)

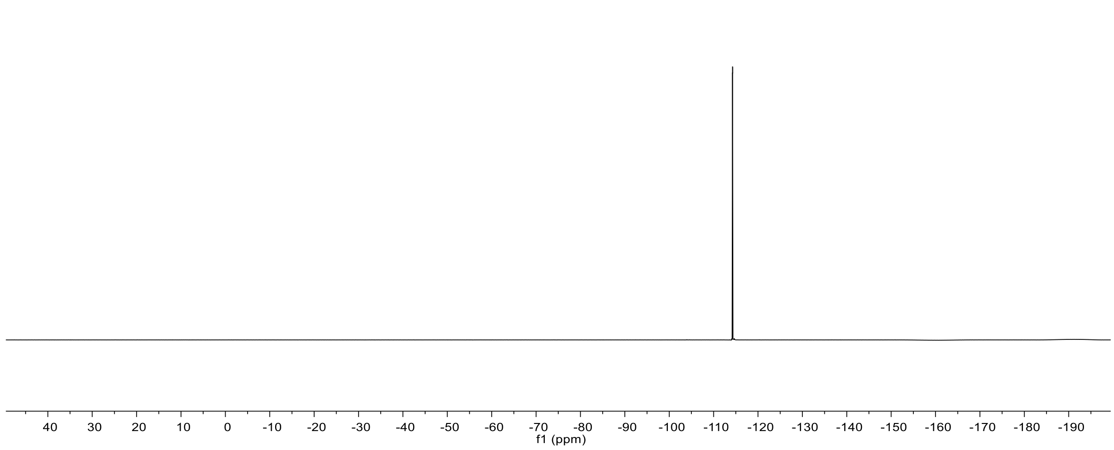

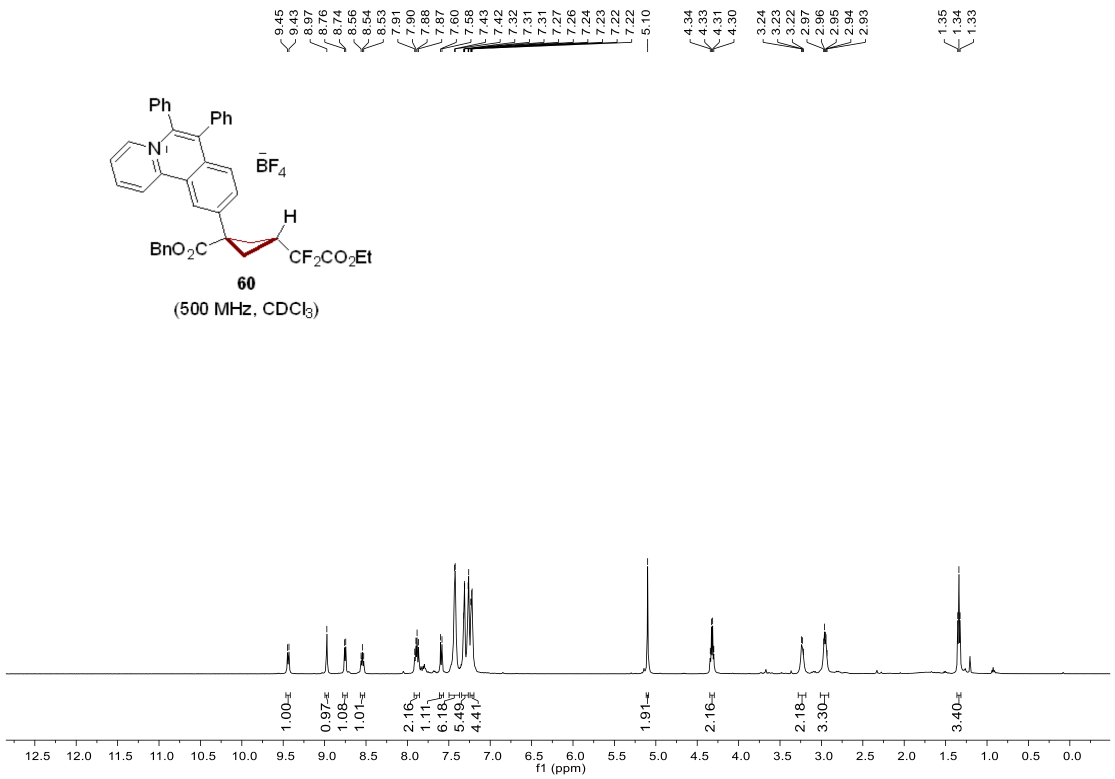
9.45
9.43
8.97
8.76
8.74
8.56
8.54
8.53
7.91
7.90
7.88
7.87
7.60
7.58
7.43
7.42
7.32
7.31
7.31
7.27
7.26
7.24
7.23
7.22
7.22
5.10
4.34
4.33
4.31
4.30
3.24
3.23
3.22
2.97
2.96
2.95
2.94
2.93
1.35
1.34
1.33
Ph
Ph
N
$\bar{B}F_4$
H
$BnO_2C$
$CF_2CO_2Et$
60
(500 MHz, $CDCl_3$)
1.00
0.97
1.08
1.01
2.16
1.11
6.18
5.49
4.41
1.91
2.16
2.18
3.30
3.40
12.5 12.0 11.5 11.0 10.5 10.0 9.5 9.0 8.5 8.0 7.5 7.0 6.5 6.0 5.5 5.0 4.5 4.0 3.5 3.0 2.5 2.0 1.5 1.0 0.5 0.0
f1 (ppm)

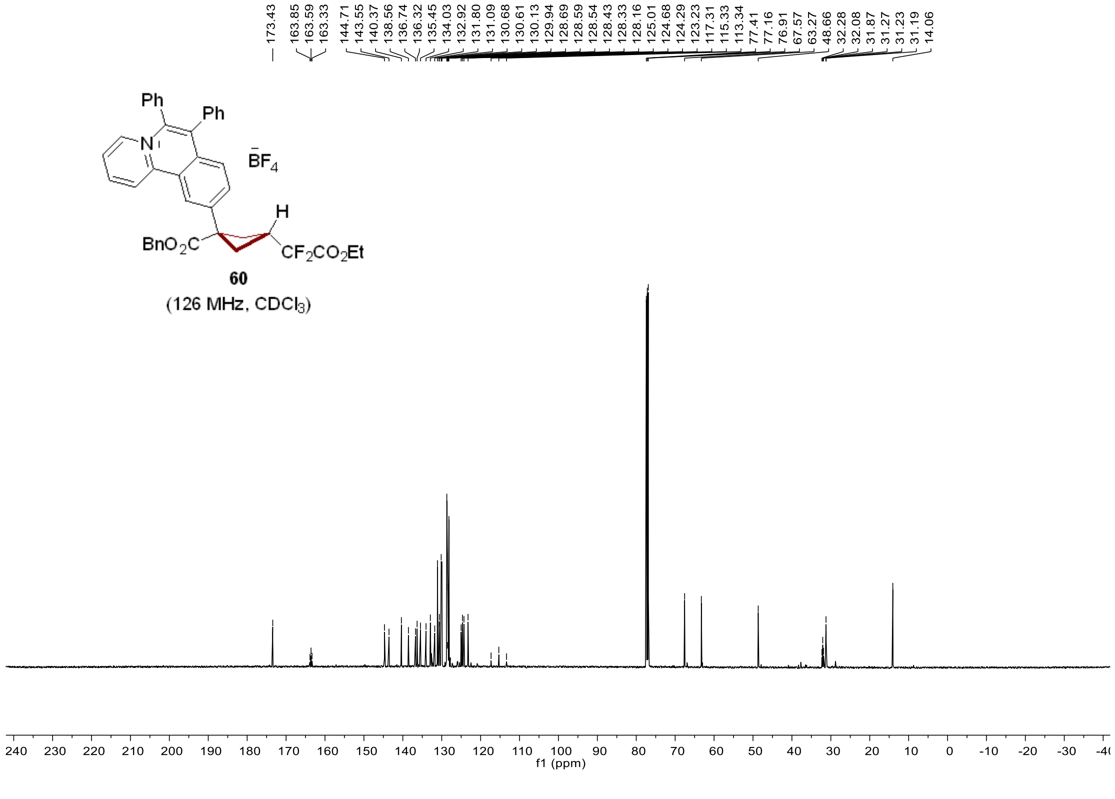
173.43
163.85
163.59
163.33
144.71
143.55
140.37
138.56
136.74
136.32
135.45
134.03
132.92
131.80
131.09
130.68
130.61
130.13
129.94
128.69
128.59
128.54
128.43
128.33
128.16
125.01
124.68
124.29
123.23
117.31
115.33
113.34
77.41
77.16
76.91
67.57
63.27
48.66
32.28
32.08
31.87
31.27
31.23
31.19
14.06
Ph
Ph
N
$\bar{B}F_4$
H
$BnO_2C$
$CF_2CO_2Et$
60
(126 MHz, $CDCl_3$)
240 230 220 210 200 190 180 170 160 150 140 130 120 110 100 90 80 70 60 50 40 30 20 10 0 -10 -20 -30 -4(
f1 (ppm)

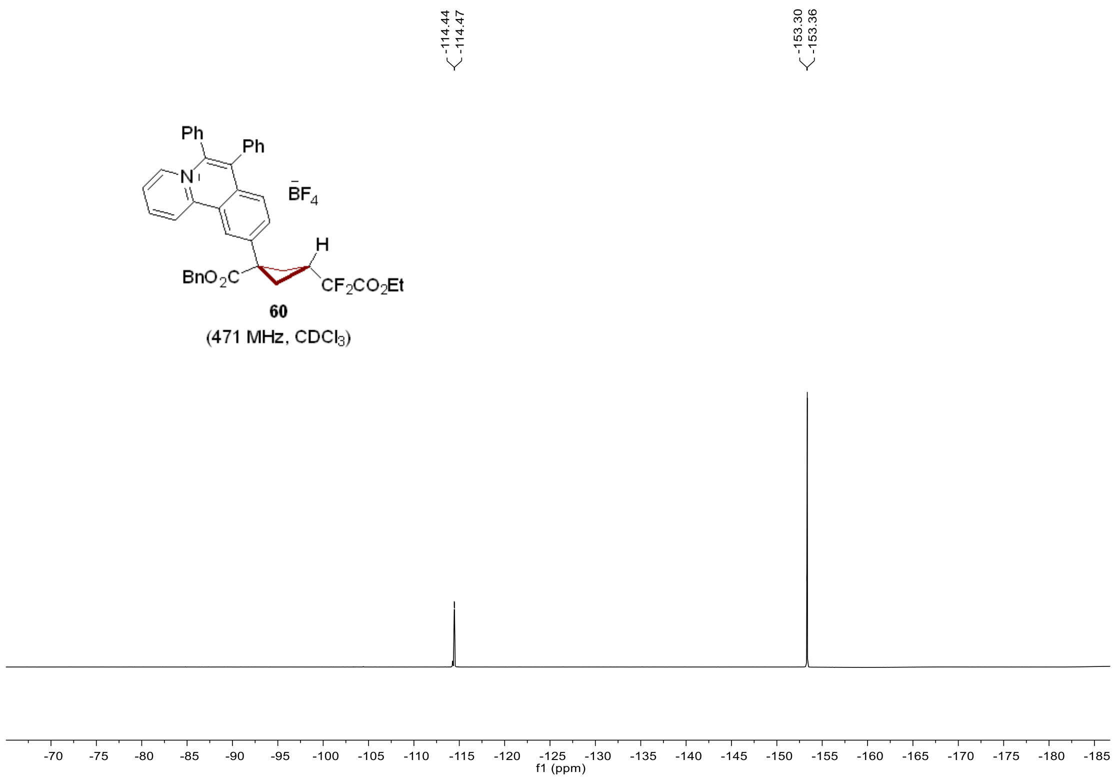
-114.44
-114.47
-153.30
-153.36
Ph
Ph
BF4
H
BnO2C
CF2CO2Et
60
(471 MHz, CDCl3)
f1 (ppm)

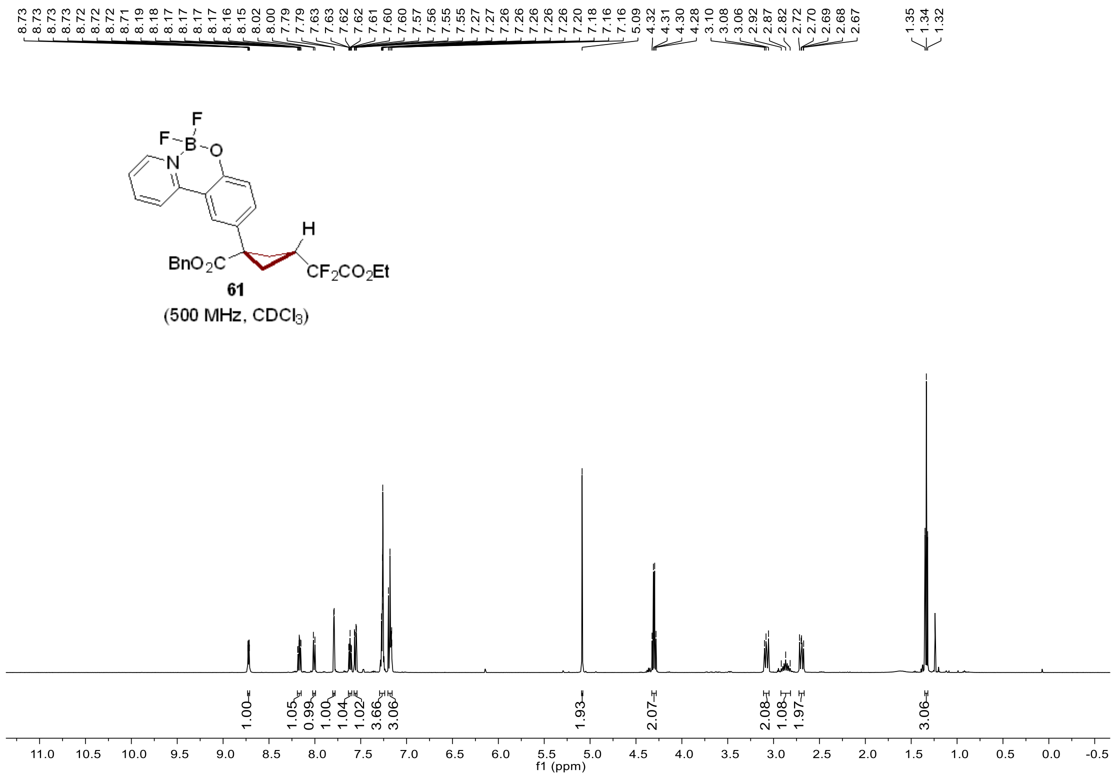
8.73 8.73 8.73 8.72 8.72 8.72 8.71 8.19 8.18 8.17 8.17 8.17 8.16 8.15 8.02 8.00 7.79 7.63 7.63 7.62 7.61 7.60 7.60 7.57 7.56 7.55 7.55 7.27 7.27 7.26 7.26 7.26 7.26 7.20 7.18 7.16 7.16 5.09 4.32 4.31 4.30 4.28 3.10 3.08 3.06 2.92 2.87 2.82 2.72 2.70 2.69 2.68 2.67
1.35 1.34 1.32
F
F
B
O
N
H
BnO2C
CF2CO2Et
61
(500 MHz, CDCl3)
1.00 1.05 0.99 1.00 1.04 1.02 3.66 3.06 1.93 2.07 2.08 1.08 1.97 3.06
f1 (ppm)

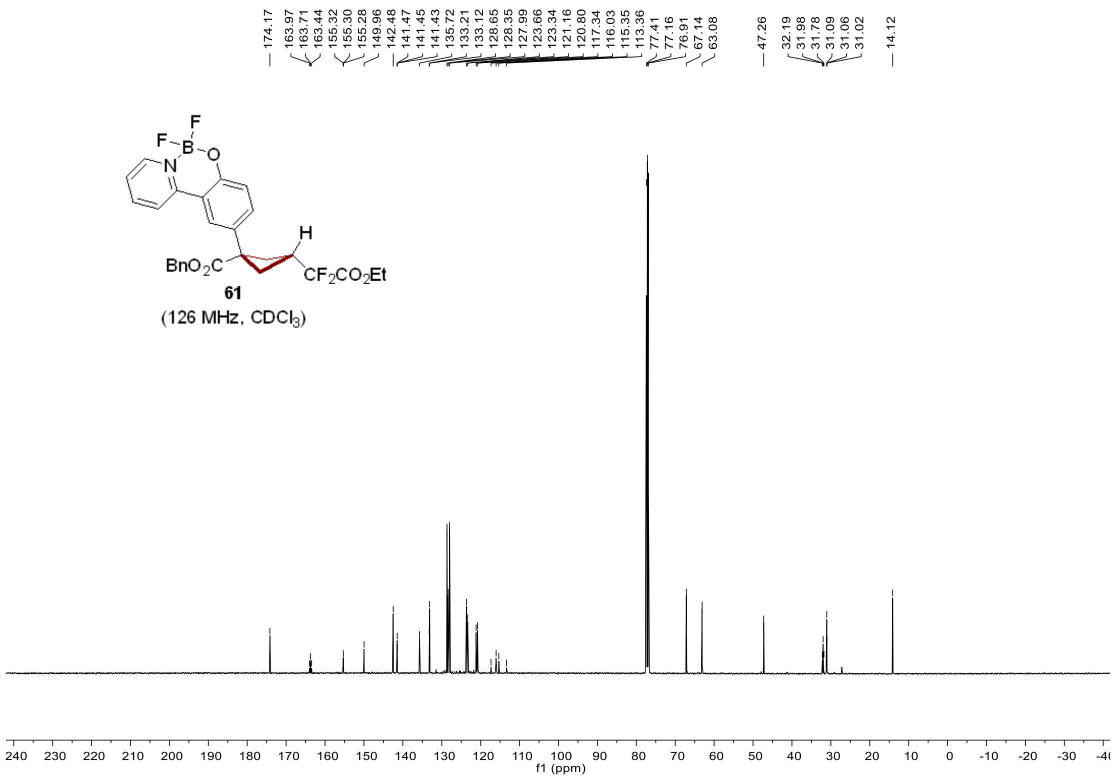

174.17
163.97
163.71
163.44
155.32
155.30
155.28
149.96
142.48
141.47
141.45
141.43
135.72
133.21
133.12
128.65
128.35
127.99
123.66
123.34
121.16
120.80
117.34
116.03
115.35
113.36
77.41
77.16
76.91
67.14
63.08
47.26
32.19
31.98
31.78
31.09
31.06
31.02
14.12
BnO2C
CF2CO2Et
61
(126 MHz, CDCl3)
f1 (ppm)


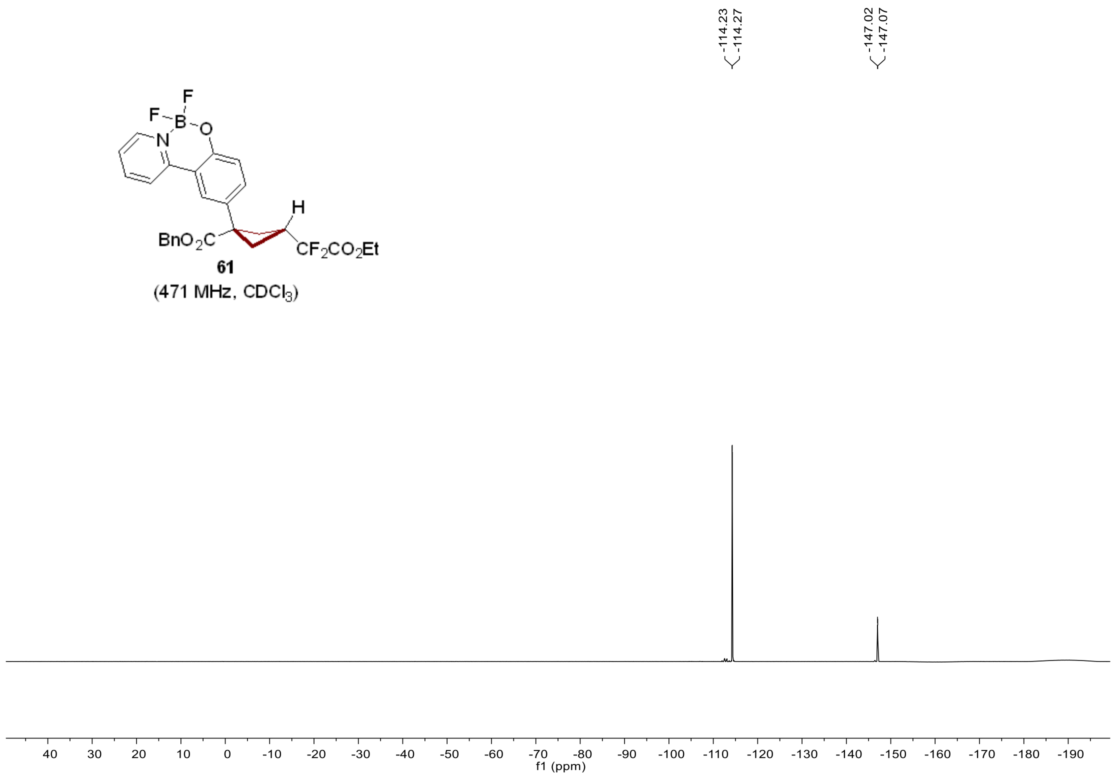

-114.23
-114.27
-147.02
-147.07
BnO2C
CF2CO2Et
61
(471 MHz, CDCl3)
f1 (ppm)

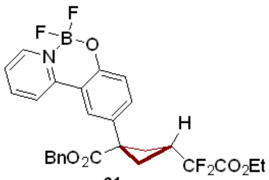

**61**
(161 MHz, $CDCl_3$)

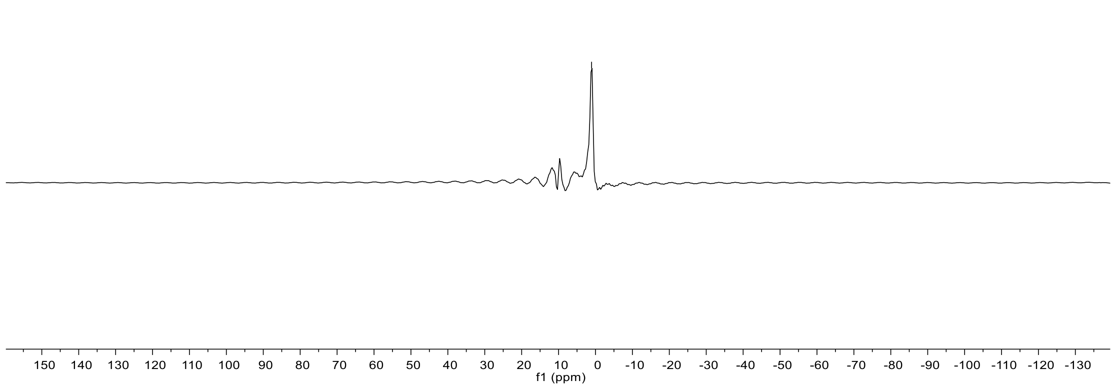

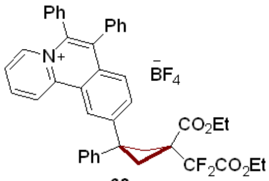

**62**
(500 MHz, $CDCl_3$)

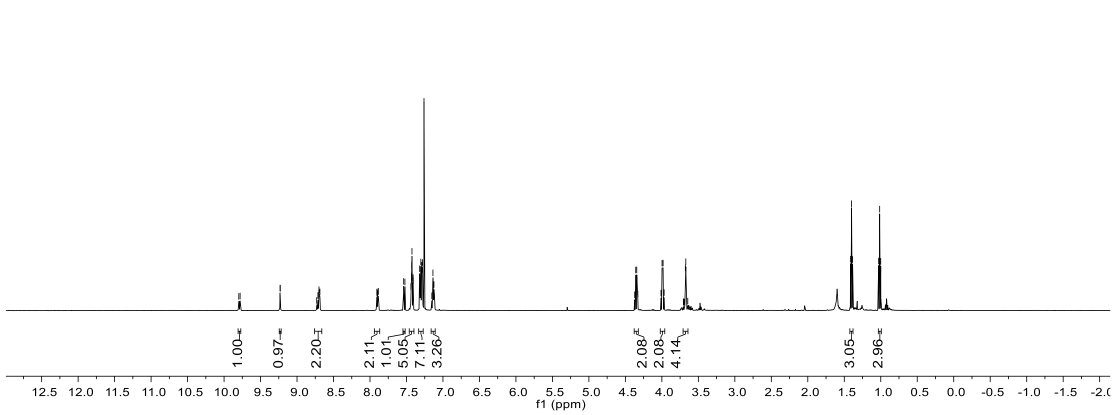

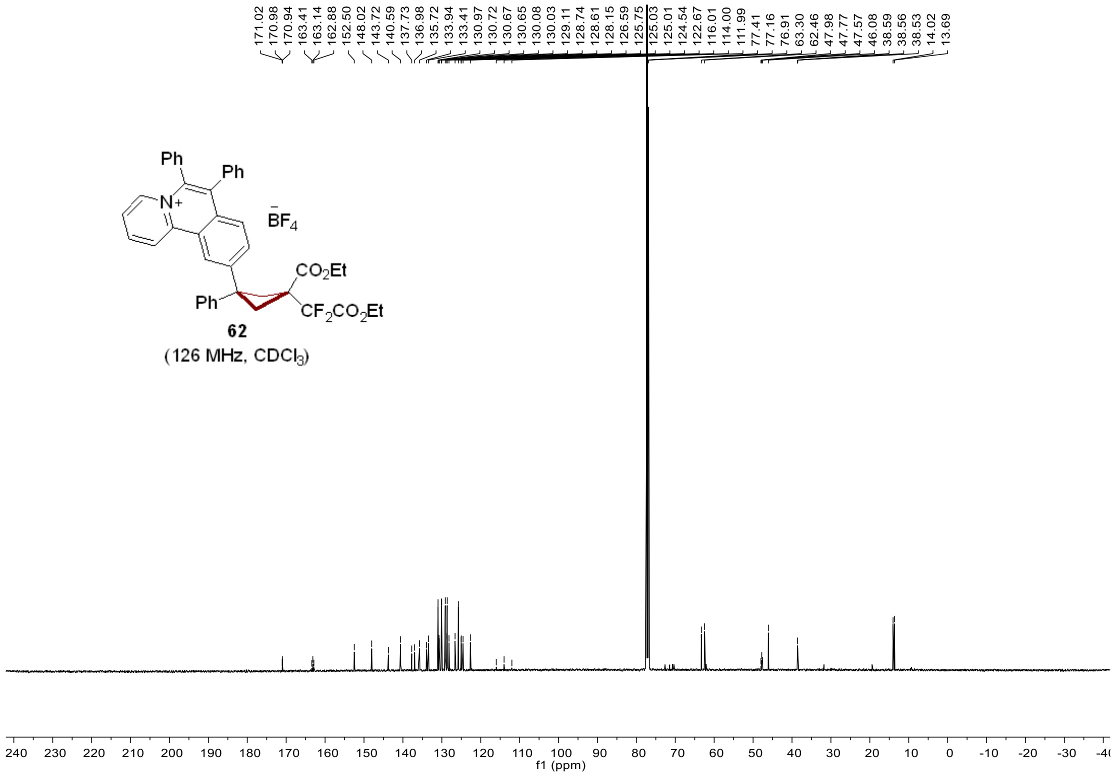


**62**

(126 MHz, $CDCl_3$)

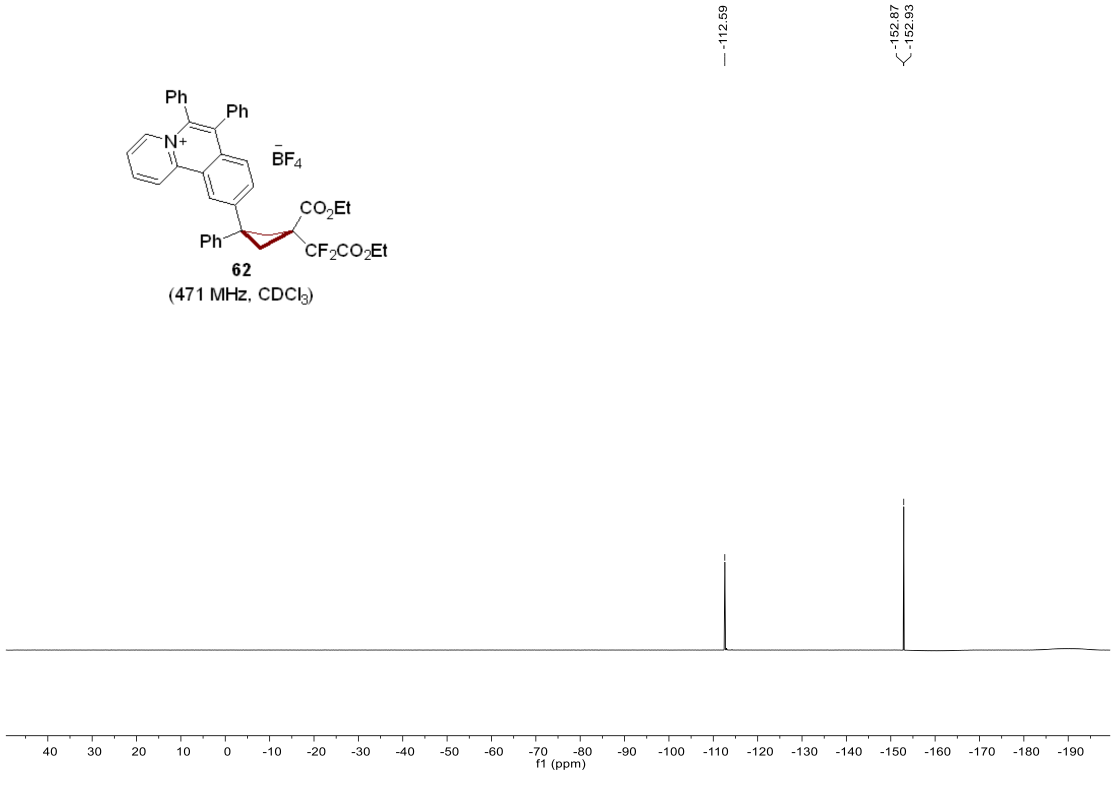


**62**

(471 MHz, $CDCl_3$)

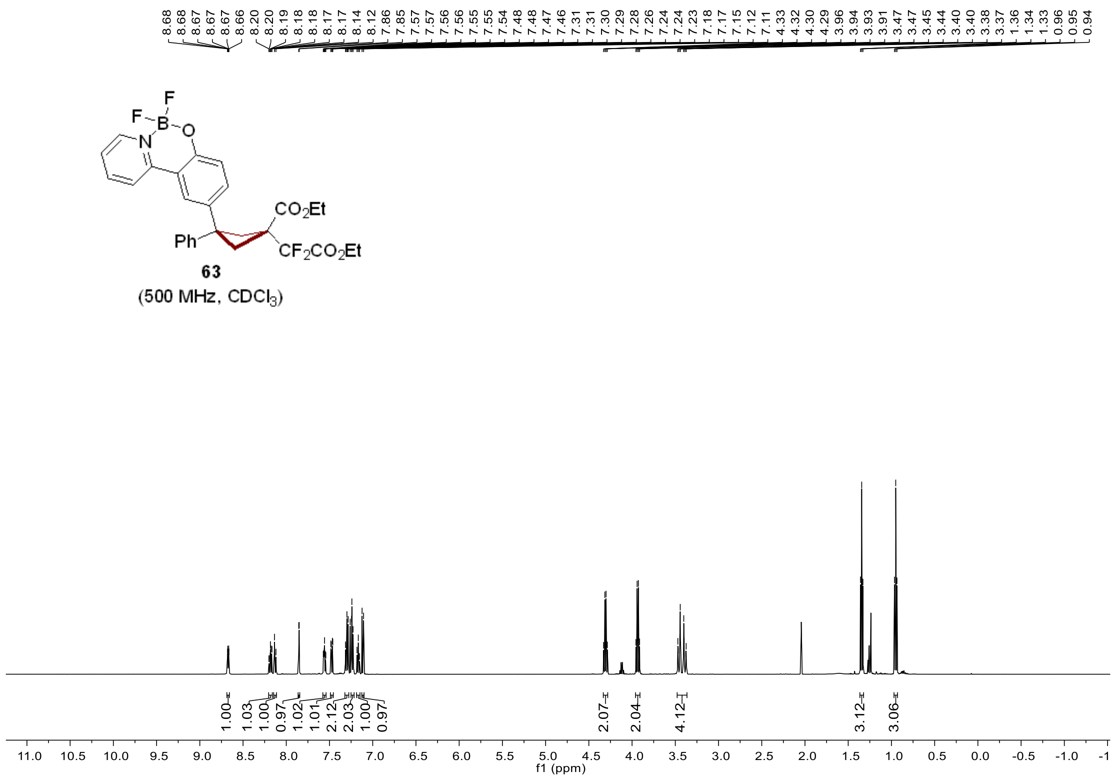
63
(500 MHz, CDCl3)
f1 (ppm)

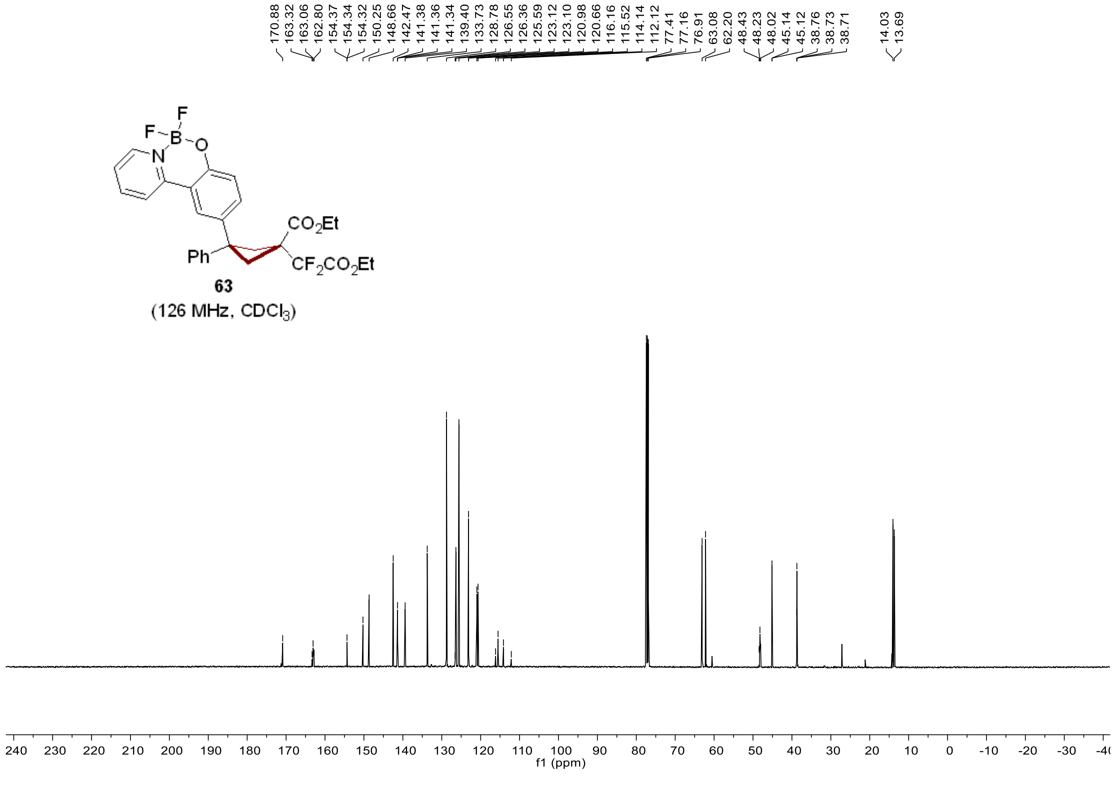
63
(126 MHz, CDCl3)
f1 (ppm)

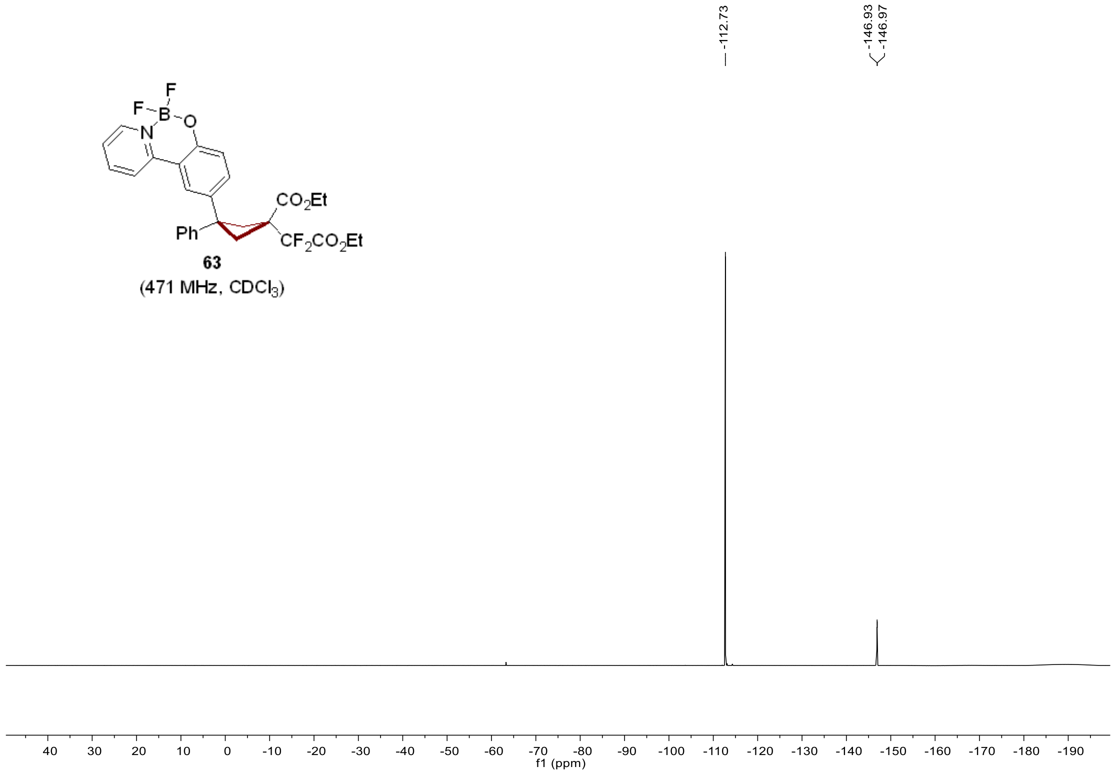
-112.73
-146.93
-146.97
63
(471 MHz, CDCl3)
f1 (ppm)

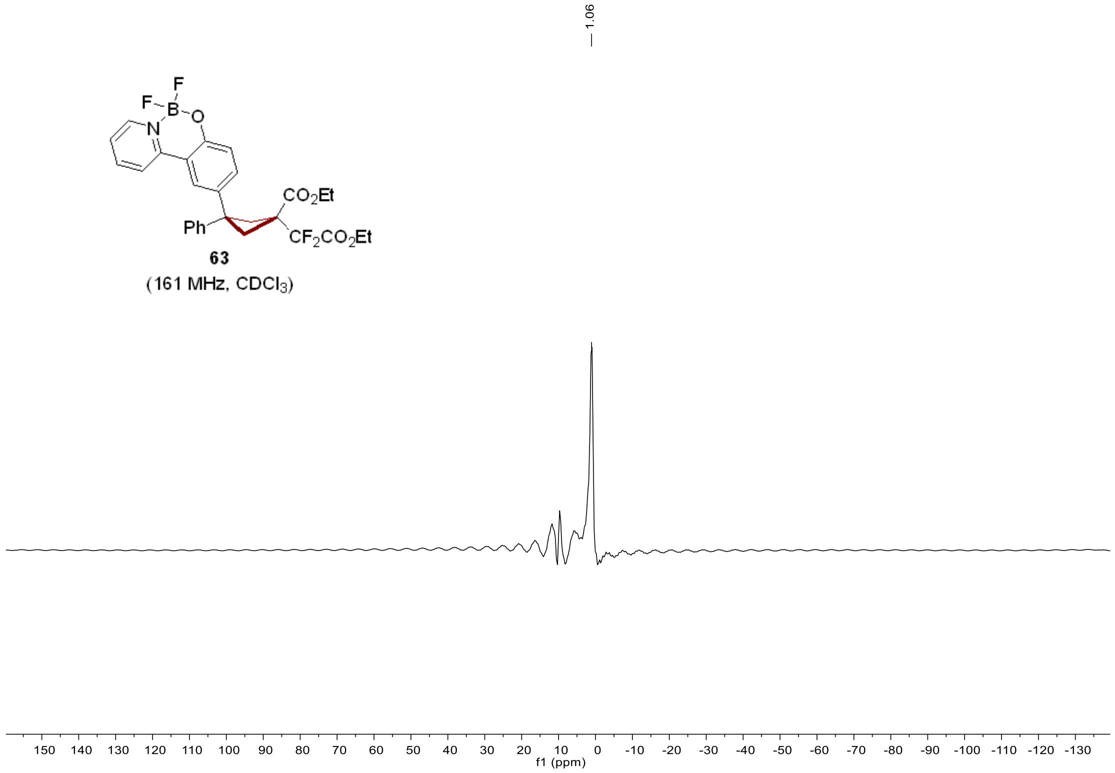
1.06
63
(161 MHz, CDCl3)
f1 (ppm)

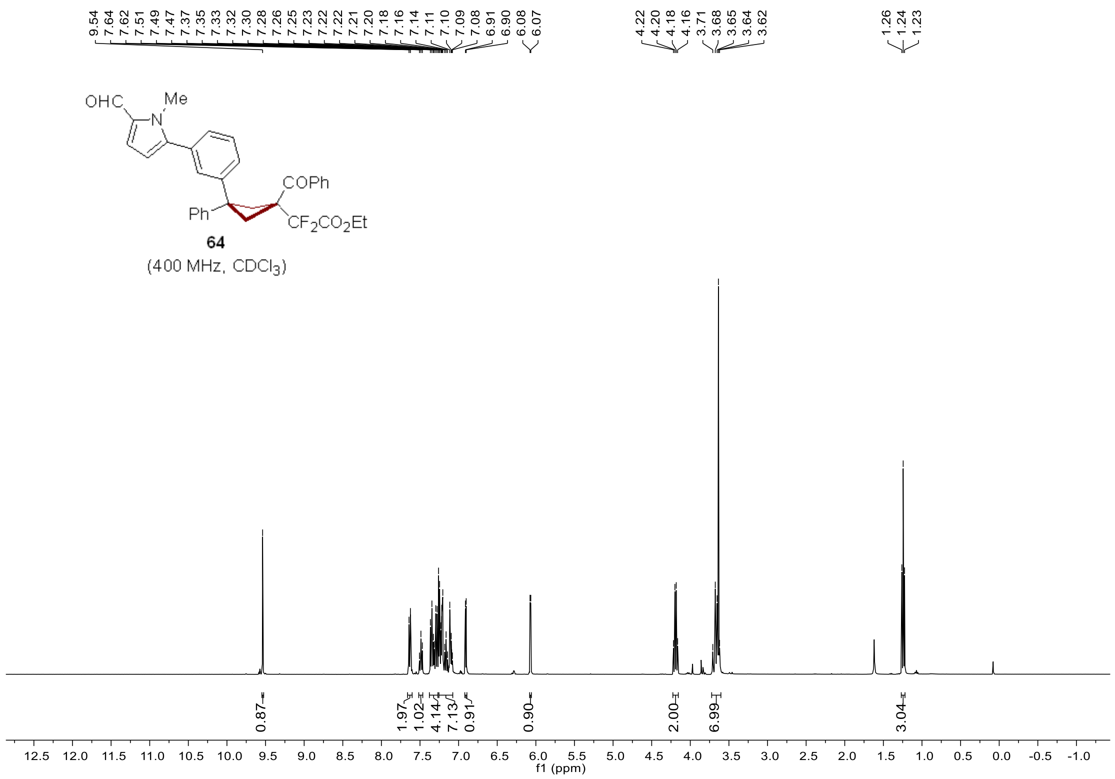
9.54 7.64 7.62 7.51 7.49 7.47 7.37 7.35 7.33 7.32 7.30 7.28 7.26 7.25 7.23 7.22 7.22 7.21 7.20 7.18 7.16 7.14 7.11 7.10 7.09 7.08 6.91 6.90 6.08 6.07
4.22 4.20 4.18 4.16 3.71 3.68 3.65 3.64 3.62
1.26 1.24 1.23
OHC
Me
N
COPh
Ph
$CF_2CO_2Et$
64
(400 MHz, $CDCl_3$)
0.87 1.97 1.02 4.14 7.13 0.91 0.90 2.00 6.99 3.04
12.5 12.0 11.5 11.0 10.5 10.0 9.5 9.0 8.5 8.0 7.5 7.0 6.5 6.0 5.5 5.0 4.5 4.0 3.5 3.0 2.5 2.0 1.5 1.0 0.5 0.0 -0.5 -1.0
f1 (ppm)

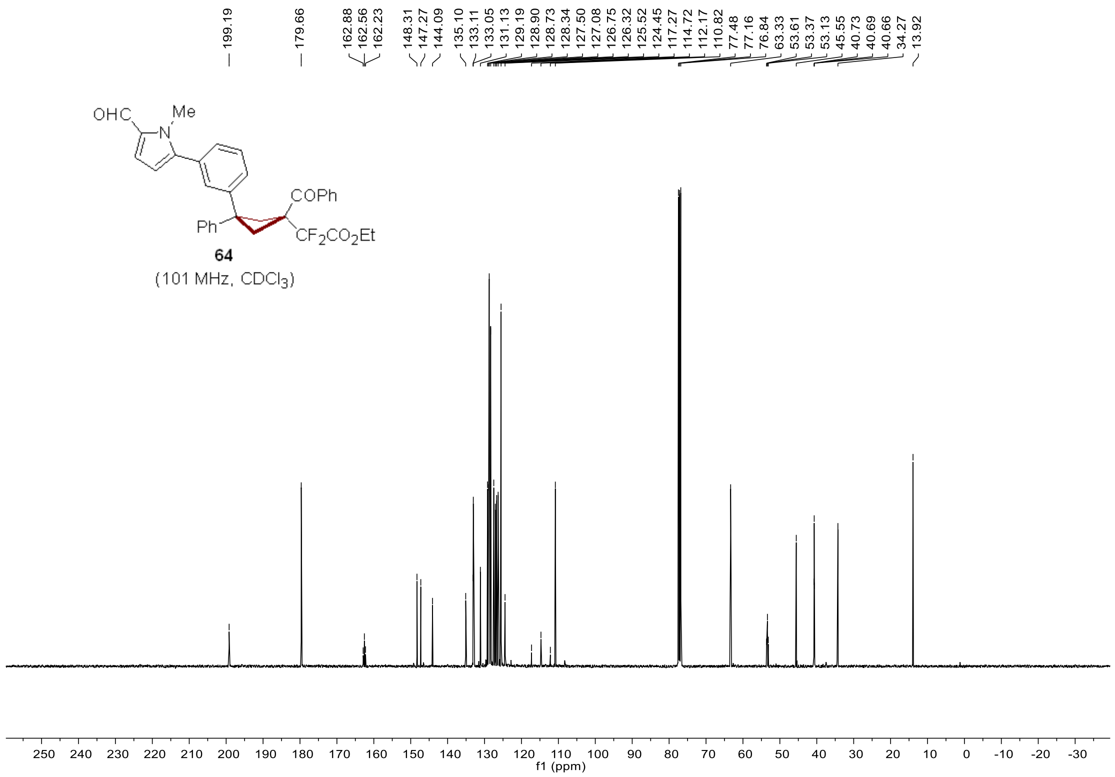
199.19
179.66
162.88 162.56 162.23
148.31 147.27 144.09 135.10 133.11 133.05 131.13 129.19 128.90 128.73 128.34 127.50 127.08 126.75 126.32 125.52 124.45 117.27 114.72 112.17 110.82 77.48 77.16 76.84 63.33 53.61 53.37 53.13 45.55 40.73 40.69 40.66 34.27 13.92
OHC
Me
N
COPh
Ph
$CF_2CO_2Et$
64
(101 MHz, $CDCl_3$)
250 240 230 220 210 200 190 180 170 160 150 140 130 120 110 100 90 80 70 60 50 40 30 20 10 0 -10 -20 -30
f1 (ppm)

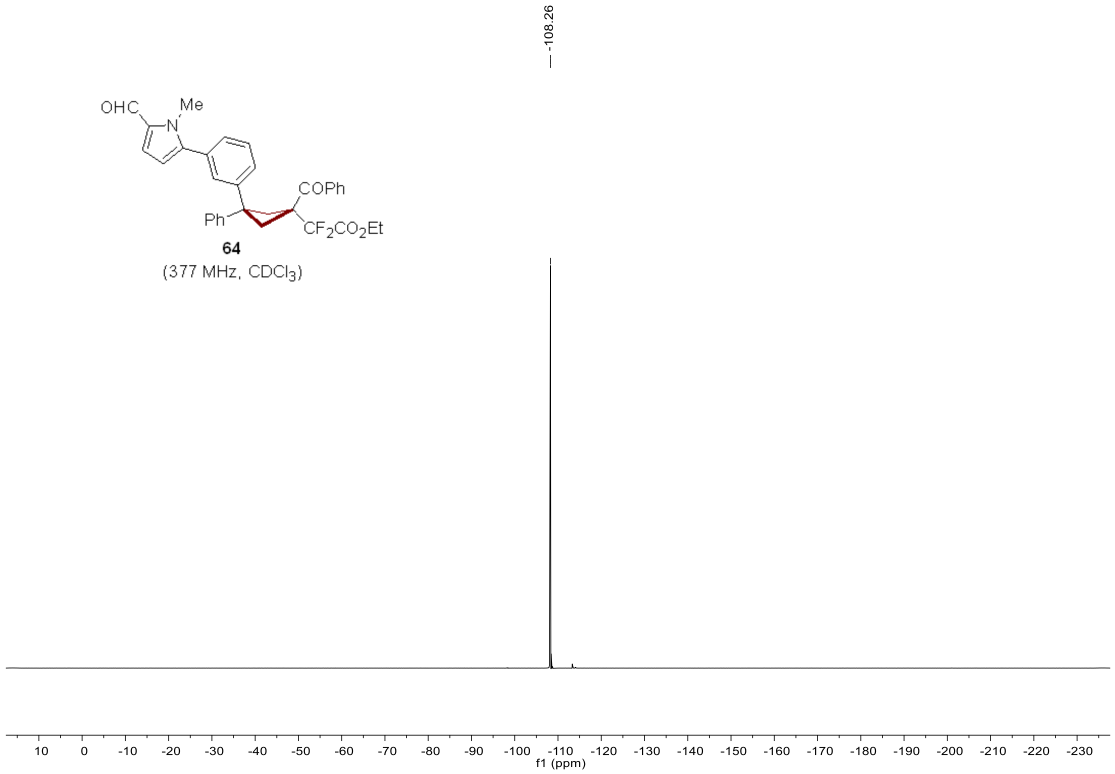

-108.26
OHC
N
Me
COPh
Ph
$CF_2CO_2Et$
64
(377 MHz, $CDCl_3$)
f1 (ppm)


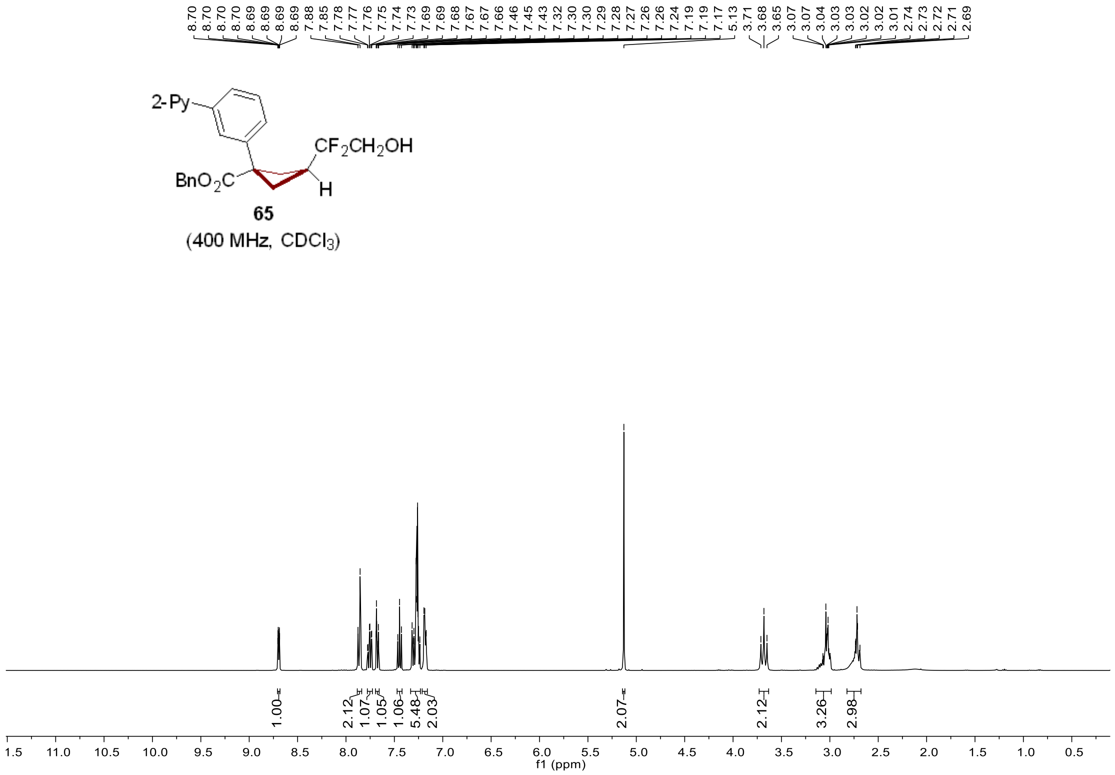

2-Py
$CF_2CH_2OH$
$BnO_2C$
H
65
(400 MHz, $CDCl_3$)
1.00
2.12
1.07
1.05
1.06
5.48
2.03
2.07
2.12
3.26
2.98
f1 (ppm)

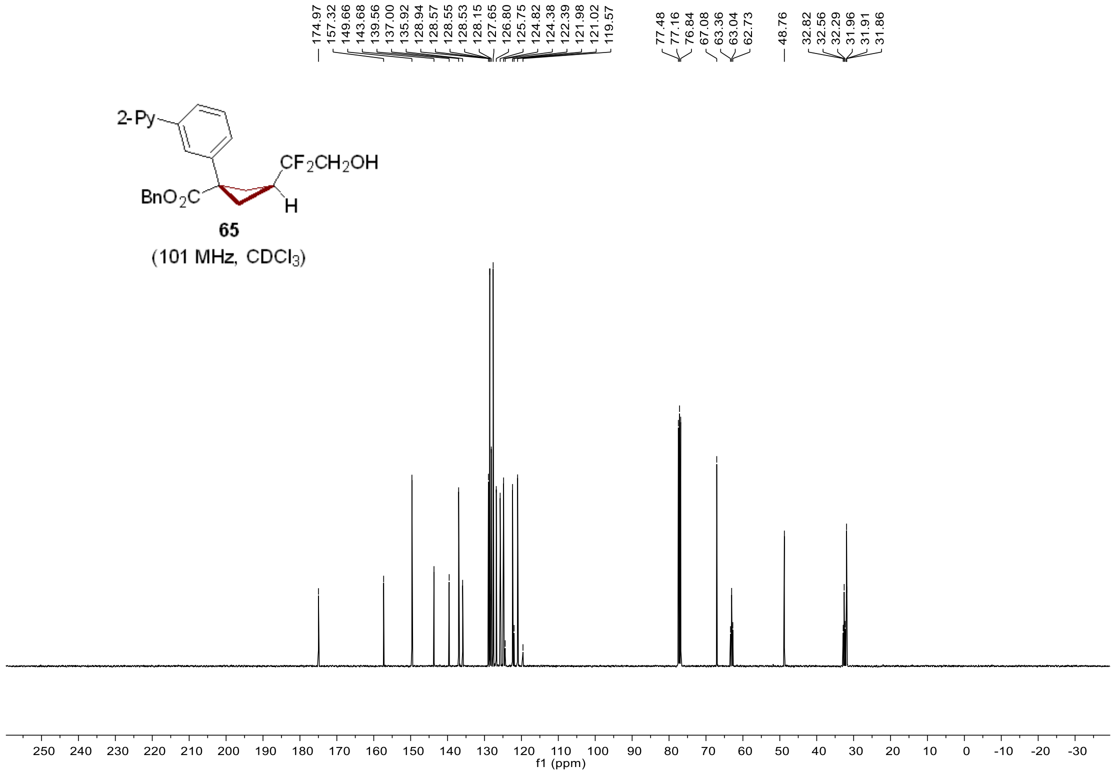


**65**

(101 MHz, $CDCl_3$)

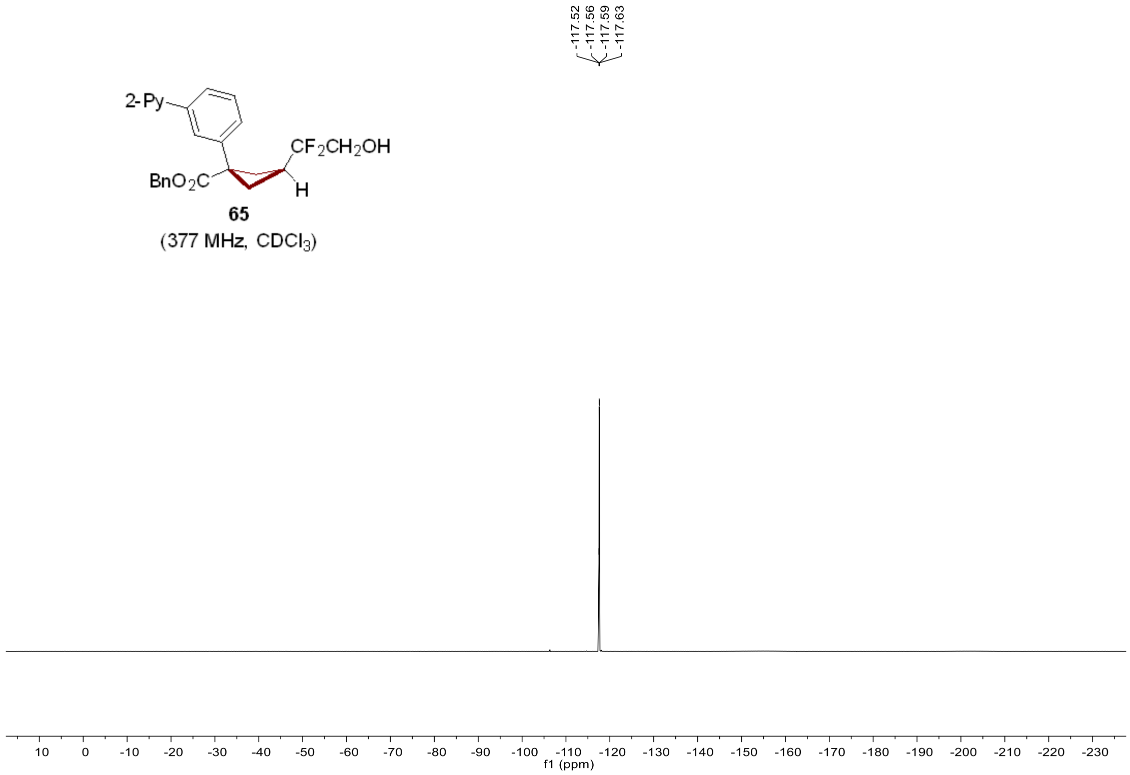


**65**

(377 MHz, $CDCl_3$)
